# Supplementary material for: Harnessing nitroarenes as nitrogen and oxygen sources for general oxo-aminomethylation of alkenes
Source: Nat Commun. 2025 Nov 14;16:9997. doi: 10.1038/s41467-025-64957-z (PMC12618553; doi:10.1038/s41467-025-64957-z)
Supplement: Supplementary file 1 — Supplementary Information [file 41467_2025_64957_MOESM1_ESM.pdf]

Supplementary Information

Harnessing nitroarenes as nitrogen and oxygen sources for general oxo-aminomethylation of alkenes

Ting Zhou[1], Jing Nie[1], Chi Wai Cheung[1,2]*, Jun-An Ma[1]*

[1]Department of Chemistry, State Key Laboratory of Synthetic Biology, Tianjin University, Tianjin 300072, P. R. of China

[2]State Key Laboratory of Synthetic Chemistry and Department of Chemistry, The Chinese University of Hong Kong, Shatin, New Territories, Hong Kong 999077, P. R. of China

*Corresponding E-mails: cw.cheung@cuhk.edu.hk (C.W.C.); majun_an68@tju.edu.cn (J.-A.M.)

Table of contents

General Considerations	S3
General Analytical Information	S3
General Reagent Information	S3
General Manipulation Considerations	S4
Supplementary Results	S5
Synthesis of starting materials	S5
Synthesis of 4-nitrophenyl 2-(4-isobutylphenyl)propanoate (***N38***)	S5
Synthesis of trifluoromethyl alkenes (***O25***, ***O27***-***O29***).	S5
Synthesis of (cyclopropylidenemethyl)benzene (***O33***)	S6
Synthesis of 1-phenyl-3-(phenylamino)propan-1-one (**111**)	S7
Synthesis of methyl 2-(*p*-tolyl)isoxazolidine-5-carboxylate) (**114**)	S7
Optimization of reaction conditions (**Table S1**)	S9
Optimization of reaction conditions using non-gaseous alkenes as reactant (**Fig. S2**)	S12
Mechanistic study	S13
**(a)** Probing water as the hydroxyl source by adding ^{18}O-water (**Fig. S3**)	S13
**(b)** Probing *γ*-arylamino cabocation as possible intermediate by adding me thanol as nucleophile (**Fig. S4**)	S16
**(c)** Probing *N*-(3-oxo alkyl) aniline as possible intermediate (**Fig. S5**)	S18
**(d)** Probing the nitrogen-based intermediates for reaction (**Fig. S6**)	S19
**(e)** Probing the reaction intermediates at the initial stage of reaction (**Fig. S7**)	S21
**(f)** Radical trap experiment (**Fig. S8**)	S25
**(g)** Probing the *N*–O cleavage step of isoxazolidine (**Fig. S9**)	S26
Substrate scope study and gram scale synthesis of **11** and **49**	S31
Diverse Functionalization of the *N*-arylamino alcohol products	S75
Stern-Volmer quenching studies (**Figs. S11–S16**)	S89

X-ray crystallographic analysis	S93
NMR Spectra	S101
References	S278

General Considerations

General Analytical Information. ^{1}H, ^{13}C and ^{19}F NMR spectra were recorded on Bruker AV 400 MHz instrument at 400 MHz (^{1}H NMR), 101 MHz (^{13}C NMR), and 376 MHz (^{19}F NMR, comp. pulse decoupling), or on Bruker AV 500 MHz instrument at 500 MHz (^{1}H NMR), 125 MHz (^{13}C NMR), and 470 MHz (^{19}F NMR, comp. pulse decoupling), or on Bruker AV 800 MHz instrument at 800 MHz (^{1}H NMR), 201 MHz (^{13}C NMR), and 753 MHz (^{19}F NMR, comp. pulse decoupling). All ^{1}H NMR spectra were measured in parts per million (ppm) downfield from tetramethylsilane (TMS, 0 ppm), or were measured relative to the residual proton signals of d_1-chloroform ($CDCl_3$, 7.26 ppm) or methanol-d_4 (CD_3OD-d_4, 3.31 ppm) or dimethyl sulfoxide- d_6 (DMSO-d_6, 2.50 ppm). All ^{13}C NMR spectra were reported in ppm relative to residual carbon signals of $CDCl_3$ (77.16 ppm) or CD_3OD-d_4 (49.00 ppm) or DMSO-d_6 (39.53 ppm) were obtained with ^{1}H decoupling. All ^{19}F NMR spectra were measured in parts per million (ppm) relative to trichlorofluoromethane ($CFCl_3$, 0 ppm). Coupling constants (*J*) are reported in hertz (Hz). Multiplicity was indicated as follows: s (singlet), d (doublet), t (triplet), q (quartet), and m (multiplet). High resolution mass spectrometry (HRMS) spectra were obtained on a Bruker micrOTOF-QII instrument. GC-MS analyses were performed on a Thermo Scientific Model Trace 1300 instrument. X-ray structural analysis was conducted on a Bruker APEX-II CCD instrument. Thin-layer chromatography (TLC) was performed on precoated GF254 silica gel plates (Qingdao Marine Chemical Inc.) and compounds were visualized with a UV light at 254 nm. Flash chromatography for purification of compounds were carried out using silica gel (200–300 mesh, Qingdao Marine Chemical Inc.). Stern-Volmer quenching experiments were performed using Edinburgh photofluorescence spectrometer FLS1000.

General Reagent Information. Unless otherwise noted, commercially available materials were used without prior purification. All known starting materials were synthesized according to the literature procedures. Anhydrous *N*-methyl-2-pyrrolidone (NMP) was purchased from J&K Scientific. 3,4,5,6-tetrakis(carbazol-9-yl)-1,2-dicyanobenzene (4CzPN, **PC4**, 97% purity) and [Bipyridine]nickel(II) dichloride (Ni(Bipy)Cl_2, 99% purity) were purchased from Bidepharm. Diethyl 1,4-dihydro-2,6-dimethyl-3,5-pyridinedicarboxylate (Hantzsch ester, **HE**, 98% purity) and *N*,*N*-

dimethylcyclohexylamine (99.5% purity) were purchased from HEOWNS. 3,3,3,-Trifluoropropene (***O1***) was purchased from Shang Fluoro.

General Manipulation Considerations. Unless otherwise noted, all manipulations for photochemical reaction were performed in Teflon screw-capped Schlenk tubes. Flash chromatography for purification of compounds were carried out using silica gel (200–300 mesh, Qingdao Marine Chemical Inc.). Preparative thin-layer chromatography (PTLC) for purification of compounds were carried out using preparative TLC (Rushan Hailan Experimental Equipment Inc.). Thin-layer chromatography (TLC) was performed on precoated GF254 silica gel plates (Qingdao Marine Chemical Inc.) and compounds were visualized with a UV light at 254 nm. The eluents used for column chromatography, PTLC and TLC were presented as ratios of solvent volumes (v/v). Ethyl acetate (EtOAc) was used as the extraction solvent to extract the products attached on the silica gel of PTLC plate. Yields reported in the publication are of isolated yields unless otherwise noted. All new starting materials and all products obtained from the photochemical reactions were characterized by ^{1}H, ^{13}C and ^{19}F NMR spectroscopies (in case the compounds contained F atoms) and high-resolution mass spectrometry (HRMS). The blue LEDs (455–460 nm, 30 W) setup for photocatalytic reactions in 20 mL Schlenk tubes were purchased from Taobao.

Supplementary Results

Synthesis of starting materials

Synthesis of 4-nitrophenyl 2-(4-isobutylphenyl)propanoate (*N38*)

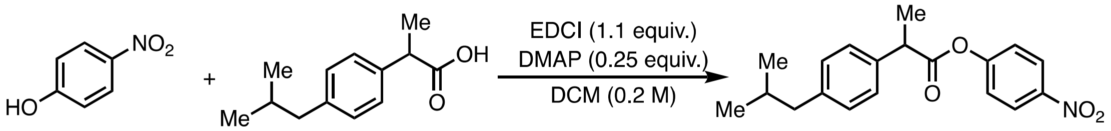

To a 250 mL round bottom flask equipped with a magnetic stirring bar was added, 4-nitrophenol (1.0 equiv., 10.0 mmol, 1.4 g), ibuprofen acid (1.0 equiv., 10.0 mmol, 2.1 g), EDCI (1.1 equiv., 11.0 mmol, 2.1 g) and DMAP (0.25 equiv., 2.50 mmol, 305.4 mg) were dissolved in DCM (50 mL, 0.2 M). The mixture was stirred at room temperature for 12 h. Upon completion of the reactions (TLC showed complete consumption of starting material). The reaction was concentrated in *vacuo*. The residue was purified by column chromatography on silica gel (petroleum ether/ethyl acetate = 10:1) to obtain the **N38** as a white solid (1.8 g, 56%).

4-nitrophenyl 2-(4-isobutylphenyl)propanoate (*N38*). ^{1}H NMR (500 MHz, $CDCl_3$) δ 8.22 (d, J = 8.7 Hz, 2H), 7.28(d, J = 7.7 Hz, 2H), 7.19 – 7.15 (m, 4H), 3.96 (q, J = 7.2 Hz, 1H), 2.47 (d, J = 7.1 Hz, 2H), 1.89 – 1.84 (m, 1H), 1.62 (d, J = 7.1 Hz, 3H), 0.91 (d, J = 6.7 Hz, 6H). **^{13}C NMR** (126 MHz, $CDCl_3$) δ 172.4, 155.8, 145.4, 141.3, 136.6, 129.8, 127.3, 125.2, 122.4, 45.4, 45.1, 30.3, 22.5, 18.5. **HRMS** (ESI) m/z: $[M+Na]^+$ Calcd for $C_{19}H_{21}NO_4Na^+$ 350.1368; Found 350.1364.

Synthesis of trifluoromethyl alkenes (*O25*, *O27*-*O29*).[1]

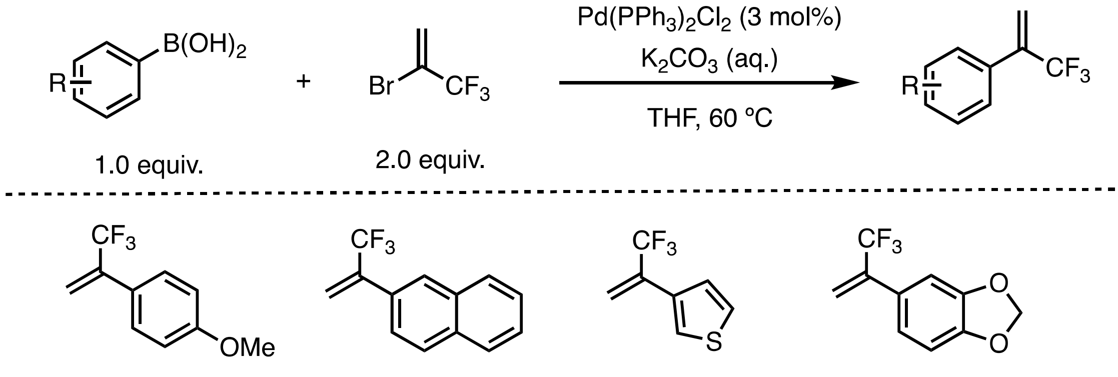

O25 ***O27*** ***O28*** ***O29***

To a Schlenk tube equipped with a stirring bar, arylboronic acid (1.0 equiv., 10 mmol) and $Pd(PPh_3)_2Cl_2$ (3 mol%, 0.3 mmol, 210.6 mg) were added. The vessel was evacuated and filled with argon (three times), and then aqueous K_2CO_3 (2.0 M, 20 mL) and THF (30 mL) were added. After adding 2-bromo-3,3,3-trifluoropropene (2.0 equiv., 20 mmol, 2.1 mL), the resulting mixture was stirred at 60 °C for 12 h. Upon completion of the reactions (TLC showed complete consumption of starting material). The reaction was concentrated in *vacuo*. The residue was purified by column chromatography on silica gel (petroleum ether) to obtain corresponding trifluoromethyl alkenes (***O25***, ***O27***-***O29***). The spectroscopic data of these compounds are consistent with the results reported in the literature.

Synthesis of (cyclopropylidenemethyl)benzene (*O33*)[2]

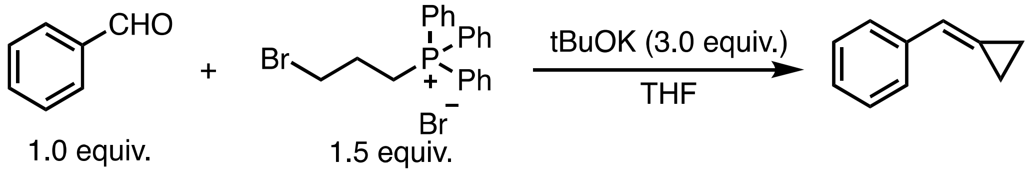

To a suspension of 1-bromopropyl-3-triphenylphoshonium bromide (1.5 equiv., 7.5 mmol, 3.5 g) in THF (15 mL) under argon atmosphere was added a solution of KO^{t}Bu (3.0 equiv., 15 mmol, 1.7 g) in THF (15 mL) via a syringe, and the reaction mixture turned to bright orange. The reaction mixture was then heated at reflux with an oil bath for 90 min and the Benzaldehyde (1.0 equiv., 5 mmol, 0.5 mL) was then added. The reaction mixture was continued at reflux for 2 h, and the reaction mixture was cooled down to room temperature. The reaction mixture was then poured in a Erlenmeyer flask

containing a large amount of pentane (50 mL) and the precipitates were filtered. The filtrate was concentrated in *vacuo*. The residue was purified by column chromatography on silica gel (petroleum ether) to obtain ***O33*** as a colorless liquid (591.5 mg, 91%). The spectroscopic data of this compound is consistent with the results reported in the literature.

Synthesis of 1-phenyl-3-(phenylamino)propan-1-one (111)[7]

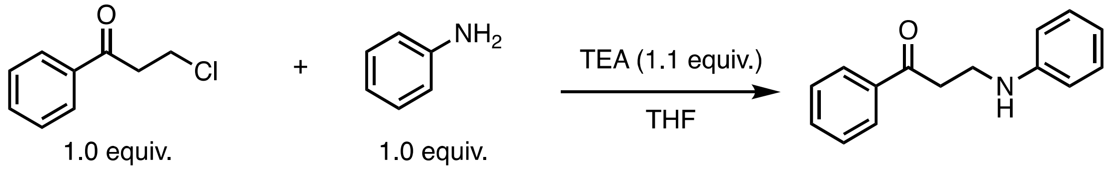

To a 50 mL round bottom flask equipped with a magnetic stirring bar was added, 3-chloro-1-phenylpropan-1-one (1.0 equiv., 5.0 mmol, 843.1 mg), aniline (1.0 equiv., 5.0 mmol, 456 μL), and TEA (1.1 equiv., 5.5 mmol, 0.8 mL) were dissolved in THF (10 mL, 0.5 M). The mixture was stirred at room temperature for 24 h. Upon completion of the reactions (TLC showed complete consumption of starting material). The reaction was concentrated in *vacuo*. The residue was purified by column chromatography on silica gel (petroleum ether/ethyl acetate = 9:1) to obtain the **111** as a white solid (889.9 mg, 79%). The spectroscopic data of this compound is consistent with the results reported in the literature.

1-phenyl-3-(phenylamino)propan-1-one (111). ^{1}**H NMR** (500 MHz, $CDCl_3$) δ 7.95 (d, J = 7.9 Hz, 2H), 7.59 – 7.56 (m, 1H), 7.46 (t, J = 7.5 Hz, 2H), 7.18 (t, J = 7.8 Hz, 2H), 6.75 – 6.64 (m, 3H), 4.12 (brs, 1H), 3.62 (t, J = 6.1 Hz, 2H), 3.29 (t, J = 6.0 Hz, 2H).

Synthesis of methyl 2-(*p*-tolyl)isoxazolidine-5-carboxylate (114) [3]

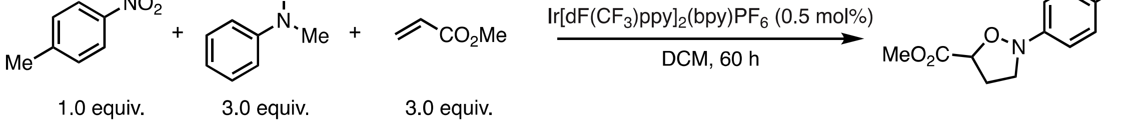

An oven-dried, transparent 100 mL Schlenk tube equipped with a stir bar was sequentially charged with 1-methyl-4-nitrobenzene (1.0 equiv., 1.0 mmol, 137.1 mg),

$Ir[dF(CF_3)ppy]_2(bpy)PF_6$ (0.5 mol %, 0.005 mmol, 5.0 mg). The tube was evacuated *in vacuo* and then backfilled with argon for three times. Dry DCM (20.0 mL, 0.05 M). was transferred into the tube via a syringe. Subsequently, *N*,*N*-dimethylaniline (3.0 equiv., 3.0 mmol, 0.4 mL) and methyl acrylate (3.0 equiv., 3.0 mmol, 0.3 mL) were transferred into the tube via syringe. The resulting mixture was stirred under an argon atmosphere was stirred for 60 h. Upon completion of the reactions (TLC showed complete consumption of starting material). The mixture was concentrated in *vacuo*. The residue was purified by column chromatography on silica gel (petroleum ether/ethyl acetate = 10:1) to obtain **114** as a yellow (137.2 mg, 62%), m.p. 41 – 43 ºC.
Methyl 2-(*p*-tolyl)isoxazolidine-5-carboxylate (114). **^{1}H NMR** (500 MHz, $CDCl_3$) δ 7.09 (d, J = 7.8 Hz, 2H), 6.99 (t, J = 7.4 Hz, 2H), 4.75 – 4.18 (m, 1H), 3.33 – 4.72 (m, 1H), 3.79 (s, 3H), 3.60 – 3.50 (m, 2H), 2.53– 2.49 (m, 2H), 2.29 (s, 3H). **^{13}C NMR** (126 MHz, $CDCl_3$) δ 171.8, 148.4, 132.2, 129.5, 115.9, 75.3, 53.8, 52.6, 32.3, 20.8. **HRMS** (ESI) m/z: $[M+H]^+$ Calcd for $C_{12}H_{16}NO_3^+$ 222.1130; Found 222.1127.

Optimization of reaction conditions

Table S1. Optimization of the oxo-aminomethylation reaction of 3,3,3-trifluoropropene.

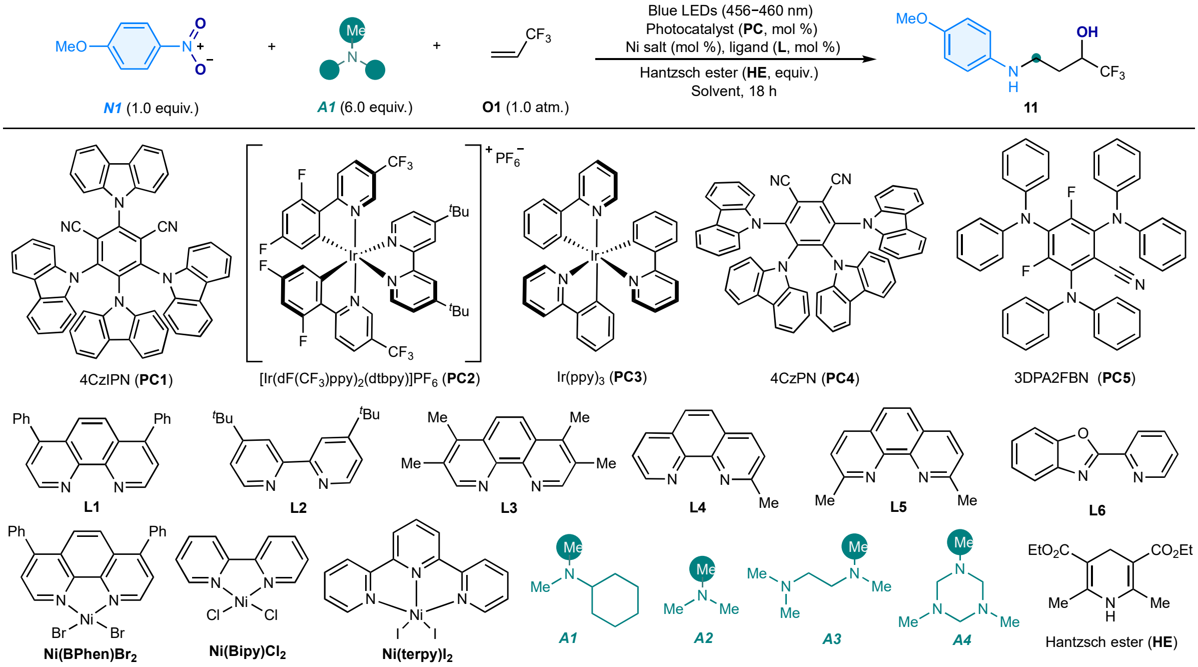

Entry	PC (mol %)	Ni salt (mol %) and ligand (mol %)	Amine (A)	HE (equiv.)	Solvent	Yield/%[a]
1	**PC1** (5)	$Ni(NO_3)_2·6H_2O$ (20), **L1** (20)	***A1***	2	1,4-Dioxane	20
2	**PC1** (5)	$Ni(NO_3)_2·6H_2O$ (20), **L1** (20)	***A1***	2	MeCN	trace
3	**PC1** (5)	$Ni(NO_3)_2·6H_2O$ (20), **L1** (20)	***A1***	2	DMF	54
4	**PC1** (5)	$Ni(NO_3)_2·6H_2O$ (20), **L1** (20)	***A1***	2	MeOH	18
5	**PC1** (5)	$Ni(NO_3)_2·6H_2O$ (20), **L1** (20)	***A1***	2	NMP	56
6	**PC1** (5)	$Ni(NO_3)_2·6H_2O$ (20), **L2** (20)	***A1***	2	NMP	31
7	**PC1** (5)	$Ni(NO_3)_2·6H_2O$ (20), **L3** (20)	***A1***	2	NMP	52
8	**PC1** (5)	$Ni(NO_3)_2·6H_2O$ (20), **L4** (20)	***A1***	2	NMP	30
9	**PC1** (5)	$Ni(NO_3)_2·6H_2O$ (20), **L5** (20)	***A1***	2	NMP	trace
10	**PC1** (5)	$Ni(NO_3)_2·6H_2O$ (20), **L6** (20)	***A1***	2	NMP	37
11	**PC1** (5)	$Ni(BF_4)_2·4H_2O$ (20), **L1** (20)	***A1***	2	NMP	62
12	**PC1** (5)	$Ni(acac)_2$ (20), **L1** (20)	***A1***	2	NMP	34
13	**PC1** (5)	$Ni(dme)Br_2$ (20), **L1** (20)	***A1***	2	NMP	39
14	**PC1** (5)	$NiBr_2$ (20), **L1** (20)	***A1***	2	NMP	36
15	**PC1** (5)	$Ni(BPhen)Br_2$ (20)	***A1***	2	NMP	57
16	**PC1** (5)	$Ni(Bipy)Cl_2$ (20)	***A1***	2	NMP	68
17	**PC1** (5)	$Ni(terpy)I_2$ (20)	***A1***	2	NMP	58
18	**PC2** (5)	$Ni(Bipy)Cl_2$ (20)	***A1***	2	NMP	46
19	**PC3** (5)	$Ni(Bipy)Cl_2$ (20)	***A1***	2	NMP	Trace
20	**PC4** (5)	$Ni(Bipy)Cl_2$ (20)	***A1***	2	NMP	70
21	**PC5** (5)	$Ni(Bipy)Cl_2$ (20)	***A1***	2	NMP	Trace
22	**PC4** (5)	$Ni(Bipy)Cl_2$ (20)	***A2***	2	NMP	0

23	**PC4** (5)	Ni(Bipy)Cl_2 (20)	***A3***	2	NMP	51
24	**PC4** (5)	Ni(Bipy)Cl_2 (20)	***A4***	2	NMP	52
25	**PC4** (4)	Ni(Bipy)Cl_2 (20)	***A1***	2	NMP	67
26	**PC4** (3)	Ni(Bipy)Cl_2 (20)	***A1***	2	NMP	71
27	**PC4** (2)	Ni(Bipy)Cl_2 (20)	***A1***	2	NMP	68
28	**PC4** (3)	Ni(Bipy)Cl_2 (15)	***A1***	2	NMP	62
29	**PC4** (3)	Ni(Bipy)Cl_2 (10)	***A1***	2	NMP	63
30	**PC4** (3)	Ni(Bipy)Cl_2 (25)	***A1***	2	NMP	63
31	**PC4** (3)	Ni(Bipy)Cl_2 (20)	***A1***	1.8	NMP	74
32	**PC4** (3)	Ni(Bipy)Cl_2 (20)	***A1***	1.5	NMP	69
33	**PC4** (3)	Ni(Bipy)Cl_2 (20)	***A1***	1.8	NMP	65[b]
34	**PC4** (3)	Ni(Bipy)Cl_2 (20)	***A1***	1.8	NMP	78[c]
35	**PC4** (3)	Ni(Bipy)Cl_2 (0)	***A1***	1.8	NMP	Trace[c]
36	**PC4** (3)	Ni(Bipy)Cl_2 (20)	***A1***	0	NMP	68[c]

[a]Reaction conditions: nitroarene (***N1***, 0.1 mmol, 1.0 equiv.), tertiary alkylamine (***A***, 6.0 equiv.), 3,3,3-trifluoropropene (***O1***, 1.0 atm.), photocatalyst (**PC1–PC4,** 3–5 mol %), Ni salt/ligand (0–25 mol %), Hantzsch ester (**HE,** 1.8–2.0 equiv.), solvent (1.0 mL), ~40 ºC, blue LEDs (30 W, 455–460 nm), 18 h.
[a] Isolated yield. [b] Reaction time of 12 h. [c] Reaction time of 24 h.

General procedure for optimization of the oxo-aminomethylation reaction of 3,3,3-trifluoropropene (Table S1): An oven-dried, transparent 20 mL Teflon screw-capped Schlenk tube equipped with a stir bar was sequentially charged with nitroarene (***N1***, 1.0 equiv., 0.1 mmol), photocatalyst (**PC1**–**PC5**), Ni catalyst, ligand (**L1**–**L6**) (or ligated Ni complex), and Hantzsch ester (diethyl 1,4-dihydro-2,6-dimethyl-3,5-pyridinedicarboxylate, **HE**). Dried *N*-methyl-2-pyrrolidone (NMP, 1.0 mL) was then transferred into the tube via syringe. Subsequently, tertiary alkylamine (***A1***–***A4***, 6.0 equiv., 0.6 mmol) was transferred into the tube via syringe. The resulting mixture was degassed via blowing with a balloon filled with 3,3,3-trifluoropropene (***O1***, ~1 L) for 2 min (**Top figure, Figure S1**), after which time the tube was quickly capped with a Teflon screw cap such that it was filled with 3,3,3-trifluoropropene in atmospheric pressure. The reaction mixture was vigorously stirred and irradiated using 30 W blue LEDs (λ = 455–460 nm) for 24 h, during which time the proximal temperature was controlled at approximately ~40 ºC

(with two fans for cooling, **bottom figure, Figure S1**). At this point, the reaction mixture was diluted with ethyl acetate (100 mL) and washed with water (50 mL × 2). The organic fraction was further dried with anhydrous Na_2SO_4 and concentrated *in*

vacuo with the aid of rotary evaporator. The residue was purified by preparative thin-layer chromatography using a mixture of petroleum ether and ethyl acetate as an eluent to afford the product **11**.

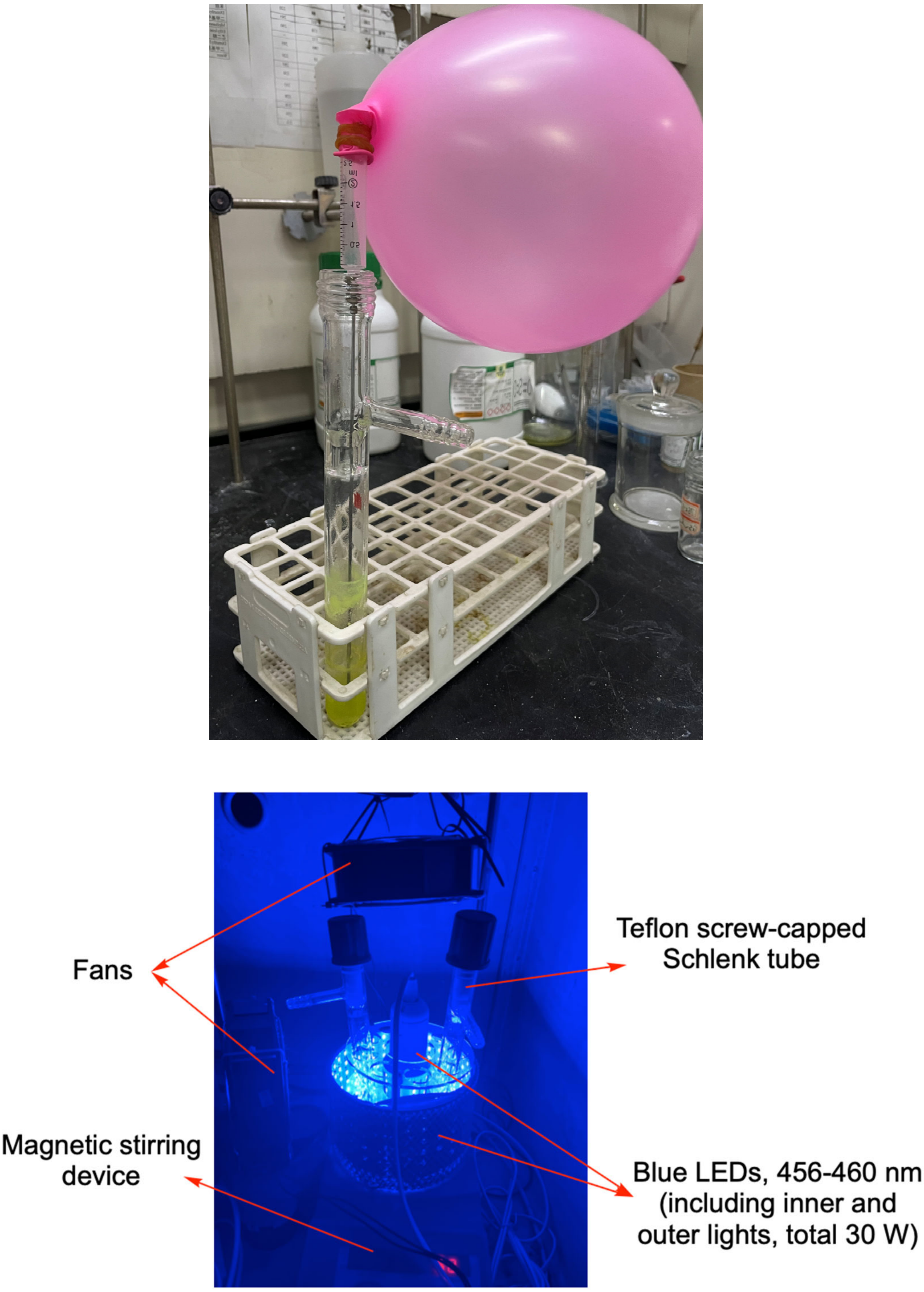

Fig. S1. Photograph of the reaction setup. The resulting mixture was degassed via blowing with a balloon filled with 3,3,3-trifluoropropene (***O1***, ~1 L), which is denser than air to promote the displacement of air (**Top**); The reaction mixture was vigorously stirred and irradiated using 30 W blue LEDs (**Bottom**).

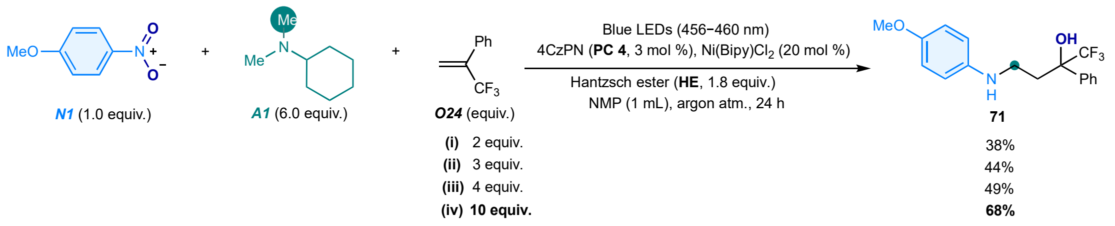

Figure S2. Optimization of reaction conditions using non-gaseous alkenes as reactant

General procedure for optimization of reaction conditions using non-gaseous alkenes as reactant (Figure S2): An oven-dried, transparent 20 mL Teflon screw-capped Schlenk tube equipped with a stir bar was sequentially charged with nitroarene (***N1***, 1.0 equiv., 0.1 mmol), 4CzPN (**PC4**, 3 mol%, 0.003 mmol), Ni(Bipy)Cl_2 (20 mol%, 0.02 mmol), and Hantzsch ester (**HE**, 1.8 equiv., 0.18 mmol). The tube was evacuated *in vacuo* and then backfilled with argon for three times. Dried *N*-methyl-2-pyrrolidone (NMP, 1.0 mL) was then transferred into the tube via syringe. Subsequently, *N*,*N*-dimethylcyclohexylamine (***A1***, 6.0 equiv., 0.6 mmol) and (3,3,3-trifluoroprop-1-en-2-yl)benzene (2.0–10.0 equiv.) were transferred into the tube via syringe. The resulting mixture was stirred under an argon atmosphere and irradiated using 30 W blue LEDs (λ = 455–460 nm) for 24 h, during which time the proximal temperature was controlled at approximately ~40 °C (with two fans for cooling). At this point, the reaction mixture was diluted with ethyl acetate (100 mL) and washed with water (50 mL × 2). The organic fraction was further dried with anhydrous Na_2SO_4 and concentrated *in vacuo* with the aid of rotary evaporator. The residue was purified by preparative thin-layer chromatography using a mixture of petroleum ether and ethyl acetate as an eluent to afford the product **71**.

Mechanistic Study

(a) Probing water as the hydroxyl source by adding ^{18}O-water

The hydroxyl group in the 3-arylamino alcohol products could originate from water, either as residual moisture in the reaction mixture or as a byproduct of nitroarene reductive deoxygenation. To determine whether water contributes to hydroxyl incorporation, we conducted the oxo-aminylmethylation reaction in the presence of excess ^{18}O-labeled water (**Figure S3**). However, high-resolution mass spectrometry (HRMS) analysis confirmed that only the unlabeled products **67** and **77** were obtained, with no ^{18}O incorporation. This result suggested that the oxygen atom from the nitro group of the nitroarenes is likely transferred directly to the alkenes, ultimately forming the hydroxyl group in 3-arylamino alcohols.

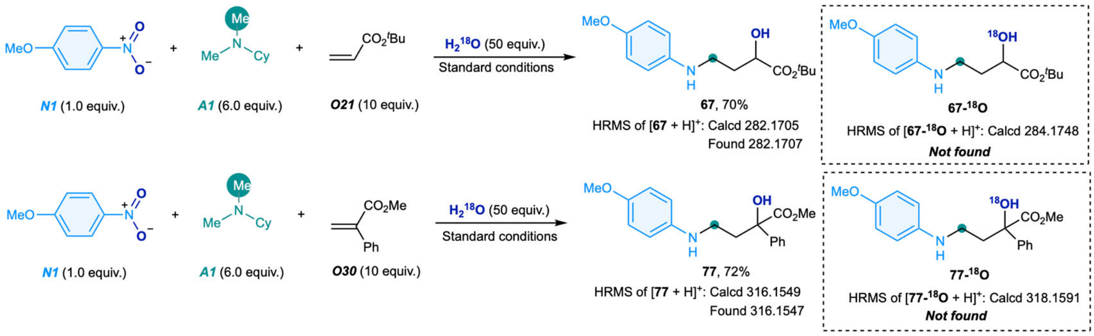

Fig. S3. Exogeneous water is not the hydroxyl source of product

Reaction Procedure: An oven-dried, transparent 20 mL Teflon screw-capped Schlenk tube equipped with a stir bar was sequentially charged with nitroarene (***N1***, 1.0 equiv., 0.1 mmol), 4CzPN (**PC4**, 3 mol%, 0.003 mmol), Ni(Bipy)Cl_2 (20 mol%, 0.02 mmol), and Hantzsch ester (**HE**, 1.8 equiv., 0.18 mmol). The tube was evacuated *in vacuo* and then backfilled with argon for three times. Dried *N*-methyl-2-pyrrolidone (NMP, 1.0 mL) was then transferred into the tube via syringe. Subsequently, $H_2{}^{18}O$ (50 equiv., 5.0 mmol), *N*,*N*-dimethylcyclohexylamine (***A1*** 6.0 equiv., 0.6 mmol) and alkene (***O21*** or ***O30***, 10 equiv., 1.0 mmol) were transferred into the tube via syringe. The resulting

mixture was stirred under an argon atmosphere and irradiated using 30 W blue LEDs (λ = 455–460 nm) for 24 h, during which time the proximal temperature was controlled at approximately ~40 ºC (with two fans for cooling). At this point, the reaction mixture was diluted with ethyl acetate (100 mL) and washed with water (50 mL × 2). The organic fraction was further dried with anhydrous Na_2SO_4 and concentrated *in vacuo* with the aid of rotary evaporator. The residue was purified by preparative thin-layer chromatography using a mixture of petroleum ether and ethyl acetate as an eluent to afford the **67** and **77** in 70% and 72% yields, respectively. HRMS analysis indicated that the products are **67** and **77** but not **67-^{18}O** and **77-^{18}O** (as shown below).

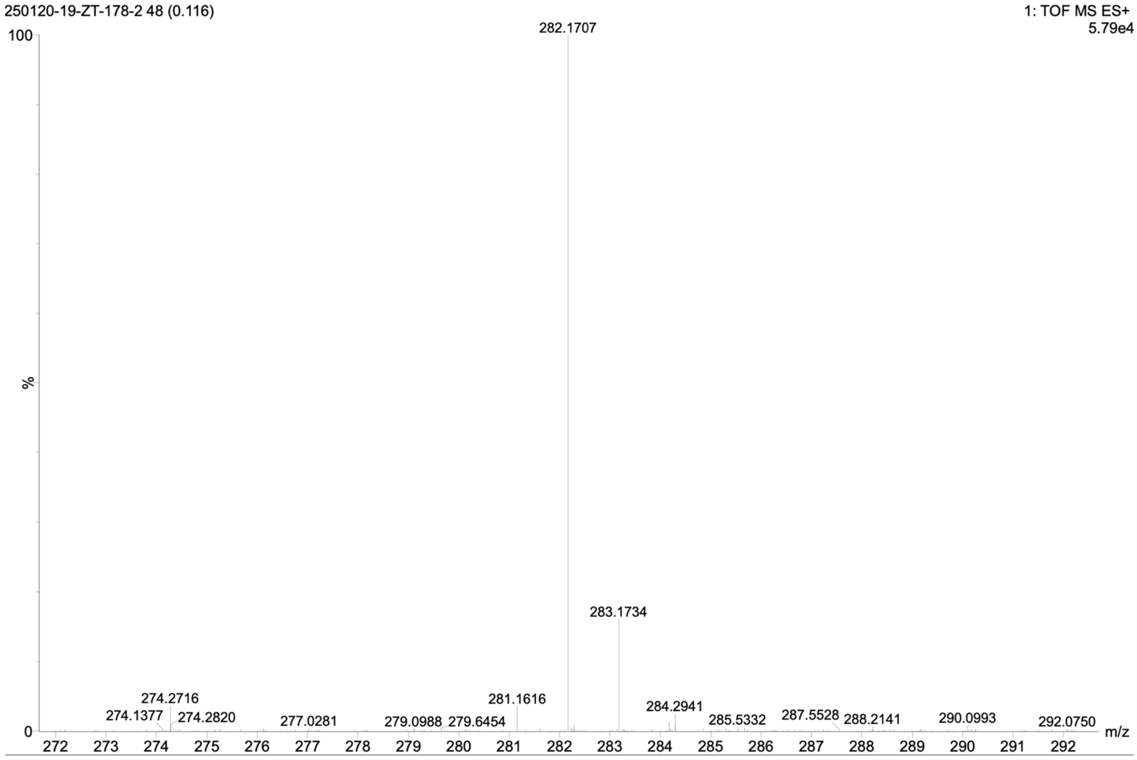

HRMS of [**67**+H]$^+$ calcd 282.1705; found 282.1707

Elemental Composition Report Page 1

Single Mass Analysis
Tolerance = 5.0 PPM / DBE: min = -1.5, max = 50.0
Element prediction: Off
Number of isotope peaks used for i-FIT = 3

Monoisotopic Mass, Even Electron Ions
101 formula(e) evaluated with 1 results within limits (up to 50 best isotopic matches for each mass)
Elements Used:
C: 16-18 H: 9-40 N: 1-8 O: 1-12
21
250120-19-ZT-178-7 110 (0.243) 1: TOF MS ES+ 3.00e+005

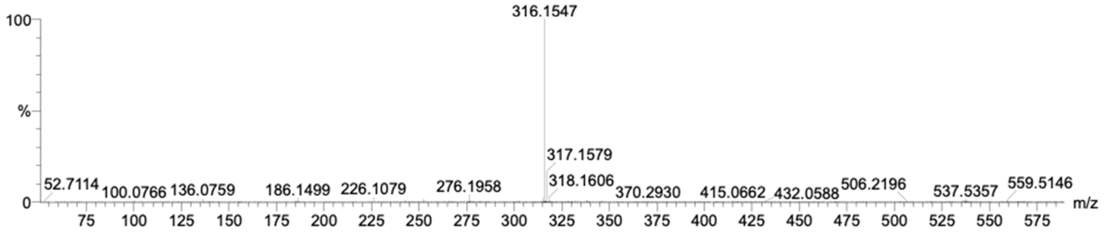

		mDa	PPM	DBE				
Minimum:				-1.5				
Maximum:		5.0	5.0	50.0				

Mass	Calc. Mass	mDa	PPM	DBE	i-FIT	Norm	Conf(%)	Formula
316.1547	316.1549	-0.2	-0.6	8.5	459.8	n/a	n/a	C18 H22 N O4

HRMS of [**77**+H]$^+$ calcd 316.1549; found 316.1547

(b) Probing γ-arylamino cabocation as possible intermediate by adding methanol as nucleophile

We hypothesized that the 3-arylaminopropyl cation species (***Int-1***) could be an intermediate, which intercepts the oxygen atom of nitroarene ***N1*** to afford the amino alcohol product **67'**. In the presence of excess methanol as a competitive nucleophile, the model reaction exclusively yielded the amino alcohol product **67**, with no formation of the 1-methoxy-substituted alkyl aniline **67'** (**Figure S4**). This result suggested that the species ***Int-1*** is unlikely to be an intermediate in product formation.

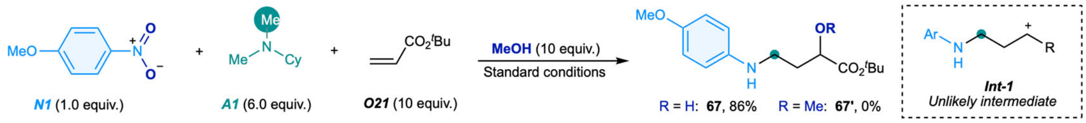

Fig. S4. γ-Arylamino cabocation species is unlikely the reaction intermediate

Reaction Procedure: An oven-dried, transparent 20 mL Teflon screw-capped Schlenk tube equipped with a stir bar was sequentially charged with nitroarene (***N1***, 1.0 equiv., 0.1 mmol), 4CzPN (**PC4**, 3 mol%, 0.003 mmol), Ni(Bipy)Cl_2 (20 mol%, 0.02 mmol), and Hantzsch ester (**HE**, 1.8 equiv., 0.18 mmol). The tube was evacuated *in vacuo* and then backfilled with argon for three times. Dried *N*-methyl-2-pyrrolidone (NMP, 1.0 mL) was then transferred into the tube via syringe. Subsequently, MeOH (10 equiv., 1.0 mmol), *N*,*N*-dimethylcyclohexylamine (***A1*** 6.0 equiv., 0.6 mmol) and *tert*-butyl acrylate (10 equiv., 1.0 mmol) were transferred into the tube via syringe. The resulting mixture was stirred under an argon atmosphere and irradiated using 30 W blue LEDs (λ = 455–460 nm) for 24 h, during which time the proximal temperature was controlled at approximately ~40 °C (with two fans for cooling). At this point, the reaction mixture was diluted with ethyl acetate (100 mL) and washed with water (50 mL × 2). The organic fraction was further dried with anhydrous Na_2SO_4 and concentrated *in vacuo* with the aid of rotary evaporator. The residue was purified by preparative thin-layer

chromatography using a mixture of petroleum ether and ethyl acetate as an eluent to afford the **67** in 86% yield. No **67’** was isolated.

(c) Probing *N*-(3-oxo alkyl) aniline as possible intermediate

We considered the possibility that the 3-oxo-propyl aniline species (***Int-2***) might serve as an intermediate, undergoing photocatalytic reduction to form the amino alcohol **112**. However, control experiments with 3-oxo-3-phenylpropyl aniline **111** under otherwise identical conditions did not produce the amino alcohol **112**, indicating that the species ***Int-2*** is unlikely to be an intermediate in the reaction (**Figure S5**).

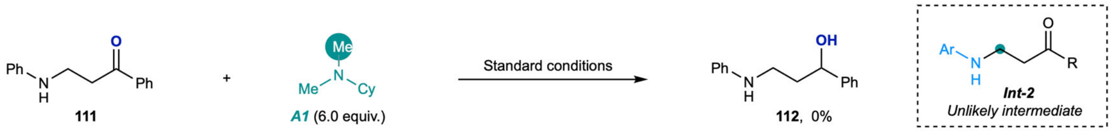

Fig. S5. *N*-(3-oxo alkyl) aniline is unlikely to be an intermediate in the reaction

Reaction Procedure: An oven-dried, transparent 20 mL Teflon screw-capped Schlenk tube equipped with a stir bar was sequentially charged with 3-oxo-3-phenylpropyl aniline (**111,** 1.0 equiv., 0.1 mmol, 22.5 mg), 4CzPN (**PC4**, 3 mol%, 0.003 mmol), Ni(Bipy)Cl_2 (20 mol%, 0.02 mmol), and Hantzsch ester (**HE**, 1.8 equiv., 0.18 mmol). The tube was evacuated *in vacuo* and then backfilled with argon for three times. Dried *N*-methyl-2-pyrrolidone (NMP, 1.0 mL) was then transferred into the tube via syringe. Subsequently, *N*,*N*-dimethylcyclohexylamine (***A1*** 6.0 equiv., 0.6 mmol) was transferred into the tube via syringe. The resulting mixture was stirred under an argon atmosphere and irradiated using 30 W blue LEDs (λ = 455–460 nm) for 24 h, during which time the proximal temperature was controlled at approximately ~40 ºC (with two fans for cooling). After the reaction, the mixture was monitored by TLC. No desired amino alcohol product **112** was detected by TLC analysis, and a majority of **111** remains unreacted.

(d) Probing the nitrogen-based intermediates for reaction

Nitroarene can undergo reduction to sequentially give nitrosoarene, *N*-aryl hydroxylamine, azoxyarene, azoarene, 1,2-diarylhydrazine, and aniline, which are all viable reaction intermediate that contribute to the *N*-arylamino alcohol products. When nitrobenzene (***N42***) was subjected to the reaction under standard conditions, *N*-trifluoroalkyl aniline **113** was formed in 94% yield. During the reaction, nitrobenzene (***N42***) is sequentially reduced to nitrosobenzene (***N42-i***), *N*-aryl hydroxylamine (***N42-ii***), azobenzene (***N42-iii***), azoxybenzene (***N42-iv***), *N*,*N*'-diphenyl hydrazine (***N42-v***), and aniline (***N42-vi***). Nitrobenzene may also react with a tertiary alkylamine (***A1***) under photoredox conditions to form *N*-phenyl imine (***N42-vii***) and *N*-methyl aniline (***N42-viii***), which could contribute to the reaction pathway. An exogenous 4-nitroanisole (***N1***) additive was introduced to act as the oxygen atom source for the hydroxyl group in the target amino alcohol product **113**, while also mimicking the redox conditions of the reaction. Nitrogen-based intermediates were then subjected to react under otherwise identical reaction conditions (**Figure S6**). Only nitrosobenzene (***N42-i***) and *N*-phenyl hydroxylamine (***N42-ii***) reacted to deliver **113** in 40% and 29% yields, respectively. The results suggested that both nitrosoarenes and *N*-aryl hydroxylamines are likely the key intermediates contributing to the formation of the amino alcohol products.

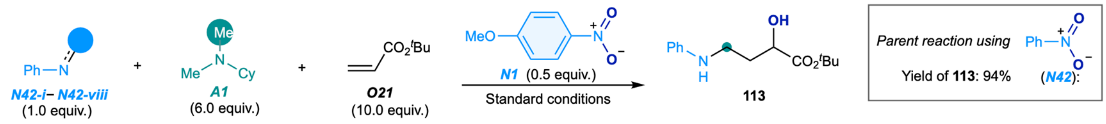

Possible nitrogen intermediates	Ph—N=O *N42-i*	Ph—NH—OH *N42-ii*	1/2 Ph—N=N—Ph *N42-iii*	1/2 Ph—N(O⁻)=N—Ph *N42-iv*	1/2 Ph—NH—HN—Ph *N42-v*	Ph—NH_2 *N42-vi*	Ph—N=CH_2 *N42-vii*	Ph—NH—Me *N42-viii*
Yield of **113**	40%	29%	0%	0%	0%	0%	0%	0%

Fig. S6. Nitrosoarene and *N*-aryl hydroxylamine are likely the reaction intermediates.

Reaction Procedure: An oven-dried, transparent 20 mL Teflon screw-capped Schlenk tube equipped with a stir bar was sequentially charged with nitroarene (***N42-i–N55-viii***,

1.0 equiv., 0.1 mmol), 4CzPN (**PC4**, 3 mol%, 0.003 mmol), Ni(Bipy)Cl_2 (20 mol%, 0.02 mmol), and Hantzsch ester (**HE**, 1.8 equiv., 0.18 mmol). The tube was evacuated *in vacuo* and then backfilled with argon for three times. Dried *N*-methyl-2-pyrrolidone (NMP, 1.0 mL) was then transferred into the tube via syringe. Subsequently, *N*,*N*-dimethylcyclohexylamine (***A1*** 6.0 equiv., 0.6 mmol) and *tert*-butyl acrylate (***O21***, 10 equiv., 1.0 mmol) were transferred into the tube via syringe. The resulting mixture was stirred under an argon atmosphere and irradiated using 30 W blue LEDs (λ = 455–460 nm) for 24 h, during which time the proximal temperature was controlled at approximately ~40 °C (with two fans for cooling). At this point, the reaction mixture was diluted with ethyl acetate (100 mL) and washed with water (50 mL × 2). The organic fraction was further dried with anhydrous Na_2SO_4 and concentrated *in vacuo* with the aid of rotary evaporator. The residue was purified by preparative thin-layer chromatography using a mixture of petroleum ether and ethyl acetate as an eluent to afford the product **113**.

(e) Probing the reaction intermediates at the initial stage of reaction

During the initial phase of the model reaction (4 h), the reaction mixture was subject to HRMS analysis to identify emerging reaction intermediates and co-products. High-resolution mass spectrometry (HRMS) analysis identified several co-products (***N1-i***, ***S1*–*S5***) (**Figure S7**).

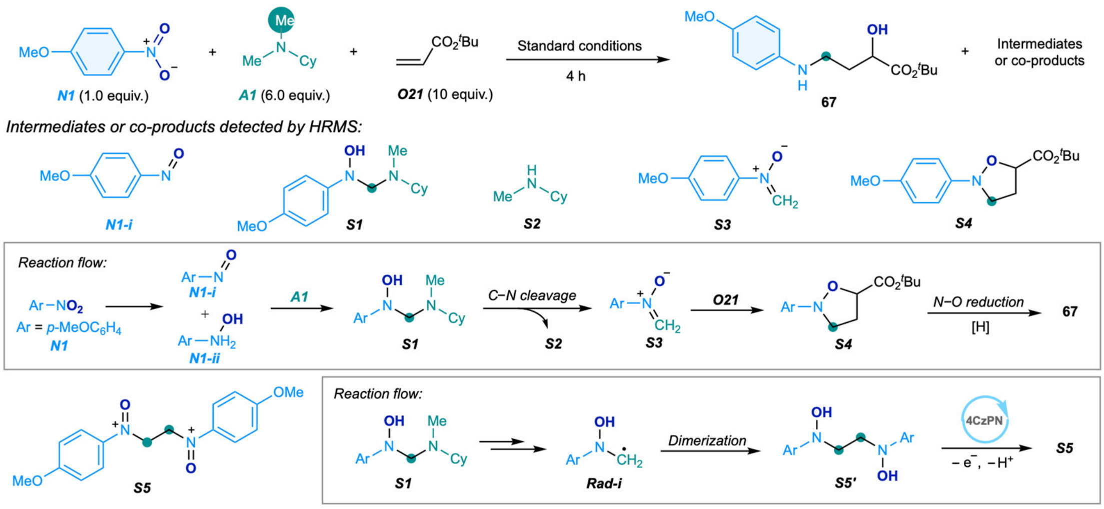

Fig. S7. Analysis of reaction co-products in the early reaction stage.

Reaction Procedure: An oven-dried, transparent 20 mL Teflon screw-capped Schlenk tube equipped with a stir bar was sequentially charged with nitroarene (***N1***, 1.0 equiv., 0.1 mmol), 4CzPN (**PC4**, 3 mol%, 0.003 mmol), Ni(Bipy)Cl_2 (20 mol%, 0.02 mmol), and Hantzsch ester (**HE**, 1.8 equiv., 0.18 mmol). The tube was evacuated *in vacuo* and then backfilled with argon for three times. Dried *N*-methyl-2-pyrrolidone (NMP, 1.0 mL) was then transferred into the tube via syringe. Subsequently, *N*,*N*-dimethylcyclohexylamine (***A1***, 6.0 equiv., 0.6 mmol) and *tert*-butyl acrylate (***O21***, 10 equiv., 1.0 mmol) were transferred into the tube via syringe. The resulting mixture was stirred under an argon atmosphere and irradiated using 30 W blue LEDs (λ = 455–460 nm) for 4 h, during which time the proximal temperature was controlled at approximately ~40 °C (with two fans for cooling). After the reaction, the mixture was detected by HRMS, and ***N1-i***, ***S1-S5*** were successfully detected by HRMS (as shown below).

Elemental Composition Report **Page 1**

Single Mass Analysis
Tolerance = 5.0 PPM / DBE: min = -1.5, max = 50.0
Element prediction: Off
Number of isotope peaks used for i-FIT = 3

Monoisotopic Mass, Even Electron Ions
33 formula(e) evaluated with 1 results within limits (up to 50 best isotopic matches for each mass)
Elements Used:
C: 7-14 H: 7-49 N: 1-8 O: 1-12
21
250120-19-ZT2-4H 28 (0.078) 1: TOF MS ES+ 1.68e+002

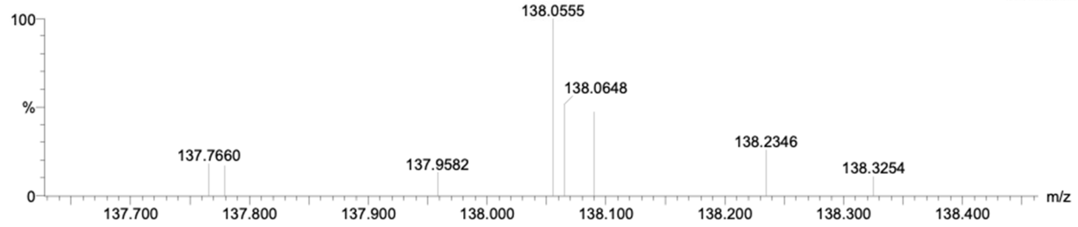

Minimum:				-1.5				
Maximum:		5.0	5.0	50.0				
Mass	Calc. Mass	mDa	PPM	DBE	i-FIT	Norm	Conf(%)	Formula
138.0555	138.0555	0.0	0.0	4.5	47.7	n/a	n/a	C7 H8 N O2

HRMS of [***N1-i***+H]$^+$ calcd 138.0555; found 138.0555

Elemental Composition Report **Page 1**

Single Mass Analysis
Tolerance = 5.0 PPM / DBE: min = -1.5, max = 50.0
Element prediction: Off
Number of isotope peaks used for i-FIT = 3

Monoisotopic Mass, Even Electron Ions
87 formula(e) evaluated with 1 results within limits (up to 50 best isotopic matches for each mass)
Elements Used:
C: 15-15 H: 7-49 N: 1-8 O: 1-12
21
250120-19-ZT2-4H 26 (0.075) 1: TOF MS ES+ 1.24e+002

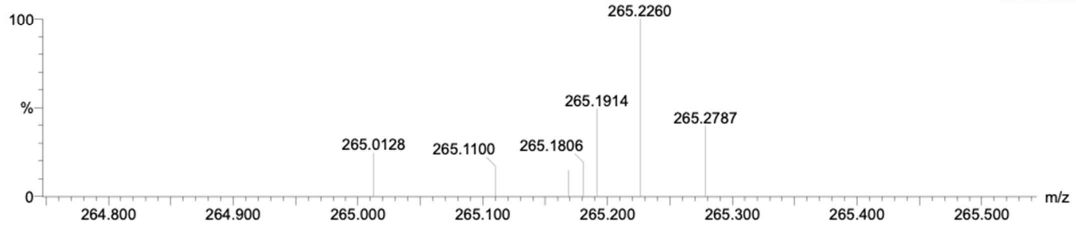

Minimum:				-1.5				
Maximum:		5.0	5.0	50.0				
Mass	Calc. Mass	mDa	PPM	DBE	i-FIT	Norm	Conf(%)	Formula
265.1914	265.1916	-0.2	-0.8	4.5	33.3	n/a	n/a	C15 H25 N2 O2

HRMS of [***S1***+H]$^+$ calcd 265.1916; found 265.1914

Elemental Composition Report

Single Mass Analysis

Tolerance = 5.0 PPM / DBE: min = -1.5, max = 50.0
Element prediction: Off
Number of isotope peaks used for i-FIT = 3

Monoisotopic Mass, Even Electron Ions
7 formula(e) evaluated with 1 results within limits (up to 50 best isotopic matches for each mass)
Elements Used:
C: 7-15 H: 7-49 N: 1-8
21
250120-19-ZT2-4H 21 (0.065)

1: TOF MS ES+
1.01e+003

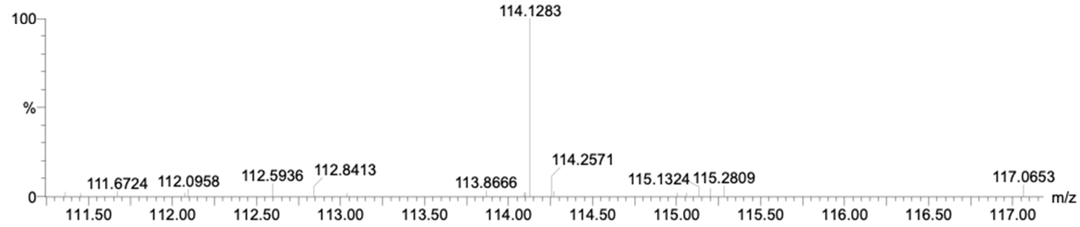

Mass	Calc. Mass	mDa	PPM	DBE	i-FIT	Norm	Conf(%)	Formula
Minimum:				-1.5				
Maximum:		5.0	5.0	50.0				
114.1283	114.1283	0.0	0.0	0.5	54.8	n/a	n/a	C7 H16 N

HRMS of [***S2***+H]$^+$ calcd 114.1283; found 114.1283

Elemental Composition Report

Single Mass Analysis

Tolerance = 5.0 PPM / DBE: min = -1.5, max = 50.0
Element prediction: Off
Number of isotope peaks used for i-FIT = 3

Monoisotopic Mass, Even Electron Ions
41 formula(e) evaluated with 1 results within limits (up to 50 best isotopic matches for each mass)
Elements Used:
C: 7-16 H: 7-49 N: 1-8 O: 1-12
21
250120-19-ZT2-4H 32 (0.086)

1: TOF MS ES+
6.92e+002

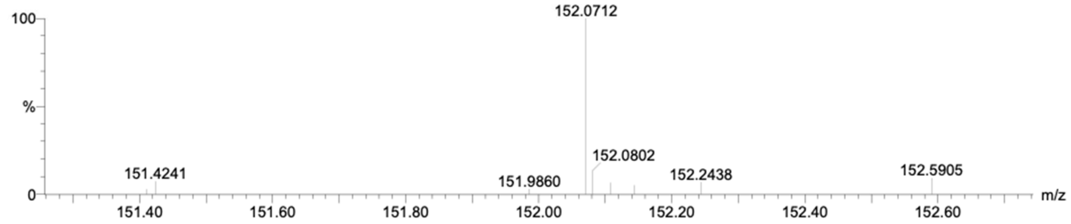

Mass	Calc. Mass	mDa	PPM	DBE	i-FIT	Norm	Conf(%)	Formula
Minimum:				-1.5				
Maximum:		5.0	5.0	50.0				
152.0712	152.0712	0.0	0.0	4.5	60.8	n/a	n/a	C8 H10 N O2

HRMS of [***S3***+H]$^+$ calcd 152.0712; found 152.0712

Elemental Composition Report **Page 1**

Single Mass Analysis
Tolerance = 5.0 PPM / DBE: min = -1.5, max = 50.0
Element prediction: Off
Number of isotope peaks used for i-FIT = 3

Monoisotopic Mass, Even Electron Ions
155 formula(e) evaluated with 1 results within limits (up to 50 best isotopic matches for each mass)
Elements Used:
C: 7-16 H: 7-49 N: 1-8 O: 1-12 Na: 1-1

21
250120-19-ZT2-4H 70 (0.158)

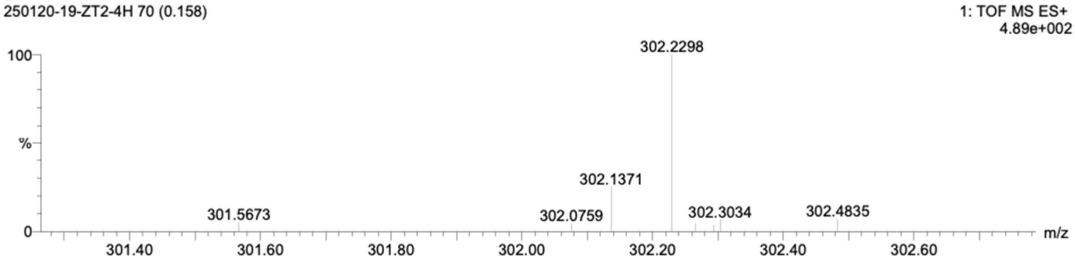

Minimum:				-1.5				
Maximum:		5.0	5.0	50.0				
Mass	Calc. Mass	mDa	PPM	DBE	i-FIT	Norm	Conf(%)	Formula
302.1371	302.1368	0.3	1.0	5.5	56.4	n/a	n/a	C15 H21 N O4 Na

HRMS of [***S4***+Na]$^{+}$ calcd 302.1368; found 302.1371

Elemental Composition Report **Page 1**

Single Mass Analysis
Tolerance = 5.0 PPM / DBE: min = -1.5, max = 50.0
Element prediction: Off
Number of isotope peaks used for i-FIT = 3

Monoisotopic Mass, Even Electron Ions
174 formula(e) evaluated with 1 results within limits (up to 50 best isotopic matches for each mass)
Elements Used:
C: 7-16 H: 7-49 N: 1-8 O: 1-12
21
250120-19-ZT2-4H 212 (0.445)

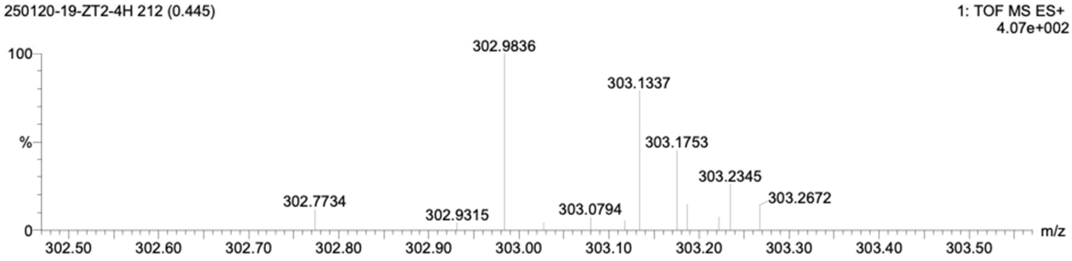

Minimum:				-1.5				
Maximum:		5.0	5.0	50.0				
Mass	Calc. Mass	mDa	PPM	DBE	i-FIT	Norm	Conf(%)	Formula
303.1337	303.1345	-0.8	-2.6	8.5	64.1	n/a	n/a	C16 H19 N2 O4

HRMS of [***S5***+H]$^{+}$ calcd 303.1345; found 303.1337

(f) Radical trap experiment

When 2,2,6,6-tetramethylpiperidine-1-oxyl (TEMPO), acting as a radical scavenger, was introduced to the model reaction, only a trace amount of *N*-arylamino alcohol product **67** was produced by TCL analysis. The results suggested that radical pathway is operative (**Figure S8**). However, other TEMPO-trapped species based on ***N1*** and ***A1*** could not be detected.

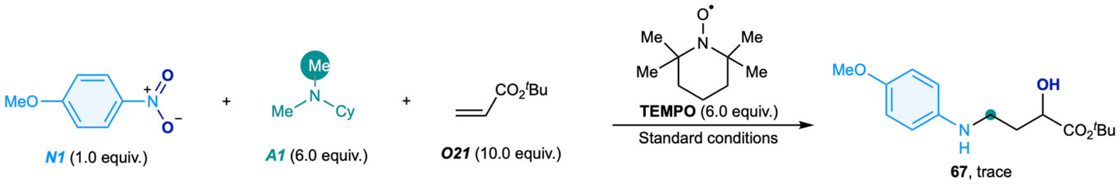

Fig. S8. Radical trap experiment study.

Reaction Procedure: An oven-dried, transparent 20 mL Teflon screw-capped Schlenk tube equipped with a stir bar was sequentially charged with nitroarene (***N1***, 1.0 equiv., 0.1 mmol), 4CzPN (**PC4**, 3 mol%, 0.003 mmol), Ni(Bipy)Cl_2 (20 mol%, 0.02 mmol), Hantzsch ester (**HE**, 1.8 equiv., 0.18 mmol) and 2,2,6,6-tetramethyl-1-piperidinyloxy (**TEMPO**, 6.0 equiv., 0.6 mmol). The tube was evacuated *in vacuo* and then backfilled with argon for three times. Dried *N*-methyl-2-pyrrolidone (NMP, 1.0 mL) was then transferred into the tube via syringe. Subsequently, *N*,*N*-dimethylcyclohexylamine (***A1*** 6.0 equiv., 0.6 mmol) and *tert*-butyl acrylate (***O21***, 10 equiv., 1.0 mmol) were transferred into the tube via syringe. The resulting mixture was stirred under an argon atmosphere and irradiated using 30 W blue LEDs (λ = 455–460 nm) for 24 h, during which time the proximal temperature was controlled at approximately ~40 °C (with two fans for cooling). After the reaction, the mixture was monitored by TLC. A trace of *N*-arylamino alcohol **67** was detected by TLC analysis (Using authenthic compound for comparison).

(g) Probing the *N*–O cleavage step of isoxazolidine

The reductive *N*–O cleavage of isoxazolidines plays a key role in the formation of 3-arylamino alcohol products. To investigate this transformation, the reductive ring-opening of isoxazolidine **114** was studied (**Figure S9**).

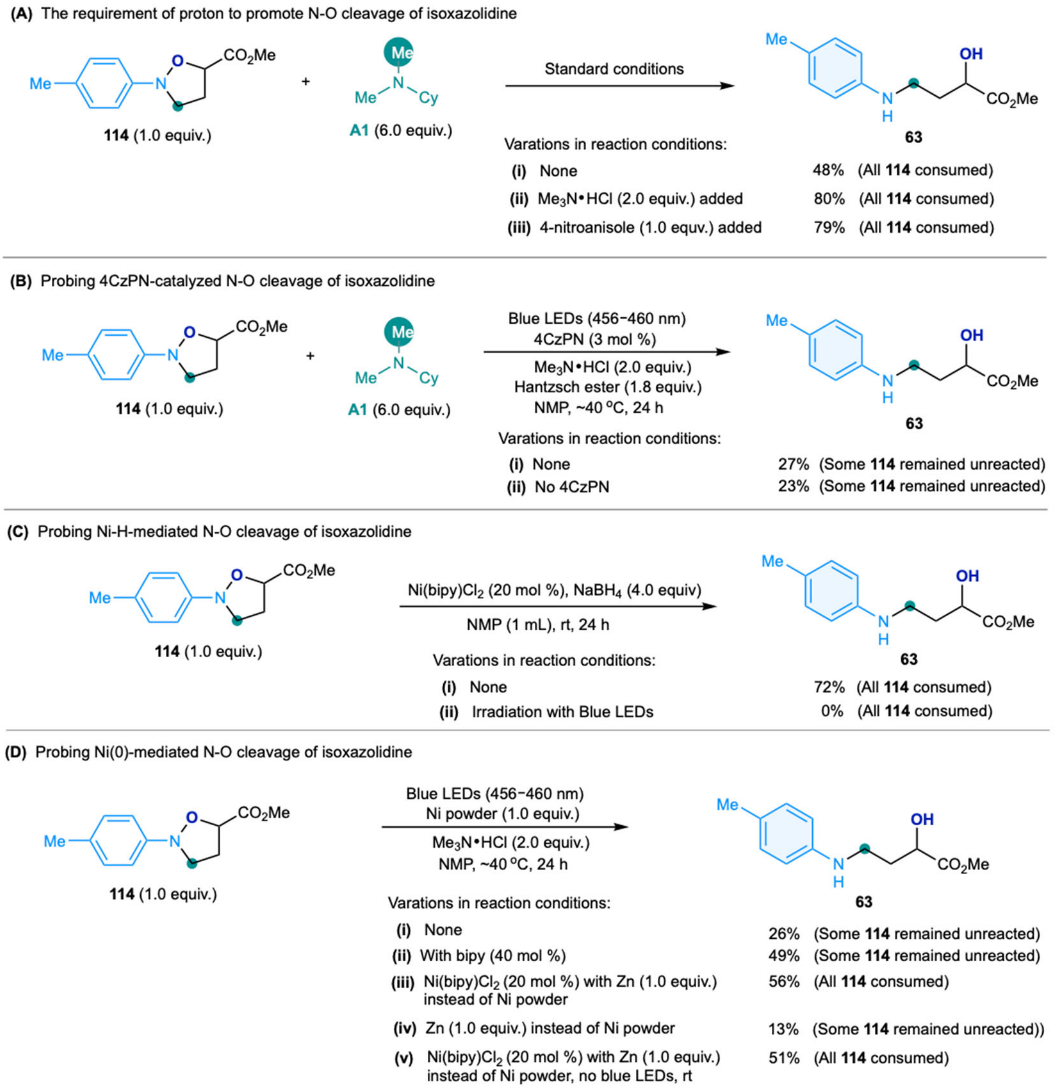

Fig. S9. Study of *N*-O cleavage of isoxazolidine.

Under standard conditions, isoxazolidine **114** underwent partial reduction, yielding 3-arylamino alcohol **63** in 48% yield, supporting its role as an intermediate (**Figure S9A(i)**). When a protic source, trimethylamine hydrochloride, was introduced, the reaction efficiency improved significantly, delivering **63** in 80% yield (**Figure S9A(ii)**). Alternatively, the addition of 4-nitroanisole also promoted the reaction to afford **63** in

79% yield, suggesting that the photocatalytic reduction of nitroarene drives the oxidation of tertiary alkylamine ***A1*** and Hantzsch ester (**HE**) that release protons to promote the ring-opening reaction of **114** (**Figure S9A(iii)**). These results highlighted the necessity of proton, generated during the photocatalytic oxidation of tertiary alkylamine ***A1*** and Hantzsch ester (**HE**), in facilitating *N*–O bond cleavage.

Under acidified conditions, the target compound **63** was obtained in yields of 27% and 23% with or without the addition of 4CzPN, respectively, demonstrating that the 4CzPN-mediated photocatalytic pathway does not influence the ring-opening process of compound **114** (**Figure S9B(i) and (ii)**).

Next, we conducted the reaction in the presence of *in-situ* generated Ni–H species using Ni(Bipy)Cl_2 and sodium borohydride. Without blue light irradiation, isoxazolidine **114** converted efficiently to **63** in 72% yield (**Figure S9C(i)**). However, under blue LEDs, **114** decomposed without forming the amino alcohol product (**Figure S9C(ii)**), implying that Ni–H species is not the primary reductant responsible for the N–O cleavage.

Finally, we explored the role of Ni(0) species, Ni^0(Bipy), as a potential ring-opening mediator. Under acidified conditions, when stoichiometric nickel powder was employed, isoxazolidine **114** underwent partial reduction, yielding 3-arylamino alcohol **63** in 26% yield (**Figure S9D(i)**). In the presence of 40 mol % of Bipy ligand, the yield was promoted to 49%, suggesting that Ni^0(Bipy) is the viable reductant (**Figure S9D(ii)**). The use of catalytic amount of Ni^0(Bipy)Cl_2 in association with stoichiometric Zn powder also led to the complete conversion, affording **63** in 56% yield (**Figure S9D(iii)**). Control experiment indicated that the *in-situ* formed Ni^0(Bipy) but not Zn powder mediates the ring-opening reaction, as the combination of Ni(II) and Zn powder can lead to much higher product yield than Zn itself (**Figure S9D(iv)**). Without blue LEDs, such reductive Ni-catalyzed ring-opening reaction still proceeded smoothly, suggesting that such event is unlikely driven by light energy but only the redox chemistry driven by Ni(0) species (**Figure S9D(v)**). These control experiments confirmed that Ni^0(Bipy) is likely the key reductant, enabling the conversion of isoxazolidines to amino alcohols.

Reactions in Fig S9(A)

Reaction Procedure: An oven-dried, transparent 20 mL Teflon screw-capped Schlenk tube equipped with a stir bar was sequentially charged with methyl 2-(*p*-tolyl)isoxazolidine-5-carboxylate (**114**, 1.0 equiv., 0.1 mmol), 4CzPN (**PC4**, 3 mol%, 0.003 mmol), Ni(Bipy)Cl_2 (20 mol%, 0.02 mmol), Hantzsch ester (**HE**, 1.8 equiv., 0.18 mmol), and trimethylamine hydrochloride (2.0 equiv., 0.2 mmol, added under **Reaction conditions ii**) or 4-nitroanisole (1.0 equiv., 0.1 mmol, added under **Reaction conditions iii**). The tube was evacuated *in vacuo* and then backfilled with argon for three times. Dried *N*-methyl-2-pyrrolidone (NMP, 1.0 mL) was then transferred into the tube via syringe. Subsequently, *N*,*N*-dimethylcyclohexylamine (***A1*** 6.0 equiv., 0.6 mmol) was transferred into the tube via syringe. The resulting mixture was stirred under an argon atmosphere and irradiated using 30 W blue LEDs (λ = 455–460 nm) for 24 h, during which time the proximal temperature was controlled at approximately ~40 ºC (with two fans for cooling). At this point, the reaction mixture was diluted with ethyl acetate (100 mL) and washed with water (50 mL × 2). The organic fraction was further dried with anhydrous Na_2SO_4 and concentrated *in vacuo* with the aid of rotary evaporator. The residue was purified by preparative thin-layer chromatography using a mixture of petroleum ether and ethyl acetate as an eluent to afford the product **63**.

Reactions in Fig S9(B)

Reaction Procedure: An oven-dried, transparent 20 mL Teflon screw-capped Schlenk tube equipped with a stir bar was sequentially charged with methyl 2-(*p*-tolyl)isoxazolidine-5-carboxylate (**114**, 1.0 equiv., 0.1 mmol), 4CzPN (**PC4**, 3 mol% or 0 mol%), Hantzsch ester (**HE**, 1.8 equiv., 0.18 mmol), trimethylamine hydrochloride (2.0 equiv., 0.2 mmol). The tube was evacuated *in vacuo* and then backfilled with argon for three times. Dried *N*-methyl-2-pyrrolidone (NMP, 1.0 mL) was then transferred into the tube via syringe. Subsequently, *N*,*N*-dimethylcyclohexylamine (***A1*** 6.0 equiv., 0.6 mmol) was transferred into the tube via syringe. The resulting mixture was stirred under an argon atmosphere and irradiated using 30 W blue LEDs (λ = 455–460 nm) for 24 h, during which time the proximal temperature was controlled at approximately ~40 ºC (with two fans for cooling). At this point, the reaction mixture was diluted with ethyl acetate (100 mL) and washed with water (50 mL × 2). The organic fraction was further dried with anhydrous Na_2SO_4 and concentrated *in vacuo* with the aid of rotary

evaporator. The residue was purified by preparative thin-layer chromatography using a mixture of petroleum ether and ethyl acetate as an eluent to afford the product **63**.

Reactions in Fig S9(C)

Reaction Procedure: An oven-dried, transparent 20 mL Teflon screw-capped Schlenk tube equipped with a stir bar was sequentially charged with methyl 2-(*p*-tolyl)isoxazolidine-5-carboxylate (**114**, 1.0 equiv., 0.1 mmol), Ni(Bipy)Cl_2 (20 mol%, 0.02 mmol), $NaBH_4$ (4.0 equiv., 0.4 mmol). The tube was evacuated *in vacuo* and then backfilled with argon for three times. Dried *N*-methyl-2-pyrrolidone (NMP, 1.0 mL) was then transferred into the tube via syringe. **Reaction Conditions i**: The resulting mixture was stirred at r.t. for 24 h; **Reaction Conditions ii**: The resulting mixture was stirred under an argon atmosphere and irradiated using 30 W blue LEDs (λ = 455–460 nm) for 24 h, during which time the proximal temperature was controlled at approximately ~40 ºC (with two fans for cooling). At this point, the reaction mixture was diluted with ethyl acetate (100 mL) and washed with water (50 mL × 2). The organic fraction was further dried with anhydrous Na_2SO_4 and concentrated *in vacuo* with the aid of rotary evaporator. The residue was purified by preparative thin-layer chromatography using a mixture of petroleum ether and ethyl acetate as an eluent to afford the product **63**.

Reactions in Fig S9(D)

Reaction Procedure: Reaction Conditions i: An oven-dried, transparent 20 mL Teflon screw-capped Schlenk tube equipped with a stir bar was sequentially charged with methyl 2-(*p*-tolyl)isoxazolidine-5-carboxylate (**114**, 1.0 equiv., 0.1 mmol), Ni powder (1.0 equiv., 0.1 mmol), trimethylamine hydrochloride (2.0 equiv., 0.2 mmol). The tube was evacuated *in vacuo* and then backfilled with argon for three times. Dried *N*-methyl-2-pyrrolidone (NMP, 1.0 mL) was then transferred into the tube via syringe. The resulting mixture was stirred under an argon atmosphere and irradiated using 30 W blue LEDs (λ = 455–460 nm) for 24 h, during which time the proximal temperature was

controlled at approximately ~40 ºC (with two fans for cooling). At this point, the reaction mixture was diluted with ethyl acetate (100 mL) and washed with water (50 mL × 2). The organic fraction was further dried with anhydrous Na_2SO_4 and concentrated *in vacuo* with the aid of rotary evaporator. The residue was purified by preparative thin-layer chromatography using a mixture of petroleum ether and ethyl acetate as an eluent to afford the product **63**. For **Reaction Conditions ii-iv**, identical procedures were performed, except that the corresponding reactants and catalysts were added accordingly in the presence or absence of blue LEDs.

Substrate scope study

General procedure for the synthesis 3-arylamino alcohols using gaseous alkenes (General Procedure A):

An oven-dried, transparent 20 mL Teflon screw-capped Schlenk tube equipped with a stir bar was sequentially charged with nitroarene (1.0 equiv., 0.1 mmol), 4CzPN (3 mol%, 0.003 mmol), Ni(Bipy)Cl_2 (20 mol%, 0.02 mmol), and Hantzsch ester (**HE,** 1.8 equiv., 0.18 mmol). Dried *N*-methyl-2-pyrrolidone (NMP, 1.0 mL) was then transferred into the tube via syringe. Subsequently, *N*,*N*-dimethylcyclohexylamine (6.0 equiv., 0.6 mmol) was transferred into the tube via syringe. The resulting mixture was degassed via blowing with a balloon filled with gaseous alkene (~1 L) for 2 min, after which time the tube was quickly capped with a Teflon screw cap such that it was filled with gaseous alkenes in atmospheric pressure. The reaction mixture was vigorously stirred and irradiated using 30 W blue LEDs (λ = 455–460 nm) for 24 h, during which time the proximal temperature was controlled at approximately ~40 °C via cooling with fans. At this point, the reaction mixture was diluted with ethyl acetate (100 mL) and washed with water (50 mL × 2). The organic fraction was further dried with anhydrous Na_2SO_4 and concentrated *in vacuo* with the aid of rotary evaporator. The residue was purified by preparative thin-layer chromatography using a mixture of petroleum ether and ethyl acetate as an eluent to afford the target product (Refer to Fig. S1 for the experimental setup).

General procedure for the synthesis 3-arylamino alcohols using non-gaseous alkenes (General Procedure B):

An oven-dried, transparent 20 mL Teflon screw-capped Schlenk tube equipped with a stir bar was sequentially charged with nitroarene (1.0 equiv., 0.1 mmol), 4CzPN (3 mol%, 0.003 mmol), Ni(Bipy)Cl_2 (20 mol%, 0.02 mmol), Hantzsch ester (**HE,** 1.8 equiv., 0.18 mmol), and non-gaseous alkene (10.0 equiv., 1.0 mmol). The tube was

evacuated *in vacuo* and then backfilled with argon for three times. Dried *N*-methyl-2-pyrrolidone (NMP, 1.0 mL) was then transferred into the tube via syringe. Subsequently, *N*,*N*-dimethylcyclohexylamine (***A1***, 6.0 equiv., 0.6 mmol) was transferred into the tube via syringe. The resulting mixture was stirred under an argon atmosphere and irradiated using 30 W blue LEDs (λ = 455–460 nm) for 24 h, during which time the proximal temperature was controlled at approximately ~40 °C (with two fans for cooling). At this point, the reaction mixture was diluted with ethyl acetate (100 mL) and washed with water (50 mL × 2). The organic fraction was further dried with anhydrous Na_2SO_4 and concentrated *in vacuo* with the aid of rotary evaporator. The residue was purified by preparative thin-layer chromatography using a mixture of petroleum ether and ethyl acetate as an eluent to afford the target product (Refer to Fig. S1 for the experimental setup).

1,1,1-trifluoro-4-((4-methoxyphenyl)amino)butan-2-ol (11)

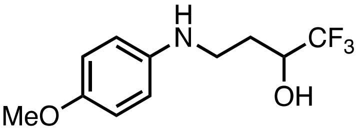

(a) 0.15 mmol scale: Following General Procedure A, the title compound was obtained as a yellow solid by preparative TLC using petroleum ether/EtOAc (5:1) as an eluent; R_f = 0.3 (petroleum ether/EtOAc = 2:1); 19.4 mg, 78% yield, m.p. 56 – 57 °C. **^{1}H NMR** (500 MHz, $CDCl_3$) δ 6.82 – 6.80 (m, 2H), 6.70 – 6.68 (m, 2H), 4.21 – 4.18 (m, 1H), 3.76 (s, 3H), 3.45 – 3.29 (m, 2H), 2.04 – 1.88 (m, 2H). (Due to the broadening effect and intermolecular hydrogen bonding, the proton signals of NH and OH could not be observed). **^{13}C NMR** (126 MHz, $CDCl_3$) δ 153.5, 141.5, 125.1 (q, $^1J_{CF}$ = 281.4 Hz), 116.0, 115.1, 70.3 (q, $^2J_{CF}$ = 31.3 Hz), 55.9, 42.7, 28.9. **^{19}F NMR** (471 MHz, $CDCl_3$) δ -79.89 (s, 3F). **HRMS** (ESI) m/z: $[M+H]^+$ Calcd for $C_{11}H_{15}NO_2F_3^+$ 250.1055; Found 250.1056.

(b) 8.0 mmol scale (Gram-scale synthesis):

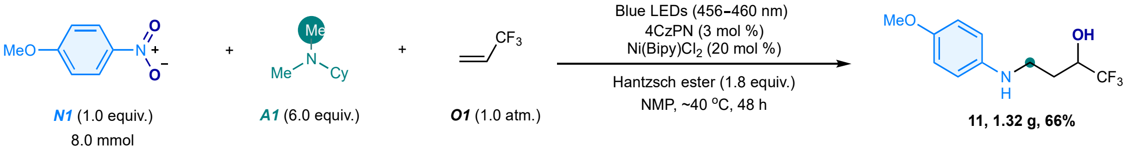

An oven-dried, transparent 150 mL Teflon screw-capped Thick-walled pressure-resistant bottle equipped with a stir bar was sequentially charged with 1-methoxy-4-nitrobenzene (1.0 equiv., 8.0 mmol, 1.2 g), 4CzPN (3 mol%, 0.24 mmol, 189.3 mg), Ni(Bipy)Cl_2 (20 mol%, 1.6 mmol, 457.3 mg**)**, and Hantzsch ester (**HE,** 1.8 equiv., 14.4 mmol, 3.7 g). Dried *N*-methyl-2-pyrrolidone (NMP, 80.0 mL) was then transferred into the tube via syringe. Subsequently, *N*,*N*-dimethylcyclohexylamine (6.0 equiv., 4.8 mmol, 7.2 mL) was transferred into the tube via syringe. The resulting mixture was degassed via blowing with two balloon filled with 3,3,3-trifluoropropene (***O1***, ~2 L) for 10 min, after which time the tube was quickly capped with a Teflon screw cap such that it was filled with 3,3,3-trifluoropropene in atmospheric pressure. The reaction mixture was vigorously stirred and irradiated using Kessil LEDs (456 nm, 40 W × 2) for 48 h, during which time the proximal temperature was controlled at approximately 40 °C via cooling with fans. At this point, the reaction mixture was diluted with ethyl acetate (~500 mL) and washed with water (~100 mL × 3). The organic fraction was further dried with anhydrous Na_2SO_4 and concentrated in vacuo with the aid of rotary evaporator. The residue was purified by column chromatography using the silica gel using petroleum ether and ethyl acetate (5:1) as an eluent to afford the **11** (1.32 g, 66%) (The experimental setup was shown below).

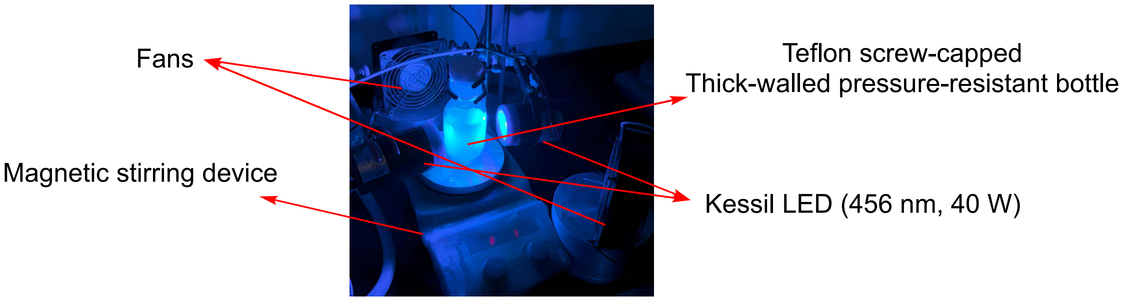

Fig. S10. Photograph of the reaction setup for the gram-scale synthesis.

1,1,1-trifluoro-4-((3-methoxyphenyl)amino)butan-2-ol (12)

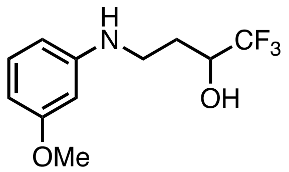

Following General Procedure A, the title compound was obtained as a brown oil by preparative TLC using petroleum ether/EtOAc (5:1) as an eluent; R_f = 0.3 (petroleum ether/EtOAc = 3:1); 18.4 mg, 74% yield. **^{1}H NMR** (400 MHz, $CDCl_3$) δ 7.11 (t, J = 8.1 Hz, 1H), 6.35 – 6.32 (m, 1H), 6.29 – 6.27 (m, 1H), 6.23 – 6.22 (m, 1H), 4.18 – 4.07 (m, 1H), 3.78 (s, 3H), 3.50 – 3.30 (m, 3H), 2.05 – 1.84 (m, 2H). (Due to the broadening effect and intermolecular hydrogen bonding, one of the proton signals of OH could not be observed). **^{13}C NMR** (201 MHz, $CDCl_3$) δ 161.0, 149.2, 130.3, 125.1 (q, $^1J_{CF}$ = 281.8 Hz), 106.7, 103.5, 99.7, 69.5 (q, $^2J_{CF}$ = 31.4 Hz), 55.3, 40.7, 29.1. **^{19}F NMR** (376 MHz, $CDCl_3$) δ -79.91 (s, 3F). **HRMS** (ESI) m/z: $[M+H]^+$ Calcd for $C_{11}H_{15}NO_2F_3^+$ 250.1055; Found 250.1059.

1,1,1-trifluoro-4-((2-methoxyphenyl)amino)butan-2-ol (13)

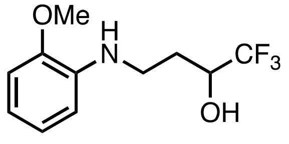

Following General Procedure A, the title compound was obtained as a yellow oil by preparative TLC using petroleum ether/EtOAc (5:1) as an eluent; R_f = 0.3 (petroleum ether/EtOAc = 3:1); 10.0 mg, 40% yield. **^{1}H NMR** (400 MHz, $CDCl_3$) δ 6.91 – 6.87 (m, 1H), 6.80 – 6.69 (m, 3H), 4.22 – 4.14 (m, 1H), 3.84 (s, 3H), 3.48 – 3.34 (m, 2H), 2.10 – 1.89 (m, 2H). (Due to the broadening effect and intermolecular hydrogen bonding, the proton signals of NH and OH could not be observed). **^{13}C NMR** (126 MHz, $CDCl_3$) δ 147.4, 137.6, 125.2 (q, $^1J_{CF}$ = 281.3 Hz), 121.4, 117.9, 111.0, 109.8, 69.7 (q, $^2J_{CF}$ = 31.4 Hz), 55.6, 40.6, 29.1. **^{19}F NMR** (376 MHz, $CDCl_3$) δ -79.87 (s, 3F). **HRMS** (ESI) m/z: $[M+H]^+$ Calcd for $C_{11}H_{15}NO_2F_3^+$ 250.1055; Found 250.1057.

4-((4-(benzyloxy)phenyl)amino)-1,1,1-trifluorobutan-2-ol (14)

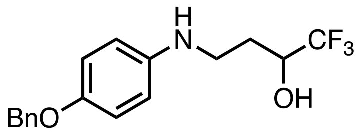

Following General Procedure A, the title compound was obtained as a brown solid by preparative TLC using petroleum ether/EtOAc (5:1) as an eluent; R_f = 0.4 (petroleum ether/EtOAc = 3:1); 21.5 mg, 66% yield, m.p. 116 – 117 ºC. **^{1}H NMR** (400 MHz, $CDCl_3$) δ 7.44 – 7.30 (m, 5H), 6.89 – 6.86 (m, 2H), 6.69 – 6.63 (m, 2H), 5.01 (s,

2H), 4.22 – 4.14 (m, 1H), 3.48 – 3.28 (m, 3H), 2.05 – 1.85 (m, 2H). (Due to the broadening effect and intermolecular hydrogen bonding, one of the proton signals of OH could not be observed). **^{13}C NMR** (126 MHz, $CDCl_3$) δ 152.6, 141.7, 137.5, 128.7, 128.0, 127.6, 125.1 (q, $^1J_{CF}$ = 281.6 Hz), 116.3, 115.8, 70.9, 70.2 (q, $^2J_{CF}$ = 31.3 Hz), 42.6, 28.9. **^{19}F NMR** (376 MHz, $CDCl_3$) δ -79.86 (s, 3F). **HRMS** (ESI) m/z: $[M+H]^+$ Calcd for $C_{17}H_{19}NO_2F_3^+$ 326.1368; Found 326.1367.

1,1,1-trifluoro-4-((4-phenoxyphenyl)amino)butan-2-ol (15)

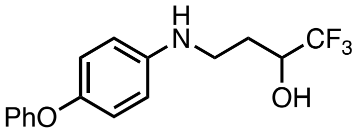

Following General Procedure A, the title compound was obtained as a brown solid by preparative TLC using petroleum ether/EtOAc (5:1) as an eluent; R$_f$ = 0.4 (petroleum ether/EtOAc = 3:1); 22.4 mg, 72% yield, m.p. 66 – 67 °C. **^{1}H NMR** (500 MHz, $CDCl_3$) δ 7.30-7.25 (m, 2H), 7.02 (t, J = 7.4 Hz, 1H), 6.94 – 6.92 (m, 4H), 6.68 (d, J = 8.3 Hz, 2H), 4.22 – 4.15 (m, 1H), 3.46 – 3.32 (m, 3H), 2.07 – 1.89 (m, 2H). (Due to the broadening effect and intermolecular hydrogen bonding, one of the proton signals of OH could not be observed). **^{13}C NMR** (126 MHz, $CDCl_3$) δ 158.8, 149.0, 144.0, 129.7, 125.1 (q, $^1J_{CF}$ = 281.5 Hz), 122.4, 121.3, 117.5, 115.1, 69.9 (q, $^2J_{CF}$ = 31.4 Hz), 41.8, 28.9. **^{19}F NMR** (471 MHz, $CDCl_3$) δ -79.88 (s, 3F). **HRMS** (ESI) m/z: $[M+H]^+$ Calcd for $C_{16}H_{17}NO_2F_3^+$ 312.1211; Found 312.1212.

4-((4-(*tert*-butyl)phenyl)amino)-1,1,1-trifluorobutan-2-ol (16)

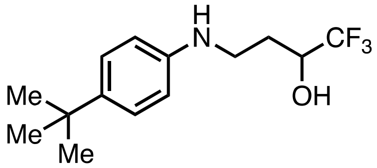

Following General Procedure A, the title compound was obtained as a yellow oil by preparative TLC using petroleum ether/EtOAc (5:1) as an eluent; R$_f$ = 0.4 (petroleum ether/EtOAc = 5:1); 21.5 mg, 78% yield. **^{1}H NMR** (400 MHz, $CDCl_3$) δ 7.24 (d, J = 8.6 Hz, 2H), 6.65 (d, J = 8.6 Hz, 2H), 4.20 – 4.12 (m, 1H), 3.46 – 3.31 (m, 3H), 2.05 – 1.85 (m, 2H), 1.28 (s, 9H). (Due to the broadening effect and intermolecular hydrogen bonding, one of the proton signals of OH could not be observed). **^{13}C NMR** (126 MHz, $CDCl_3$) δ 145.3, 141.9, 126.3, 125.1 (q, $^1J_{CF}$ = 281.6 Hz), 113.8, 70.0 (q,

$^{2}J_{CF}$ = 31.3 Hz), 41.5, 34.1, 31.6, 29.0. **^{19}F NMR** (376 MHz, $CDCl_3$) δ -79.85 (s, 3F). **HRMS** (ESI) m/z: $[M+H]^+$ Calcd for $C_{14}H_{21}NOF_3^+$ 276.1575; Found 276.1577.

4-([1,1'-biphenyl]-4-ylamino)-1,1,1-trifluorobutan-2-ol (17)

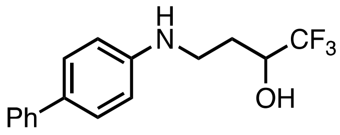

Following General Procedure A, the title compound was obtained as a yellow solid by preparative TLC using petroleum ether/EtOAc (5:1) as an eluent; R$_f$ = 0.4 (petroleum ether/EtOAc = 3:1); 21.6 mg, 73% yield, m.p. 112 – 113 ºC. **^{1}H NMR** (500 MHz, $CDCl_3$) δ 7.53 (d, J = 7.6 Hz, 2H), 7.46 (d, J = 8.1 Hz, 2H), 7.40 (t, J = 7.7 Hz, 2H), 7.29 – 7.25 (m, 1H), 6.74 (d, J = 8.0 Hz, 2H), 4.20 – 4.14 (m, 1H), 3.50 – 3.37 (m, 2H), 3.25 (brs, 1H), 2.08 – 1.89 (m, 2H). (Due to the broadening effect and intermolecular hydrogen bonding, one of the proton signals of OH could not be observed). **^{13}C NMR** (126 MHz, $CDCl_3$) δ 147.0, 141.1, 131.7, 128.8, 128.2, 126.5, 126.5, 125.1 (q, $^{1}J_{CF}$ = 281.3 Hz), 114.0, 69.7 (q, $^{2}J_{CF}$ = 31.5 Hz), 40.9, 29.1. **^{19}F NMR** (471 MHz, $CDCl_3$) δ -79.88 (s, 3F). **HRMS** (ESI) m/z: $[M+H]^+$ Calcd for $C_{16}H_{17}NOF_3^+$ 296.1262; Found 296.1263.

1,1,1-trifluoro-4-((4-(methylthio)phenyl)amino)butan-2-ol (18)

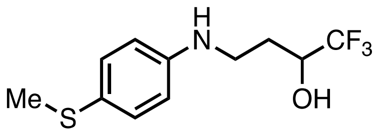

Following General Procedure A, the title compound was obtained as a yellow solid by preparative TLC using petroleum ether/EtOAc (5:1) as an eluent; R$_f$= 0.4 (petroleum ether/EtOAc = 3:1); 13.3 mg, 50% yield, m.p. 86 – 87 ºC. **^{1}H NMR** (500 MHz, $CDCl_3$) δ 7.22 (d, J = 8.9 Hz, 2H), 6.62 (d, J = 8.7 Hz, 2H), 4.18 – 4.11 (m, 1H), 3.44 – 3.32 (m, 2H), 3.01 (brs, 1H), 2.42 (s, 3H), 2.05 – 1.86 (m, 2H). (Due to the broadening effect and intermolecular hydrogen bonding, one of the proton signals of OH could not be observed). **^{13}C NMR** (126 MHz, $CDCl_3$) δ 146.4, 131.3, 125.9, 125.1 (q, $^{1}J_{CF}$ = 281.7 Hz), 114.3, 69.6 (q, $^{2}J_{CF}$ = 31.4 Hz), 40.8, 29.0, 18.9. **^{19}F NMR** (471 MHz, $CDCl_3$) δ -79.92 (s, 3F). **HRMS** (ESI) m/z: $[M+H]^+$ Calcd for $C_{11}H_{15}NOF_3S^+$ 266.0826; Found 266.0825.

***Tert*-butyl (4-((4,4,4-trifluoro-3-hydroxybutyl)amino)phenyl)carbamate (19)**

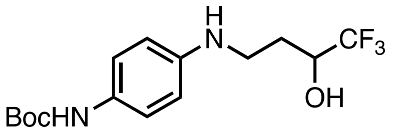

Following General Procedure A, the title compound was obtained as a brown solid by preparative TLC using petroleum ether/EtOAc (3:1) as an eluent; R*f* = 0.3 (petroleum ether/EtOAc = 2:1); 24.7 mg, 74% yield, m.p. 79 – 80 ºC. **^{1}H NMR** (500 MHz, $CDCl_3$) δ 7.08 – 7.04 (m, 1H), 6.98 (brs, 1H), 6.58 – 6.52 (m, 1H), 6.46 (d, *J* = 8.0 Hz, 1H), 6.32 (d, *J* = 8.1 Hz, 1H), 4.14 – 4.04 (m, 1H), 3.46 – 3.28 (m, 3H), 1.95 – 1.81 (m, 2H), 1.50 (s, 9H). (Due to the broadening effect and intermolecular hydrogen bonding, one of the proton signals of OH could not be observed). **^{13}C NMR** (201 MHz, $CDCl_3$) δ 153.2, 148.9, 139.4, 129.8, 125.3 (q, $^{1}J_{CF}$ = 281.8 Hz), 108.6, 80.8, 68.9 (q, $^{2}J_{CF}$ = 31.2 Hz), 40.0, 29.8, 28.4. **^{19}F NMR** (471 MHz, $CDCl_3$) δ -79.92 (s, 3F). **HRMS** (ESI) m/z: $[M+H]^+$ Calcd for $C_{15}H_{22}N_2O_3F_3^+$ 335.1583; Found 335.1581.

1,1,1-trifluoro-4-((4-fluorophenyl)amino)butan-2-ol (20)

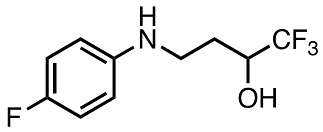

Following General Procedure A, the title compound was obtained as a brown oil by preparative TLC using petroleum ether/EtOAc (5:1) as an eluent; R*f* = 0.4 (petroleum ether/EtOAc = 3:1); 14.2 mg, 60% yield. **^{1}H NMR** (500 MHz, $CDCl_3$) δ 6.93 – 6.90 (m, 2H), 6.63 – 6.61 (m, 2H), 4.20 – 4.14 (m, 1H), 3.44 – 3.30 (m, 3H), 2.05 – 1.88 (m, 2H). (Due to the broadening effect and intermolecular hydrogen bonding, one of the proton signals of OH could not be observed). **^{13}C NMR** (201 MHz, $CDCl_3$) δ 156.7 (d, $^{1}J_{CF}$ = 236.9 Hz), 144.0, 125.1 (q, $^{1}J_{CF}$ = 281.5 Hz), 116.0 (d, $^{2}J_{CF}$ = 22.3 Hz), 114.9 (d, $^{3}J_{CF}$ = 7.5 Hz), 69.8 (q, $^{2}J_{CF}$ = 31.6 Hz), 41.8, 29.0. **^{19}F NMR** (471 MHz, $CDCl_3$) δ -79.93 (s, 3F), -126.34 (s, 1F). **HRMS** (ESI) m/z: $[M+H]^+$ Calcd for $C_{10}H_{12}NOF_4^+$ 238.0855; Found 238.0858.

4-((4-chlorophenyl)amino)-1,1,1-trifluorobutan-2-ol (21)

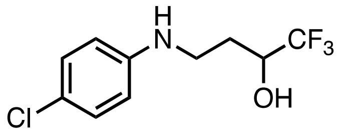

Following General Procedure A, the title compound was obtained as a brown oil by preparative TLC using petroleum ether/EtOAc (5:1) as an eluent; R*f* = 0.4 (petroleum ether/EtOAc = 3:1); 11.1 mg, 44% yield. **^{1}H NMR** (500 MHz, $CDCl_3$) δ 7.14 (d, J = 8.7 Hz, 2H), 6.58 (d, J = 8.6 Hz, 2H), 4.18 – 4.13 (m, 1H), 3.47 – 3.30 (m, 3H), 2.05 – 1.87 (m, 2H). (Due to the broadening effect and intermolecular hydrogen bonding, one of the proton signals of OH could not be observed). **^{13}C NMR** (126 MHz, $CDCl_3$) δ 146.4, 129.3, 125.1 (q, $^1J_{CF}$ = 281.7 Hz), 123.1, 114.6, 69.5 (q, $^2J_{CF}$ = 31.5 Hz), 40.8, 29.0. **^{19}F NMR** (471 MHz, $CDCl_3$) δ -79.95 (s, 3F). **HRMS** (ESI) m/z: $[M+H]^+$ Calcd for $C_{10}H_{12}NOF_3Cl^+$ 254.0560; Found 254.0562.

4-((4-bromophenyl)amino)-1,1,1-trifluorobutan-2-ol (22)

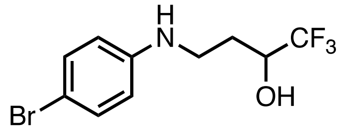

Following General Procedure A, the title compound was obtained as a light green oil by preparative TLC using petroleum ether/EtOAc (5:1) as an eluent; R*f* = 0.4 (petroleum ether/EtOAc = 3:1); 14.0 mg, 47% yield. **^{1}H NMR** (500 MHz, $CDCl_3$) δ 7.27 (d, J = 8.7 Hz, 2H), 6.53 (d, J = 8.2 Hz, 2H), 4.16 – 4.12 (m, 1H), 3.42 – 3.30 (m, 3H), 2.05 – 1.86 (m, 2H). (Due to the broadening effect and intermolecular hydrogen bonding, one of the proton signals of OH could not be observed). **^{13}C NMR** (126 MHz, $CDCl_3$) δ 146.8, 132.2, 125.1 (q, $^1J_{CF}$ = 281.7 Hz), 115.0, 110.1, 69.5 (q, $^2J_{CF}$ = 31.6 Hz), 40.6, 29.0. **^{19}F NMR** (471 MHz, $CDCl_3$) δ -79.96 (s, 3F). **HRMS** (ESI) m/z: $[M+H]^+$ Calcd for $C_{10}H_{12}NOF_3Br^+$ 298.0054; Found 298.0058.

4-((4,4,4-trifluoro-3-hydroxybutyl)amino)phenyl acetate (23)

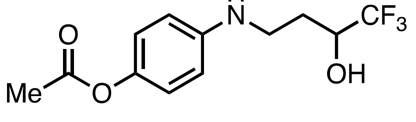

Following General Procedure A, the title compound was obtained as a red solid by preparative TLC using petroleum ether/EtOAc (5:1) as an eluent; R*f* = 0.3 (petroleum ether/EtOAc = 3:1); 10.2 mg, 37% yield, m.p. 77 – 78 ºC. **^{1}H NMR** (500 MHz, $CDCl_3$) δ 6.92 – 6.90 (m, 2H), 6.64 – 6.62 (m, 2H), 4.15 – 4.10 (m, 1H), 3.65 (brs, 1H), 3.42 – 3.29 (m, 2H), 2.27 (s, 3H), 2.05 – 1.85 (m, 2H). (Due to the broadening

effect and intermolecular hydrogen bonding, one of the proton signals of OH could not be observed). **^{13}C NMR** (201 MHz, $CDCl_3$) δ 170.5, 145.8, 142.8, 125.1 (q, $^1J_{CF}$ = 281.9 Hz), 122.4, 114.0, 69.6 (q, $^2J_{CF}$ = 31.5 Hz), 41.1, 29.0, 21.2. **^{19}F NMR** (471 MHz, $CDCl_3$) δ -79.90 (s, 3F). **HRMS** (ESI) m/z: $[M+H]^+$ Calcd for $C_{12}H_{15}NO_3F_3^+$ 278.1004; Found 278.1005.

4-((4-(difluoromethoxy)phenyl)amino)-1,1,1-trifluorobutan-2-ol (24)

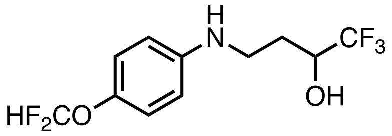

Following General Procedure A, the title compound was obtained as a brown oil by preparative TLC using petroleum ether/EtOAc (5:1) as an eluent; R_f = 0.3 (petroleum ether/EtOAc = 3:1); 15.7 mg, 55% yield. **^{1}H NMR** (500 MHz, $CDCl_3$) δ 6.99 (d, J = 8.3 Hz, 2H), 6.62 (d, J = 8.3 Hz, 2H), 6.39 (t, J = 74.4 Hz, 1H), 4.19 – 4.13 (m, 1H), 3.44 – 3.31 (m, 2H), 3.08 (brs, 1H), 2.05 – 1.87 (m, 2H). (Due to the broadening effect and intermolecular hydrogen bonding, one of the proton signals of OH could not be observed). **^{13}C NMR** (126 MHz, $CDCl_3$) δ 145.7, 143.2 (t, $^2J_{CF}$ = 3.0 Hz), 125.1 (q, $^1J_{CF}$ = 281.6 Hz), 121.7, 116.5 (t, $^1J_{CF}$ = 259.2 Hz), 114.3, 69.6 (q, $^2J_{CF}$ = 31.5 Hz), 41.1, 29.0. **^{19}F NMR** (471 MHz, $CDCl_3$) δ -79.95 (s, 3F), -80.18 (s, 2F). **HRMS** (ESI) m/z: $[M+H]^+$ Calcd for $C_{11}H_{13}NO_2F_5^+$ 286.0866; Found 286.0867.

1,1,1-trifluoro-4-((4-(trifluoromethoxy)phenyl)amino)butan-2-ol (25)

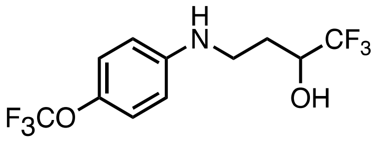

Following General Procedure A, the title compound was obtained as a red oil by preparative TLC using petroleum ether/EtOAc (5:1) as an eluent; R_f = 0.3 (petroleum ether/EtOAc = 3:1); 20.9 mg, 69% yield. **^{1}H NMR** (500 MHz, $CDCl_3$) δ 7.06 (d, J = 8.4 Hz, 2H), 6.62 (d, J = 8.4 Hz, 2H), 4.19 – 4.12 (m, 1H), 3.45 – 3.32 (m, 2H), 3.10 (brs, 1H), 2.06 – 1.88 (m, 2H). (Due to the broadening effect and intermolecular hydrogen bonding, one of the proton signals of OH could not be observed). **^{13}C NMR** (126 MHz, $CDCl_3$) δ 146.6, 141.2, 125.1 (q, $^1J_{CF}$ = 281.6 Hz), 122.7, 120.8 (q, $^1J_{CF}$ = 255.5 Hz), 113.8, 69.6 (q, $^2J_{CF}$ = 31.5 Hz), 40.8, 29.0. **^{19}F NMR**

(471 MHz, $CDCl_3$) δ -58.48 (s, 3F), -79.97 (s, 3F). **HRMS** (ESI) m/z: $[M+H]^+$ Calcd for $C_{11}H_{12}NO_2F_6^+$ 304.0772; Found 304.0775.

1,1,1-trifluoro-4-(*p*-tolylamino)butan-2-ol (26)

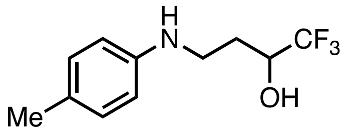

Following General Procedure A, the title compound was obtained as a colorless crystalline solid by preparative TLC using petroleum ether/EtOAc (5:1) as an eluent; R_f = 0.4 (petroleum ether/EtOAc = 3:1); 18.8 mg, 81% yield, m.p. 64 – 65 ºC. **^{1}H NMR** (500 MHz, $CDCl_3$) δ 7.03 (d, J = 7.8 Hz, 2H), 6.62 (d, J = 7.8 Hz, 2H), 4.20 – 4.13 (m, 1H), 3.45 – 3.31 (m, 3H), 2.26 (s, 3H), 2.05 – 1.89 (m, 2H). (Due to the broadening effect and intermolecular hydrogen bonding, one of the proton signals of OH could not be observed). **^{13}C NMR** (201 MHz, $CDCl_3$) δ 145.3, 130.0, 128.4, 125.1 (q, $^1J_{CF}$ = 281.7 Hz), 114.3, 69.9 (q, $^2J_{CF}$ = 31.5 Hz), 41.6, 29.0, 20.5. **^{19}F NMR** (471 MHz, $CDCl_3$) δ -79.88 (s, 3F). **HRMS** (ESI) m/z: $[M+H]^+$ Calcd for $C_{11}H_{15}NOF_3^+$ 234.1106; Found 234.1108.

1,1,1-trifluoro-4-(o-tolylamino)butan-2-ol (27)

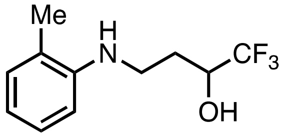

Following General Procedure A, the title compound was obtained as a brown oil by preparative TLC using petroleum ether/EtOAc (5:1) as an eluent; R_f = 0.4 (petroleum ether/EtOAc = 3:1); 10.5 mg, 45% yield. **^{1}H NMR** (500 MHz, $CDCl_3$) δ 7.16 (t, J = 7.9 Hz, 1H), 7.09 (d, J = 7.3 Hz, 1H), 6.75 – 6.69 (m, 2H), 4.21 – 4.16 (m, 1H), 3.52 – 3.32 (m, 3H), 2.15 (s, 3H), 2.11 – 1.93 (m, 2H). (Due to the broadening effect and intermolecular hydrogen bonding, one of the proton signals of OH could not be observed). **^{13}C NMR** (126 MHz, $CDCl_3$) δ 145.7, 130.4, 127.4, 125.1 (q, $^1J_{CF}$ = 281.5 Hz), 123.1, 118.3, 110.6, 70.0 (q, $^2J_{CF}$ = 31.4 Hz), 40.9, 29.0, 17.6. **^{19}F NMR** (471 MHz, $CDCl_3$) δ -79.95 (s, 3F). **HRMS** (ESI) m/z: $[M+H]^+$ Calcd for $C_{11}H_{15}NOF_3^+$ 234.1106; Found 234.1107.

1,1,1-trifluoro-4-((4-(4,4,5,5-tetramethyl-1,3,2-dioxaborolan-2-yl)phenyl)amino)

butan-2-ol (28)

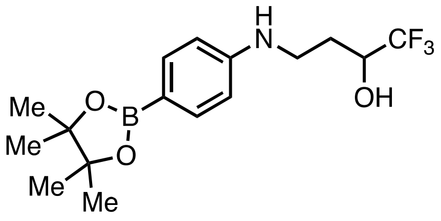

Following General Procedure A, the title compound was obtained as a yellow solid by preparative TLC using petroleum ether/EtOAc (5:1) as an eluent; R_f = 0.3 (petroleum ether/EtOAc = 3:1); 16.3 mg, 47% yield, m.p. 82 – 83 ºC. **^{1}H NMR** (500 MHz, $CDCl_3$) δ 7.66 – 7.61 (m, 2H), 6.67 – 6.61 (m, 2H), 4.14 – 4.11 (m, 1H), 3.48 – 3.36 (m, 2H), 2.05 – 1.87 (m, 2H), 1.32 (s, 12H). (Due to the broadening effect and intermolecular hydrogen bonding, the proton signals of NH and OH could not be observed). **^{13}C NMR** (201 MHz, $CDCl_3$) δ 150.4, 136.6, 129.5, 125.1 (q, $^1J_{CF}$ = 281.6 Hz), 112.3, 83.5, 69.4 (q, $^2J_{CF}$ = 31.3 Hz), 39.9, 29.1, 25.0. **^{19}F NMR** (471 MHz, $CDCl_3$) δ -79.96 (s, 3F). **HRMS** (ESI) m/z: $[M+H]^+$ Calcd for $C_{16}H_{24}NO_3F_3B^+$ 346.1801; Found 346.1802.

1,1,1-trifluoro-4-((3-(4,4,5,5-tetramethyl-1,3,2-dioxaborolan-2-yl)phenyl)amino) butan-2-ol (29)

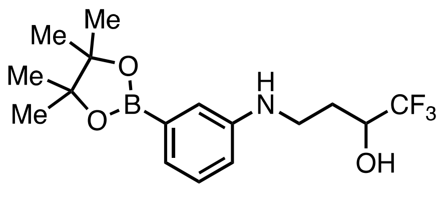

Following General Procedure A, the title compound was obtained as a yellow oil by preparative TLC using petroleum ether/EtOAc (5:1) as an eluent; R_f = 0.3 (petroleum ether/EtOAc = 3:1); 10.0 mg, 28% yield. **^{1}H NMR** (500 MHz, $CDCl_3$) δ 7.26 – 7.21 (m, 2H), 7.13 (s, 1H), 6.80 – 6.78 (m, 1H), 4.17 – 4.14 (m, 1H), 3.49 – 3.36 (m, 2H), 2.04 – 1.88 (m, 2H), 1.34 (s, 12H). (Due to the broadening effect and intermolecular hydrogen bonding, the proton signals of NH and OH could not be observed). **^{13}C NMR** (201 MHz, $CDCl_3$) δ 147.1, 129.0, 125.3, 125.1 (q, $^1J_{CF}$ = 281.6 Hz), 121.3, 120.0, 116.7, 83.9, 69.7 (q, $^2J_{CF}$ = 31.5 Hz), 41.1, 29.1, 25.0. **^{19}F NMR** (471 MHz, $CDCl_3$) δ -79.91 (s, 3F). **HRMS** (ESI) m/z: $[M+H]^+$ Calcd for $C_{16}H_{24}BNO_3F_3^+$ 346.1801; Found 346.1803.

1,1,1-trifluoro-4-((4-(trifluoromethyl)phenyl)amino)butan-2-ol (30)

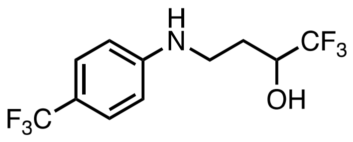

Following General Procedure A, the title compound was obtained as a yellow oil by preparative TLC using petroleum ether/EtOAc (5:1) as an eluent; R_f = 0.3 (petroleum ether/EtOAc = 3:1); 10.0 mg, 35% yield. **^{1}H NMR** (500 MHz, $CDCl_3$) δ 7.42 (d, J = 8.3 Hz, 2H), 6.64 (d, J = 8.2 Hz, 2H), 4.30 – 4.12 (m, 2H), 3.49 – 3.37 (m, 2H), 2.08 – 1.90 (m, 2H). (Due to the broadening effect and intermolecular hydrogen bonding, one of the proton signals of OH could not be observed). **^{13}C NMR** (201 MHz, $CDCl_3$) δ 150.4, 126.9 (q, $^3J_{CF}$ = 3.7 Hz), 125.0 (q, $^1J_{CF}$ = 282.1 Hz), 125.0 (q, $^1J_{CF}$ = 270.6 Hz), 119.6 (q, $^2J_{CF}$ = 33.0 Hz), 112.2, 69.3 (q, $^2J_{CF}$ = 31.8 Hz), 39.9, 29.0. **^{19}F NMR** (471 MHz, $CDCl_3$) δ -61.11 (s, 3F), -80.02 (s, 3F). **HRMS** (ESI) m/z: $[M+H]^+$ Calcd for $C_{11}H_{12}NOF_6^+$ 288.0823; Found 288.0825.

1,1,1-trifluoro-4-((3-(trifluoromethyl)phenyl)amino)butan-2-ol (31)

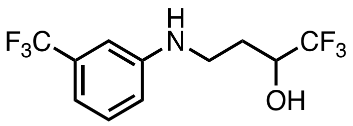

Following General Procedure A, the title compound was obtained as a yellow oil by preparative TLC using petroleum ether/EtOAc (5:1) as an eluent; R_f = 0.3 (petroleum ether/EtOAc = 3:1); 12.3 mg, 43% yield. **^{1}H NMR** (500 MHz, $CDCl_3$) δ 7.29 – 7.26 (m, 1H), 6.98 (d, J = 7.6 Hz, 1H), 6.84 (s, 1H), 6.78 (d, J = 8.3 Hz, 1H), 4.17 – 4.12 (m, 1H), 3.48 – 3.35 (m, 2H), 2.84 (brs, 1H), 2.07 – 1.89 (m, 2H). (Due to the broadening effect and intermolecular hydrogen bonding, one of the proton signals of OH could not be observed). **^{13}C NMR** (201 MHz, $CDCl_3$) δ 148.1, 131.8 (q, $^2J_{CF}$ = 31.8 Hz), 129.9, 125.1 (q, $^1J_{CF}$ = 281.6 Hz), 124.4 (q, $^1J_{CF}$ = 272.5 Hz), 116.2, 114.6 (q, $^3J_{CF}$ = 4.0 Hz), 109.4 (d, $^3J_{CF}$ = 3.7 Hz), 69.4 (q, $^2J_{CF}$ = 31.7 Hz), 40.3, 29.0. **^{19}F NMR** (471 MHz, $CDCl_3$) δ -62.89 (s, 3F), -79.98 (s, 3F). **HRMS** (ESI) m/z: $[M+H]^+$ Calcd for $C_{11}H_{12}NOF_6^+$ 288.0823; Found 288.0824.

4-((4,4,4-trifluoro-3-hydroxybutyl)amino)benzaldehyde (32)

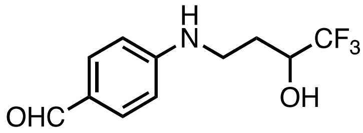

Following General Procedure A, the title compound was obtained as a red oil by preparative TLC using petroleum ether/EtOAc (5:1) as an eluent; R_f = 0.3 (petroleum

ether/EtOAc = 3:1); 12.6 mg, 51% yield. (2-(4-Nitrophenyl)-1,3-dioxolane is used as nitroarene substrate, the ethylene glycol protecting group is cleaved directly to generate the aldehyde product under the reaction conditions). **^{1}H NMR** (500 MHz, $CDCl_3$) δ 9.63 (s, 1H), 7.65 (d, J = 8.3 Hz, 2H), 6.61 (d, J = 8.3 Hz, 2H), 4.87 (brs, 1H), 4.16 – 4.09 (m, 1H), 4.00 (brs, 1H), 3.53 – 3.40 (m, 2H), 2.07 – 1.90 (m, 2H). (Due to the broadening effect and intermolecular hydrogen bonding, one of the proton signals of OH could not be observed). **^{13}C NMR** (126 MHz, $CDCl_3$) δ 191.0, 153.5, 132.8, 126.4, 125.1 (q, $^1J_{CF}$ = 281.9 Hz), 112.0, 68.9 (q, $^2J_{CF}$ = 31.4 Hz), 39.5, 28.9. **^{19}F NMR** (471 MHz, $CDCl_3$) δ -79.88 (s, 3F). **HRMS** (ESI) m/z: $[M+H]^+$ Calcd for $C_{11}H_{13}NO_2F_3^+$ 248.0898; Found 248.0896.

1-(3-((4,4,4-trifluoro-3-hydroxybutyl)amino)phenyl)propan-1-one (33)

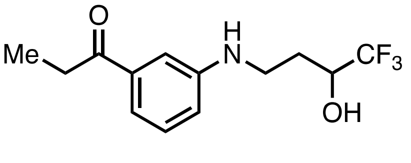

Following General Procedure A, the title compound was obtained as a light green solid by preparative TLC using petroleum ether/EtOAc (5:1) as an eluent; R_f = 0.3 (petroleum ether/EtOAc = 3:1); 16.8 mg, 61% yield, m.p. 86 – 87 ºC. **^{1}H NMR** (800 MHz, $CDCl_3$) δ 7.32 (d, J = 7.6 Hz, 1H), 7.27 – 7.25 (m, 1H), 7.23 (s, 1H), 6.84 – 6.82 (m, 1H), 4.17 – 4.13 (m, 1H), 3.49 – 3.39 (m, 3H), 2.97 (q, J = 7.2 Hz, 2H), 2.06 – 1.91 (m, 2H), 1.20 (t, J = 7.2 Hz, 3H). (Due to the broadening effect and intermolecular hydrogen bonding, one of the proton signals of OH could not be observed). **^{13}C NMR** (201 MHz, $CDCl_3$) δ 201.8, 148.2, 138.1, 129.6, 125.1 (q, $^1J_{CF}$ = 281.8 Hz), 118.2, 118.0, 111.9, 69.4 (q, $^2J_{CF}$ = 31.6 Hz), 40.4, 32.0, 29.1, 8.5. **^{19}F NMR** (471 MHz, $CDCl_3$) δ -79.89 (s, 3F). **HRMS** (ESI) m/z: $[M+H]^+$ Calcd for $C_{13}H_{17}NO_2F_3^+$ 276.1211; Found 276.1212.

1,1,1-trifluoro-4-((4-((trimethylsilyl)ethynyl)phenyl)amino)butan-2-ol (34)

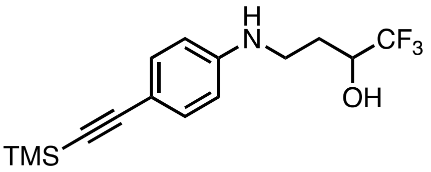

Following General Procedure A, the title compound was obtained as a red oil by preparative TLC using petroleum ether/EtOAc (5:1) as an eluent; R_f = 0.5 (petroleum ether/EtOAc = 3:1); 11.7 mg, 37% yield. **^{1}H NMR** (500 MHz, $CDCl_3$) δ

7.30 (d, J = 8.1 Hz, 2H), 6.53 (d, J = 8.1 Hz, 2H), 4.14 – 4.10 (m, 1H), 4.00 (brs, 1H), 3.42 – 3.34 (m, 2H), 2.05 – 1.89 (m, 2H), 0.22 (s, 9H). (Due to the broadening effect and intermolecular hydrogen bonding, one of the proton signals of OH could not be observed). **^{13}C NMR** (126 MHz, $CDCl_3$) δ 148.0, 133.6, 125.1 (q, $^1J_{CF}$ = 281.8 Hz), 112.7, 112.1, 106.1, 91.6, 69.4 (q, $^2J_{CF}$ = 31.6 Hz), 40.1, 29.0, 0.3. **^{19}F NMR** (471 MHz, $CDCl_3$) δ -80.00 (s, 3F). **HRMS** (ESI) m/z: $[M+H]^+$ Calcd for $C_{15}H_{21}NOF_3Si^+$ 316.1345; Found 316.1345.

4-((3,4-dimethoxyphenyl)amino)-1,1,1-trifluorobutan-2-ol (35)

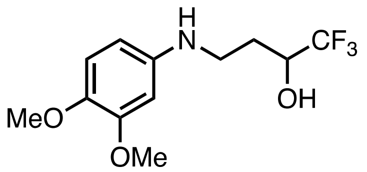

Following General Procedure A, the title compound was obtained as a brown oil by preparative TLC using petroleum ether/EtOAc (5:1) as an eluent; R$_f$ = 0.4 (petroleum ether/EtOAc = 3:1); 17.3 mg, 62% yield. **^{1}H NMR** (500 MHz, $CDCl_3$) δ 6.77 – 6.75 (m, 1H), 6.31 (s, 1H), 6.25 – 6.23 (m, 1H), 4.21 – 4.14 (m, 1H), 3.83 (s, 3H), 3.81 (s, 3H), 3.53 (brs, 1H), 3.43 – 3.25 (m, 2H), 2.03 – 1.87 (m, 2H). (Due to the broadening effect and intermolecular hydrogen bonding, one of the proton signals of OH could not be observed). **^{13}C NMR** (101 MHz, $CDCl_3$) δ 150.1, 142.8, 142.2, 125.1 (q, $^1J_{CF}$ = 281.7 Hz), 113.0, 105.1, 100.4, 70.0 (q, $^2J_{CF}$ = 31.4 Hz), 56.7, 55.9, 42.3, 28.9. **^{19}F NMR** (471 MHz, $CDCl_3$) δ -79.87 (s, 3F). **HRMS** (ESI) m/z: $[M+H]^+$ Calcd for $C_{12}H_{17}NO_3F_3^+$ 280.1161; Found 280.1163.

1,1,1-trifluoro-4-((4-methoxy-3-(trifluoromethyl)phenyl)amino)butan-2-ol (36)

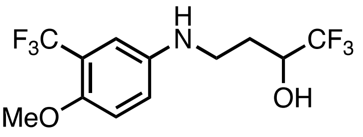

Following General Procedure A, the title compound was obtained as a yellow solid by preparative TLC using petroleum ether/EtOAc (5:1) as an eluent; R$_f$ = 0.3 (petroleum ether/EtOAc = 3:1); 20.9 mg, 66% yield, m.p. 61 – 62 ºC. **^{1}H NMR** (500 MHz, $CDCl_3$) δ 6.91 – 6.89 (m, 2H), 6.82 – 6.79 (m, 1H), 4.20 – 4.13 (m, 1H), 3.83 (s, 3H), 3.43 – 3.29 (m, 3H), 2.05 – 1.87 (m, 2H). (Due to the broadening effect and intermolecular hydrogen bonding, one of the proton signals of OH could not be observed). **^{13}C NMR** (126 MHz, $CDCl_3$) δ 150.5, 141.3, 125.1 (q, $^1J_{CF}$ = 281.7 Hz),

123.7 (q, ${}^{1}J_{CF}$ = 272.6 Hz), 119.7 (q, ${}^{2}J_{CF}$ = 30.7 Hz), 118.0, 114.3, 112.8 (q, ${}^{3}J_{CF}$ = 5.4 Hz), 69.7 (q, ${}^{2}J_{CF}$ = 31.4 Hz), 56.9, 41.7, 28.9. **^{19}F NMR** (471 MHz, $CDCl_3$) δ -62.29 (s, 3F), -79.94 (s, 3F). **HRMS** (ESI) m/z: $[M+H]^+$ Calcd for $C_{12}H_{14}NO_2F_6^+$ 318.0929; Found 318.0927.

2-methoxy-5-((4,4,4-trifluoro-3-hydroxybutyl)amino)benzonitrile (37)

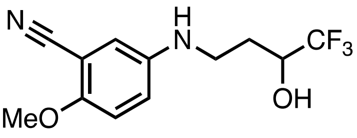

Following General Procedure A, the title compound was obtained as a yellow solid by preparative TLC using petroleum ether/EtOAc (5:1) as an eluent; R_f = 0.3 (petroleum ether/EtOAc = 3:1); 17.5 mg, 64% yield, m.p. 73 – 74 ºC. **^{1}H NMR** (500 MHz, $CDCl_3$) δ 6.85 – 6.84 (m, 2H), 6.80 (s, 1H), 4.18 – 4.12 (m, 1H), 3.85 (s, 3H), 3.39 – 3.27 (m, 3H), 2.03 – 1.87 (m, 2H). (Due to the broadening effect and intermolecular hydrogen bonding, one of the proton signals of OH could not be observed). **^{13}C NMR** (126 MHz, $CDCl_3$) δ 154.5, 141.9, 125.1 (q, ${}^{1}J_{CF}$ = 281.6 Hz), 120.1, 117.2, 117.0, 113.1, 102.0, 69.4 (q, ${}^{2}J_{CF}$ = 31.6 Hz), 56.6, 41.2, 28.9. **^{19}F NMR** (471 MHz, $CDCl_3$) δ -79.90 (s, 3F). **HRMS** (ESI) m/z: $[M+H]^+$ Calcd for $C_{12}H_{14}N_2O_2F_3^+$ 275.1007; Found 275.1008.

1,1,1-trifluoro-4-((4-methoxy-3,5-dimethylphenyl)amino)butan-2-ol (38)

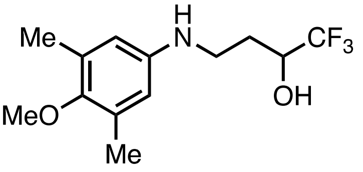

Following General Procedure A, the title compound was obtained as a yellow oil by preparative TLC using petroleum ether/EtOAc (5:1) as an eluent; R_f = 0.3 (petroleum ether/EtOAc = 3:1); 15.1 mg, 54% yield. **^{1}H NMR** (500 MHz, $CDCl_3$) δ 6.36 (s, 2H), 4.19 – 4.13 (m, 1H), 3.66 (s, 3H), 3.41 – 3.27 (m, 3H), 2.23 (s, 6H), 2.03 – 1.85 (m, 2H). (Due to the broadening effect and intermolecular hydrogen bonding, one of the proton signals of OH could not be observed). **^{13}C NMR** (126 MHz, $CDCl_3$) δ 150.1, 143.6, 131.8, 125.1 (q, ${}^{1}J_{CF}$ = 281.5 Hz), 114.4, 70.0 (q, ${}^{2}J_{CF}$ = 31.4 Hz), 60.1, 41.9, 29.0, 16.4. **^{19}F NMR** (471 MHz, $CDCl_3$) δ -79.87 (s, 3F). **HRMS** (ESI) m/z: $[M+H]^+$ Calcd for $C_{13}H_{19}NO_2F_3^+$ 278.1368; Found 278.1367.

1,1,1-trifluoro-4-(naphthalen-2-ylamino)butan-2-ol (39)

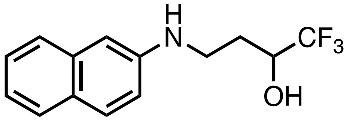

Following General Procedure A, the title compound was obtained as a red solid by preparative TLC using petroleum ether/EtOAc (5:1) as an eluent; R*f* = 0.3 (petroleum ether/EtOAc = 3:1); 18.3 mg, 68% yield, m.p. 120 – 121 ºC. **^{1}H NMR** (500 MHz, $CDCl_3$) δ 7.69 – 7.62 (m, 3H), 7.39 – 7.36 (m, 1H), 7.25 – 7.21 (m, 1H), 6.90 – 6.88 (m, 2H), 4.20 – 4.17 (m, 1H), 3.55 – 3.42 (m, 3H), 2.12 – 1.93 (m, 2H). (Due to the broadening effect and intermolecular hydrogen bonding, one of the proton signals of OH could not be observed). **^{13}C NMR** (201 MHz, $CDCl_3$) δ 145.4, 135.1, 129.3, 128.0, 127.8, 126.6, 126.2, 125.2 (q, $^{1}J_{CF}$ = 281.6 Hz), 122.6, 118.3, 105.5, 69.6 (q, $^{2}J_{CF}$ = 31.4 Hz), 40.7, 29.0. **^{19}F NMR** (471 MHz, $CDCl_3$) δ -79.89 (s, 3F). **HRMS** (ESI) m/z: $[M+H]^+$ Calcd for $C_{14}H_{15}NOF_3^+$ 270.1106; Found 270.1107.

4-((4-(1*H*-pyrrol-1-yl)phenyl)amino)-1,1,1-trifluorobutan-2-ol (40)

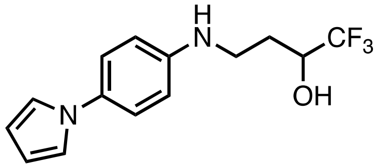

Following General Procedure A, the title compound was obtained as a brown solid by preparative TLC using petroleum ether/EtOAc (5:1) as an eluent; R*f* = 0.3 (petroleum ether/EtOAc = 3:1); 20.4 mg, 72% yield, m.p. 129 – 130 ºC. **^{1}H NMR** (500 MHz, $CDCl_3$) δ 7.25 – 7.17 (m, 2H), 6.97 – 6.96 (m, 2H), 6.73 – 6.69 (m, 2H), 6.33 – 6.27 (m, 2H), 4.19 – 4.13 (m, 1H), 3.47 – 3.34 (m, 2H), 3.26 (brs, 1H), 2.07 – 1.89 (m, 2H). (Due to the broadening effect and intermolecular hydrogen bonding, one of the proton signals of OH could not be observed). **^{13}C NMR** (126 MHz, $CDCl_3$) δ 145.9, 132.9, 125.1 (q, $^{1}J_{CF}$ = 281.5 Hz), 122.6, 119.8, 114.2, 109.7, 69.6 (q, $^{2}J_{CF}$ = 31.5 Hz), 41.0, 29.0. **^{19}F NMR** (471 MHz, $CDCl_3$) δ -79.88 (s, 3F). **HRMS** (ESI) m/z: $[M+H]^+$ Calcd for $C_{14}H_{16}N_2OF_3^+$ 285.1215; Found 285.1213.

1,1,1-trifluoro-4-((4-(pyridin-4-yloxy)phenyl)amino)butan-2-ol (41)

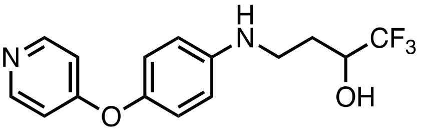

Following General Procedure A, the title compound was obtained as a white solid by preparative TLC using petroleum ether/EtOAc (5:1) as an eluent; R*f*

= 0.3 (petroleum ether/EtOAc = 3:1); 19.3 mg, 62% yield, m.p. 147 – 148 ºC. **^{1}H NMR** (500 MHz, $CDCl_3$) δ 8.31 – 8.30 (m, 2H), 6.92 – 6.89 (m, 2H), 6.77 – 6.76 (m, 2H), 6.68 – 6.65 (m, 2H), 4.19 – 4.15 (m, 1H), 4.03 (brs, 1H), 3.50 – 3.30 (m, 2H), 2.05 – 1.94 (m, 2H). (Due to the broadening effect and intermolecular hydrogen bonding, one of the proton signals of OH could not be observed). **^{13}C NMR** (126 MHz, $CDCl_3$) δ 166.4, 150.6, 146.1, 145.1, 125.4 (q, $^1J_{CF}$ = 281.9 Hz), 122.2, 114.2, 111.8, 69.1 (q, $^2J_{CF}$ = 31.1 Hz), 41.1, 29.2. **^{19}F NMR** (471 MHz, $CDCl_3$) δ -79.83 (s, 3F). **HRMS** (ESI) m/z: $[M+H]^+$ Calcd for $C_{15}H_{16}N_2O_2F_3^+$ 313.1164; Found 313.1161.

1,1,1-trifluoro-4-((6-methoxypyridin-3-yl)amino)butan-2-ol (42)

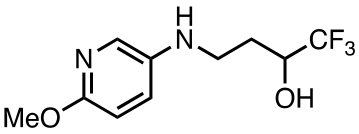

Following General Procedure A, the title compound was obtained as a brown solid by preparative TLC using petroleum ether/EtOAc (5:1) as an eluent; R*f* = 0.3 (petroleum ether/EtOAc = 3:1); 13.5 mg, 54% yield, m.p. 71 – 72 ºC. **^{1}H NMR** (500 MHz, $CDCl_3$) δ 7.57 (s, 1H), 7.03 (d, J = 9.0 Hz, 2H), 6.64 (d, J = 8.7 Hz, 2H), 4.19 – 4.12 (m, 1H), 3.85 (s, 3H), 3.70 (brs, 1H), 3.39 – 3.25 (m, 2H), 2.02 – 1.87 (m, 2H). (Due to the broadening effect and intermolecular hydrogen bonding, one of the proton signals of OH could not be observed). **^{13}C NMR** (126 MHz, $CDCl_3$) δ 158.1, 138.8, 131.2, 127.0, 125.2 (q, $^1J_{CF}$ = 281.8 Hz), 111.0, 69.3 (q, $^2J_{CF}$ = 31.3 Hz), 53.7, 41.9, 29.0. **^{19}F NMR** (471 MHz, $CDCl_3$) δ -79.82 (s, 3F). **HRMS** (ESI) m/z: $[M+H]^+$ Calcd for $C_{10}H_{14}N_2O_2F_3^+$ 251.1007; Found 251.1011.

1,1,1-trifluoro-4-(quinolin-6-ylamino)butan-2-ol (43)

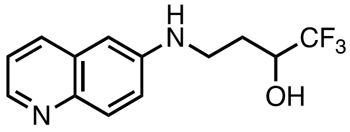

Following General Procedure A, the title compound was obtained as a white solid by preparative TLC using petroleum ether/EtOAc (5:1) as an eluent; R*f* = 0.3 (petroleum ether/EtOAc = 3:1); 16.7 mg, 62% yield, m.p. 168 – 169 ºC. **^{1}H NMR** (500 MHz, CD_3OD_SPE) δ 8.46 – 8.43 (m, 1H), 8.05 (d, J = 8.3 Hz, 1H), 7.73 (d, J = 9.2 Hz, 1H), 7.34 – 7.30 (m, 1H), 7.23 – 7.21 (m, 1H), 6.79 (s, 1H), 4.16 – 4.10 (m, 1H), 3.42 – 3.39 (m, 2H), 2.08 – 1.85 (m, 2H). (Due to the broadening effect and

intermolecular hydrogen bonding, the proton signals of NH and OH could not be observed). **^{13}C NMR** (126 MHz, CD_3OD_SPE) δ 148.7, 145.9, 143.2, 136.0, 132.1, 129.4, 127.1 (q, $^{1}J_{CF}$ = 281.3 Hz), 123.5, 122.5, 103.0, 68.9 (q, $^{2}J_{CF}$ = 30.9 Hz), 40.2, 30.2. **^{19}F NMR** (471 MHz, CD_3OD_SPE) δ -81.29 (s, 3F). **HRMS** (ESI) m/z: $[M+H]^+$ Calcd for $C_{13}H_{14}N_2OF_3^+$ 271.1058; Found 271.1059.

Tert-butyl 6-((4,4,4-trifluoro-3-hydroxybutyl)amino)-1*H*-indole-1-carboxylate (44)

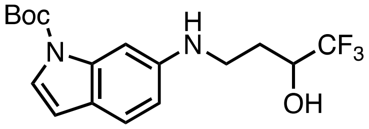

Following General Procedure A, the title compound was obtained as a red oil by preparative TLC using petroleum ether/EtOAc (5:1) as an eluent; R_f = 0.3 (petroleum ether/EtOAc = 3:1); 25.6 mg, 71% yield. **^{1}H NMR** (500 MHz, $CDCl_3$) δ 7.57 (s, 1H), 7.39 – 7.33 (m, 2H), 6.65 – 6.63 (m, 1H), 6.44 (d, *J* = 3.9 Hz, 1H), 4.22 – 4.15 (m, 1H), 3.54 – 3.36 (m, 3H), 2.08 – 1.90 (m, 2H), 1.65 (s, 9H). (Due to the broadening effect and intermolecular hydrogen bonding, one of the proton signals of OH could not be observed). **^{13}C NMR** (201 MHz, $CDCl_3$) δ 150.1, 145.3, 136.9, 125.2 (q, $^{1}J_{CF}$ = 281.6 Hz), 123.9, 123.3, 121.6, 111.9, 107.4, 99.8, 83.5, 69.8 (q, $^{2}J_{CF}$ = 31.3 Hz), 41.7, 29.0, 28.3. **^{19}F NMR** (471 MHz, $CDCl_3$) δ -79.87 (s, 3F). **HRMS** (ESI) m/z: $[M+H]^+$ Calcd for $C_{17}H_{22}N_2O_3F_3^+$ 359.1583; Found 359.1583.

4-(benzo[*b*]thiophen-5-ylamino)-1,1,1-trifluorobutan-2-ol (45)

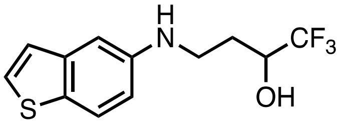

Following General Procedure A, the title compound was obtained as a yellow solid by preparative TLC using petroleum ether/EtOAc (5:1) as an eluent; R_f = 0.3 (petroleum ether/EtOAc = 3:1); 20.9 mg, 76% yield, m.p. 123 – 124 ºC. **^{1}H NMR** (500 MHz, $CDCl_3$) δ 7.66 (d, *J* = 8.6 Hz, 1H), 7.40 (d, *J* = 5.1 Hz, 1H), 7.19 (d, *J* = 5.3 Hz, 1H), 7.09 (s, 1H), 6.78 (d, *J* = 8.6 Hz, 1H), 4.23 – 4.19 (m, 1H), 3.54 – 3.40 (m, 2H), 3.13 (brs, 1H), 2.11 – 1.93 (m, 2H). (Due to the broadening effect and intermolecular hydrogen bonding, one of the proton signals of OH could not be observed). **^{13}C NMR** (126 MHz, $CDCl_3$) δ 145.1, 141.1, 130.7, 127.4, 125.1 (q, $^{1}J_{CF}$ = 281.4 Hz), 123.5, 123.2, 114.7, 106.2, 69.9 (q, $^{2}J_{CF}$ = 31.4 Hz), 41.6, 29.0. **^{19}F NMR** (471 MHz, $CDCl_3$)

δ -79.89 (s, 3F). **HRMS** (ESI) m/z: $[M+H]^+$ Calcd for $C_{12}H_{13}NOF_3S^+$ 276.0670; Found 276.0672.

4-((2,2-difluorobenzo[*d*][1,3]dioxol-5-yl)amino)-1,1,1-trifluorobutan-2-ol (46)

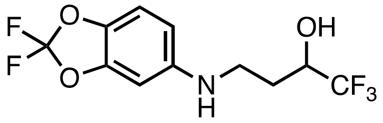

Following General Procedure A, the title compound was obtained as a red solid by preparative TLC using petroleum ether/EtOAc (5:1) as an eluent; R_f = 0.3 (petroleum ether/EtOAc = 3:1); 17.3 mg, 58% yield, m.p. 54 – 55 ºC. **^{1}H NMR** (500 MHz, $CDCl_3$) δ 6.86 (d, J = 8.6 Hz, 1H), 6.42 (s, 1H), 6.30 – 6.27 (m, 1H), 4.16 – 4.12 (m, 1H), 3.40 – 3.28 (m, 2H), 2.05 – 1.87 (m, 2H). (Due to the broadening effect and intermolecular hydrogen bonding, the proton signals of NH and OH could not be observed). **^{13}C NMR** (126 MHz, $CDCl_3$) δ 144.9, 144.8, 136.6, 131.9 (t, $^1J_{CF}$ = 253.7 Hz), 125.0 (q, $^1J_{CF}$ = 281.6 Hz), 110.0, 107.5, 96.1, 69.6 (q, $^2J_{CF}$ = 31.6 Hz), 41.4, 28.9. **^{19}F NMR** (471 MHz, $CDCl_3$) δ -50.39 (s, 2F), -79.96 (s, 3F). **HRMS** (ESI) m/z: $[M+H]^+$ Calcd for $C_{11}H_{11}NO_3F_5^+$ 300.0659; Found 300.0660.

4,4,5,5,6,6,7,7,7-nonafluoro-1-((4-methoxyphenyl)amino)heptan-3-ol (47)

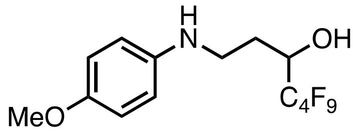

Following General Procedure A, the title compound was obtained as a brown solid by preparative TLC using petroleum ether/EtOAc (8:1) as an eluent; R_f = 0.6 (petroleum ether/EtOAc = 3:1); 14.4 mg, 36% yield, m.p. 69 – 70 ºC. **^{1}H NMR** (500 MHz, $CDCl_3$) δ 6.82 – 6.80 (m, 2H), 6.72 – 6.70 (m, 2H), 4.44 – 4.39 (m, 1H), 3.76 (s, 3H), 3.49 – 3.44 (m, 1H), 3.37 – 3.32 (m, 1H), 2.05 – 1.96 (m, 2H). (Due to the broadening effect and intermolecular hydrogen bonding, the proton signals of NH and OH could not be observed). **^{13}C NMR** (201 MHz, $CDCl_3$) δ 153.8, 141.2, 116.4, 115.0, 70.2 (dd, J = 27.8, 23.0 Hz), 55.9, 43.3, 28.0. **^{19}F NMR** (471 MHz, $CDCl_3$) δ -80.9 (t, J = 10.2 Hz), -120.4 – -121.6 (m), -121.8 – -123.8 (m), -125.0 – -127.4 (m). **HRMS** (ESI) m/z: $[M+H]^+$ Calcd for $C_{14}H_{15}NO_2F_9^+$ 400.0959; Found 400.0958.

4,4,5,5,6,6,7,7,8,8,9,9,9-tridecafluoro-1-((4-methoxyphenyl)amino)nonan-3-ol (48)

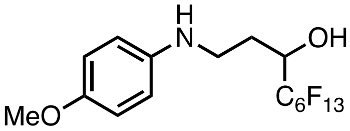

Following General Procedure A, the title compound was obtained as a brown solid by preparative TLC using petroleum ether/EtOAc (8:1) as an eluent; R_f = 0.6 (petroleum ether/EtOAc = 3:1); 12.0 mg, 24% yield, m.p. 85 – 86 ºC. **^{1}H NMR** (500 MHz, $CDCl_3$) δ 6.82 – 6.80 (m, 2H), 6.73 – 6.71 (m, 2H), 4.45 – 4.41 (m, 1H), 3.76 (s, 3H), 3.50 – 3.33 (m, 3H), 2.05 – 1.97 (m, 2H). (Due to the broadening effect and intermolecular hydrogen bonding, one of the proton signals of OH could not be observed). **^{13}C NMR** (201 MHz, $CDCl_3$) δ 153.8, 141.1, 116.5, 115.0, 70.2 (dd, J = 28.0, 22.9 Hz), 55.9, 43.4, 28.0. **^{19}F NMR** (471 MHz, $CDCl_3$) δ -80.8 (t, J = 10.3 Hz), -120.7 – -121.3 (m), -121.6 – -121.7 (m), -122.0 – -122.3 (m), -122.7 – -123.0 (m), -125.3 – -127.0 (m). **HRMS** (ESI) m/z: $[M+H]^+$ Calcd for $C_{16}H_{15}NO_2F_{13}^+$ 500.0895; Found 500.0896.

3-((4-methoxyphenyl)amino)-1-phenylpropan-1-ol (49)

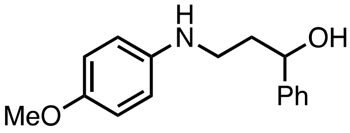

(a) 0.15 mmol scale: Following General Procedure B, the title compound was obtained as a colourless crystalline solid by preparative TLC using petroleum ether/EtOAc (5:1) as an eluent; R_f = 0.3 (petroleum ether/EtOAc = 3:1); 14.9 mg, 58% yield, m.p. 79 – 80 ºC. **^{1}H NMR** (500 MHz, $CDCl_3$) δ 7.37 – 7.25 (m, 5H), 6.80 – 6.77 (m, 2H), 6.64 – 6.61 (m, 2H), 4.92 – 4.89 (m, 1H), 3.74 (s, 3H), 3.29 – 3.20 (m, 3H), 2.05 – 2.01 (m, 2H). (Due to the broadening effect and intermolecular hydrogen bonding, one of the proton signals of OH could not be observed). **^{13}C NMR** (126 MHz, $CDCl_3$) δ 152.8, 144.6, 142.3, 128.6, 127.7, 125.8, 115.3, 115.0, 74.1, 55.9, 43.3, 38.3. **HRMS** (ESI) m/z: $[M+H]^+$ Calcd for $C_{16}H_{20}NO_2^+$ 258.1494; Found 258.1495.

(b) 5.0 mmol scale (Gram-scale synthesis):

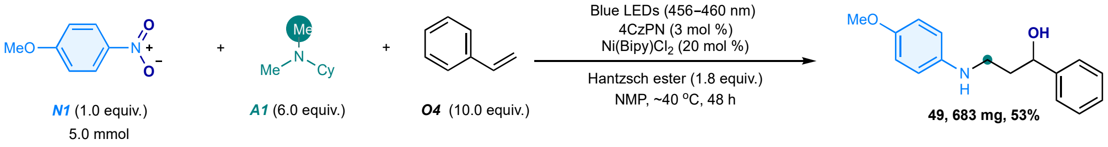

An oven-dried, transparent 150 mL Teflon screw-capped Thick-walled pressure-resistant bottle equipped with a stir bar was sequentially charged with 1-methoxy-4-nitrobenzene (1.0 equiv., 5.0 mmol, 765.1 mg), 4CzPN (3 mol%, 0.15 mmol, 118.3 mg), Ni(Bipy)Cl_2 (20 mol%, 1.0 mmol, 285.8 mg), and Hantzsch ester (**HE,** 1.8 equiv., 9.0 mmol, 2.3 g). Dried *N*-methyl-2-pyrrolidone (NMP, 50.0 mL) was then transferred into the tube via syringe. Subsequently, *N*,*N*-dimethylcyclohexylamine (6.0 equiv., 30.0 mmol, 4.5 mL) and styrene (10.0 equiv., 50.0 mmol, 5.8 mL) were transferred into the tube via syringe. The reaction mixture was vigorously stirred and irradiated using Kessil LEDs (456 nm, 40 W × 2) for 48 h, during which time the proximal temperature was controlled at approximately 40 ºC via cooling with fans. At this point, the reaction mixture was diluted with ethyl acetate (~500 mL) and washed with water (~100 mL × 3). The organic fraction was further dried with anhydrous Na_2SO_4 and concentrated in vacuo with the aid of rotary evaporator. The residue was purified by column chromatography using the silica gel using petroleum ether and ethyl acetate (5:1) as an eluent to afford the product **49** (683 mg, 53%). Refer to Fig. S10 for the experimental setup for the gram-scale synthesis.

***Tert*-butyl (4-(1-hydroxy-3-((4-methoxyphenyl)amino)propyl)phenyl)carbamate (50)**

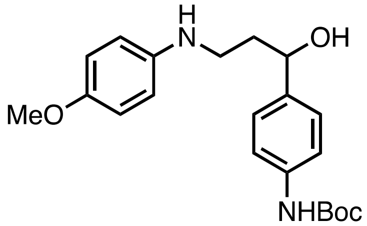

Following General Procedure B, the title compound was obtained as a red solid by preparative TLC using petroleum ether/EtOAc (3:1) as an eluent; R*f* = 0.2 (petroleum ether/EtOAc = 2:1); 17.5 mg, 47% yield, m.p. 98 – 99 ºC. **^{1}H NMR** (500 MHz, $CDCl_3$) δ 7.33 (d, J = 8.1 Hz, 2H), 7.27 (d, J = 7.8 Hz, 2H), 6.78 (d, J = 8.0 Hz, 2H), 6.62 (d, J = 7.5 Hz, 2H), 6.54 (brs, 1H), 4.87 – 4.84 (m, 1H), 3.75 (s, 3H), 3.23 – 3.20 (m, 2H), 2.05 – 1.97 (m, 2H), 1.52 (s, 9H). (Due to the broadening effect and intermolecular hydrogen bonding, the proton signals of NH and OH could not be observed). **^{13}C NMR** (126 MHz, $CDCl_3$) δ 152.9, 152.7, 142.4, 139.2, 137.8, 126.5, 118.8, 115.2, 115.0, 80.7, 73.7, 55.9, 43.2, 38.3, 28.5. **HRMS** (ESI) m/z: $[M+Na]^+$ Calcd for $C_{21}H_{28}N_2O_4Na^+$ 395.1947; Found 395.1948.

Tert-butyl (4-(3-(4-methoxyphenyl)-1,3-oxazinan-6-yl)phenyl)carbamate (50')

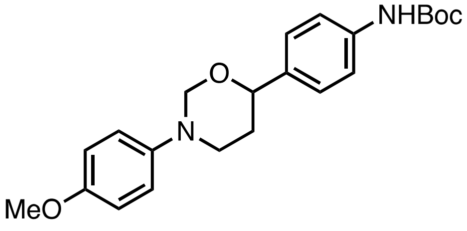

Following General Procedure B, the title compound was obtained as a yellow solid by preparative TLC using petroleum ether/EtOAc (5:1) as an eluent; R*f* = 0.5 (petroleum ether/EtOAc = 3:1); 17.1 mg, 45% yield, m.p. 124 – 125 ºC. **^{1}H NMR** (500 MHz, $CDCl_3$) δ 7.33 – 7.26 (m, 4H), 7.15 – 7.12 (m, 2H), 6.87 – 6.85 (m, 2H), 6.50 (brs, 1H), 5.25 – 5.23 (m, 1H), 4.79 – 4.77 (m, 1H), 4.63 – 4.60 (m, 1H), 3.79 (s, 3H), 3.75– 3.71 (m, 1H), 3.52 – 3.46 (m, 1H), 1.99 – 1.91 (m, 1H), 1.58 – 1.54 (m, 1H), 1.51 (s, 9H). (Due to the broadening effect and intermolecular hydrogen bonding, one of the proton signals of OH could not be observed). **^{13}C NMR** (126 MHz, $CDCl_3$) δ 154.5, 152.8, 143.0, 137.9, 137.0, 126.8, 120.9, 118.6, 114.5, 82.6, 80.6, 79.6, 55.7, 51.3, 30.9, 28.5. **HRMS** (ESI) m/z: $[M+H]^+$ Calcd for $C_{22}H_{29}N_2O_4^+$ 385.2122; Found 385.2121.

3-((4-methoxyphenyl)amino)-1-(4-(trifluoromethyl)phenyl)propan-1-ol (51)

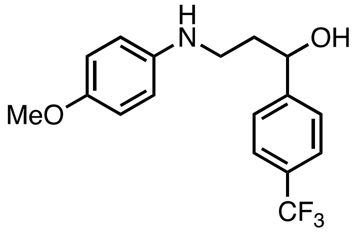

Following General Procedure B, the title compound was obtained as a yellow oil by preparative TLC using petroleum ether/EtOAc (5:1) as an eluent; R*f* = 0.3 (petroleum ether/EtOAc = 3:1); 27.6 mg, 85% yield. **^{1}H NMR** (500 MHz, DMSO-*d*6) δ 7.69 (d, J = 7.9 Hz, 2H), 7.57 (d, J = 7.9 Hz, 2H), 6.70 (d, J = 8.7 Hz, 2H), 6.49 (d, J = 8.5 Hz, 2H), 5.50 – 5.49 (m, 1H), 5.15 (brs, 1H), 4.82 – 4.78 (m, 1H), 3.62 (s, 3H), 3.07 – 2.96 (m, 2H), 1.86 – 1.82 (m, 2H). (Due to the broadening effect and intermolecular hydrogen bonding, one of the proton signals of OH could not be observed). **^{13}C NMR** (201 MHz, DMSO-*d*6) δ 151.2, 150.6, 143.3, 127.3 (q, $^{2}J_{CF}$ = 31.6 Hz), 126.4, 124.4 (q, $^{1}J_{CF}$ = 271.8 Hz). 124.9 (q, $^{3}J_{CF}$ = 3.8 Hz), 114.6, 113.0, 69.8, 55.3, 40.6, 38.7. **^{19}F NMR** (471 MHz, DMSO-*d*6) δ -60.68 (s, 3F). **HRMS** (ESI) m/z: $[M+H]^+$ Calcd for $C_{17}H_{19}NO_2F_3^+$ 326.1368; Found 326.1367.

3-((4-methoxyphenyl)amino)-1-(4-(4,4,5,5-tetramethyl-1,3,2-dioxaborolan-2-yl)phenyl)propan-1-ol (52)

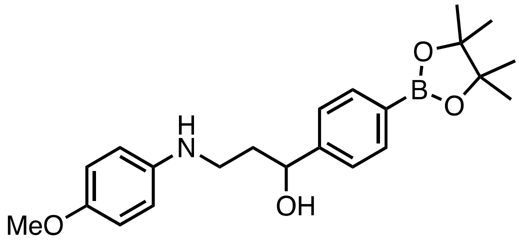

Following General Procedure B, the title compound was obtained as a brown solid by preparative TLC using petroleum ether/EtOAc (3:1) as an eluent; R*f* = 0.3 (petroleum ether/EtOAc = 2:1); 27.4 mg, 58% yield, m.p. 105 – 106 ºC. **^{1}H NMR** (500 MHz, $CDCl_3$) δ 7.80 (d, *J* = 7.5 Hz, 2H), 7.37 (d, *J* = 7.5 Hz, 2H), 6.78 (d, *J* = 8.2 Hz, 2H), 6.63 (d, *J* = 8.3 Hz, 2H), 4.94 – 4.92 (m, 1H), 3.75 (s, 3H), 3.37 (brs, 1H), 3.25 – 3.22 (m, 2H), 2.04 – 2.00 (m, 2H), 1.34 (s, 12H). (Due to the broadening effect and intermolecular hydrogen bonding, one of the proton signals of OH could not be observed). **^{13}C NMR** (126 MHz, $CDCl_3$) δ 152.8, 147.7, 142.3, 135.2, 125.1, 115.3, 114.9, 83.9, 74.1, 55.9, 43.3, 38.3, 25.0. **HRMS** (ESI) m/z: $[M+H]^+$ Calcd for $C_{22}H_{31}NO_4B^+$ 384.2346; Found 384.2348.

3-(4-methoxyphenyl)-6-(4-(4,4,5,5-tetramethyl-1,3,2-dioxaborolan-2-yl)phenyl)-1,3-oxazinane (52')

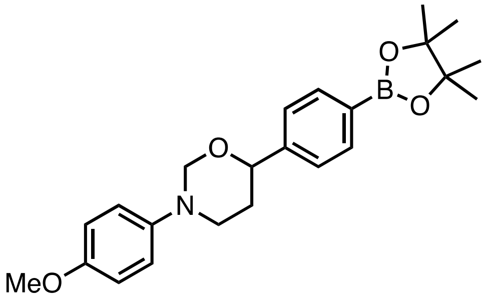

Following General Procedure B, the title compound was obtained as a yellow solid by preparative TLC using petroleum ether/EtOAc (5:1) as an eluent; R*f* = 0.5 (petroleum ether/EtOAc = 3:1); 10.8 mg, 27% yield, m.p. 143 – 144 ºC. **^{1}H NMR** (500 MHz, $CDCl_3$) δ 7.79 (d, *J* = 7.6 Hz, 2H), 7.36 (d, *J* = 7.6 Hz, 2H), 7.14 (d, *J* = 8.4 Hz, 2H), 6.86 (d, *J* = 8.4 Hz, 2H), 5.27 (d, *J* = 10.7 Hz, 1H), 4.79 (d, *J* = 10.7 Hz, 1H), 4.68 (d, *J* = 11.3 Hz, 1H), 3.79 (s, 3H), 3.75 – 3.71 (m, 1H), 3.53 – 3.48 (m, 1H), 1.98 – 1.90 (m, 1H), 1.62 – 1.59 (m, 1H), 1.33 (s, 12H). (Due to the broadening effect and intermolecular hydrogen bonding, one of the proton signals of OH could not be observed). **^{13}C NMR** (126 MHz, $CDCl_3$) δ 154.4, 145.3, 142.8, 135.0, 125.1, 120.9, 114.4, 83.8, 82.5, 79.8,

55.6, 51.2, 31.0, 24.9. **HRMS** (ESI) m/z: $[M+H]^+$ Calcd for $C_{23}H_{31}NO_4B^+$ 396.2341; Found 396.2343.

3-((4-methoxyphenyl)amino)-1-(2-vinylphenyl)propan-1-ol (53)

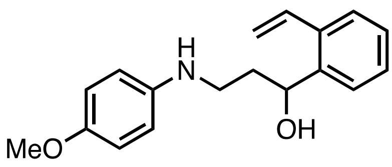

Following General Procedure B, the title compound was obtained as a yellow oil by preparative TLC using petroleum ether/EtOAc (5:1) as an eluent; R_f = 0.3 (petroleum ether/EtOAc = 3:1); 14.4 mg, 51% yield. **^{1}H NMR** (500 MHz, $CDCl_3$) δ 7.54 (d, J = 7.6 Hz, 1H), 7.45 (d, J = 7.6 Hz, 1H), 7.32 – 7.25 (m, 2H), 7.01 – 6.95 (m, 1H), 6.78 (d, J = 8.1 Hz, 2H), 6.63 (d, J = 8.3 Hz, 2H), 5.60 (d, J = 17.2 Hz, 1H), 5.29 (d, J = 10.9 Hz, 1H), 5.23 – 5.21 (m, 1H), 3.75 (s, 3H), 3.35 – 3.26 (m, 3H), 2.04 – 1.93 (m, 2H). (Due to the broadening effect and intermolecular hydrogen bonding, one of the proton signals of OH could not be observed). **^{13}C NMR** (126 MHz, $CDCl_3$) δ 152.8, 142.3, 141.6, 135.5, 134.1, 128.2, 127.6, 126.4, 125.5, 116.8, 115.2, 114.9, 70.6, 55.9, 43.4, 37.3. **HRMS** (ESI) m/z: $[M+H]^+$ Calcd for $C_{18}H_{22}NO_2^+$ 284.1651; Found 284.1649.

3-((4-methoxyphenyl)amino)-1-(perfluorophenyl)propan-1-ol (54)

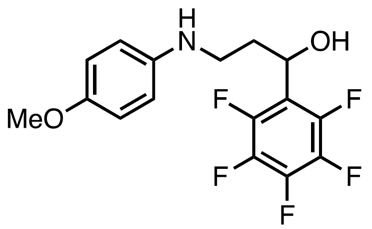

Following General Procedure B, the title compound was obtained as a light green solid by preparative TLC using petroleum ether/EtOAc (3:1) as an eluent; R_f = 0.2 (petroleum ether/EtOAc = 2:1); 28.8 mg, 83% yield, m.p. 129 – 130 ºC. **^{1}H NMR** (500 MHz, DMSO-d_6) δ 7.69 (d, J = 8.3 Hz, 2H), 7.49 (d, J = 8.3 Hz, 2H), 5.14 (brs, 1H), 5.08 – 5.05 (m, 1H), 3.62 (s, 3H), 3.35 – 3.33 (m, 1H), 3.03 – 3.00 (m, 2H), 2.19 – 2.12 (m, 1H), 1.99 – 1.93 (m, 1H). **^{13}C NMR** (201 MHz, DMSO-d_6) δ 150.7, 145.2 – 144.4 (m), 143.8 – 143.6 (m), 143.1 (d, J = 5.5 Hz), 140.3 – 139.9 (m), 139.0 – 138.6 (m), 137.7 – 137.3 (m), 136.5 – 135.9 (m), 118.6 – 118.2 (m), 114.6, 113.1 (d, J = 5.4 Hz), 62.3 (d, J = 20.7 Hz), 55.3, 40.5 (d, J = 8.8 Hz), 35.2 (d, J = 11.8 Hz). **^{19}F NMR** (471 MHz, DMSO-d_6) δ -143.4 (dd, J = 24.2, 7.7 Hz, 2F), -157.0 (td, J

= 22.1, 4.9 Hz, 1F), -163.1 (td, *J* = 23.0, 7.4 Hz, 2F). **HRMS** (ESI) m/z: $[M+H]^+$ Calcd for $C_{16}H_{15}NO_2F_5^+$ 348.1023; Found 348.1025.

3-((4-methoxyphenyl)amino)-1-(naphthalen-1-yl)propan-1-ol (55)

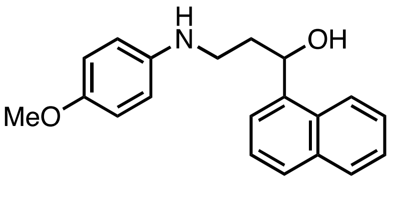

Following General Procedure B, the title compound was obtained as a yellow oil by preparative TLC using petroleum ether/EtOAc (5:1) as an eluent; R_f = 0.3 (petroleum ether/EtOAc = 3:1); 21.4 mg, 70% yield. **^{1}H NMR** (500 MHz, $CDCl_3$) δ 8.02 – 8.00 (m, 1H), 7.89 – 7.87 (m, 1H), 7.79 (d, *J* = 8.3 Hz, 1H), 7.70 (d, *J* = 7.1 Hz, 1H), 7.51 – 7.47 (m, 3H), 6.81 – 6.79 (m, 2H), 6.66 – 6.64 (m, 2H), 5.70 – 5.68 (m, 1H), 3.76 (s, 3H), 3.46 (brs, 1H), 3.36 – 3.33 (m, 2H), 2.29 – 2.09 (m, 2H). (Due to the broadening effect and intermolecular hydrogen bonding, one of the proton signals of OH could not be observed). **^{13}C NMR** (126 MHz, $CDCl_3$) δ 152.8, 142.4, 140.2, 133.9, 130.2, 129.1, 128.1, 126.2, 125.7, 125.6, 123.1, 122.9, 115.3, 115.0, 70.9, 55.9, 43.5, 37.4. **HRMS** (ESI) m/z: $[M+H]^+$ Calcd for $C_{20}H_{22}NO_2^+$ 308.1651; Found 308.1652.

3-((4-methoxyphenyl)amino)-1-(naphthalen-2-yl)propan-1-ol (56)

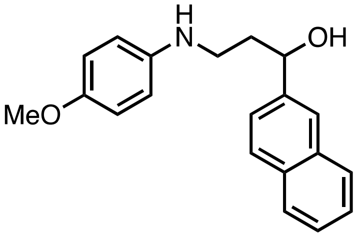

Following General Procedure B, the title compound was obtained as a yellow solid by preparative TLC using petroleum ether/EtOAc (5:1) as an eluent; R_f = 0.3 (petroleum ether/EtOAc = 3:1); 17.8 mg, 58% yield, m.p. 111 – 112 ºC. **^{1}H NMR** (500 MHz, $CDCl_3$) δ 7.85 – 7.82 (m, 4H), 7.49 – 7.47 (m, 3H), 6.79 (d, *J* = 8.2 Hz, 2H), 6.63 (d, *J* = 8.3 Hz, 2H), 5.08 – 5.06 (m, 1H), 3.75 (s, 3H), 3.42 (brs, 1H), 3.29 – 3.27 (m, 2H), 2.13 – 2.09 (m, 2H). (Due to the broadening effect and intermolecular hydrogen bonding, one of the proton signals of OH could not be observed). **^{13}C NMR** (201 MHz, $CDCl_3$) δ 152.8, 142.3, 142.0, 133.4, 133.1, 128.4, 128.1, 127.8, 126.3, 126.0, 124.5, 124.1, 115.3, 115.0, 74.2, 55.9, 43.3, 38.2. **HRMS** (ESI) m/z: $[M+H]^+$ Calcd for $C_{20}H_{22}NO_2^+$ 308.1651; Found 308.1651.

3-(4-methoxyphenyl)-6-(naphthalen-2-yl)-1,3-oxazinane (56')

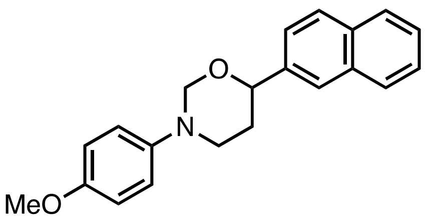

Following General Procedure B, the title compound was obtained as a yellow solid by preparative TLC using petroleum ether/EtOAc (5:1) as an eluent; R*f* = 0.5 (petroleum ether/EtOAc = 3:1); 7.3 mg, 23% yield, m.p. 99 – 100 ºC. **^{1}H NMR** (500 MHz, $CDCl_3$) *δ* 7.84 – 7.82 (m, 4H), 7.49 – 7.44 (m, 3H), 7.19 (d, *J* = 9.1 Hz, 2H), 6.89 (d, *J* = 9.2 Hz, 2H), 5.34 – 5.32 (m, 1H), 4.88 – 4.84 (m, 2H), 3.81 – 3.76 (m, 4H), 3.61 – 3.55 (m, 1H), 2.11 – 2.03 (m, 1H), 1.71 – 1.68 (m, 1H). (Due to the broadening effect and intermolecular hydrogen bonding, one of the proton signals of OH could not be observed). **^{13}C NMR** (126 MHz, $CDCl_3$) *δ* 154.5, 143.0, 139.8, 133.4, 133.1, 128.3, 128.1, 127.8, 126.2, 125.9, 124.7, 124.3, 121.0, 114.6, 82.7, 80.0, 55.7, 51.4, 31.0. **HRMS** (ESI) m/z: $[M+H]^+$ Calcd for $C_{21}H_{22}NO_2^+$ 320.1651; Found 320.1649.

3-((4-methoxyphenyl)amino)-1-(pyridin-2-yl)propan-1-ol (57)

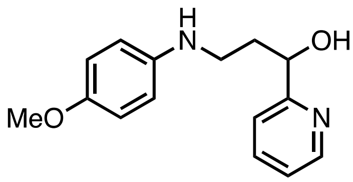

Following General Procedure B, the title compound was obtained as a white solid by preparative TLC using petroleum ether/EtOAc (5:1) as an eluent; R*f* = 0.3 (petroleum ether/EtOAc = 3:1); 13.9 mg, 54% yield, m.p. 65 – 66 ºC. **^{1}H NMR** (500 MHz, $CDCl_3$) *δ* 8.54 (d, *J* = 4.8 Hz, 1H), 7.71 – 7.67 (m, 1H), 7.30 (d, *J* = 7.9 Hz, 1H), 7.22 – 7.19 (m, 1H), 6.79 – 6.76 (m, 2H), 6.62 – 6.59 (m, 2H), 4.94 – 4.92 (m, 1H), 3.74 (s, 3H), 3.37 – 3.25 (m, 2H), 2.20 – 1.91 (m, 2H). (Due to the broadening effect and intermolecular hydrogen bonding, one of the proton signals of OH could not be observed). **^{13}C NMR** (201 MHz, $CDCl_3$) *δ* 161.9, 152.4, 148.4, 142.6, 136.9, 122.5, 120.3, 115.0, 114.7, 72.3, 55.9, 42.4, 37.7. **HRMS** (ESI) m/z: $[M+H]^+$ Calcd for $C_{15}H_{19}N_2O_2^+$ 259.1447; Found 259.1448.

3-(4-methoxyphenyl)-6-(naphthalen-2-yl)-1,3-oxazinane (57')

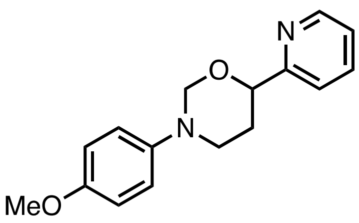

Following General Procedure B, the title compound was obtained as a yellow solid by preparative TLC using petroleum ether/EtOAc (5:1) as an eluent; R*f* = 0.5 (petroleum ether/EtOAc = 3:1); 9.6 mg, 36% yield, m.p. 99 – 100 ºC. **^{1}H NMR** (500 MHz, $CDCl_3$) δ 8.53 – 8.52 (m, 1H), 7.71 – 7.68 (m, 1H), 7.49 (d, J = 8.0 Hz, 1H), 7.21 – 7.16 (m, 1H), 7.13 (d, J = 9.3 Hz, 2H), 6.85 (d, J = 9.3 Hz, 2H), 5.31 – 5.28 (m, 1H), 4.84 – 4.79 (m, 2H), 3.78 (s, 3H), 3.76 – 3.71 (m, 1H), 3.57 – 3.51 (m, 1H), 1.91 – 1.88 (m, 2H). (Due to the broadening effect and intermolecular hydrogen bonding, one of the proton signals of OH could not be observed). **^{13}C NMR** (126 MHz, $CDCl_3$) δ 161.4, 154.5, 148.9, 142.9, 137.0, 122.5, 120.8, 120.1, 114.6, 82.5, 80.4, 55.7, 51.0, 29.7. **HRMS** (ESI) m/z: $[M+H]^+$ Calcd for $C_{16}H_{19}N_2O_2^+$ 271.1447; Found 271.1447.

3-((4-methoxyphenyl)amino)-1-(thiophen-2-yl)propan-1-ol (58)

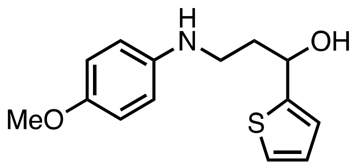

Following General Procedure B, the title compound was obtained as a brown solid by preparative TLC using petroleum ether/EtOAc (5:1) as an eluent; R*f* = 0.3 (petroleum ether/EtOAc = 3:1); 15.5 mg, 59% yield, m.p. 68 – 69 ºC. **^{1}H NMR** (500 MHz, $CDCl_3$) δ 7.25 – 7.24 (m, 1H), 6.98 – 6.97 (m, 2H), 6.79 – 6.77 (m, 2H), 6.65 – 6.62 (m, 2H), 5.17 – 5.15 (m, 1H), 3.74 (s, 3H), 3.33 – 3.24 (m, 3H), 2.15 – 2.11 (m, 2H). (Due to the broadening effect and intermolecular hydrogen bonding, one of the proton signals of OH could not be observed). **^{13}C NMR** (126 MHz, $CDCl_3$) δ 152.9, 148.7, 142.2, 126.9, 124.6, 115.3, 115.0, 70.1, 55.9, 43.2, 38.6. **HRMS** (ESI) m/z: $[M+H]^+$ Calcd for $C_{14}H_{18}NO_2S^+$ 264.1058; Found 264.1059.

3-((4-methoxyphenyl)amino)-1-(thiophen-3-yl)propan-1-ol (59)

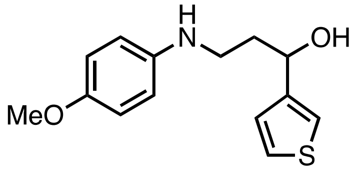

Following General Procedure B, the title compound was obtained as a brown solid by preparative TLC using petroleum ether/EtOAc (5:1) as an eluent; R*f* = 0.3 (petroleum ether/EtOAc = 3:1); 14.5 mg, 55% yield, m.p. 82 – 83 ºC. **^{1}H NMR** (500

MHz, $CDCl_3$) δ 7.32 – 7.30 (m, 1H), 7.21 (s, 1H), 7.08 – 7.07 (m, 1H), 6.78 (d, J = 8.3 Hz, 2H), 6.62 (d, J = 8.3 Hz, 2H), 5.01 – 4.99 (m, 1H), 3.75 (s, 3H), 3.40 (brs, 1H), 3.27 – 3.24 (m, 2H), 2.09 – 2.03 (m, 2H). (Due to the broadening effect and intermolecular hydrogen bonding, one of the proton signals of OH could not be observed). **^{13}C NMR** (126 MHz, $CDCl_3$) δ 152.8, 146.1, 142.3, 126.4, 125.7, 120.8, 115.2, 115.0, 70.4, 55.9, 43.2, 37.6. **HRMS** (ESI) m/z: $[M+H]^+$ Calcd for $C_{14}H_{18}NO_2S^+$ 264.1058; Found 264.1061.

3-((4-methoxyphenyl)amino)-1-(4-methylthiazol-5-yl)propan-1-ol (60)

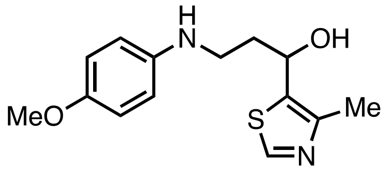

Following General Procedure B, the title compound was obtained as a yellow solid by preparative TLC using petroleum ether/EtOAc (5:1) as an eluent; R_f = 0.3 (petroleum ether/EtOAc = 3:1); 18.7 mg, 71% yield, m.p. 93 – 94 ºC. **^{1}H NMR** (500 MHz, $CDCl_3$) δ 8.59 (s, 1H), 6.79 (d, J = 8.9 Hz, 2H), 6.64 (d, J = 9.0 Hz, 2H), 5.22 – 5.19 (m, 1H), 3.74 (s, 3H), 3.60 (brs, 1H), 3.33 – 3.23 (m, 2H), 2.37 (s, 3H), 2.11 – 1.98 (m, 2H). (Due to the broadening effect and intermolecular hydrogen bonding, one of the proton signals of OH could not be observed). **^{13}C NMR** (201 MHz, $CDCl_3$) δ 153.0, 150.9, 148.0, 142.0, 136.3, 115.5, 115.0, 67.5, 55.9, 43.4, 38.5, 15.3. **HRMS** (ESI) m/z: $[M+H]^+$ Calcd for $C_{14}H_{19}N_2O_2S^+$ 279.1167; Found 279.1168.

(*E*)-5-((4-methoxyphenyl)amino)-1-phenylpent-1-en-3-ol (61)

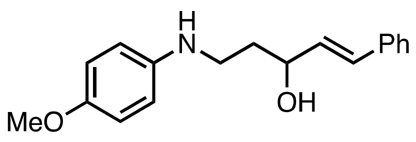

Following General Procedure B, the title compound was obtained as a yellow solid by preparative TLC using petroleum ether/EtOAc (5:1) as an eluent; R_f = 0.3 (petroleum ether/EtOAc = 3:1); 18.2 mg, 64% yield, m.p. 96 – 97 ºC. **^{1}H NMR** (500 MHz, $CDCl_3$) δ 7.39 – 7.23 (m, 5H), 6.79 (d, J = 8.1 Hz, 2H), 6.65 – 6.60 (m, 3H), 6.25 (dd, J = 15.9, 6.2 Hz, 1H), 4.53 – 4.49 (m, 1H), 3.75 (s, 3H), 3.34 – 3.18 (m, 3H), 1.98 – 1.86 (m, 2H). (Due to the broadening effect and intermolecular hydrogen bonding, one of the proton signals of OH could not be observed). **^{13}C NMR** (126 MHz, $CDCl_3$) δ 152.7, 142.3, 136.7, 132.1, 130.3, 128.7, 127.8, 126.6, 115.2, 115.0, 72.4,

55.9, 43.0, 36.4. **HRMS** (ESI) m/z: $[M+H]^+$ Calcd for $C_{18}H_{22}NO_2^+$ 284.1651; Found 284.1646.

Methyl 2-hydroxy-4-((4-methoxyphenyl)amino)butanoate (62)

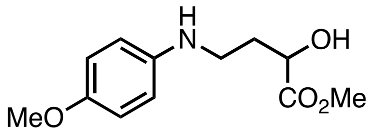

Following General Procedure B, the title compound was obtained as a yellow solid by preparative TLC using petroleum ether/EtOAc (5:1) as an eluent; R_f = 0.3 (petroleum ether/EtOAc = 3:1); 15.3 mg, 64% yield, m.p. 171 – 172 ºC. **^{1}H NMR** (800 MHz, $CDCl_3$) δ 6.78 (d, J = 8.8 Hz, 2H), 6.60 (d, J = 8.8 Hz, 2H), 4.35 – 4.34 (m, 1H), 3.74 (s, 6H), 3.41 (brs, 1H), 3.29 (t, J = 6.5 Hz, 2H), 2.13 – 2.10 (m, 1H), 1.98 – 1.94 (m, 1H). (Due to the broadening effect and intermolecular hydrogen bonding, one of the proton signals of OH could not be observed). **^{13}C NMR** (201 MHz, $CDCl_3$) δ 175.6, 152.5, 142.2, 115.0, 114.5, 69.5, 55.9, 52.7, 41.5, 33.5. **HRMS** (ESI) m/z: $[M+H]^+$ Calcd for $C_{12}H_{18}NO_4^+$ 240.1236; Found 240.1238.

Methyl 2-hydroxy-4-(p-tolylamino)butanoate (63)

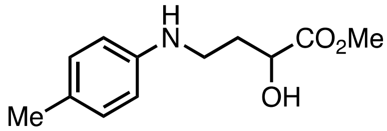

Following General Procedure B, the title compound was obtained as a yellow solid by preparative TLC using petroleum ether/EtOAc (3:1) as an eluent; R_f = 0.2 (petroleum ether/EtOAc = 2:1); 18.1 mg, 81% yield, m.p. 62 – 63 ºC. **^{1}H NMR** (500 MHz, $CDCl_3$) δ 6.99 (d, J = 7.9 Hz, 2H), 6.55 (d, J = 7.9 Hz, 2H), 4.35 – 4.33 (m, 1H), 3.74 (s, 3H), 3.45 (brs, 1H), 3.33 – 3.30 (m, 2H), 2.24 (s, 3H), 2.16– 2.09 (m, 1H), 2.00– 1.93 (m, 1H). (Due to the broadening effect and intermolecular hydrogen bonding, one of the proton signals of OH could not be observed). **^{13}C NMR** (126 MHz, $CDCl_3$) δ 175.6, 145.8, 129.9, 127.0, 113.3, 69.3, 52.7, 40.7, 33.4, 20.5. **HRMS** (ESI) m/z: $[M+H]^+$ Calcd for $C_{12}H_{18}NO_3^+$ 224.1287; Found 224.1288.

Ethyl 2-hydroxy-4-((4-methoxyphenyl)amino)butanoate (64)

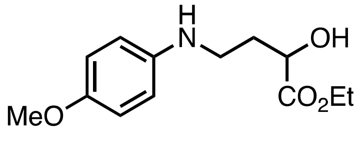

Following General Procedure B, the title compound was obtained as a yellow solid by preparative TLC using petroleum ether/EtOAc (3:1) as an eluent; R$_f$ = 0.2 (petroleum ether/EtOAc = 2:1); 17.1 mg, 68% yield, m.p. 177 – 178 ºC. **^{1}H NMR** (500 MHz, $CDCl_3$) δ 6.78 (d, J = 8.7 Hz, 2H), 6.60 (d, J = 8.4 Hz, 2H), 4.33 – 4.31 (m, 1H), 4.26 – 4.17 (m, 2H), 3.75 (s, 3H), 3.31 – 3.28 (m, 2H), 3.15 (brs, 1H), 2.14 – 1.94 (m, 2H), 1.25 (t, J = 7.1 Hz, 3H). (Due to the broadening effect and intermolecular hydrogen bonding, one of the proton signals of OH could not be observed). **^{13}C NMR** (126 MHz, $CDCl_3$) δ 175.2, 152.4, 142.2, 115.0, 114.5, 69.5, 62.0, 55.9, 41.5, 33.5, 14.3. **HRMS** (ESI) m/z: $[M+H]^+$ Calcd for $C_{13}H_{20}NO_4^+$ 254.1392; Found 254.1389.

Phenethyl 2-hydroxy-4-((4-methoxyphenyl)amino)butanoate (65)

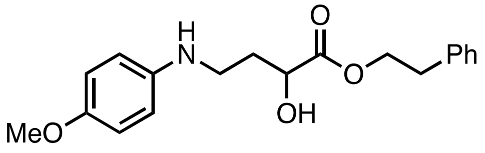

Following General Procedure B, the title compound was obtained as a yellow solid by preparative TLC using petroleum ether/EtOAc (3:1) as an eluent; R$_f$ = 0.2 (petroleum ether/EtOAc = 2:1); 16.8 mg, 51% yield, m.p. 62 – 63 ºC. **^{1}H NMR** (500 MHz, $CDCl_3$) δ 7.31 – 7.17 (m, 5H), 6.78 (d, J = 8.2 Hz, 2H), 6.56 (d, J = 8.3 Hz, 2H), 4.36 – 4.29 (m, 3H), 3.74 (s, 3H), 3.22 – 3.19 (m, 2H), 2.92 – 2.89 (m, 2H), 2.07 – 1.88 (m, 2H). (Due to the broadening effect and intermolecular hydrogen bonding, the proton signals of NH and OH could not be observed). **^{13}C NMR** (126 MHz, $CDCl_3$) δ 175.1, 152.4, 142.2, 137.4, 129.0, 128.7, 126.9, 115.0, 114.5, 69.4, 66.2, 55.9, 41.2, 35.0, 33.4. **HRMS** (ESI) m/z: $[M+H]^+$ Calcd for $C_{19}H_{24}NO_4^+$ 330.1705; Found 330.1704.

Benzyl 2-hydroxy-4-((4-methoxyphenyl)amino)butanoate (66)

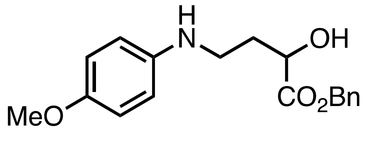

Following General Procedure B, the title compound was obtained as a yellow solid by preparative TLC using petroleum ether/EtOAc (3:1) as an eluent; R$_f$ = 0.2 (petroleum ether/EtOAc = 2:1); 24.2 mg, 77% yield, m.p. 71 – 72 ºC. **^{1}H NMR** (500 MHz, $CDCl_3$) δ 7.38 – 7.29 (m, 5H), 6.78 – 6.75 (m, 2H), 6.57 – 6.55 (m, 2H), 5.20 –

5.12 (m, 2H), 4.39 – 4.36 (m, 1H), 3.74 (s, 3H), 3.40 (brs, 1H), 3.28 – 3.25 (m, 2H), 2.15 – 1.98 (m, 2H). (Due to the broadening effect and intermolecular hydrogen bonding, one of the proton signals of OH could not be observed). **^{13}C NMR** (126 MHz, $CDCl_3$) δ 175.0, 152.5, 142.2, 135.2, 128.8, 128.8, 128.6, 115.0, 114.5, 69.5, 67.6, 55.9, 41.4, 33.4. **HRMS** (ESI) m/z: $[M+H]^+$ Calcd for $C_{18}H_{22}NO_4^+$ 316.1549; Found 316.1547.

***Tert*-butyl 2-hydroxy-4-((4-methoxyphenyl)amino)butanoate (67)**

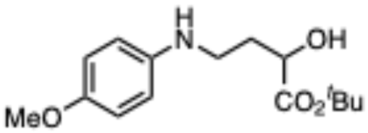

Following General Procedure B, the title compound was obtained as a yellow oil by preparative TLC using petroleum ether/EtOAc (3:1) as an eluent; R_f = 0.2 (petroleum ether/EtOAc = 2:1); 25.0 mg, 89% yield. **^{1}H NMR** (500 MHz, $CDCl_3$) δ 6.78 (d, J = 8.1 Hz, 2H), 6.60 (d, J = 7.6 Hz, 2H), 4.20 – 4.18 (m, 1H), 3.74 (s, 3H), 3.28 – 3.25 (m, 3H), 2.11 – 1.88 (m, 2H), 1.48 (s, 9H). (Due to the broadening effect and intermolecular hydrogen bonding, one of the proton signals of OH could not be observed). **^{13}C NMR** (201 MHz, $CDCl_3$) δ 174.3, 152.3, 142.5, 115.0, 114.4, 82.8, 69.7, 55.9, 41.6, 33.8, 28.1. **HRMS** (ESI) m/z: $[M+H]^+$ Calcd for $C_{15}H_{24}NO_4^+$ 282.1705; Found 282.1703.

***Tert*-butyl 2-hydroxy-4-((2-vinylphenyl)amino)butanoate (68)**

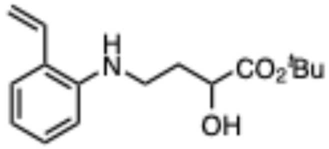

Following General Procedure B, the title compound was obtained as a yellow oil by preparative TLC using petroleum ether/EtOAc (3:1) as an eluent; R_f = 0.2 (petroleum ether/EtOAc = 2:1); 17.4 mg, 63% yield. **^{1}H NMR** (500 MHz, $CDCl_3$) δ 7.26 (d, J = 6.9 Hz, 1H), 7.18 (t, J = 7.8 Hz, 1H), 6.77 – 6.70 (m, 2H), 6.66 (d, J = 8.1 Hz, 1H), 5.60 (d, J = 17.1 Hz, 1H), 5.30 (d, J = 11.0 Hz, 1H), 4.21 – 4.19 (m, 1H), 3.38 – 3.31 (m, 2H), 3.08 (brs, 1H), 2.16 – 2.11 (m, 1H), 1.99 – 1.92 (m, 1H), 1.48 (s, 9H). (Due to the broadening effect and intermolecular hydrogen bonding, one of the proton signals of OH could not be observed). **^{13}C NMR** (126 MHz, $CDCl_3$) δ 174.3, 145.3,

133.0, 129.1, 127.5, 124.4, 117.3, 116.3, 110.6, 83.0, 69.8, 40.8, 33.6, 28.1. **HRMS** (ESI) m/z: $[M+H]^+$ Calcd for $C_{16}H_{24}NO_3^+$ 278.1756; Found 278.1753.

2-hydroxy-4-((4-methoxyphenyl)amino)-*N*-phenylbutanamide (69)

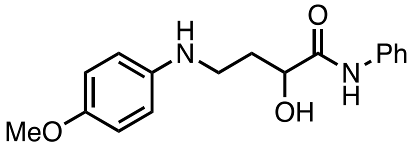

Following General Procedure B, the title compound was obtained as a white solid by preparative TLC using petroleum ether/EtOAc (3:1) as an eluent; R_f = 0.2 (petroleum ether/EtOAc = 2:1); 26.4 mg, 88% yield, m.p. 131 – 132 ºC. **^{1}H NMR** (500 MHz, $CDCl_3$) δ 8.76 (s, 1H), 7.58 (d, J = 7.8 Hz, 2H), 7.33 (t, J = 7.6 Hz, 2H), 7.12 (t, J = 7.5 Hz, 1H), 6.81 (d, J = 8.6 Hz, 2H), 6.73 (d, J = 7.9 Hz, 2H), 4.47 – 4.44 (m, 2H), 3.75 (s, 3H), 3.44 – 3.32 (m, 2H), 2.29 – 2.05 (m, 2H). (Due to the broadening effect and intermolecular hydrogen bonding, one of the proton signals of OH could not be observed). **^{13}C NMR** (126 MHz, $CDCl_3$) δ 171.7, 154.0, 141.1, 137.4, 129.2, 124.6, 119.8, 116.9, 115.0, 73.7, 55.8, 44.8, 32.6. **HRMS** (ESI) m/z: $[M+H]^+$ Calcd for $C_{17}H_{21}N_2O_3^+$ 301.1552; Found 301.1551.

3-((4-methoxyphenyl)amino)-1,1-diphenylpropan-1-ol (70)

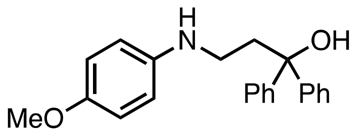

Following General Procedure B, the title compound was obtained as a white solid by preparative TLC using petroleum ether/EtOAc (5:1) as an eluent; R_f = 0.3 (petroleum ether/EtOAc = 3:1); 11.7 mg, 35% yield, m.p. 154 – 155 ºC. **^{1}H NMR** (500 MHz, $CDCl_3$) δ 7.46 (d, J = 7.5 Hz, 4H), 7.32 (t, J = 7.7 Hz, 4H), 7.25 – 7.21 (m, 2H), 6.74 (d, J = 8.2 Hz, 2H), 6.56 (d, J = 8.3 Hz, 2H), 4.12 (brs, 1H), 3.73 (s, 3H), 3.20 – 3.18 (m, 2H), 2.60 – 2.58 (m, 2H). (Due to the broadening effect and intermolecular hydrogen bonding, one of the proton signals of OH could not be observed). **^{13}C NMR** (126 MHz, $CDCl_3$) δ 153.5, 147.2, 141.7, 128.4, 127.0, 126.1, 116.4, 114.9, 78.9, 55.8, 42.9, 40.1. **HRMS** (ESI) m/z: $[M+H]^+$ Calcd for $C_{22}H_{24}NO_2^+$ 334.1807; Found 334.1808.

1,1,1-trifluoro-4-((4-methoxyphenyl)amino)-2-phenylbutan-2-ol (71)

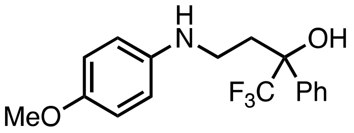

Following General Procedure B, the title compound was obtained as a colourless crystalline solid by preparative TLC using petroleum ether/EtOAc (5:1) as an eluent; R*f* = 0.3 (petroleum ether/EtOAc = 3:1); 22.1 mg, 68% yield, m.p. 111 – 112 ºC. **^{1}H NMR** (500 MHz, $CDCl_3$) *δ* 7.65 (d, *J* = 7.3 Hz, 2H), 7.45 – 7.38 (m, 3H), 6.76 (d, *J* = 8.3 Hz, 2H), 6.61 (d, *J* = 8.3 Hz, 2H), 3.74 (s, 3H), 3.34 – 3.32 (m, 1H), 3.12 – 3.08 (m, 1H), 2.48 – 2.37 (m, 2H). (Due to the broadening effect and intermolecular hydrogen bonding, the proton signals of NH and OH could not be observed). **^{13}C NMR** (201 MHz, $CDCl_3$) *δ* 154.5, 140.6, 138.1, 128.6, 128.5, 126.9, 125.4 (q, $^{1}J_{CF}$ = 285.2 Hz), 117.6, 114.9, 78.2 (q, $^{2}J_{CF}$ = 28.3 Hz), 55.8, 43.1, 33.2. **^{19}F NMR** (471 MHz, $CDCl_3$) *δ* -80.05 (s, 3F). **HRMS** (ESI) m/z: $[M+H]^+$ Calcd for $C_{17}H_{19}NO_2F_3^+$ 326.1368; Found 326.1367.

1,1,1-trifluoro-2-(4-methoxyphenyl)-4-((4-methoxyphenyl)amino)butan-2-ol (72)

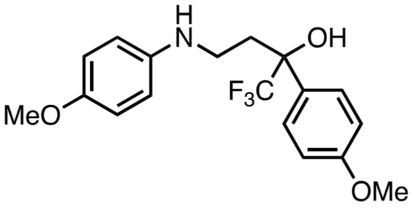

Following General Procedure B, the title compound was obtained as a yellow solid by preparative TLC using petroleum ether/EtOAc (5:1) as an eluent; R*f* = 0.3 (petroleum ether/EtOAc = 3:1); 22.0 mg, 62% yield, m.p. 77 – 78 ºC. **^{1}H NMR** (500 MHz, $CDCl_3$) *δ* 7.55 (d, *J* = 8.3 Hz, 2H), 6.95 (d, *J* = 8.4 Hz, 2H), 6.76 (d, *J* = 8.1 Hz, 2H), 6.61 (d, *J* = 8.2 Hz, 2H), 3.84 (s, 3H), 3.74 (s, 3H), 3.34 – 3.29 (m, 1H), 3.14 – 3.08 (m, 1H), 2.44 – 2.33 (m, 2H). (Due to the broadening effect and intermolecular hydrogen bonding, the proton signals of NH and OH could not be observed). **^{13}C NMR** (126 MHz, $CDCl_3$) *δ* 159.8, 154.4, 140.7, 129.9, 128.2, 125.5 (q, $^{1}J_{CF}$ = 285.1 Hz), 117.6, 114.9, 113.9, 78.0 (q, $^{2}J_{CF}$ = 28.5 Hz), 55.8, 55.4, 43.0, 33.1. **^{19}F NMR** (471 MHz, $CDCl_3$) *δ* -80.48 (s, 3F). **HRMS** (ESI) m/z: $[M+H]^+$ Calcd for $C_{18}H_{21}NO_3F_3^+$ 356.1474; Found 356.1471.

2-(3,5-dichlorophenyl)-1,1,1-trifluoro-4-((4-methoxyphenyl)amino)butan-2-ol (73)

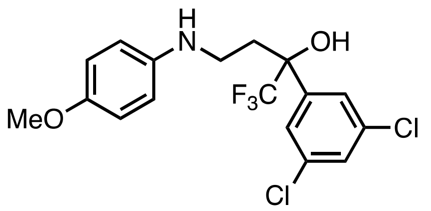

Following General Procedure B, the title compound was obtained as a brown oil by preparative TLC using petroleum ether/EtOAc (5:1) as an eluent; R_f = 0.3 (petroleum ether/EtOAc = 3:1); 17.3 mg, 44% yield. **^{1}H NMR** (500 MHz, $CDCl_3$) δ 7.55 – 7.54 (m, 2H), 7.40 – 7.39 (m, 1H), 6.81 – 6.79 (m, 2H), 6.70 – 6.68 (m, 2H), 3.76 (s, 3H), 3.42 – 3.38 (m, 1H), 3.12 – 3.06 (m, 1H), 2.48 – 2.42 (m, 1H), 2.30 – 2.25 (m, 1H). (Due to the broadening effect and intermolecular hydrogen bonding, the proton signals of NH and OH could not be observed). **^{13}C NMR** (201 MHz, $CDCl_3$) δ 155.0, 142.1, 139.9, 135.4, 128.9, 125.7, 124.9 (d, $^1J_{CF}$ = 285.6 Hz), 118.2, 115.0, 78.0 (q, $^2J_{CF}$ = 28.7 Hz), 55.8, 43.4, 32.8. **^{19}F NMR** (471 MHz, $CDCl_3$) δ -79.68 (s, 3F). **HRMS** (ESI) m/z: $[M+H]^+$ Calcd for $C_{17}H_{17}NO_2F_3Cl_2^+$ 394.0588; Found 394.0586.

1,1,1-trifluoro-4-((4-methoxyphenyl)amino)-2-(naphthalen-2-yl)butan-2-ol (74)

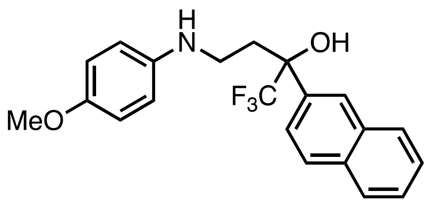

Following General Procedure B, the title compound was obtained as a yellow oil by preparative TLC using petroleum ether/EtOAc (5:1) as an eluent; R_f = 0.3 (petroleum ether/EtOAc = 3:1); 25.5 mg, 68% yield. **^{1}H NMR** (500 MHz, $CDCl_3$) δ 8.22 (s, 1H), 7.92 – 7.88 (m, 3H), 7.69 (d, J = 8.8 Hz, 1H), 7.55 – 7.52 (m, 2H), 6.75 – 6.73 (m, 2H), 6.62 – 6.60 (m, 2H), 3.73 (s, 3H), 3.37 – 3.32 (m, 1H), 3.14 – 3.09 (m, 1H), 2.56 – 2.49 (m, 2H). (Due to the broadening effect and intermolecular hydrogen bonding, the proton signals of NH and OH could not be observed). **^{13}C NMR** (126 MHz, $CDCl_3$) δ 154.4, 140.5, 135.5, 133.2, 133.1, 128.6, 128.3, 127.7, 126.9, 126.8, 126.5, 125.5 (q, $^1J_{CF}$ = 285.2 Hz), 124.1, 117.6, 114.9, 78.5 (q, $^2J_{CF}$ = 28.4 Hz), 55.7, 43.0, 33.1. **^{19}F NMR** (471 MHz, $CDCl_3$) δ -79.74 (s, 3F). **HRMS** (ESI) m/z: $[M+H]^+$ Calcd for $C_{21}H_{21}NO_2F_3^+$ 376.1524; Found 376.1525.

1,1,1-trifluoro-4-((4-methoxyphenyl)amino)-2-(thiophen-3-yl)butan-2-ol (75)

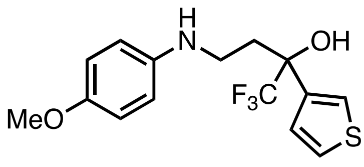

Following General Procedure B, the title compound was obtained as a yellow solid by preparative TLC using petroleum ether/EtOAc (5:1) as an eluent; R_f = 0.3 (petroleum ether/EtOAc = 3:1); 28.1 mg, 85% yield, m.p. 110 – 111 ºC. **^{1}H NMR** (500 MHz, $CDCl_3$) δ 7.53 (s, 1H), 7.39 – 7.37 (m, 1H), 7.14 – 7.13 (m, 1H), 6.78 (d, J = 8.1 Hz, 2H), 6.63 (d, J = 8.3 Hz, 2H), 3.75 (s, 3H), 3.34 – 3.30 (m, 1H), 3.15 – 3.10 (m, 1H), 2.41 – 2.25 (m, 2H). (Due to the broadening effect and intermolecular hydrogen bonding, the proton signals of NH and OH could not be observed). **^{13}C NMR** (126 MHz, $CDCl_3$) δ 154.4, 140.6, 140.0, 126.6, 126.1, 125.1 (q, $^1J_{CF}$ = 284.7 Hz), 124.4, 117.6, 114.9, 77.7 (q, $^2J_{CF}$ = 29.2 Hz), 55.8, 42.9, 33.5. **^{19}F NMR** (471 MHz, $CDCl_3$) δ -80.84 (s, 3F). **HRMS** (ESI) m/z: $[M+H]^+$ Calcd for $C_{15}H_{17}NO_2F_3S^+$ 332.0932; Found 332.0930.

2-(benzo[*d*][1,3]dioxol-5-yl)-1,1,1-trifluoro-4-((4-methoxyphenyl)amino)butan-2-ol (76)

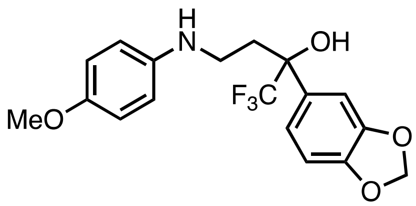

Following General Procedure B, the title compound was obtained as a yellow solid by preparative TLC using petroleum ether/EtOAc (5:1) as an eluent; R_f = 0.3 (petroleum ether/EtOAc = 3:1); 21.8 mg, 59% yield, m.p. 60 – 61 ºC. **^{1}H NMR** (500 MHz, $CDCl_3$) δ 7.13 – 7.11 (m, 2H), 6.85 (d, J = 8.2 Hz, 1H), 6.77 (d, J = 8.3 Hz, 2H), 6.64 (d, J = 8.3 Hz, 2H), 6.00 (s, 2H), 3.75 (s, 3H), 3.34 – 3.30 (m, 1H), 3.15 – 3.11 (m, 1H), 2.43 – 2.29 (m, 2H). (Due to the broadening effect and intermolecular hydrogen bonding, the proton signals of NH and OH could not be observed). **^{13}C NMR** (126 MHz, $CDCl_3$) δ 154.5, 148.0, 147.8, 140.5, 131.9, 125.3 (q, $^1J_{CF}$ = 285.0 Hz), 120.6, 117.7, 114.9, 108.2, 107.7, 101.5, 78.2 (q, $^2J_{CF}$ = 28.4 Hz), 55.8, 43.1, 33.1. **^{19}F NMR** (471 MHz, $CDCl_3$) δ -80.37 (s, 3F). **HRMS** (ESI) m/z: $[M+H]^+$ Calcd for $C_{18}H_{19}NO_4F_3^+$ 370.1266; Found 370.1264.

Methyl 2-hydroxy-4-((4-methoxyphenyl)amino)-2-phenylbutanoate (77)

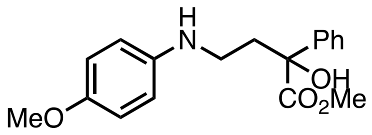

Following General Procedure B, the title compound was obtained as a yellow solid by preparative TLC using petroleum ether/EtOAc (3:1) as an eluent; R*f* = 0.2 (petroleum ether/EtOAc = 2:1); 29.9 mg, 95% yield, m.p. 65 – 66 ºC. **^{1}H NMR** (500 MHz, $CDCl_3$) *δ* 7.60 (d, *J* = 7.6 Hz, 2H), 7.38 – 7.29 (m, 3H), 6.77 (d, *J* = 8.2 Hz, 2H), 6.56 (d, *J* = 8.3 Hz, 2H), 3.96 (brs, 1H), 3.74 (s, 3H), 3.72 (s, 3H), 3.27 – 3.17 (m, 2H), 2.60 – 2.54 (m, 1H), 2.33 – 2.28 (m, 1H). (Due to the broadening effect and intermolecular hydrogen bonding, one of the proton signals of OH could not be observed). **^{13}C NMR** (126 MHz, $CDCl_3$) *δ* 175.5, 152.5, 142.1, 141.7, 128.5, 128.0, 125.4, 114.9, 114.8, 78.2, 55.9, 53.3, 40.9, 38.6. **HRMS** (ESI) m/z: $[M+H]^+$ Calcd for $C_{18}H_{22}NO_4^+$ 316.1549; Found 316.1548.

Phenethyl 2-hydroxy-4-((4-methoxyphenyl)amino)-2-methylbutanoate (78)

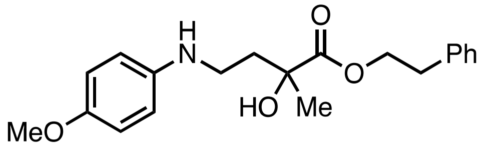

Following General Procedure B, the title compound was obtained as a yellow solid by preparative TLC using petroleum ether/EtOAc (5:1) as an eluent; R*f* = 0.3 (petroleum ether/EtOAc = 3:1); 27.1 mg, 79% yield, m.p. 76 – 77 ºC. **^{1}H NMR** (800 MHz, $CDCl_3$) *δ* 7.30 (t, *J* = 7.5 Hz, 2H), 7.23 (t, *J* = 7.4 Hz, 1H), 7.19 (d, *J* = 6.9 Hz, 2H), 6.77 (d, *J* = 8.9 Hz, 2H), 6.53 (d, *J* = 8.9 Hz, 2H), 4.38 – 4.35 (m, 1H), 4.28 – 4.25 (m, 1H), 3.74 (s, 3H), 3.55 (brs, 1H), 3.12 – 3.05 (m, 2H), 2.90 – 2.88 (m, 2H), 2.11 – 2.07 (m, 1H), 1.90 – 1.86 (m, 1H), 1.38 (s, 3H). (Due to the broadening effect and intermolecular hydrogen bonding, one of the proton signals of OH could not be observed). **^{13}C NMR** (201 MHz, $CDCl_3$) *δ* 177.1, 152.4, 142.3, 137.5, 129.0, 128.7, 126.8, 115.0, 114.5, 74.1, 66.3, 55.9, 40.7, 38.7, 35.0, 27.0. **HRMS** (ESI) m/z: $[M+H]^+$ Calcd for $C_{20}H_{26}NO_4^+$ 344.1862; Found 344.1860.

(2*R*,3*S*)-1,1,1,4,4,4-hexafluoro-3-(((4-methoxyphenyl)amino)methyl)butan-2-ol ((±)-79)

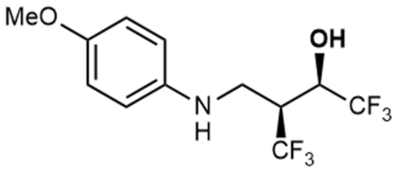

(±)-79

Following General Procedure A, the title compound was obtained as a brown oil by preparative TLC using petroleum ether/EtOAc (5:1) as an eluent; R_f = 0.3 (petroleum ether/EtOAc = 3:1); 13.1 mg, 41% yield (d.r. > 20:1). **^{1}H NMR** (500 MHz, $CDCl_3$) δ 6.82 (d, J = 9.0 Hz, 2H), 6.69 (d, J = 8.9 Hz, 2H), 4.50 – 4.45 (m, 1H), 3.76 (s, 3H), 3.71 – 3.67 (m, 1H), 3.59 – 3.55 (m, 1H), 3.00 – 2.95 (m, 1H). (Due to the broadening effect and intermolecular hydrogen bonding, the proton signals of NH and OH could not be observed). **^{13}C NMR** (201 MHz, $CDCl_3$) δ 153.9, 140.1, 126.8 (q, $^{1}J_{CF}$ = 280.8 Hz), 124.4 (q, $^{1}J_{CF}$ = 282.4 Hz), 116.0, 115.2, 68.9 (q, $^{2}J_{CF}$ = 32.8 Hz), 55.9, 43.9 (q, $^{2}J_{CF}$ = 25.4 Hz), 42.0 (q, $^{3}J_{CF}$ = 3.1 Hz). **^{19}F NMR** (471 MHz, $CDCl_3$) δ -64.98 (q, J = 7.5 Hz, 3F), -75.09 (q, J = 7.5 Hz, 3F). **HRMS** (ESI) m/z: $[M+H]^+$ Calcd for $C_{12}H_{14}NO_2F_6^+$ 318.0929; Found 318.0929.

(1-(((4-methoxyphenyl)amino)methyl)cyclopropyl)(phenyl)methanol (80)

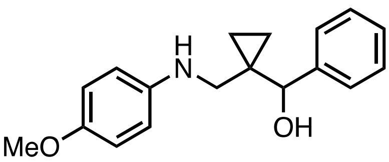

Following General Procedure B, the title compound was obtained as a yellow oil by preparative TLC using petroleum ether/EtOAc (5:1) as an eluent; R_f = 0.3 (petroleum ether/EtOAc = 3:1); 14.7 mg, 52% yield. **^{1}H NMR** (500 MHz, $CDCl_3$) δ 7.41 – 7.27 (m, 5H), 6.76 (d, J = 8.2 Hz, 2H), 6.58 (d, J = 7.9 Hz, 2H), 4.47 – 4.30 (m, 1H), 3.81 – 3.75 (m, 4H), 3.31 (d, J = 12.3 Hz, 1H), 2.61 (d, J = 12.0 Hz, 1H), 0.81 – 0.75 (m, 2H), 0.65 – 0.52 (m, 2H). (Due to the broadening effect and intermolecular hydrogen bonding, one of the proton signals of OH could not be observed). **^{13}C NMR** (126 MHz, $CDCl_3$) δ 153.2, 142.8, 142.1, 128.3, 127.5, 126.3, 115.9, 114.8, 80.1, 55.9, 51.5, 26.1, 10.1, 9.4. **HRMS** (ESI) m/z: $[M+H]^+$ Calcd for $C_{18}H_{22}NO_2^+$ 284.1651; Found 284.1649.

1-(dimethyl(phenyl)silyl)-3-((4-methoxyphenyl)amino)propan-1-ol (81)

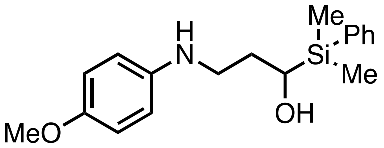

Following General Procedure B, the title compound was obtained as a brown oil by preparative TLC using petroleum ether/EtOAc (5:1) as an eluent; R_f = 0.3

(petroleum ether/EtOAc = 3:1); 7.9 mg, 25% yield. **^{1}H NMR** (500 MHz, $CDCl_3$) δ 7.57 – 7.56 (m, 2H), 7.39 – 7.36 (m, 3H), 6.78 – 6.76 (m, 2H), 6.60 – 6.58 (m, 2H), 3.78 – 3.73 (m, 4H), 3.28 – 3.24 (m, 2H), 1.83 – 1.79 (m, 2H), 0.36 (s, 6H). (Due to the broadening effect and intermolecular hydrogen bonding, the proton signals of NH and OH could not be observed). **^{13}C NMR** (126 MHz, $CDCl_3$) δ 152.6, 142.6, 136.5, 134.3, 129.6, 128.1, 115.0, 115.0, 65.4, 55.9, 45.1, 32.6, -5.5, -5.6. **HRMS** (ESI) m/z: $[M+H]^+$ Calcd for $C_{18}H_{26}NO_2Si^+$ 316.1733; Found 316.1732.

Diethyl (1-hydroxy-3-((4-methoxyphenyl)amino)propyl)phosphonate (82)

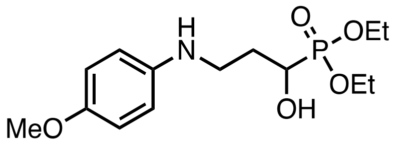

Following General Procedure B, the title compound was obtained as a brown oil by preparative TLC using petroleum ether/EtOAc (5:1) as an eluent; R_f = 0.3 (petroleum ether/EtOAc = 3:1); 11.7 mg, 37% yield. **^{1}H NMR** (500 MHz, $CDCl_3$) δ 6.78 (d, J = 8.1 Hz, 2H), 6.64 (d, J = 8.3 Hz, 2H), 4.18 – 4.08 (m, 5H), 3.74 (s, 3H), 3.42 – 3.29 (m, 2H), 2.08 – 1.97 (m, 2H), 1.34 – 1.30 (m, 6H). (Due to the broadening effect and intermolecular hydrogen bonding, the proton signals of NH and OH could not be observed). **^{13}C NMR** (126 MHz, $CDCl_3$) δ 152.7, 142.1, 115.1, 115.0, 67.1 (d, J = 163.2 Hz), 62.8 (d, J = 7.1 Hz), 55.9, 42.7 (d, J = 14.6 Hz), 30.7, 16.6 (d, J = 5.5 Hz). **^{31}P NMR** (202 MHz, $CDCl_3$) δ 24.4. **HRMS** (ESI) m/z: $[M+H]^+$ Calcd for $C_{14}H_{25}NO_5P^+$ 318.1470; Found 318.1469.

1,1,1-trifluoro-4-((4-methoxyphenyl)amino)hexan-2-ol ((±)-83)

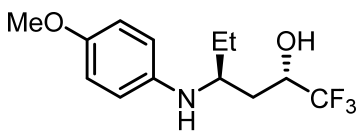

(±)-83

Following General Procedure A, the title compound was obtained as a brown oil by preparative TLC using petroleum ether/EtOAc (20:1) as an eluent; R_f = 0.4 (petroleum ether/EtOAc = 10:1); 11.4 mg, 41% yield (d.r. > 20:1). **^{1}H NMR** (500 MHz, $CDCl_3$) δ 6.82 – 6.80 (m, 2H), 6.77 – 6.75 (m, 2H), 4.32 – 4.28 (m, 1H), 3.76 (s, 3H), 3.49 – 3.42 (m, 1H), 1.97 – 1.93 (m, 1H), 1.69 – 1.62 (m, 2H), 1.50 – 1.43 (m, 1H), 0.90 (t, J = 7.4 Hz, 3H). (Due to the broadening effect and intermolecular hydrogen bonding, the proton signals of NH and OH could not be

observed). **^{13}C NMR** (201 MHz, CDCl$_3$) δ 154.6, 139.2, 124.8 (q, $^1J_{CF}$ = 280.7 Hz), 118.8, 115.0, 71.4 (q, $^2J_{CF}$ = 31.2 Hz), 58.1, 55.8, 27.8, 9.7. **^{19}F NMR** (471 MHz, CDCl$_3$) δ -80.72 (s, 3F). **HRMS** (ESI) m/z: $[M+H]^+$ Calcd for $C_{13}H_{19}NO_2F_3^+$ 278.1368; Found 278.1369.

1,1,1-trifluoro-4-((4-methoxyphenyl)amino)octan-2-ol ((±)-84)

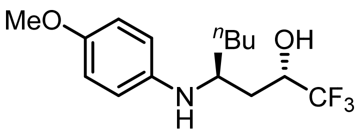

(±)-84

Following General Procedure A, the title compound was obtained as a brown oil by preparative TLC using petroleum ether/EtOAc (20:1) as an eluent; R$_f$ = 0.4 (petroleum ether/EtOAc = 10:1); 7.6 mg, 25% yield (d.r. > 20:1). **^{1}H NMR** (500 MHz, CDCl$_3$) δ 6.81 – 6.68 (m, 4H), 4.32 – 4.22 (m, 1H), 3.77 (s, 3H), 3.51 – 3.43 (m, 1H), 3.21 (brs, 1H), 2.01 – 1.94 (m, 1H), 1.75 – 1.59 (m, 3H), 1.47 – 1.29 (m, 3H), 0.93 – 0.84 (m, 4H). (Due to the broadening effect and intermolecular hydrogen bonding, one of the proton signals of OH could not be observed). **^{13}C NMR** (126 MHz, CDCl$_3$) δ 154.4, 139.5, 124.8 (q, $^1J_{CF}$ = 280.2 Hz), 118.6, 115.0, 71.4 (q, $^2J_{CF}$ = 31.3 Hz), 56.8, 55.8, 35.1, 32.8, 27.7, 22.8, 14.1. **^{19}F NMR** (471 MHz, CDCl$_3$) δ -80.70 (s, 3F). **HRMS** (ESI) m/z: $[M+H]^+$ Calcd for $C_{15}H_{23}NO_2F_3^+$ 306.1681; Found 306.1683.

1,1,1-trifluoro-4-((4-methoxyphenyl)amino)-6-methylheptan-2-ol ((±)-85)

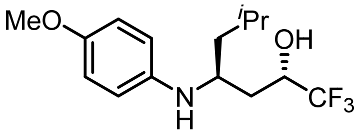

(±)-85

Following General Procedure A, the title compound was obtained as a brown oil by preparative TLC using petroleum ether/EtOAc (20:1) as an eluent; R$_f$ = 0.3 (petroleum ether/EtOAc = 10:1); 6.4 mg, 21% yield (d.r. > 20:1). **^{1}H NMR** (500 MHz, CDCl$_3$) δ 6.80 (d, J = 8.2 Hz, 2H), 6.73 (d, J = 8.5 Hz, 2H), 4.32 – 4.23 (m, 1H), 3.77 (s, 3H), 3.55 – 3.46 (m, 1H), 3.28 (brs, 1H), 2.01 – 1.95 (m, 1H), 1.68 – 1.57 (m, 3H), 1.50 – 1.45 (m, 1H), 0.90 – 0.84 (m, 6H). (Due to the broadening effect and intermolecular hydrogen bonding, one of the proton signals of OH could not be observed). **^{13}C NMR** (126 MHz, CDCl$_3$) δ 154.3, 139.6, 124.8 (q, $^1J_{CF}$ = 281.1 Hz), 118.2, 115.0, 71.3 (q, $^2J_{CF}$ = 31.3 Hz), 55.8, 54.8, 45.6, 33.2, 25.0, 23.3,

22.1. **^{19}F NMR** (471 MHz, $CDCl_3$) δ -80.68 (s, 3F). **HRMS** (ESI) m/z: $[M+H]^+$ Calcd for $C_{15}H_{23}NO_2F_3^+$ 306.1681; Found 306.1680.

1,1,1-trifluoro-5-(2-methoxyethoxy)-4-((4-methoxyphenyl)amino)pentan-2-ol ((±)-86)

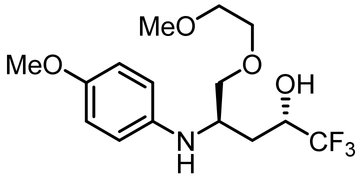

(±)-86

Following General Procedure A, the title compound was obtained as a colourless crystalline solid by preparative TLC using petroleum ether/EtOAc (20:1) as an eluent; R_f = 0.3 (petroleum ether/EtOAc = 10:1); 12.8 mg, 38% yield (d.r. > 20:1), m.p. 62 – 63 ºC. **^{1}H NMR** (500 MHz, $CDCl_3$) δ 6.80 (d, J = 8.2 Hz, 2H), 6.75 (d, J = 8.5 Hz, 2H), 4.32 – 4.28 (m, 1H), 3.76 (s, 3H), 3.66 – 3.51 (m, 7H), 3.39 (s, 3H), 1.98 – 1.95 (m, 2H). (Due to the broadening effect and intermolecular hydrogen bonding, the proton signals of NH and OH could not be observed). **^{13}C NMR** (201 MHz, $CDCl_3$) δ 154.2, 139.5, 125.0 (q, $^1J_{CF}$ = 280.7 Hz), 118.4, 115.0, 72.0, 71.9, 70.8, 70.0 (q, $^2J_{CF}$ = 31.2 Hz), 59.2, 55.8, 55.1, 31.5. **^{19}F NMR** (471 MHz, $CDCl_3$) δ -80.44 (s, 3F). **HRMS** (ESI) m/z: $[M+H]^+$ Calcd for $C_{15}H_{23}NO_4F_3^+$ 338.1579; Found 338.1578.

4-((4,4,4-trifluoro-3-hydroxybutyl)amino)phenyl 2-(4-isobutylphenyl)propanoate (87)

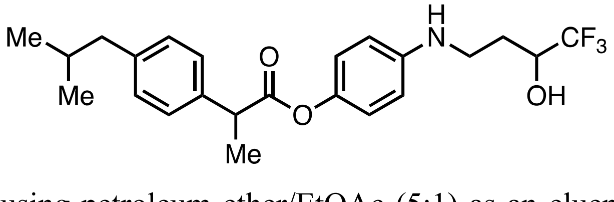

Following General Procedure A, the title compound was obtained as a yellow solid by preparative TLC using petroleum ether/EtOAc (5:1) as an eluent; R_f = 0.3 (petroleum ether/EtOAc = 3:1); 17.8 mg, 42% yield, m.p. 83 – 84 ºC. **^{1}H NMR** (500 MHz, $CDCl_3$) δ 7.30 – 7.28 (m, 2H), 7.13 (d, J = 7.6 Hz, 2H), 6.83 – 6.81 (m, 2H), 6.60 – 6.57 (m, 2H), 4.12 – 4.07 (m, 1H), 3.92 – 3.88 (m, 1H), 3.47 – 3.26 (m, 3H), 2.46 (d, J = 6.7 Hz, 2H), 2.01 – 1.95 (m, 1H), 1.90 – 1.82 (m, 2H), 1.58 (d, J = 6.9 Hz, 3H), 0.91 (d, J = 6.4 Hz, 6H). (Due to the broadening effect and intermolecular hydrogen bonding, one of the proton signals of OH could not be observed). **^{13}C NMR** (126 MHz, $CDCl_3$) δ 174.1, 145.6, 143.1,

140.9, 137.5, 129.6, 127.3, 125.1 (q, $^1J_{CF}$ = 281.7 Hz), 122.2, 114.0, 69.6 (q, $^2J_{CF}$ = 31.5 Hz), 45.3, 45.2, 41.2, 30.3, 29.0, 22.5, 18.7. **^{19}F NMR** (471 MHz, $CDCl_3$) δ -79.91 (s, 3F). **HRMS** (ESI) m/z: $[M+H]^+$ Calcd for $C_{23}H_{29}NO_3F_3^+$ 424.2100; Found 424.2098.

4-((4-(2,4-dichlorophenoxy)phenyl)amino)-1,1,1-trifluorobutan-2-ol (88)

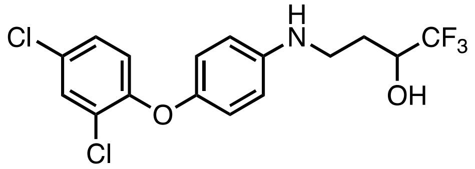

Following General Procedure A, the title compound was obtained as a brown oil by preparative TLC using petroleum ether/EtOAc (5:1) as an eluent; R$_f$ = 0.3 (petroleum ether/EtOAc = 3:1); 19.6 mg, 51% yield. **^{1}H NMR** (500 MHz, $CDCl_3$) δ 7.43 (s, 1H), 7.11 (d, J = 8.8 Hz, 1H), 6.90 – 6.87 (m, 2H), 6.76 (d, J = 8.7 Hz, 1H), 6.69 – 6.66 (m, 2H), 4.21 – 4.15 (m, 1H), 3.46 – 3.32 (m, 2H), 3.03 (brs, 2H), 2.07 – 1.89 (m, 2H). (Due to the broadening effect and intermolecular hydrogen bonding, one of the proton signals of OH could not be observed). **^{13}C NMR** (126 MHz, $CDCl_3$) δ 153.2, 148.3, 144.5, 130.4, 127.9, 127.8, 125.1, 125.1 (q, $^1J_{CF}$ = 281.5 Hz), 120.7, 119.2, 115.0, 69.7 (q, $^2J_{CF}$ = 31.4 Hz), 41.5, 29.0. **^{19}F NMR** (471 MHz, $CDCl_3$) δ -79.89 (s, 3F). **HRMS** (ESI) m/z: $[M+H]^+$ Calcd for $C_{16}H_{15}NO_2F_3Cl_2^+$ 380.0432; Found 380.0430.

3-((4-butylphenyl)amino)-1-(4-fluorophenyl)propan-1-ol (89)

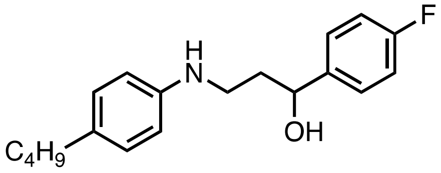

Following General Procedure B, the title compound was obtained as a yellow solid by preparative TLC using petroleum ether/EtOAc (5:1) as an eluent; R$_f$ = 0.3 (petroleum ether/EtOAc = 3:1); 22.6 mg, 75% yield, m.p. 88 – 90 ºC. **^{1}H NMR** (500 MHz, $CDCl_3$) δ 7.35 – 7.26 (m, 2H), 7.06 – 7.01 (m, 4H), 6.60 (d, J = 7.8 Hz, 2H), 4.91 – 4.88 (m, 1H), 3.38– 3.26 (m, 3H), 2.53 – 2.50 (m, 2H), 2.05 – 1.99 (m, 2H), 1.58 – 1.52 (m, 2H), 1.38 – 1.32 (m, 2H), 0.92 (t, J = 7.4 Hz, 3H). (Due to the broadening effect and intermolecular hydrogen bonding, one of the proton signals of OH could not be observed). **^{13}C NMR** (126 MHz, $CDCl_3$) δ 162.3 (d, $^1J_{CF}$ = 245.4 Hz), 146.0, 140.3 (d, $^3J_{CF}$ = 2.9 Hz), 132.9, 129.3, 127.5 (d, $^3J_{CF}$ = 8.1 Hz), 115.4 (d, $^2J_{CF}$ = 21.2 Hz), 113.8, 73.4, 42.5, 38.5, 34.8, 34.1, 22.5, 14.1. **^{19}F NMR** (471 MHz,

$CDCl_3$) δ -115.07 (s, 1F). **HRMS** (ESI) m/z: $[M+H]^+$ Calcd for $C_{19}H_{25}NOF^+$ 302.1920; Found 302.1919.

3-((4-ethoxyphenyl)amino)-1-(naphthalen-2-yl)propan-1-ol (90)

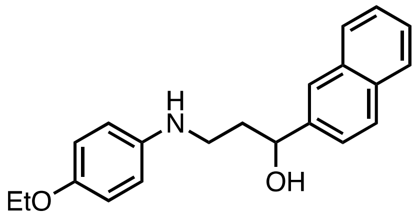

Following General Procedure B, the title compound was obtained as a yellow solid by preparative TLC using petroleum ether/EtOAc (5:1) as an eluent; R_f = 0.3 (petroleum ether/EtOAc = 3:1); 18.0 mg, 56% yield, m.p. 99 – 100 ºC. **¹H NMR** (500 MHz, $CDCl_3$) δ 7.87 – 7.81 (m, 4H), 7.47 – 7.46 (m, 3H), 6.77 (d, *J* = 8.0 Hz, 2H), 6.61 (d, *J* = 8.2 Hz, 2H), 5.07 – 5.05 (m, 1H), 3.97– 3.93 (m, 2H), 3.39 (brs, 1H), 3.27 – 3.25 (m, 2H), 2.11 – 2.08 (m, 2H), 1.41 – 1.35 (m, 3H). (Due to the broadening effect and intermolecular hydrogen bonding, one of the proton signals of OH could not be observed). **¹³C NMR** (126 MHz, $CDCl_3$) δ 152.1, 142.2, 141.9, 133.4, 133.0, 128.4, 128.1, 127.8, 126.3, 126.0, 124.4, 124.1, 115.8, 115.3, 74.2, 64.2, 43.4, 38.2, 15.1. **HRMS** (ESI) m/z: $[M+H]^+$ Calcd for $C_{21}H_{24}NO_2^+$ 322.1807; Found 322.1808.

1-(naphthalen-2-yl)-3-((4-phenoxyphenyl)amino)propan-1-ol (91)

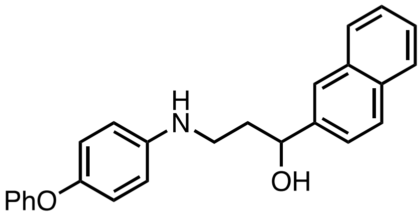

Following General Procedure B, the title compound was obtained as a yellow solid by preparative TLC using petroleum ether/EtOAc (5:1) as an eluent; R_f = 0.3 (petroleum ether/EtOAc = 3:1); 25.9 mg, 70% yield, m.p. 76 – 77 ºC. **¹H NMR** (500 MHz, $CDCl_3$) δ 7.81 – 7.77 (m, 5H), 7.46 – 7.43 (m, 3H), 7.27 – 7.21 (m, 1H), 7.00 – 6.87 (m, 5H), 6.57 (d, *J* = 8.3 Hz, 2H), 5.00 – 4.98 (m, 1H), 3.41 (brs, 1H), 3.30– 3.13 (m, 2H), 2.12 – 2.01 (m, 2H). (Due to the broadening effect and intermolecular hydrogen bonding, one of the proton signals of OH could not be observed). **¹³C NMR** (126 MHz, $CDCl_3$) δ 159.1, 148.1, 144.9, 141.8, 133.4, 133.0, 129.6, 128.5, 128.0, 127.8, 126.4, 126.0, 124.5, 124.0, 122.1, 121.2, 117.2, 114.5, 73.9, 42.5, 38.1. **HRMS** (ESI) m/z: $[M+H]^+$ Calcd for $C_{25}H_{25}NO_2^+$ 370.1807; Found 370.1809.

1-(naphthalen-2-yl)-3-(phenylamino)propan-1-ol (92)

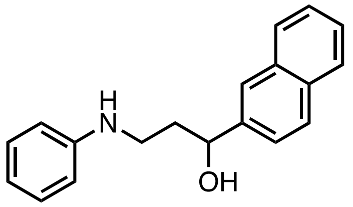

Following General Procedure B, the title compound was obtained as a brown solid by preparative TLC using petroleum ether/EtOAc (5:1) as an eluent; R$_f$ = 0.3 (petroleum ether/EtOAc = 3:1); 23.2 mg, 84% yield, m.p. 79 – 80 ºC. **^{1}H NMR** (500 MHz, $CDCl_3$) δ 7.89 – 7.81 (m, 4H), 7.54 – 7.45 (m, 3H), 7.31 (t, J = 7.8 Hz, 2H), 7.14 (d, J = 8.0 Hz, 2H), 6.99 (t, J = 7.3 Hz, 1H), 5.34 – 5.31 (m, 1H), 3.81– 3.70 (m, 2H), 2.73 – 2.66 (m, 1H), 2.38 – 2.32 (m, 1H). (Due to the broadening effect and intermolecular hydrogen bonding, the proton signals of NH and OH could not be observed). **^{13}C NMR** (126 MHz, $CDCl_3$) δ 151.8, 137.7, 133.3, 133.2, 129.0, 128.6, 128.1, 127.8, 126.4, 126.2, 125.7, 124.4, 121.9, 115.2, 79.3, 54.3, 37.1. **HRMS** (ESI) m/z: $[M+H]^+$ Calcd for $C_{19}H_{20}NO^+$ 278.1545; Found 278.1543.

3-((4-ethoxyphenyl)amino)-1-(4-fluorophenyl)propan-1-ol (93)

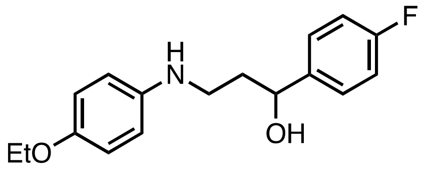

Following General Procedure B, the title compound was obtained as a brown solid by preparative TLC using petroleum ether/EtOAc (5:1) as an eluent; R$_f$ = 0.3 (petroleum ether/EtOAc = 3:1); 19.1 mg, 66% yield, m.p. 97 – 98 ºC. **^{1}H NMR** (500 MHz, $CDCl_3$) δ 7.34 – 7.31 (m, 2H), 7.03 (t, J = 8.5 Hz, 2H), 6.78 (d, J = 8.1 Hz, 2H), 6.62 (d, J = 8.2 Hz, 2H), 4.91 – 4.88 (m, 1H), 3.98– 3.94 (m, 2H), 3.36 (brs, 1H), 3.25 – 3.23 (m, 2H), 1.99 (q, J = 6.2 Hz, 2H), 1.37 (t, J = 6.9 Hz, 3H). (Due to the broadening effect and intermolecular hydrogen bonding, one of the proton signals of OH could not be observed). **^{13}C NMR** (126 MHz, $CDCl_3$) δ 162.2 (d, $^1J_{CF}$ = 245.2 Hz), 152.2, 142.1, 140.3 (d, $^3J_{CF}$ = 3.2 Hz), 127.4 (d, $^3J_{CF}$ = 7.9 Hz), 115.8, 115.4, 115.4 (d, $^2J_{CF}$ = 21.3 Hz), 73.6, 64.2, 43.5, 38.4, 15.1. **^{19}F NMR** (471 MHz, $CDCl_3$) δ -115.17 (s, 1F). **HRMS** (ESI) m/z: $[M+H]^+$ Calcd for $C_{17}H_{21}NO_2F^+$ 290.1556; Found 290.1554.

1-(4-chlorophenyl)-3-((4-methoxyphenyl)amino)propan-1-ol (94)

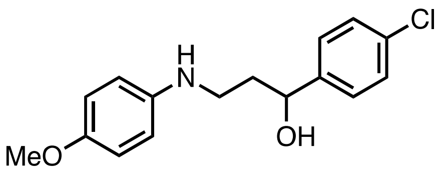

Following General Procedure B, the title compound was obtained as a brown solid by preparative TLC using petroleum ether/EtOAc (5:1) as an eluent; R*f* = 0.3 (petroleum ether/EtOAc = 3:1); 7.6 mg, 26% yield, m.p. 81 – 82 ºC. **^{1}H NMR** (500 MHz, $CDCl_3$) δ 7.33 – 7.29 (m, 4H), 6.79 (d, J = 8.2 Hz, 2H), 6.64 (d, J = 8.3 Hz, 2H), 4.93 – 4.90 (m, 1H), 3.75 (s, 3H), 3.40 (brs, 1H), 3.27– 3.25 (m, 2H), 2.01 – 1.97 (m, 2H). (Due to the broadening effect and intermolecular hydrogen bonding, one of the proton signals of OH could not be observed). **^{13}C NMR** (126 MHz, $CDCl_3$) δ 153.0, 143.1, 142.1, 133.3, 128.8, 127.2, 115.5, 115.0, 73.7, 55.9, 43.5, 38.4. **HRMS** (ESI) m/z: $[M+H]^+$ Calcd for $C_{16}H_{19}NO_2Cl^+$ 292.1104; Found 292.1105.

***Tert*-butyl 2-hydroxy-4-(phenylamino)butanoate (113)**

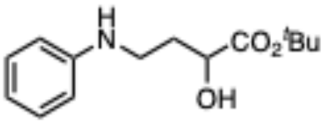

Following General Procedure B, the title compound was obtained as a yellow solid by preparative TLC using petroleum ether/EtOAc (5:1) as an eluent; R*f* = 0.3 (petroleum ether/EtOAc = 3:1); 23.6 mg, 94% yield, m.p. 65 – 66 ºC. **^{1}H NMR** (500 MHz, $CDCl_3$) δ 7.18 (t, J = 7.6 Hz, 2H), 6.70 (t, J = 7.4 Hz, 1H), 6.62 (d, J = 7.9 Hz, 2H), 4.20 – 4.18 (m, 1H), 3.33 – 3.30 (m, 2H), 3.05 (brs, 1H), 2.13 – 2.07 (m, 1H), 1.96– 1.90 (m, 1H), 1.48 (s, 9H). (Due to the broadening effect and intermolecular hydrogen bonding, one of the proton signals of OH could not be observed). **^{13}C NMR** (126 MHz, $CDCl_3$) δ 174.4, 148.3, 129.4, 117.5, 112.9, 83.0, 69.6, 40.5, 33.7, 28.1. **HRMS** (ESI) m/z: $[M+H]^+$ Calcd for $C_{14}H_{22}NO_3^+$ 252.1600; Found 252.1600.

Diverse Functionalization of the *N*-arylamino alcohols[4-14].

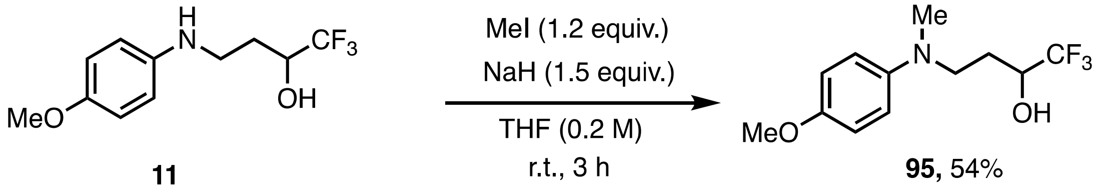

To a 10 mL round bottom flask equipped with a magnetic stirring bar was added, **11** (1.0 equiv., 0.1 mmol, 24.9 mg) was dissolved in dry THF (0.5 mL, 0.2 M) and cooled to 0 °C in an ice-bath.. To this solution was added NaH (60% in mineral oil, 1.5 equiv., 0.15 mmol, 6.0 mg,). The mixture was stirred at 0 ºC for 1 h before adding MeI (1.2 equiv., 0.12 mmol, 15.0 μL) dropwise. Then the mixture was allowed to warm to room temperature and stirred for another 3 h. Upon completion of the reactions (TLC showed complete consumption of starting material). The reaction was quenched with saturated NH_4Cl (10 mL) and extracted with EtOAc (3 × 10 mL). The combined organic layers were washed with brine, dried over anhydrous Na_2SO_4, filtered and concentrated in *vacuo*. The residue was purified by preparative thin-layer chromatography using a mixture of petroleum ether and ethyl acetate (PE:EA = 5:1) as an eluent to afford the product **95.**

1,1,1-trifluoro-4-((4-methoxyphenyl)(methyl)amino)butan-2-ol (95). The title compound was obtained as a brown oil by preparative TLC using petroleum ether/EtOAc (5:1) as an eluent; R_f= 0.4 (petroleum ether/EtOAc = 3:1); 14.2 mg, 54% yield. **^{1}H NMR** (500 MHz, $CDCl_3$) δ 6.80 (d, J = 9.0 Hz, 2H), 6.60 (d, J = 9.1 Hz, 2H), 3.75 (s, 3H), 3.71 – 3.66 (m, 1H), 3.57 (s, 3H), 3.33 – 3.23 (m, 2H), 1.96 – 1.84 (m, 2H). (Due to the broadening effect and intermolecular hydrogen bonding, one of the proton signals of OH could not be observed). **^{13}C NMR** (126 MHz, $CDCl_3$) δ 152.5, 142.3, 125.6 (q, $^1J_{CF}$ = 284.4 Hz), 115.1, 114.4, 77.8 (q, $^2J_{CF}$ = 29.5 Hz), 60.9, 55.9, 40.8, 29.1. **^{19}F NMR** (471 MHz, $CDCl_3$) δ -76.65 (s, 3F). **HRMS** (ESI) m/z: $[M+H]^+$ Calcd for $C_{12}H_{17}NO_2F_3^+$ 264.1211; Found 264.1212.

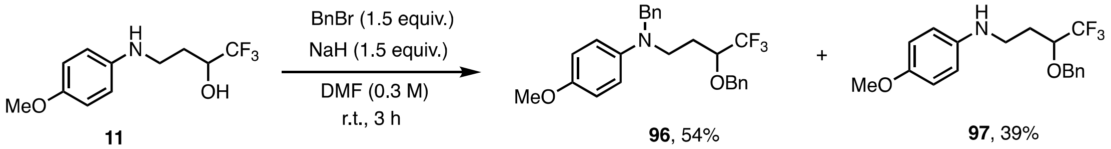

To a 10 mL round bottom flask equipped with a magnetic stirring bar was added, **11** (1.0 equiv., 0.1 mmol, 24.9 mg) was dissolved in dry *N*,*N*-dimethylformamide (0.2 mL) and cooled to 0 °C in an ice-bath. To this solution was added NaH (60% in mineral oil, 1.5 equiv., 0.15 mmol, 6.0 mg,) and it was stirred for 1 h. A solution of benzyl bromide (1.5 equiv., 0.15 mmol, 18.0 μL) in *N*,*N*-dimethylformamide solution (0.1 mL) was added dropwise to the reaction mixture at 0 ºC. Then the mixture was allowed to warm to room temperature and stirred for another 3 h. Upon completion of the reactions (TLC showed complete consumption of starting material). The reaction was quenched with saturated NH_4Cl (10 mL) and extracted with EtOAc (3 × 10 mL). The combined organic layers were were washed with brine, dried over anhydrous Na_2SO_4, filtered and concentrated in *vacuo*. The residue was purified by preparative thin-layer chromatography using a mixture of petroleum ether and ethyl acetate (PE:EA = 10:1) as an eluent to afford the product **96** and **97**.

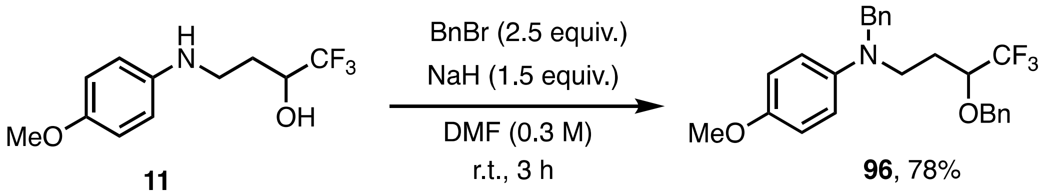

To a 10 mL round bottom flask equipped with a magnetic stirring bar was added, **11** (1.0 equiv., 0.1 mmol, 24.9 mg) was dissolved in dry *N*,*N*-dimethylformamide (0.2 mL) and cooled to 0 °C in an ice-bath. To this solution was added NaH (60% in mineral oil, 1.5 equiv., 0.15 mmol, 6.0 mg,) and it was stirred for 1 h. A solution of benzyl bromide (2.5 equiv., 0.25 mmol, 30.0 μL) in *N*,*N*-dimethylformamide solution (0.1 mL) was added dropwise to the reaction mixture at 0 ºC. Then the mixture was allowed to warm to room temperature and stirred for another 3 h. Upon completion of the reactions (TLC showed complete consumption of starting material). The reaction was quenched with saturated NH_4Cl (10 mL) and extracted with EtOAc (3 × 10 mL). The combined organic

layers were were washed with brine, dried over anhydrous Na_2SO_4, filtered and concentrated in *vacuo*. The residue was purified by preparative thin-layer chromatography using a mixture of petroleum ether and ethyl acetate (PE:EA = 10:1) as an eluent to afford the product **96**.

***N*-benzyl-*N*-(3-(benzyloxy)-4,4,4-trifluorobutyl)-4-methoxyaniline (96).** The title compound was obtained as a yellow oil by preparative TLC using petroleum ether/EtOAc (10:1) as an eluent; R_f= 0.5 (petroleum ether/EtOAc = 5:1); 33.6 mg, 78% yield. **^{1}H NMR** (500 MHz, $CDCl_3$) δ 7.34 – 7.18 (m, 10H), 6.77 (d, J = 8.5 Hz, 2H), 6.70 (d, J = 8.6 Hz, 2H), 4.80 (d, J = 11.3 Hz, 1H), 4.41 (d, J = 11.4 Hz, 1H), 4.38 – 4.30 (m, 2H), 3.78 – 3.73 (m, 4H), 3.43 – 3.26 (m, 2H), 1.98 – 1.83 (m, 2H). **^{13}C NMR** (126 MHz, $CDCl_3$) δ 152.4, 143.2, 139.0, 137.0, 128.7, 128.6, 128.3, 128.3, 127.3, 127.1, 125.7 (q, $^1J_{CF}$ = 284.4 Hz), 116.0, 114.9, 75.1 (q, $^2J_{CF}$ = 29.5 Hz), 74.4, 56.5, 55.8, 47.2, 26.9. **^{19}F NMR** (471 MHz, $CDCl_3$) δ -76.02 (s, 3F). **HRMS** (ESI) m/z: $[M+H]^+$ Calcd for $C_{25}H_{27}NO_2F_3^+$ 430.1994; Found 430.1995.

***N*-(3-(benzyloxy)-4,4,4-trifluorobutyl)-4-methoxyaniline (97).** The title compound was obtained as a yellow oil by preparative TLC using petroleum ether/EtOAc (10:1) as an eluent; R_f= 0.2 (petroleum ether/EtOAc = 5:1); 13.3 mg, 39% yield. **^{1}H NMR** (500 MHz, $CDCl_3$) δ 7.40 – 7.30 (m, 5H), 6.75 (d, J = 8.3 Hz, 2H), 6.44 (d, J = 8.3 Hz, 2H), 4.89 (d, J = 11.4 Hz, 1H), 4.56 (d, J = 11.5 Hz, 1H), 3.92 – 3.89 (m, 1H), 3.74 (s, 3H), 3.16 – 3.14 (m, 2H), 1.93 – 1.84 (m, 2H). (Due to the broadening effect and intermolecular hydrogen bonding, one of the proton signals of NH could not be observed). **^{13}C NMR** (126 MHz, $CDCl_3$) δ 152.3, 142.1, 136.9, 128.8, 128.8, 128.6, 125.8 (q, $^1J_{CF}$ = 284.4 Hz), 115.0, 114.2, 74.6 (q, $^2J_{CF}$ = 29.5 Hz), 74.5, 55.9, 40.4, 28.8. **^{19}F NMR** (471 MHz, $CDCl_3$) δ -76.22 (s, 3F). **HRMS** (ESI) m/z: $[M+H]^+$ Calcd for $C_{18}H_{21}NO_2F_3^+$ 340.1524; Found 340.1523.

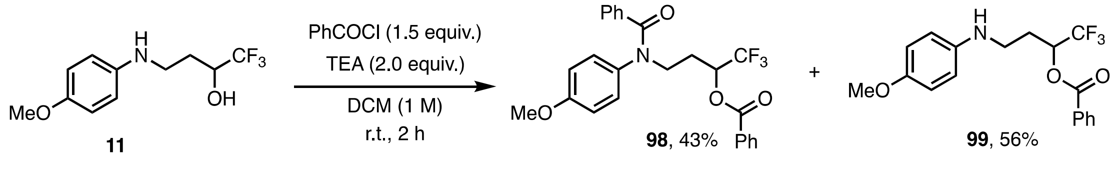

To a stirred solution of **11** (1.0 equiv., 0.1 mmol, 24.9 mg) in dry dichloromethane (1 mL, 0.1 M) were added triethylamine (2.0 equiv., 0.2 mmol, 28.0 μL) and Benzoyl chloride (1.5 equiv., 0.15 mmol, 18.0 μL,) dropwisely under 0 °C. Then the mixture was allowed to warm to room temperature and stirred for 2 h. Upon completion of the reactions (TLC showed complete consumption of starting material). The reaction was quenched with H_2O (10 mL) and extracted with CH_2Cl_2 (3 × 10 mL). The combined organic layers were were washed with brine, dried over anhydrous $MgSO_4$, filtered and concentrated in *vacuo*. The residue was purified by preparative thin-layer chromatography using a mixture of petroleum ether and ethyl acetate (PE:EA = 10:1) as an eluent to afford the product **98** and **99**.

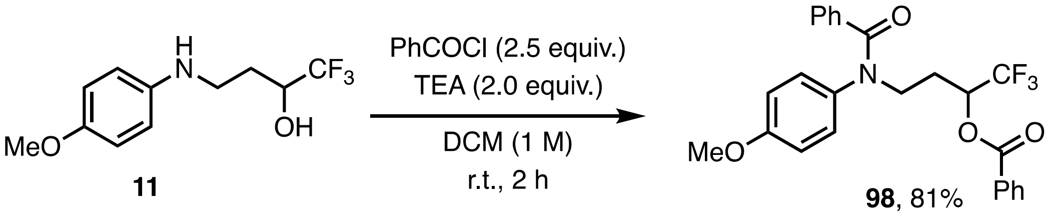

11 **98**, 81%

To a stirred solution of **11** (1.0 equiv., 0.1 mmol, 24.9 mg) in dry dichloromethane (1 mL, 0.1 M) were added triethylamine (2.0 equiv., 0.2 mmol, 28.0 μL) and Benzoyl chloride (2.5 equiv., 0.2 mmol, 29.0 μL,) dropwisely under 0 °C. Then the mixture was allowed to warm to room temperature and stirred for 2 h. Upon completion of the reactions (TLC showed complete consumption of starting material). The reaction was quenched with H_2O (10 mL) and extracted with CH_2Cl_2 (3 × 10 mL). The combined organic layers were were washed with brine, dried over anhydrous $MgSO_4$, filtered and concentrated in *vacuo*. The residue was purified by preparative thin-layer chromatography using a mixture of petroleum ether and ethyl acetate (PE:EA = 10:1) as an eluent to afford the product **98**.

1,1,1-trifluoro-4-(*N*-(4-methoxyphenyl)benzamido)butan-2-yl benzoate (98). The title compound was obtained as a white solid by preparative TLC using petroleum ether/EtOAc (10:1) as an eluent; R_f= 0.5 (petroleum ether/EtOAc = 5:1); 37.0 mg, 81% yield, m.p. 133 – 134 ºC. **^{1}H NMR** (500 MHz, $CDCl_3$) δ 8.07 (d, J = 7.6 Hz, 2H), 7.62 – 7.42 (m, 3H), 7.26 – 7.12 (m, 5H), 6.92 (d, J = 8.2 Hz, 2H), 6.72 (d, J = 8.3 Hz, 2H),

5.74 – 5.53 (m, 1H), 4.10 – 3.94 (m, 2H), 3.73 (s, 3H), 2.30 – 2.25 (m, 2H). **^{13}C NMR** (126 MHz, $CDCl_3$) δ 170.6, 165.1, 158.3, 135.9, 135.6, 133.9, 130.2, 129.7, 128.9, 128.7, 127.8, 123.9 (q, $^1J_{CF}$ = 280.6 Hz), 114.6, 68.3 (q, $^2J_{CF}$ = 32.8 Hz), 55.4, 46.1, 26.2. **^{19}F NMR** (471 MHz, $CDCl_3$) δ -76.84 (s, 3F). **HRMS** (ESI) m/z: $[M+Na]^+$ Calcd for $C_{25}H_{22}NO_4F_3Na^+$ 480.1399; Found 480.1398.

1,1,1-trifluoro-4-((4-methoxyphenyl)amino)butan-2-yl benzoate (99). The title compound was obtained as a brown oil by preparative TLC using petroleum ether/EtOAc (10:1) as an eluent; R_f= 0.2 (petroleum ether/EtOAc = 5:1); 19.6 mg, 56% yield. **^{1}H NMR** (500 MHz, $CDCl_3$) δ 7.31 – 7.25 (m, 3H), 7.19 – 7.16 (m, 2H), 6.94 (d, J = 8.1 Hz, 2H), 6.77 (d, J = 8.2 Hz, 2H), 5.19 (brs, 1H), 4.61 – 4.55 (m, 1H), 4.07 – 4.04 (m, 1H), 3.75 – 3.70 (m, 4H), 1.93 – 1.77 (m, 2H). **^{13}C NMR** (126 MHz, $CDCl_3$) δ 172.5, 158.6, 135.0, 134.9, 130.3, 129.1, 128.8, 128.0, 125.4 (q, $^1J_{CF}$ = 280.9 Hz), 114.9, 67.6 (q, $^2J_{CF}$ = 31.2 Hz), 55.5, 46.2, 28.0. **^{19}F NMR** (471 MHz, $CDCl_3$) δ -79.02 (s, 3F). **HRMS** (ESI) m/z: $[M+Na]^+$ Calcd for $C_{18}H_{18}NO_3F_3Na^+$ 376.1136; Found 376.1139.

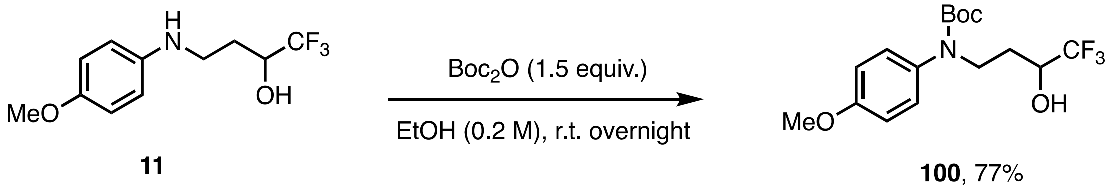

An oven-dried, transparent 10 mL round bottom flask equipped with a stir bar was sequentially charged with **11** (1.0 equiv., 0.1 mmol, 24.9 mg), Boc_2O (1.5 equiv., 0.15 mmol, 32.7 mg), and EtOH (0.5 mL, 0.2 M) at room temperature. The mixture was stirred at the same temperature for overnight. Upon completion of the reactions (TLC showed complete consumption of starting material). The reaction was extracted with EtOAc (3 × 10 mL). The combined organic layers were washed with brine, dried over anhydrous Na_2SO_4, filtered and concentrated in *vacuo*. The residue was purified by preparative thin-layer chromatography using a mixture of petroleum ether and ethyl acetate (PE:EA = 5:1) as an eluent to afford the product **100**.

***Tert*-butyl (4-methoxyphenyl)(4,4,4-trifluoro-3-hydroxybutyl)carbamate (100).** The title compound was obtained as a white solid by preparative TLC using petroleum ether/EtOAc (5:1) as an eluent; R_f= 0.4 (petroleum ether/EtOAc = 3:1); 26.9 mg, 77% yield, m.p. 101 – 102 ºC. **^{1}H NMR** (500 MHz, $CDCl_3$) δ 7.02 (d, J = 8.6 Hz, 2H), 6.87 (d, J = 8.3 Hz, 2H), 4.85 (brs, 1H), 4.15 – 4.02 (m, 2H), 3.81 (s, 3H), 3.59 – 3.32 (m, 1H), 1.87 – 1.68 (m, 2H), 1.37 (s, 9H). **^{13}C NMR** (126 MHz, $CDCl_3$) δ 158.3, 156.9, 134.6, 128.4, 125.4 (q, $^1J_{CF}$ = 280.8 Hz), 114.4, 81.2, 67.7 (q, $^2J_{CF}$ = 31.3 Hz), 55.5, 45.9, 28.8, 28.3. **^{19}F NMR** (471 MHz, $CDCl_3$) δ -79.05 (s, 3F). **HRMS** (ESI) m/z: $[M+Na]^+$ Calcd for $C_{16}H_{22}NO_4F_3Na^+$ 372.1399; Found 372.1400.

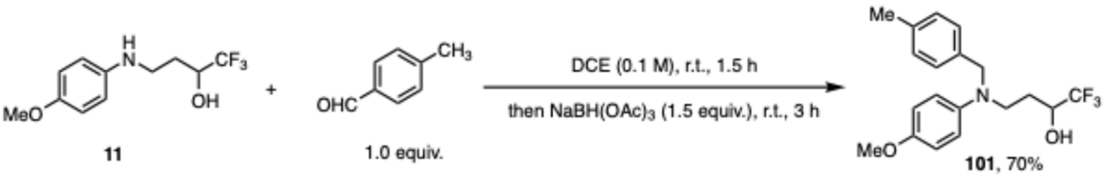

To a stirred solution of **11** (1.0 equiv., 0.1 mmol, 24.9 mg) in DCE (1 mL, 0.1 M) were added *p*-tolualdehyde (1.0 equiv., 0.1 mmol, 12.0 μL) at 0 °C. Then the mixture was allowed to warm to room temperature and stirred for 1.5 h. Then Sodium triacetoxyborohydride (1.5 equiv., 0.15 mmol, 31.8 mg) was added to the reaction mixture. Then the mixture was allowed to warm to room temperature and stirred for 3 h. Upon completion of the reactions (TLC showed complete consumption of starting material). The reaction was quenched with saturated $NaHCO_3$ (10 mL) and extracted with EtOAc (3 × 10 mL). The combined organic layers were washed with brine, dried over anhydrous Na_2SO_4, filtered and concentrated in *vacuo*. The residue was purified by preparative thin-layer chromatography using a mixture of petroleum ether and ethyl acetate (PE:EA = 5:1) as an eluent to afford the product **101.**

1,1,1-trifluoro-4-((4-methoxyphenyl)(4-methylbenzyl)amino)butan-2-ol (101). The title compound was obtained as a yellow solid by preparative TLC using petroleum ether/EtOAc (5:1) as an eluent; R_f= 0.5 (petroleum ether/EtOAc = 3:1); 24.7 mg, 70% yield, m.p. 75 – 76 ºC. **^{1}H NMR** (500 MHz, $CDCl_3$) δ 7.11 – 7.06 (m, 4H), 6.89 (d, J = 8.5 Hz, 2H), 6.82 (d, J = 8.4 Hz, 2H), 4.70 (brs, 1H), 4.28 – 4.20 (m, 2H), 4.09 – 4.05

(m, 1H), 3.77 (s, 3H), 3.46 – 3.34 (m, 2H), 2.32 (s, 3H), 1.90 – 1.77 (m, 2H). **^{13}C NMR** (126 MHz, $CDCl_3$) δ 154.6, 142.6, 137.1, 134.5, 129.3, 128.3, 125.1 (q, $^1J_{CF}$ = 281.6 Hz), 120.4, 114.8, 70.3 (q, $^2J_{CF}$ = 31.5 Hz), 59.0, 55.7, 49.4, 26.0, 21.2. **^{19}F NMR** (471 MHz, $CDCl_3$) δ -79.98 (s, 3F). **HRMS** (ESI) m/z: $[M+H]^+$ Calcd for $C_{19}H_{23}NO_2F_3^+$ 354.1675; Found 354.1676.

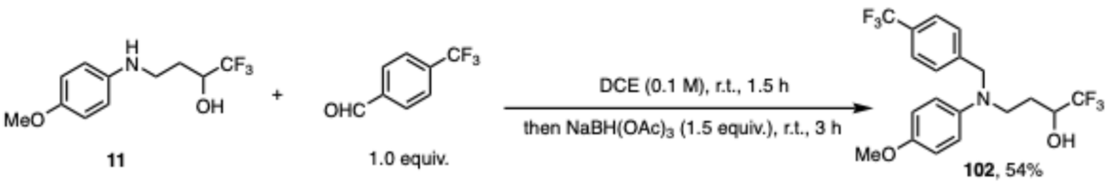

To a stirred solution of **11** (1.0 equiv., 0.1 mmol, 24.9 mg) in DCE (1 mL, 0.1 M) were added 4-(Trifluoromethyl)benzaldehyde (1.0 equiv., 0.1 mmol, 14.0 μL) at 0 °C. Then the mixture was allowed to warm to room temperature and stirred for 1.5 h. Then Sodium triacetoxyborohydride (1.5 equiv., 0.15 mmol, 31.8 mg) was added to the reaction mixture. Then the mixture was allowed to warm to room temperature and stirred for 3 h. Upon completion of the reactions (TLC showed complete consumption of starting material). The reaction was quenched with saturated $NaHCO_3$ (10 mL) and extracted with EtOAc (3 × 10 mL). The combined organic layers were washed with brine, dried over anhydrous Na_2SO_4, filtered and concentrated in *vacuo*. The residue was purified by preparative thin-layer chromatography using a mixture of petroleum ether and ethyl acetate (PE:EA = 5:1) as an eluent to afford the product **102.**

1,1,1-trifluoro-4-((4-methoxyphenyl)(4-(trifluoromethyl)benzyl)amino)butan-2-ol (102). The title compound was obtained as a brown solid by preparative TLC using petroleum ether/EtOAc (5:1) as an eluent; R_f= 0.4 (petroleum ether/EtOAc = 3:1); 22.0 mg, 54% yield, m.p. 81 – 82 ºC. **^{1}H NMR** (500 MHz, $CDCl_3$) δ 7.54 (d, J = 7.8 Hz, 2H), 7.29 (d, J = 7.9 Hz, 2H), 6.82 – 6.80 (m, 4H), 4.41 – 4.32 (m, 2H), 4.11 – 4.06 (m, 2H), 3.76 (s, 3H), 3.51 – 3.40 (m, 2H), 1.97 – 1.80 (m, 2H). **^{13}C NMR** (126 MHz, $CDCl_3$) δ 154.4, 142.1, 142.0, 129.7 (q, $^2J_{CF}$ = 32.4 Hz), 128.3, 125.6 (q, $^3J_{CF}$ = 3.8 Hz), 125.1 (q, $^1J_{CF}$ = 281.4 Hz), 124.2 (q, $^1J_{CF}$ = 271.9 Hz), 119.4, 114.9, 70.0 (q, $^2J_{CF}$ =

31.2 Hz), 58.0, 55.7, 49.7, 26.3. **^{19}F NMR** (471 MHz, $CDCl_3$) δ -62.43 (s, 3F), -79.97 (s, 3F). **HRMS** (ESI) m/z: $[M+H]^+$ Calcd for $C_{19}H_{20}NO_2F_6^+$ 408.1398; Found 408.1396.

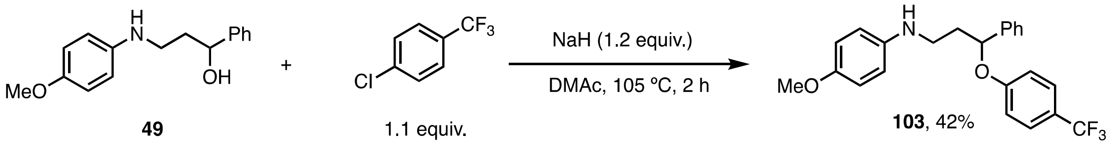

To a 10 mL round bottom flask equipped with a magnetic stirring bar was added, **49** (1.0 equiv., 0.1 mmol, 25.7 mg) was dissolved in dry DMAc (0.1 mL, 1.0 M) and cooled to 0 °C in an ice-bath. To this solution was added NaH (60% in mineral oil, 1.2 equiv., 0.12 mmol, 4.8 mg,). The mixture was heated at 90 °C for 1.5 h. To this solution was added 4-chlorobenzotrifluoride (1.1 equiv., 0.11 mmol, 15.0 μL), and the mixture was heated at 105°C for 2 h. The reaction was extracted with EtOAc (3 × 10 mL). The combined organic layers were washed with brine, dried over anhydrous Na_2SO_4, filtered and concentrated in *vacuo*. The residue was purified by preparative thin-layer chromatography using a mixture of petroleum ether and ethyl acetate (PE:EA = 5:1) as an eluent to afford the product **103**.

4-methoxy-*N*-(3-phenyl-3-(4-(trifluoromethyl)phenoxy)propyl)aniline (103). The title compound was obtained as a yellow oil by preparative TLC using petroleum ether/EtOAc (5:1) as an eluent; R_f= 0.6 (petroleum ether/EtOAc = 3:1); 16.8 mg, 42% yield. **^{1}H NMR** (500 MHz, $CDCl_3$) δ 7.43 (d, J = 8.2 Hz, 2H), 7.34 – 7.25 (m, 5H), 6.90 (d, J = 8.4 Hz, 2H), 6.76 (d, J = 8.5 Hz, 2H), 6.56 (d, J = 8.4 Hz, 2H), 5.34 – 5.29 (m, 1H), 3.74 (s, 3H), 3.33 – 3.23 (m, 2H), 2.33 – 2.11 (m, 2H). (Due to the broadening effect and intermolecular hydrogen bonding, one of the proton signals of NH could not be observed). **^{13}C NMR** (126 MHz, $CDCl_3$) δ 160.5, 152.3, 142.4, 140.8, 129.0, 128.1, 126.9 (q, $^3J_{CF}$ = 3.8 Hz), 126.6 (q, $^1J_{CF}$ = 271.5 Hz), 125.9, 123.0 (q, $^2J_{CF}$ = 32.7 Hz), 115.9, 115.0, 114.3, 78.7, 55.9, 41.6, 38.6. **^{19}F NMR** (471 MHz, $CDCl_3$) δ -61.51 (s, 3F). **HRMS** (ESI) m/z: $[M+H]^+$ Calcd for $C_{23}H_{23}NO_2F_3^+$ 402.1681; Found 402.1682.

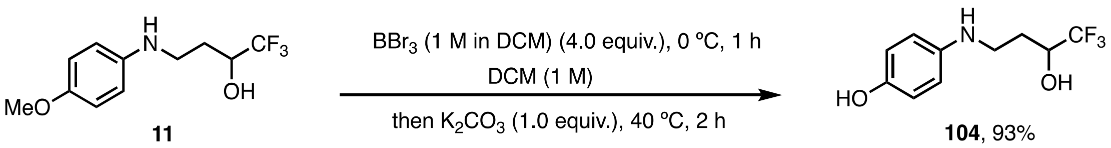

To a 10 mL round bottom flask equipped with a magnetic stirring bar was added, BBr_3 (4.0 equiv., 1 M in DCM, 0.4 mL) was added dropwise to a solution of **11** (1.0 equiv., 0.1 mmol, 24.9 mg) in dichloromethane (0.1 mL, 1 M) at 0 °C. The mixture was stirred at 0 °C for 1 h. Upon completion of the reactions (TLC showed complete consumption of starting material). K_2CO_3 (1.0 equiv., 0.1 mmol, 27.64 mg) was added to the reaction mixture. Then the mixture was allowed to warm to 40 °C and stirred for 2 h. The reaction was extracted with DCM (3 × 10 mL). The combined organic layers were washed with brine, dried over anhydrous $MgSO_4$, filtered and concentrated in *vacuo*. The residue was purified by preparative thin-layer chromatography using a mixture of petroleum ether and ethyl acetate (PE:EA = 2:1) as an eluent to afford the product **104**.

4-((4,4,4-trifluoro-3-hydroxybutyl)amino)phenol (104). The title compound was obtained as a brown solid by preparative TLC using petroleum ether/EtOAc (2:1) as an eluent; R_f = 0.2 (petroleum ether/EtOAc = 1:1); 21.9 mg, 93% yield, m.p. 71 – 72 °C. **^{1}H NMR** (500 MHz, DMSO-*d*6) δ 8.42 (brs, 1H), 6.55 (d, J = 8.1 Hz, 2H), 6.44 (d, J = 8.3 Hz, 2H), 6.19 (brs, 1H), 4.96 (brs, 1H), 4.16 – 3.95 (m, 1H), 3.08 – 3.07 (m, 2H), 1.84 – 1.62 (m, 2H). **^{13}C NMR** (126 MHz, DMSO-*d*6) δ 148.4, 141.7, 126.1 (q, $^1J_{CF}$ = 282.9 Hz), 115.7, 113.5, 66.5 (q, $^2J_{CF}$ = 29.9 Hz), 39.6, 29.2. **^{19}F NMR** (471 MHz, DMSO-*d*6) δ -78.14 (s, 3F). **HRMS** (ESI) m/z: $[M+H]^+$ Calcd for $C_{10}H_{13}NO_2F_3^+$ 236.0898; Found 236.0899.

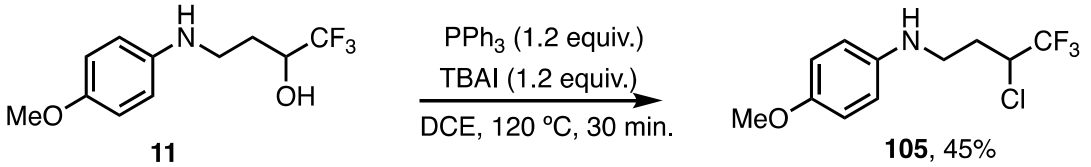

An oven-dried, transparent 10 mL Teflon screw-capped Schlenk tube equipped with a stir bar was sequentially charged with **11** (1.0 equiv., 0.1 mmol, 24.9 mg), PPh_3 (1.2 equiv., 0.12 mmol, 31.5 mg), and TBAI (1.2 equiv., 0.12 mmol, 44.3 mg). The tube was evacuated *in vacuo* and then backfilled with argon for three times. Dry DCE (1.0

mL, 0.1 M). was transferred into the tube via a syringe. The resulting mixture was stirred under an argon atmosphere was stirred at 120 °C for 30 min. Upon completion of the reactions (TLC showed complete consumption of starting material). The mixture was concentrated in *vacuo*. The residue was purified by preparative thin-layer chromatography using a mixture of petroleum ether and ethyl acetate (PE:EA = 5:1) as an eluent to afford the product **105**.

***N*-(3-chloro-4,4,4-trifluorobutyl)-4-methoxyaniline (105).** The title compound was obtained as a yellow oil by preparative TLC using petroleum ether/EtOAc (5:1) as an eluent; R_f= 0.5 (petroleum ether/EtOAc = 3:1); 12.1 mg, 45% yield. **^{1}H NMR** (500 MHz, $CDCl_3$) δ 6.80 (d, J = 8.6 Hz, 2H), 6.61 (d, J = 8.3 Hz, 2H), 4.37 – 4.30 (m, 1H), 3.75 (s, 3H), 3.57 – 3.33 (m, 3H), 2.34 – 2.27 (m, 1H), 2.05 – 1.98 (m, 1H). **^{13}C NMR** (126 MHz, $CDCl_3$) δ 152.7, 141.6, 124.3 (q, $^1J_{CF}$ = 278.3 Hz), 115.1, 114.5, 55.9, 55.3 (q, $^2J_{CF}$ = 33.3 Hz), 40.9, 31.2. **^{19}F NMR** (471 MHz, $CDCl_3$) δ -74.60 (s, 3F). **HRMS** (ESI) m/z: $[M+H]^+$ Calcd for $C_{11}H_{14}NOClF_3^+$ 268.0716; Found 268.0715.

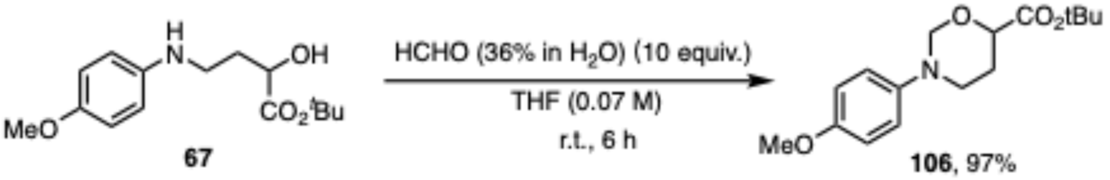

To a 10 mL round bottom flask equipped with a magnetic stirring bar was added, HCHO (36% in H_2O, 10.0 equiv., 1 mmol, 30.0 μL)) was added to a solution of **67** (1.0 equiv., 0.1 mmol, 28.1 mg) in THF (1.5 mL, 0.07 M). The mixture was stirred at room temperature for 6 h. Upon completion of the reactions (TLC showed complete consumption of starting material). The reaction was concentrated in *vacuo*. The residue was purified by preparative thin-layer chromatography using a mixture of petroleum ether and ethyl acetate (PE:EA = 15:1) as an eluent to afford the product **106**.

***Tert*-butyl 3-(4-methoxyphenyl)-1,3-oxazinane-6-carboxylate (106)**. The title compound was obtained as a colourless crystalline solid by preparative TLC using petroleum ether/EtOAc (15:1) as an eluent; R_f= 0.3 (petroleum ether/EtOAc = 10:1); 28.3 mg, 97% yield, m.p. 47 – 48 ºC. **^{1}H NMR** (500 MHz, $CDCl_3$) δ 7.08 (d, J = 8.3

Hz, 2H), 6.82 (d, J = 8.4 Hz, 2H), 5.17 (d, J = 10.7 Hz, 1H), 4.67 (d, J = 10.7 Hz, 1H), 4.19 (d, J = 10.4 Hz, 1H), 3.76 (s, 3H), 3.64 (d, J = 13.7 Hz, 1H), 3.44 – 3.39 (m, 1H), 1.93– 1.86 (m, 1H), 1.77 – 1.74 (m, 1H), 1.47 (s, 9H). **^{13}C NMR** (126 MHz, $CDCl_3$) δ 170.1, 154.6, 142.5, 121.1, 114.5, 81.7, 81.6, 75.9, 55.6, 50.4, 28.1, 25.7. **HRMS** (ESI) m/z: $[M+H]^+$ Calcd for $C_{16}H_{24}NO_4^+$ 294.1705; Found 294.1706.

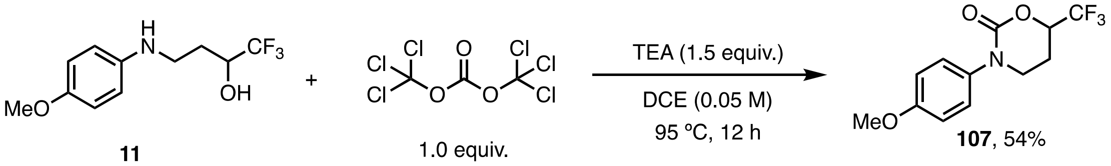

An oven-dried, transparent 10 mL Teflon screw-capped Schlenk tube equipped with a stir bar was sequentially charged with **11** (1.0 equiv., 0.1 mmol, 24.9 mg) and triphosgene (1.0 equiv., 0.1 mmol, 29.7 mg). The tube was evacuated *in vacuo* and then backfilled with argon for three times. Anhydrous DCE (2.0 mL, 0.05 M) was transferred into the tube via syringe. Subsequently, triethylamine (1.5 equiv., 0.15 mmol, 21.0 μL) was transferred into the tube via syringe. The resulting mixture was stirred under an argon atmosphere was stirred at 95 °C for 12 h. Upon completion of the reactions (TLC showed complete consumption of starting material). The reaction was extracted with DCM (3 × 10 mL). The combined organic layers were washed with brine, dried over anhydrous $MgSO_4$, filtered and concentrated in *vacuo*. The residue was purified by preparative thin-layer chromatography using a mixture of petroleum ether and ethyl acetate (PE:EA = 15:1) as an eluent to afford the product **107**.

3-(4-methoxyphenyl)-6-(trifluoromethyl)-1,3-oxazinan-2-one (107). The title compound was obtained as a brown solid by preparative TLC using petroleum ether/EtOAc (15:1) as an eluent; R_f= 0.3 (petroleum ether/EtOAc = 10:1); 14.9 mg, 54% yield, m.p. 143 – 144 ºC. **^{1}H NMR** (500 MHz, $CDCl_3$) δ 7.22 (d, J = 8.3 Hz, 2H), 6.92 (d, J = 8.3 Hz, 2H), 4.79 – 4.75 (m, 1H), 3.84 – 3.70 (m, 5H), 2.36 – 2.30 (m, 2H). **^{13}C NMR** (126 MHz, $CDCl_3$) δ 158.8, 150.7, 135.0, 127.2, 122.8 (q, $^1J_{CF}$ = 280.3 Hz), 114.8, 74.1 (q, $^2J_{CF}$ = 34.3 Hz), 55.6, 47.1, 21.4. **^{19}F NMR** (471 MHz, $CDCl_3$) δ -78.62 (s, 3F). **HRMS** (ESI) m/z: $[M+Na]^+$ Calcd for $C_{12}H_{12}NO_3F_3Na^+$ 298.0667; Found 298.0669.

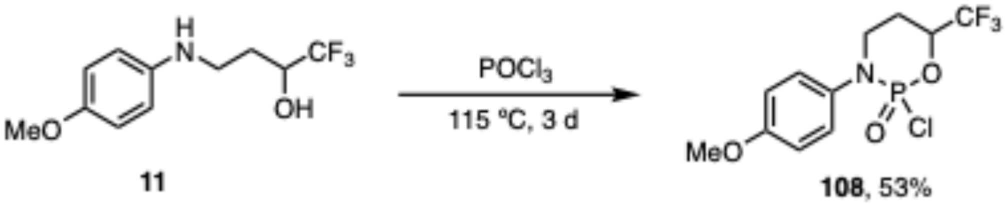

11

108, 53%

An oven-dried, transparent 20 mL Teflon screw-capped Schlenk tube equipped with a stir bar was sequentially charged with **11** (0.1 mmol, 24.9 mg) was dissolved in 3 mL of $POCl_3$ and the mixture was heated at 115 °C for 3 d. Upon completion of the reactions (TLC showed complete consumption of starting material). The mixture was concentrated in *vacuo*. The residue was dissolved in $CHCl_3$ and poured into a 10% (v/v) aqueous ammoniacal solution, and the biphasic system was stirred at room temperature for 1 h. The organic layer was separated and the aqueous layer extracted with Et_2O (3 × 10 mL). The combined organic layers were washed with brine, dried over anhydrous Na_2SO_4, filtered and concentrated in *vacuo*. The residue was purified by preparative thin-layer chromatography using a mixture of petroleum ether and ethyl acetate (PE:EA = 15:1) as an eluent to afford the product **108**.

2-chloro-3-(4-methoxyphenyl)-6-(trifluoromethyl)-1,3,2-oxazaphosphinane 2-oxide (108). The title compound was obtained as a colourless crystalline solid by preparative TLC using petroleum ether/EtOAc (15:1) as an eluent; R_f = 0.4 (petroleum ether/EtOAc = 10:1); 17.4 mg, 53% yield, m.p. 119 – 120 ºC. **^{1}H NMR** (500 MHz, $CDCl_3$) δ 7.25 (d, J = 8.6 Hz, 2H), 6.90 (d, J = 8.3 Hz, 2H), 4.83 – 4.80 (m, 1H), 3.83 – 3.78 (m, 4H), 3.55 – 3.47 (m, 1H), 2.44 – 2.17 (m, 2H). **^{13}C NMR** (126 MHz, $CDCl_3$) δ 158.8 (d, J = 2.3 Hz), 133.1, 126.6 (d, J = 4.2 Hz), 122.1 (qd, $^1J_{CF}$ = 279.7, 15.9 Hz), 114.9 (d, J = 2.0 Hz), 77.4 (q, $^2J_{CF}$ = 35.6 Hz), 55.6, 49.1 (d, J = 2.1 Hz), 25.2. **^{19}F NMR** (471 MHz, $CDCl_3$) δ -79.04 (s, 3F). **^{31}P NMR** (202 MHz, $CDCl_3$) δ 4.63 (d, J = 31.3 Hz). **HRMS** (ESI) m/z: $[M+H]^+$ Calcd for $C_{11}H_{13}NO_3F_3ClP^+$ 330.0274; Found 330.0274.

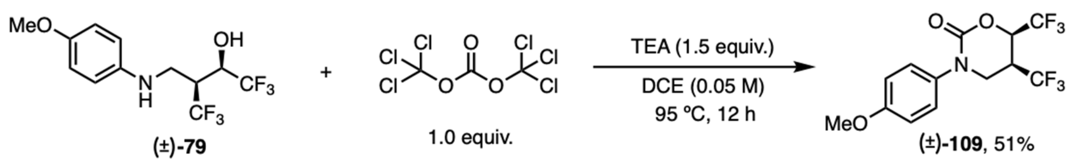

An oven-dried, transparent 10 mL Teflon screw-capped Schlenk tube equipped with a stir bar was sequentially charged with **(±)-79** (1.0 equiv., 0.1 mmol, 31.7 mg) and triphosgene (1.0 equiv., 0.1 mmol, 29.7 mg). The tube was evacuated *in vacuo* and then backfilled with argon for three times. Anhydrous DCE (2.0 mL, 0.05 M) was transferred into the tube via syringe. Subsequently, triethylamine (1.5 equiv., 0.15 mmol, 21.0 μL) was transferred into the tube via syringe. The resulting mixture was stirred under an argon atmosphere was stirred at 95 °C for 12 h. Upon completion of the reactions (TLC showed complete consumption of starting material). The reaction was extracted with DCM (3 × 10 mL). The combined organic layers were washed with brine, dried over anhydrous $MgSO_4$, filtered and concentrated in *vacuo*. The residue was purified by preparative thin-layer chromatography using a mixture of petroleum ether and ethyl acetate (PE:EA = 15:1) as an eluent to afford the product **(±)-109**.

3-(4-methoxyphenyl)-5,6-bis(trifluoromethyl)-1,3-oxazinan-2-one ((±)-109) The title compound was obtained as a colourless crystalline solid by preparative TLC using petroleum ether/EtOAc (15:1) as an eluent; R_f = 0.3 (petroleum ether/EtOAc = 10:1); 17.5 mg, 51% yield, m.p. 141 – 143 ºC. **^{1}H NMR** (500 MHz, $CDCl_3$) δ 7.20 – 7.18 (m, 2H), 6.95 – 6.92 (m, 2H), 5.05 – 4.95 (m, 1H), 3.96 – 3.95 (m, 2H), 3.81 (s, 3H), 3.37 – 3.25 (m, 1H). **^{13}C NMR** (126 MHz, $CDCl_3$) δ 159.3, 149.3, 134.0, 127.5, 123.9 (q, $^1J_{CF}$ = 280.3 Hz), 121.9 (q, $^1J_{CF}$ = 282.0 Hz). 115.1, 72.6 (q, $^2J_{CF}$ = 35.4 Hz), 55.7, 47.1, 37.1 (q, $^2J_{CF}$ = 30.3 Hz). **^{19}F NMR** (471 MHz, $CDCl_3$) δ -65.6 (q, J = 8.7 Hz, 3F), -74.0 (q, J = 8.3 Hz, 3F). **HRMS** (ESI) m/z: $[M+Na]^+$ Calcd for $C_{13}H_{12}NO_3F_6^+$ 344.0721; Found 344.0723.

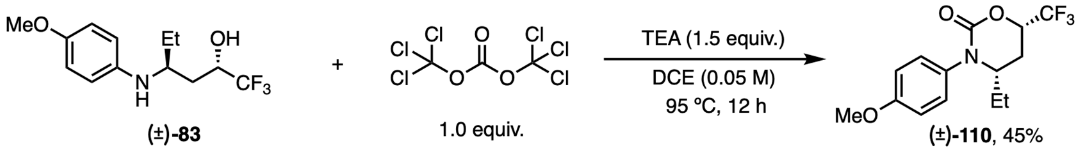

An oven-dried, transparent 10 mL Teflon screw-capped Schlenk tube equipped with a stir bar was sequentially charged with **(±)-83** (1.0 equiv., 0.1 mmol, 27.7 mg) and triphosgene (1.0 equiv., 0.1 mmol, 29.7 mg). The tube was evacuated *in vacuo* and then backfilled with argon for three times. Anhydrous DCE (2.0 mL, 0.05 M) was transferred into the tube via syringe. Subsequently, triethylamine (1.5 equiv., 0.15 mmol, 21.0 μL) was transferred into the tube via syringe. The resulting mixture was stirred under an argon atmosphere was stirred at 95 ^{o}C for 12 h. Upon completion of the reactions (TLC showed complete consumption of starting material). The reaction was extracted with DCM (3 × 10 mL). The combined organic layers were washed with brine, dried over anhydrous $MgSO_4$, filtered and concentrated in *vacuo*. The residue was purified by preparative thin-layer chromatography using a mixture of petroleum ether and ethyl acetate (PE:EA = 20:1) as an eluent to afford the product **(±)-110**.

4-ethyl-3-(4-methoxyphenyl)-6-(trifluoromethyl)-1,3-oxazinan-2-one ((±)-110). The title compound was obtained as a colourless crystalline solid by preparative TLC using petroleum ether/EtOAc (20:1) as an eluent; R_f = 0.3 (petroleum ether/EtOAc = 15:1); 13.6 mg, 45% yield, m.p. 103 – 105 ºC. **^{1}H NMR** (500 MHz, $CDCl_3$) δ 7.17 – 7.14 (m, 2H), 6.94 – 6.92 (m, 2H), 4.79 – 4.71 (m, 1H), 3.86 – 3.75 (m, 4H), 2.42 – 2.30 (m, 1H), 2.02 – 1.94 (m, 1H), 1.65 – 1.48 (m, 2H), 0.86 – 0.82 (m, 3H). **^{13}C NMR** (126 MHz, $CDCl_3$) δ 159.0, 151.5, 131.7, 129.2, 122.6 (q, $^1J_{CF}$ = 279.4 Hz), 114.7, 72.8 (q, $^2J_{CF}$ = 34.4 Hz), 57.6, 55.6, 38.3, 27.2, 8.9. **^{19}F NMR** (471 MHz, $CDCl_3$) δ -79.01 (s, 3F). **HRMS** (ESI) m/z: $[M+Na]^+$ Calcd for $C_{14}H_{16}NO_3F_3Na^+$ 326.0980; Found 326.0981.

Stern-Volmer quenching studies

Stern-Volmer quenching experiments were carried using a solution of photocatalyst 4CzPN (**PC4**, 0.10 mM) and variable concentrations (0.05, 0.10, 0.15, 0.20, 0.25 mM) of Hantzsch ester (**HE**), *N*,*N*-dimethylcyclohexylamine (**CyNMe$_2$**, ***A1***), 1-methoxy-4-nitrobenzene (***p*-MeOPhNO$_2$**, ***N1***), *tert*-butyl acrylate (**alkene**, ***O21***), and **Ni(Bipy)Cl$_2$** in NMP. The standard solutions of samples were prepared in the nitrogen-filled glovebox. 3 mL of sample solutions were transferred via syringe into the PTFE-stopped, parafilm-sealed quartz cuvettes (3.5 mL, 10 mm) under the positive argon pressure prior to the measurement. The intensity of the emission peak at 597 nm (λ_{ex} = 382 nm), which was expressed as the ratio I_0/I (I_0: emission intensity of photocatalyst **PC4** at 597 nm in the absence of quencher; I: observed intensity) as a function of the quencher concentration, was measured. The Stern-Volmer plots for each component are provided in the **Figs. S11–S16**. The results suggested that Hantzsch ester (**HE**), **CyNMe$_2$** (***A1***), 1-methoxy-4-nitrobenzene (***p*-MeOPhNO$_2$**, ***N1***), **alkene** (***O21***), and **Ni(Bipy)Cl$_2$** were all able to quench the photoexcited **PC4**. Among them, Hantzsch ester (**HE**) is the most effective quencher to initiate the subsequent photocatalytic events.

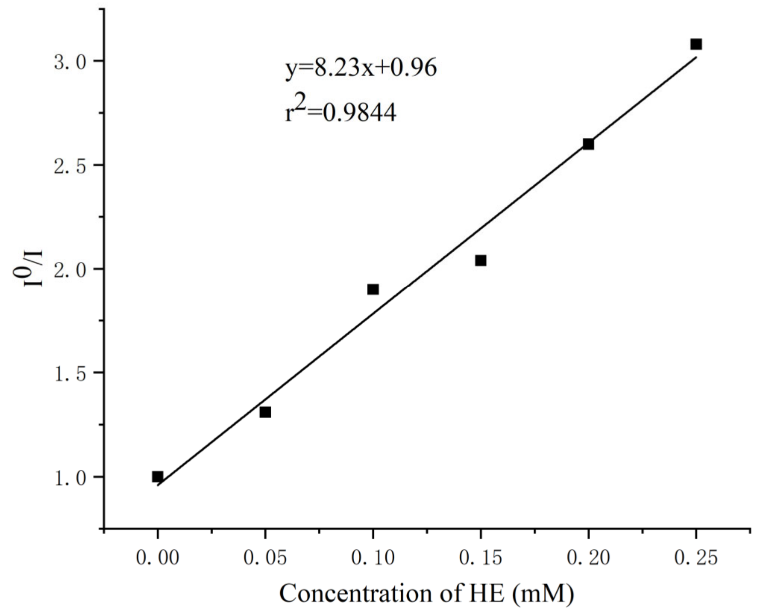

Fig. S11. Stern-Volmer fluorescence quenching studies of photocatalyst **PC4** (0.1 mM) with varying concentrations of Hantzsch ester (**HE**).

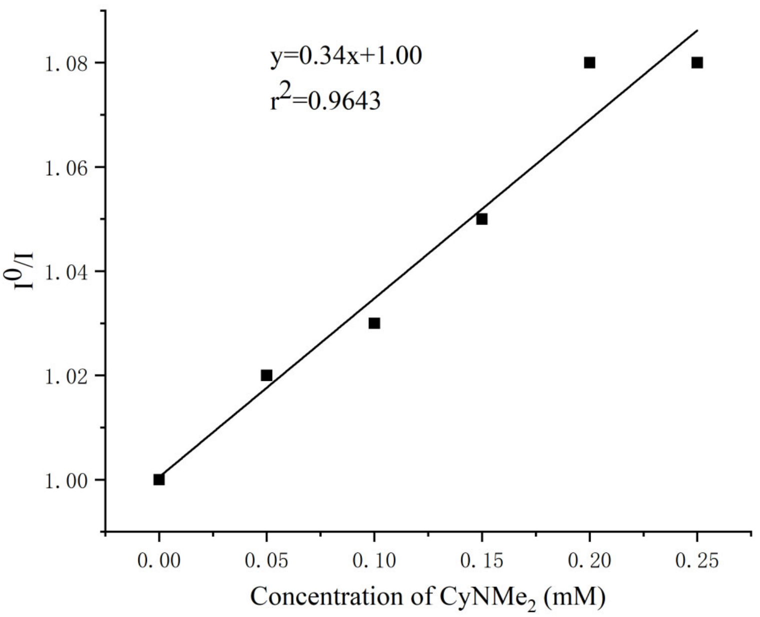

Fig. S12. Stern-Volmer fluorescence quenching studies of photocatalyst **PC4** (0.1 mM) with varying concentrations of *N*,*N*-dimethylcyclohexylamine (**CyNMe$_2$**, *A1*).

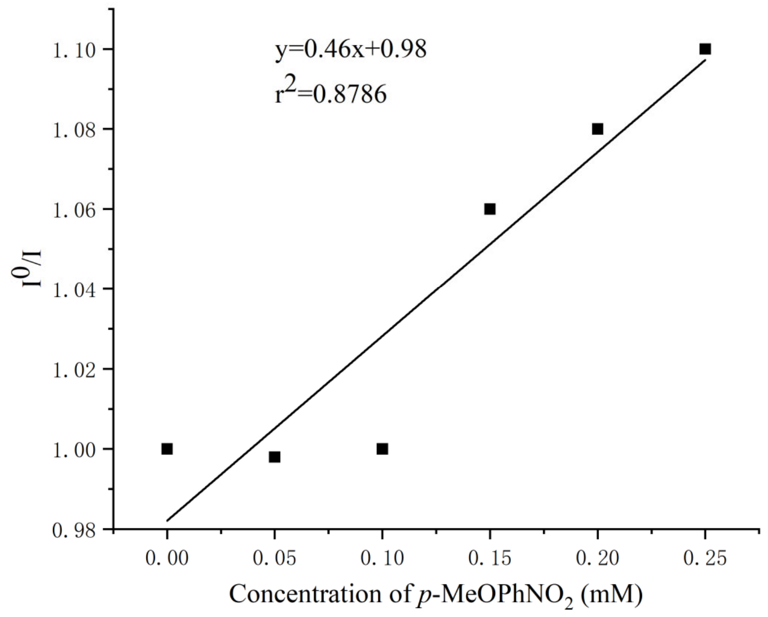

Fig. S13. Stern-Volmer fluorescence quenching studies of photocatalyst **PC4** (0.1 mM) with varying concentrations of 1-methoxy-4-nitrobenzene (***p*-MeOPhNO$_2$**, ***N1***).

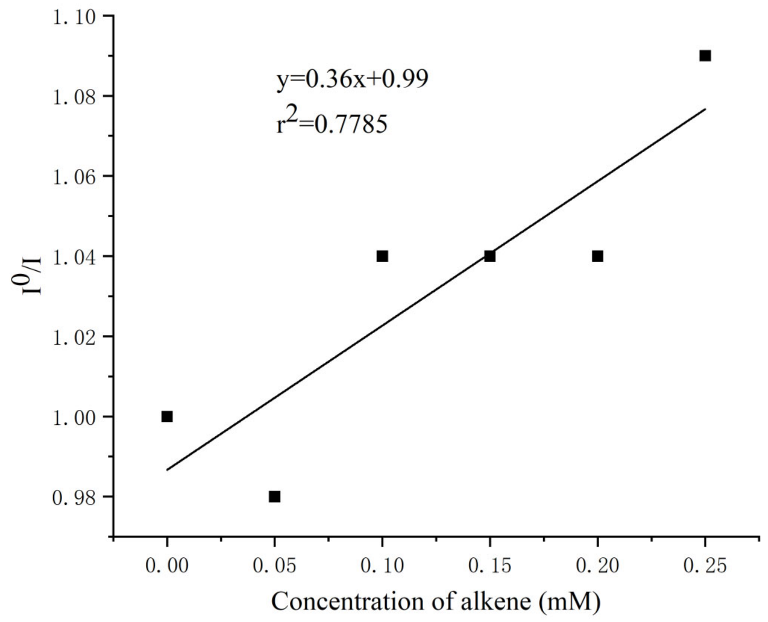

Fig. S14. Stern-Volmer fluorescence quenching studies of photocatalyst **PC4** (0.1 mM) with varying concentrations of *tert*-butyl acrylate (**alkene**, ***O21***).

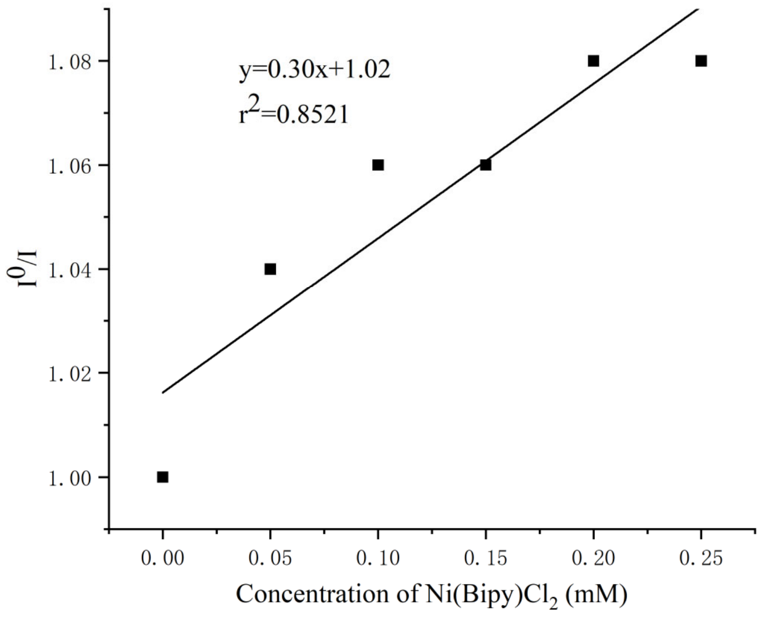

Fig. S15. Stern-Volmer fluorescence quenching studies of photocatalyst **PC4** (0.1 mM) with varying concentrations of **Ni(Bipy)Cl$_2$**.

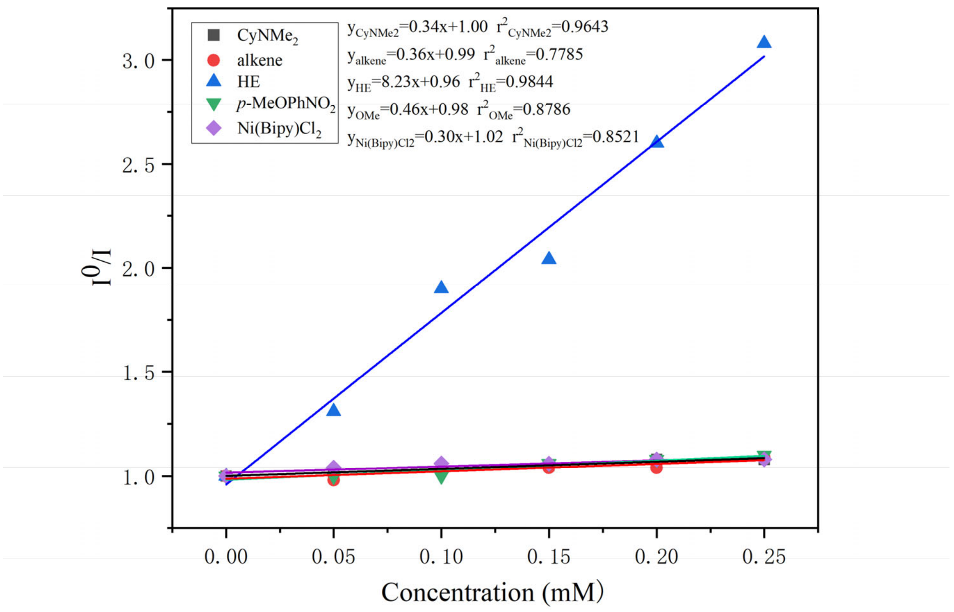

Fig. S16. Stern-Volmer fluorescence quenching studies of photocatalyst **PC4** (0. 1 mM) with varying concentrations of redox active ester Hantzsch ester (**HE**), *N*,*N*-dimethylcyclohexylamine (**CyNMe$_2$**, ***A1***), 1-methoxy-4-nitrobenzene (***p*-MeOPhNO$_2$**, ***N1***), *tert*-butyl acrylate (**alkene**, ***O21***), and **Ni(Bipy)Cl$_2$**.

X-ray crystallographic analysis

The single crystal of 3-arylamino alcohol product **26** was obtained by slow evaporation of the solvent system based on CH_2Cl_2 and cyclohexane. The ORTEP representation with 50% probability thermal ellipsoids were presented. Crystallographic data for the compound **26** has been deposited at the Cambridge Crystallographic Data Centre, under deposition numbers CCDC 2426457 (**26**). Copies of the data can be obtained free of charge via https://www.ccdc.cam.ac.uk/structures/.

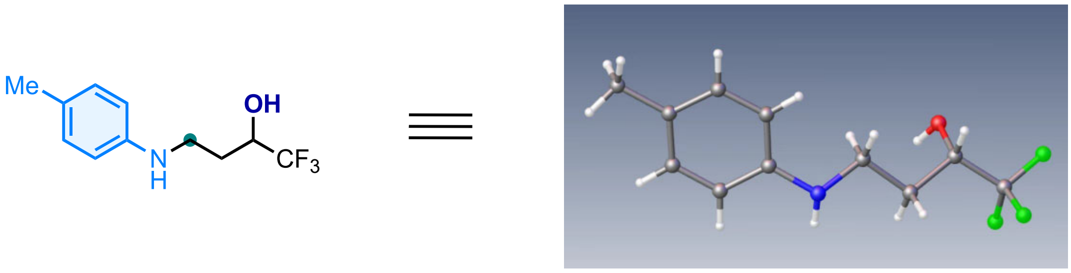

Empirical formula	$C_{11}H_{14}F_3NO$
Formula weight	233.23
Temperature/K	170
Crystal system	monoclinic
Space group	Cc
a/Å	25.055(9)
b/Å	5.511(2)
c/Å	8.319(3)
α/°	90
β/°	102.14(2)
γ/°	90
Volume/Å^3	1123.0(7)
Z	4
ϱ_{calc}g/cm^3	1.379
μ/mm^{-1}	0.122
F(000)	488.0
Crystal size/mm^3	0.12 × 0.05 × 0.03
Radiation	MoKα (λ = 0.71073)
2Θ range for data collection/°	6.654 to 52.972
Index ranges	-31 ≤ h ≤ 30, -6 ≤ k ≤ 6, -10 ≤ l ≤ 10
Reflections collected	3615
Independent reflections	1836 [R_{int} = 0.0921, R_{sigma} = 0.1044]
Data/restraints/parameters	1836/2/147
Goodness-of-fit on F^2	1.113
Final R indexes [I>=2σ (I)]	R_1 = 0.0913, wR_2 = 0.2578
Final R indexes [all data]	R_1 = 0.1196, wR_2 = 0.2881
Largest diff. peak/hole / e Å^{-3}	0.48/-0.40
Flack parameter	-0.5(10)

The single crystal of 3-arylamino alcohol product **49** was obtained by slow evaporation of the solvent system based on CH_2Cl_2 and cyclohexane. The ORTEP representation with 50% probability thermal ellipsoids were presented. Crystallographic data for the compound **49** has been deposited at the Cambridge Crystallographic Data Centre, under deposition numbers CCDC 2426464 (**49**). Copies of the data can be obtained free of charge via https://www.ccdc.cam.ac.uk/structures/.

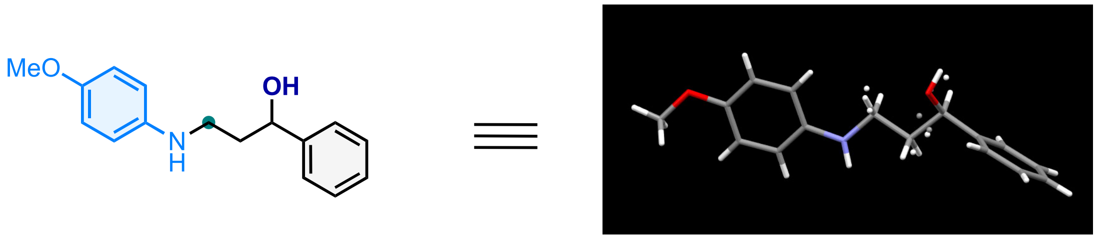

Empirical formula	$C_{16}H_{19}NO_2$
Formula weight	257.32
Temperature/K	170
Crystal system	monoclinic
Space group	$P2_1/c$
a/Å	15.4884(18)
b/Å	7.8257(9)
c/Å	11.7108(16)
α/°	90
β/°	107.184(4)
γ/°	90
Volume/Å^3	1356.1(3)
Z	4
ϱ_{calc}g/cm^3	1.260
μ/mm^{-1}	0.083
F(000)	552.0
Crystal size/mm^3	0.15 × 0.06 × 0.05
Radiation	MoKα (λ = 0.71073)
2Θ range for data collection/°	5.506 to 52.74
Index ranges	$-19 \le h \le 19, -9 \le k \le 9, -13 \le l \le 14$
Reflections collected	11474
Independent reflections	2733 [R_{int} = 0.0800, R_{sigma} = 0.0634]
Data/restraints/parameters	2733/0/192
Goodness-of-fit on F^2	1.028
Final R indexes [I>=2σ (I)]	R_1 = 0.0613, wR_2 = 0.1439
Final R indexes [all data]	R_1 = 0.0947, wR_2 = 0.1675
Largest diff. peak/hole / e Å^{-3}	0.45/-0.47

The single crystal of 3-arylamino alcohol product **71** was obtained by slow evaporation of the solvent system based on CH_2Cl_2 and cyclohexane. The ORTEP representation with 50% probability thermal ellipsoids were presented. A disordered X-ray structure was obtained. Crystallographic data for the compound **71** has been deposited at the Cambridge Crystallographic Data Centre, under deposition numbers CCDC 2426459 (**71**). Copies of the data can be obtained free of charge via https://www.ccdc.cam.ac.uk/structures/.

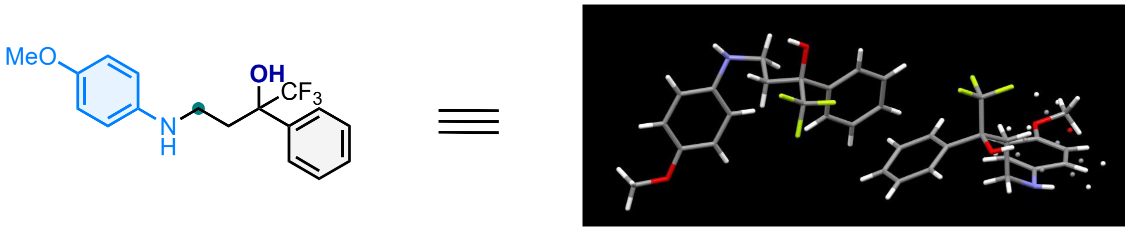

Empirical formula	$C_{17}H_{18}F_3NO_2$
Formula weight	325.32
Temperature/K	110
Crystal system	orthorhombic
Space group	Pbca
a/Å	20.015(3)
b/Å	9.7127(14)
c/Å	32.352(5)
α/°	90
β/°	90
γ/°	90
Volume/Å^3	6289.2(16)
Z	16
ϱ_{calc}g/cm^3	1.374
μ/mm^{-1}	0.113
F(000)	2720.0
Crystal size/mm^3	0.15 × 0.09 × 0.08
Radiation	MoKα (λ = 0.71073)
2Θ range for data collection/°	4.07 to 55.112
Index ranges	$-26 \le h \le 24$, $-12 \le k \le 12$, $-39 \le l \le 39$
Reflections collected	49765
Independent reflections	7099 [R_{int} = 0.1065, R_{sigma} = 0.0614]
Data/restraints/parameters	7099/170/496
Goodness-of-fit on F^2	1.022
Final R indexes [I>=2σ (I)]	R_1 = 0.0527, wR_2 = 0.1059
Final R indexes [all data]	R_1 = 0.1052, wR_2 = 0.1316
Largest diff. peak/hole / e Å^{-3}	0.21/-0.20

The single crystal of 3-arylamino alcohol product **(±)-86** was obtained by slow evaporation of the solvent system based on CH_2Cl_2 and cyclohexane. The ORTEP representation with 50% probability thermal ellipsoids were presented. The crystallographic structures of both enantiomers of **(±)-86** were determined from a single crystal. Crystallographic data for the compound **(±)-86** has been deposited at the Cambridge Crystallographic Data Centre, under deposition numbers CCDC 2464714 ((±)-**86**). Copies of the data can be obtained free of charge via https://www.ccdc.cam.ac.uk/structures/.

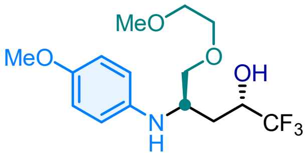

(±)-86

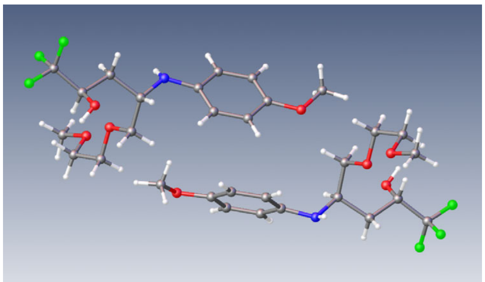

Empirical formula	$C_{15}H_{22}F_3NO_4$
Formula weight	337.33
Temperature/K	170
Crystal system	triclinic
Space group	P-1
a/Å	6.1353(5)
b/Å	7.4394(6)
c/Å	36.232(3)
α/°	89.159(3)
β/°	88.160(3)
γ/°	85.751(3)
Volume/Å^3	1648.2(2)
Z	4
ϱ_{calc}g/cm^3	1.359
μ/mm^{-1}	0.119
F(000)	712.0
Crystal size/mm^3	0.15 × 0.06 × 0.05
Radiation	MoKα (λ = 0.71073)
2Θ range for data collection/°	4.5 to 52.87
Index ranges	$-7 \leq h \leq 7, -9 \leq k \leq 9, -45 \leq l \leq 45$
Reflections collected	19244
Independent reflections	6776 [R_{int} = 0.0714, R_{sigma} = 0.0801]
Data/restraints/parameters	6776/2/429
Goodness-of-fit on F^2	1.065
Final R indexes [I>=2σ (I)]	R_1 = 0.0625, wR_2 = 0.1258
Final R indexes [all data]	R_1 = 0.1102, wR_2 = 0.1524
Largest diff. peak/hole / e Å^{-3}	0.22/-0.22

The single crystal of 1,3-oxazinane product **106** was obtained by slow evaporation of the solvent system based on CH_2Cl_2 and cyclohexane. The ORTEP representation with 50% probability thermal ellipsoids were presented. Crystallographic data for the compound **106** has been deposited at the Cambridge Crystallographic Data Centre, under deposition numbers CCDC 2426465 (**106**). Copies of the data can be obtained free of charge via https://www.ccdc.cam.ac.uk/structures/.

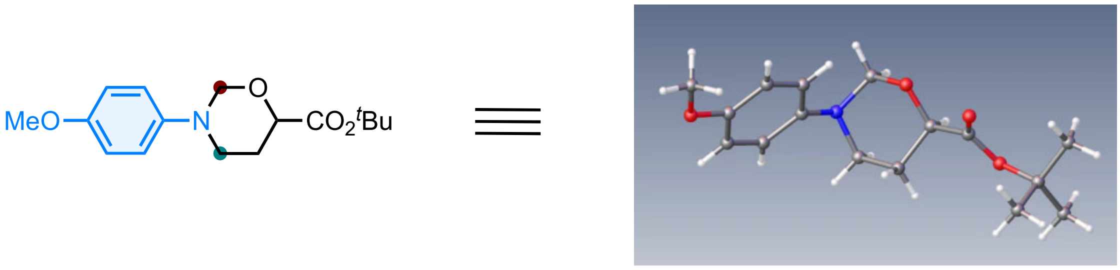

Empirical formula	$C_{16}H_{23}NO_4$
Formula weight	293.35
Temperature/K	170
Crystal system	orthorhombic
Space group	$Pca2_1$
a/Å	11.8715(5)
b/Å	14.2673(5)
c/Å	9.2905(3)
α/°	90
β/°	90
γ/°	90
Volume/Å^3	1573.57(10)
Z	4
ϱ_{calc}g/cm^3	1.238
μ/mm^{-1}	0.088
F(000)	632.0
Crystal size/mm^3	0.12 × 0.06 × 0.04
Radiation	MoKα (λ = 0.71073)
2Θ range for data collection/°	4.464 to 52.744
Index ranges	-14 ≤ h ≤ 11, -17 ≤ k ≤ 17, -11 ≤ l ≤ 11
Reflections collected	8112
Independent reflections	3096 [R_{int} = 0.0584, R_{sigma} = 0.0664]
Data/restraints/parameters	3096/1/194
Goodness-of-fit on F^2	1.042
Final R indexes [I>=2σ (I)]	R_1 = 0.0440, wR_2 = 0.0964
Final R indexes [all data]	R_1 = 0.0558, wR_2 = 0.1046
Largest diff. peak/hole / e Å^{-3}	0.14/-0.17
Flack parameter	0.2(10)

The single crystal of 1,3,2-oxazaphosphinane 2-oxide product **108** was obtained by slow evaporation of the solvent system based on CH_2Cl_2 and cyclohexane. The ORTEP representation with 50% probability thermal ellipsoids were presented. Crystallographic data for the compound **108** has been deposited at the Cambridge Crystallographic Data Centre, under deposition numbers CCDC 2426466 (**108**). Copies of the data can be obtained free of charge via https://www.ccdc.cam.ac.uk/structures/.

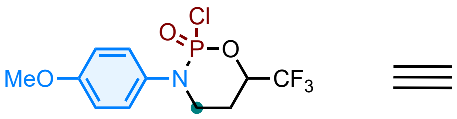

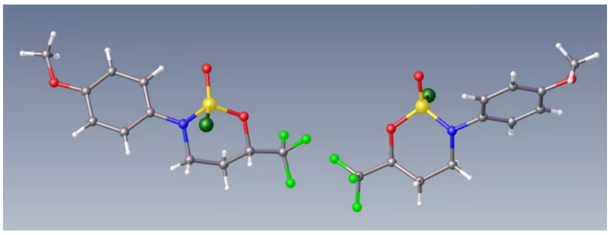

Empirical formula	$C_{11}H_{12}ClF_3NO_3P$
Formula weight	329.64
Temperature/K	170
Crystal system	triclinic
Space group	P-1
a/Å	9.927(3)
b/Å	11.691(3)
c/Å	13.070(4)
α/°	70.427(9)
β/°	78.287(9)
γ/°	89.811(9)
Volume/Å^3	1396.0(7)
Z	4
ϱ_{calc}g/cm^3	1.568
μ/mm^{-1}	0.428
F(000)	672.0
Crystal size/mm^3	0.12 × 0.04 × 0.03
Radiation	MoKα (λ = 0.71073)
2Θ range for data collection/°	3.706 to 52.854
Index ranges	$-12 \le h \le 11, -14 \le k \le 14, -16 \le l \le 16$
Reflections collected	14784
Independent reflections	5686 [R_{int} = 0.1040, R_{sigma} = 0.1356]
Data/restraints/parameters	5686/0/363
Goodness-of-fit on F^2	1.024
Final R indexes [I>=2σ (I)]	R_1 = 0.0828, wR_2 = 0.1868
Final R indexes [all data]	R_1 = 0.1687, wR_2 = 0.2389
Largest diff. peak/hole / e Å^{-3}	0.56/-0.59

The single crystal of 1,3-oxazinan-2-one product **(±)-109** was obtained by slow evaporation of the solvent system based on CH_2Cl_2 and cyclohexane. The ORTEP representation with 50% probability thermal ellipsoids were presented. Although the crystal originated from a racemic mixture, the crystallographic structure revealed only one enantiomer of **(±)-109**. Crystallographic data for the compound **(±)-109** has been deposited at the Cambridge Crystallographic Data Centre, under deposition numbers CCDC 2464379 (**(±)-109**). Copies of the data can be obtained free of charge via https://www.ccdc.cam.ac.uk/structures/.

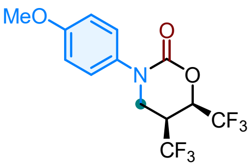

(±)-109

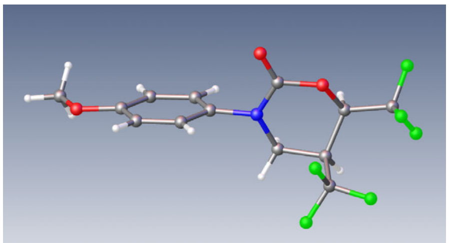

Empirical formula	$C_{13}H_{11}F_6NO_3$
Formula weight	343.23
Temperature/K	170
Crystal system	orthorhombic
Space group	$Pna2_1$
a/Å	11.3634(6)
b/Å	22.6450(13)
c/Å	5.4218(3)
α/°	90
β/°	90
γ/°	90
Volume/Å^3	1395.16(13)
Z	4
ρ_{calc}g/cm^3	1.634
μ/mm^{-1}	0.167
F(000)	696.0
Crystal size/mm^3	0.15 × 0.06 × 0.05
Radiation	MoKα (λ = 0.71073)
2Θ range for data collection/°	4.01 to 52.694
Index ranges	$-14 \le h \le 14, -27 \le k \le 19, -6 \le l \le 6$
Reflections collected	8906
Independent reflections	2500 [R_{int} = 0.0698, R_{sigma} = 0.0643]
Data/restraints/parameters	2500/1/209
Goodness-of-fit on F^2	1.063
Final R indexes [I>=2σ (I)]	R_1 = 0.0444, wR_2 = 0.0976
Final R indexes [all data]	R_1 = 0.0596, wR_2 = 0.1086
Largest diff. peak/hole / e Å^{-3}	0.21/-0.23
Flack parameter	1.2(8)

The single crystal of 1,3-oxazinan-2-one product **(±)-110** was obtained by slow evaporation of the solvent system based on CH_2Cl_2 and cyclohexane. The ORTEP representation with 50% probability thermal ellipsoids were presented. Although the crystal originated from a racemic mixture, the crystallographic structure revealed only one enantiomer of **(±)-110**, with two molecules showing normal geometry and one exhibiting a distorted structure. Crystallographic data for the compound **(±)-110** has been deposited at the Cambridge Crystallographic Data Centre, under deposition numbers CCDC 2464376 (**(±)-110**). Copies of the data can be obtained free of charge via https://www.ccdc.cam.ac.uk/structures/.

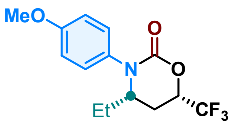

(±)-110

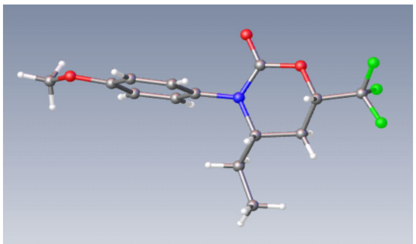

Empirical formula	$C_{14}H_{16}F_3NO_3$
Formula weight	303.28
Temperature/K	170
Crystal system	triclinic
Space group	P-1
a/Å	9.1929(7)
b/Å	13.2385(11)
c/Å	18.9694(15)
α/°	72.832(2)
β/°	79.483(2)
γ/°	86.551(2)
Volume/Å^3	2168.6(3)
Z	6
ρ_{calc}g/cm^3	1.393
μ/mm^{-1}	0.122
F(000)	948.0
Crystal size/mm^3	0.12 × 0.06 × 0.05
Radiation	MoKα (λ = 0.71073)
2Θ range for data collection/°	4.454 to 52.786
Index ranges	$-11 \le h \le 10$, $-16 \le k \le 16$, $-23 \le l \le 23$
Reflections collected	24991
Independent reflections	8864 [R_{int} = 0.0818, R_{sigma} = 0.0964]
Data/restraints/parameters	8864/229/639
Goodness-of-fit on F^2	1.062
Final R indexes [I>=2σ (I)]	R_1 = 0.0772, wR_2 = 0.1617
Final R indexes [all data]	R_1 = 0.1539, wR_2 = 0.2021
Largest diff. peak/hole / e Å^{-3}	0.33/-0.34

NMR Spectra

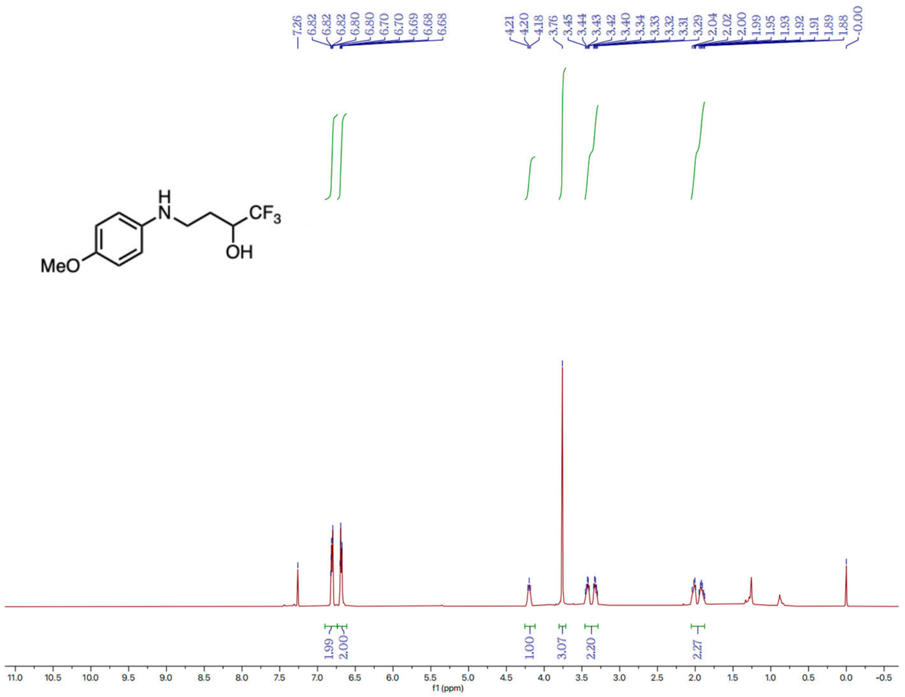

^{1}H NMR spectrum (500 MHz, Chloroform-*d*) of compound **11**

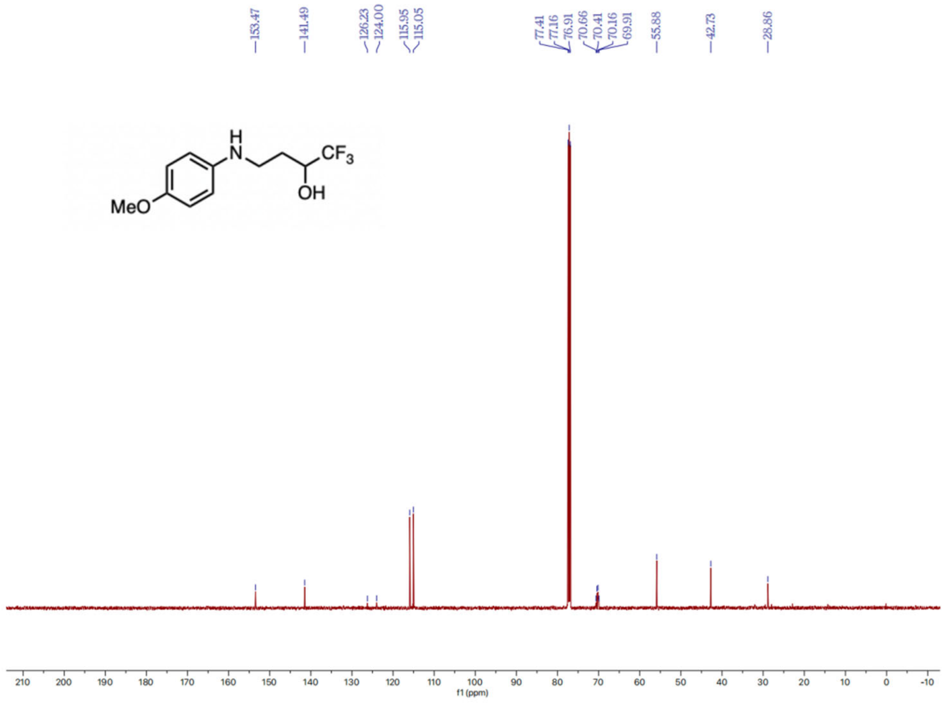

^{13}C NMR spectrum (126 MHz, Chloroform-*d*) of compound **11**

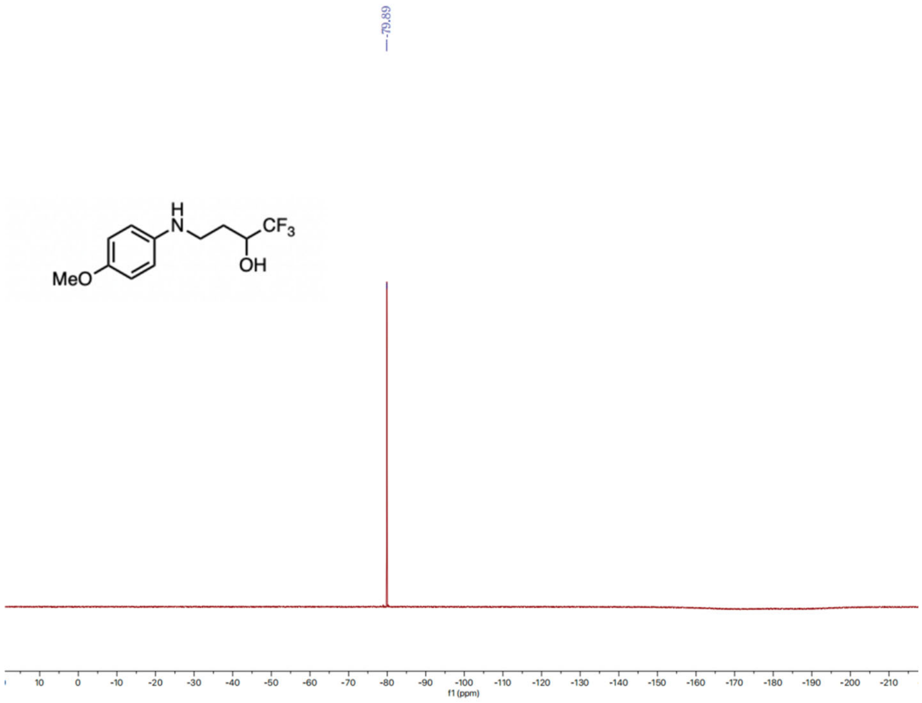

^{19}F NMR spectrum (376 MHz, Chloroform-*d*) of compound **11**

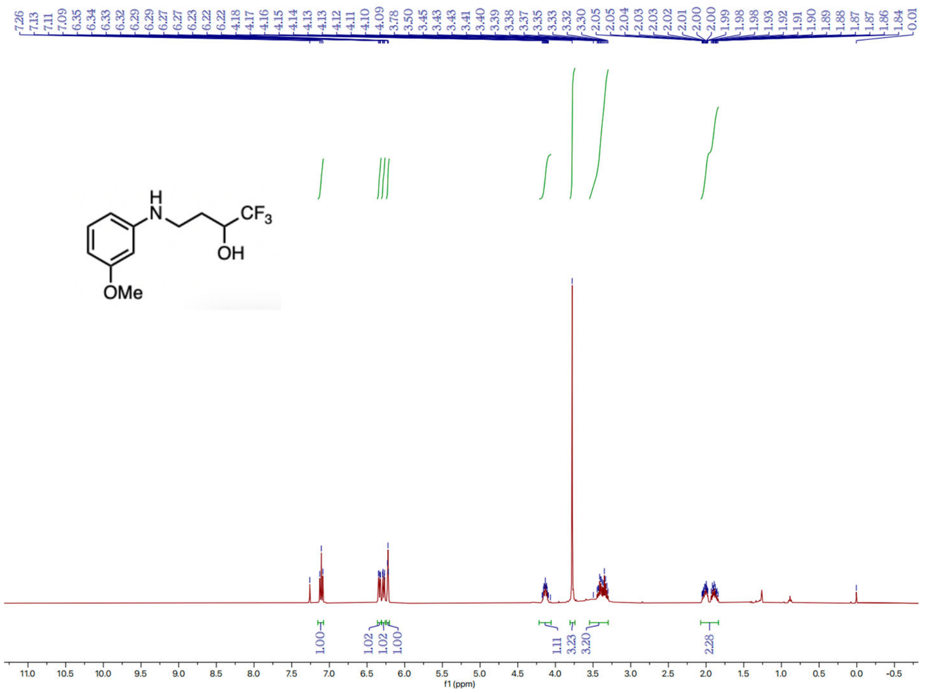

^{1}H NMR spectrum (400 MHz, Chloroform-*d*) of compound **12**

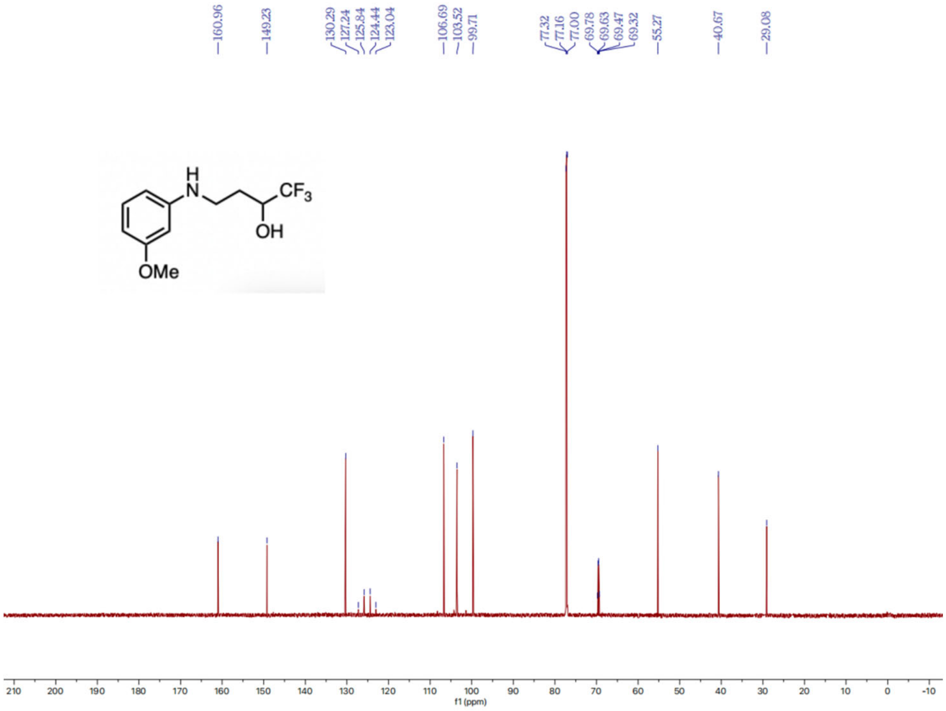

^{13}C NMR spectrum (201 MHz, Chloroform-*d*) of compound **12**

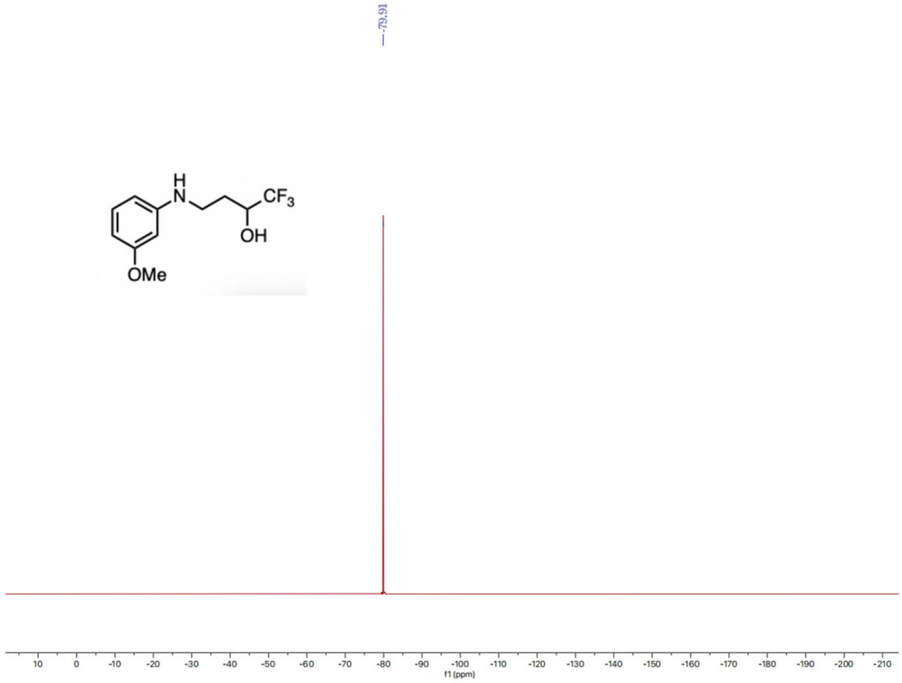

^{19}F NMR spectrum (376 MHz, Chloroform-*d*) of compound **12**

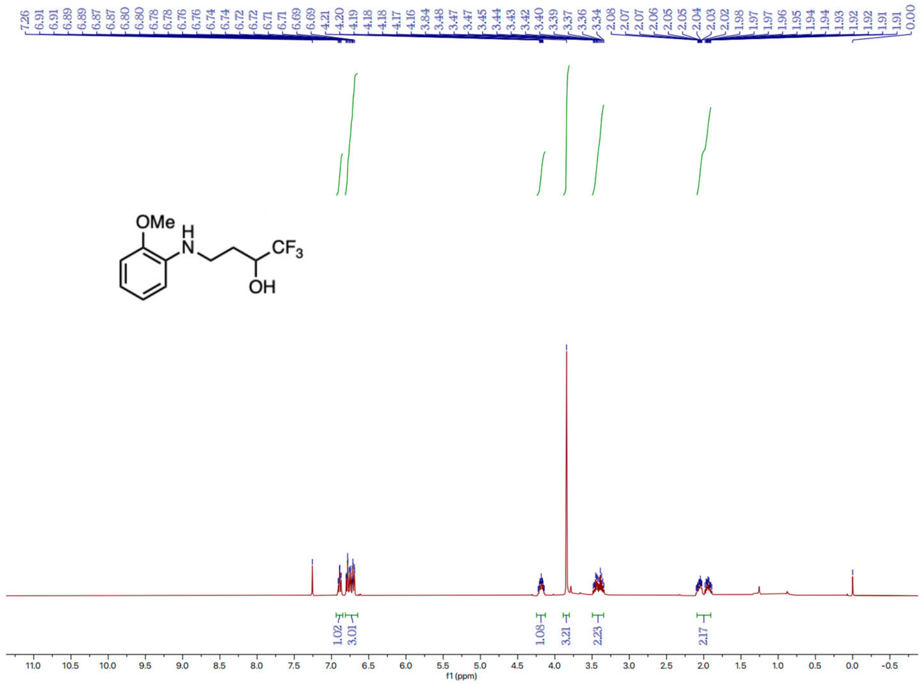

^{1}H NMR spectrum (400 MHz, Chloroform-*d*) of compound **13**

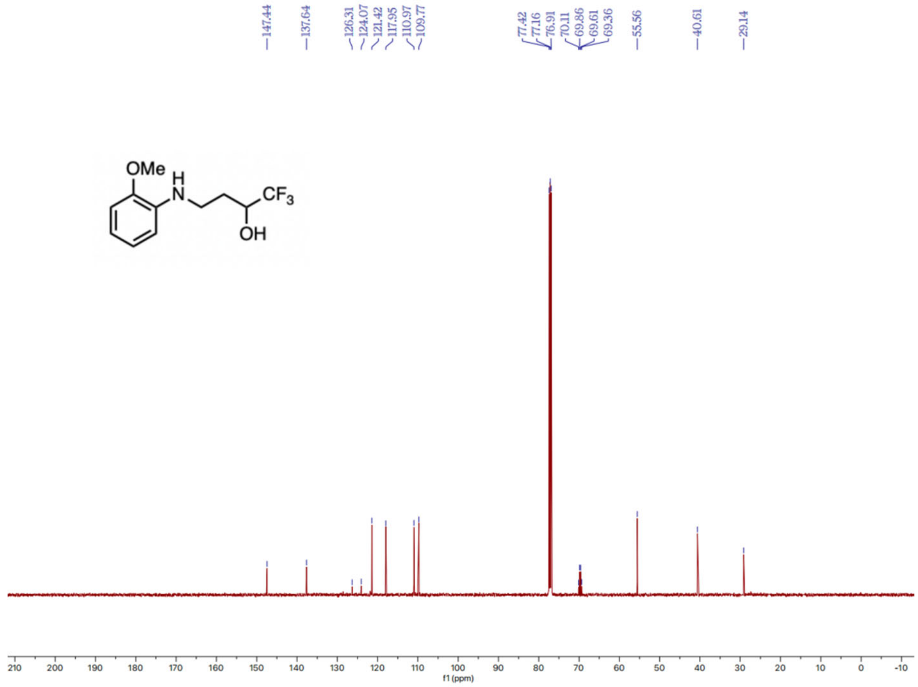

^{13}C NMR spectrum (126 MHz, Chloroform-*d*) of compound **13**

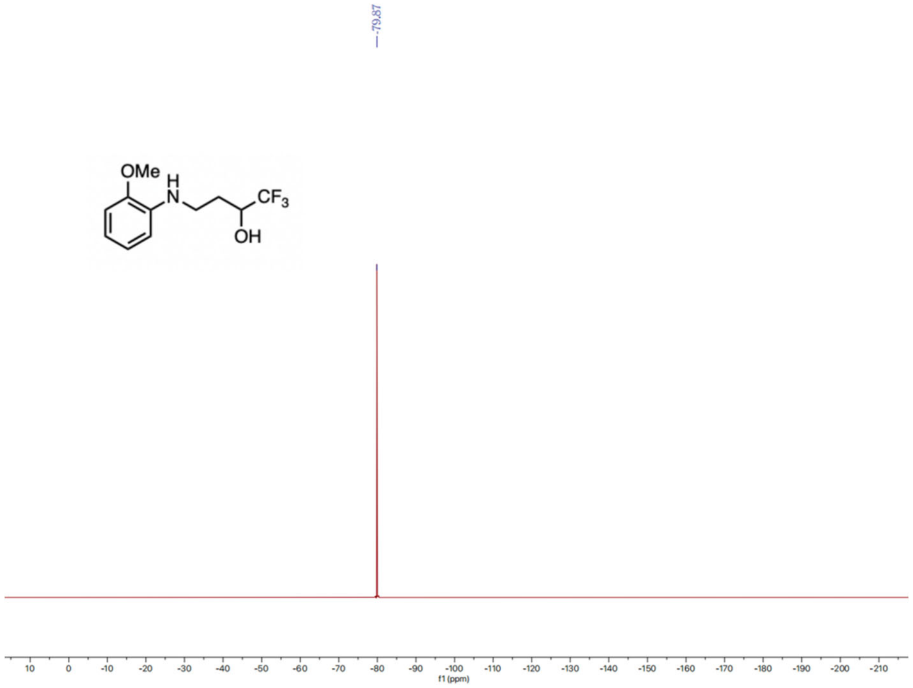

^{19}F NMR spectrum (376 MHz, Chloroform-*d*) of compound **13**

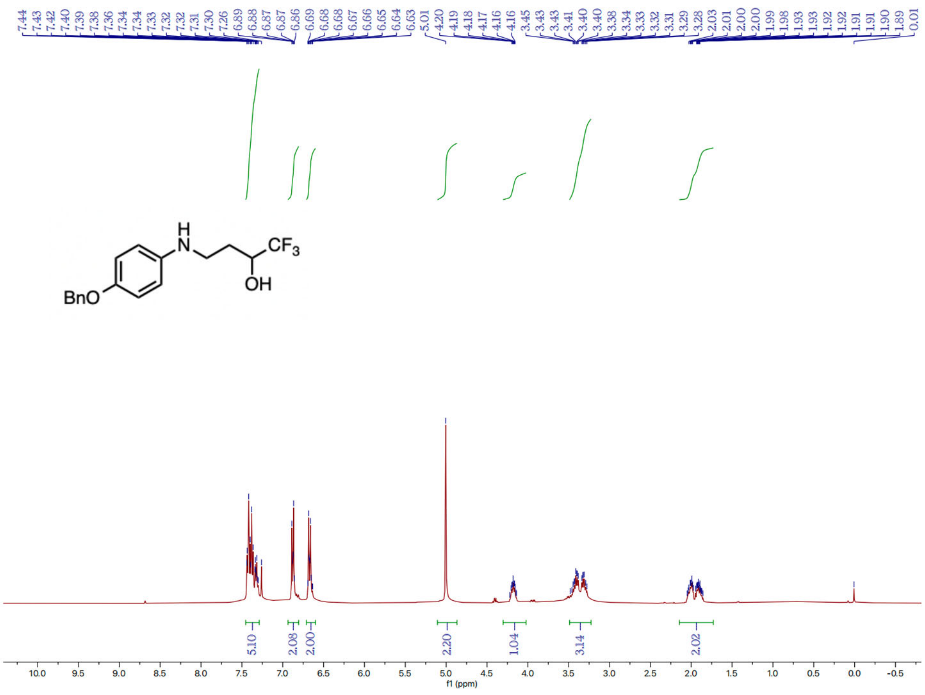

^{1}H NMR spectrum (400 MHz, Chloroform-*d*) of compound **14**

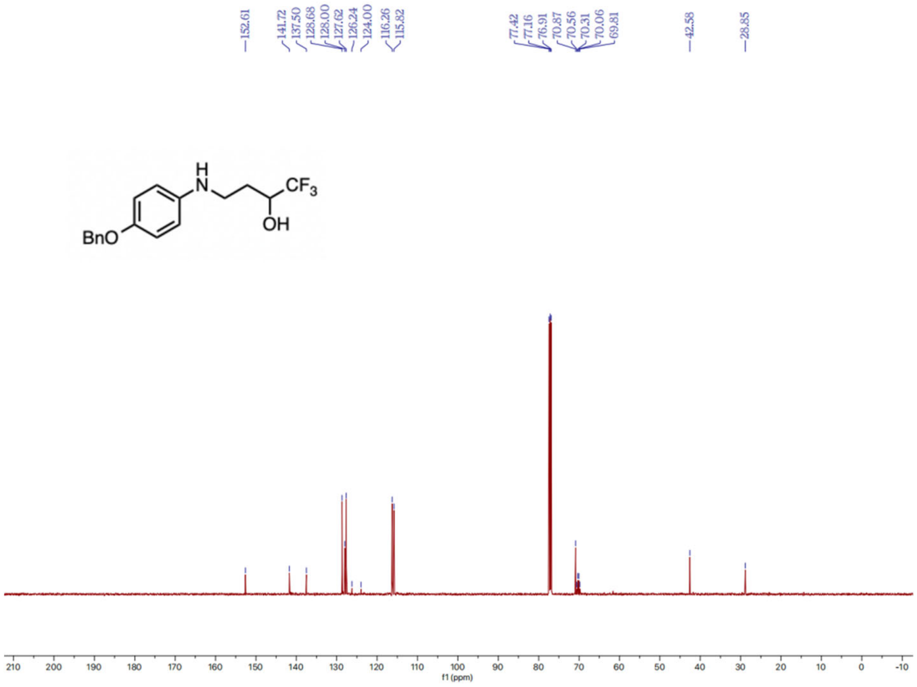

^{13}C NMR spectrum (126 MHz, Chloroform-*d*) of compound **14**

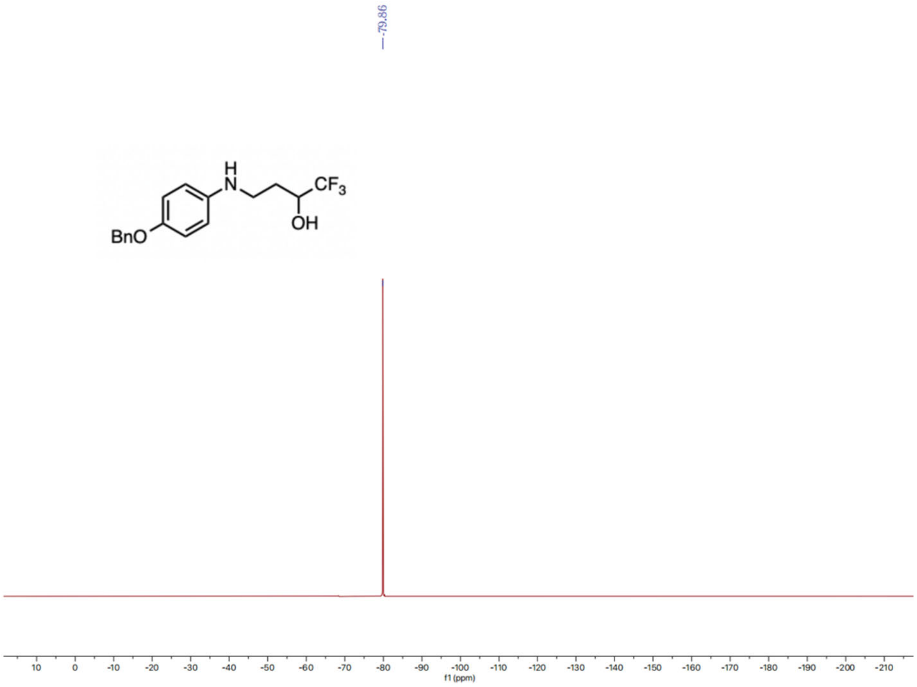

^{19}F NMR spectrum (376 MHz, Chloroform-*d*) of compound **14**

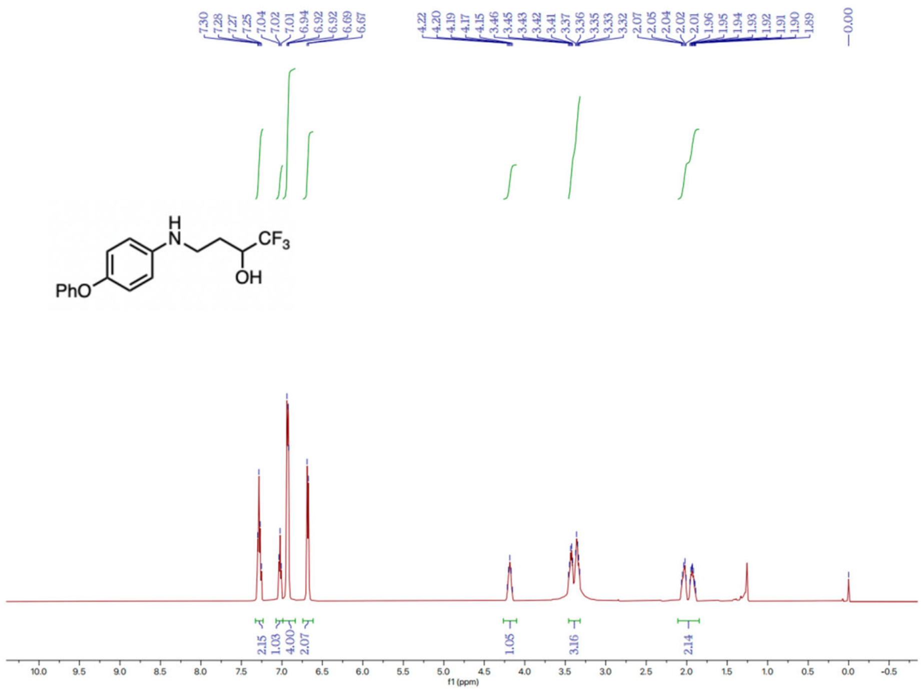

^{1}H NMR spectrum (500 MHz, Chloroform-*d*) of compound **15**

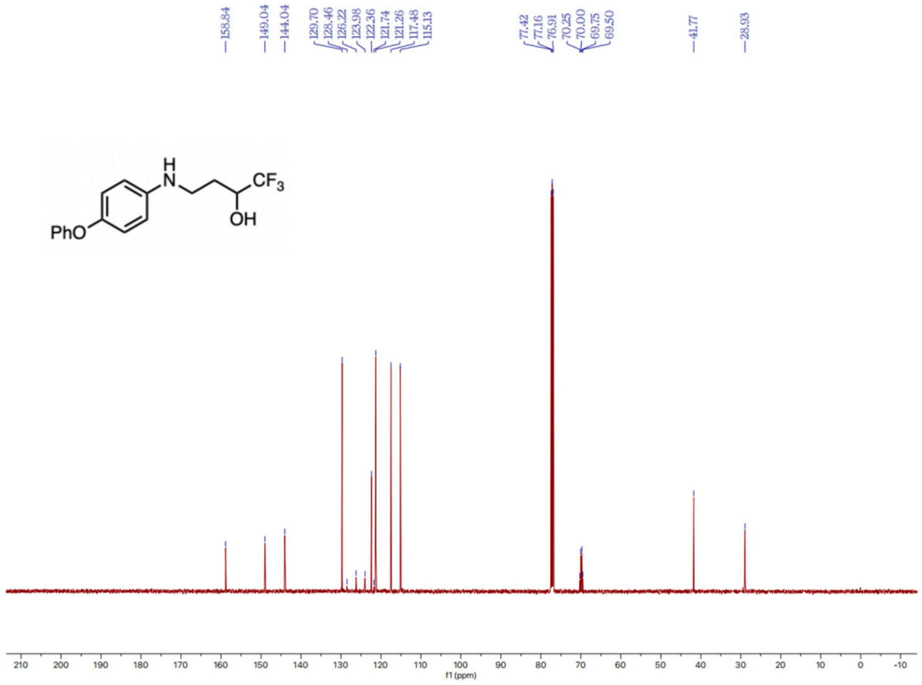

^{13}C NMR spectrum (126 MHz, Chloroform-*d*) of compound **15**

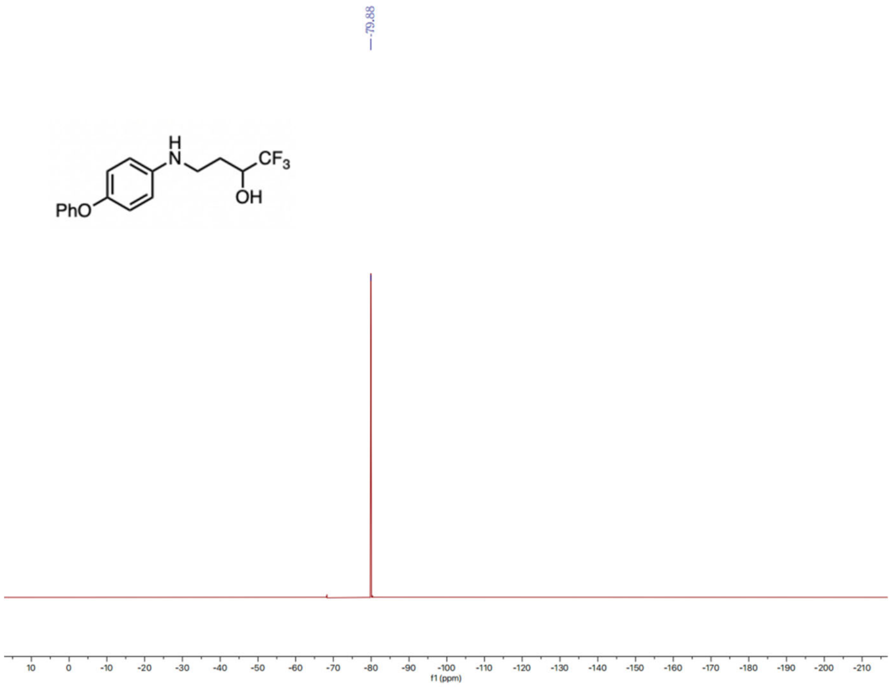

^{19}F NMR spectrum (471 MHz, Chloroform-*d*) of compound **15**

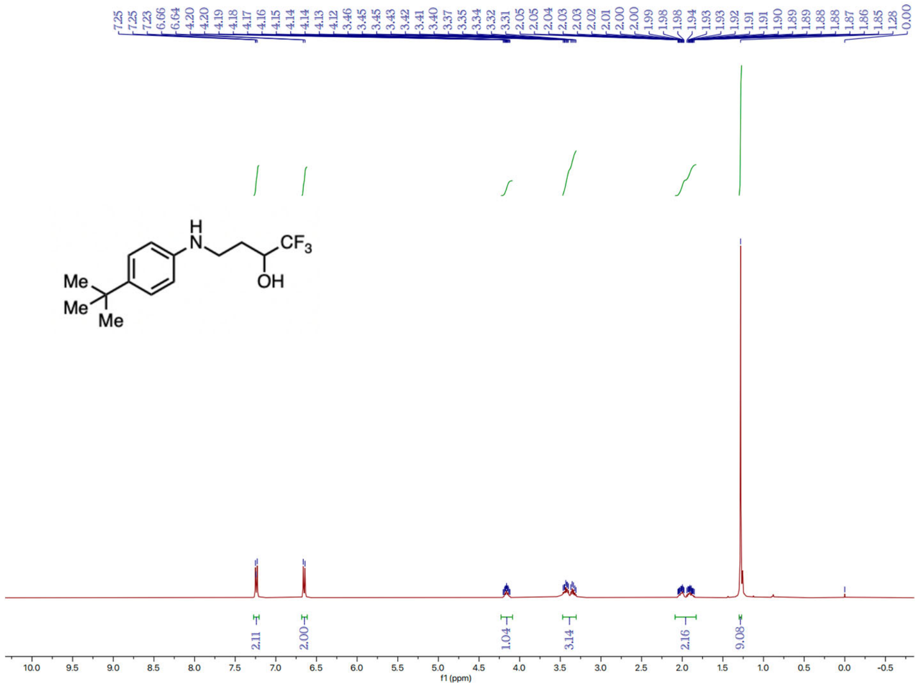

^{1}H NMR spectrum (400 MHz, Chloroform-*d*) of compound **16**

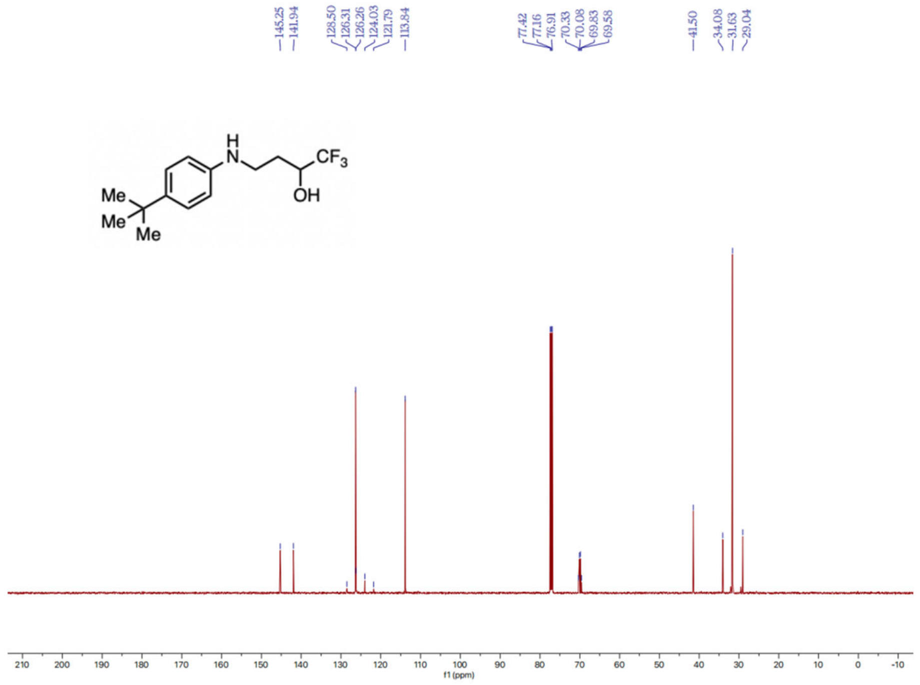

^{13}C NMR spectrum (126 MHz, Chloroform-*d*) of compound **16**

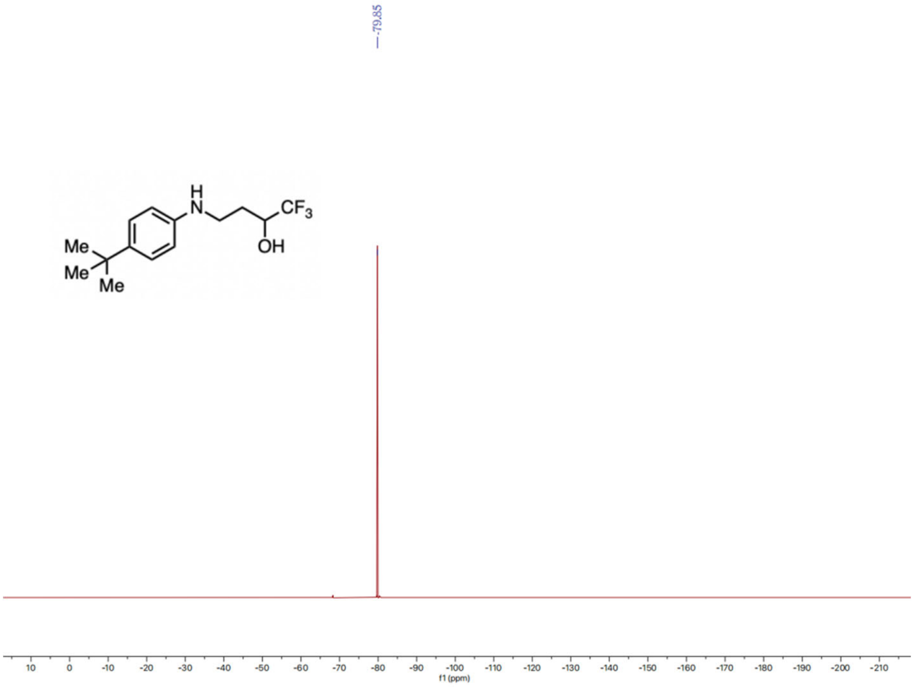

^{19}F NMR spectrum (376 MHz, Chloroform-*d*) of compound **16**

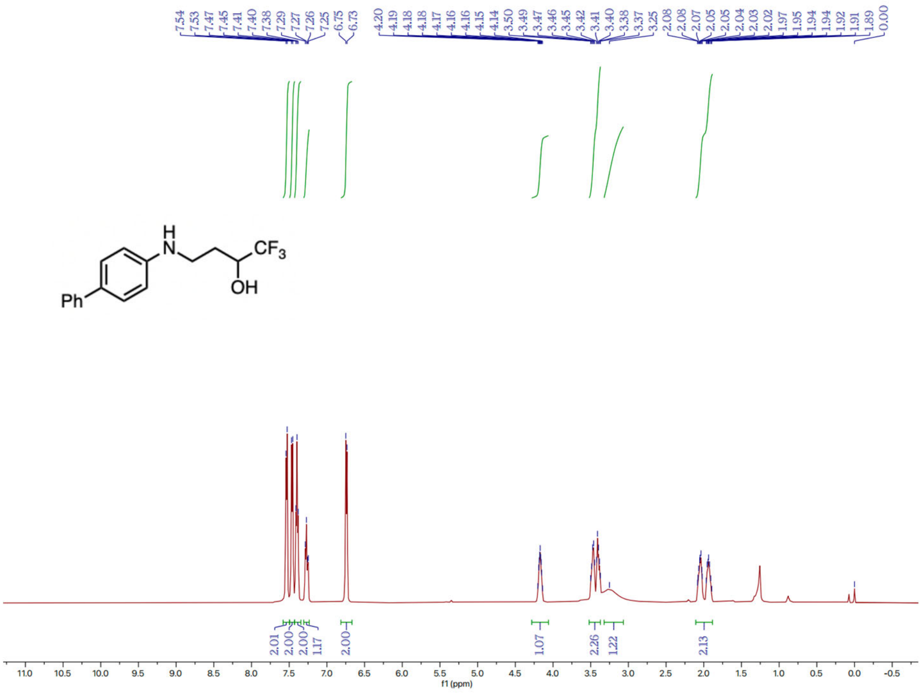

^{1}H NMR spectrum (500 MHz, Chloroform-*d*) of compound **17**

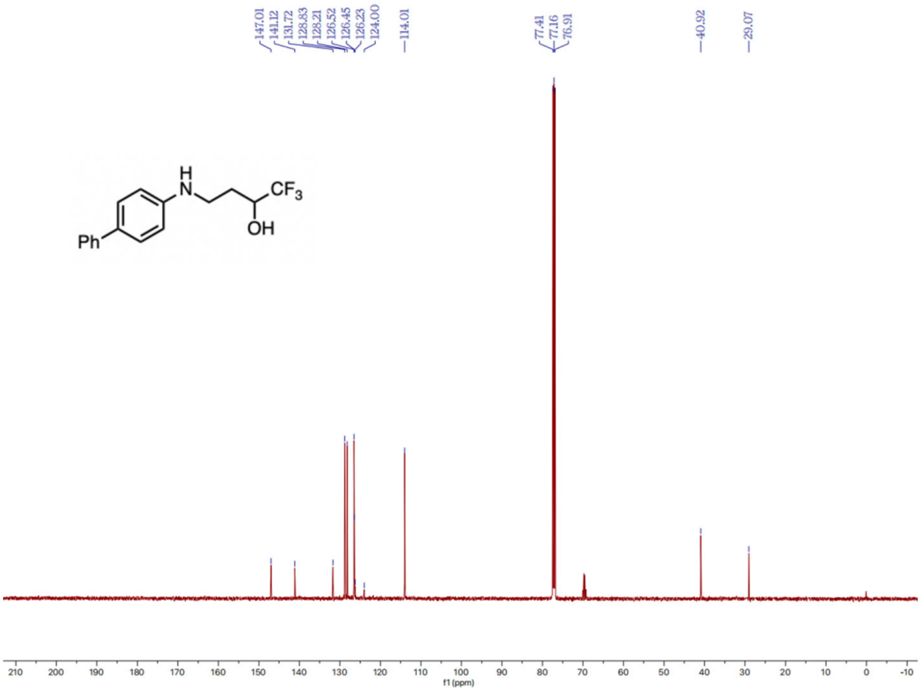

^{13}C NMR spectrum (126 MHz, Chloroform-*d*) of compound **17**

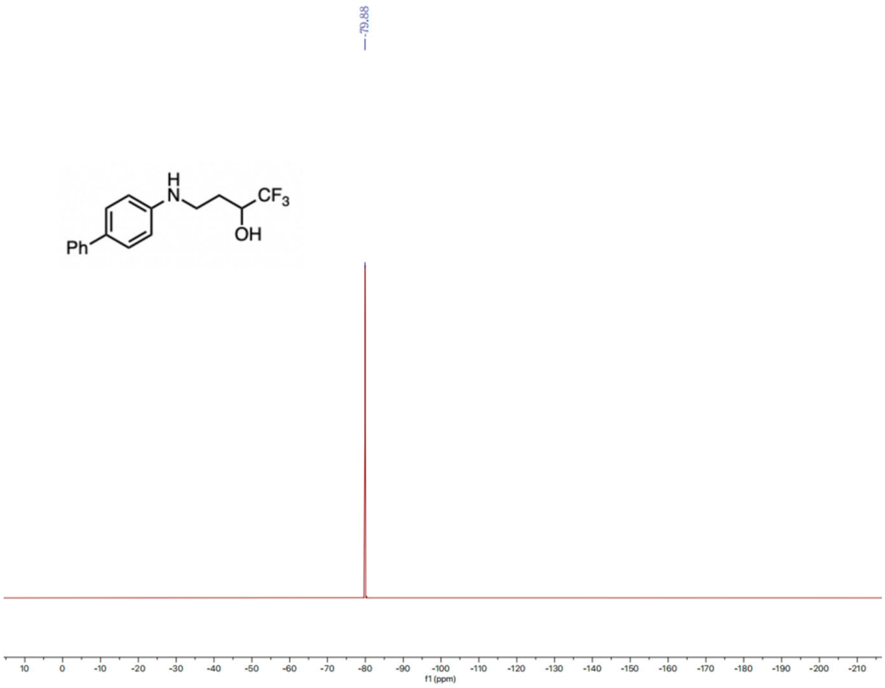

^{19}F NMR spectrum (471 MHz, Chloroform-*d*) of compound **17**

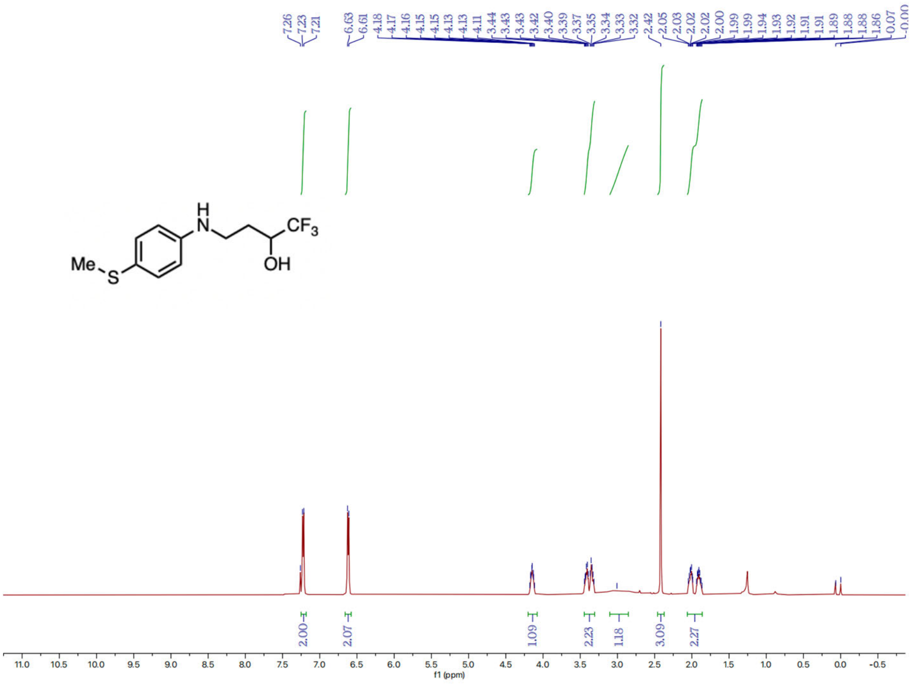

^{1}H NMR spectrum (500 MHz, Chloroform-*d*) of compound **18**

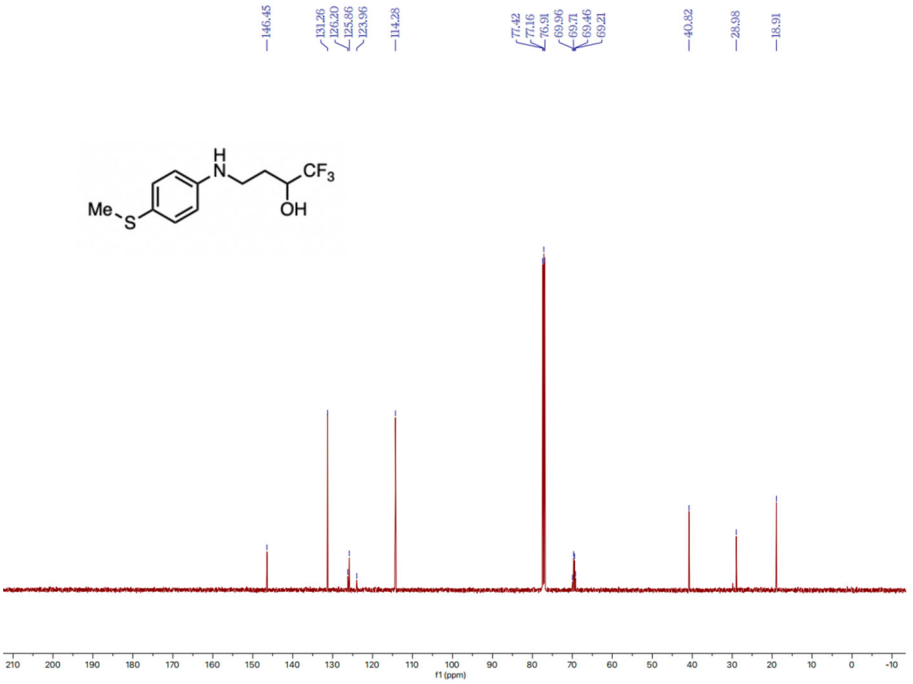

^{13}C NMR spectrum (126 MHz, Chloroform-*d*) of compound **18**

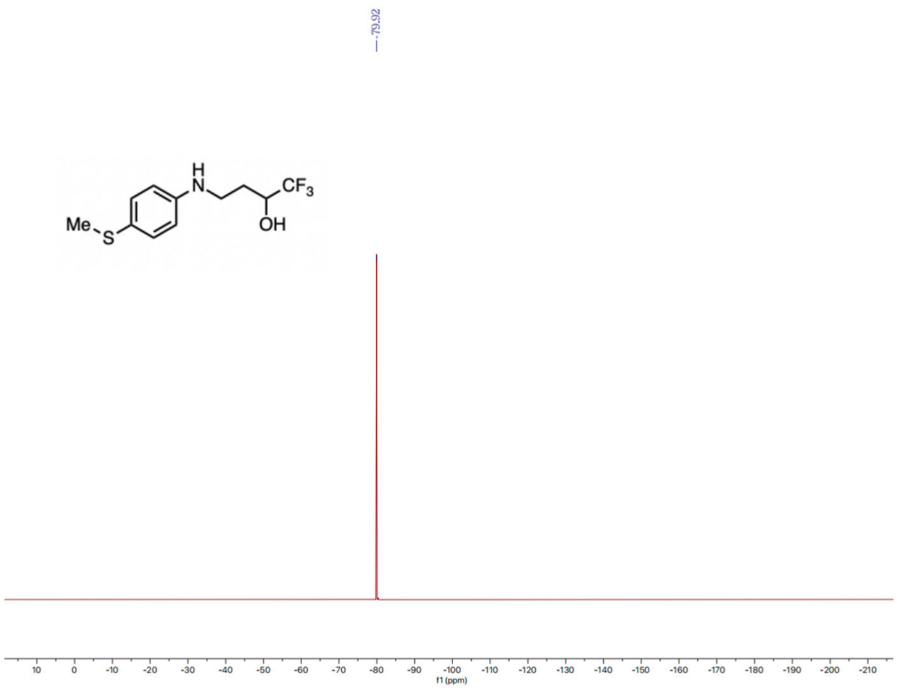

^{19}F NMR spectrum (471 MHz, Chloroform-*d*) of compound **18**

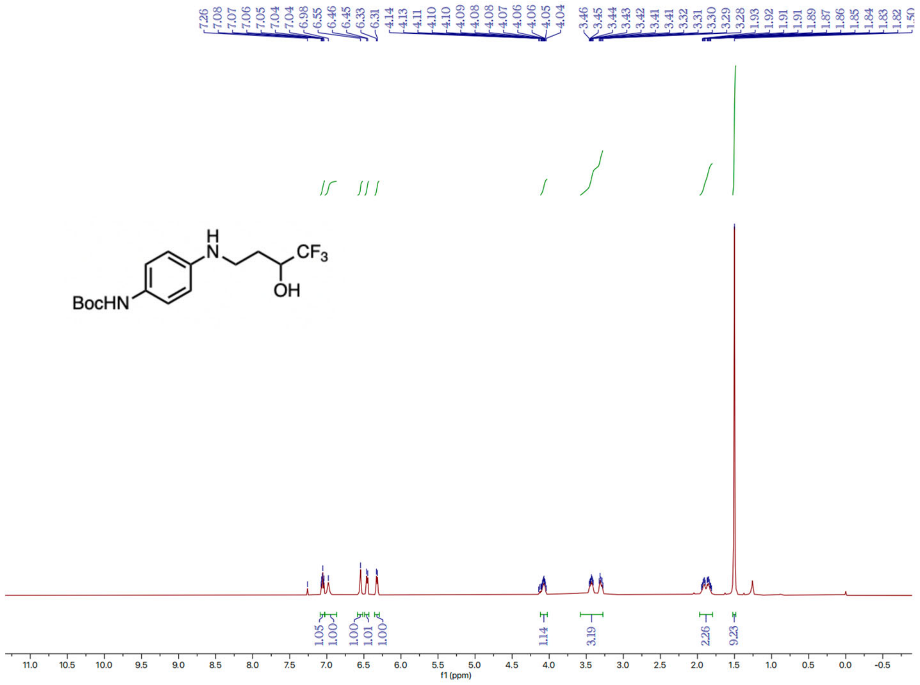

^{1}H NMR spectrum (500 MHz, Chloroform-*d*) of compound **19**

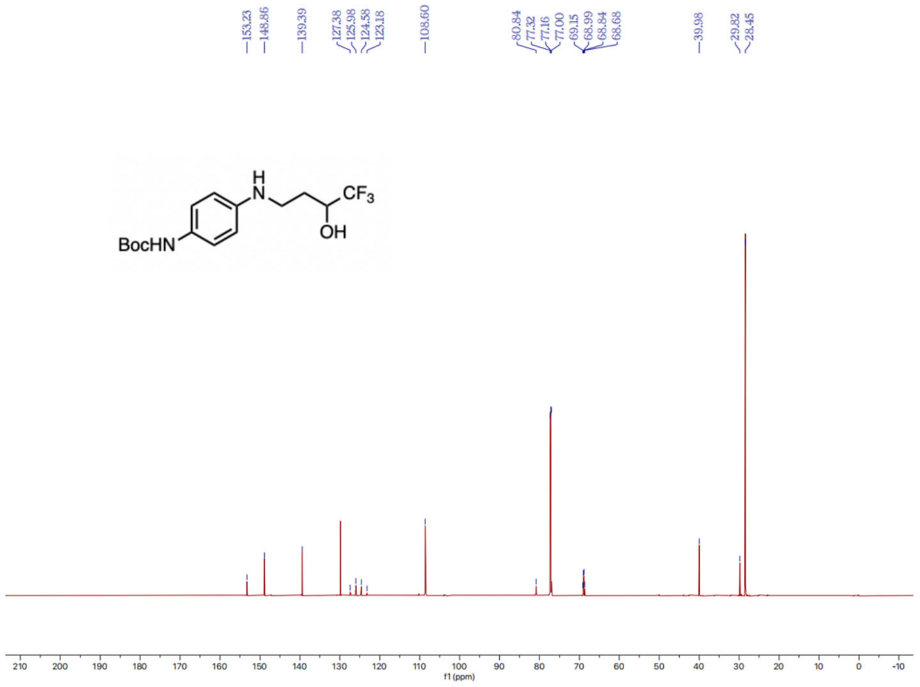

^{13}C NMR spectrum (201 MHz, Chloroform-*d*) of compound **19**

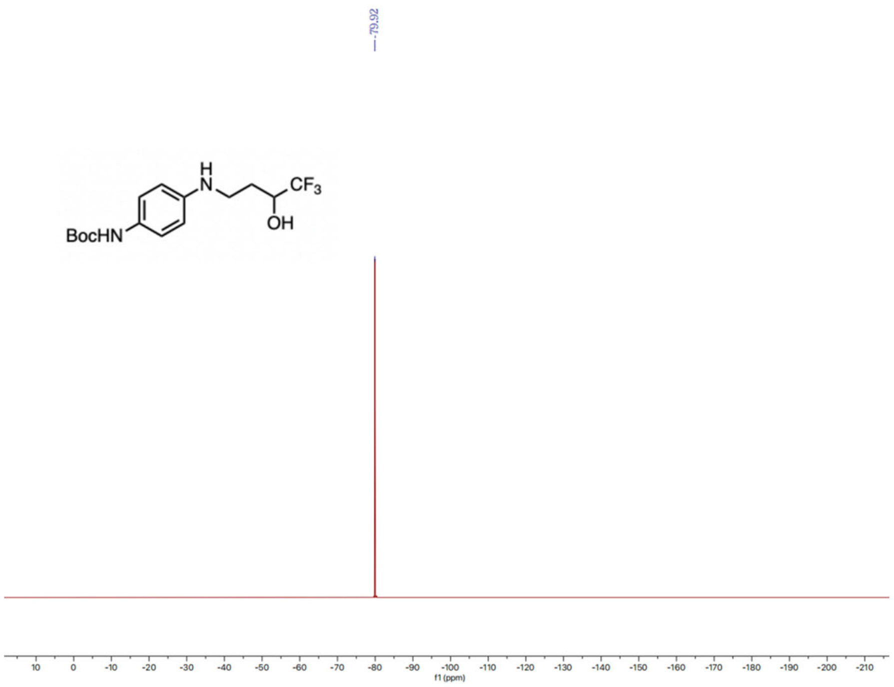

^{19}F NMR spectrum (471 MHz, Chloroform-*d*) of compound **19**

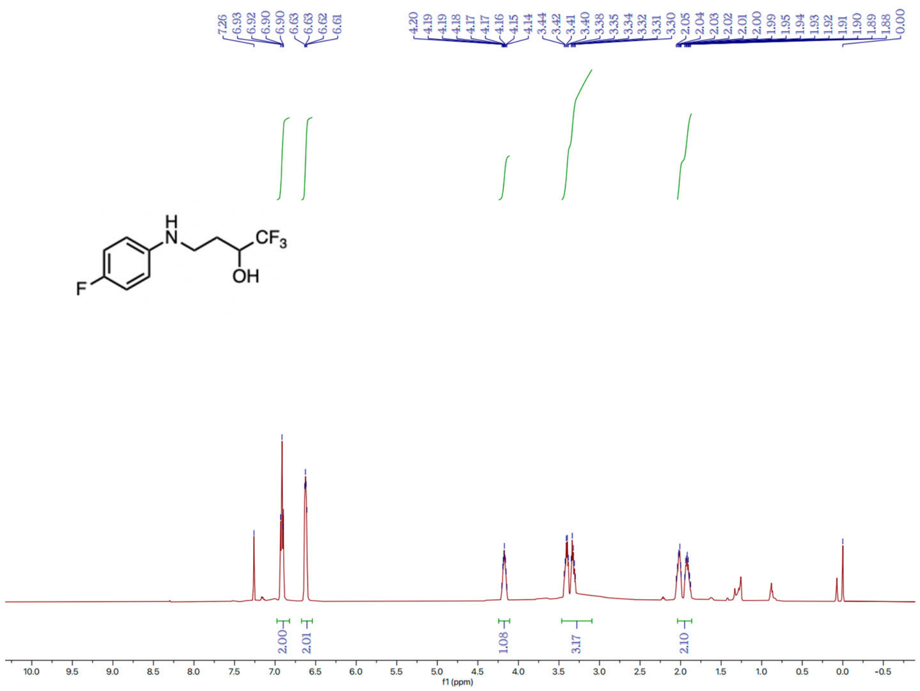

^{1}H NMR spectrum (500 MHz, Chloroform-*d*) of compound **20**

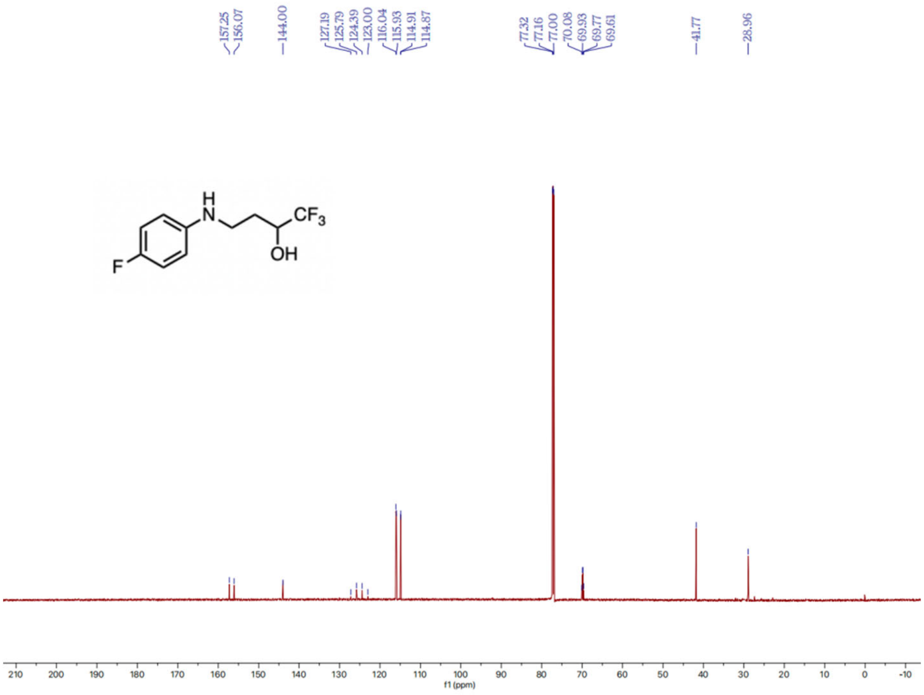

^{13}C NMR spectrum (201 MHz, Chloroform-*d*) of compound **20**

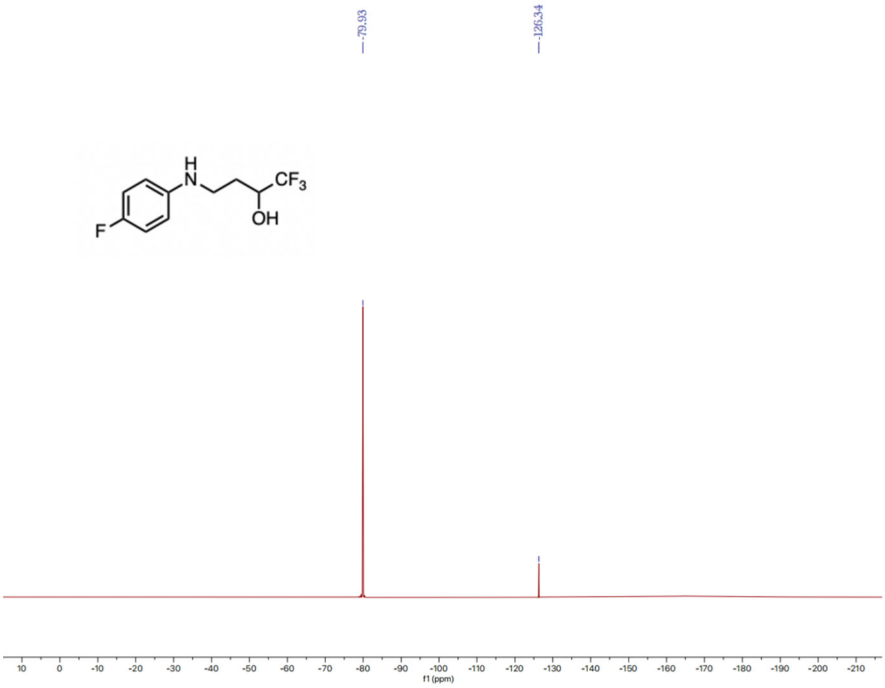

^{19}F NMR spectrum (471 MHz, Chloroform-*d*) of compound **20**

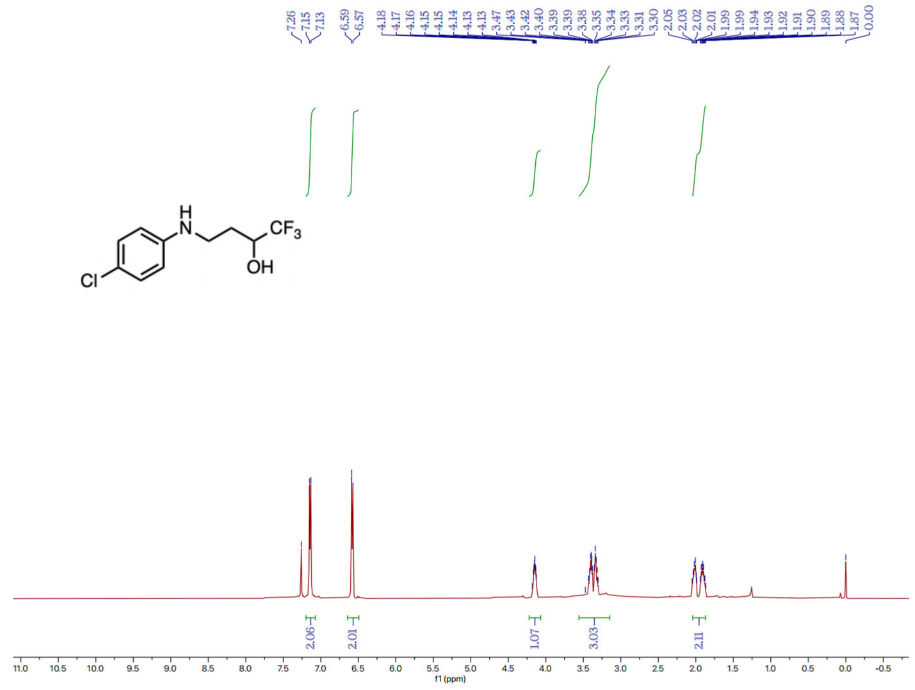

^{1}H NMR spectrum (500 MHz, Chloroform-*d*) of compound **21**

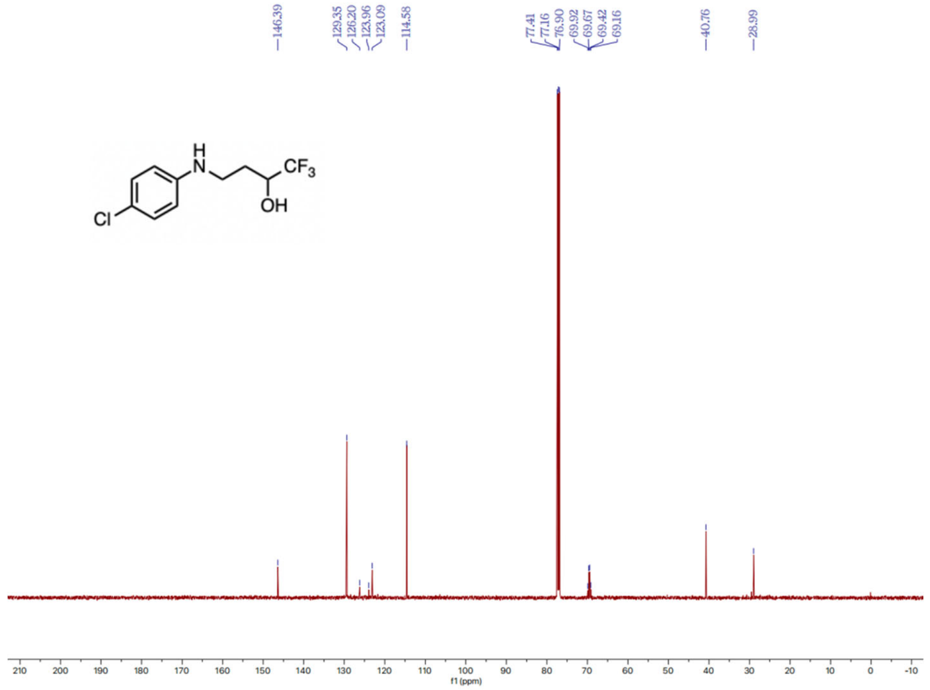

^{13}C NMR spectrum (126 MHz, Chloroform-*d*) of compound **21**

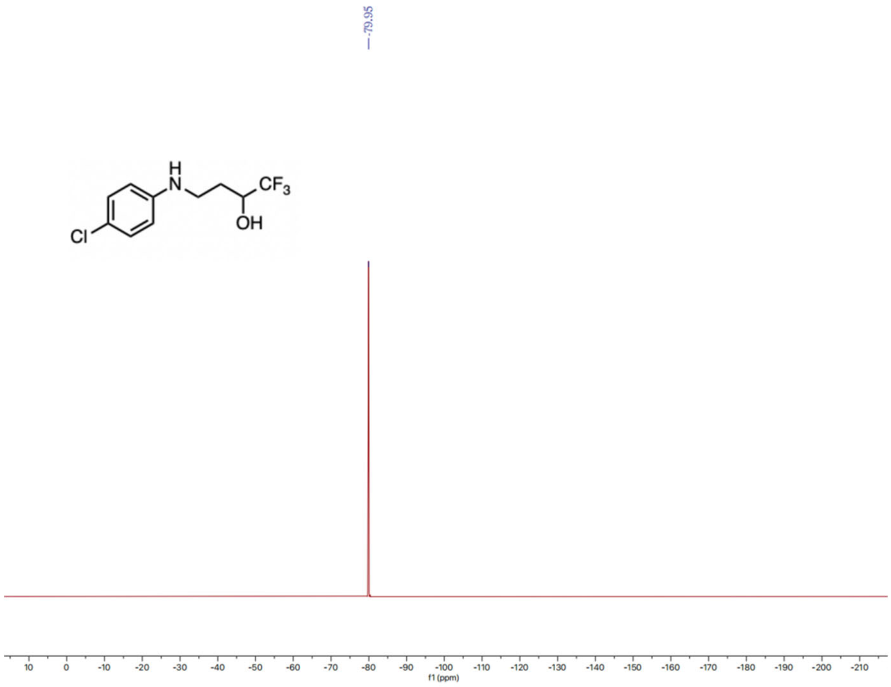

^{19}F NMR spectrum (471 MHz, Chloroform-*d*) of compound **21**

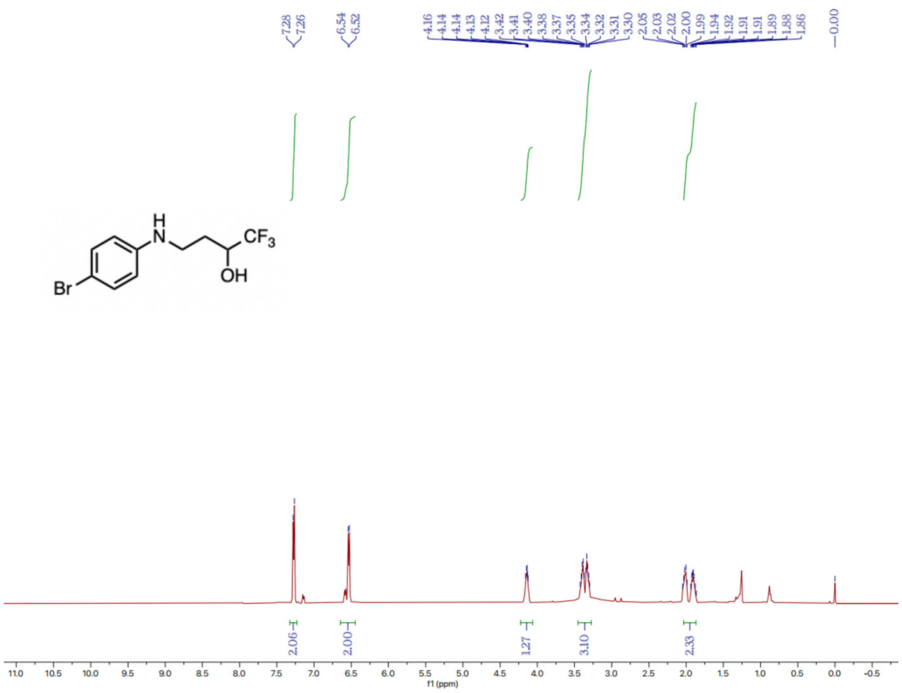

^{1}H NMR spectrum (500 MHz, Chloroform-*d*) of compound **22**

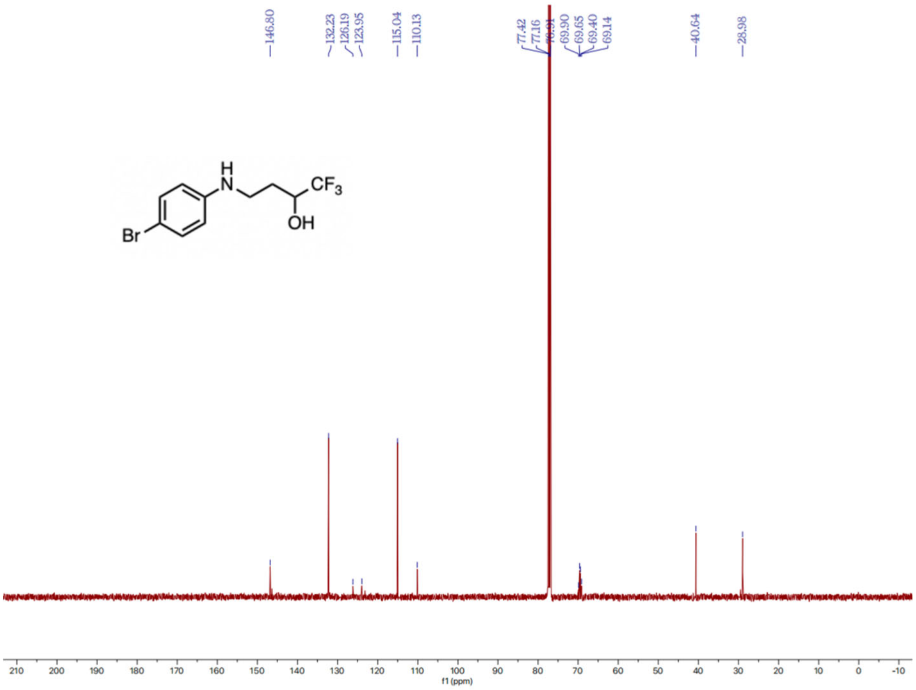

^{13}C NMR spectrum (126 MHz, Chloroform-*d*) of compound **22**

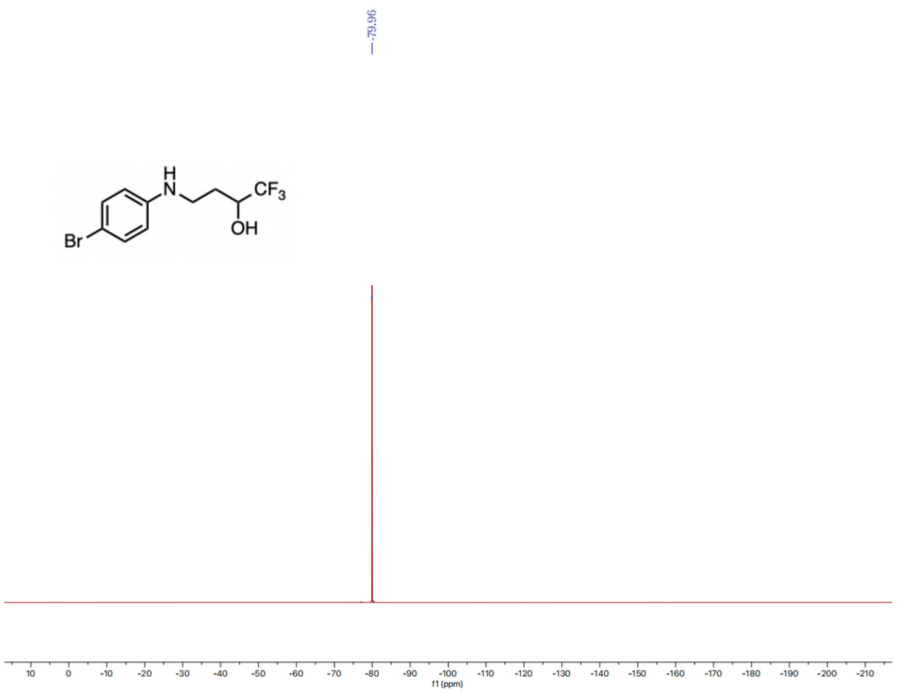

^{19}F NMR spectrum (471 MHz, Chloroform-*d*) of compound **22**

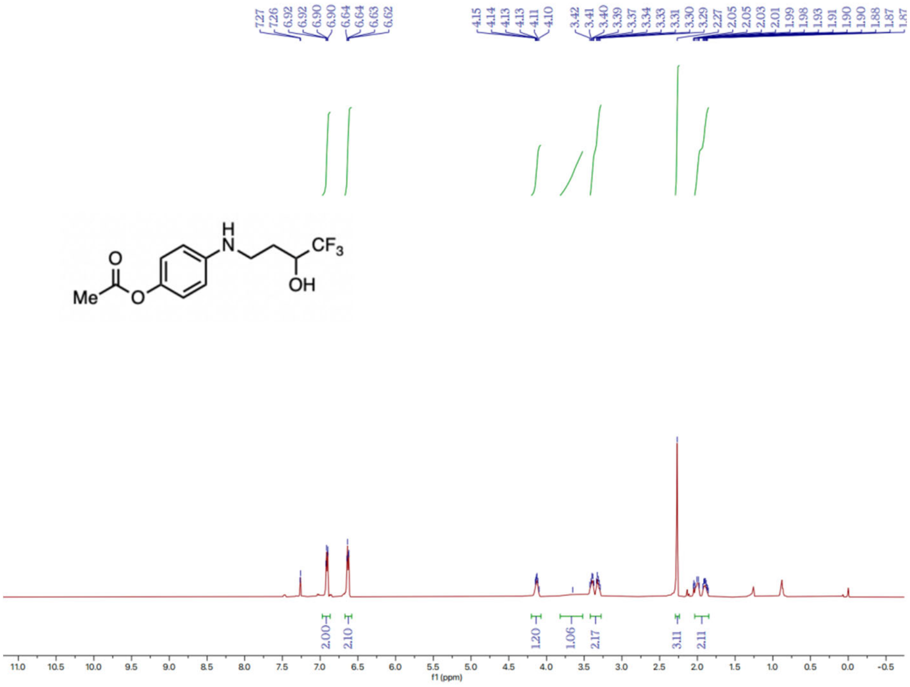

^{1}H NMR spectrum (500 MHz, Chloroform-*d*) of compound **23**

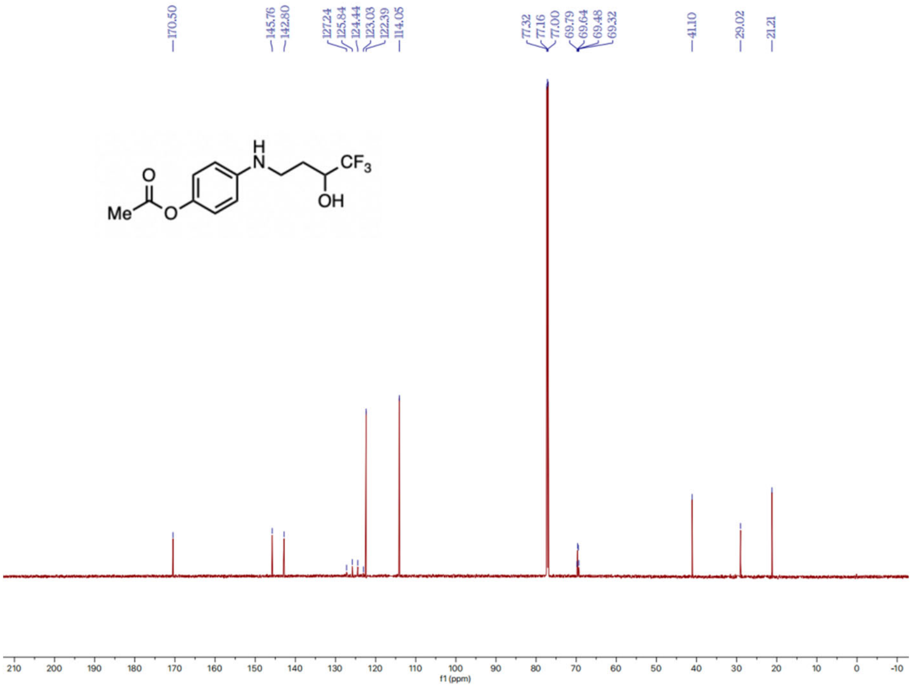

^{13}C NMR spectrum (201 MHz, Chloroform-*d*) of compound **23**

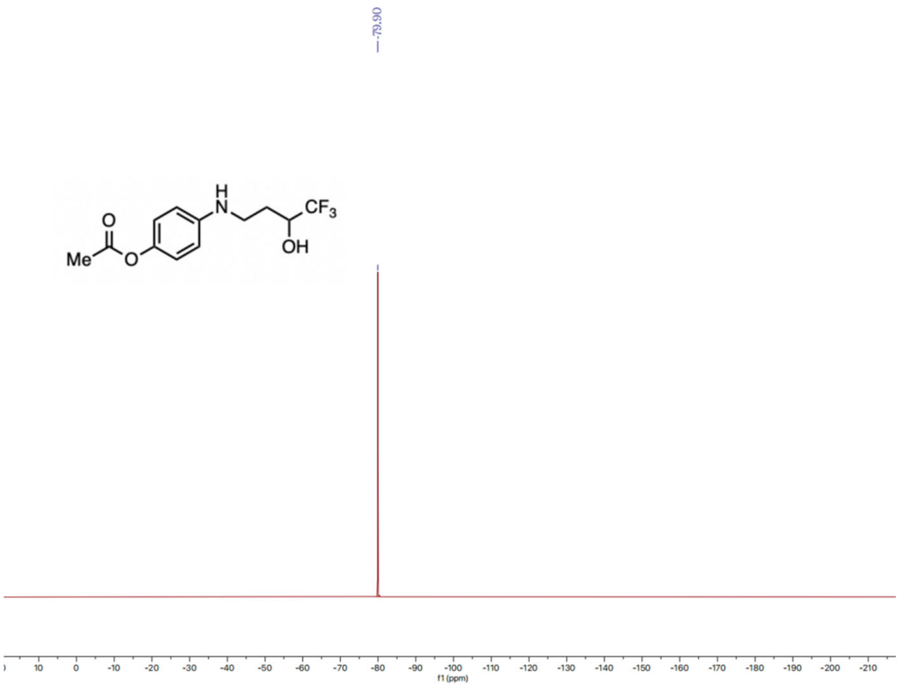

^{19}F NMR spectrum (471 MHz, Chloroform-*d*) of compound **23**

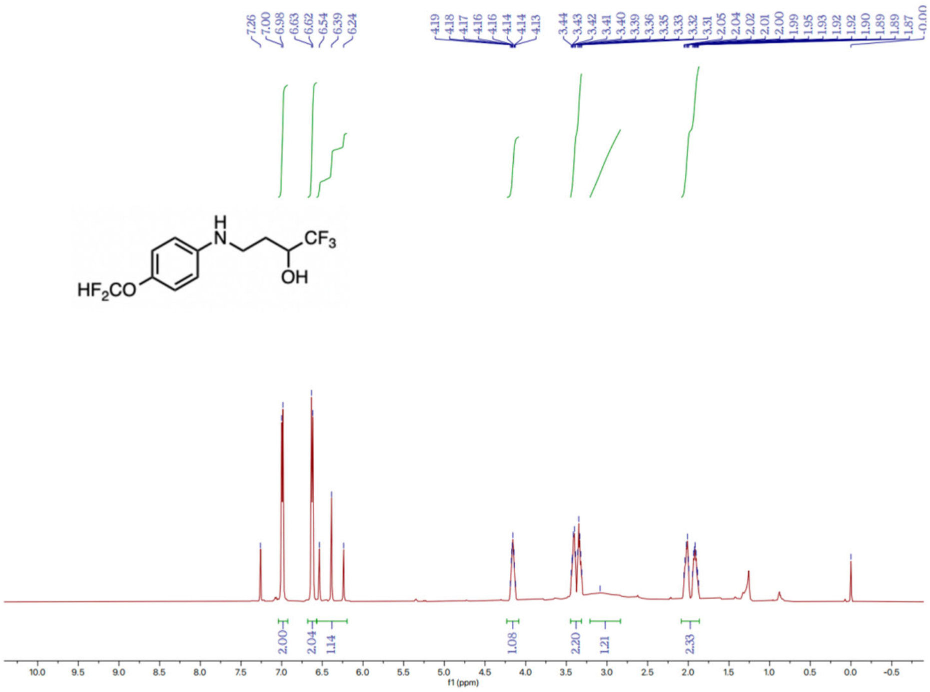

^{1}H NMR spectrum (500 MHz, Chloroform-*d*) of compound **24**

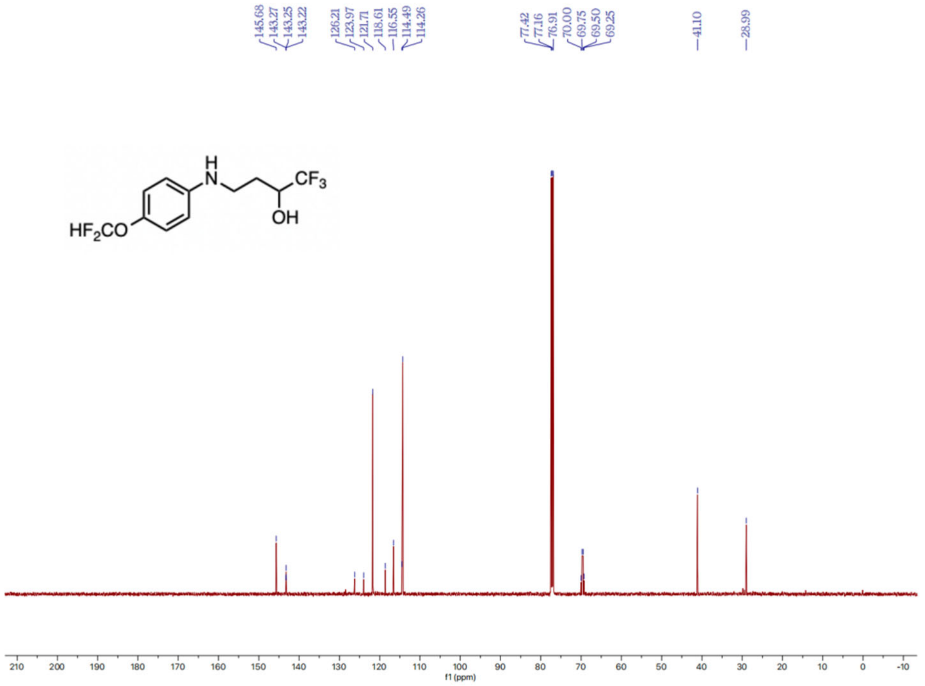

^{13}C NMR spectrum (126 MHz, Chloroform-*d*) of compound **24**

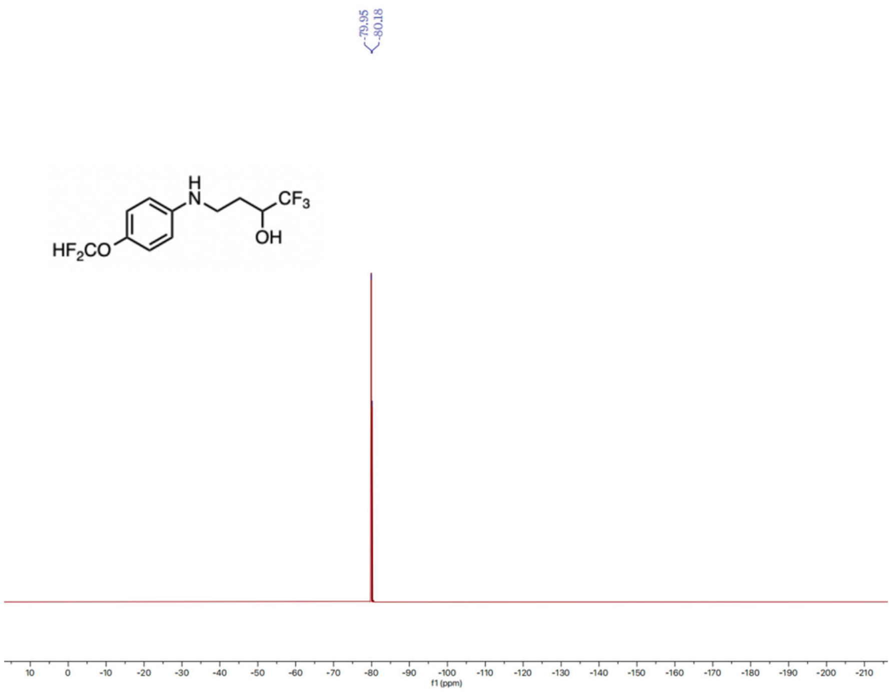

^{19}F NMR spectrum (471 MHz, Chloroform-*d*) of compound **24**

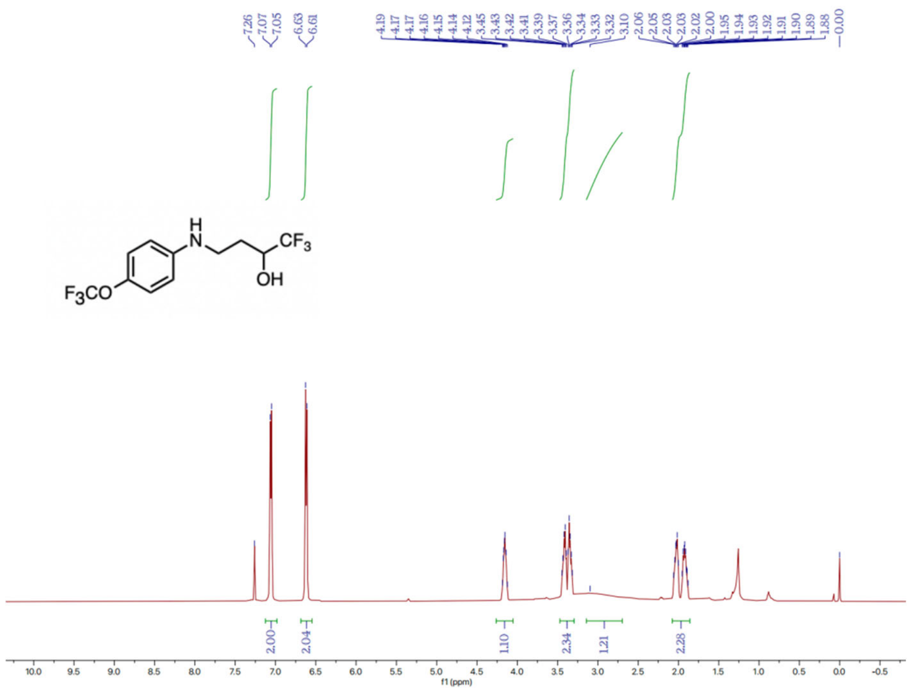

^{1}H NMR spectrum (500 MHz, Chloroform-*d*) of compound **25**

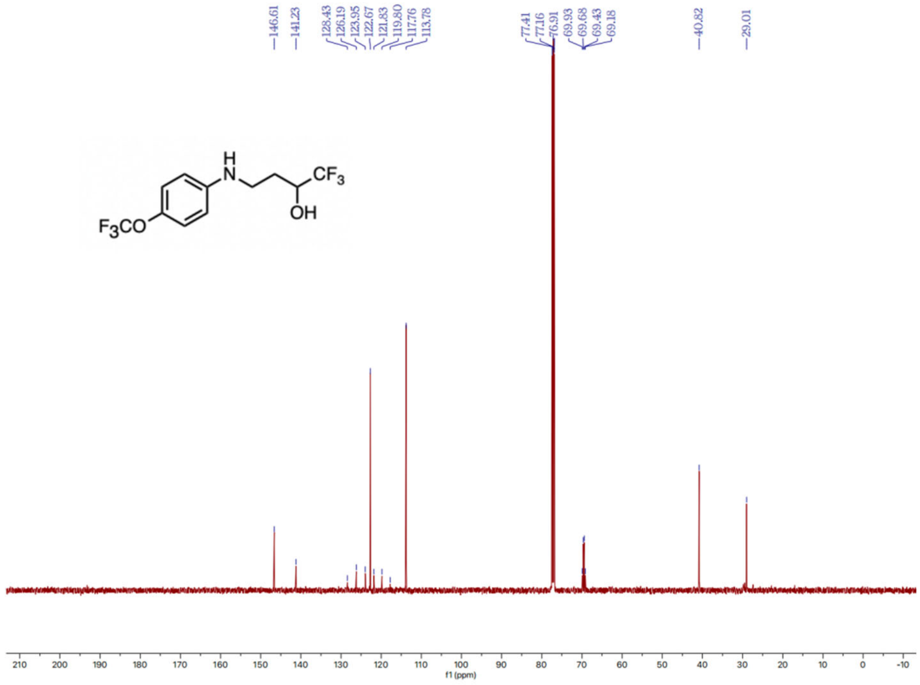

^{13}C NMR spectrum (126 MHz, Chloroform-*d*) of compound **25**

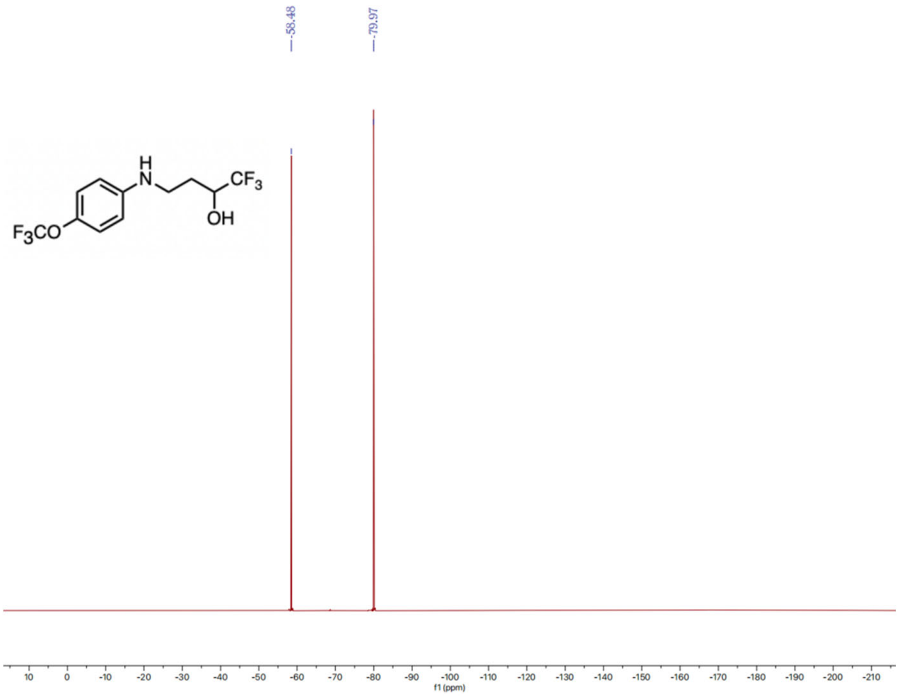

^{19}F NMR spectrum (471 MHz, Chloroform-*d*) of compound **25**

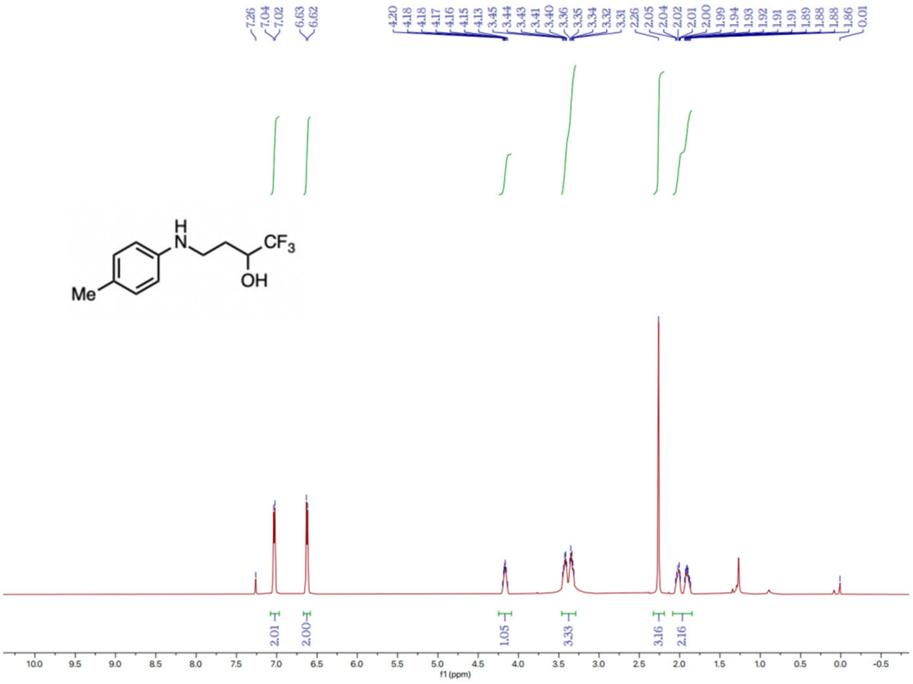

^{1}H NMR spectrum (500 MHz, Chloroform-*d*) of compound **26**

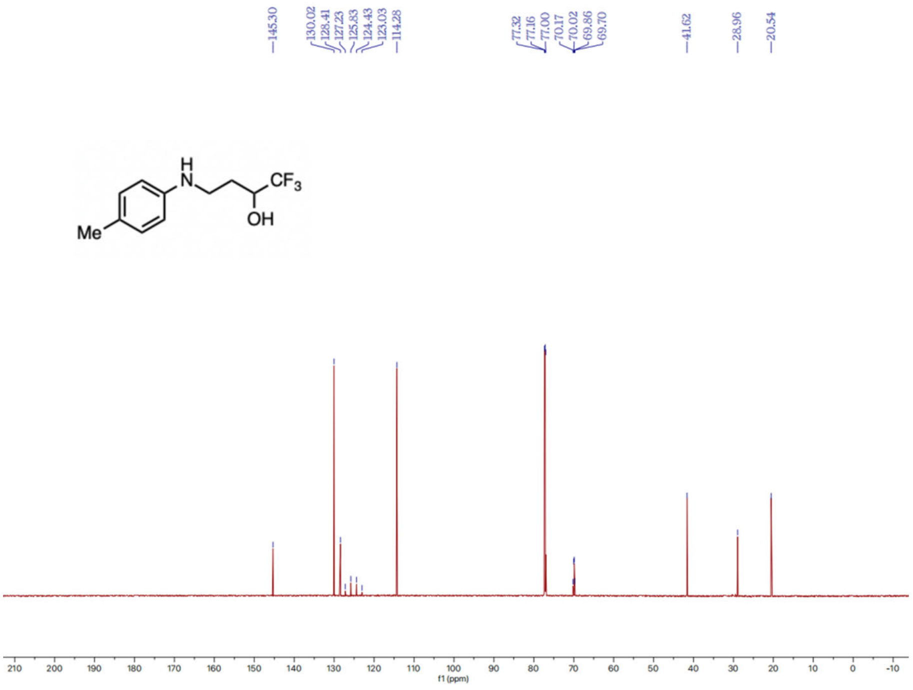

^{13}C NMR spectrum (210 MHz, Chloroform-*d*) of compound **26**

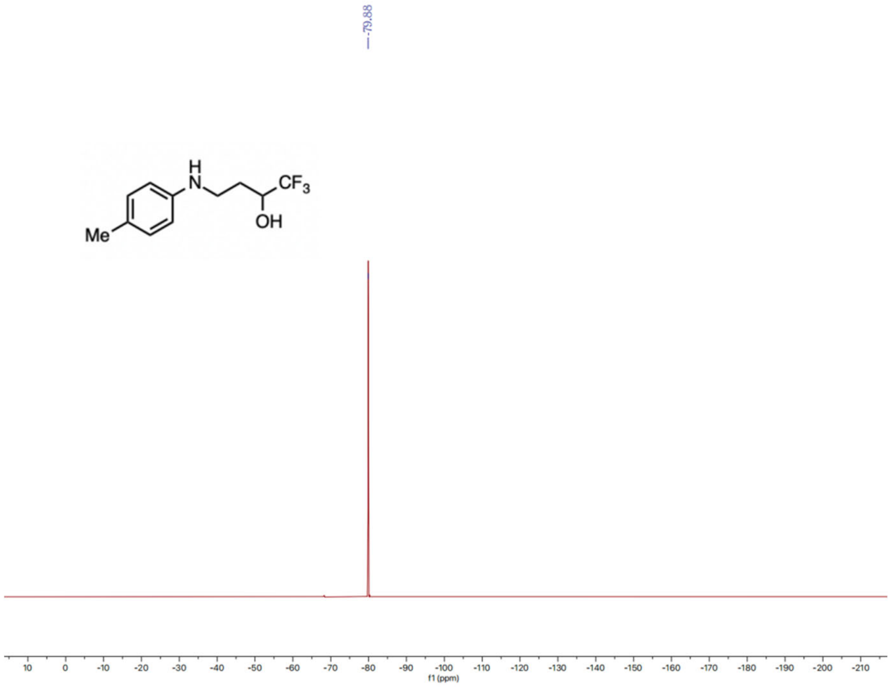

^{19}F NMR spectrum (471 MHz, Chloroform-*d*) of compound **26**

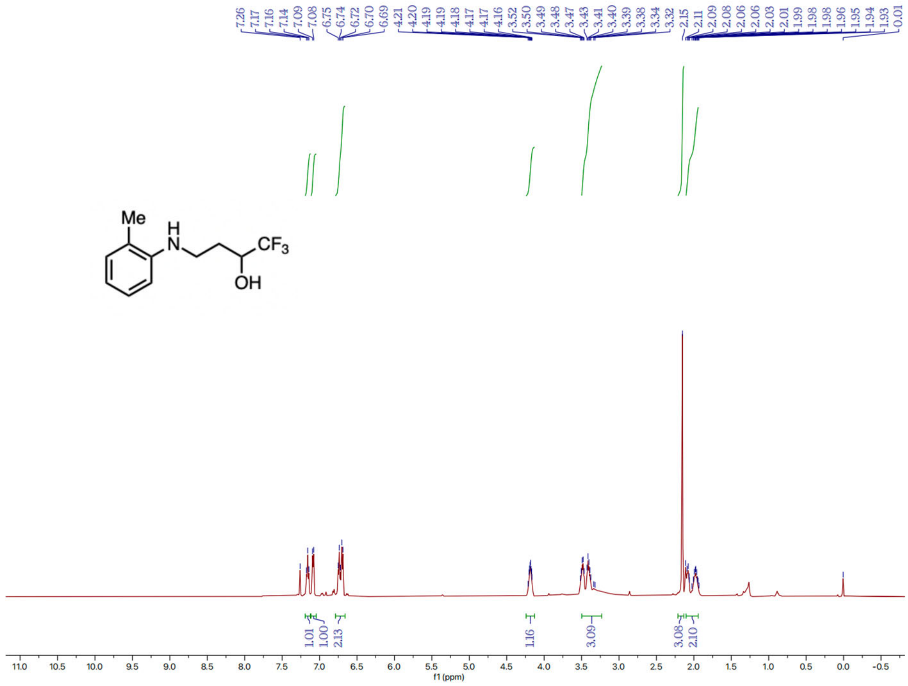

^{1}H NMR spectrum (500 MHz, Chloroform-*d*) of compound **27**

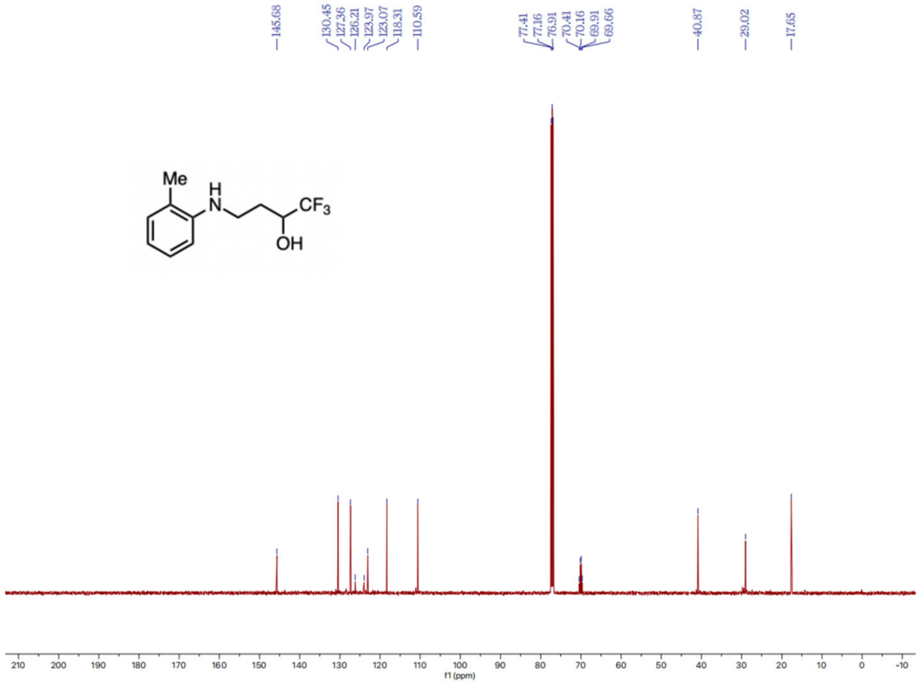

^{13}C NMR spectrum (126 MHz, Chloroform-*d*) of compound **27**

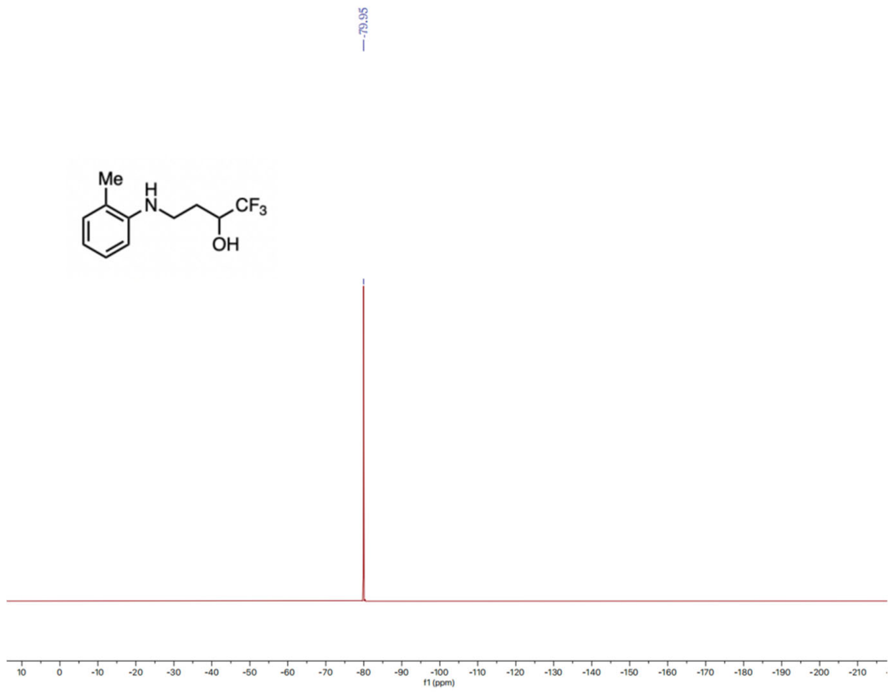

^{19}F NMR spectrum (471 MHz, Chloroform-*d*) of compound **27**

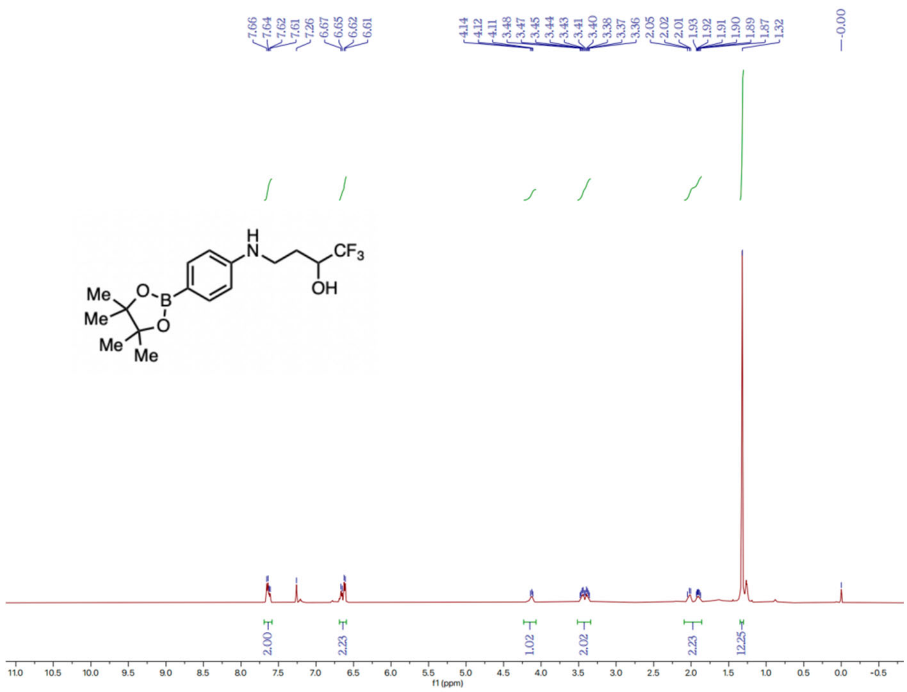

^{1}H NMR spectrum (500 MHz, Chloroform-*d*) of compound **28**

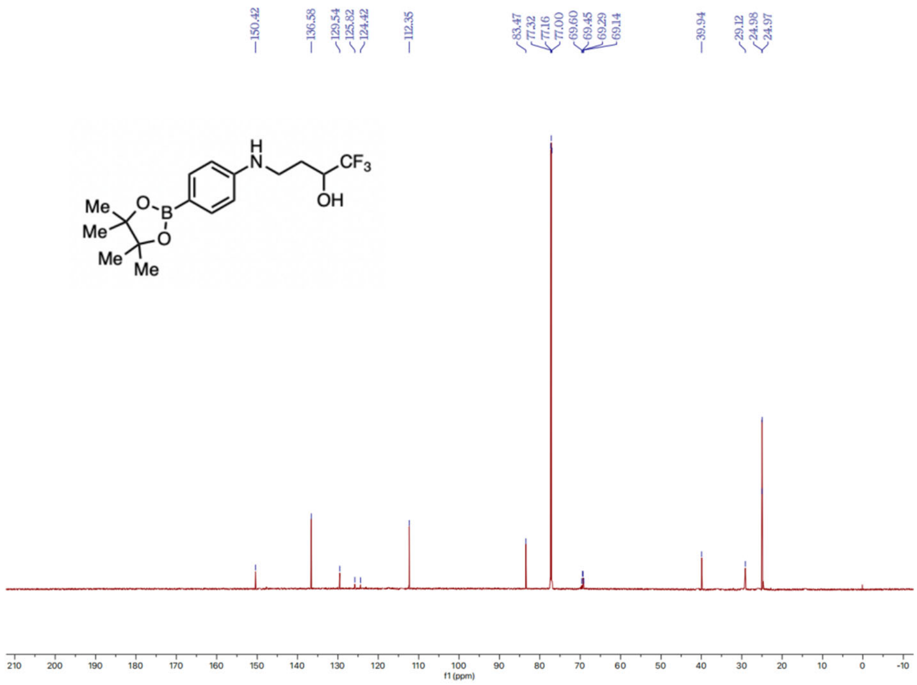

^{13}C NMR spectrum (201 MHz, Chloroform-*d*) of compound **28**

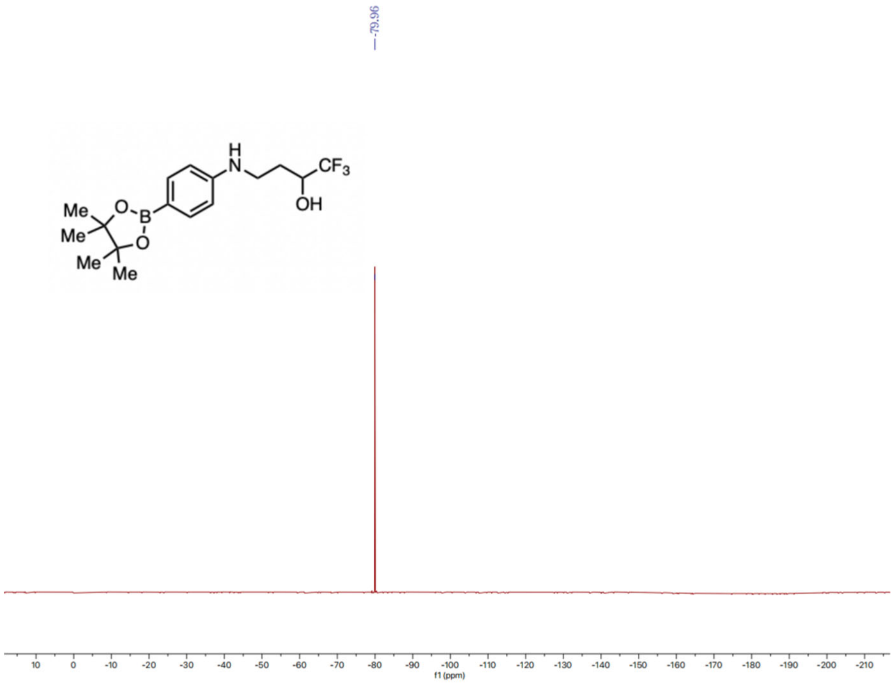

^{19}F NMR spectrum (471 MHz, Chloroform-*d*) of compound **28**

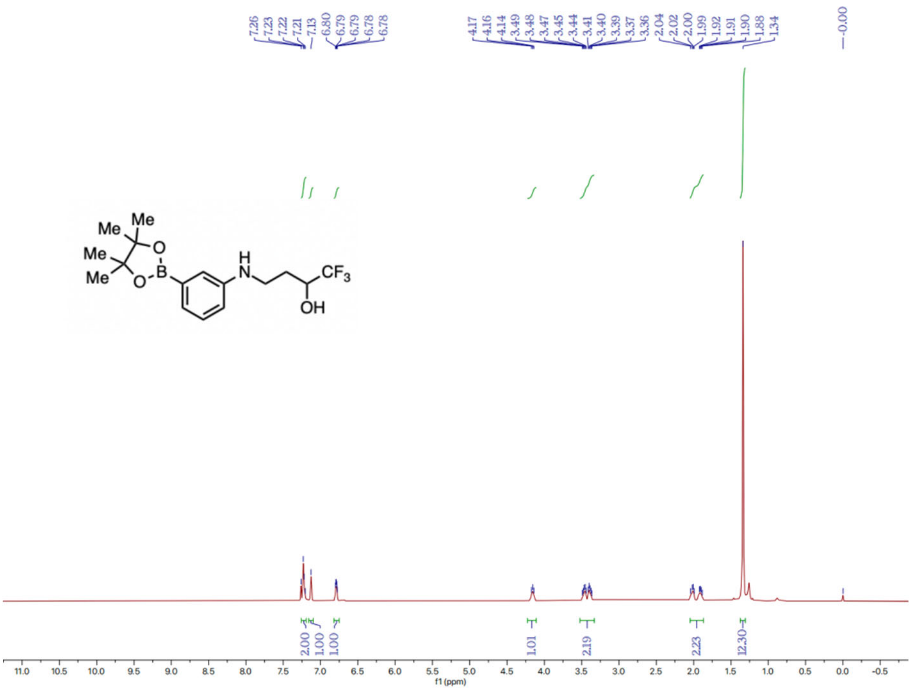

^{1}H NMR spectrum (500 MHz, Chloroform-*d*) of compound **29**

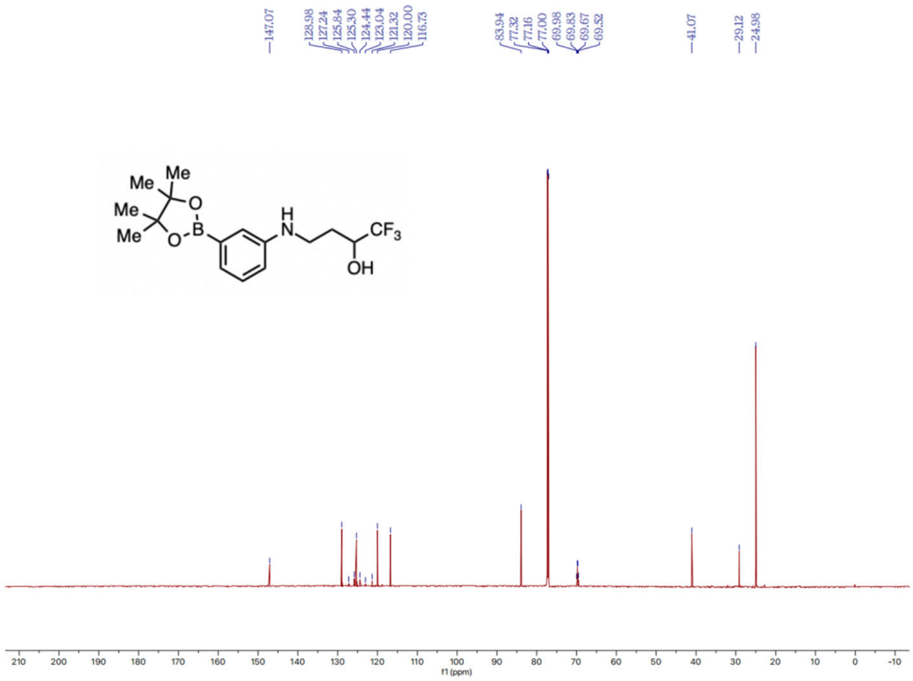

^{13}C NMR spectrum (201 MHz, Chloroform-*d*) of compound **29**

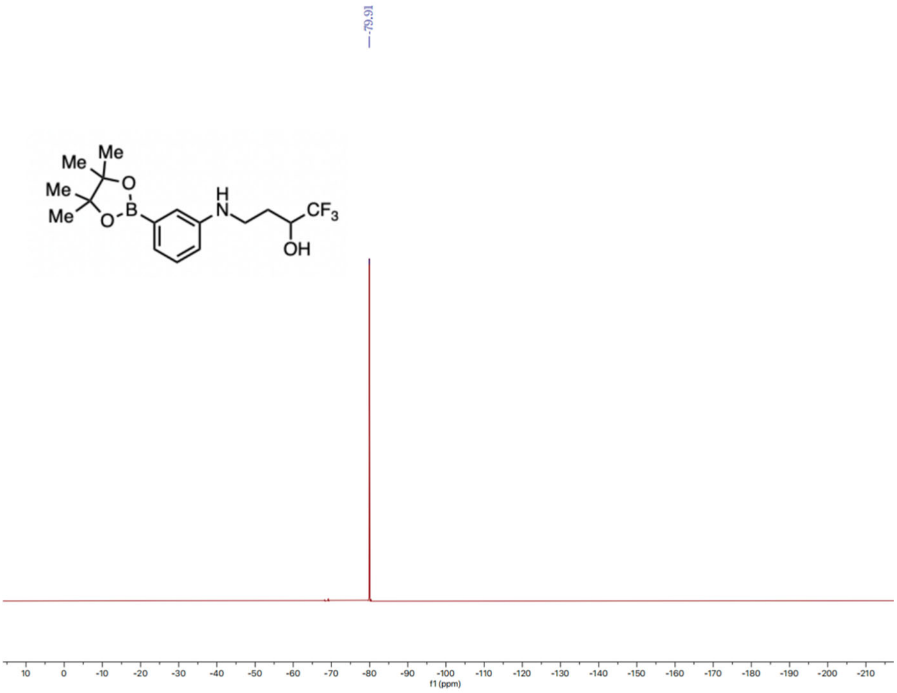

^{19}F NMR spectrum (471 MHz, Chloroform-*d*) of compound **29**

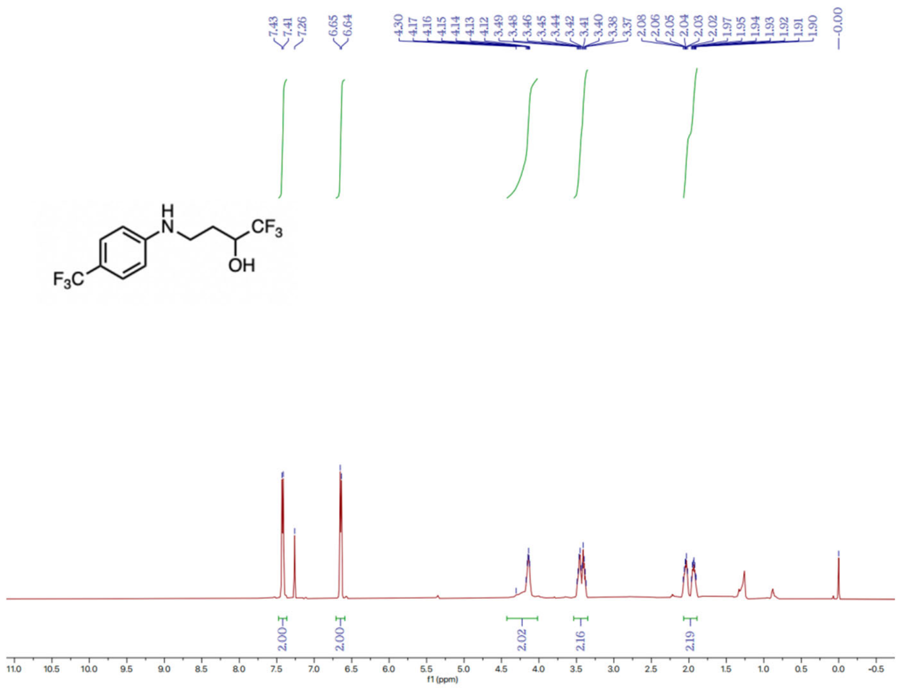

^{1}H NMR spectrum (500 MHz, Chloroform-*d*) of compound **30**

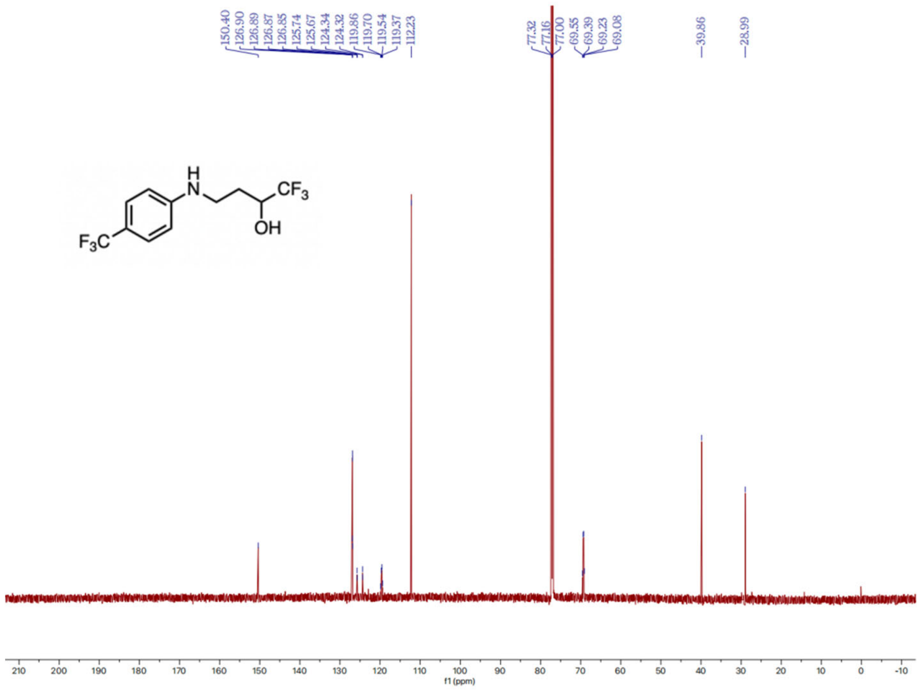

^{13}C NMR spectrum (210 MHz, Chloroform-*d*) of compound **30**

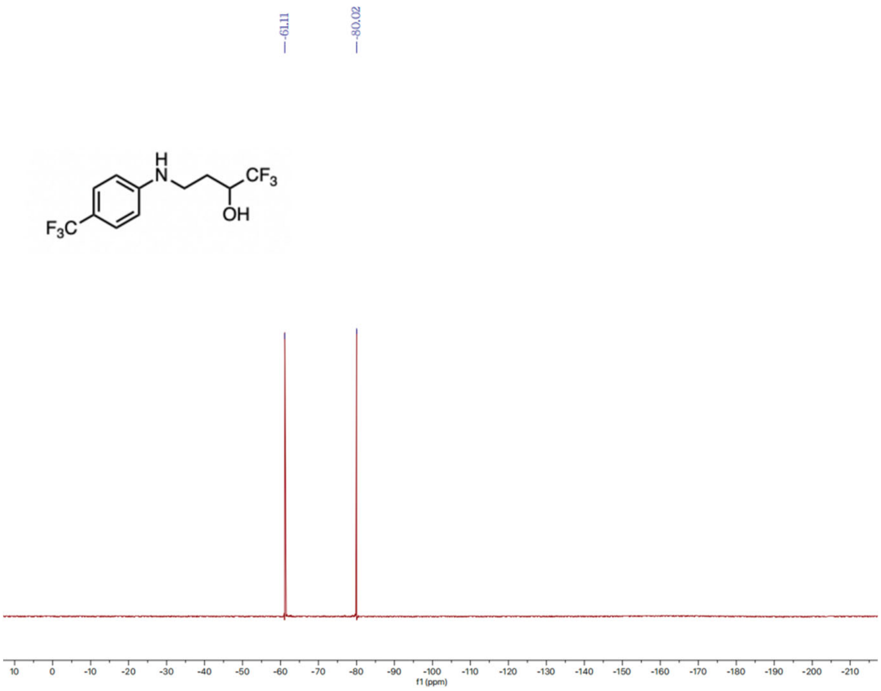

^{19}F NMR spectrum (471 MHz, Chloroform-*d*) of compound **30**

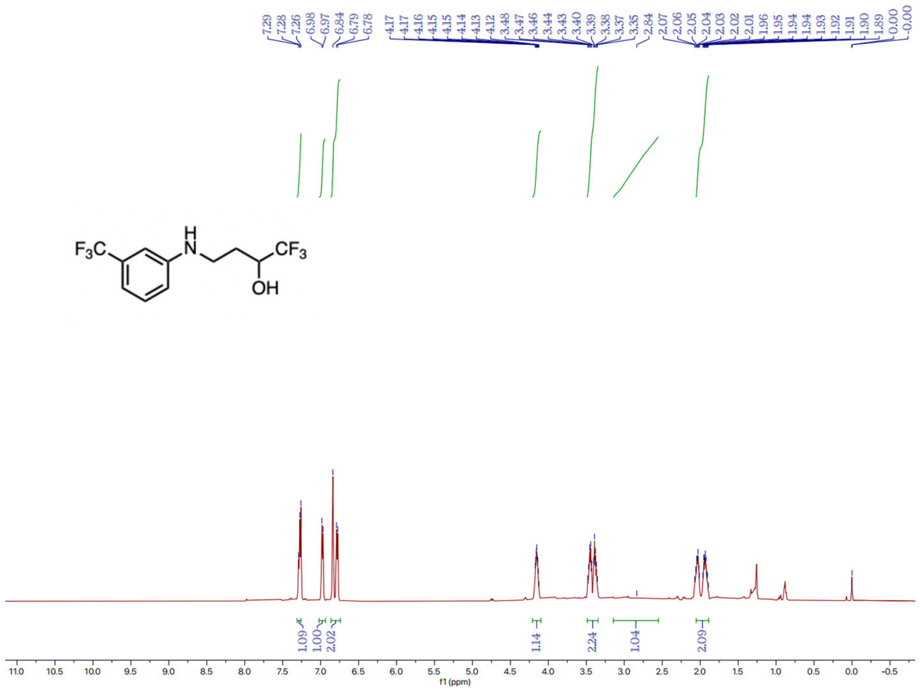

^{1}H NMR spectrum (500 MHz, Chloroform-*d*) of compound **31**

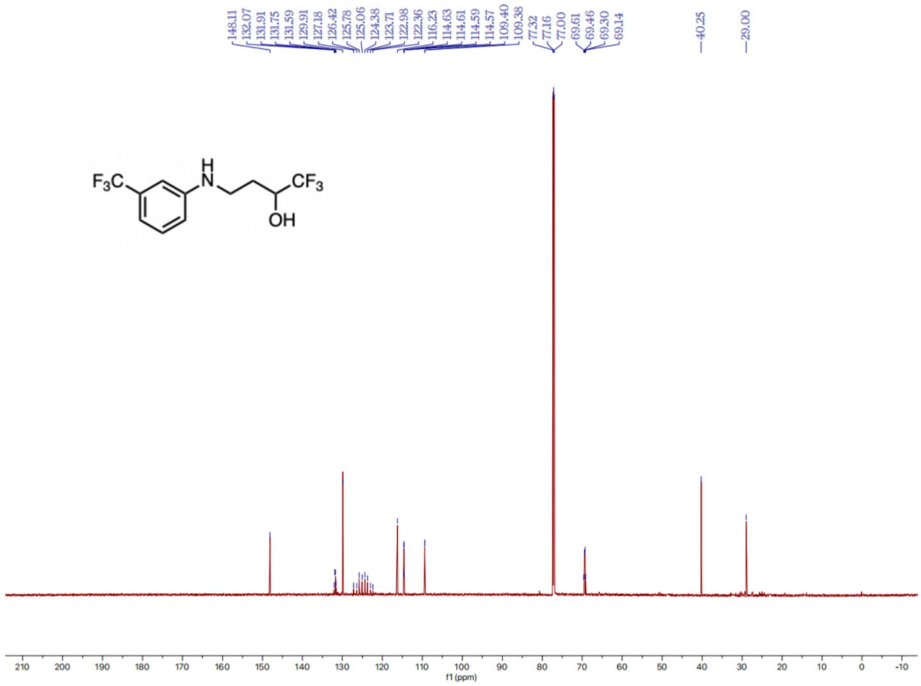

^{13}C NMR spectrum (201 MHz, Chloroform-*d*) of compound **31**

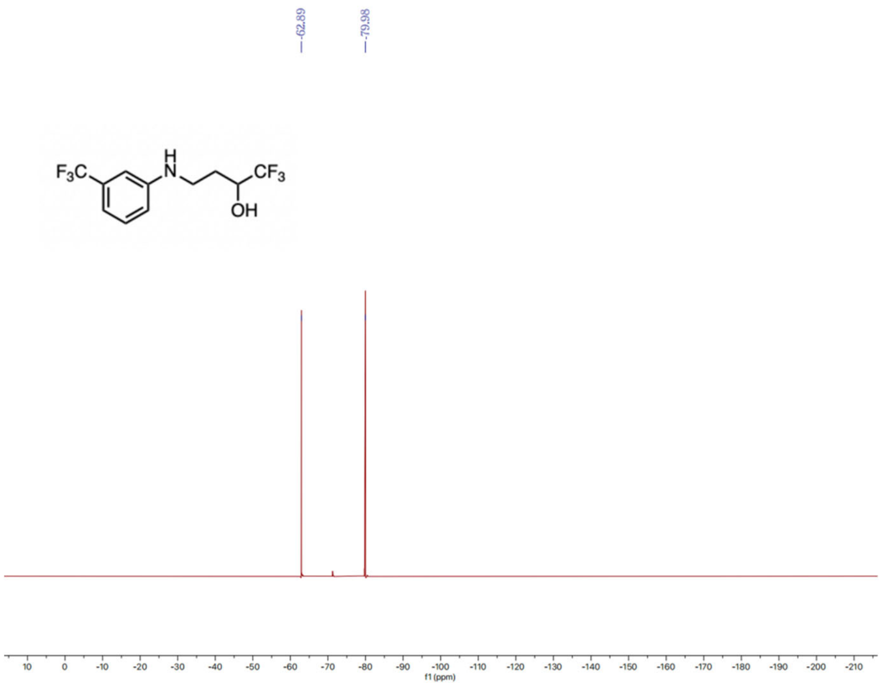

^{19}F NMR spectrum (471 MHz, Chloroform-*d*) of compound **31**

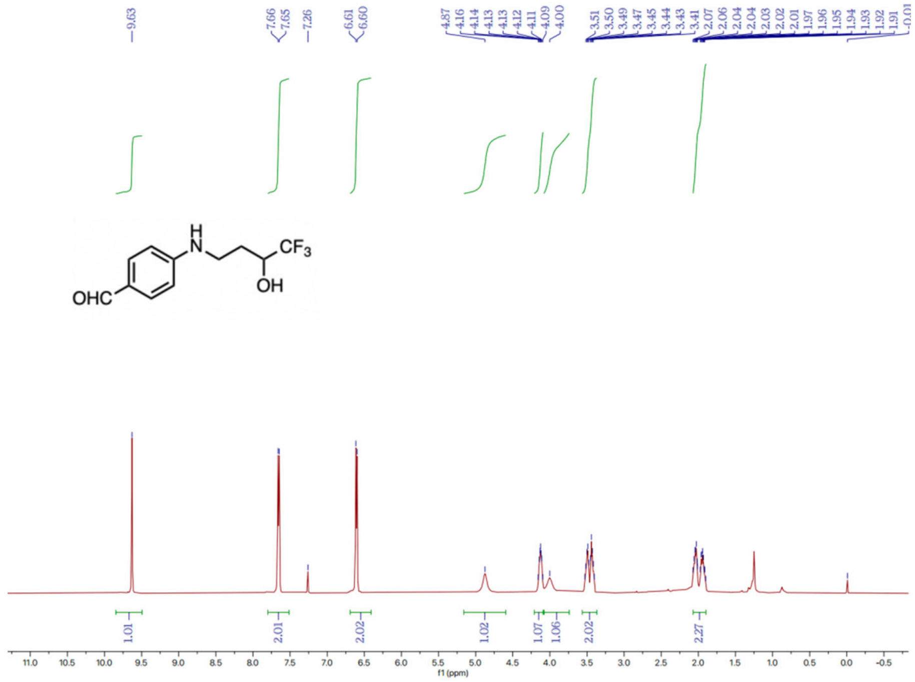

^{1}H NMR spectrum (500 MHz, Chloroform-*d*) of compound **32**

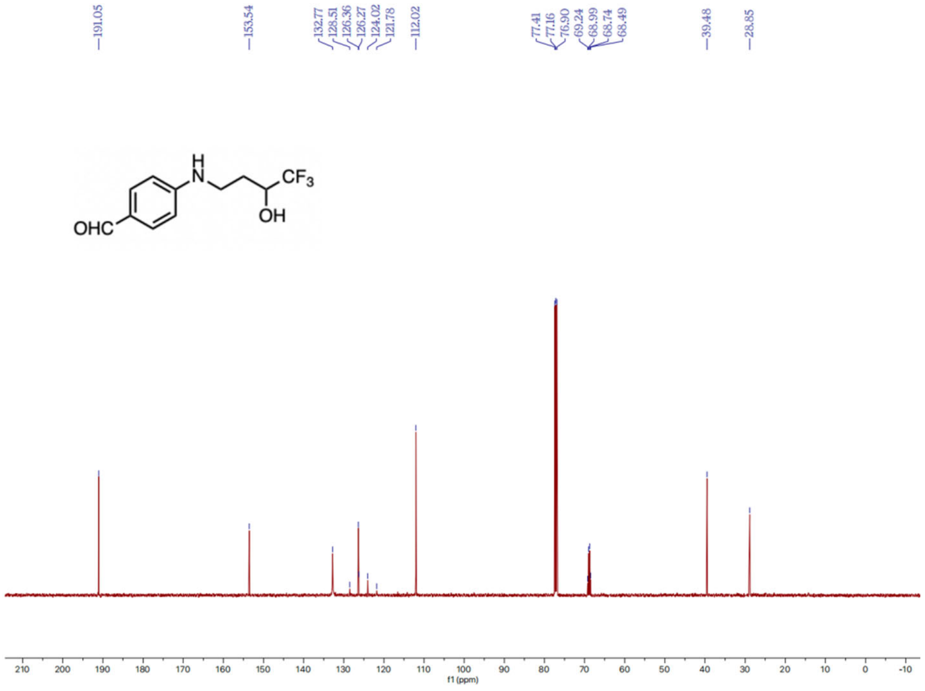

^{13}C NMR spectrum (126 MHz, Chloroform-*d*) of compound **32**

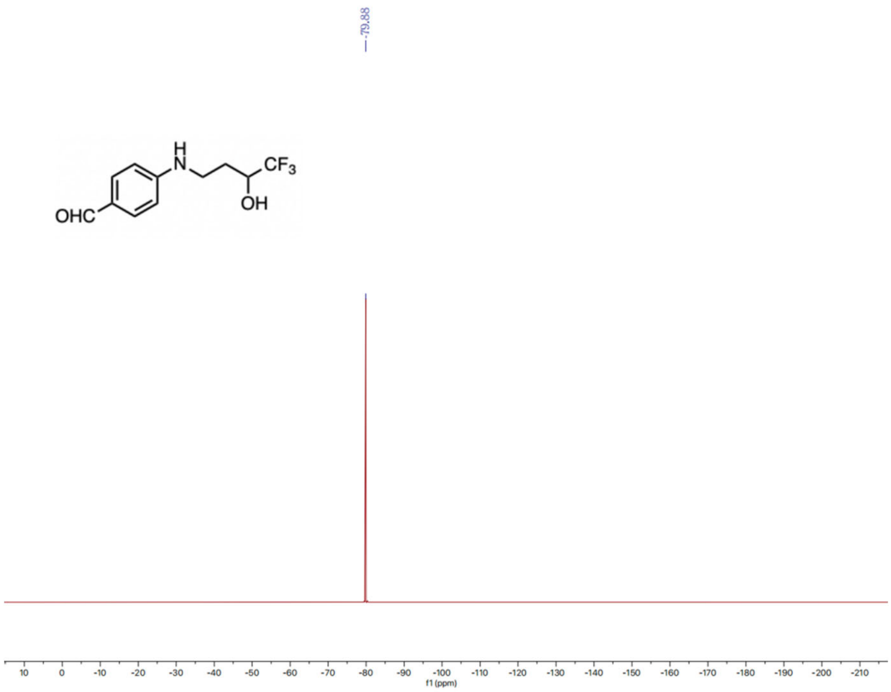

^{19}F NMR spectrum (471 MHz, Chloroform-*d*) of compound **32**

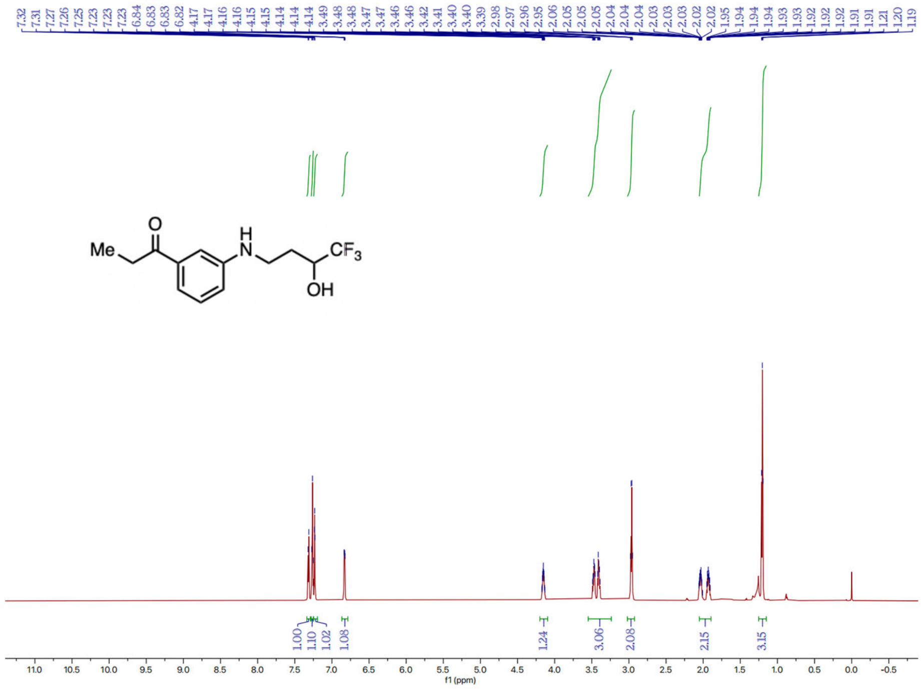

^{1}H NMR spectrum (800 MHz, Chloroform-*d*) of compound **33**

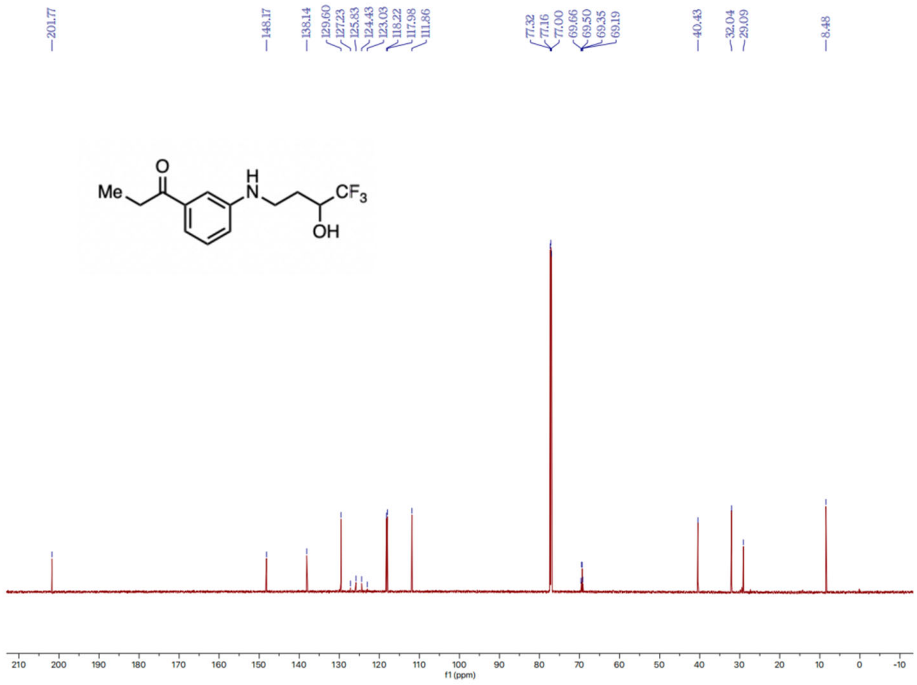

^{13}C NMR spectrum (201 MHz, Chloroform-*d*) of compound **33**

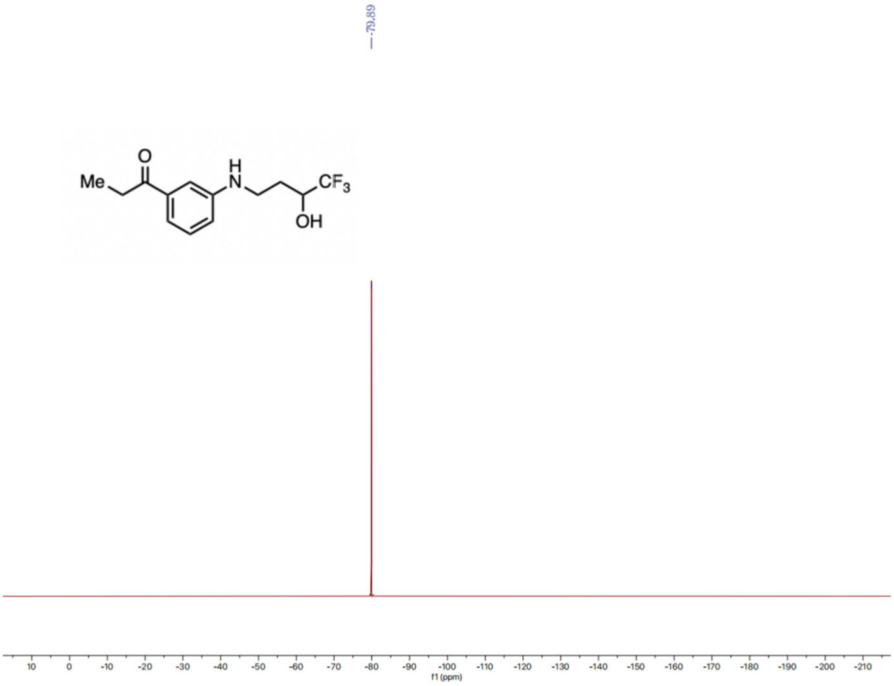

^{19}F NMR spectrum (471 MHz, Chloroform-*d*) of compound **33**

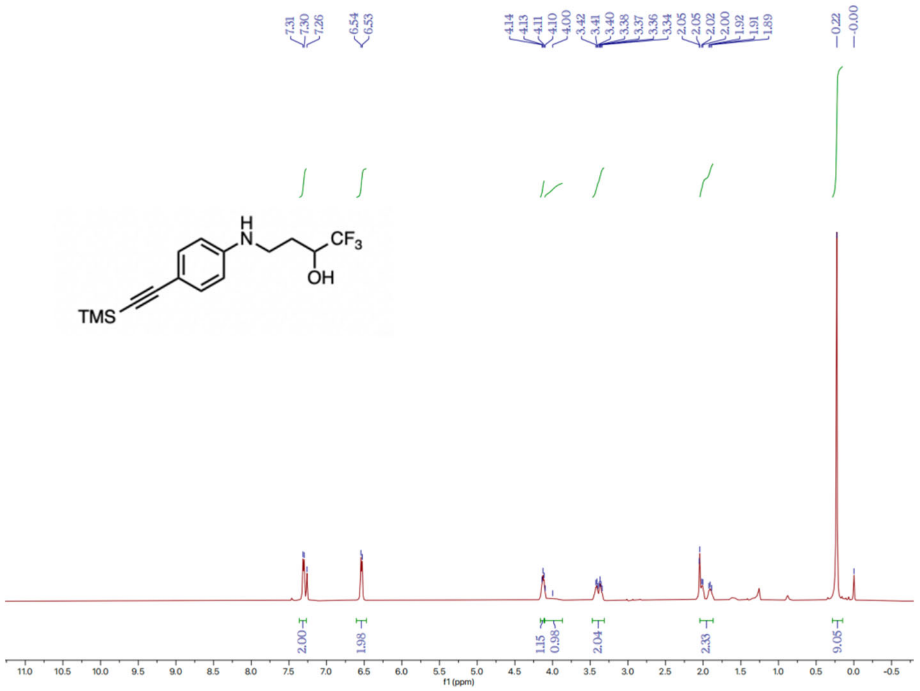

^{1}H NMR spectrum (500 MHz, Chloroform-*d*) of compound **34**

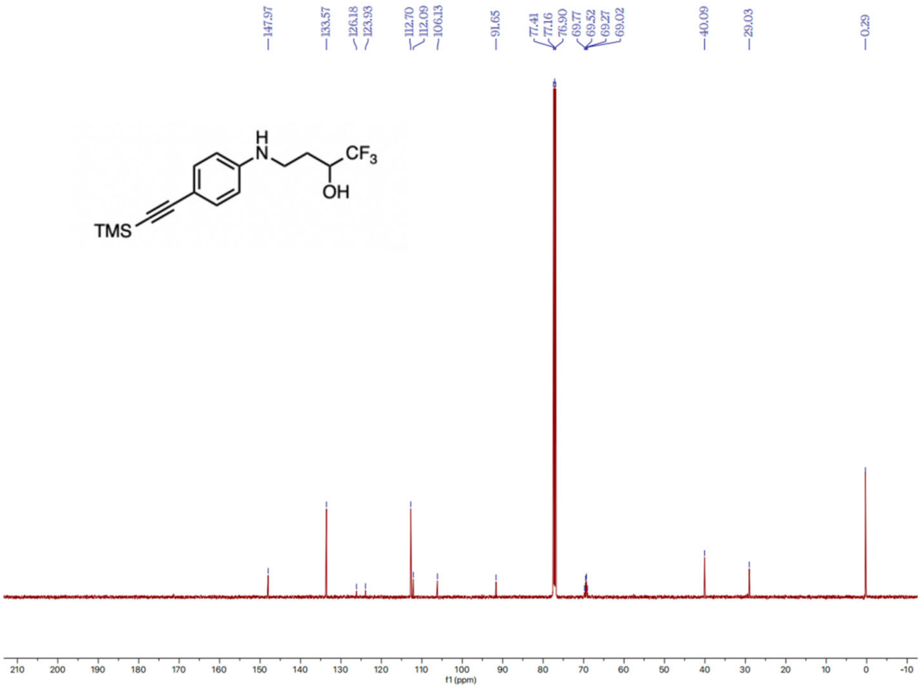

^{13}C NMR spectrum (126 MHz, Chloroform-*d*) of compound **34**

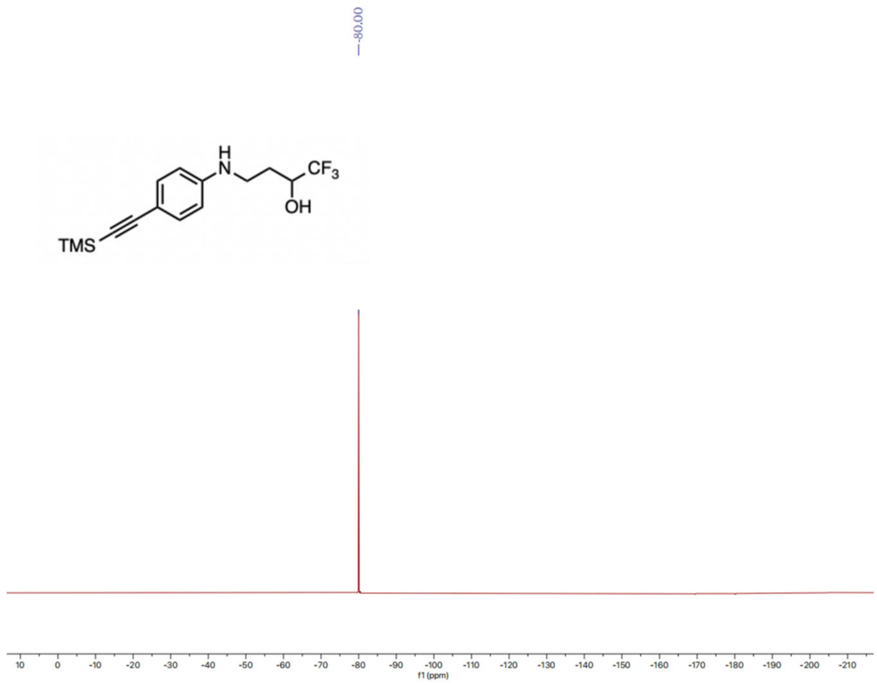

^{19}F NMR spectrum (471 MHz, Chloroform-*d*) of compound **34**

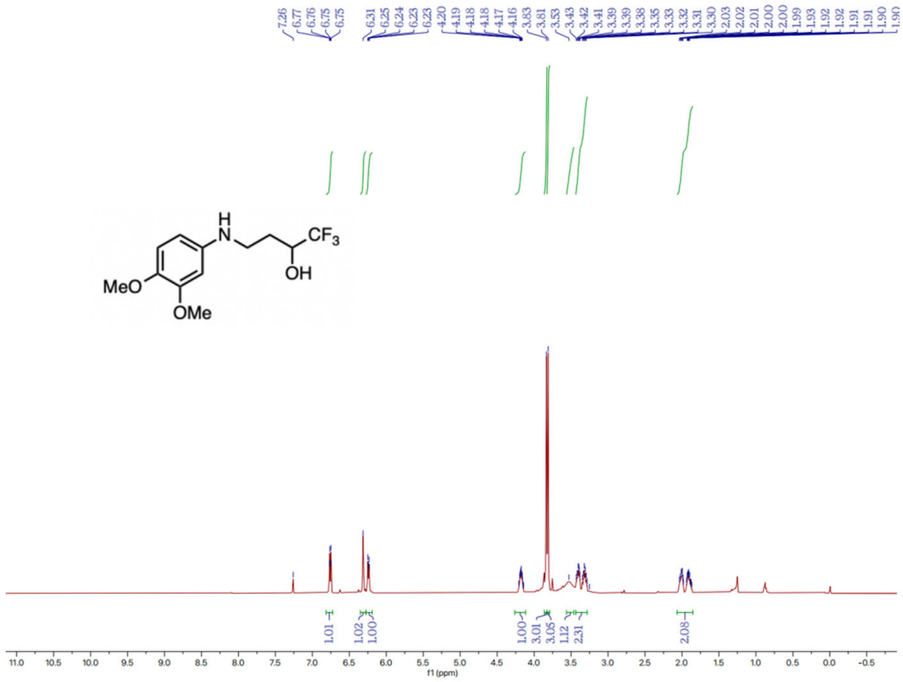

^{1}H NMR spectrum (500 MHz, Chloroform-*d*) of compound **35**

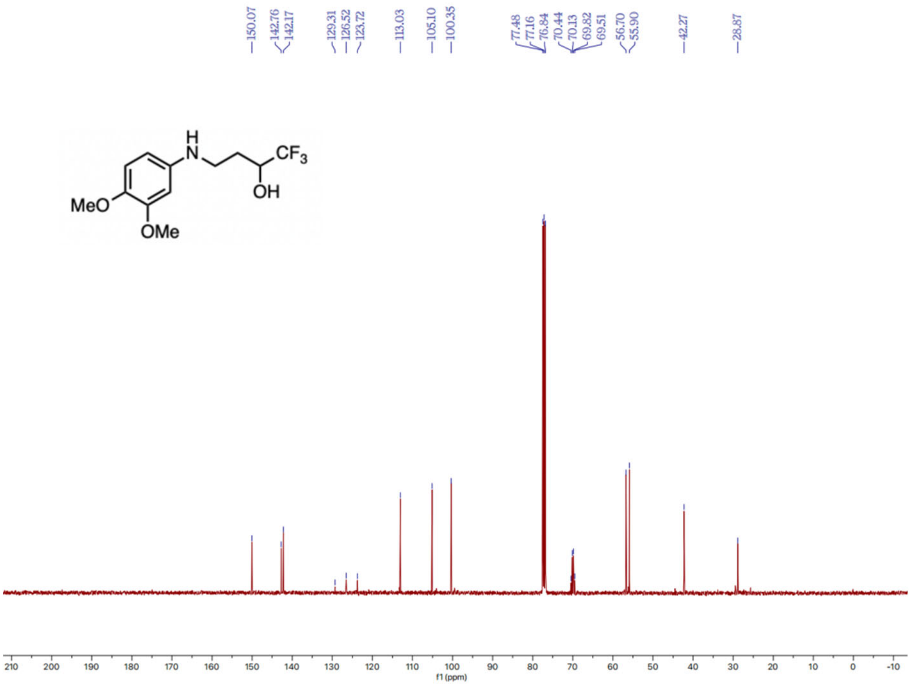

^{13}C NMR spectrum (101 MHz, Chloroform-*d*) of compound **35**

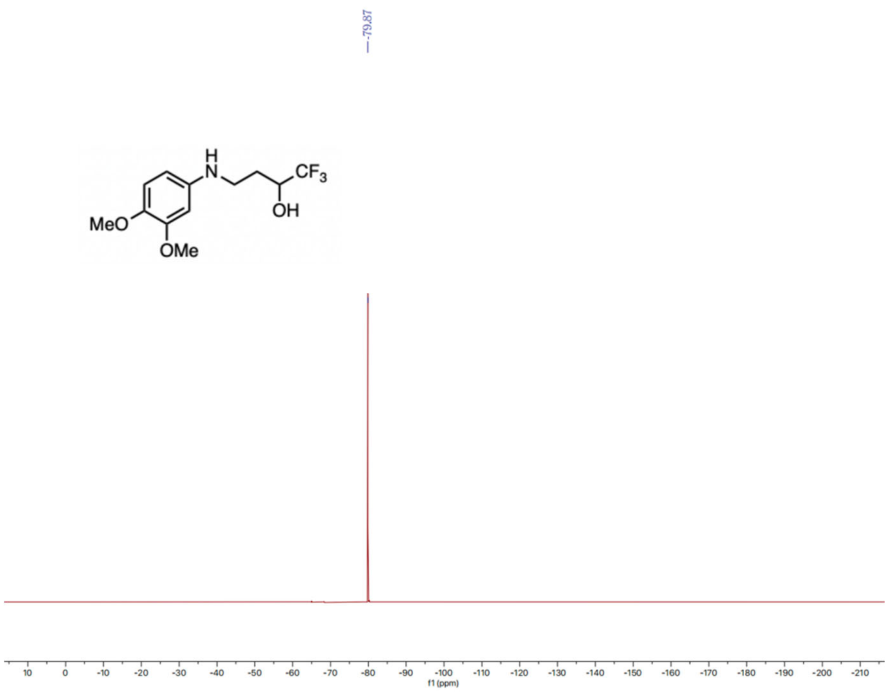

^{19}F NMR spectrum (471 MHz, Chloroform-*d*) of compound **35**

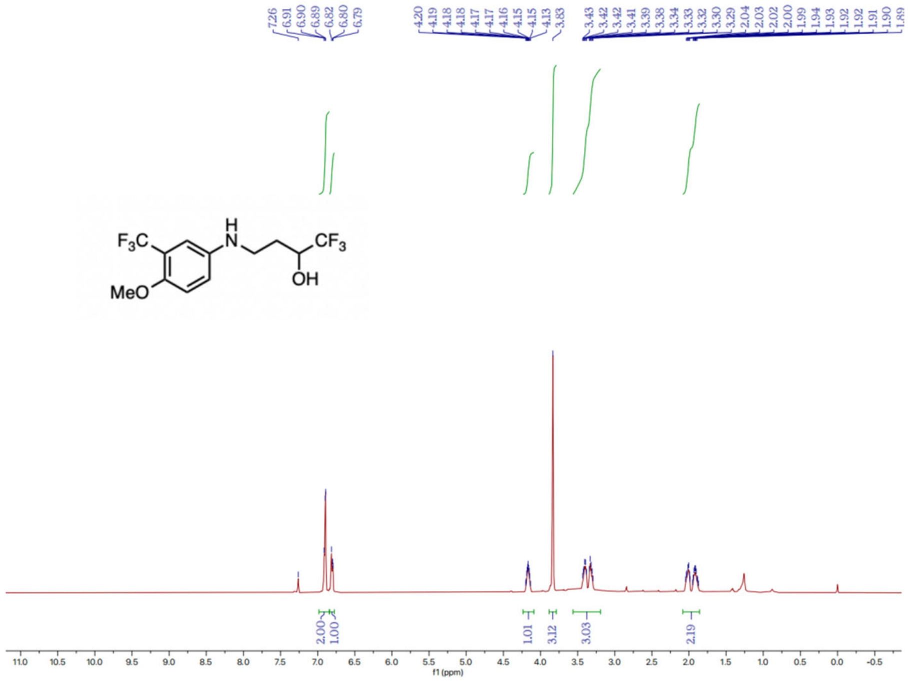

^{1}H NMR spectrum (500 MHz, Chloroform-*d*) of compound **36**

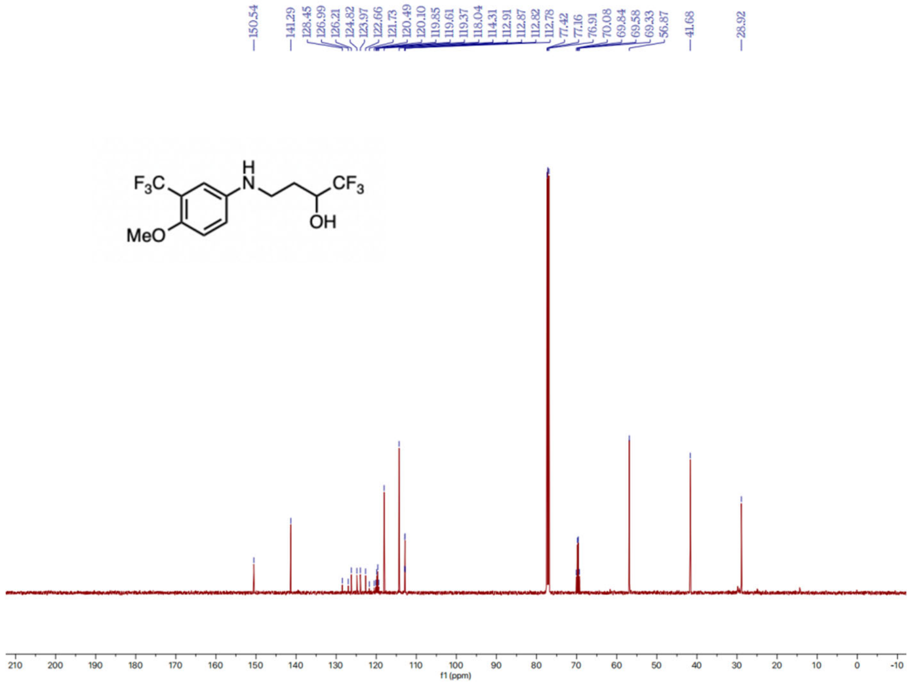

^{13}C NMR spectrum (126 MHz, Chloroform-*d*) of compound **36**

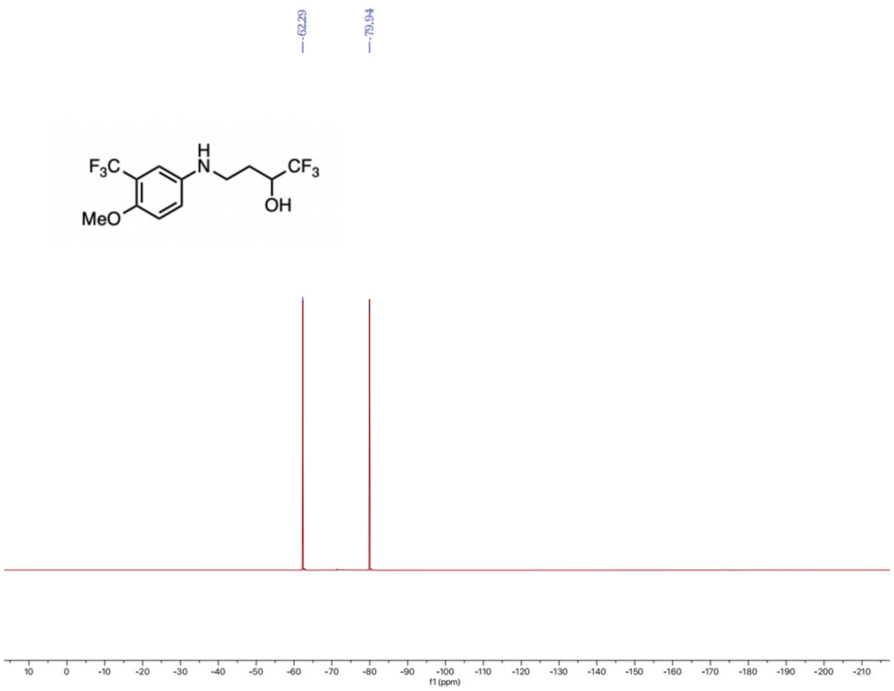

^{19}F NMR spectrum (471 MHz, Chloroform-*d*) of compound **36**

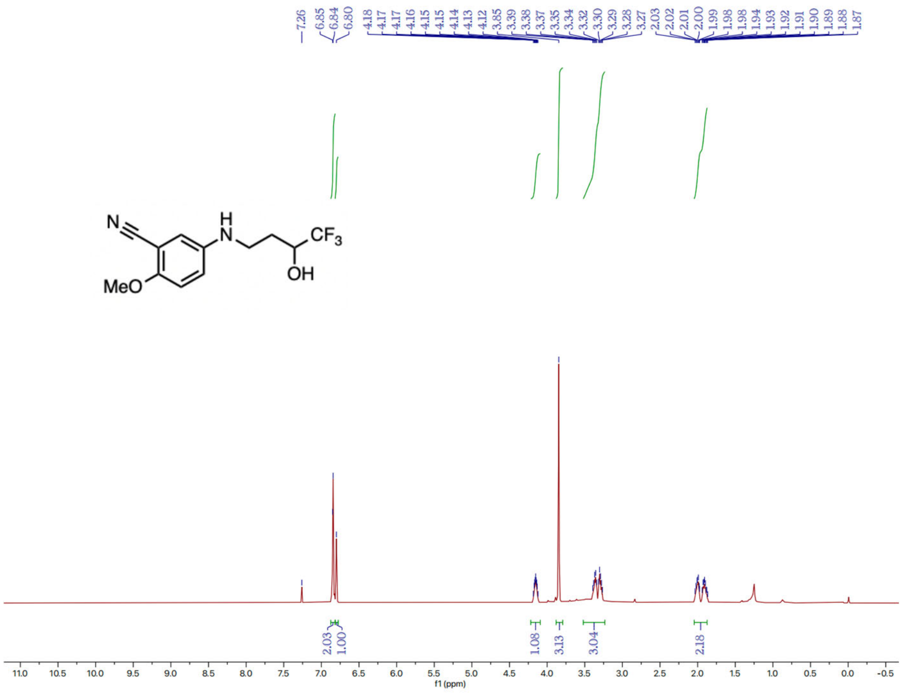

^{1}H NMR spectrum (500 MHz, Chloroform-*d*) of compound **37**

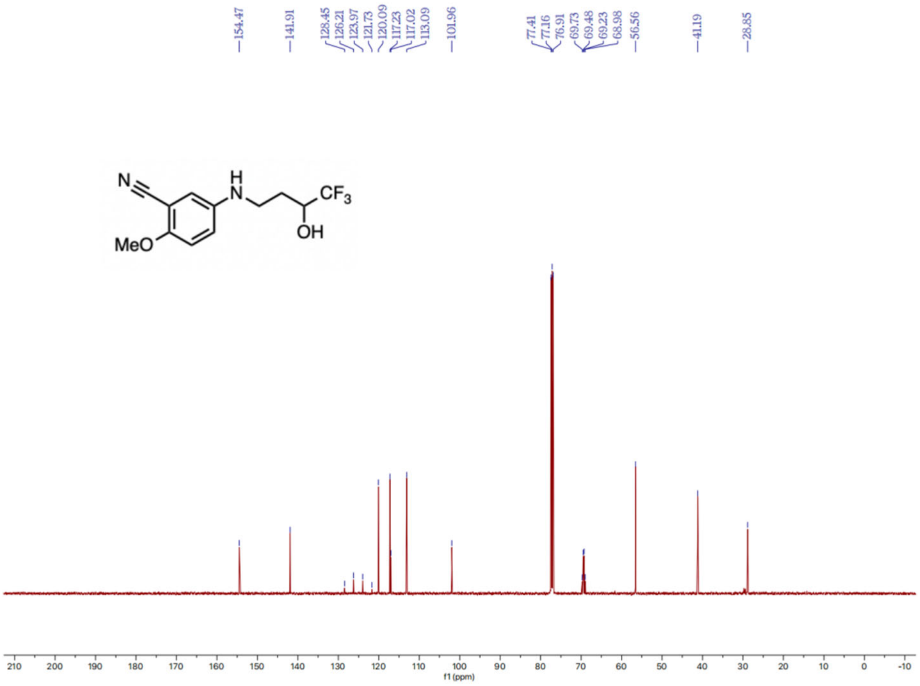

^{13}C NMR spectrum (126 MHz, Chloroform-*d*) of compound **37**

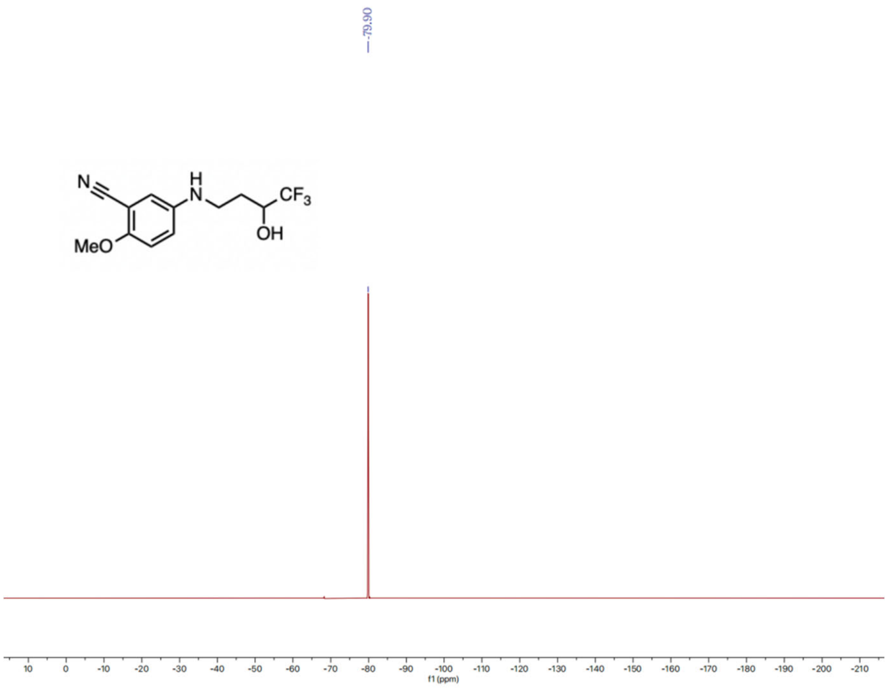

^{19}F NMR spectrum (471 MHz, Chloroform-*d*) of compound **37**

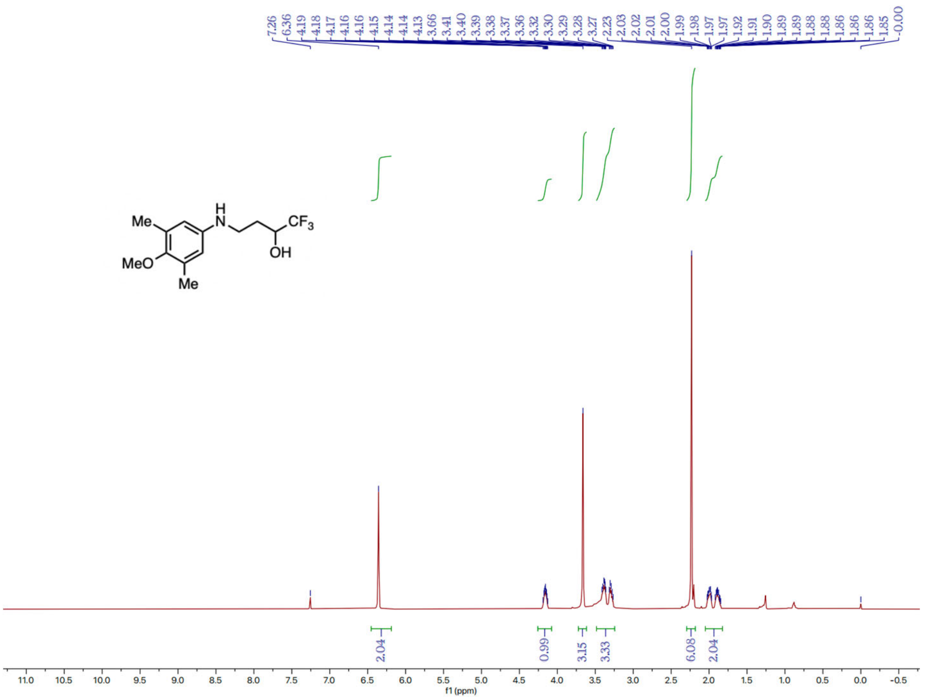

^{1}H NMR spectrum (500 MHz, Chloroform-*d*) of compound **38**

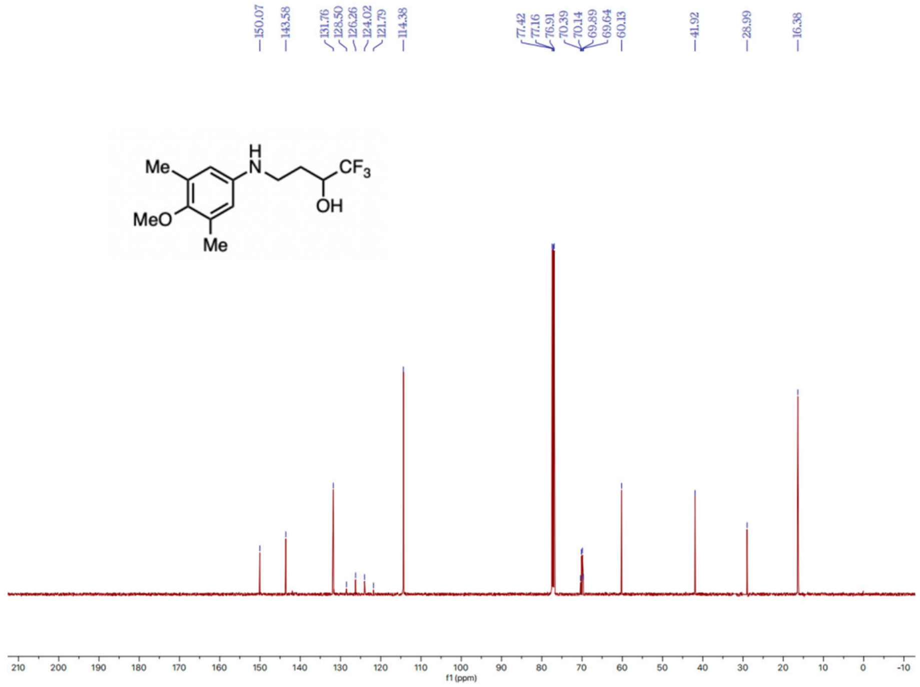

^{13}C NMR spectrum (126 MHz, Chloroform-*d*) of compound **38**

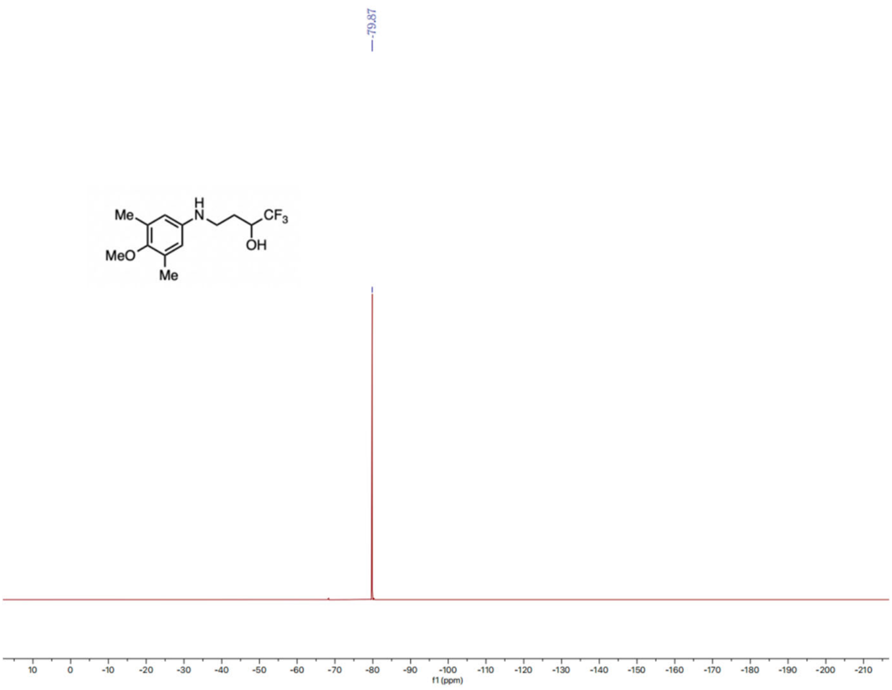

^{19}F NMR spectrum (471 MHz, Chloroform-*d*) of compound **38**

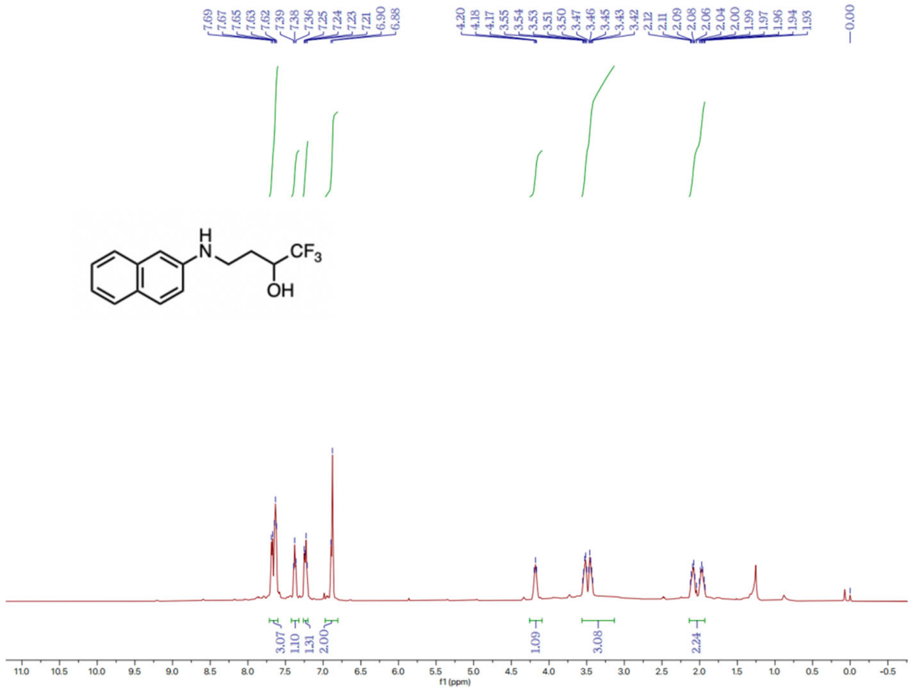

^{1}H NMR spectrum (500 MHz, Chloroform-*d*) of compound **39**

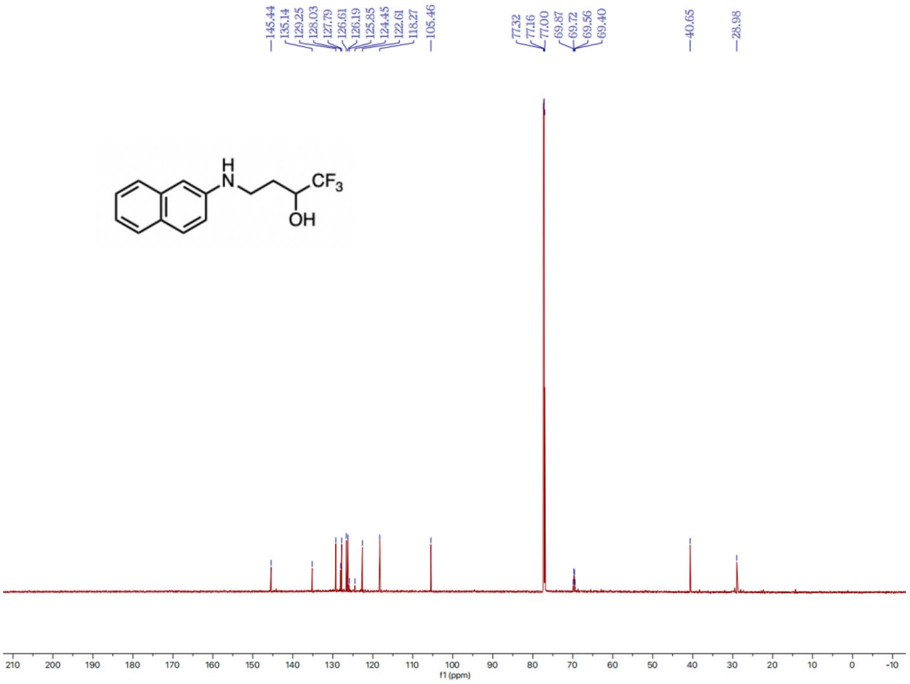

^{13}C NMR spectrum (201 MHz, Chloroform-*d*) of compound **39**

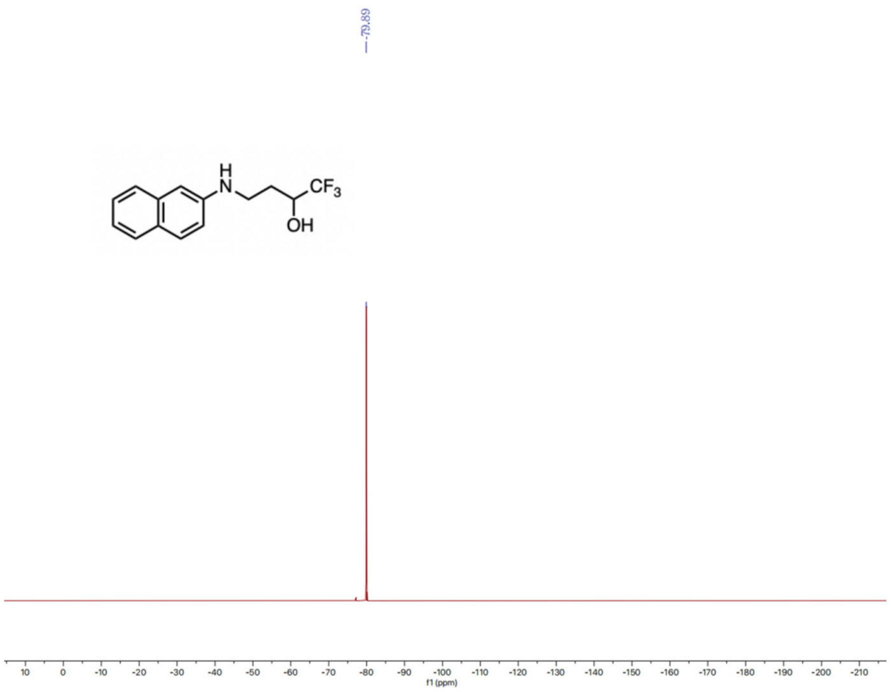

^{19}F NMR spectrum (471 MHz, Chloroform-*d*) of compound **39**

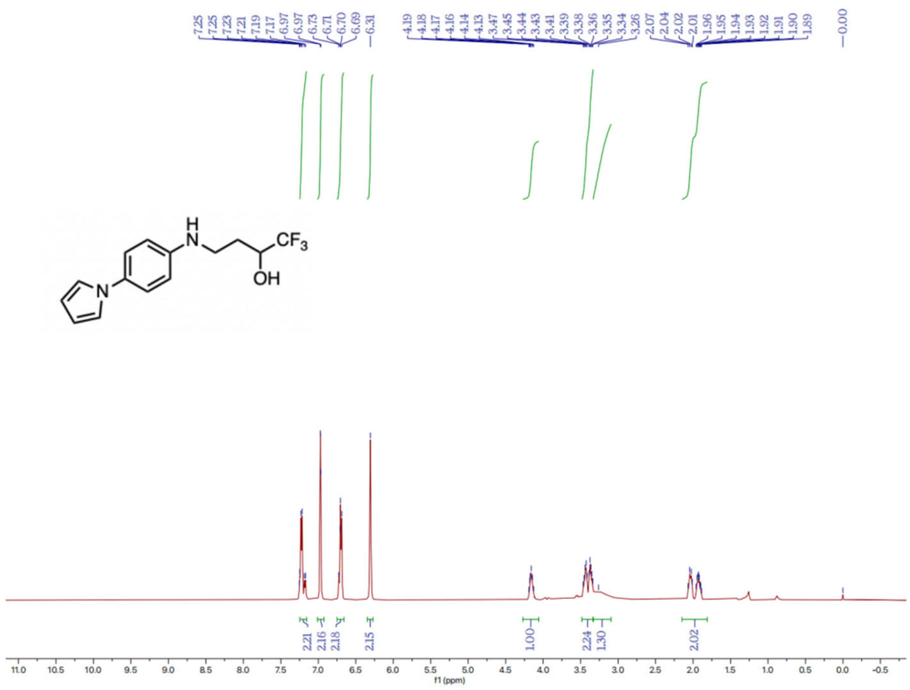

^{1}H NMR spectrum (500 MHz, Chloroform-*d*) of compound **40**

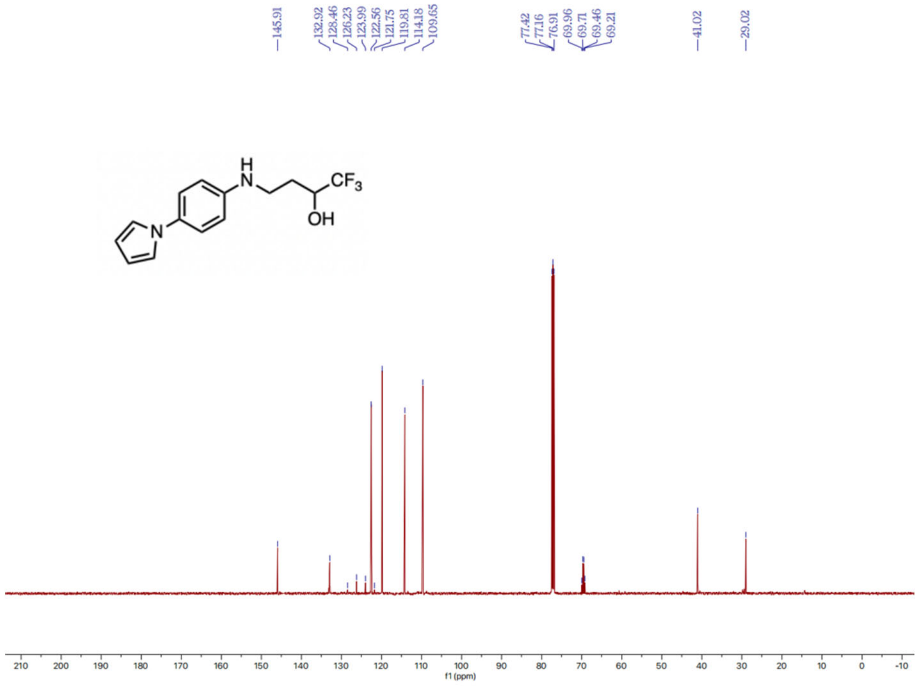

^{13}C NMR spectrum (126 MHz, Chloroform-*d*) of compound **40**

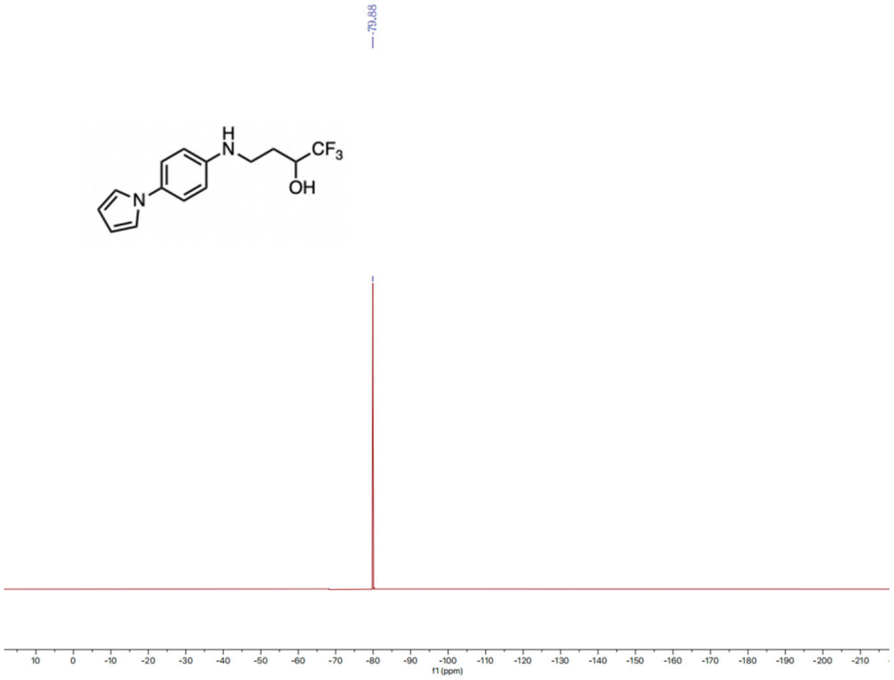

^{19}F NMR spectrum (471 MHz, Chloroform-*d*) of compound **40**

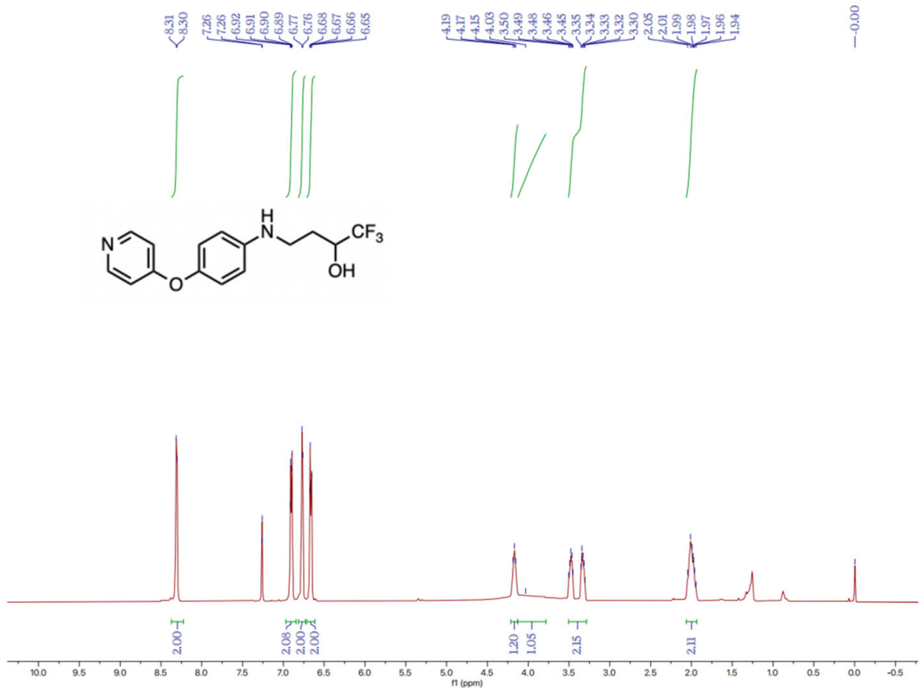

^{1}H NMR spectrum (500 MHz, Chloroform-*d*) of compound **41**

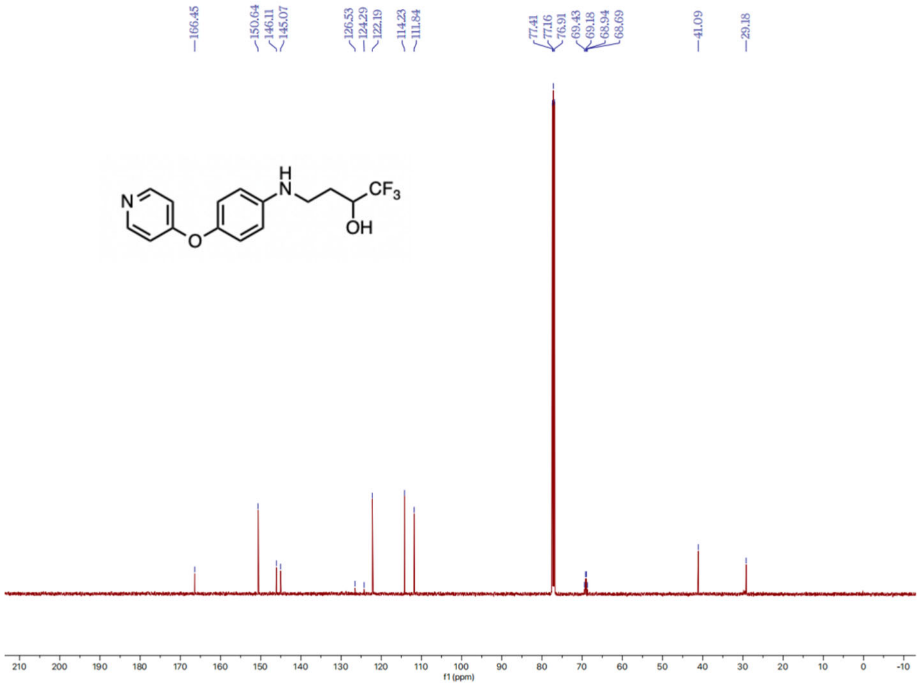

^{13}C NMR spectrum (126 MHz, Chloroform-*d*) of compound **41**

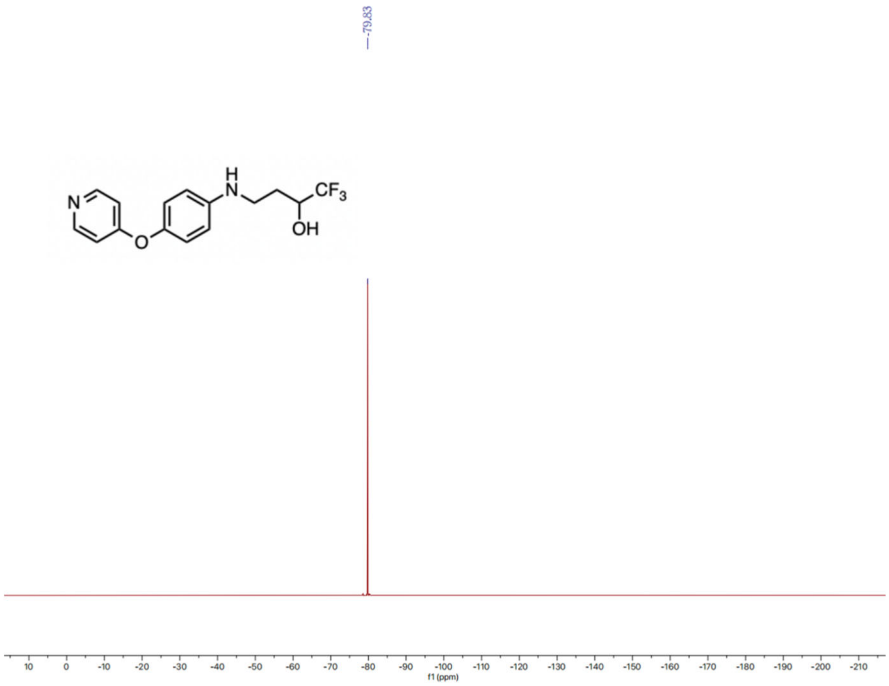

^{19}F NMR spectrum (471 MHz, Chloroform-*d*) of compound **41**

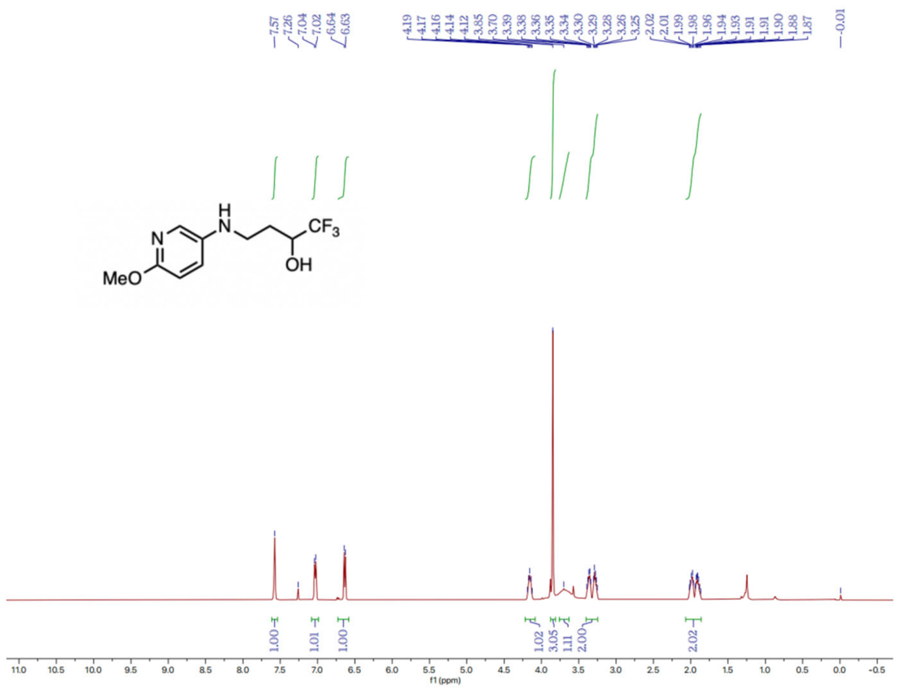

^{1}H NMR spectrum (500 MHz, Chloroform-*d*) of compound **42**

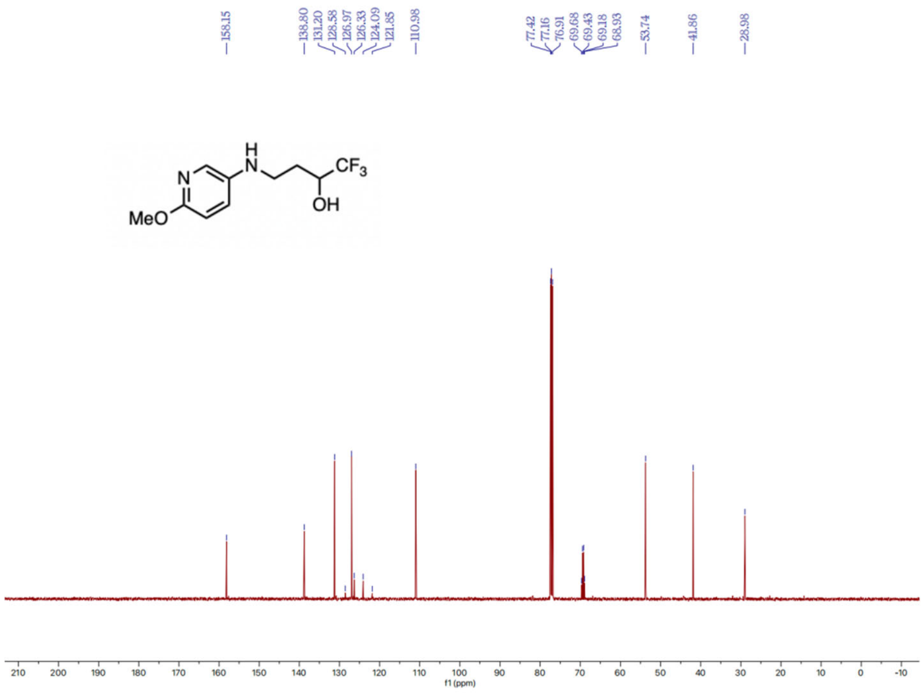

^{13}C NMR spectrum (126 MHz, Chloroform-*d*) of compound **42**

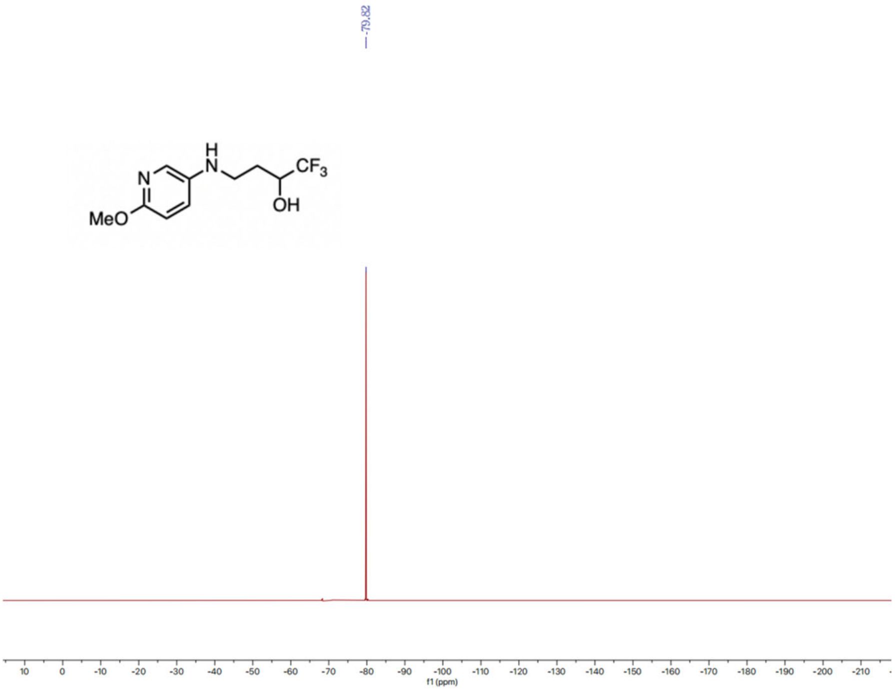

^{19}F NMR spectrum (471 MHz, Chloroform-*d*) of compound **42**

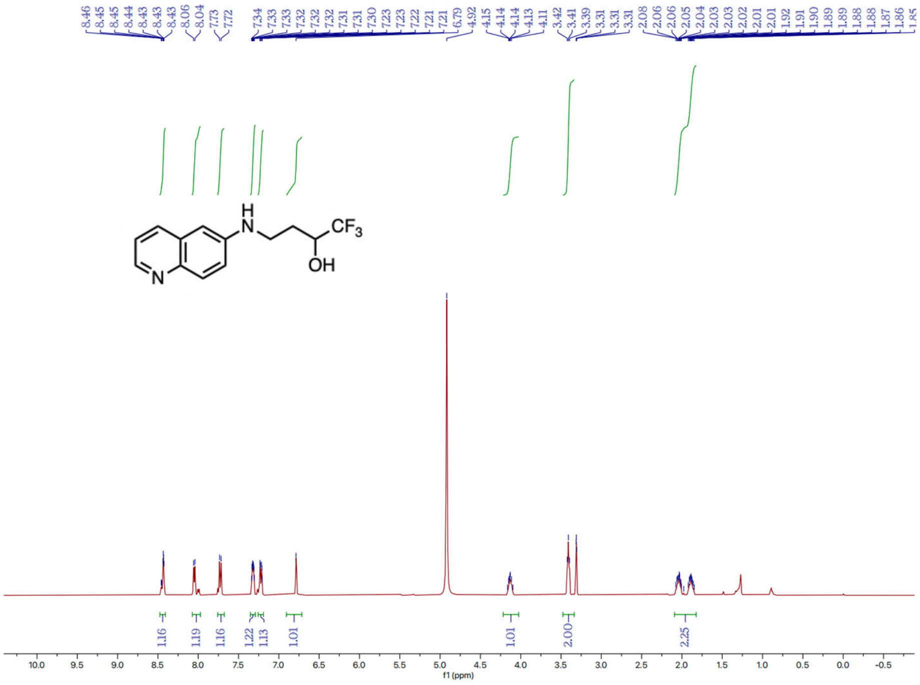

^{1}H NMR spectrum (500 MHz, CD3CD-*d4*) of compound **43**

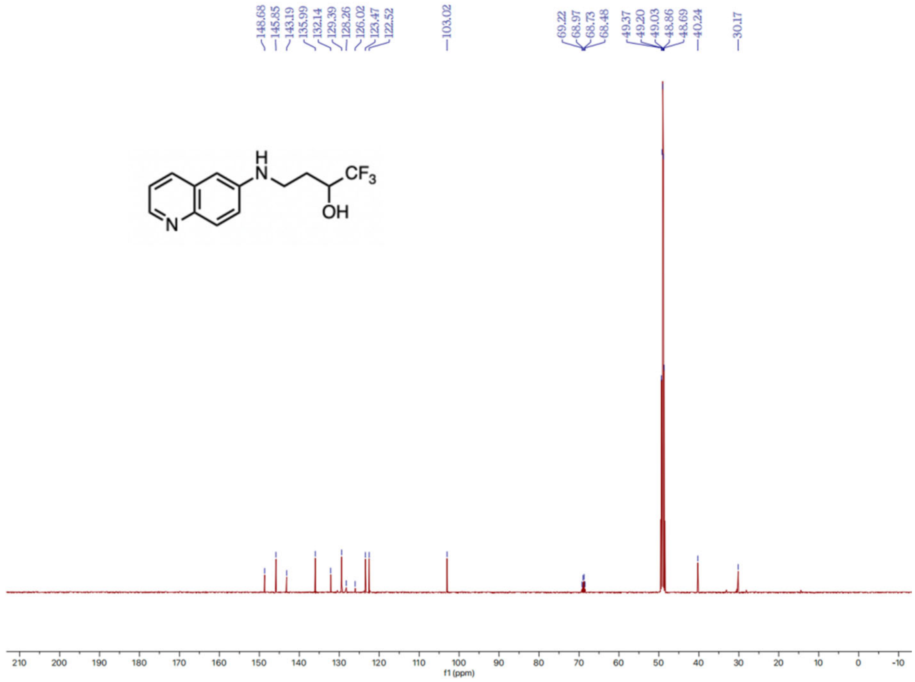

^{13}C NMR spectrum (126 MHz, CD3CD-*d4*) of compound **43**

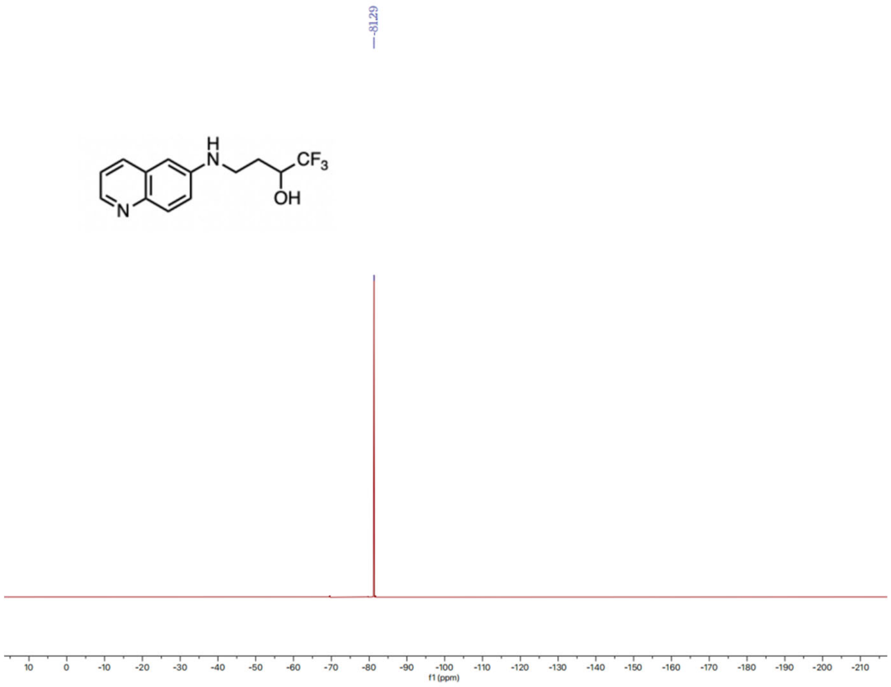

^{19}F NMR spectrum (471 MHz, CD_3CD-d_4) of compound **43**

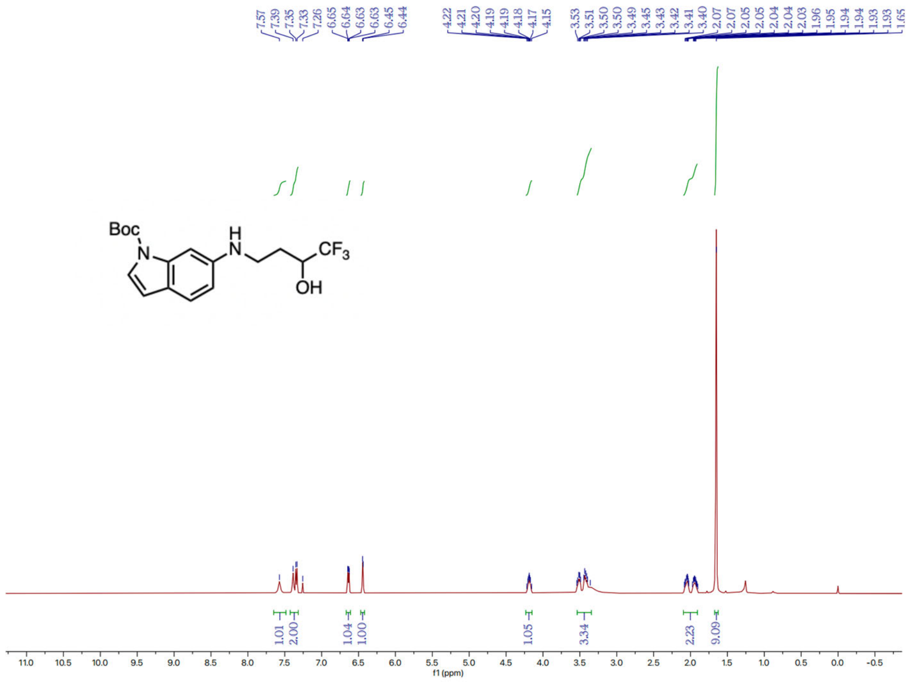

^{1}H NMR spectrum (500 MHz, Chloroform-*d*) of compound **44**

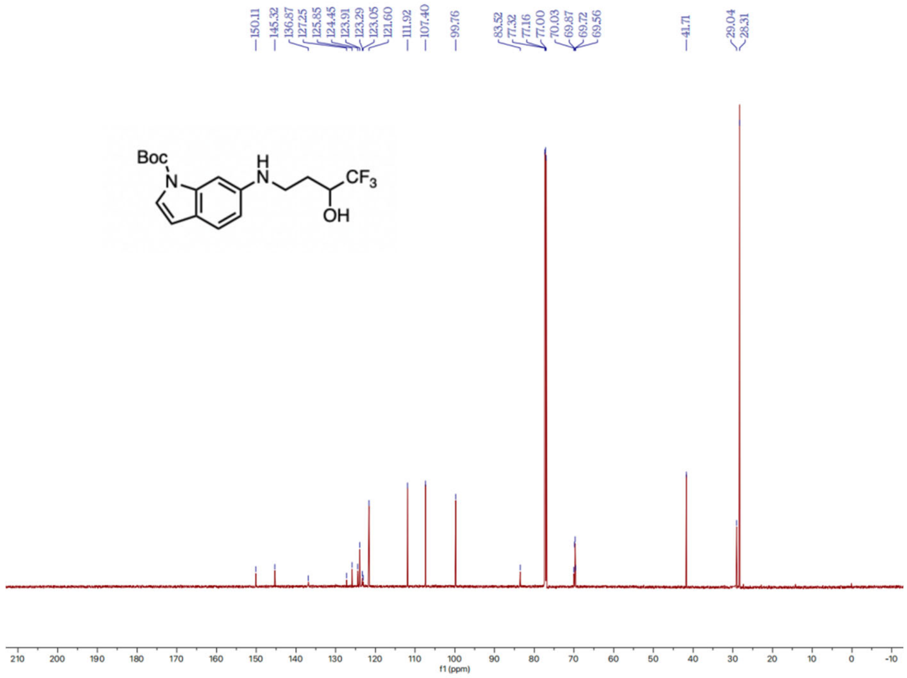

^{13}C NMR spectrum (201 MHz, Chloroform-*d*) of compound **44**

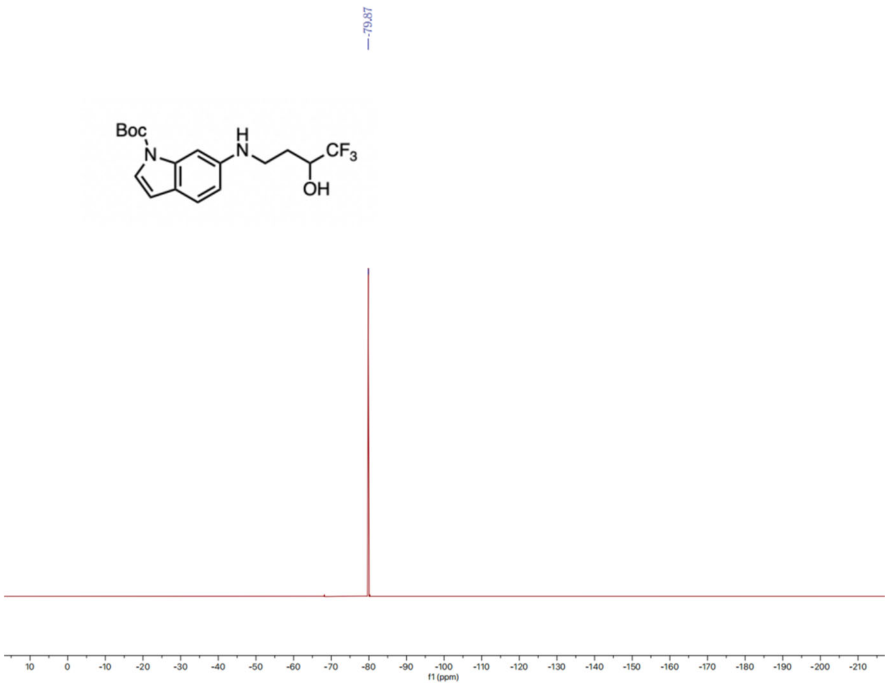

^{19}F NMR spectrum (471 MHz, Chloroform-*d*) of compound **44**

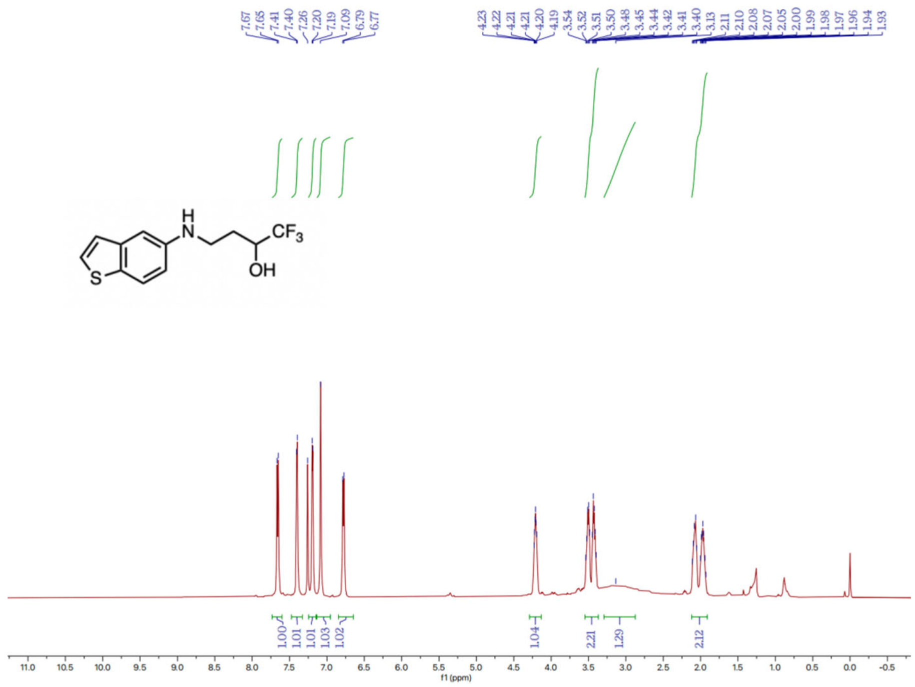

^{1}H NMR spectrum (500 MHz, Chloroform-*d*) of compound **45**

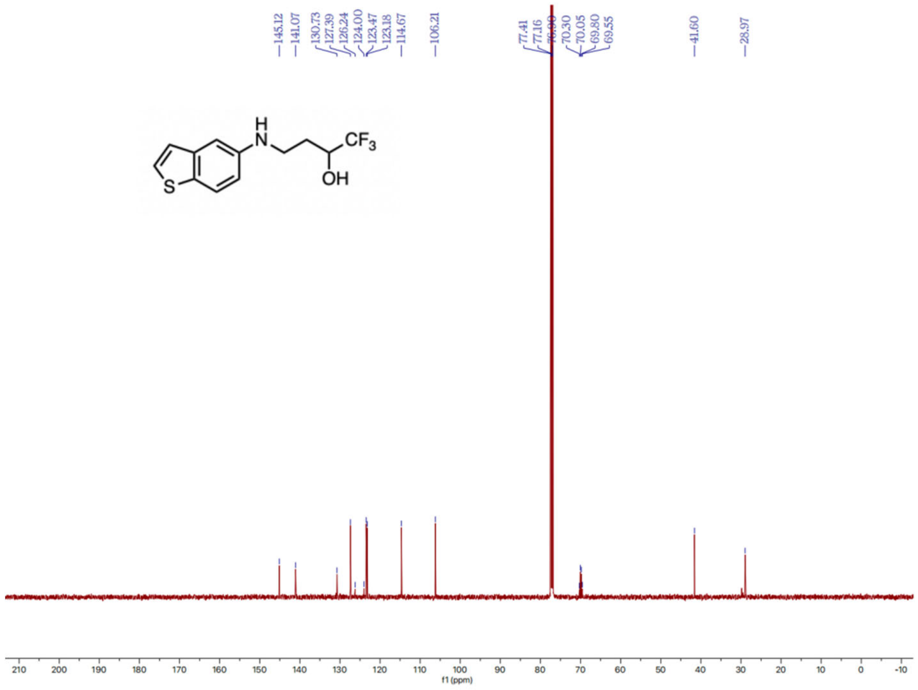

^{13}C NMR spectrum (126 MHz, Chloroform-*d*) of compound **45**

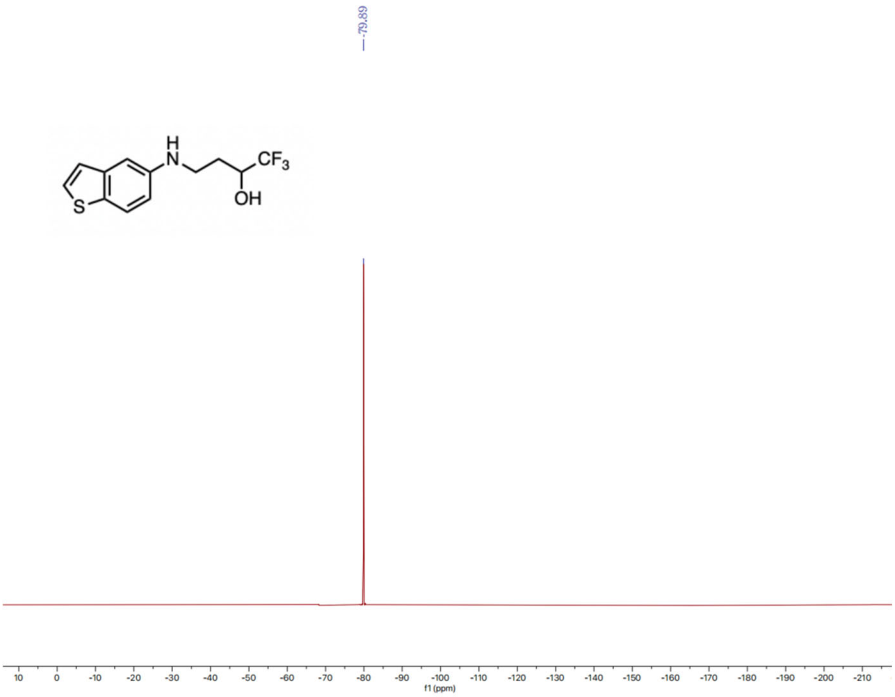

^{19}F NMR spectrum (471 MHz, Chloroform-*d*) of compound **45**

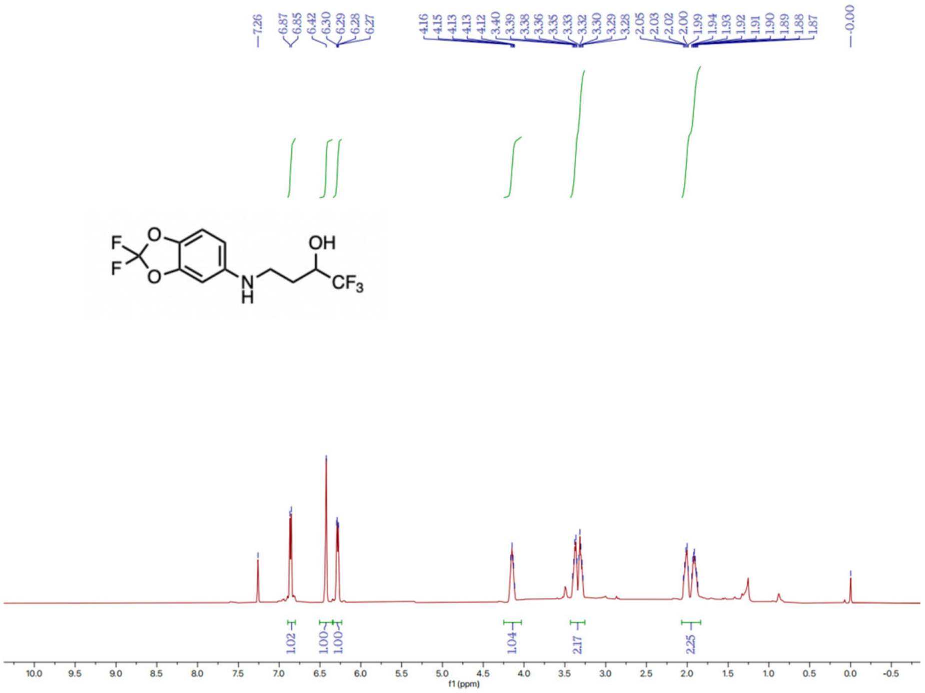

^{1}H NMR spectrum (500 MHz, Chloroform-*d*) of compound **46**

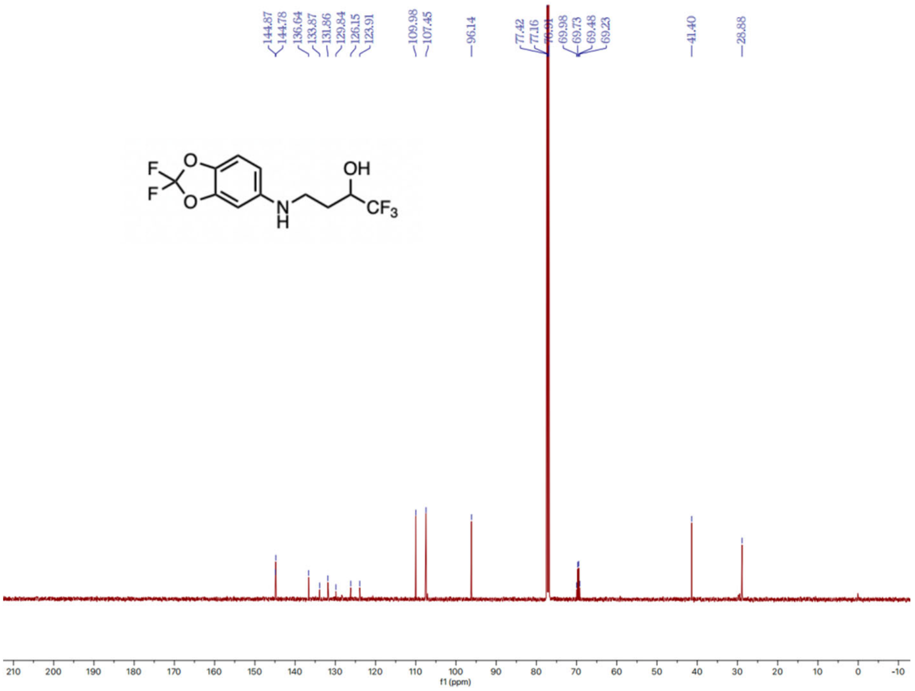

^{13}C NMR spectrum (126 MHz, Chloroform-*d*) of compound **46**

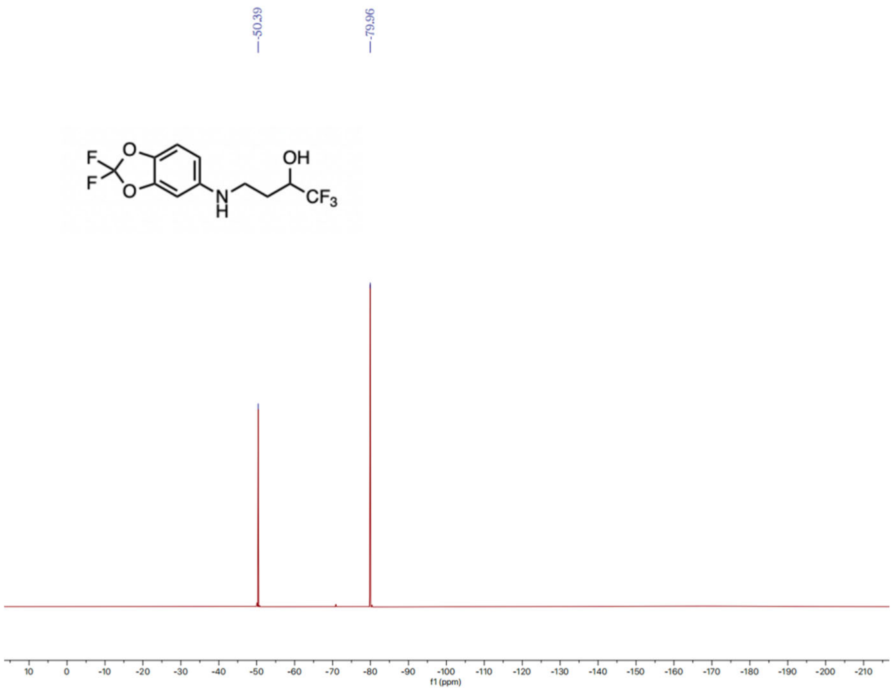

^{19}F NMR spectrum (471 MHz, Chloroform-*d*) of compound **46**

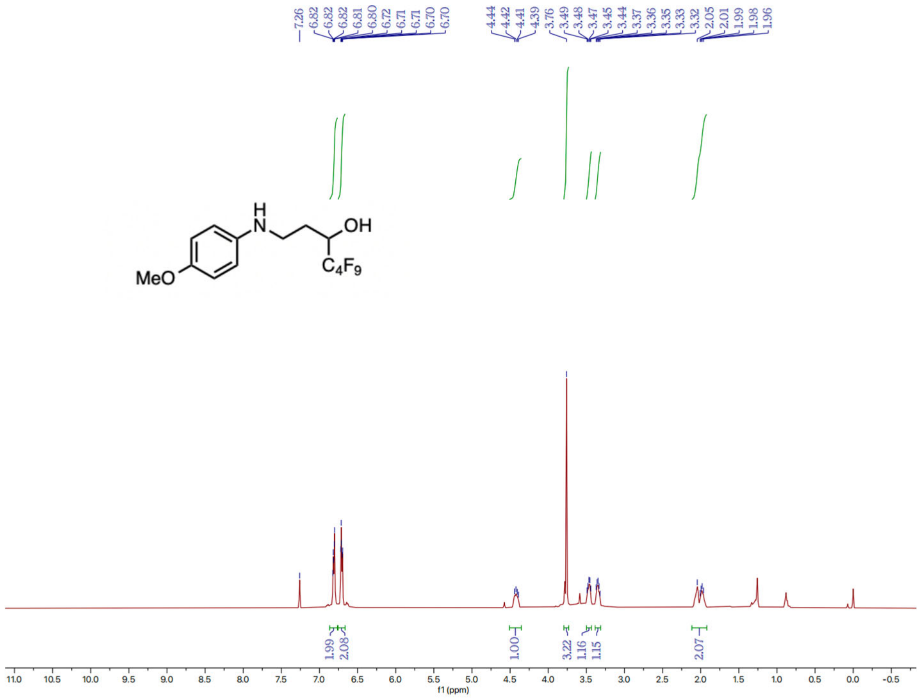

^{1}H NMR spectrum (500 MHz, Chloroform-*d*) of compound **47**

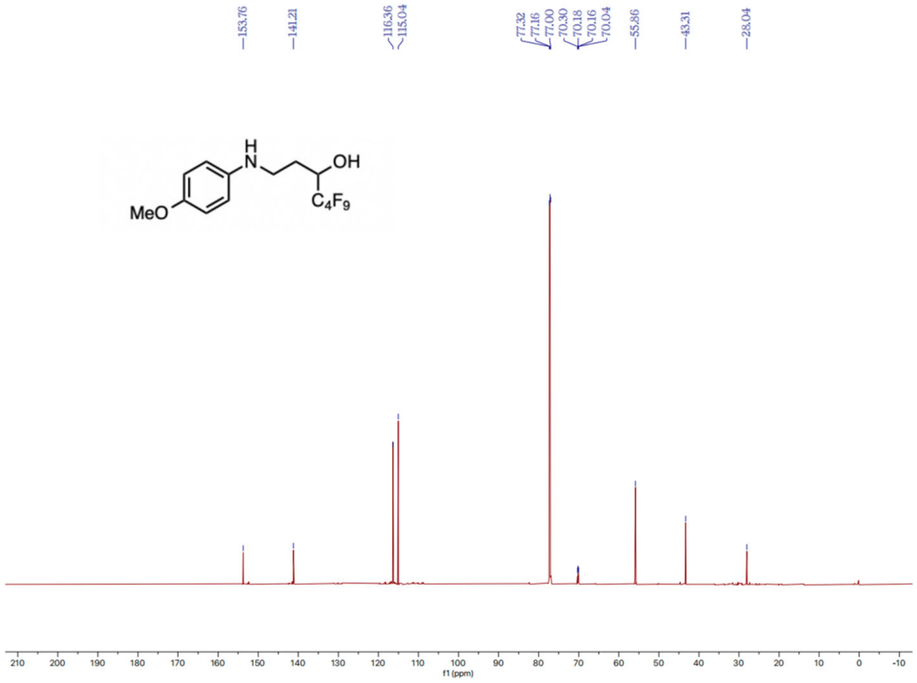

^{13}C NMR spectrum (201 MHz, Chloroform-*d*) of compound **47**

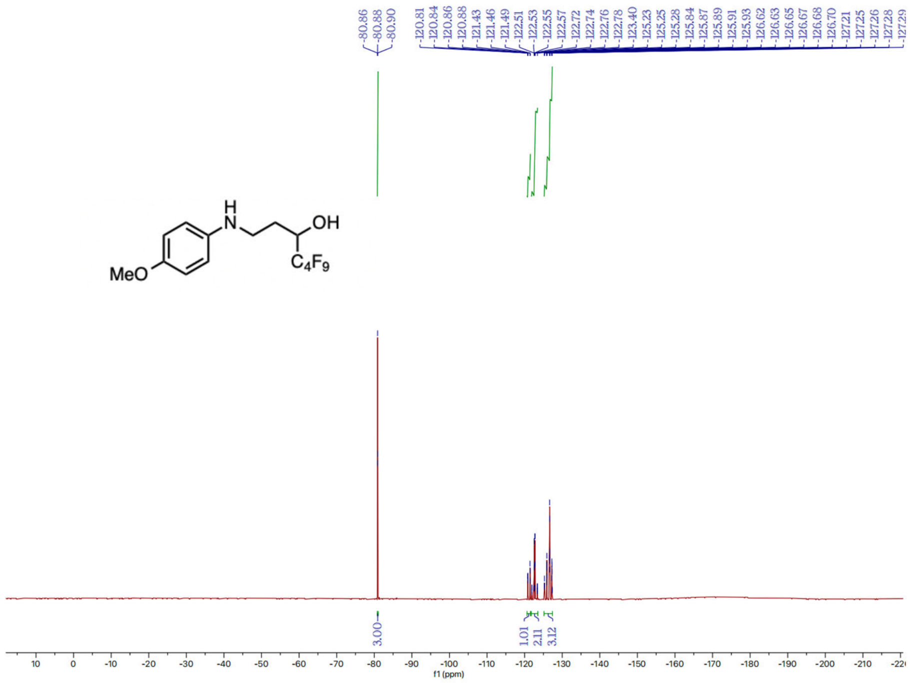

^{19}F NMR spectrum (471 MHz, Chloroform-*d*) of compound **47**

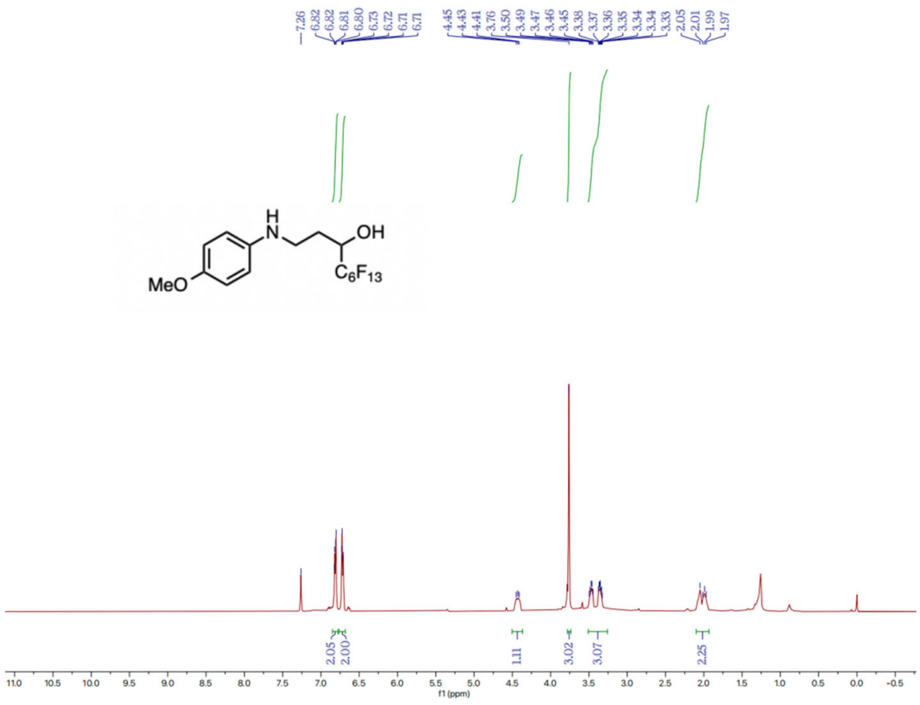

^{1}H NMR spectrum (500 MHz, Chloroform-*d*) of compound **48**

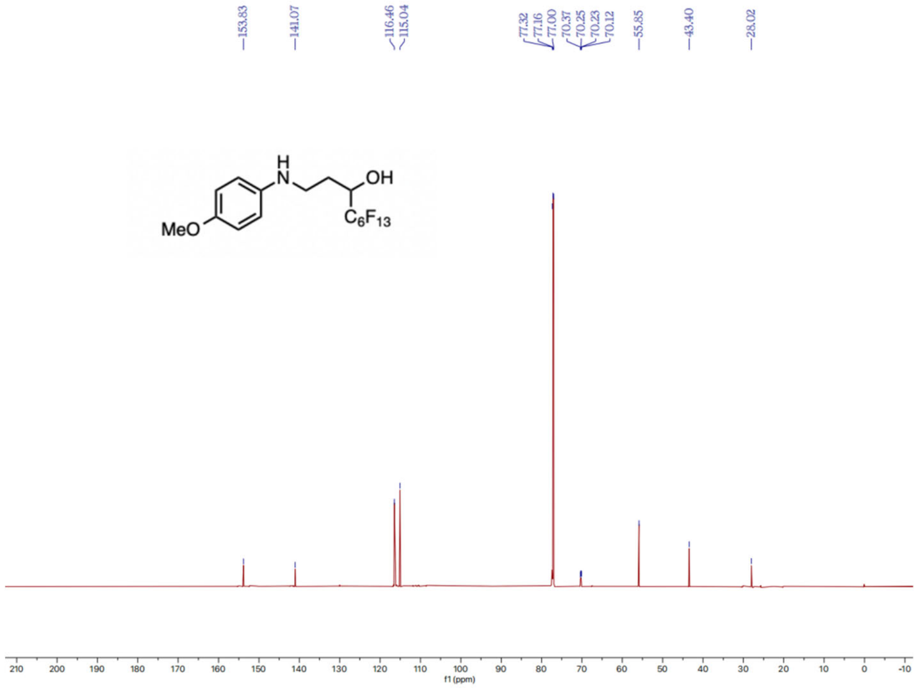

^{13}C NMR spectrum (201 MHz, Chloroform-*d*) of compound **48**

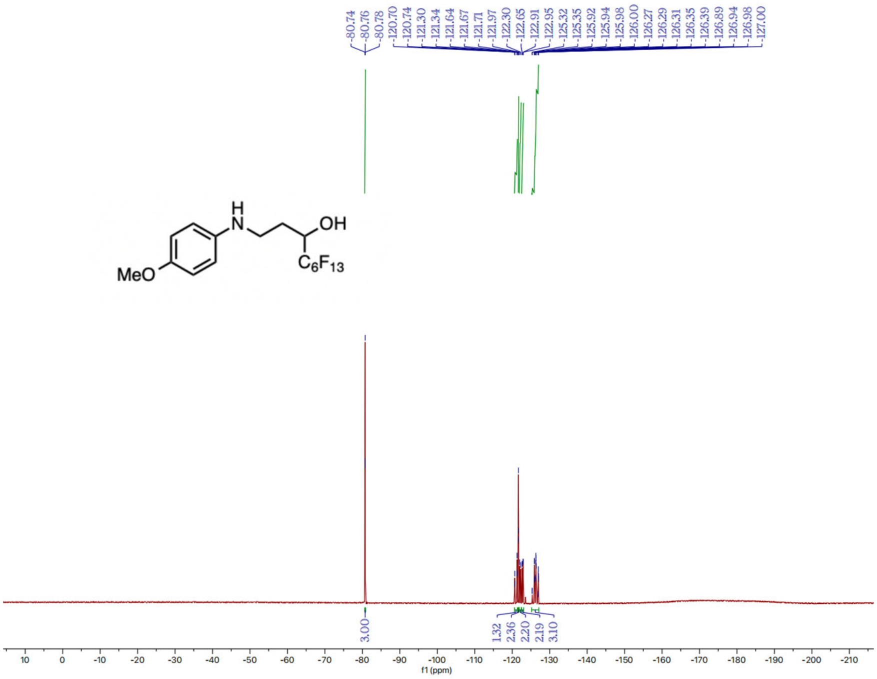

^{19}F NMR spectrum (471 MHz, Chloroform-*d*) of compound **48**

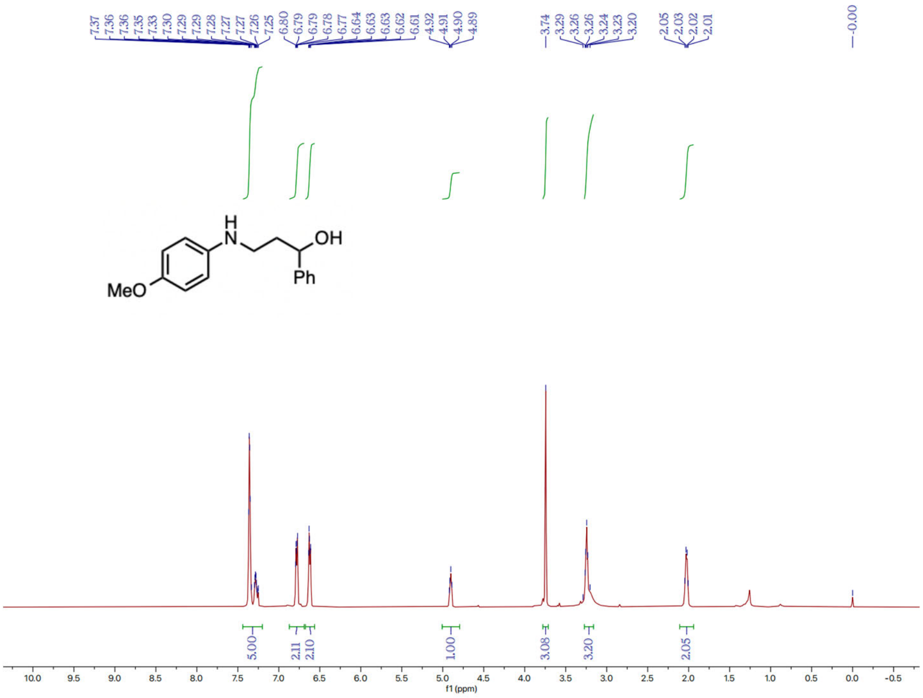

^{1}H NMR spectrum (500 MHz, Chloroform-*d*) of compound **49**

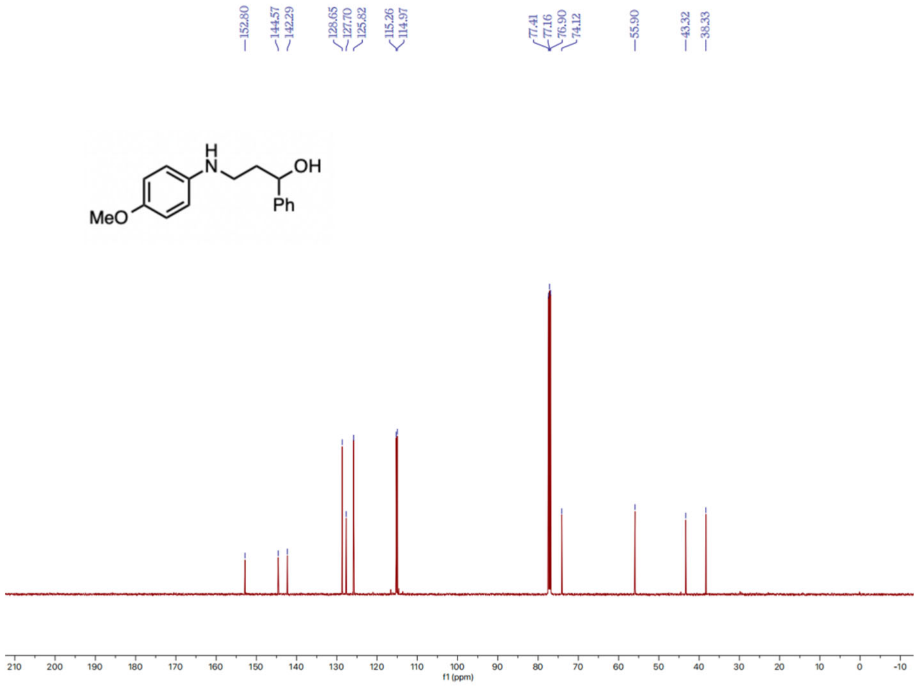

^{13}C NMR spectrum (126 MHz, Chloroform-*d*) of compound **49**

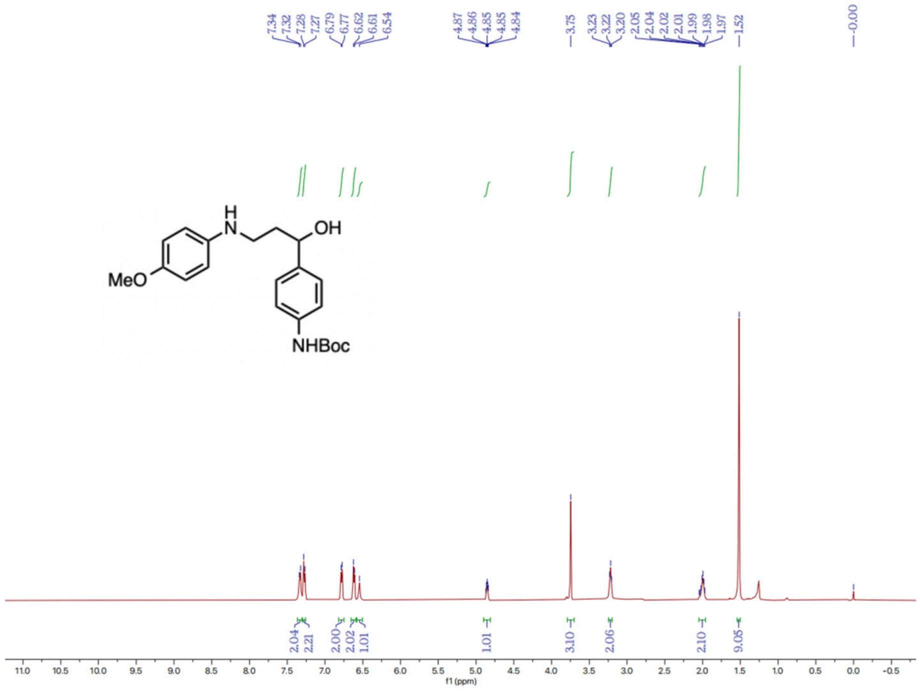

^{1}H NMR spectrum (500 MHz, Chloroform-*d*) of compound **50**

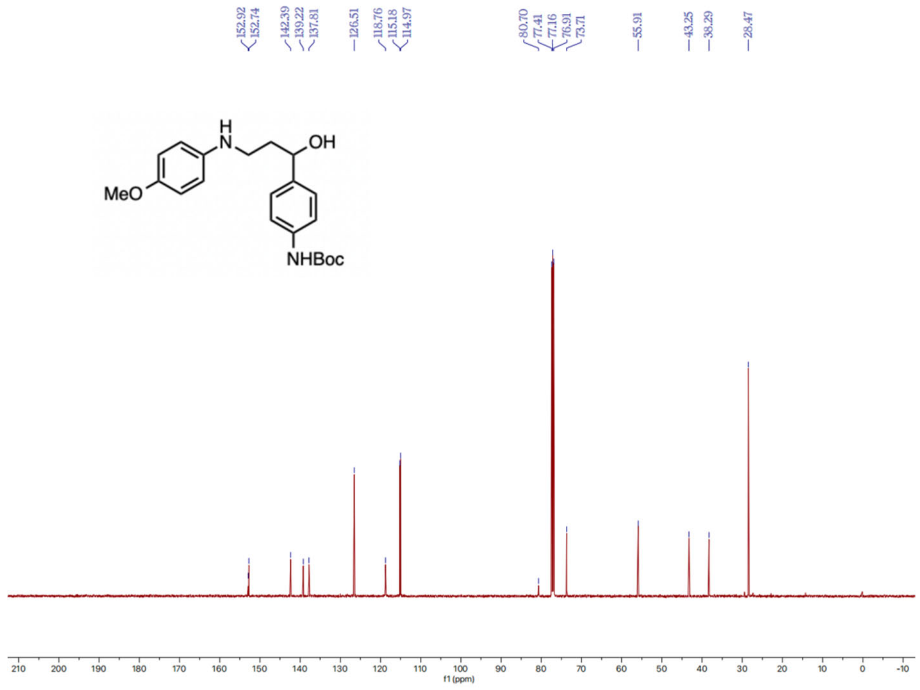

^{13}C NMR spectrum (126 MHz, Chloroform-*d*) of compound **50**

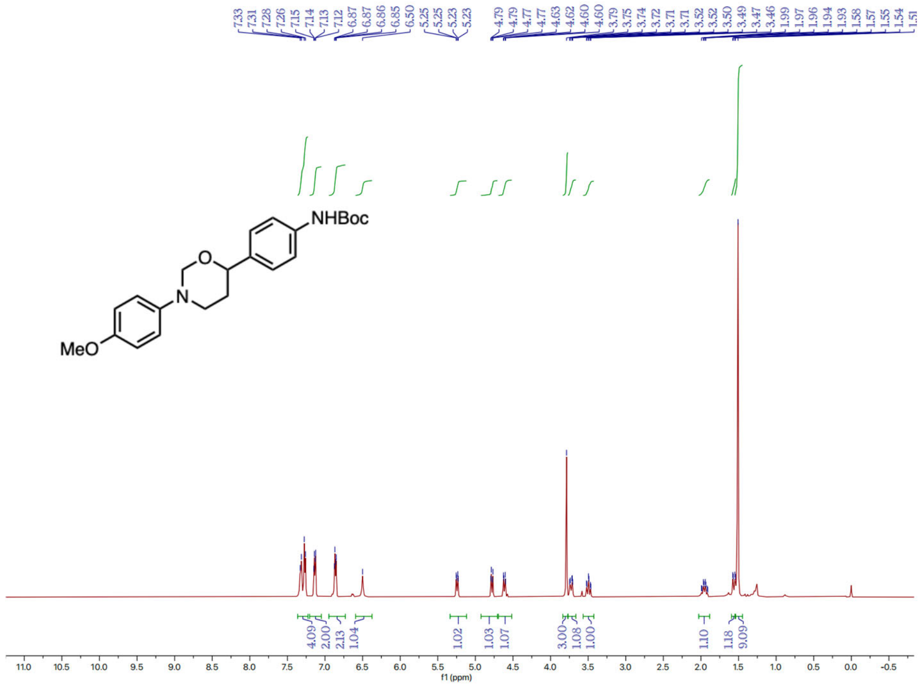

^{1}H NMR spectrum (500 MHz, Chloroform-*d*) of compound **50’**

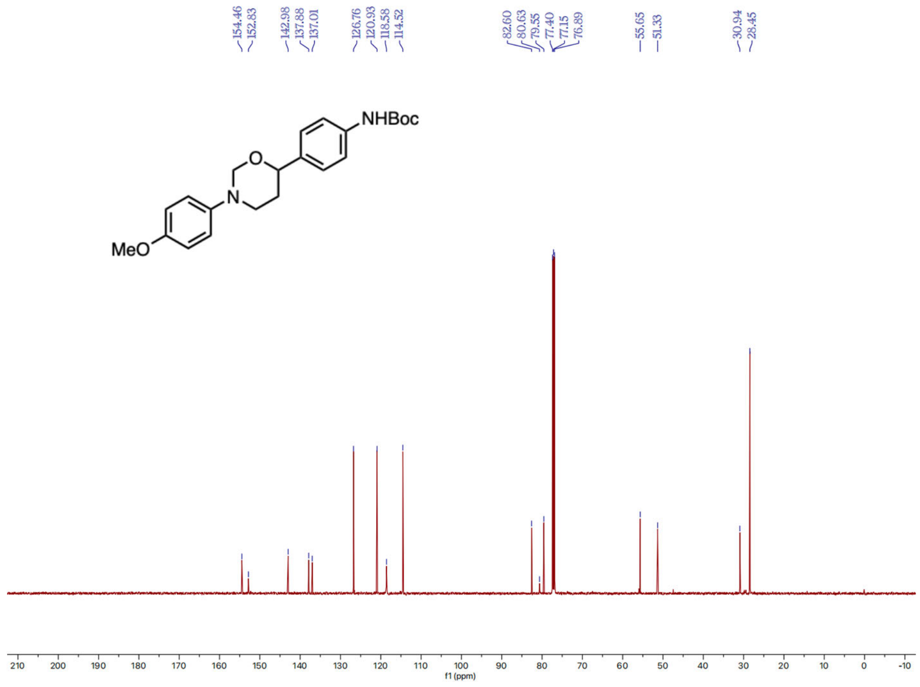

^{13}C NMR spectrum (126 MHz, Chloroform-*d*) of compound **50’**

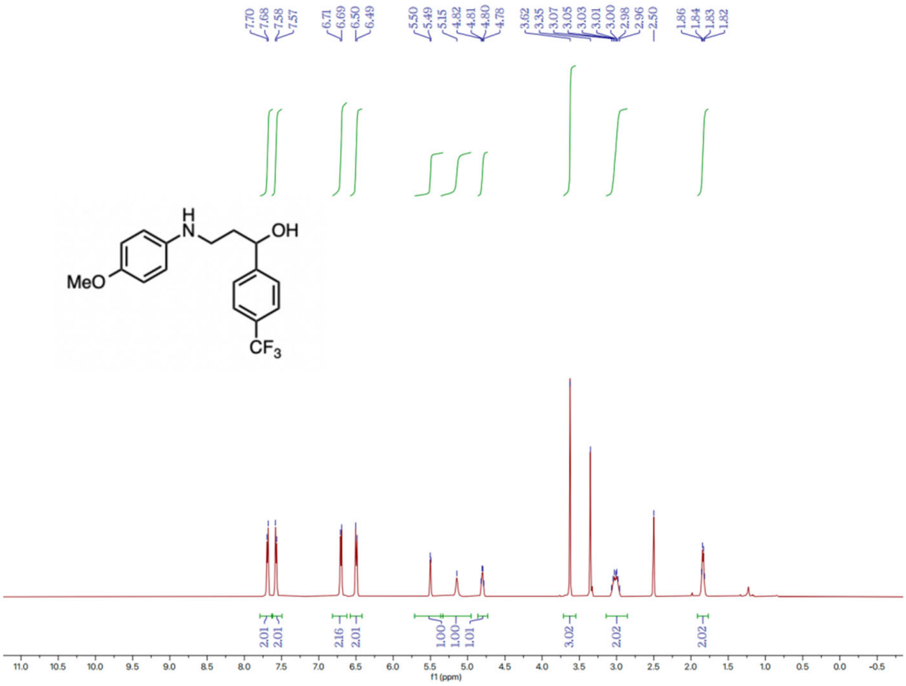

^{1}H NMR spectrum (500 MHz, DMSO-d_6) of compound **51**

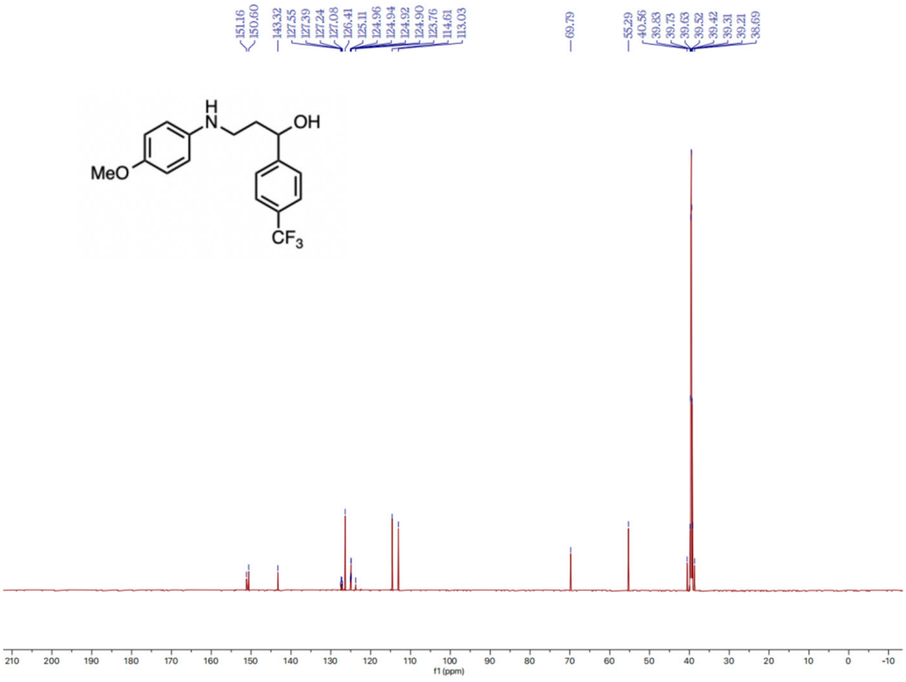

^{13}C NMR spectrum (201 MHz, DMSO-d_6) of compound **51**

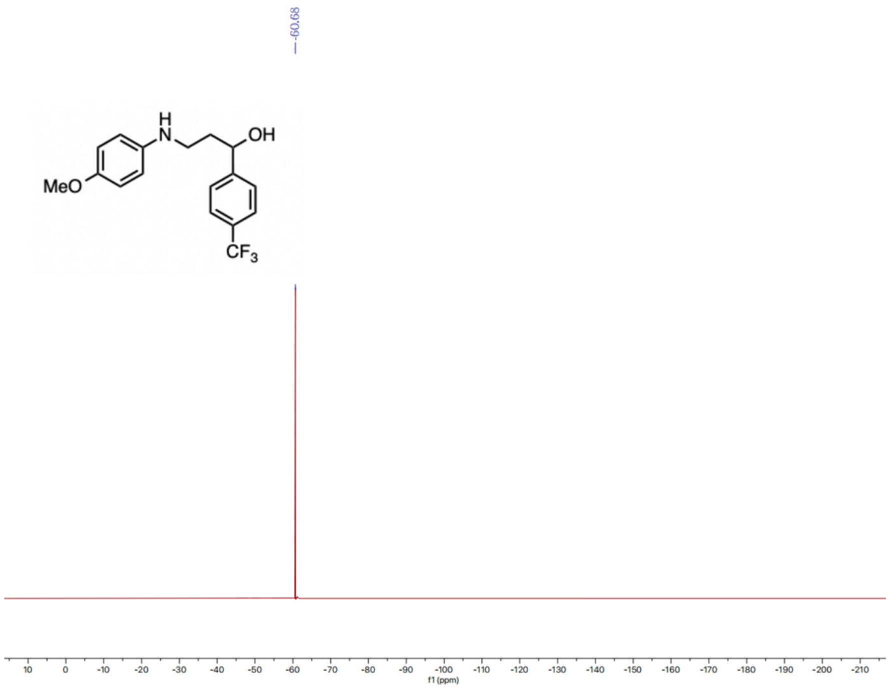

^{19}F NMR spectrum (471 MHz, DMSO-d_6) of compound **51**

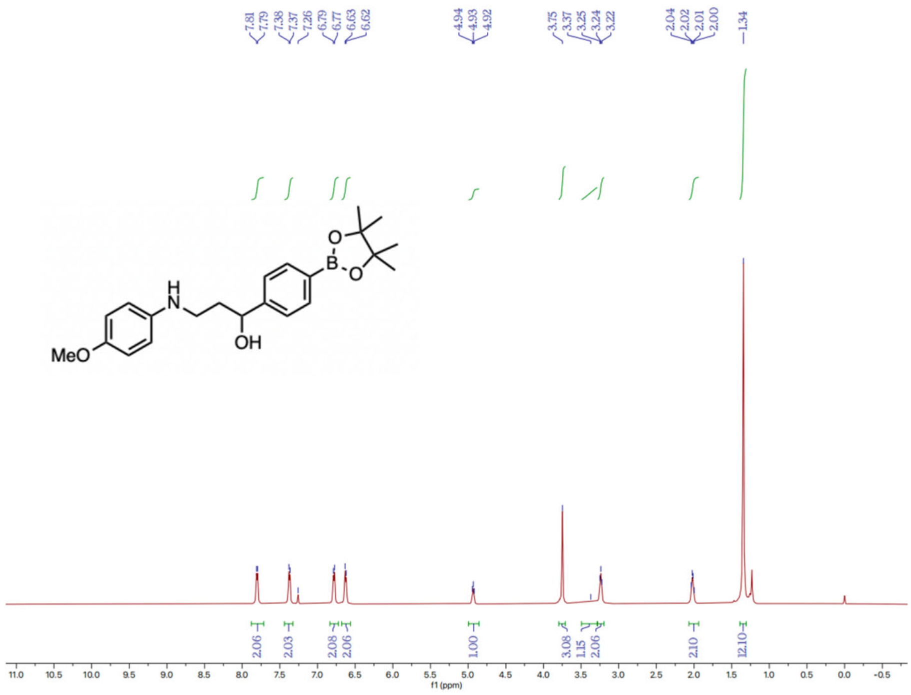

^{1}H NMR spectrum (500 MHz, Chloroform-*d*) of compound **52**

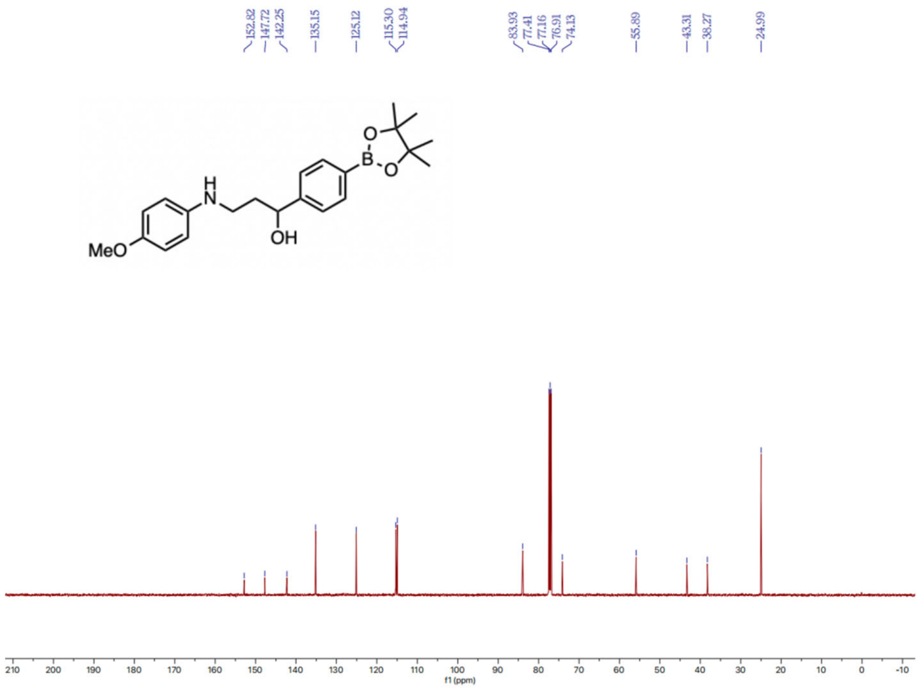

^{13}C NMR spectrum (126 MHz, Chloroform-*d*) of compound **52**

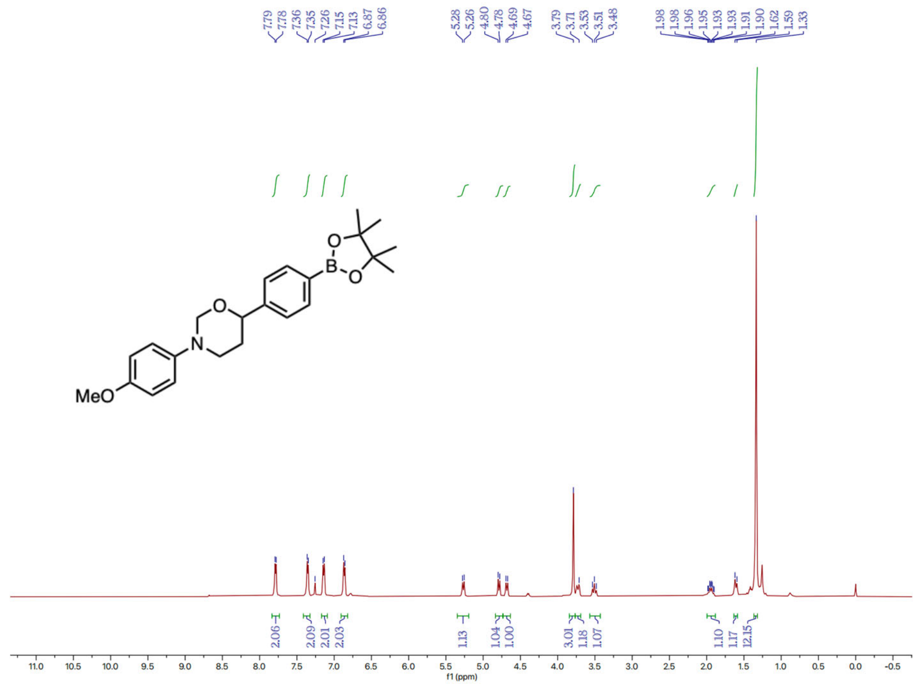

^{1}H NMR spectrum (500 MHz, Chloroform-*d*) of compound **52’**

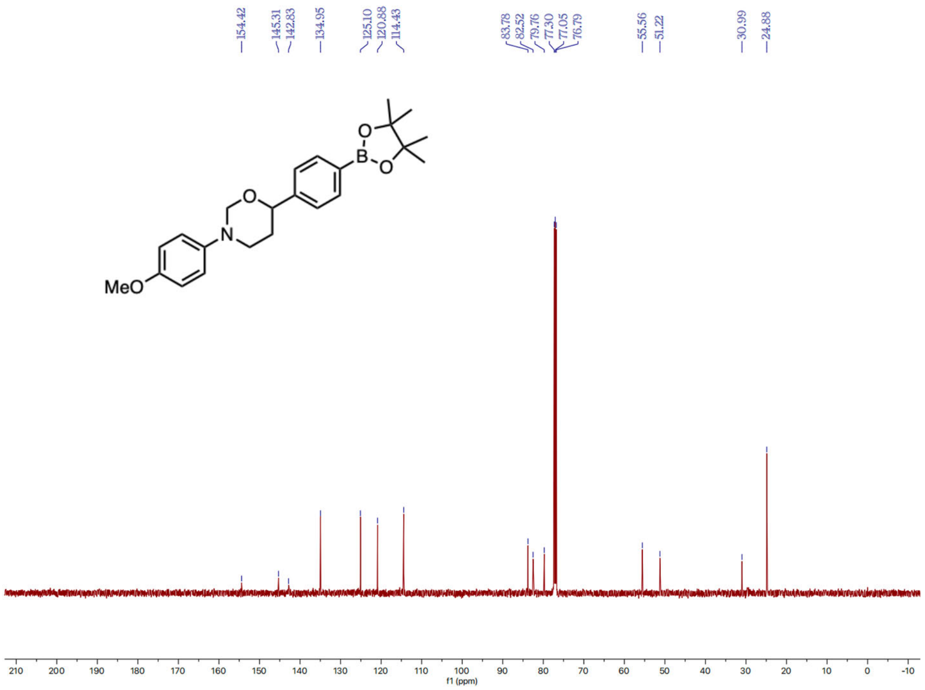

^{13}C NMR spectrum (126 MHz, Chloroform-*d*) of compound **52’**

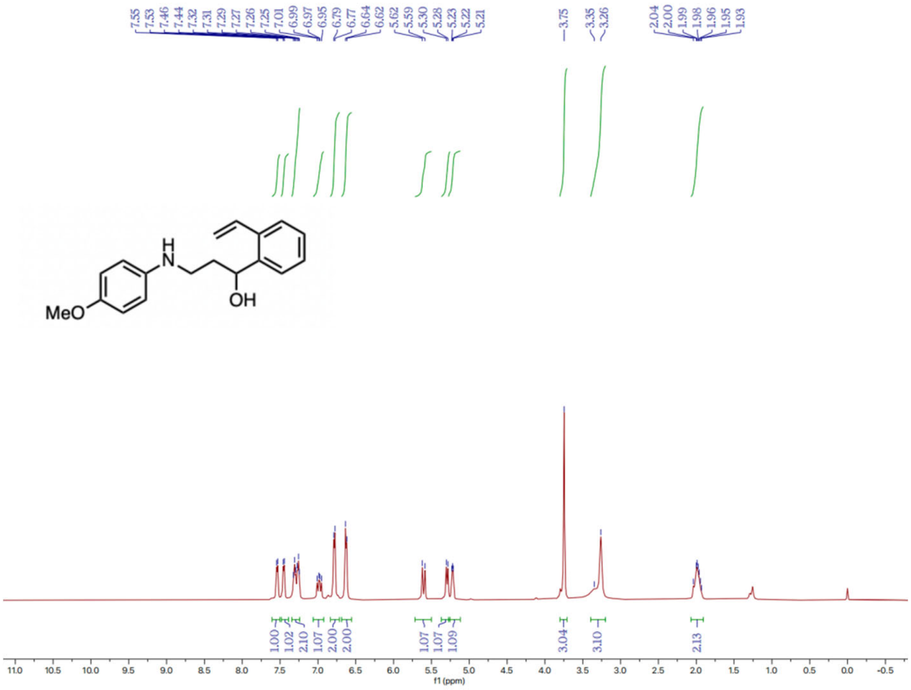

^{1}H NMR spectrum (500 MHz, Chloroform-*d*) of compound **53**

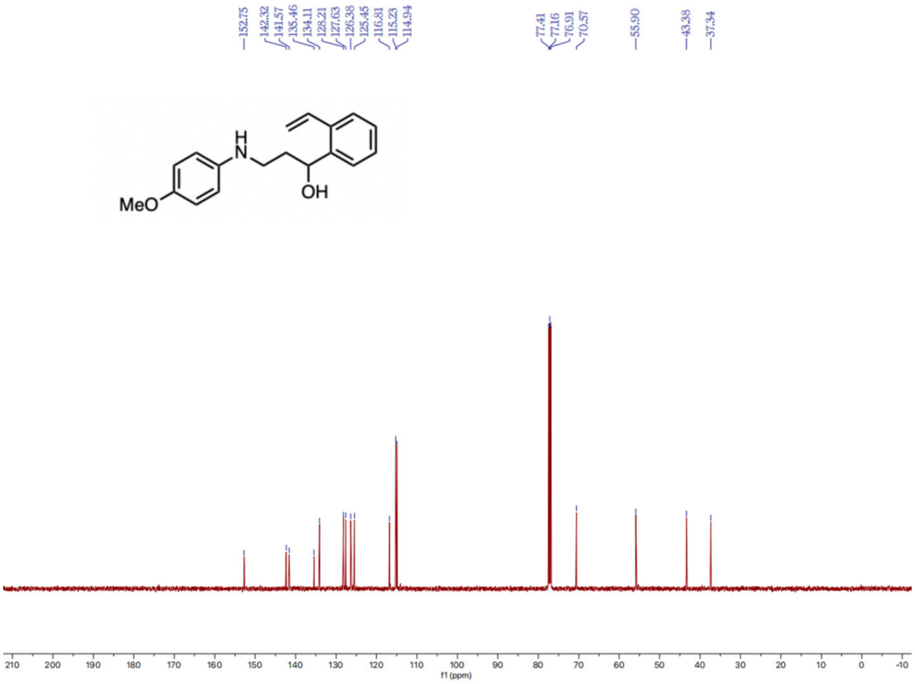

^{13}C NMR spectrum (126 MHz, Chloroform-*d*) of compound **53**

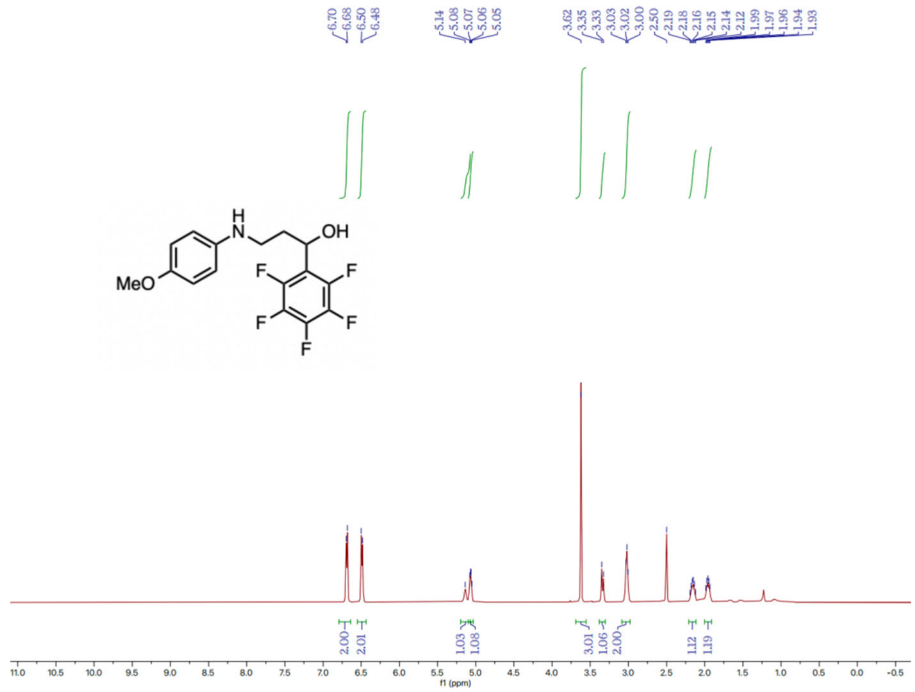

^{1}H NMR spectrum (500 MHz, DMSO-d_6) of compound **54**

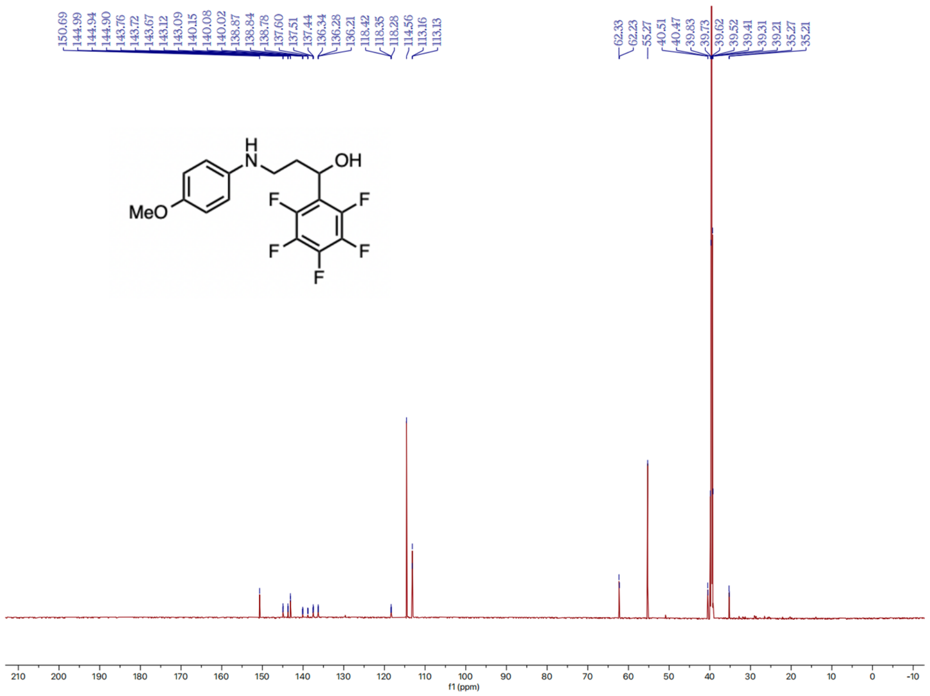

^{13}C NMR spectrum (201 MHz, DMSO-d_6) of compound **54**

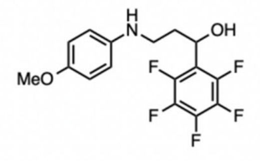

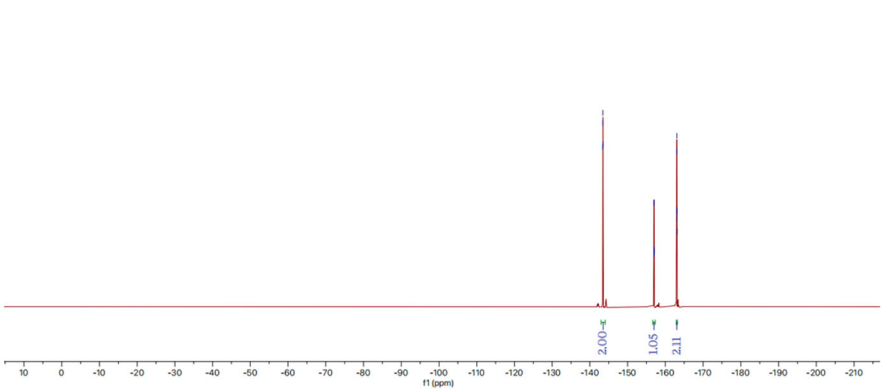

^{19}F NMR spectrum (471 MHz, DMSO-d_6) of compound **54**

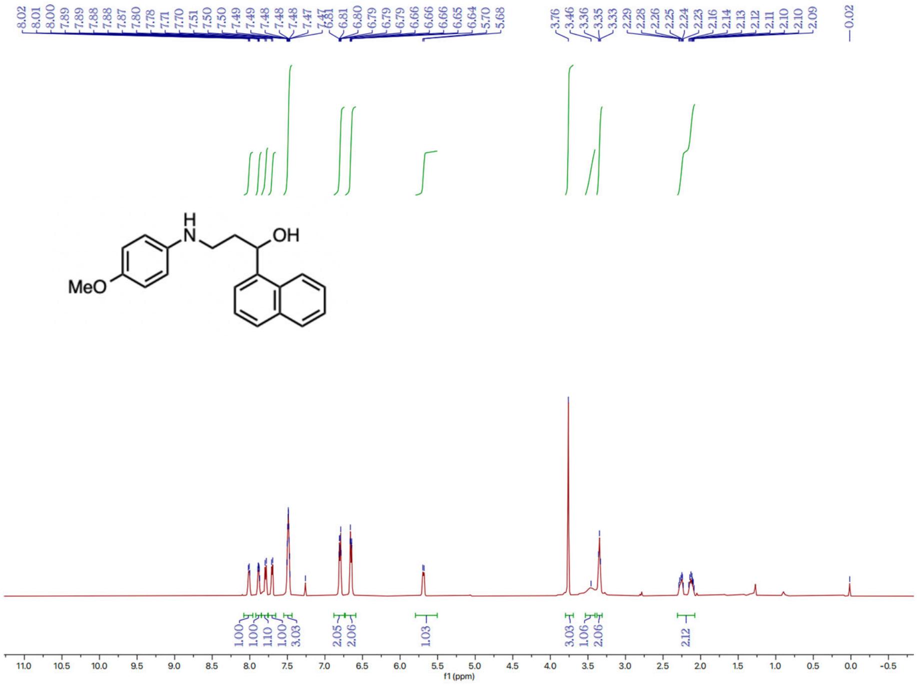

^{1}H NMR spectrum (500 MHz, Chloroform-*d*) of compound **55**

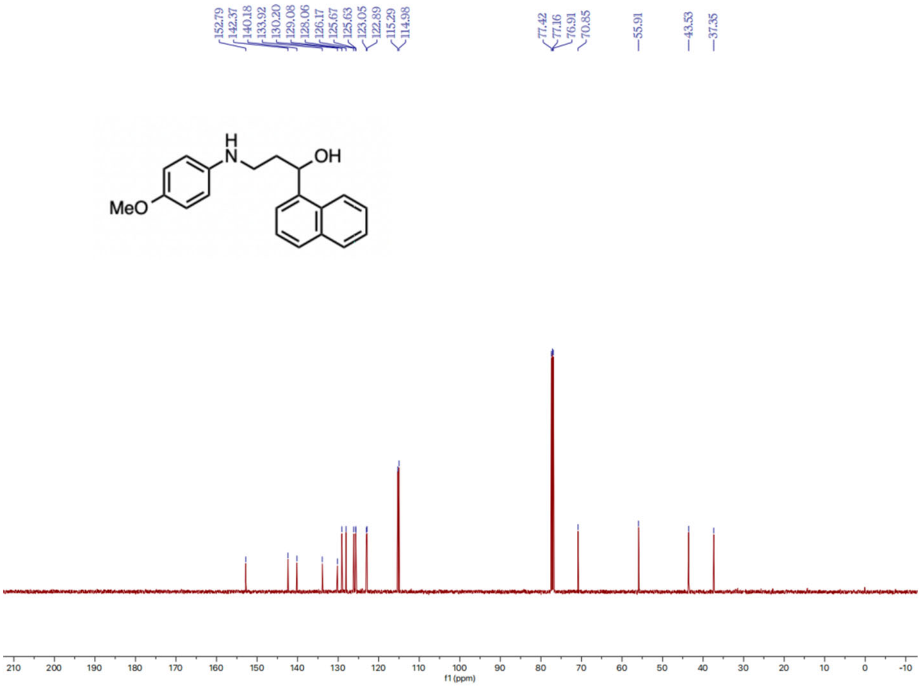

^{13}C NMR spectrum (126 MHz, Chloroform-*d*) of compound **55**

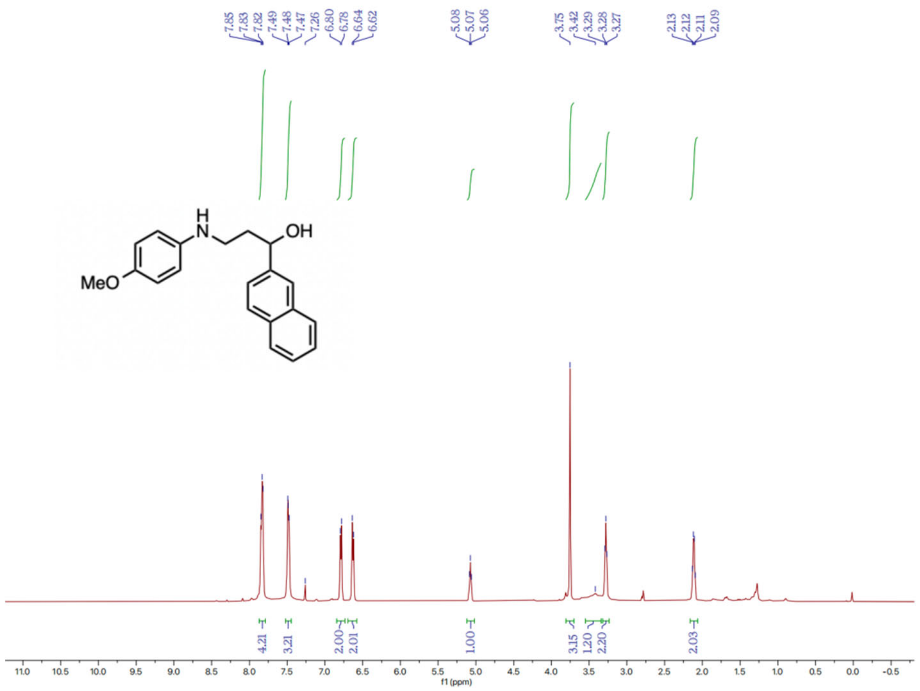

^{1}H NMR spectrum (500 MHz, Chloroform-*d*) of compound **56**

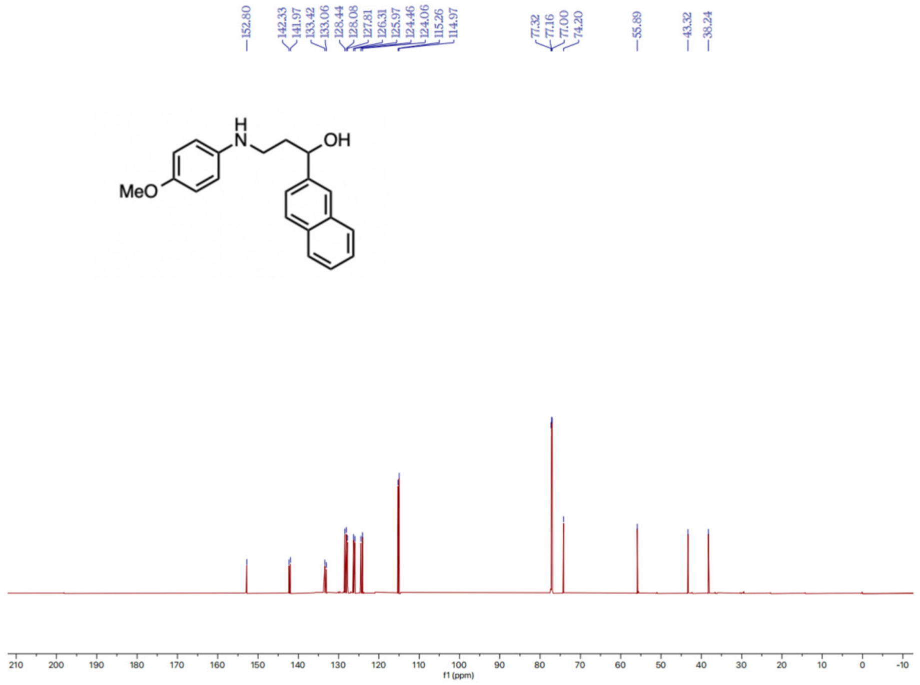

^{13}C NMR spectrum (201 MHz, Chloroform-*d*) of compound **56**

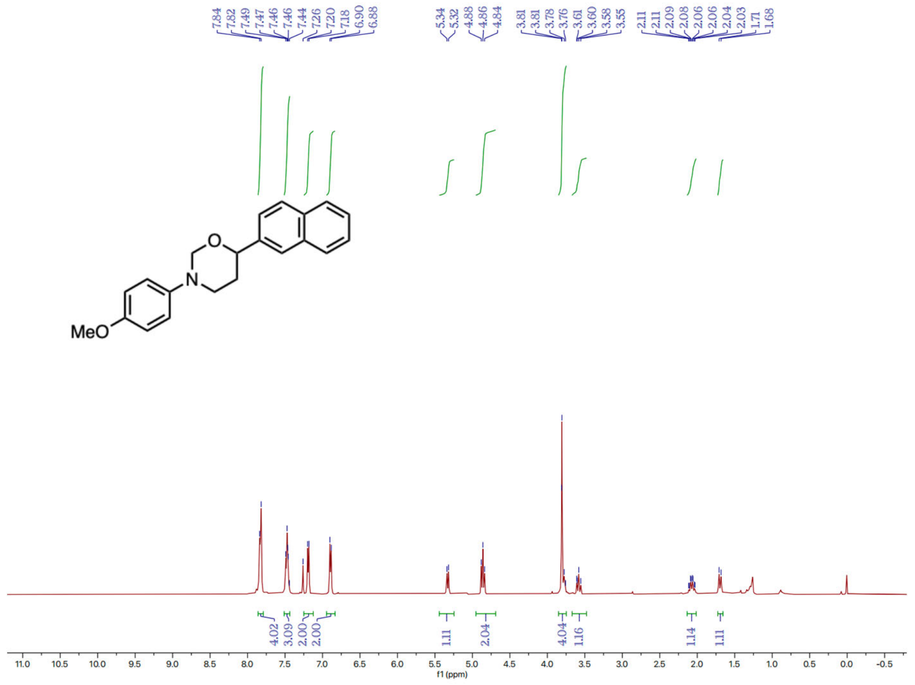

^{1}H NMR spectrum (500 MHz, Chloroform-*d*) of compound **56’**

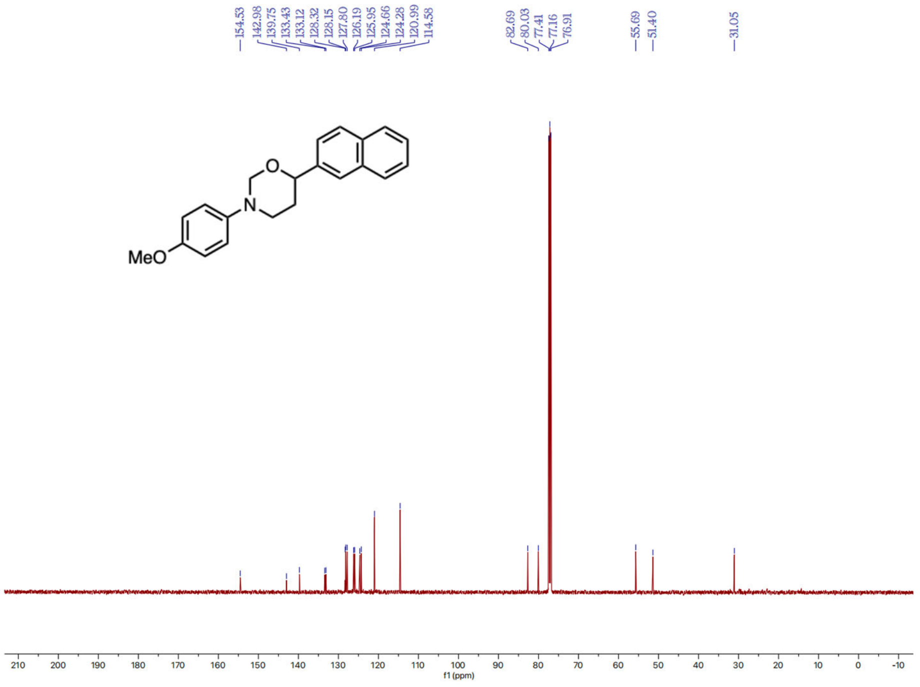

^{13}C NMR spectrum (126 MHz, Chloroform-*d*) of compound **56’**

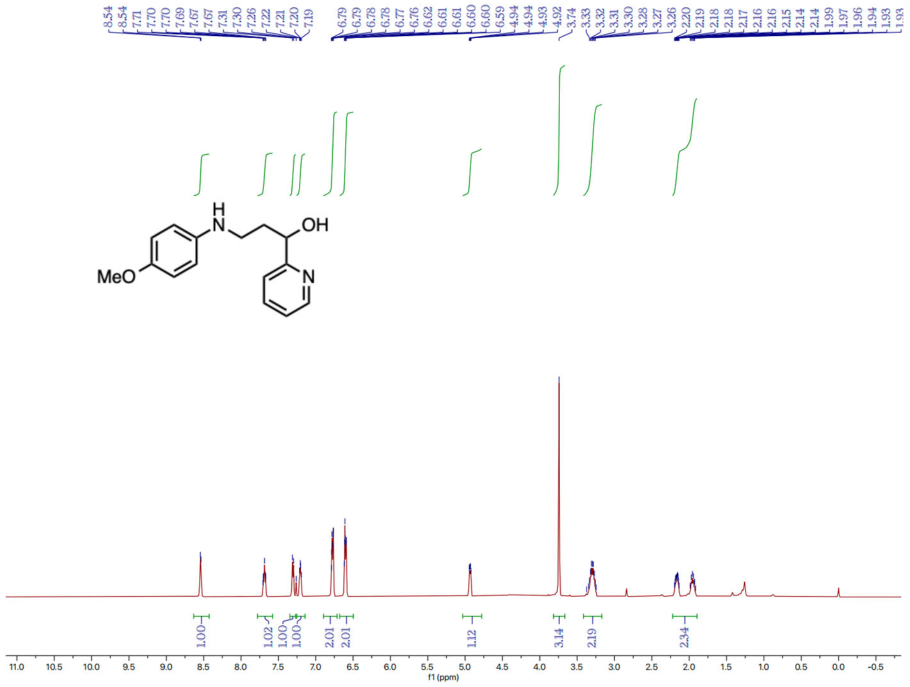

^{1}H NMR spectrum (500 MHz, Chloroform-*d*) of compound **57**

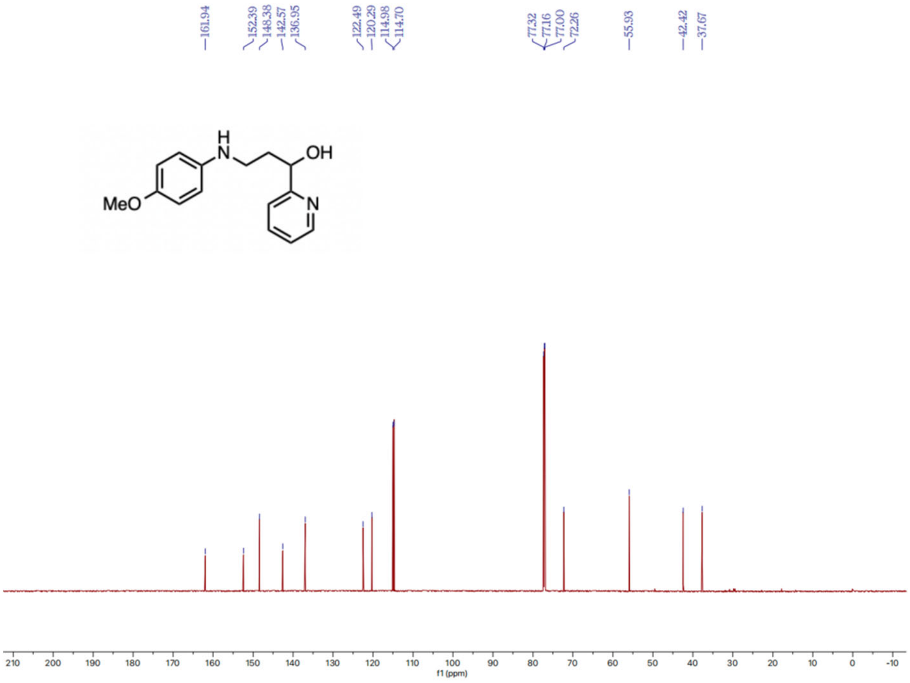

^{13}C NMR spectrum (201 MHz, Chloroform-*d*) of compound **57**

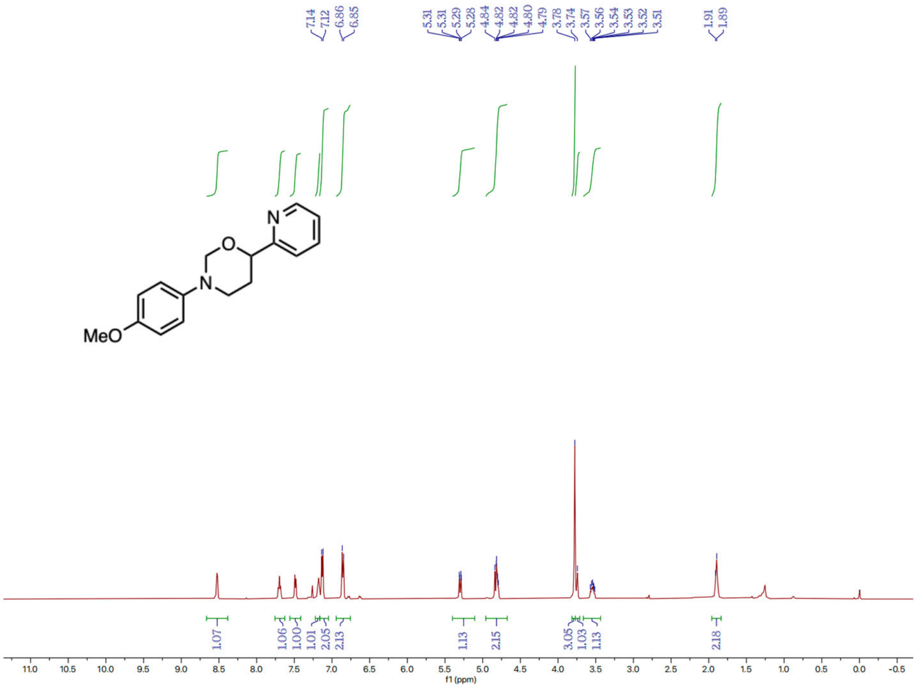

^{1}H NMR spectrum (500 MHz, Chloroform-*d*) of compound **57’**

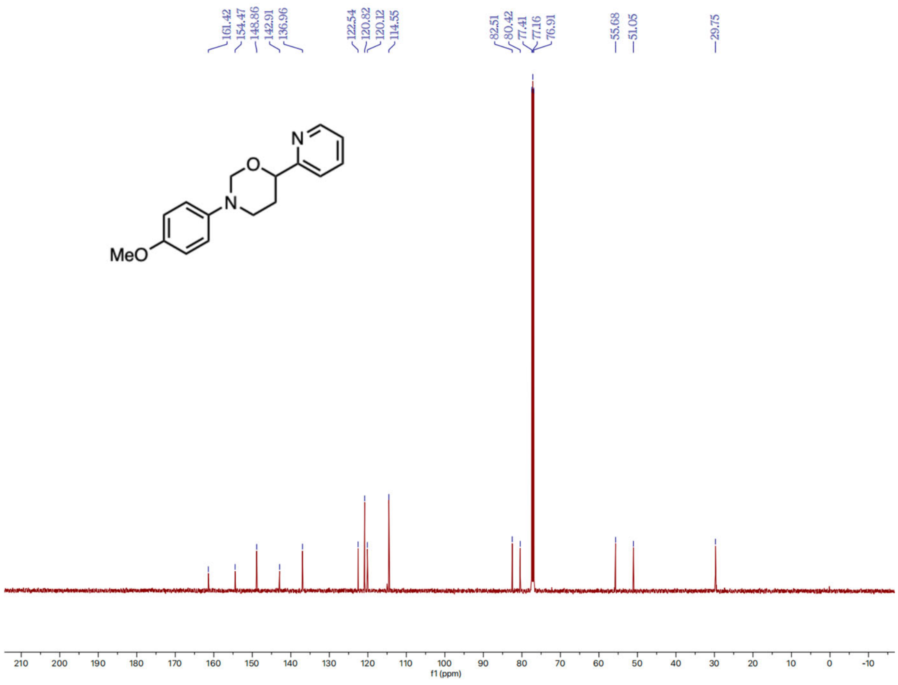

^{13}C NMR spectrum (126 MHz, Chloroform-*d*) of compound **57’**

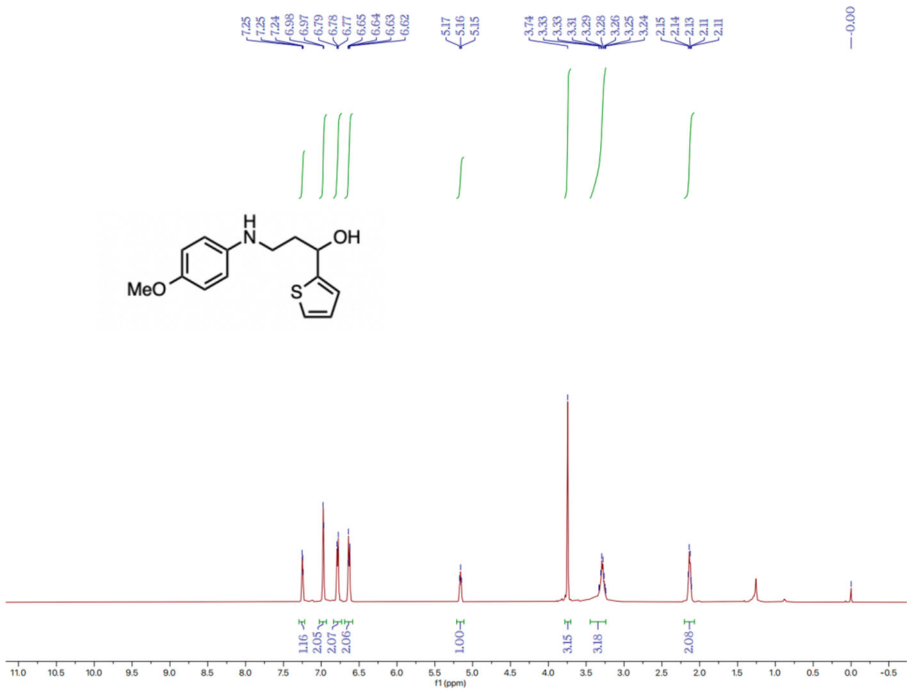

^{1}H NMR spectrum (500 MHz, Chloroform-*d*) of compound **58**

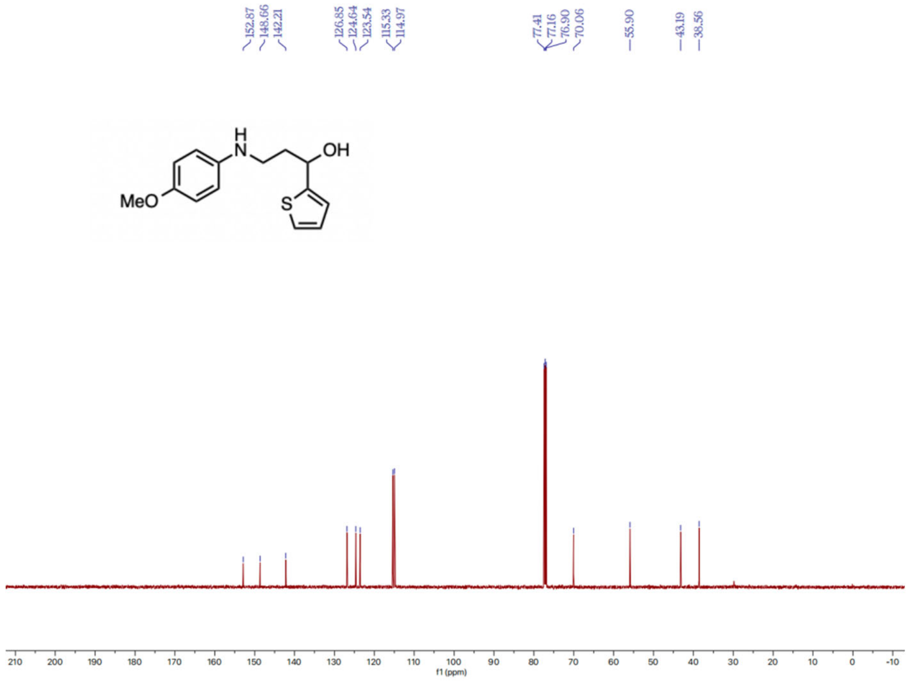

^{13}C NMR spectrum (126 MHz, Chloroform-*d*) of compound **58**

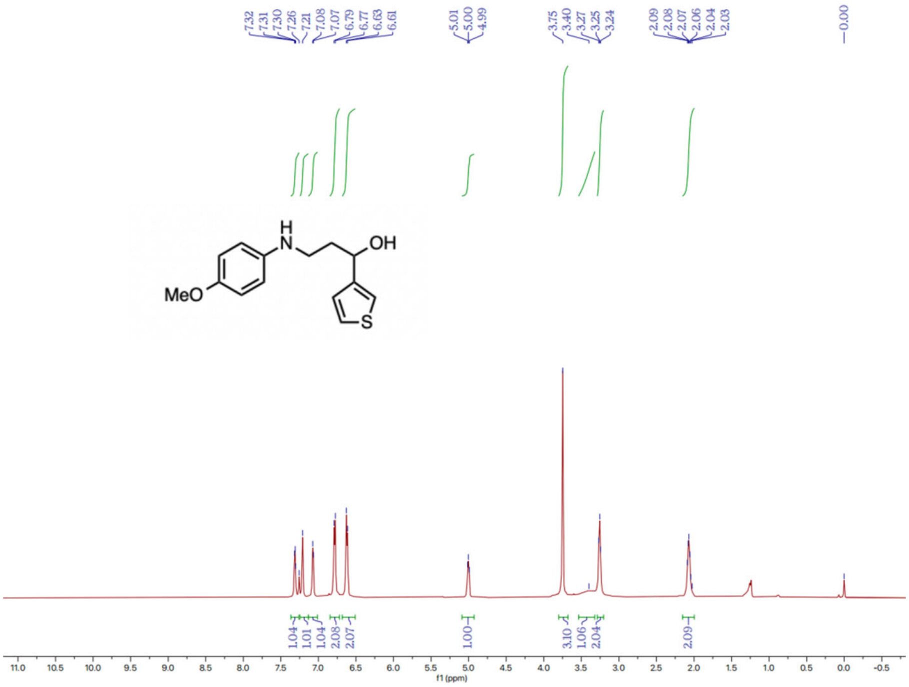

^{1}H NMR spectrum (500 MHz, Chloroform-*d*) of compound **59**

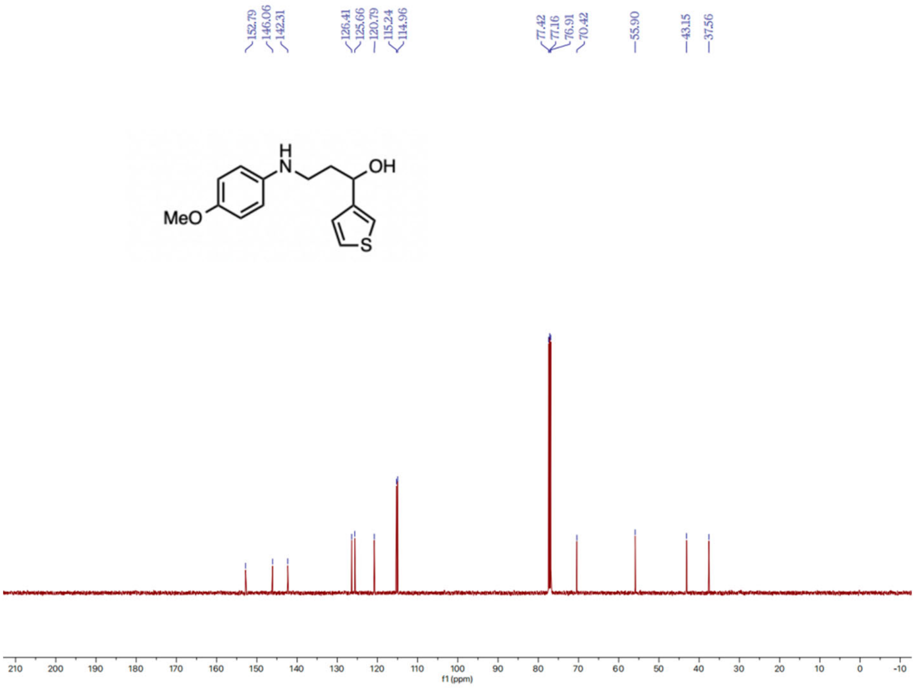

^{13}C NMR spectrum (126 MHz, Chloroform-*d*) of compound **59**

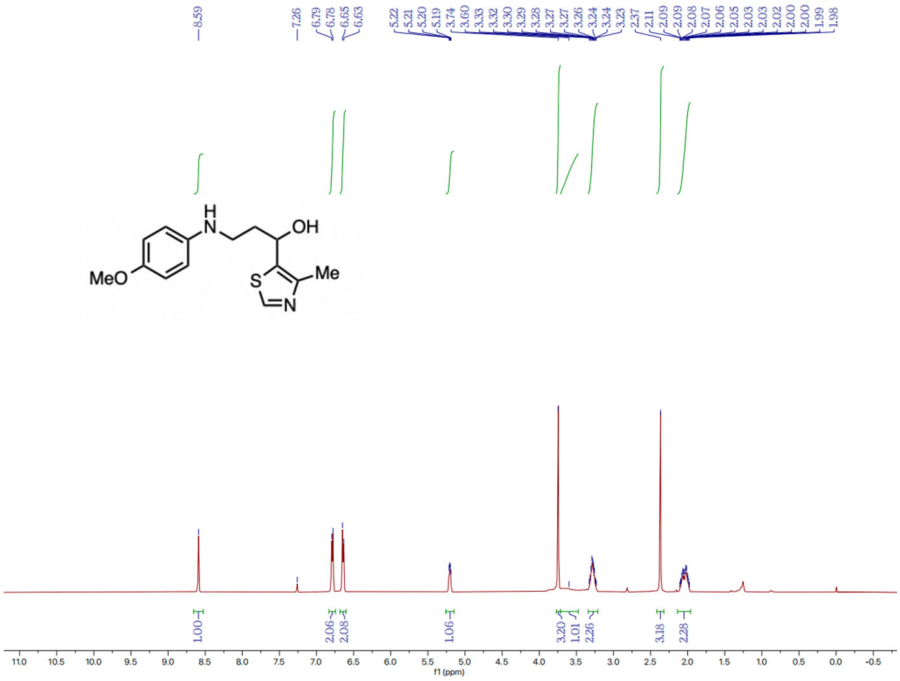

^{1}H NMR spectrum (500 MHz, Chloroform-*d*) of compound **60**

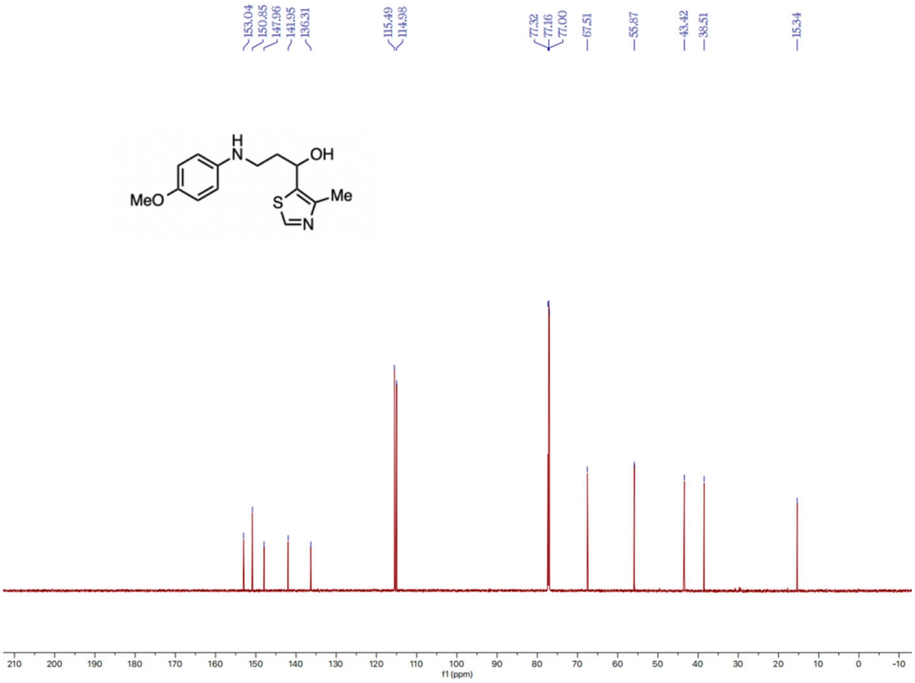

^{13}C NMR spectrum (201 MHz, Chloroform-*d*) of compound **60**

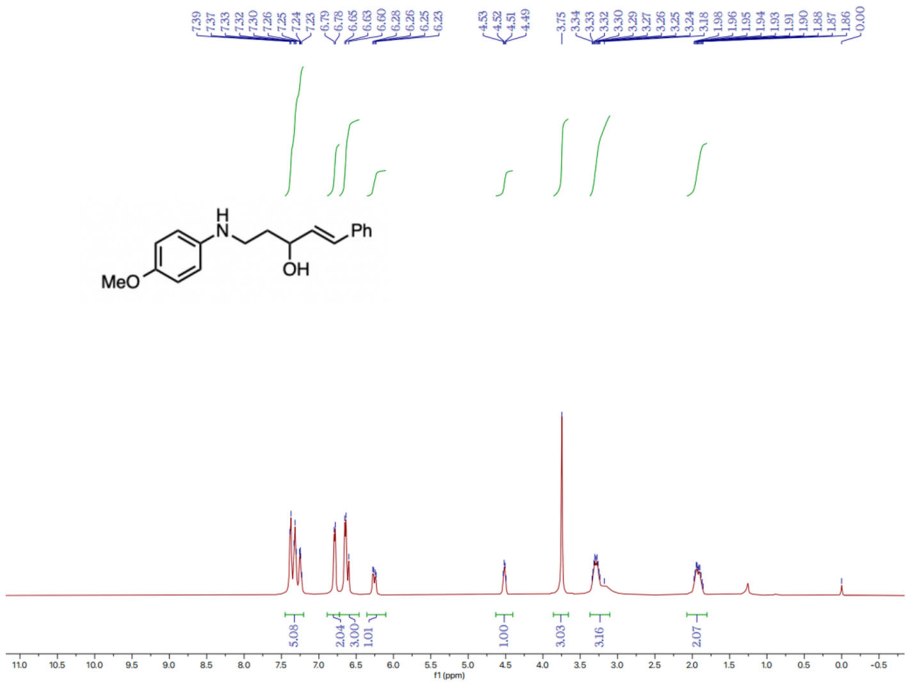

^{1}H NMR spectrum (500 MHz, Chloroform-*d*) of compound **61**

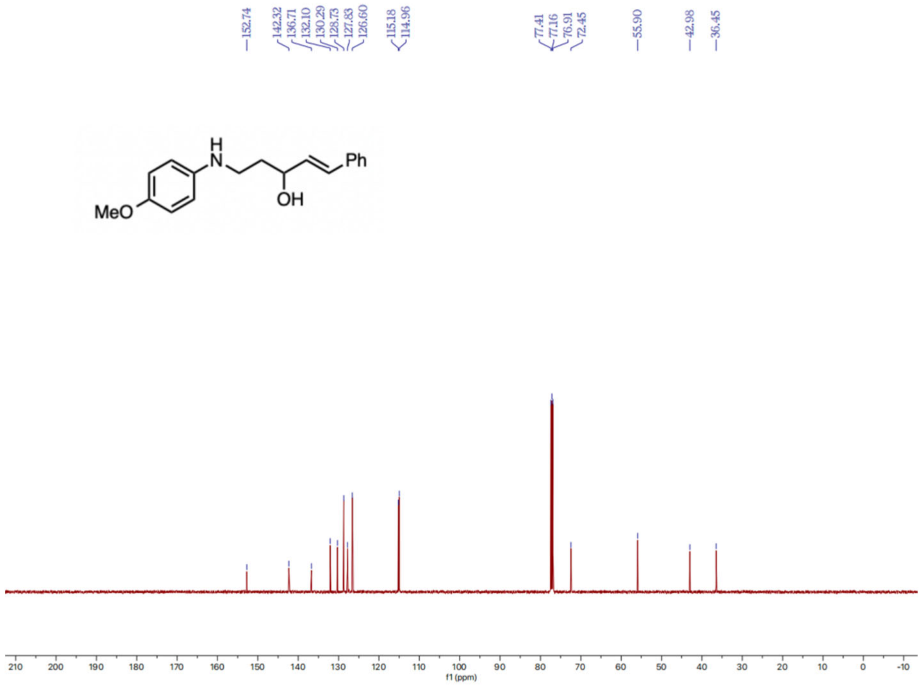

^{13}C NMR spectrum (126 MHz, Chloroform-*d*) of compound **61**

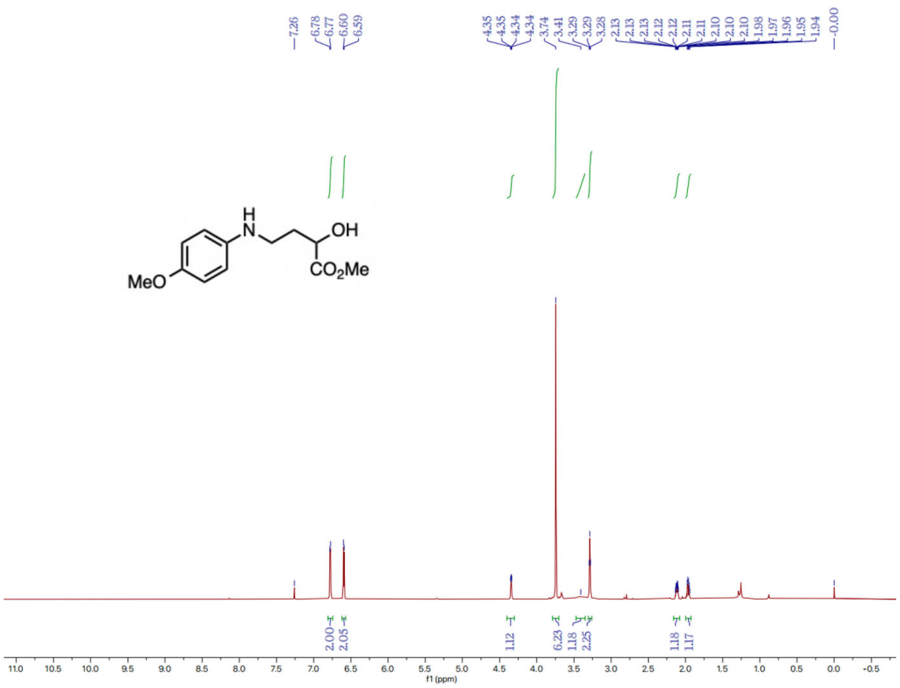

^{1}H NMR spectrum (800 MHz, Chloroform-*d*) of compound **62**

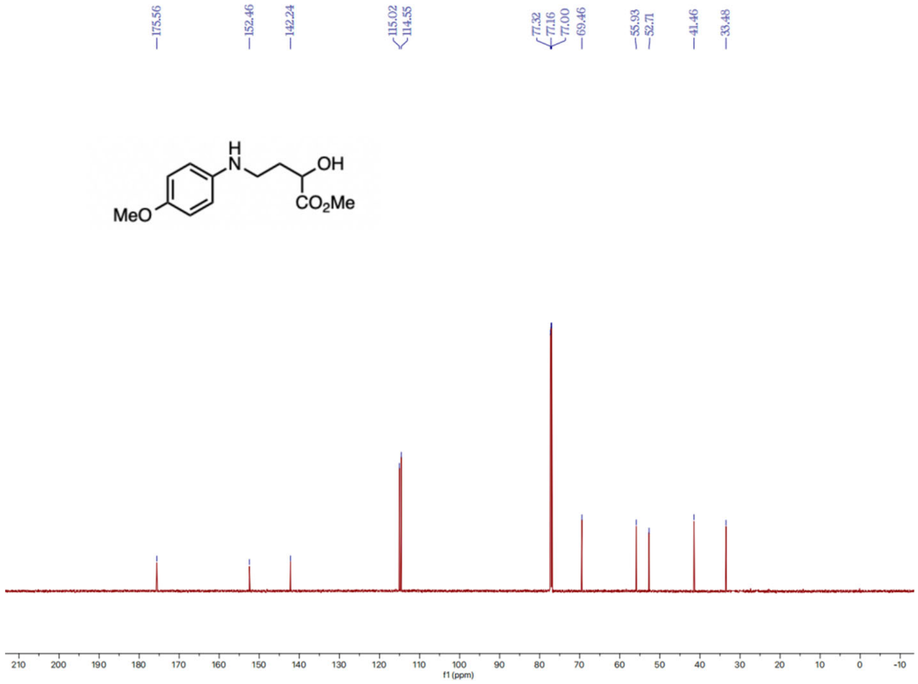

^{13}C NMR spectrum (201 MHz, Chloroform-*d*) of compound **62**

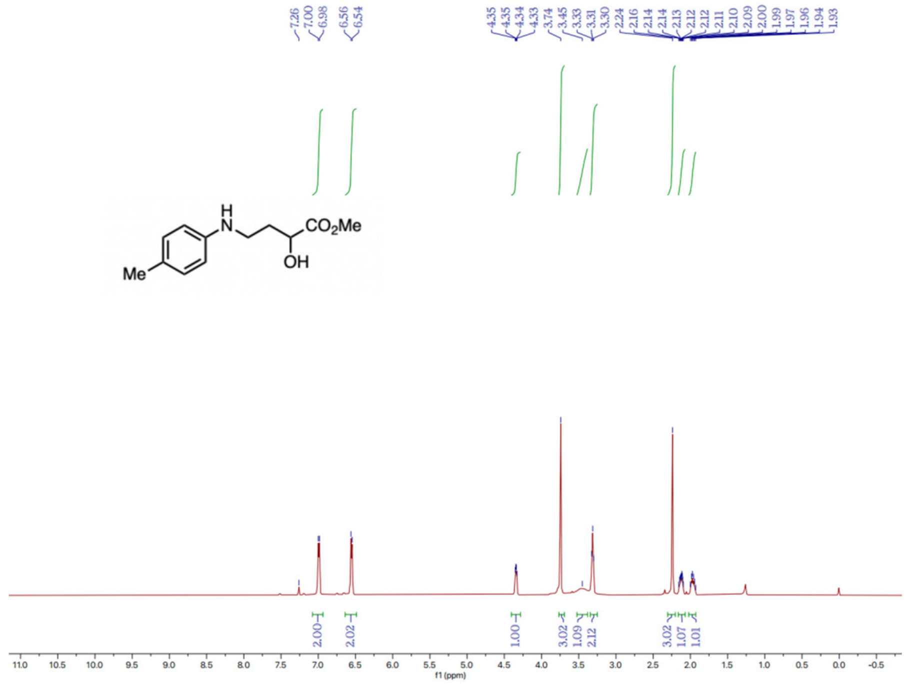

^{1}H NMR spectrum (500 MHz, Chloroform-*d*) of compound **63**

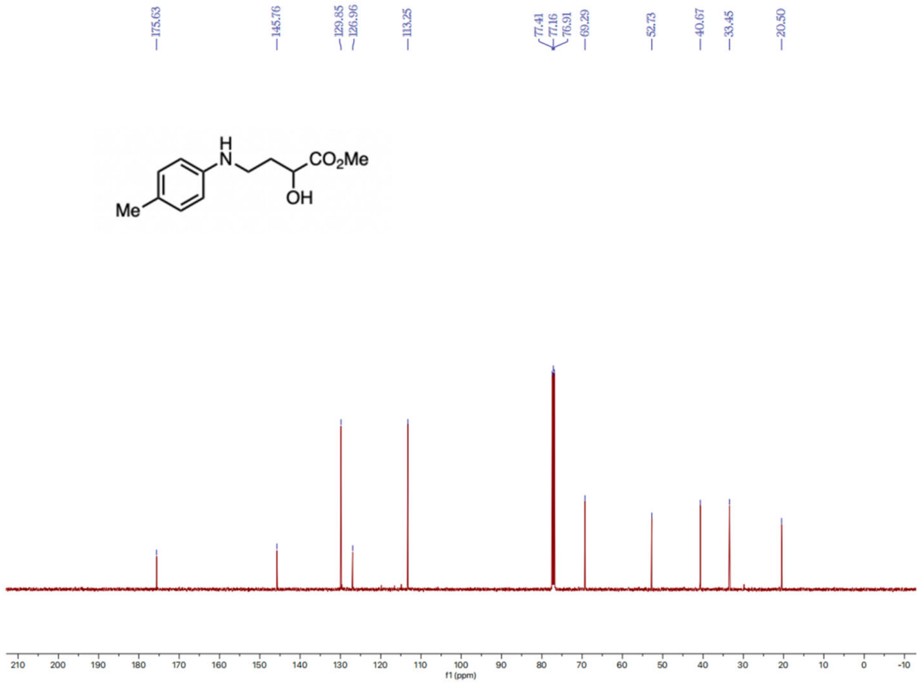

^{13}C NMR spectrum (126 MHz, Chloroform-*d*) of compound **63**

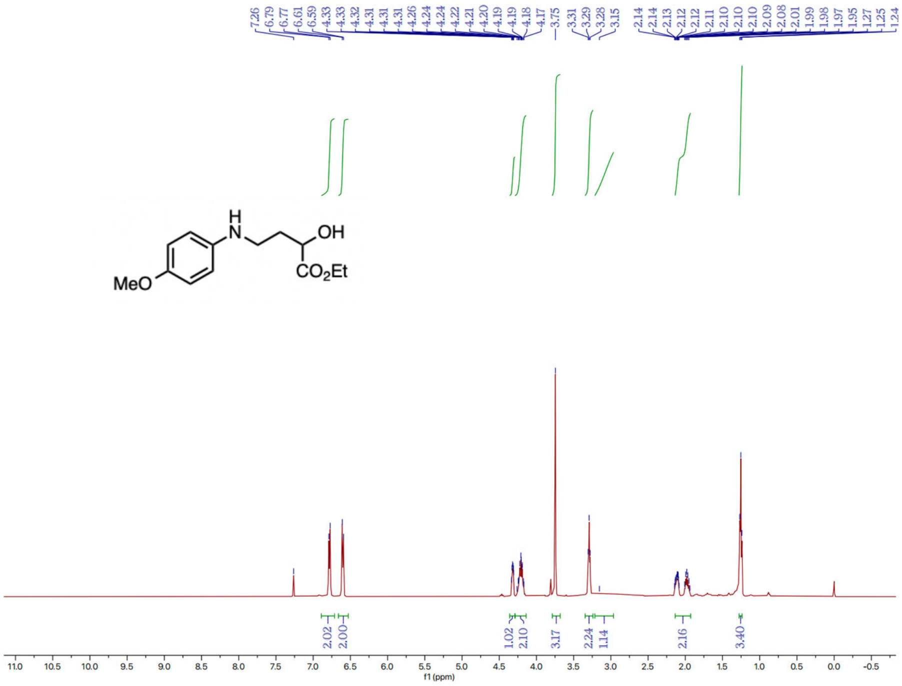

^{1}H NMR spectrum (500 MHz, Chloroform-*d*) of compound **64**

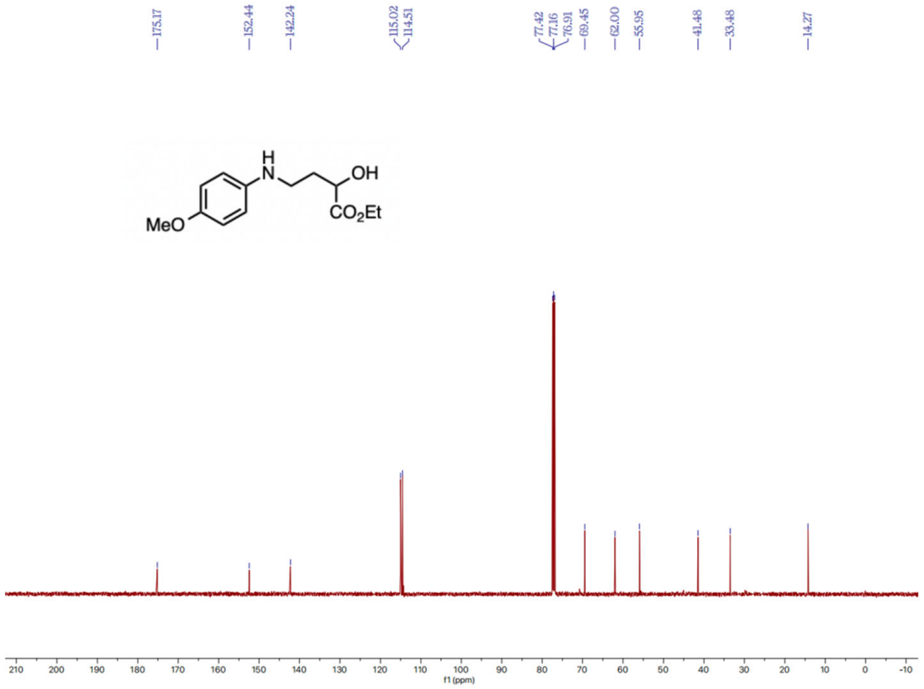

^{13}C NMR spectrum (126 MHz, Chloroform-*d*) of compound **64**

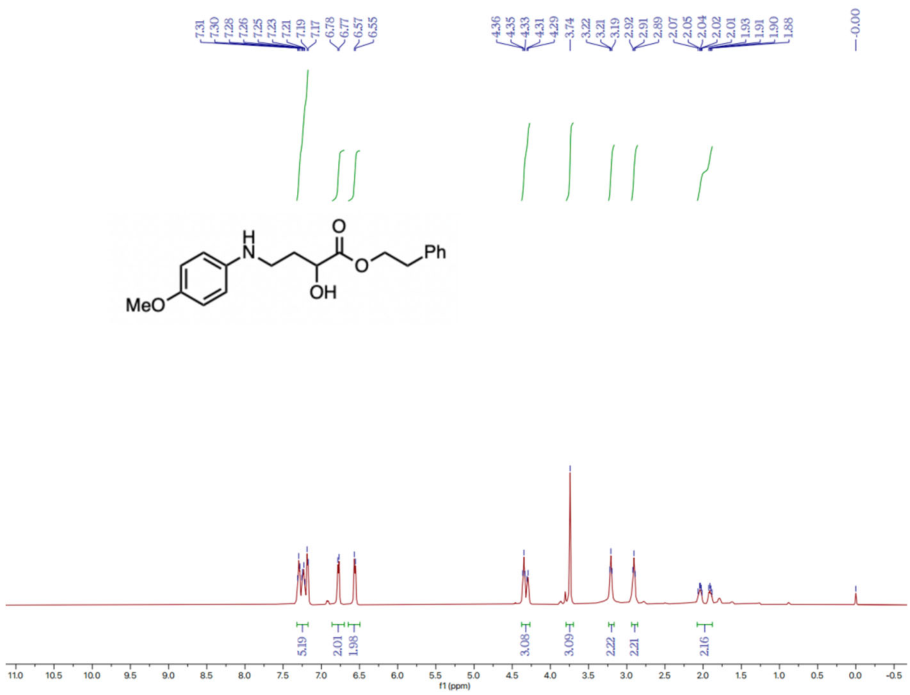

^{1}H NMR spectrum (500 MHz, Chloroform-*d*) of compound **65**

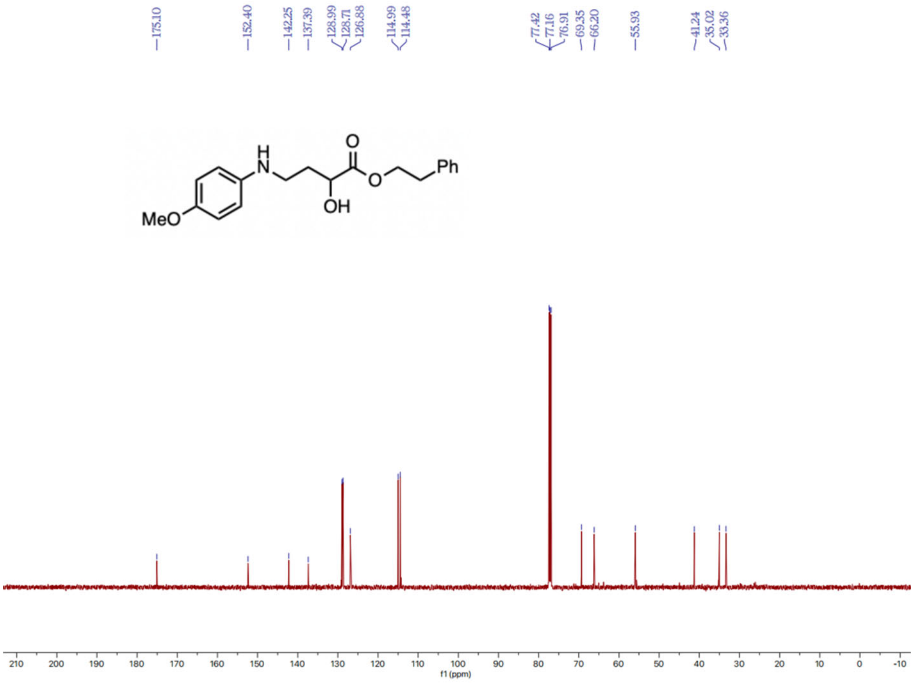

^{13}C NMR spectrum (126 MHz, Chloroform-*d*) of compound **65**

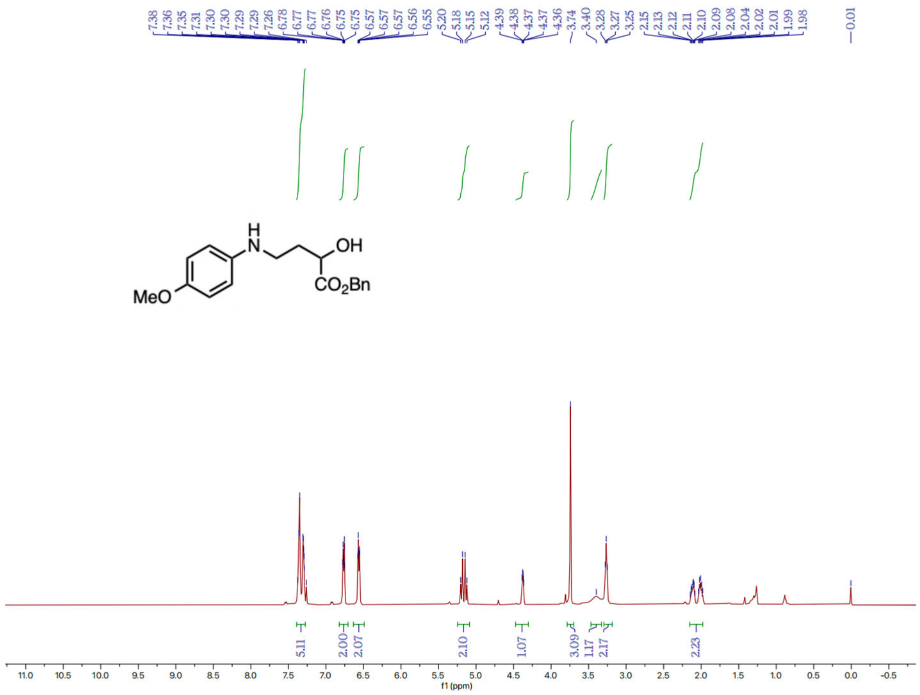

^{1}H NMR spectrum (500 MHz, Chloroform-*d*) of compound **66**

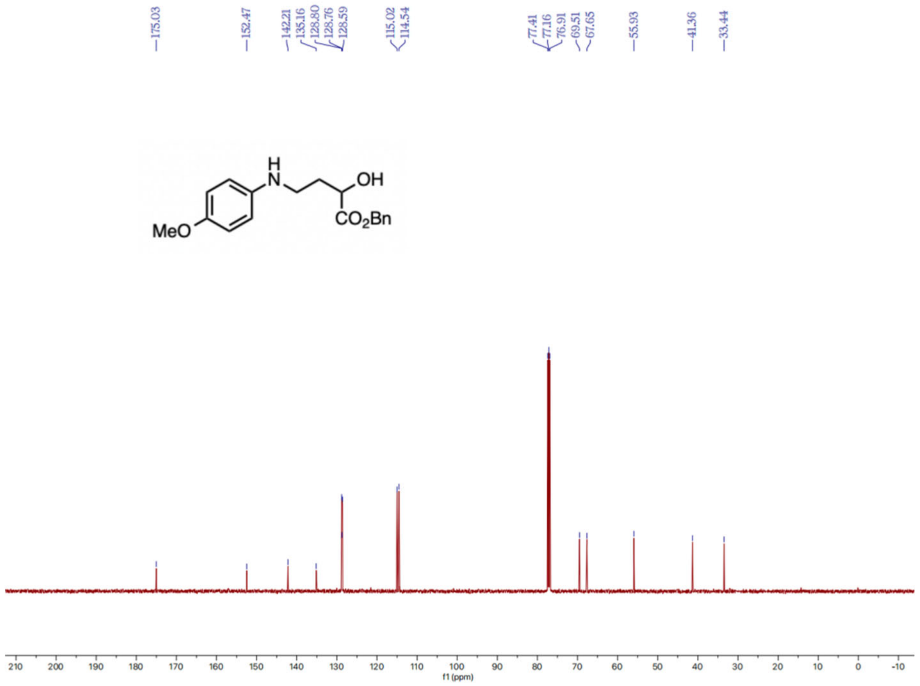

^{13}C NMR spectrum (126 MHz, Chloroform-*d*) of compound **66**

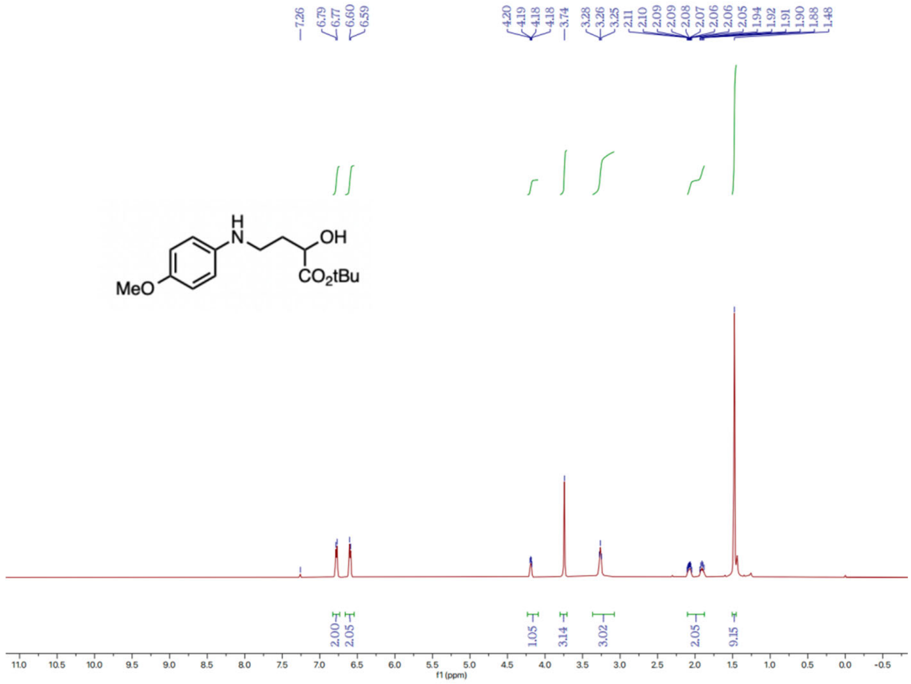

^{1}H NMR spectrum (500 MHz, Chloroform-*d*) of compound **67**

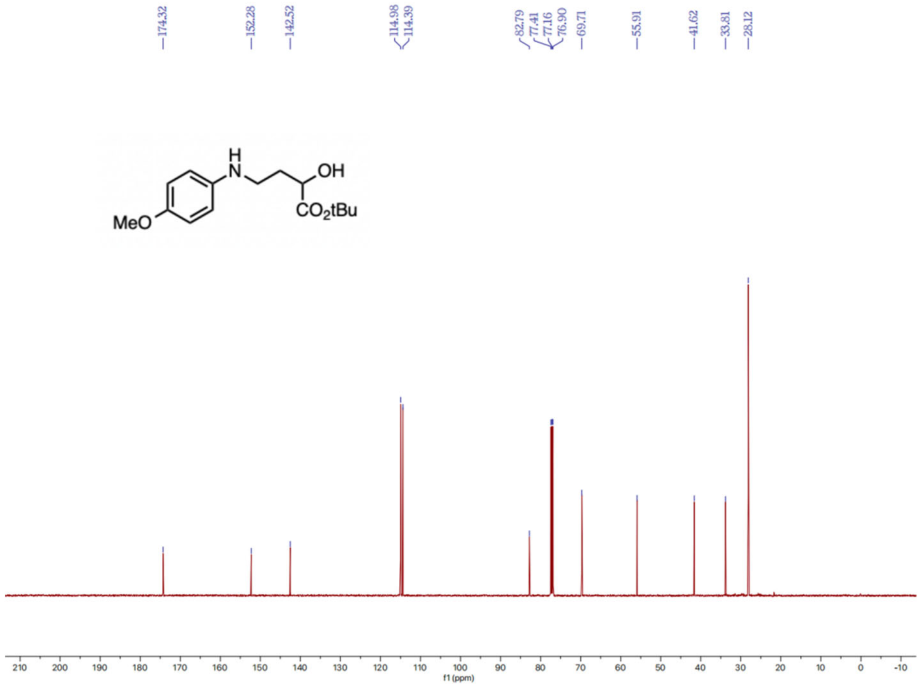

^{13}C NMR spectrum (126 MHz, Chloroform-*d*) of compound **67**

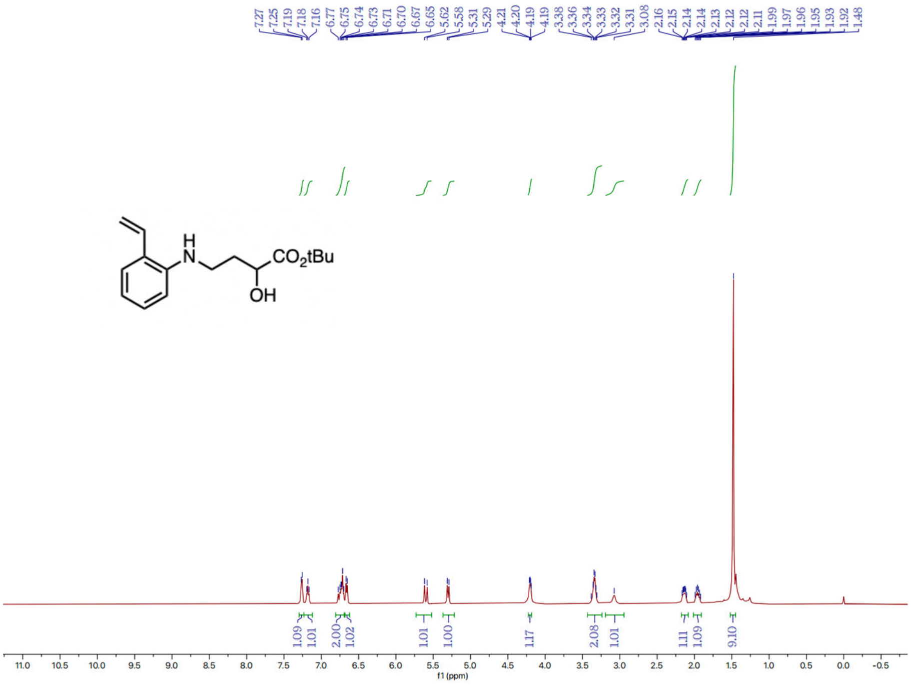

^{1}H NMR spectrum (500 MHz, Chloroform-*d*) of compound **68**

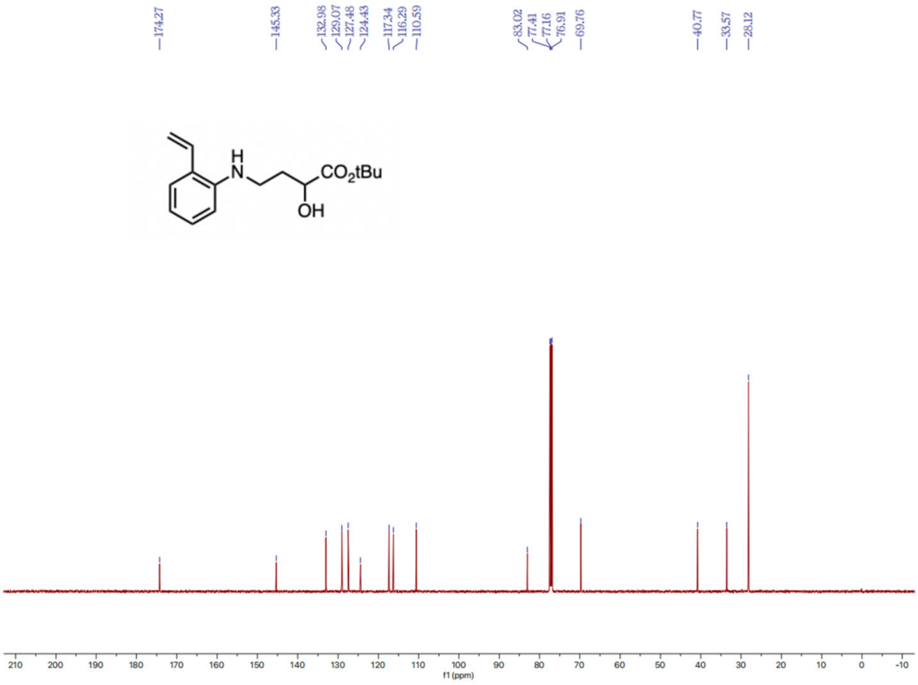

^{13}C NMR spectrum (126 MHz, Chloroform-*d*) of compound **68**

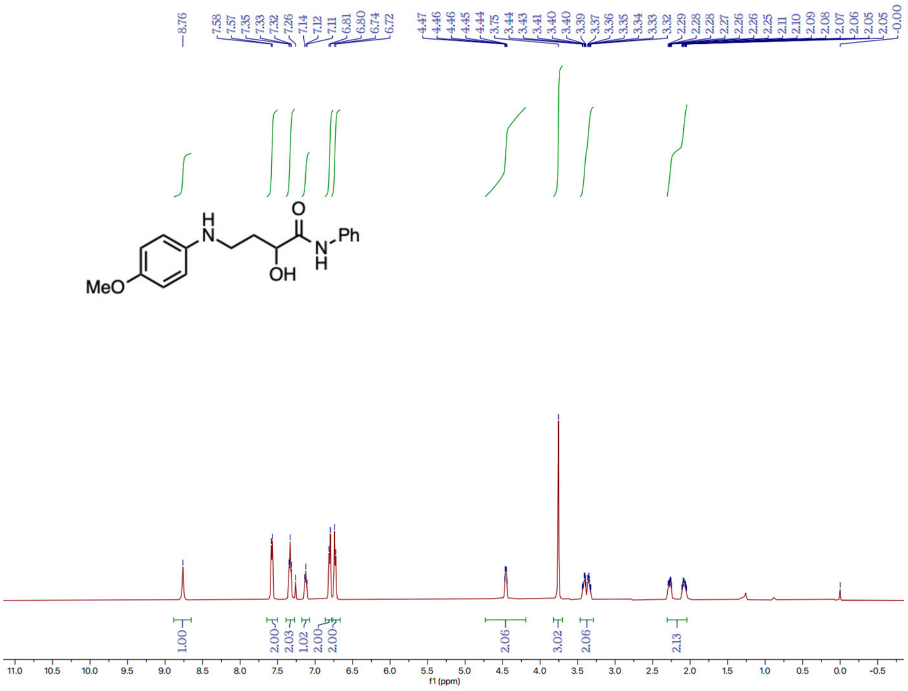

^{1}H NMR spectrum (500 MHz, Chloroform-*d*) of compound **69**

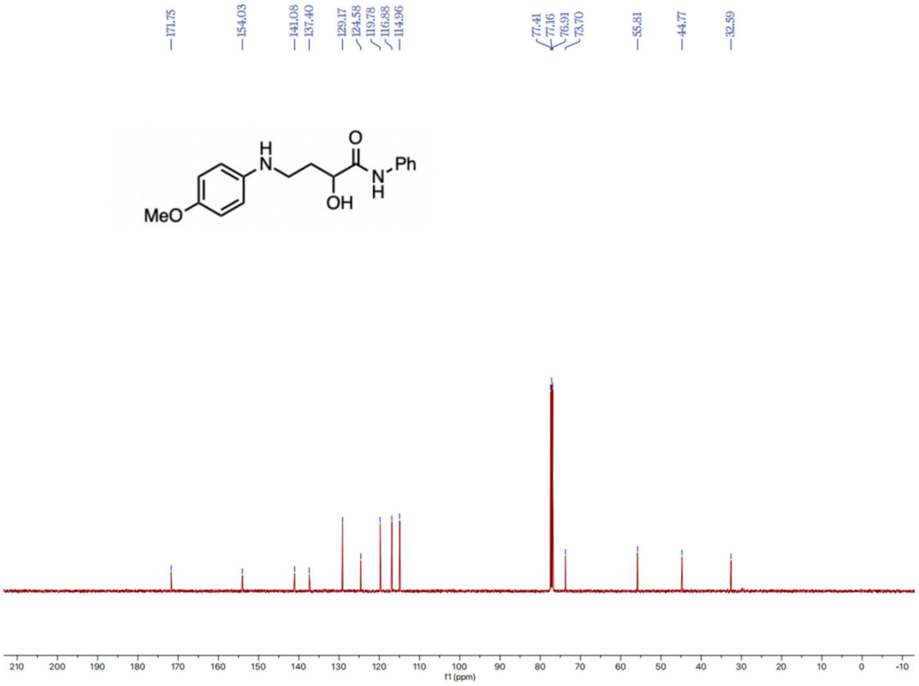

^{13}C NMR spectrum (126 MHz, Chloroform-*d*) of compound **69**

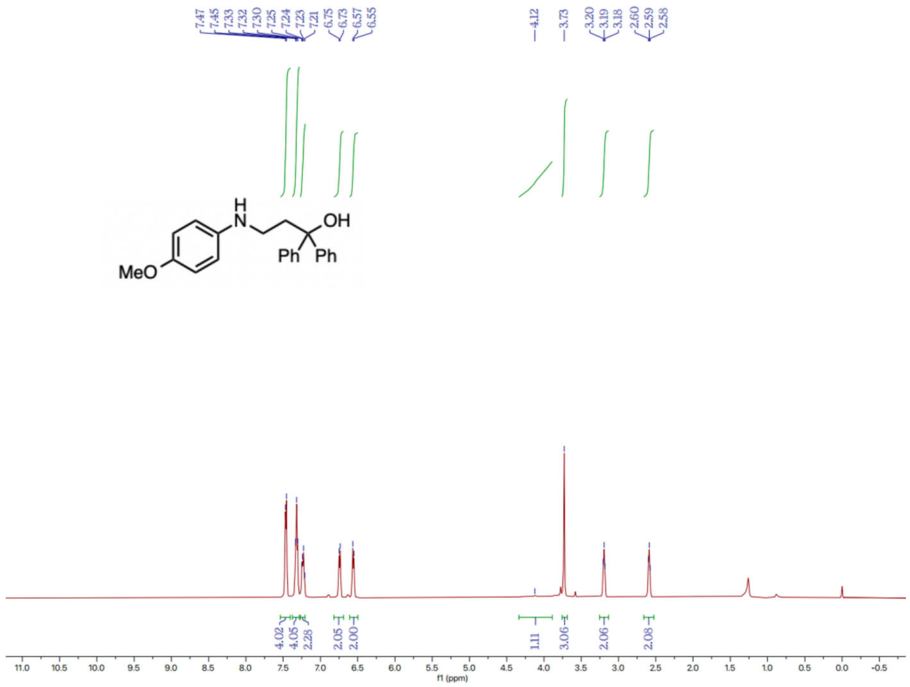

^{1}H NMR spectrum (500 MHz, Chloroform-*d*) of compound **70**

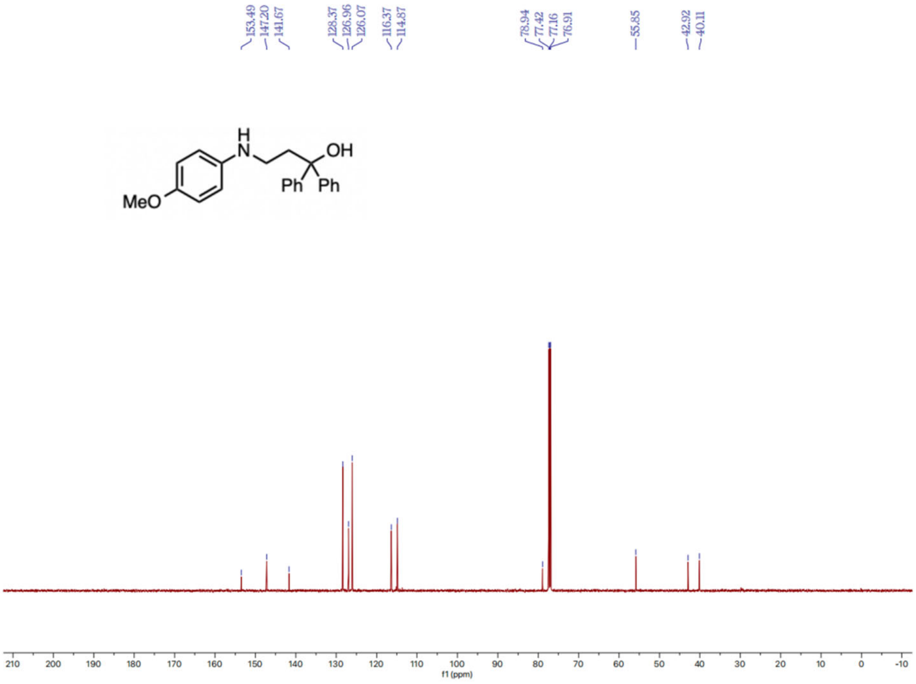

^{13}C NMR spectrum (126 MHz, Chloroform-*d*) of compound **70**

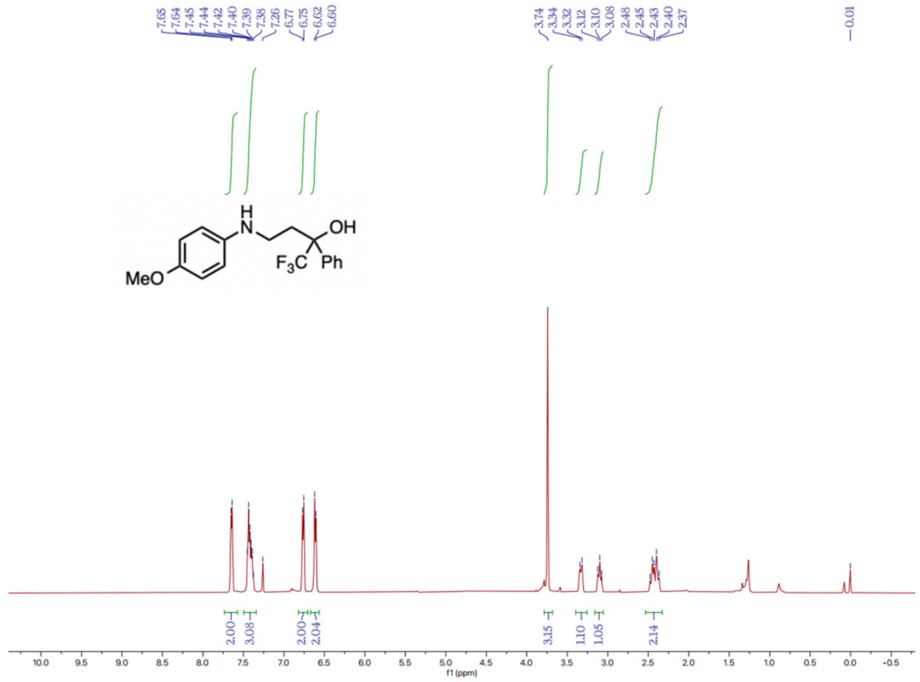

^{1}H NMR spectrum (500 MHz, Chloroform-*d*) of compound **71**

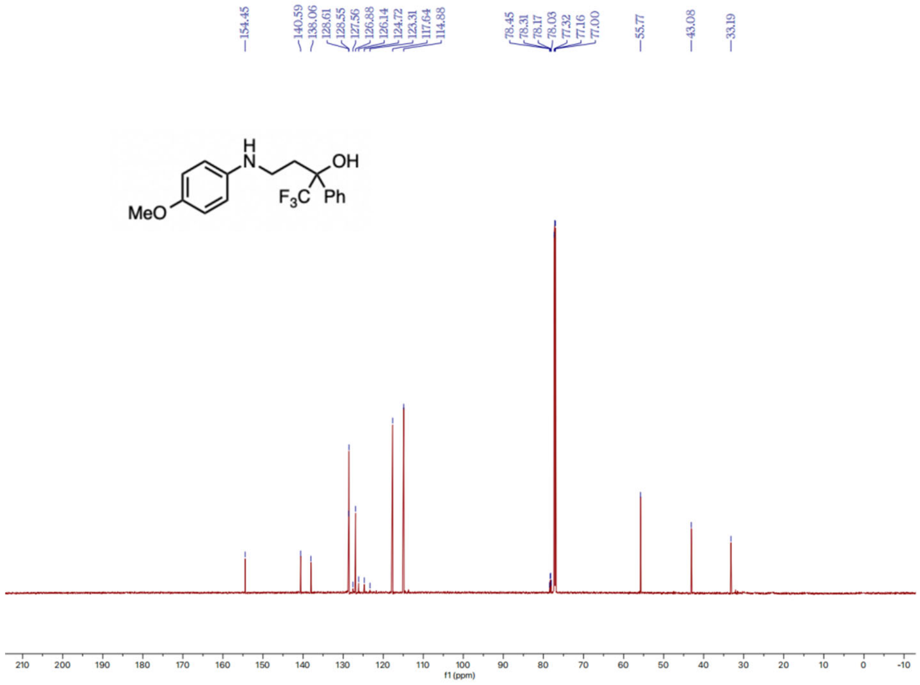

^{13}C NMR spectrum (201 MHz, Chloroform-*d*) of compound **71**

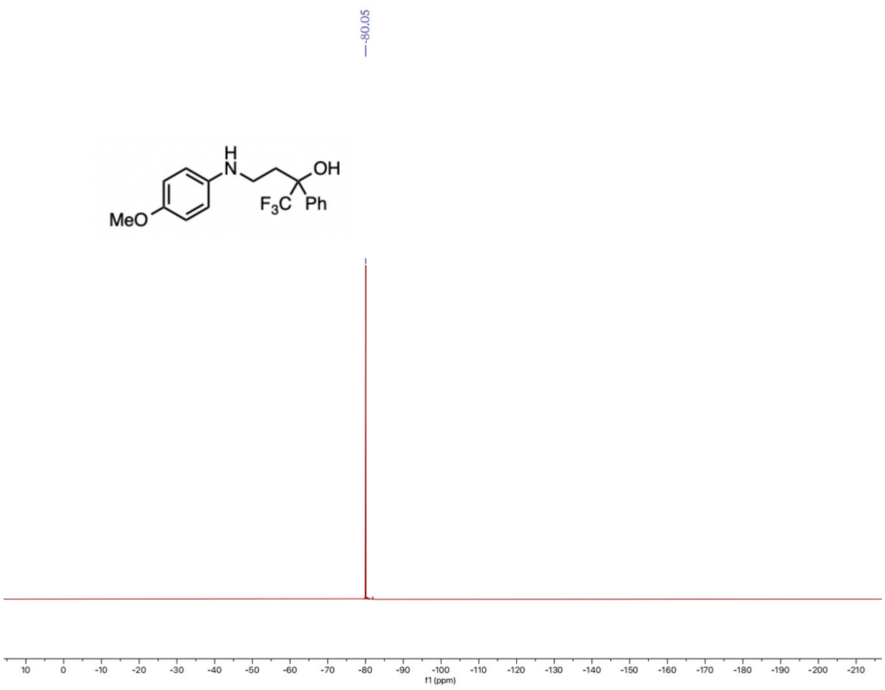

^{19}F NMR spectrum (471 MHz, Chloroform-*d*) of compound **71**

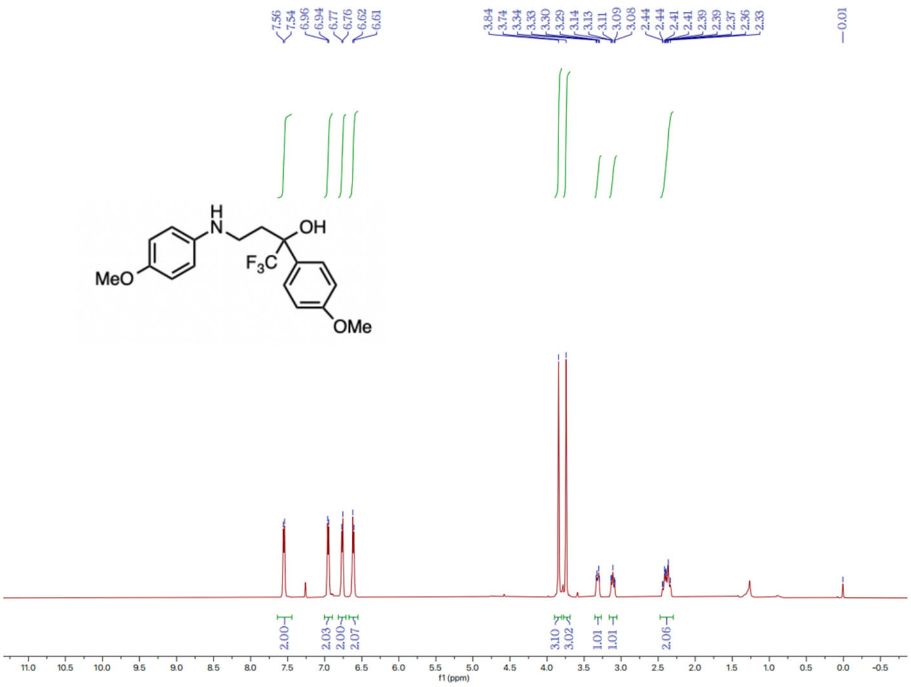

^{1}H NMR spectrum (500 MHz, Chloroform-*d*) of compound **72**

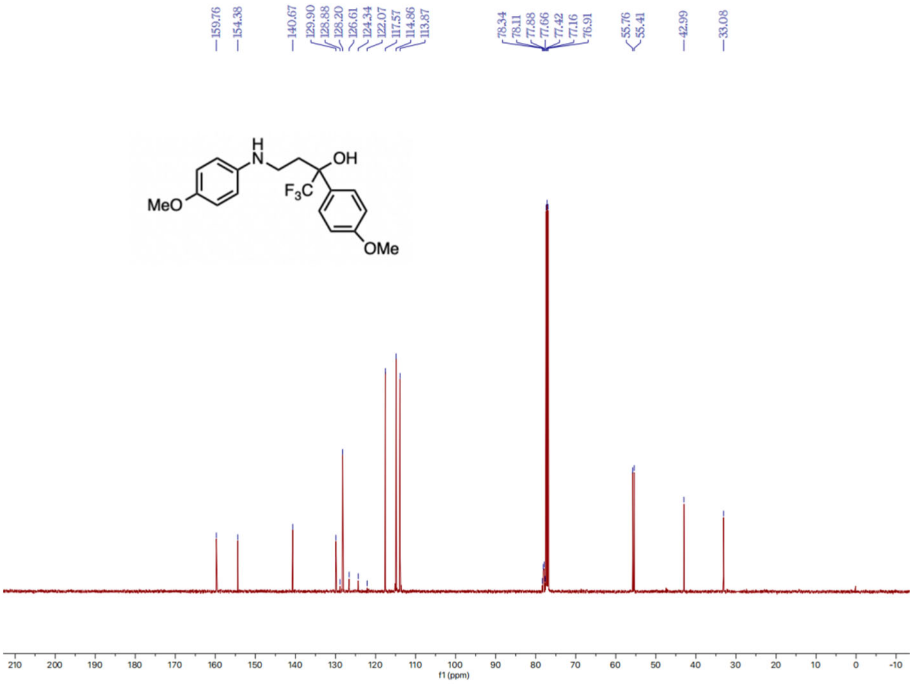

^{13}C NMR spectrum (126 MHz, Chloroform-*d*) of compound **72**

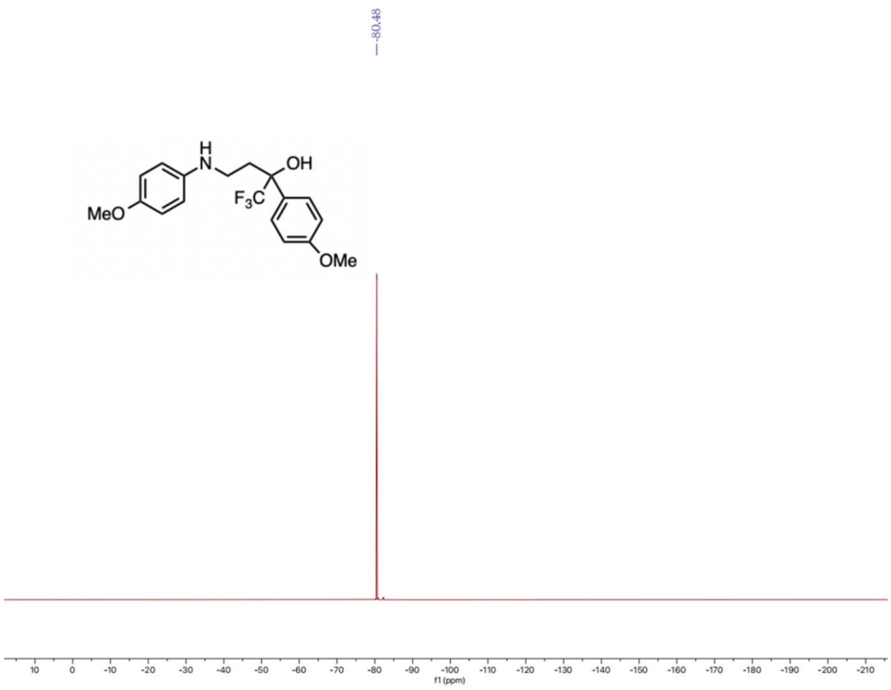

^{19}F NMR spectrum (471 MHz, Chloroform-*d*) of compound **72**

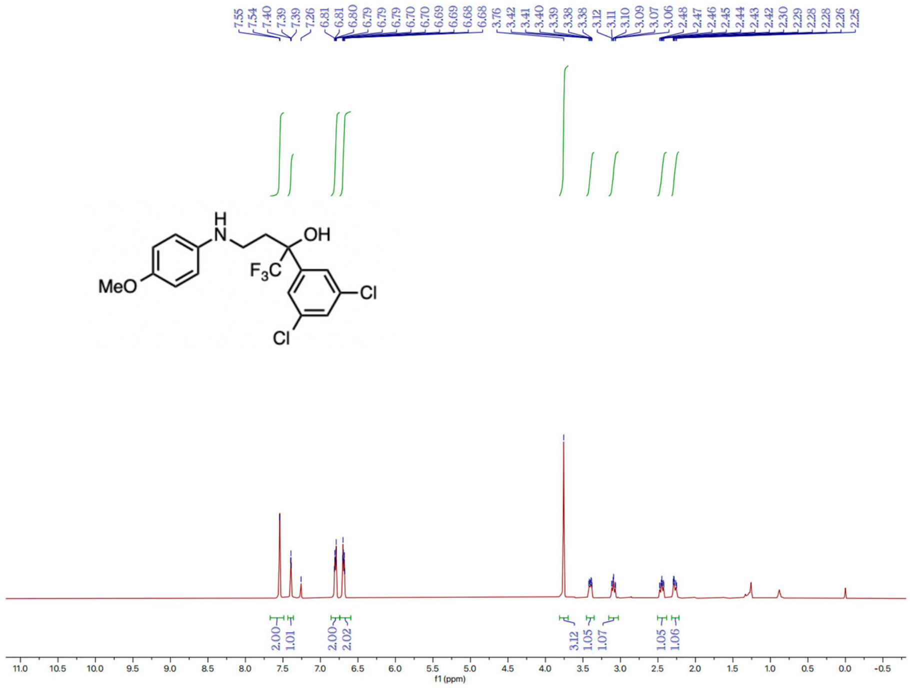

^{1}H NMR spectrum (500 MHz, Chloroform-*d*) of compound **73**

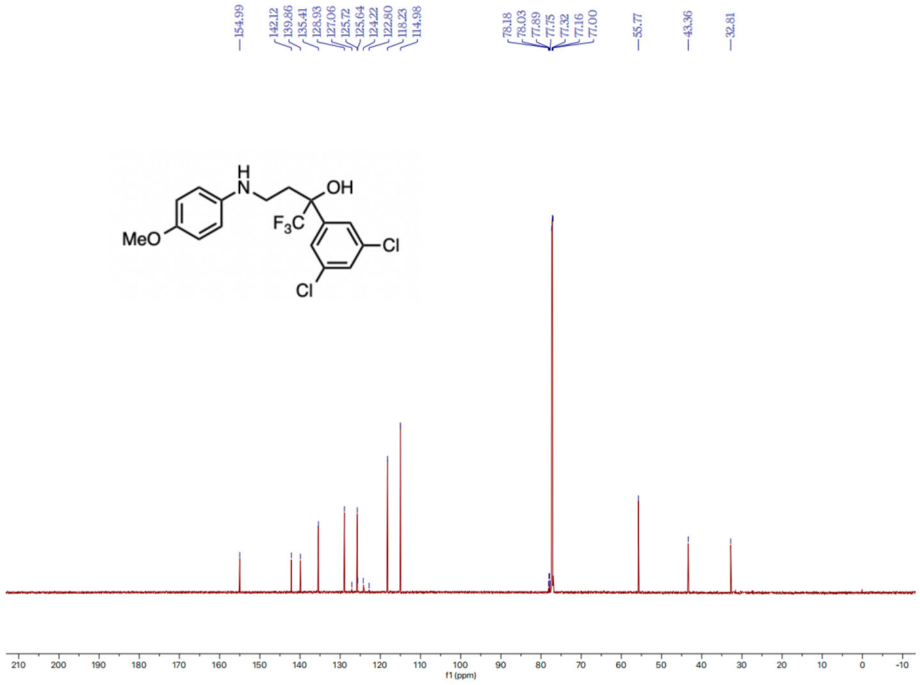

^{13}C NMR spectrum (201 MHz, Chloroform-*d*) of compound **73**

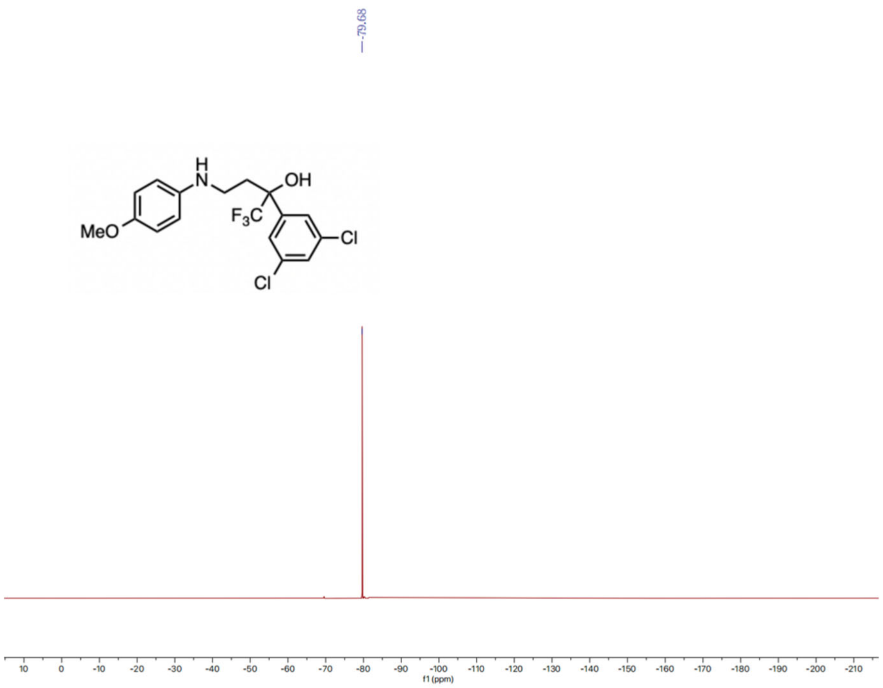

^{19}F NMR spectrum (471 MHz, Chloroform-*d*) of compound **73**

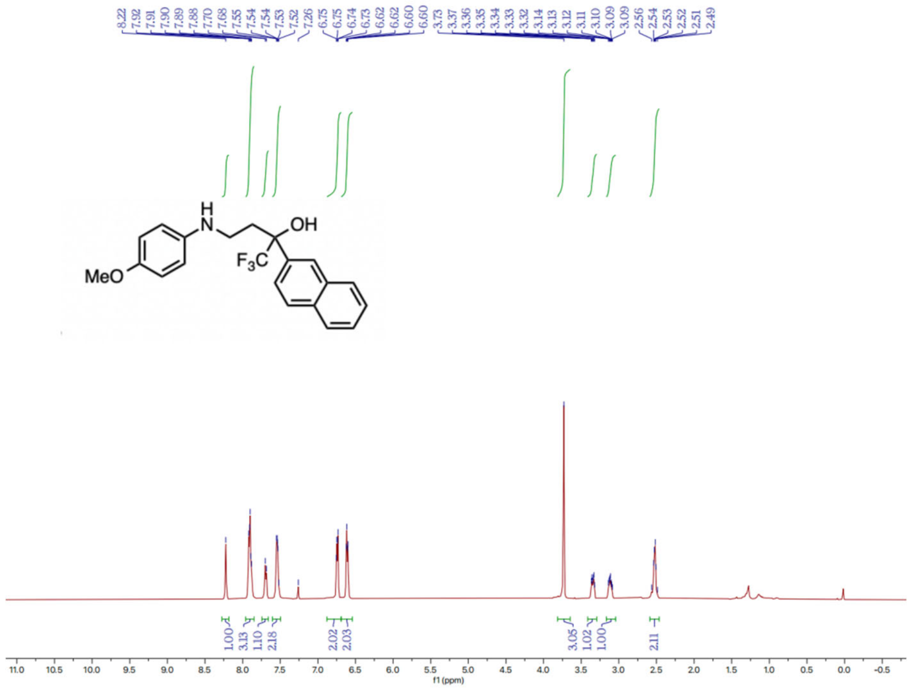

^{1}H NMR spectrum (500 MHz, Chloroform-*d*) of compound **74**

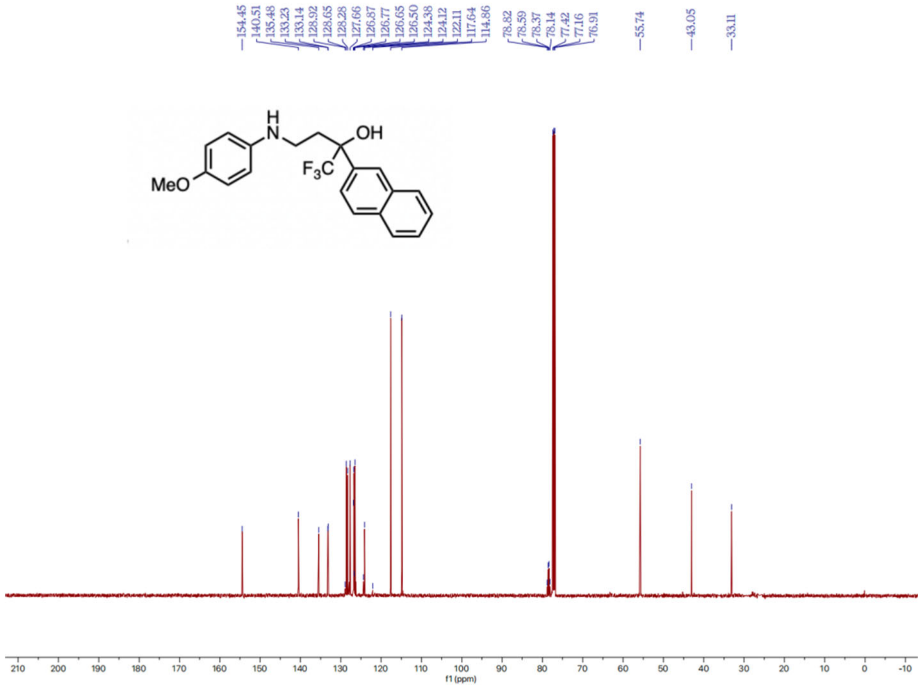

^{13}C NMR spectrum (126 MHz, Chloroform-*d*) of compound **74**

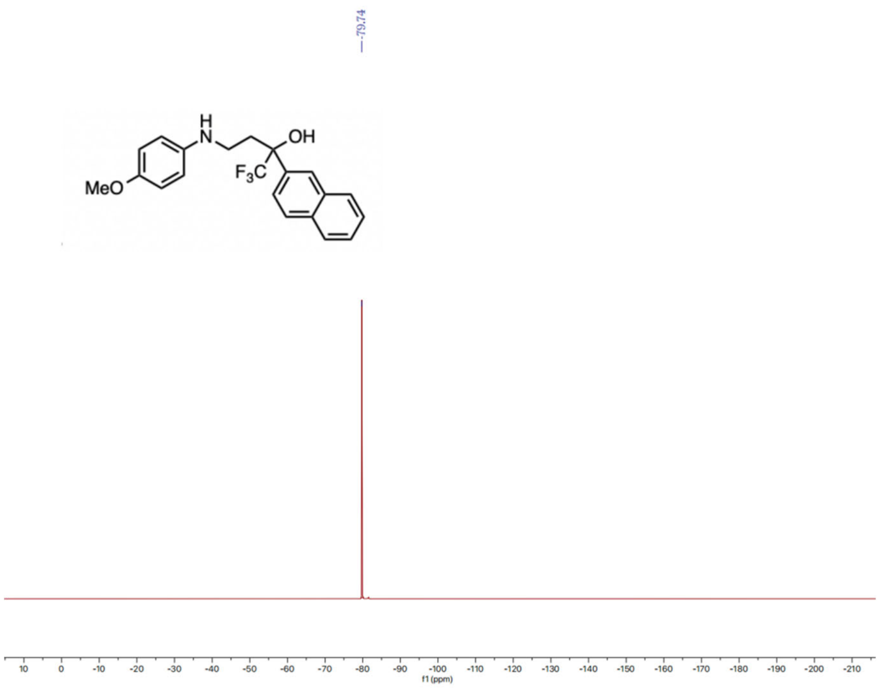

^{19}F NMR spectrum (471 MHz, Chloroform-*d*) of compound **74**

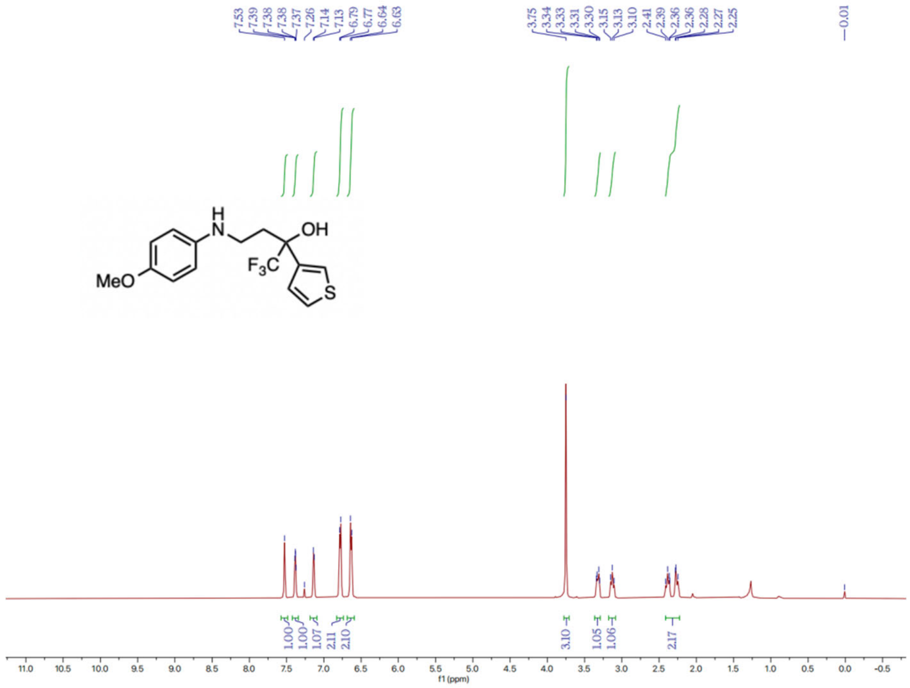

^{1}H NMR spectrum (500 MHz, Chloroform-*d*) of compound **75**

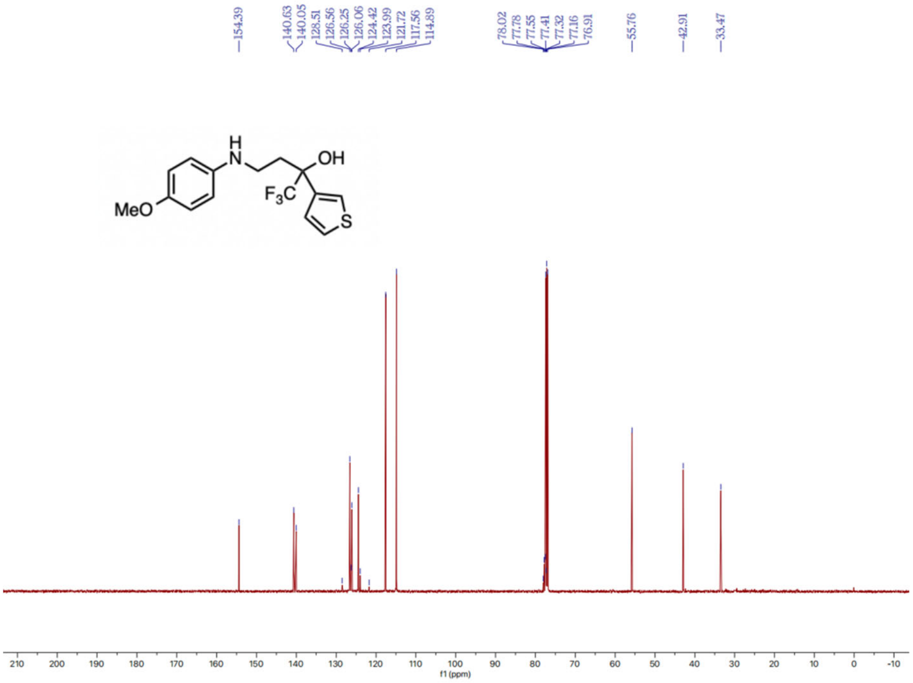

^{13}C NMR spectrum (126 MHz, Chloroform-*d*) of compound **75**

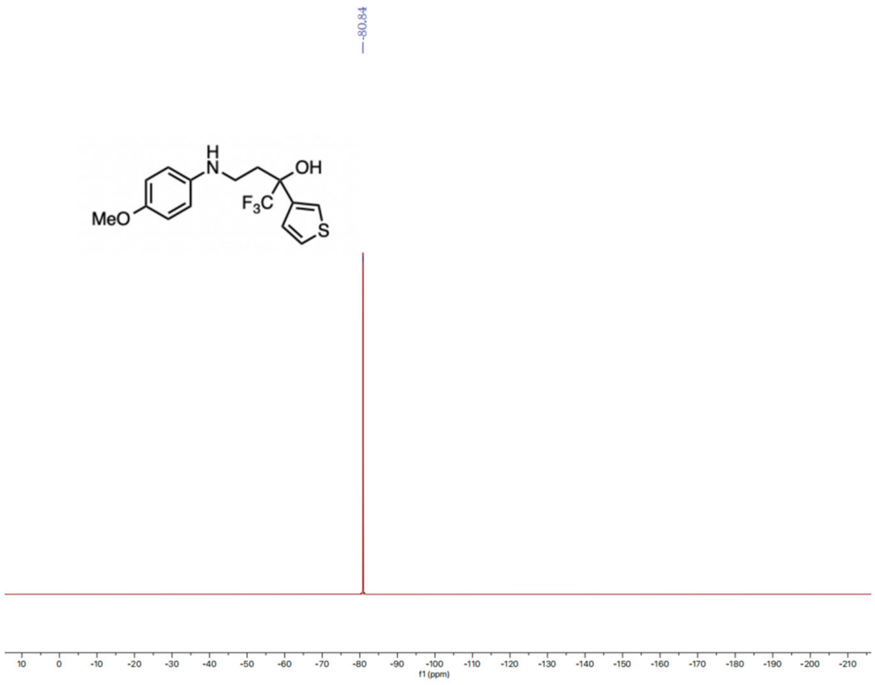

^{19}F NMR spectrum (471 MHz, Chloroform-*d*) of compound **75**

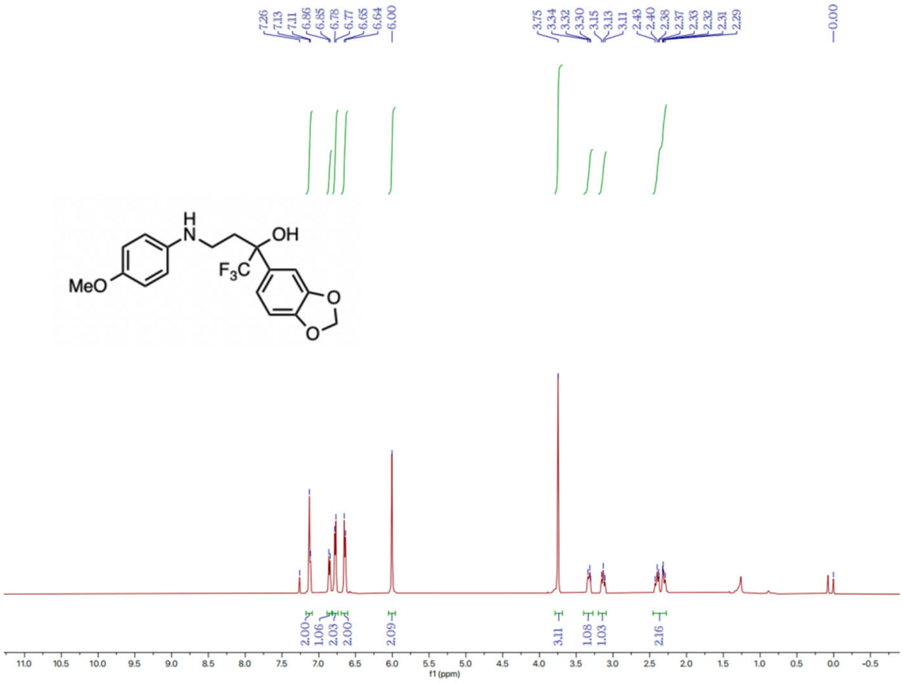

^{1}H NMR spectrum (500 MHz, Chloroform-*d*) of compound **76**

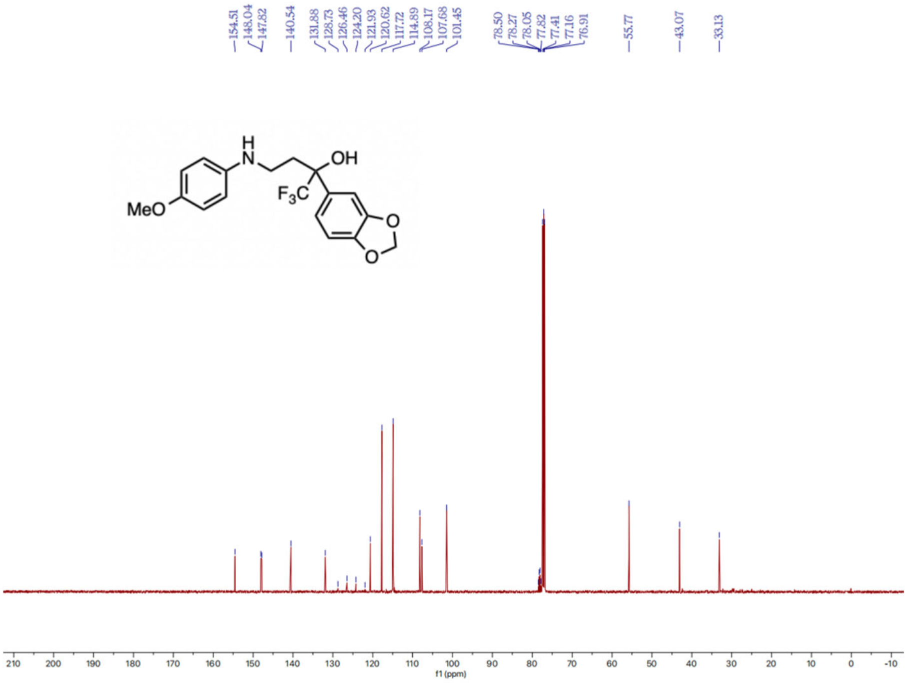

^{13}C NMR spectrum (126 MHz, Chloroform-*d*) of compound **76**

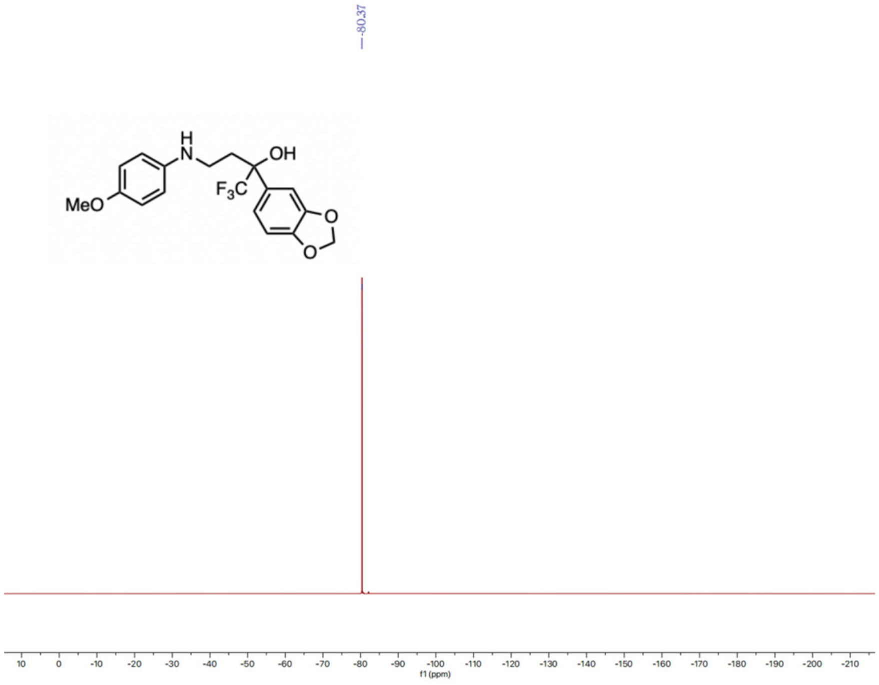

^{19}F NMR spectrum (471 MHz, Chloroform-*d*) of compound **76**

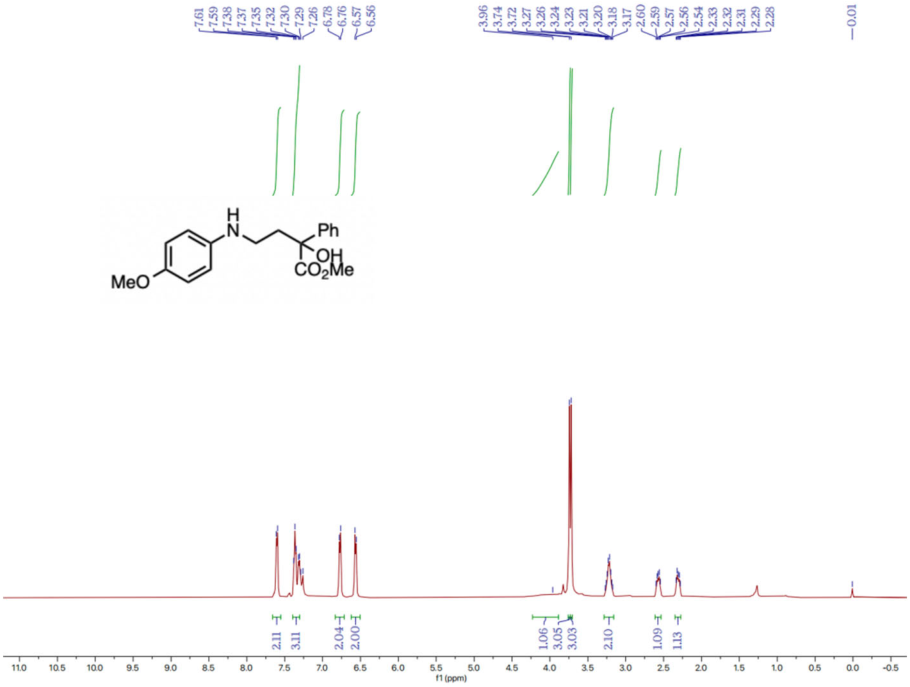

^{1}H NMR spectrum (500 MHz, Chloroform-*d*) of compound **77**

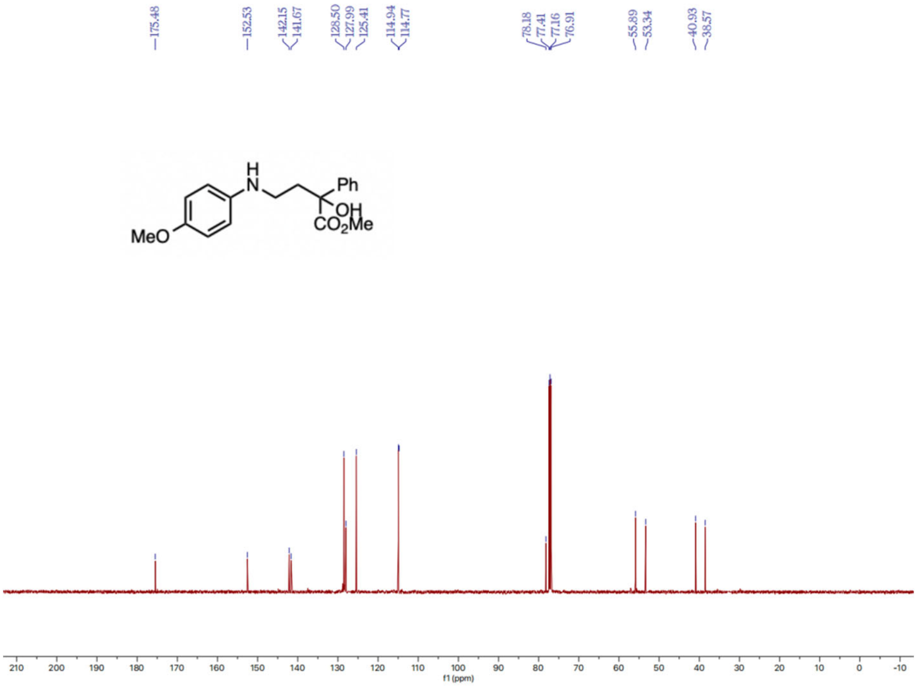

^{13}C NMR spectrum (126 MHz, Chloroform-*d*) of compound **77**

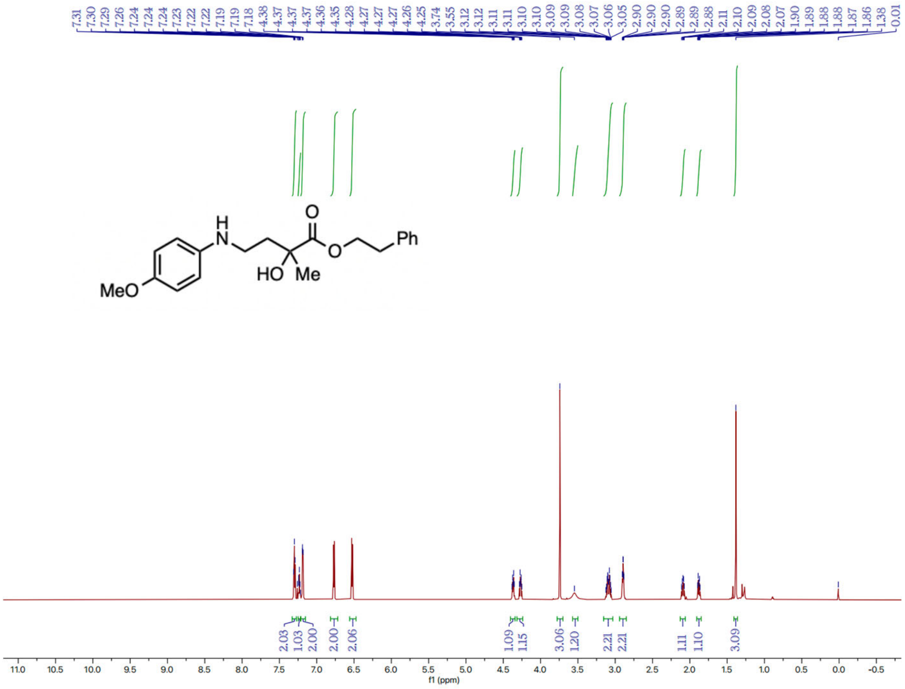

^{1}H NMR spectrum (800 MHz, Chloroform-*d*) of compound **78**

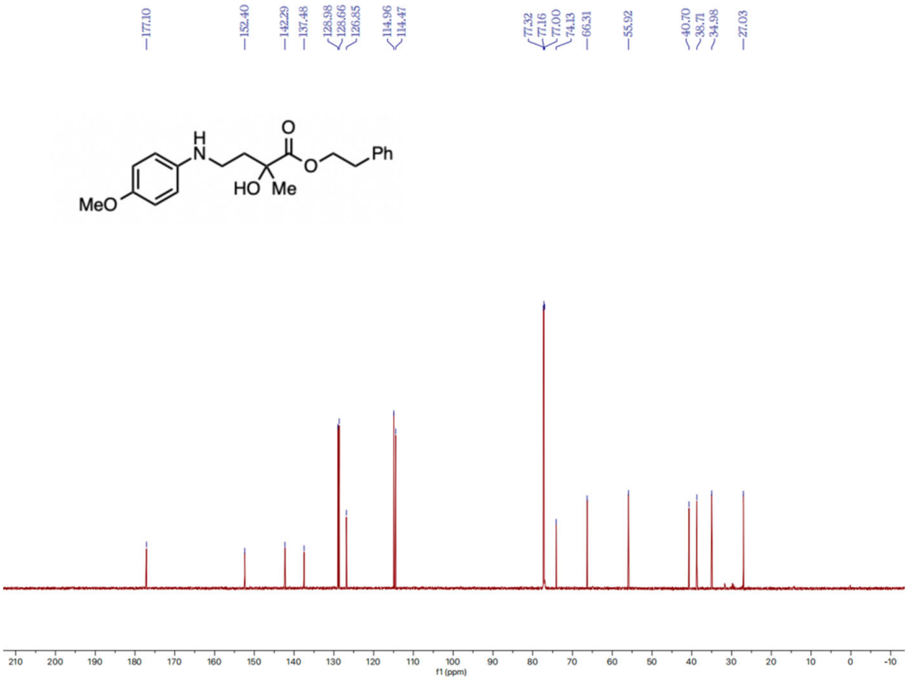

^{13}C NMR spectrum (201 MHz, Chloroform-*d*) of compound **78**

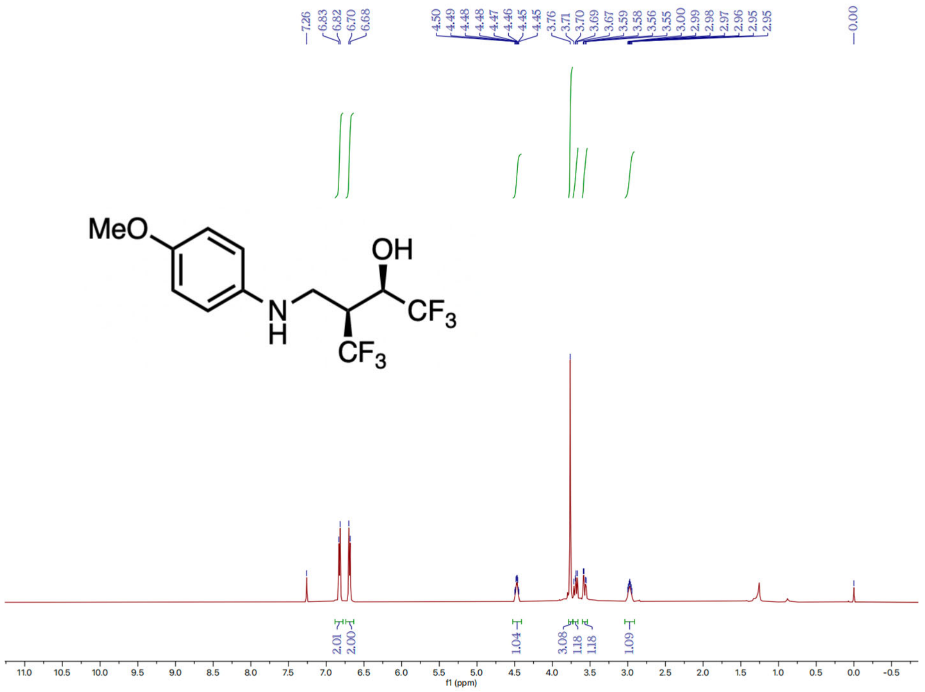

^{1}H NMR spectrum (500 MHz, Chloroform-*d*) of compound **(±)-79**

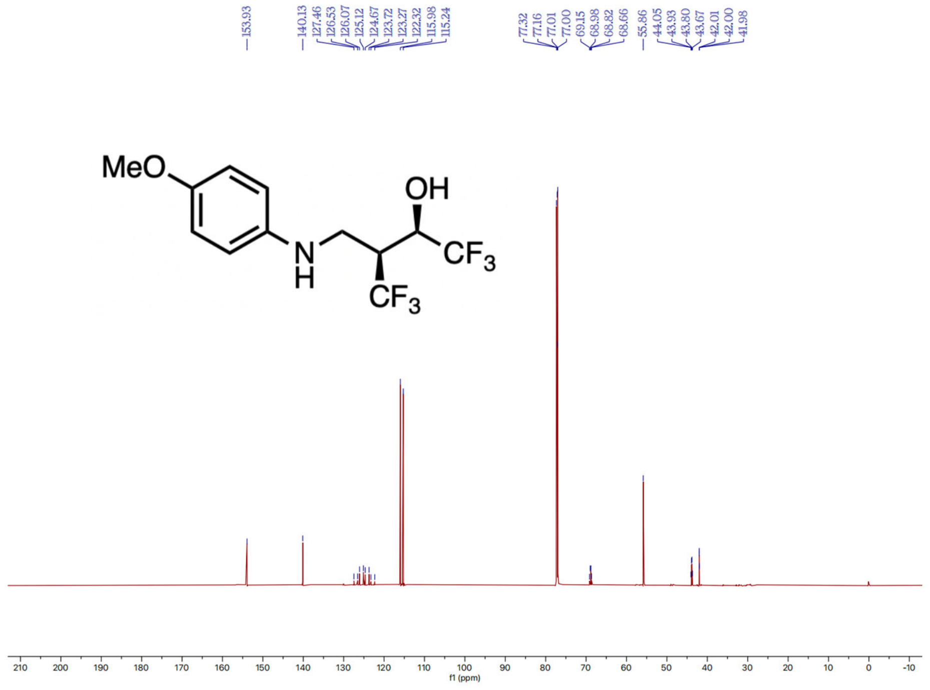

^{13}C NMR spectrum (201 MHz, Chloroform-*d*) of compound **(±)-79**

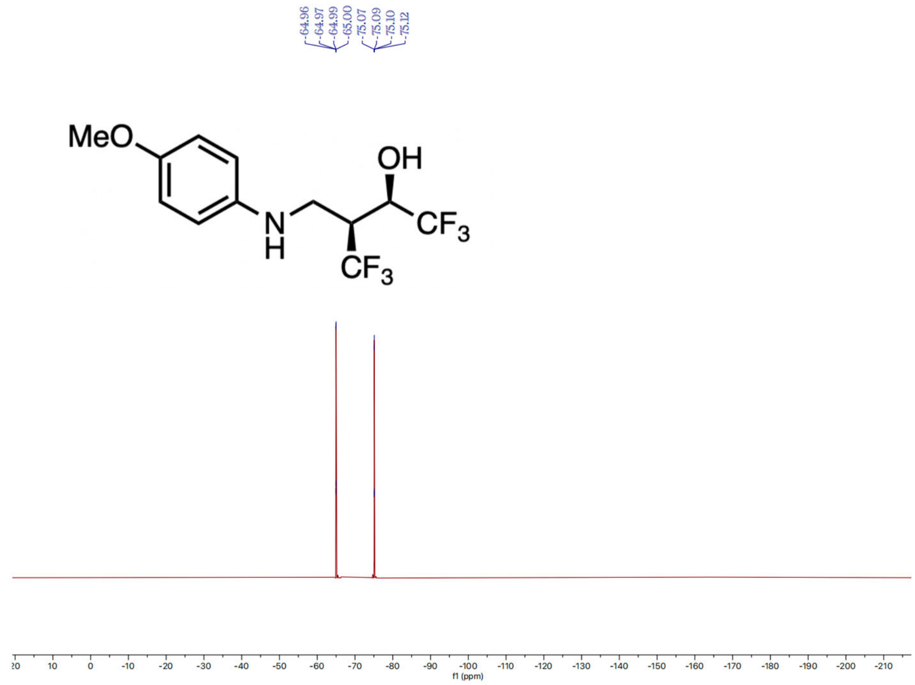

^{19}F NMR spectrum (471 MHz, Chloroform-*d*) of compound **(±)-79**

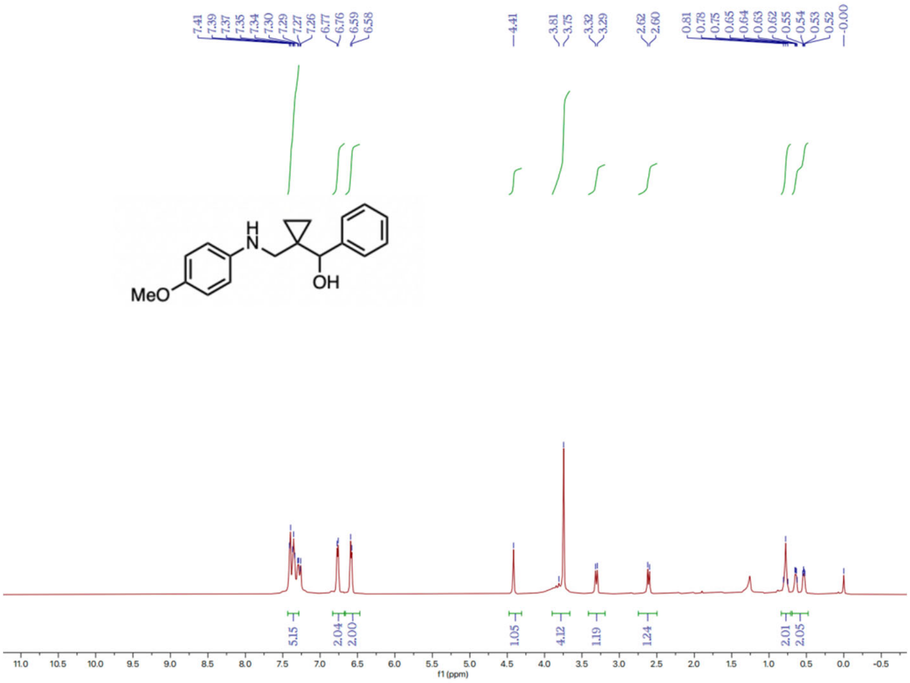

^{1}H NMR spectrum (500 MHz, Chloroform-*d*) of compound **80**

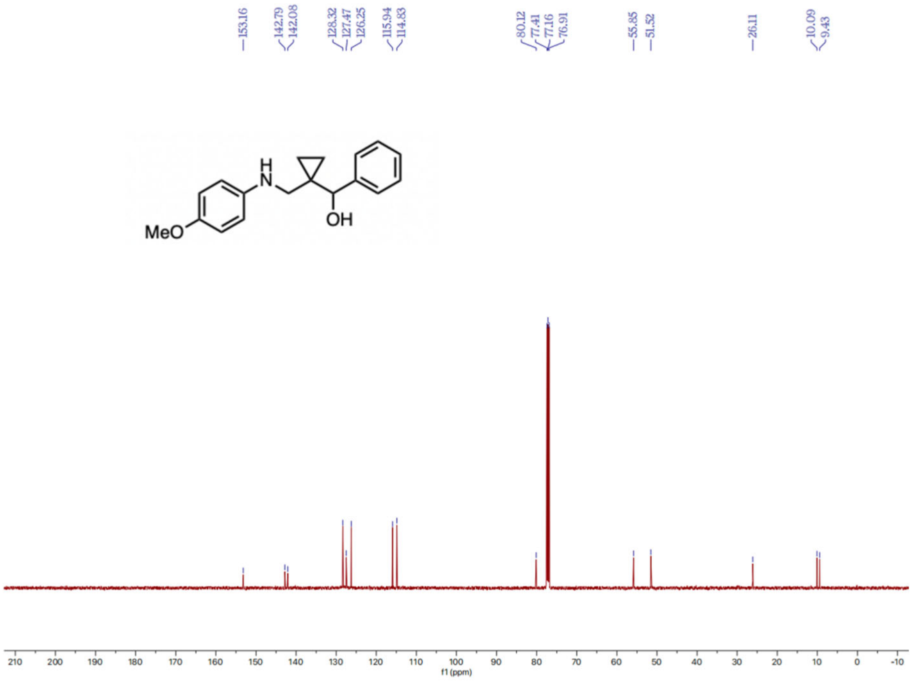

^{13}C NMR spectrum (126 MHz, Chloroform-*d*) of compound **80**

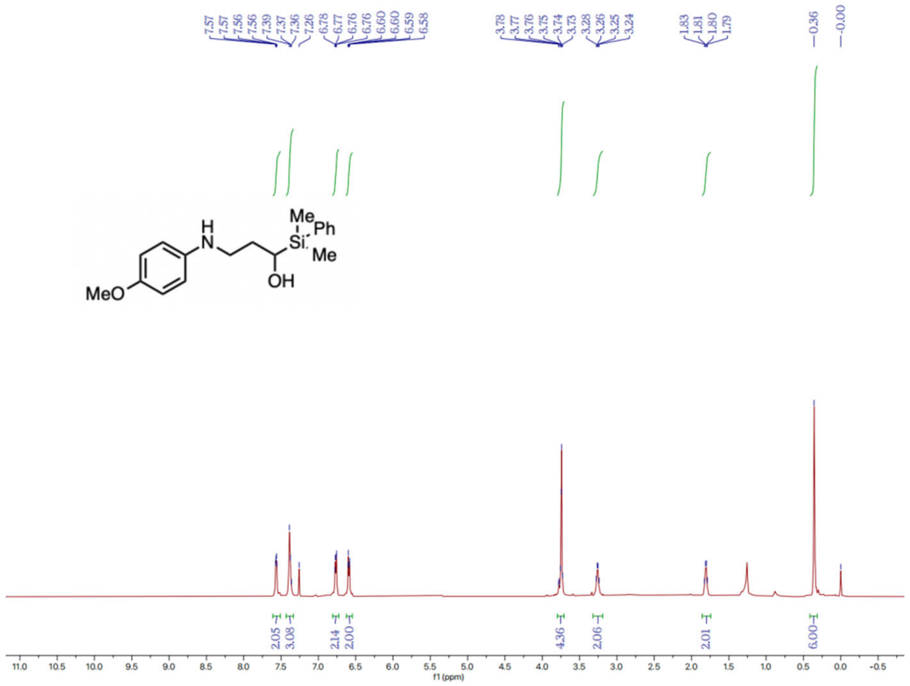

^{1}H NMR spectrum (500 MHz, Chloroform-*d*) of compound **81**

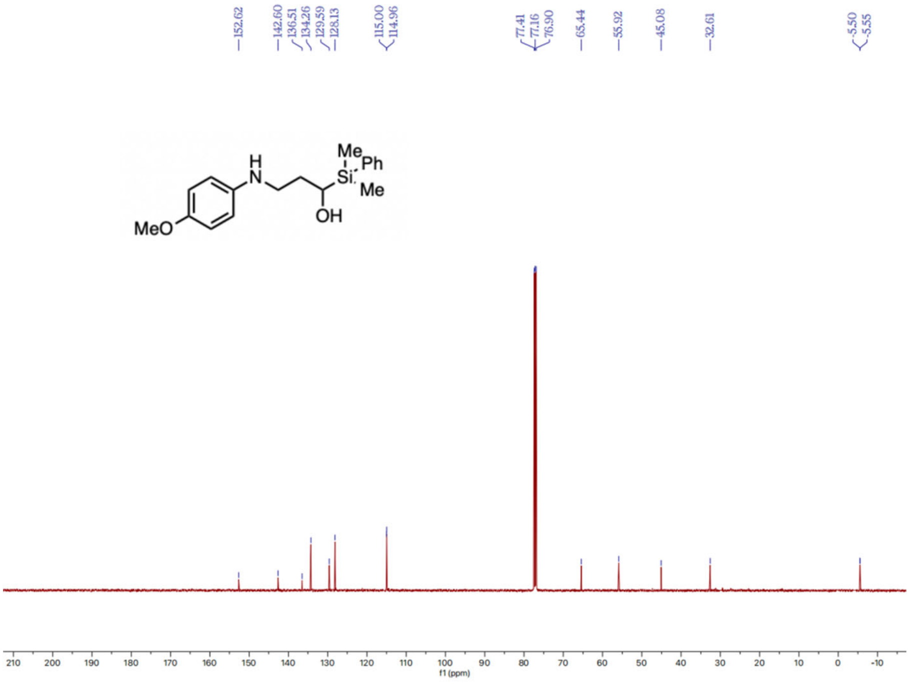

^{13}C NMR spectrum (126 MHz, Chloroform-*d*) of compound **81**

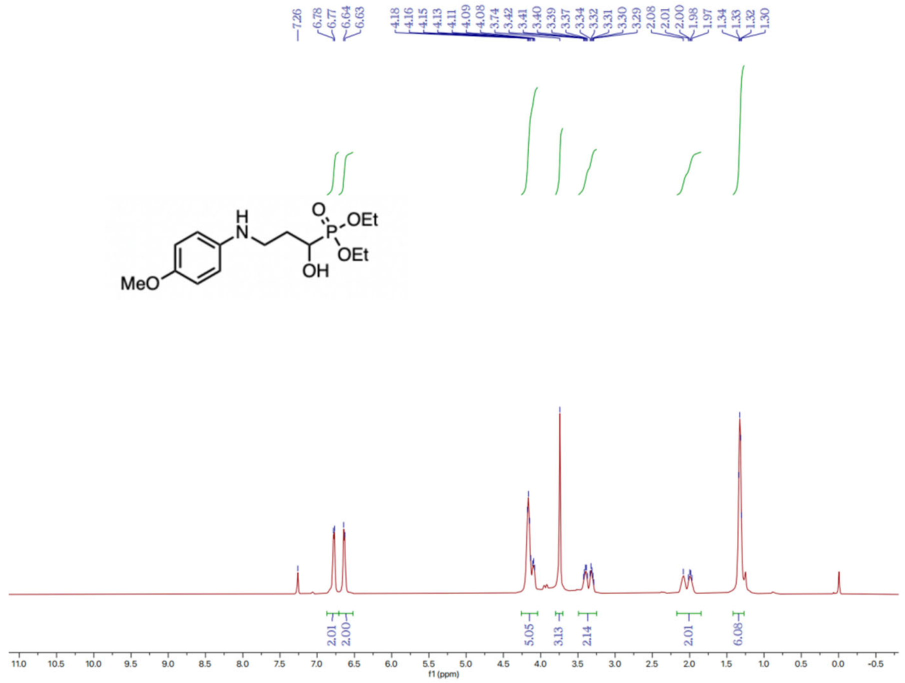

^{1}H NMR spectrum (500 MHz, Chloroform-*d*) of compound **82**

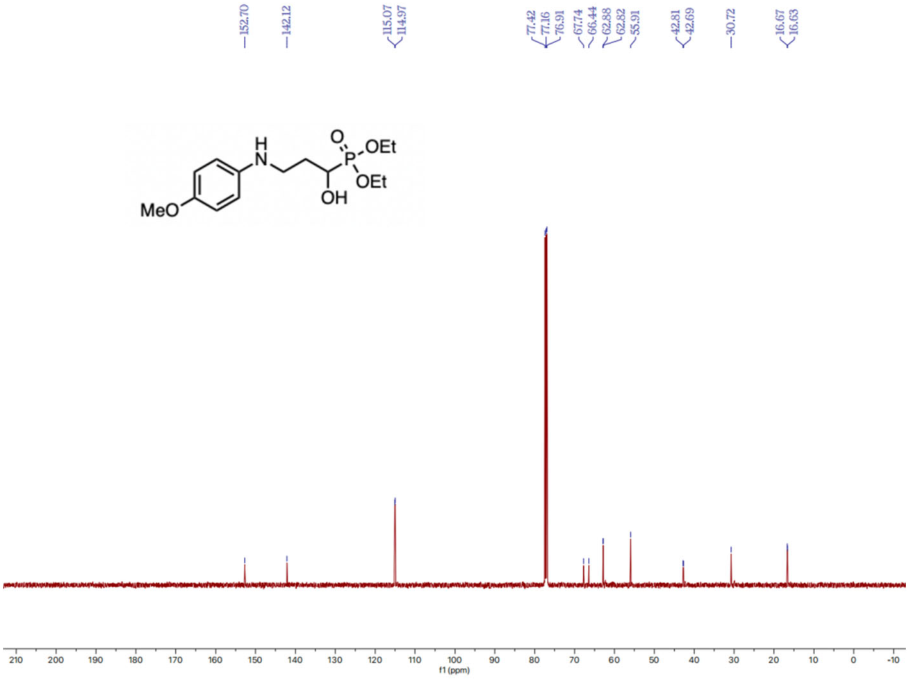

^{13}C NMR spectrum (126 MHz, Chloroform-*d*) of compound **82**

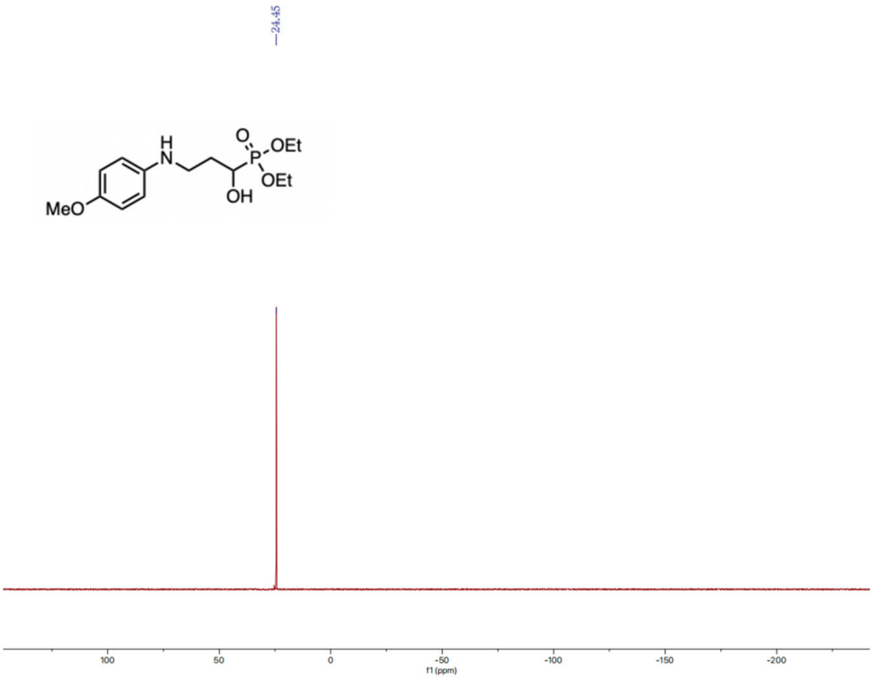

^{31}P NMR spectrum (202 MHz, Chloroform-*d*) of compound **82**

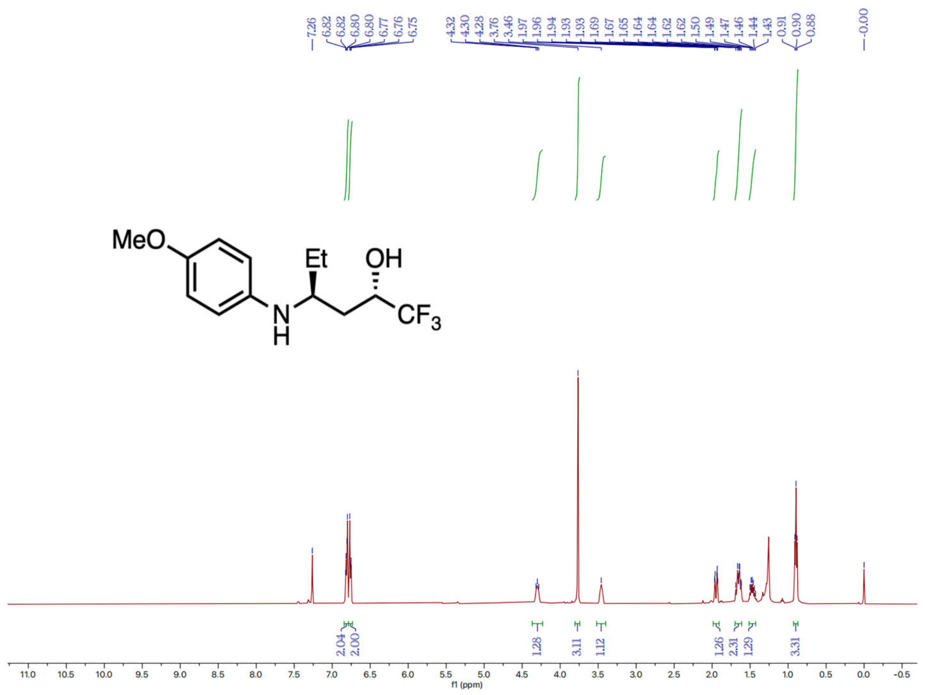

^{1}H NMR spectrum (500 MHz, Chloroform-*d*) of compound **(±)-83**

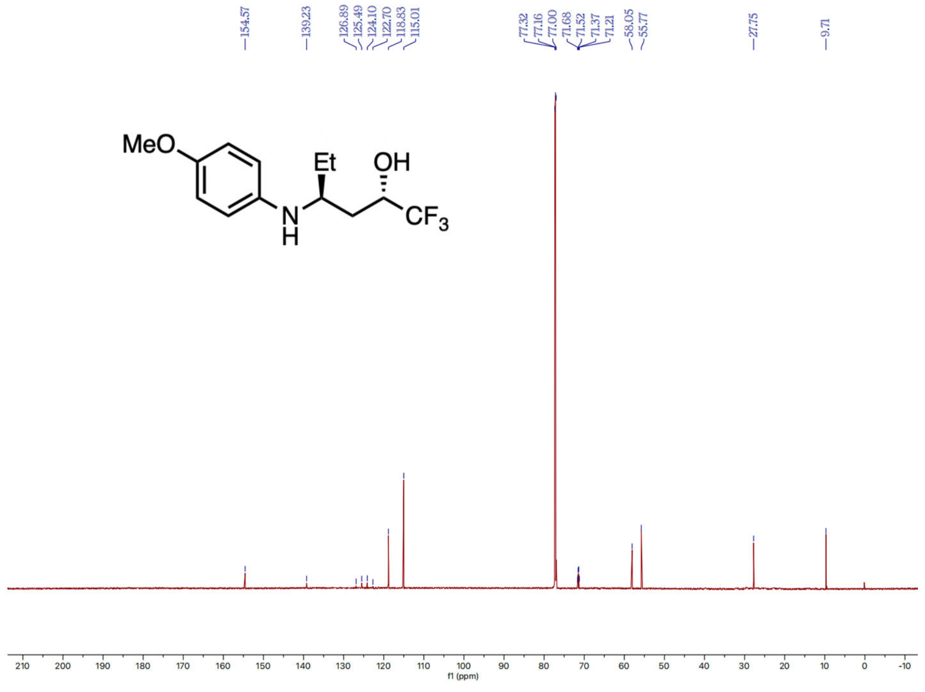

^{13}C NMR spectrum (201 MHz, Chloroform-*d*) of compound **(±)-83**

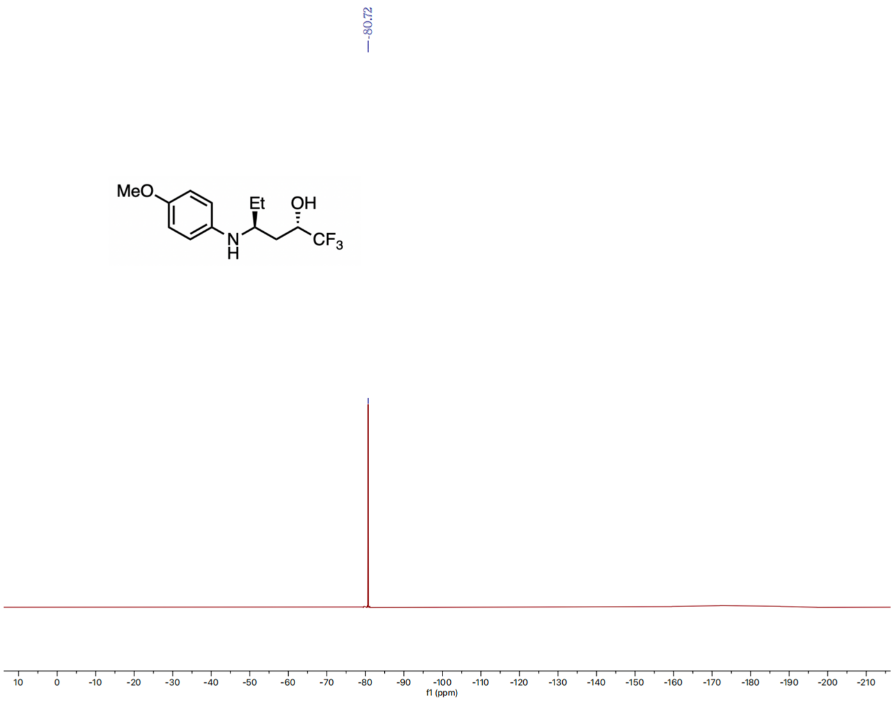

^{19}F NMR spectrum (471 MHz, Chloroform-*d*) of compound **(±)-83**

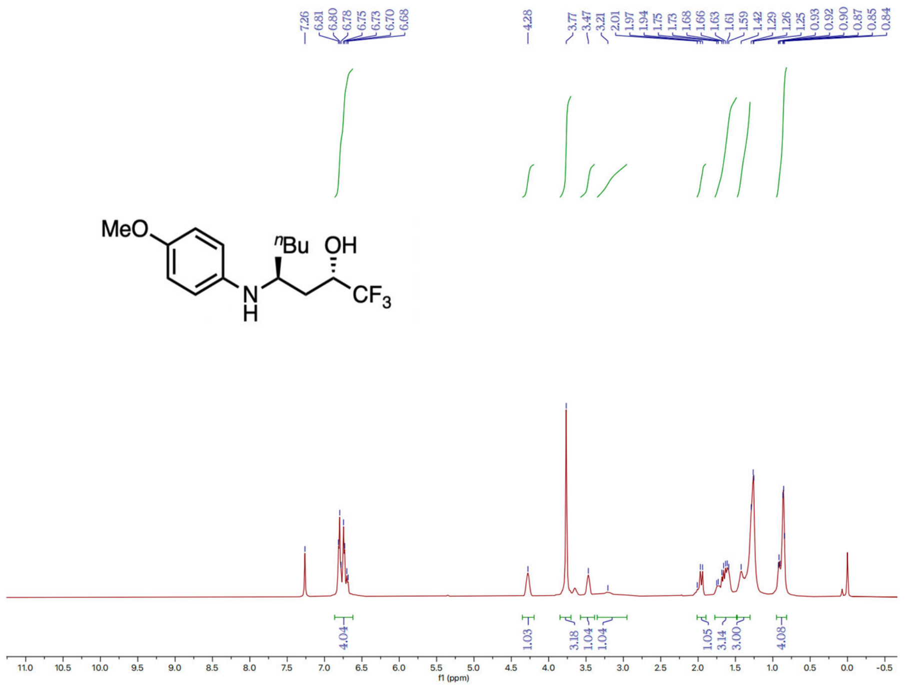

^{1}H NMR spectrum (500 MHz, Chloroform-*d*) of compound **(±)-84**

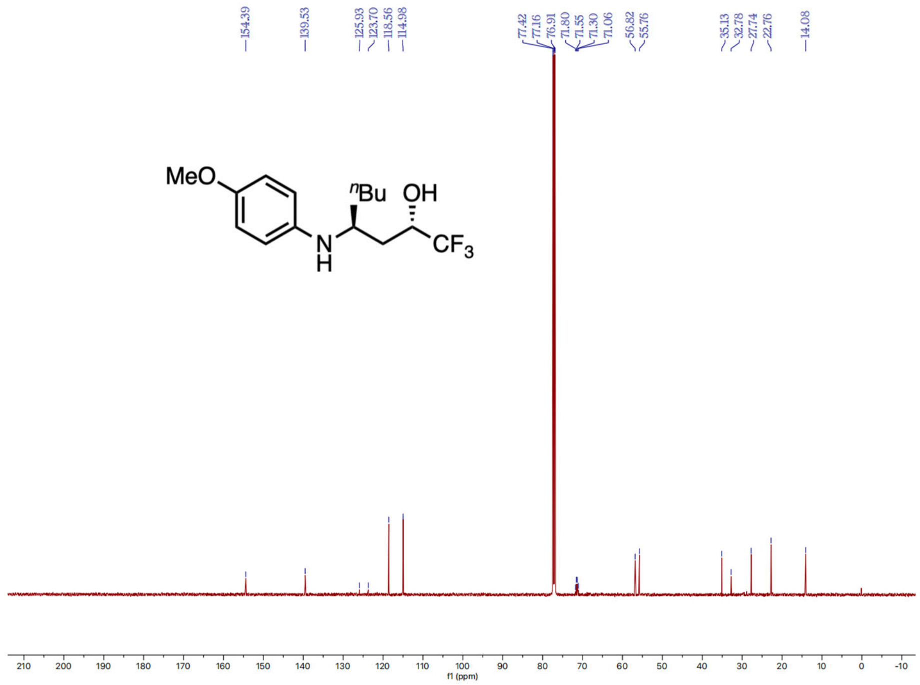

^{13}C NMR spectrum (126 MHz, Chloroform-*d*) of compound **(±)-84**

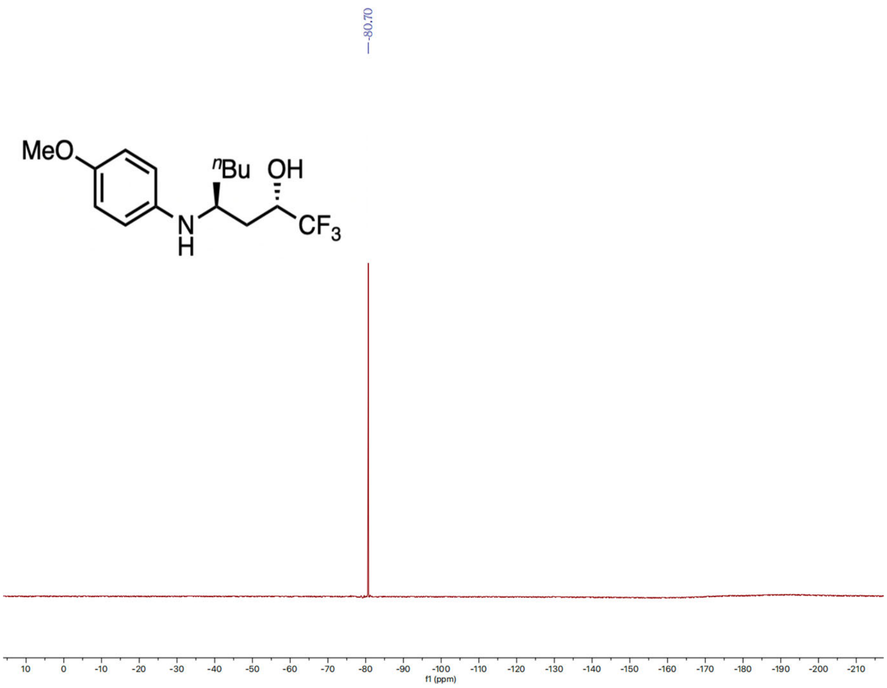

^{19}F NMR spectrum (471 MHz, Chloroform-*d*) of compound **(±)-84**

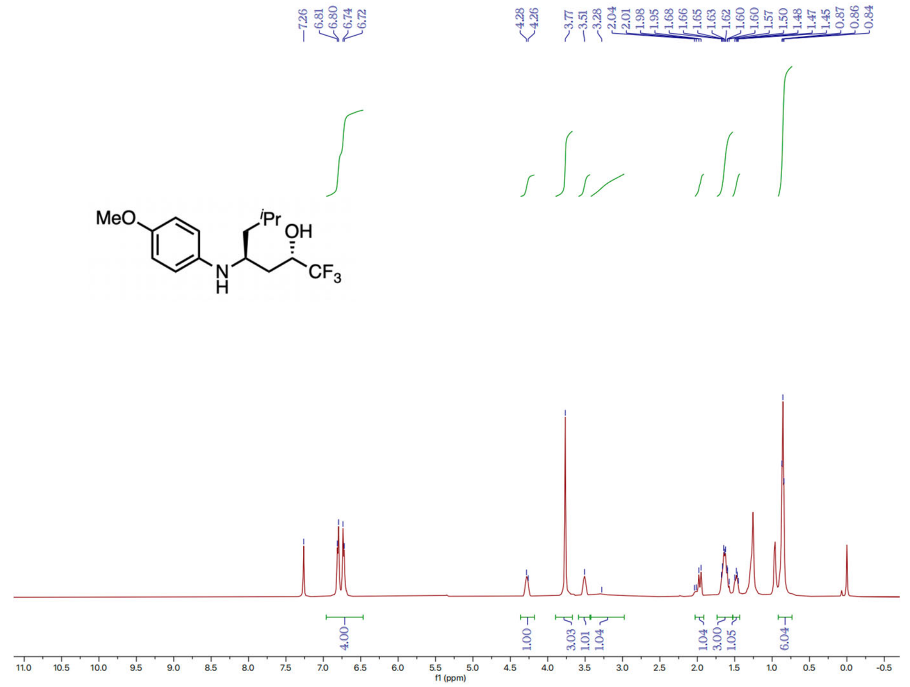

^{1}H NMR spectrum (500 MHz, Chloroform-*d*) of compound **(±)-85**

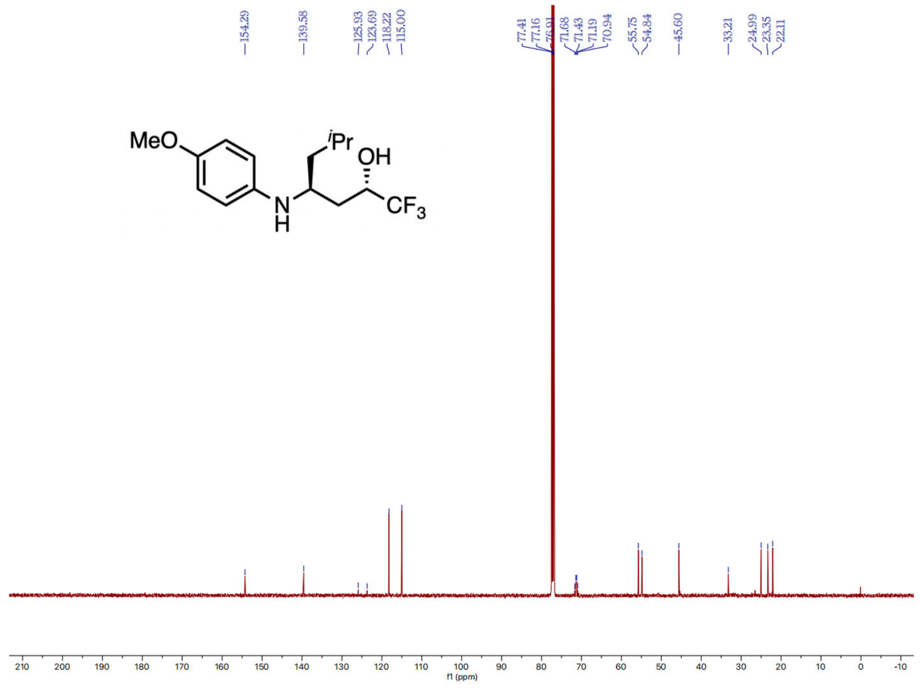

^{13}C NMR spectrum (126 MHz, Chloroform-*d*) of compound **(±)-85**

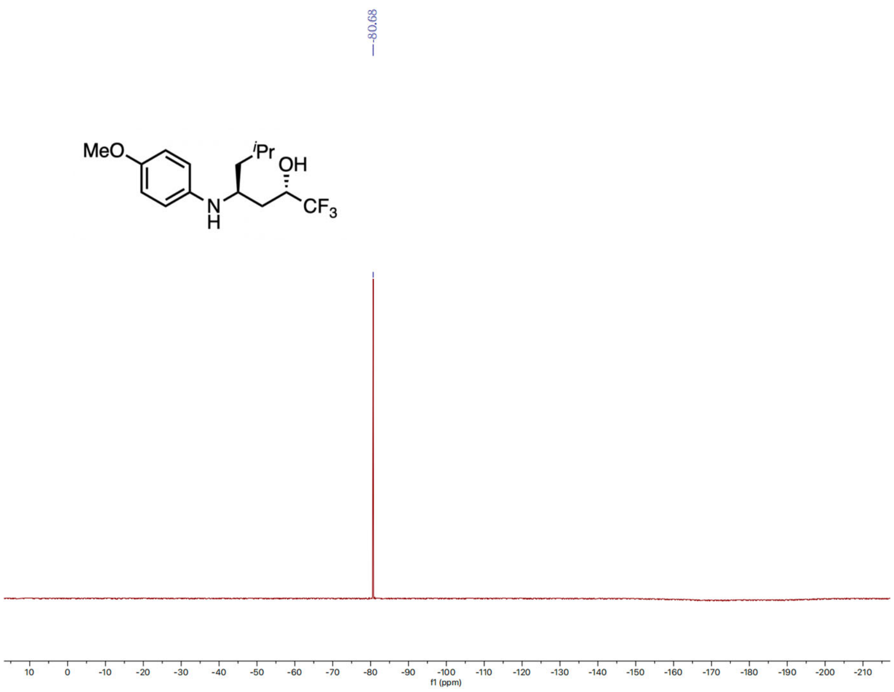

^{19}F NMR spectrum (471 MHz, Chloroform-*d*) of compound **(±)-85**

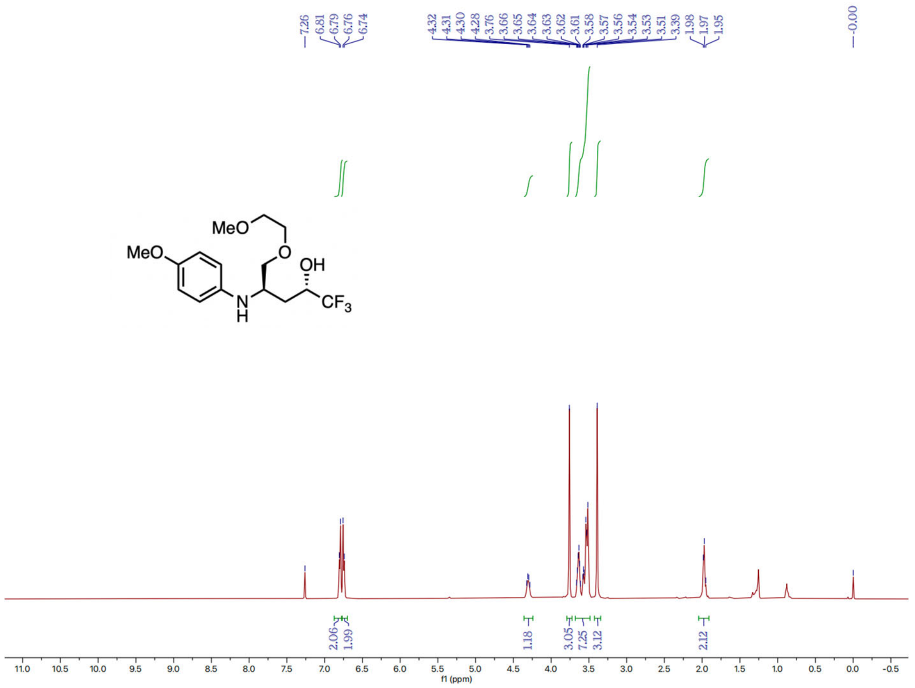

^{1}H NMR spectrum (500 MHz, Chloroform-*d*) of compound **(±)-86**

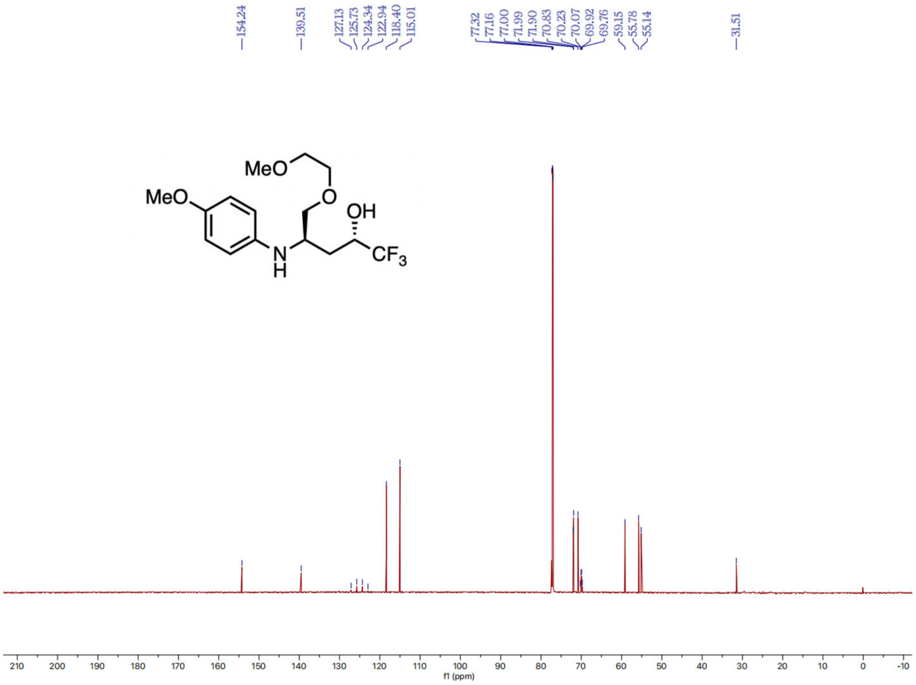

^{13}C NMR spectrum (201 MHz, Chloroform-*d*) of compound **(±)-86**

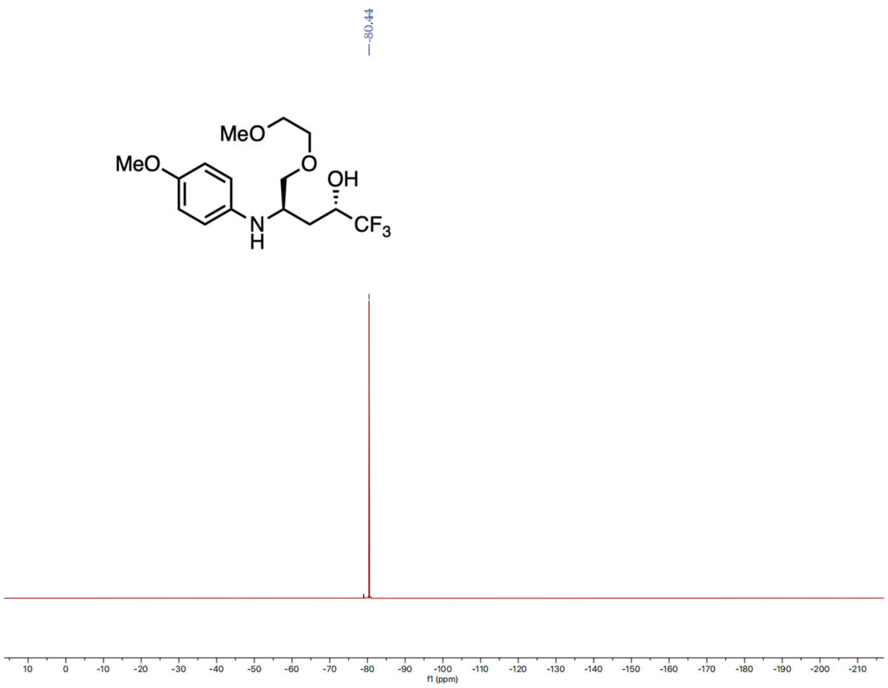

^{19}F NMR spectrum (471 MHz, Chloroform-*d*) of compound **(±)-86**

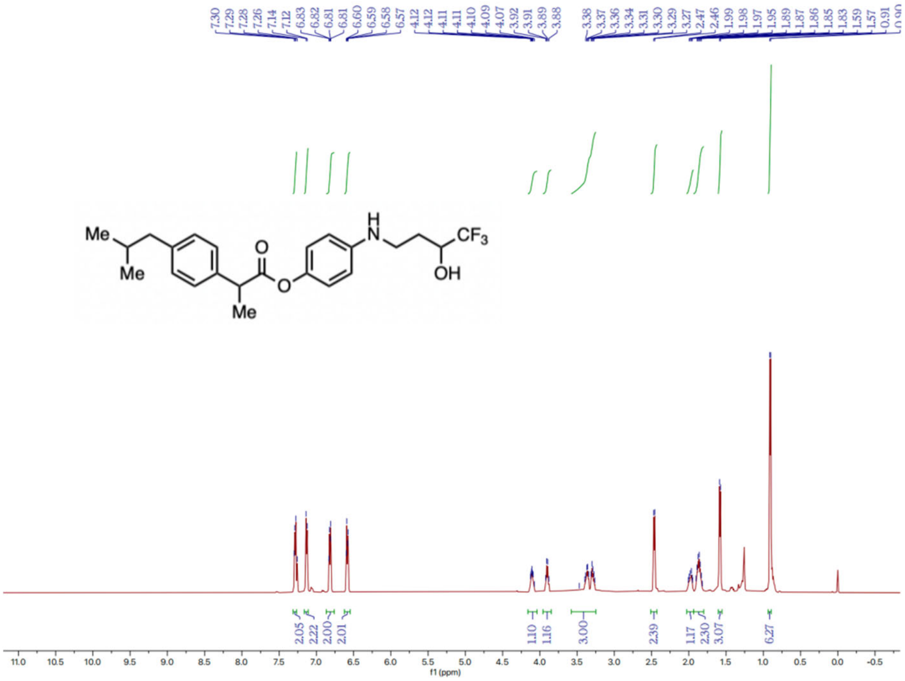

^{1}H NMR spectrum (500 MHz, Chloroform-*d*) of compound **87**

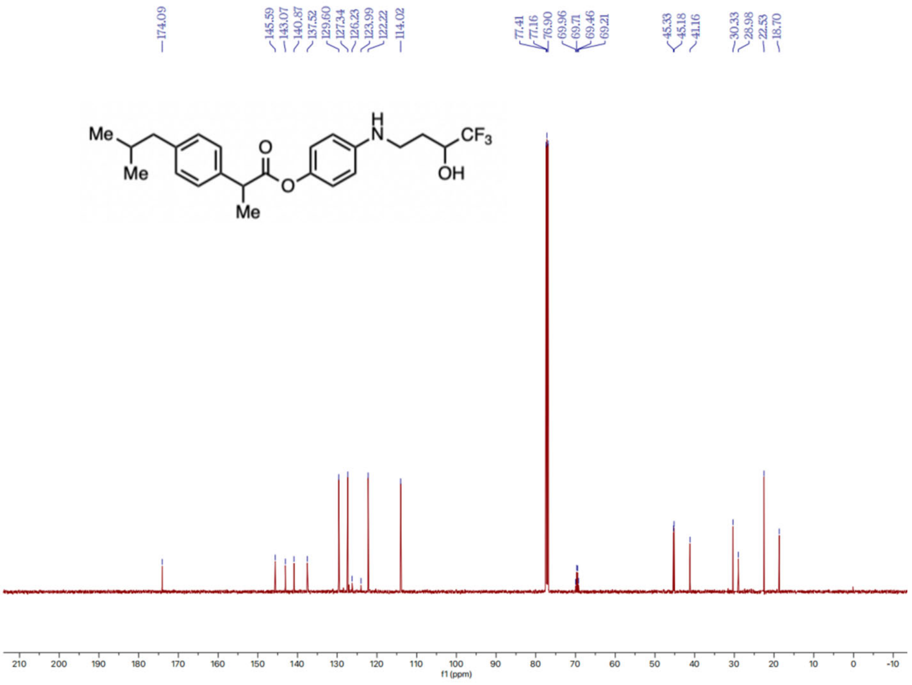

^{13}C NMR spectrum (126 MHz, Chloroform-*d*) of compound **87**

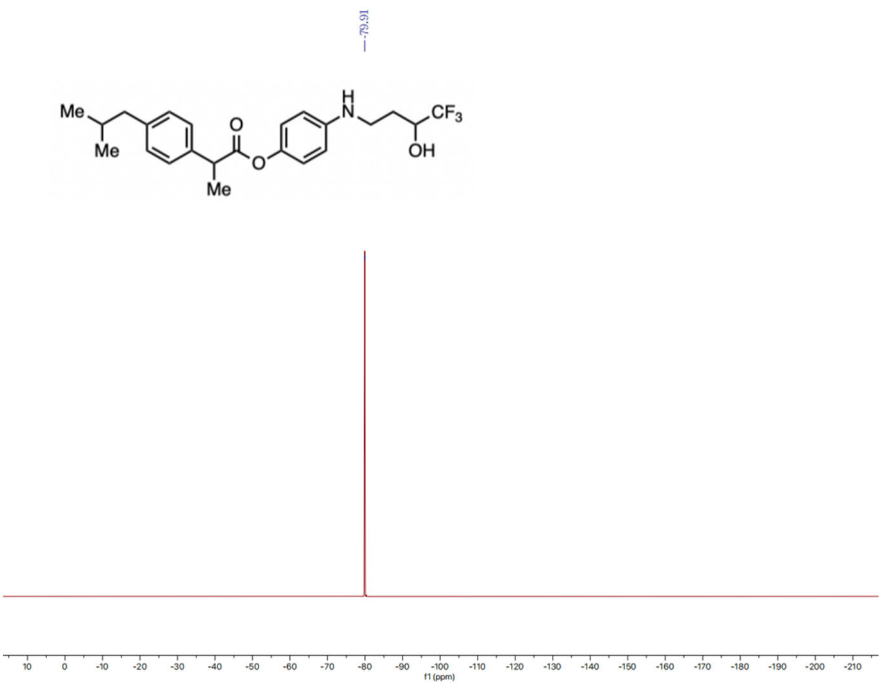

^{19}F NMR spectrum (471 MHz, Chloroform-*d*) of compound **87**

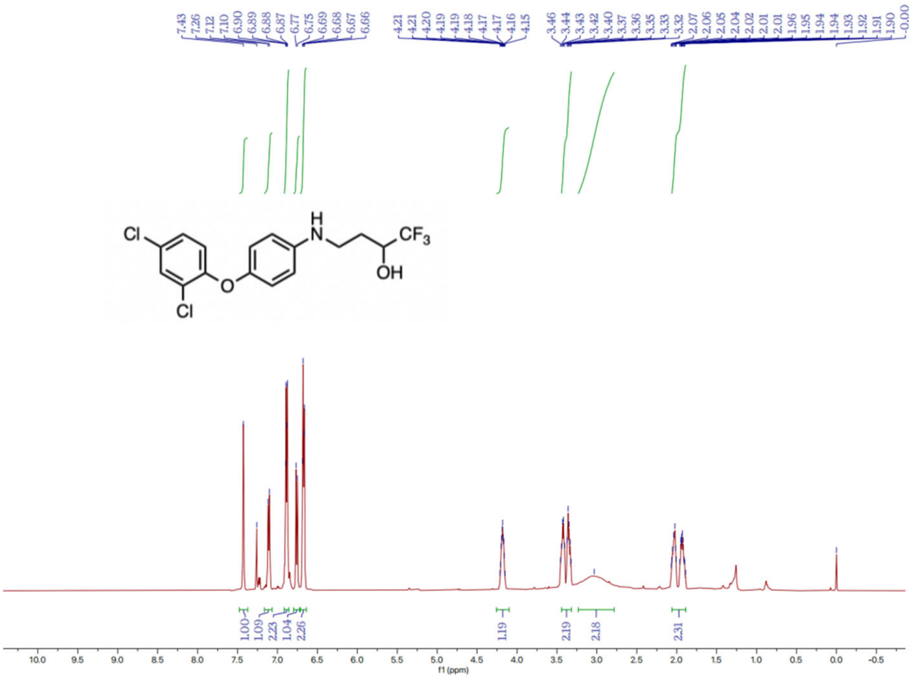

^{1}H NMR spectrum (500 MHz, Chloroform-*d*) of compound **88**

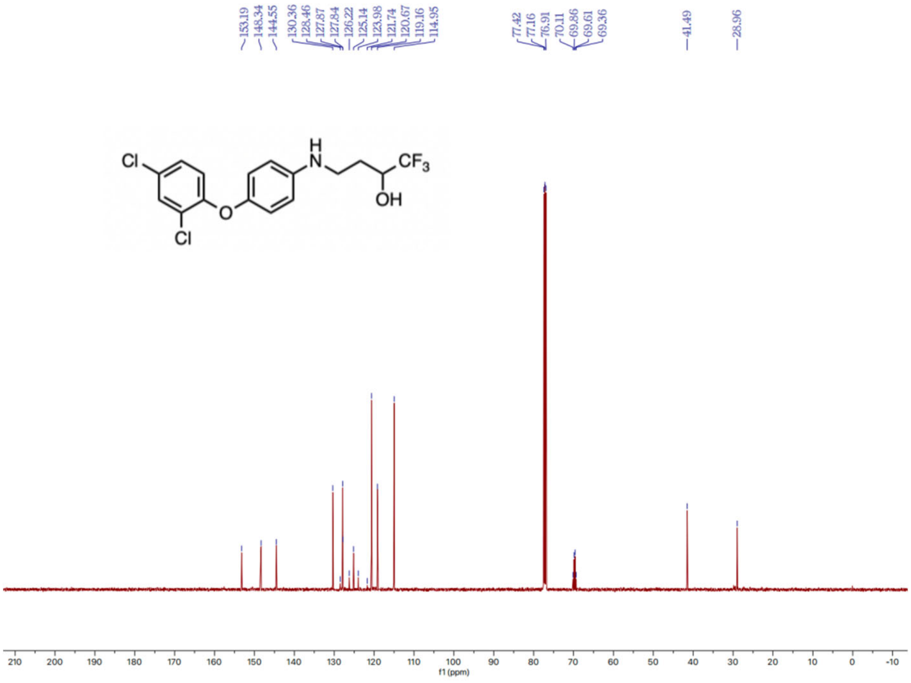

^{13}C NMR spectrum (126 MHz, Chloroform-*d*) of compound **88**

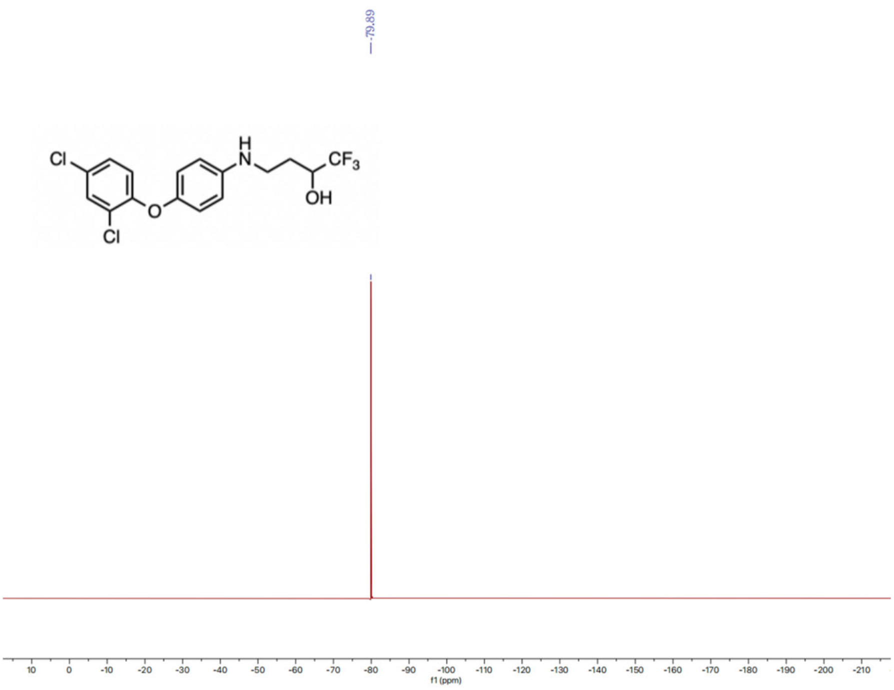

^{19}F NMR spectrum (471 MHz, Chloroform-*d*) of compound **88**

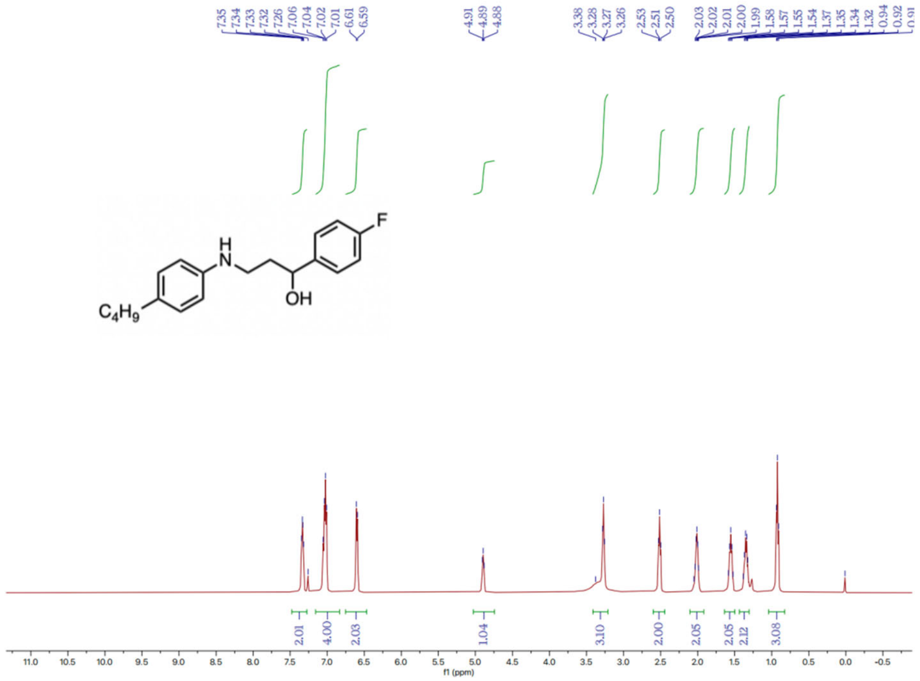

^{1}H NMR spectrum (500 MHz, Chloroform-*d*) of compound **89**

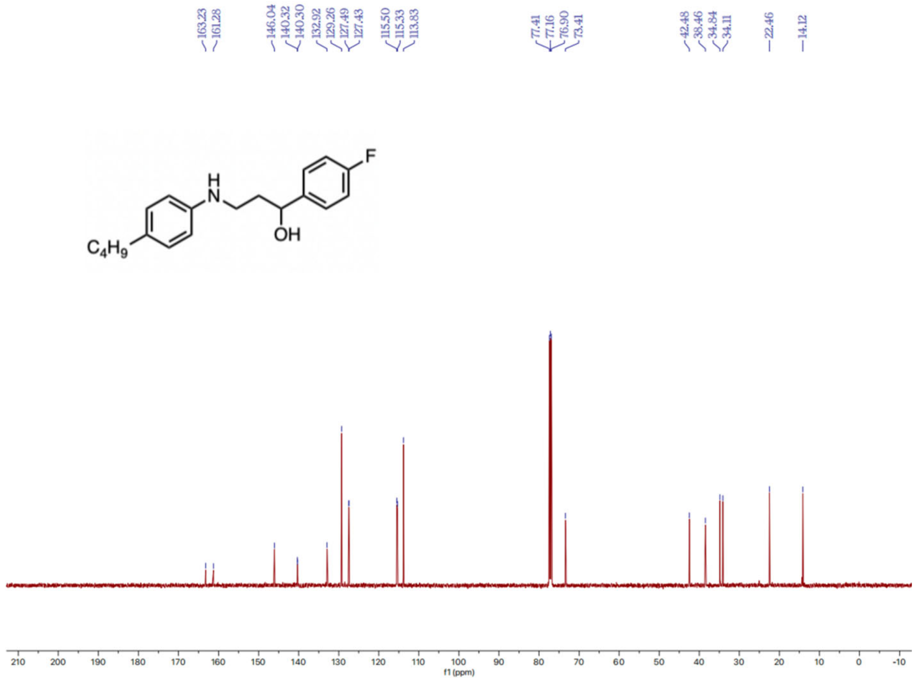

^{13}C NMR spectrum (126 MHz, Chloroform-*d*) of compound **89**

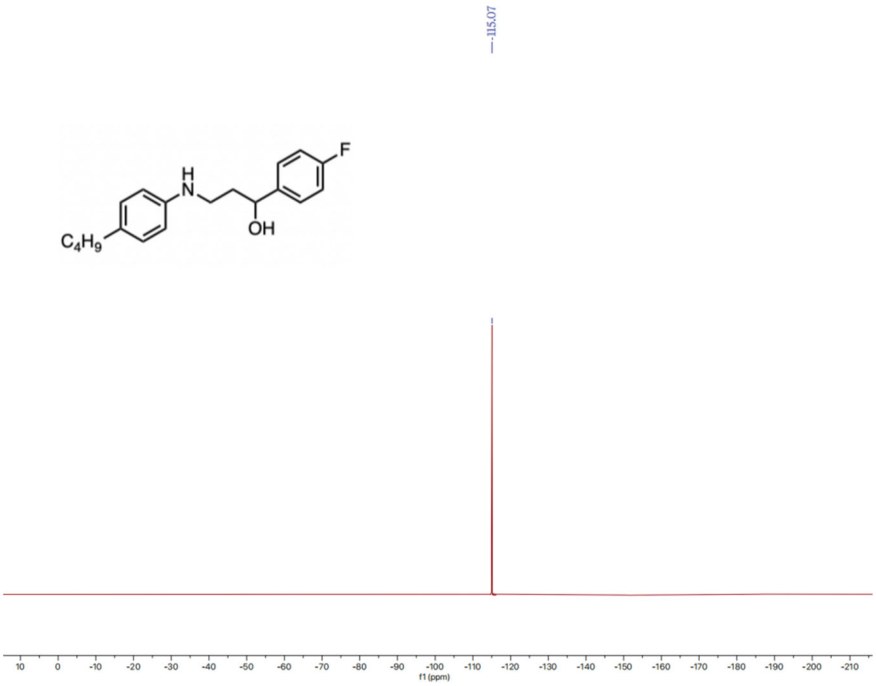

^{19}F NMR spectrum (471 MHz, Chloroform-*d*) of compound **89**

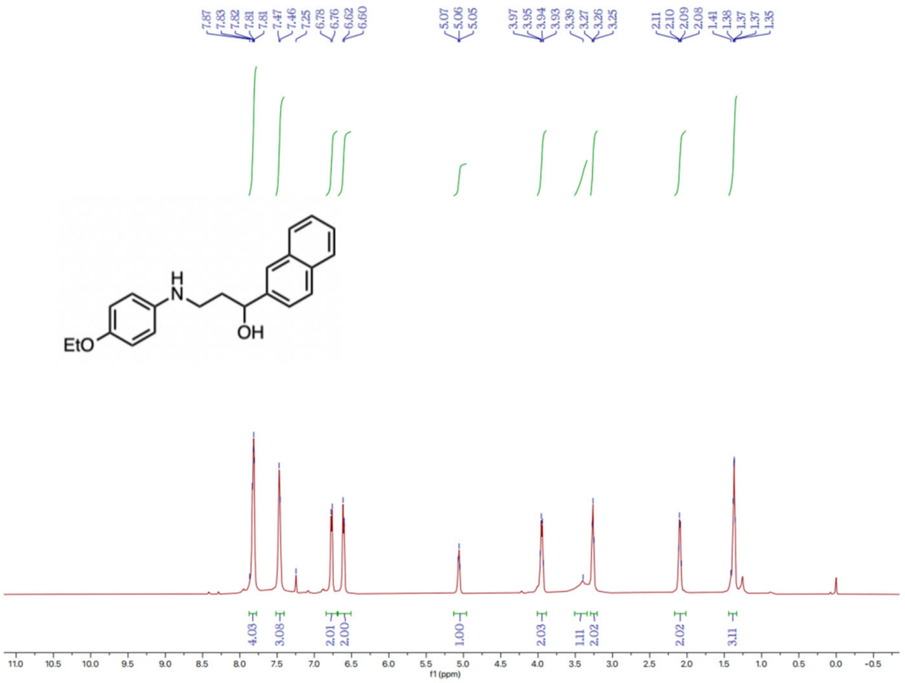

^{1}H NMR spectrum (500 MHz, Chloroform-*d*) of compound **90**

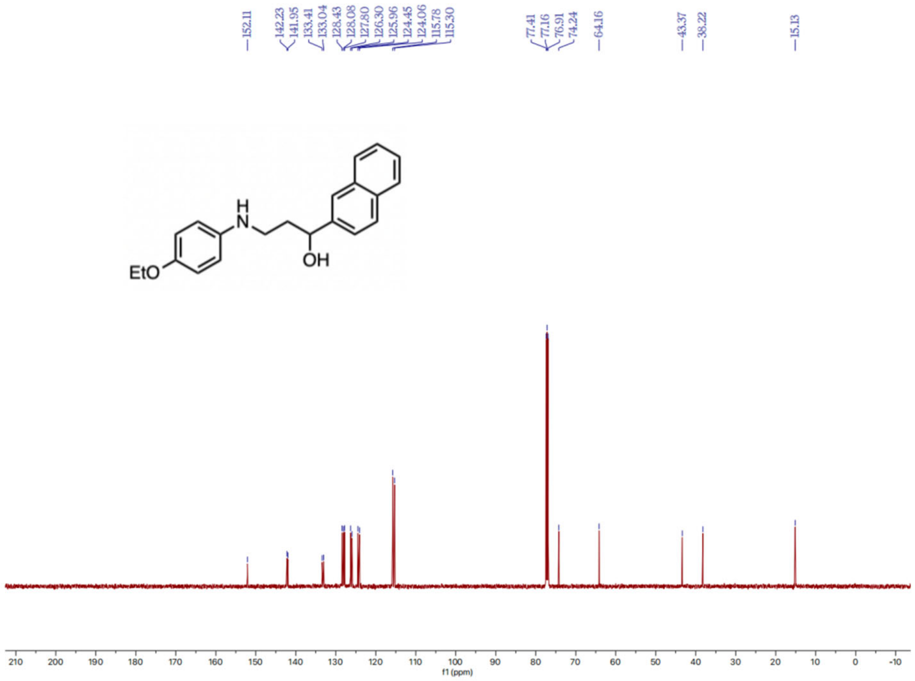

^{13}C NMR spectrum (126 MHz, Chloroform-*d*) of compound **90**

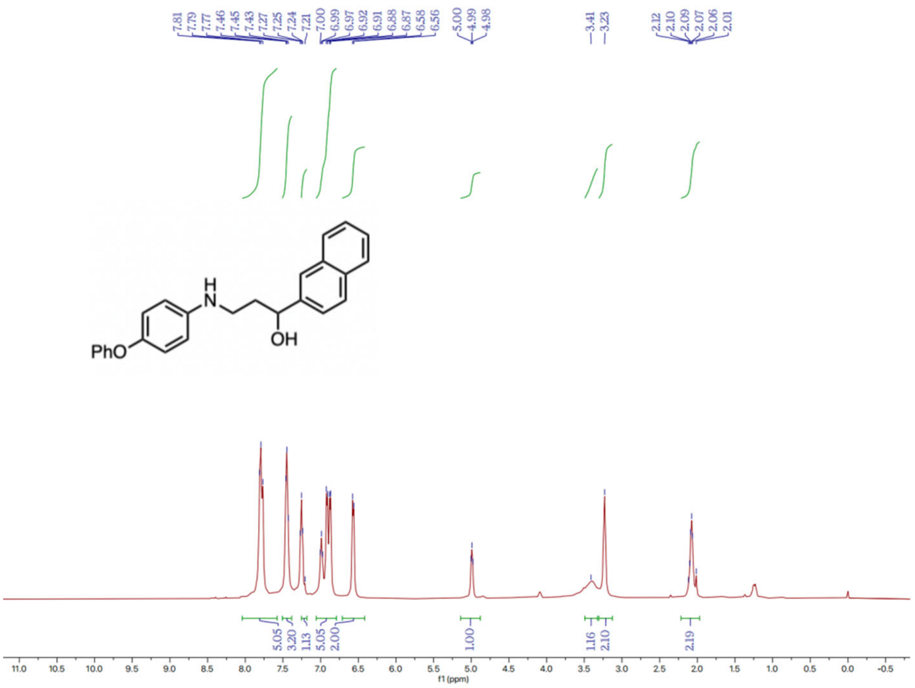

^{1}H NMR spectrum (500 MHz, Chloroform-*d*) of compound **91**

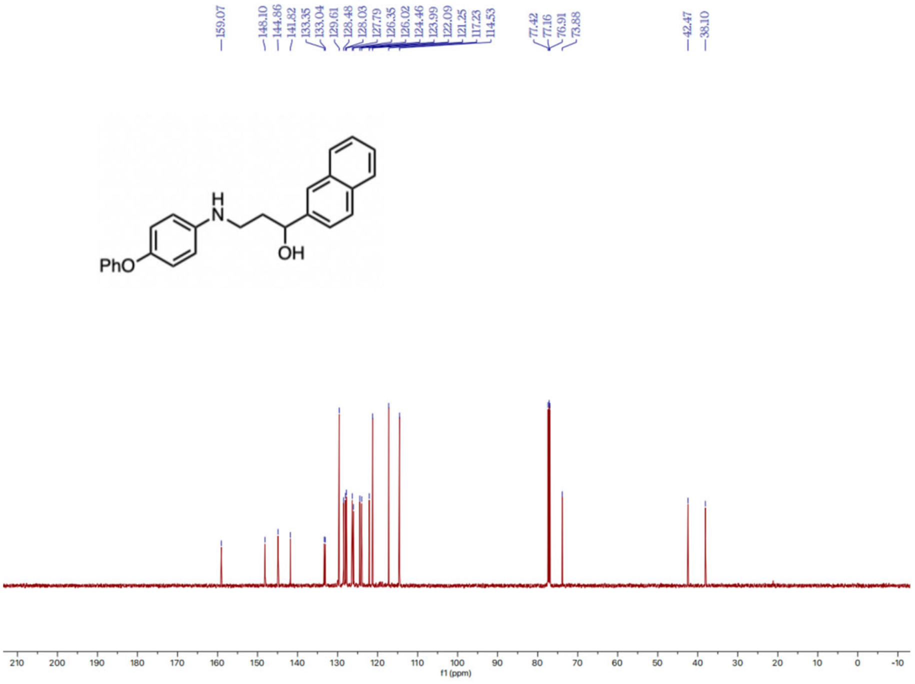

^{13}C NMR spectrum (126 MHz, Chloroform-*d*) of compound **91**

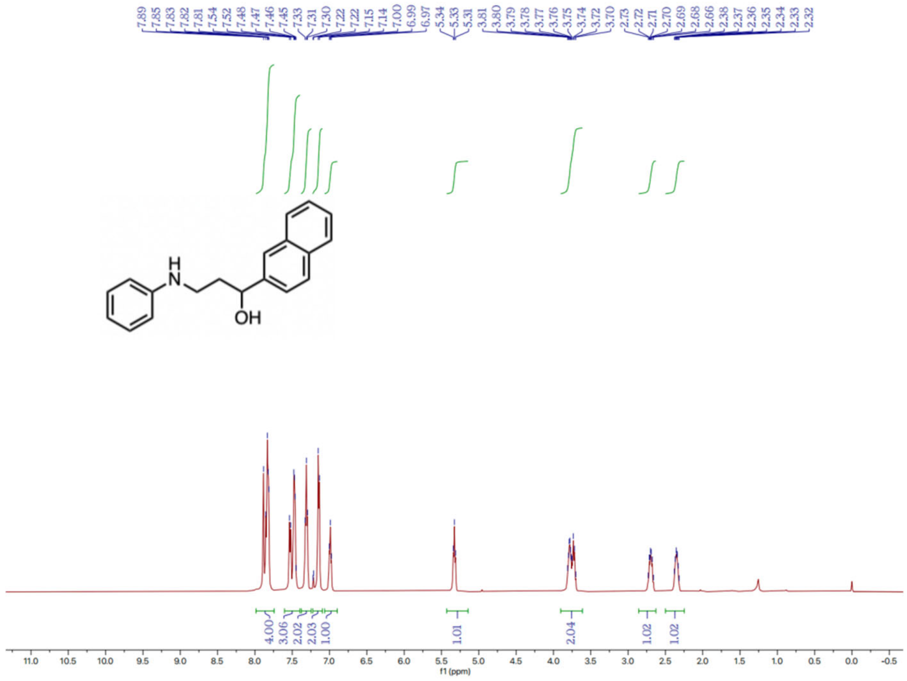

^{1}H NMR spectrum (500 MHz, Chloroform-*d*) of compound **92**

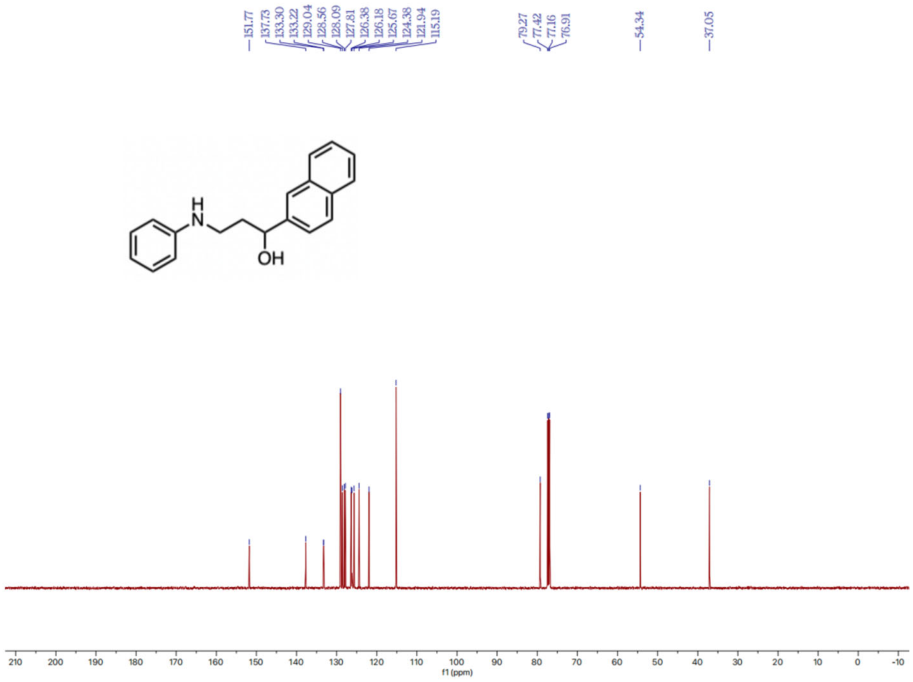

^{13}C NMR spectrum (126 MHz, Chloroform-*d*) of compound **92**

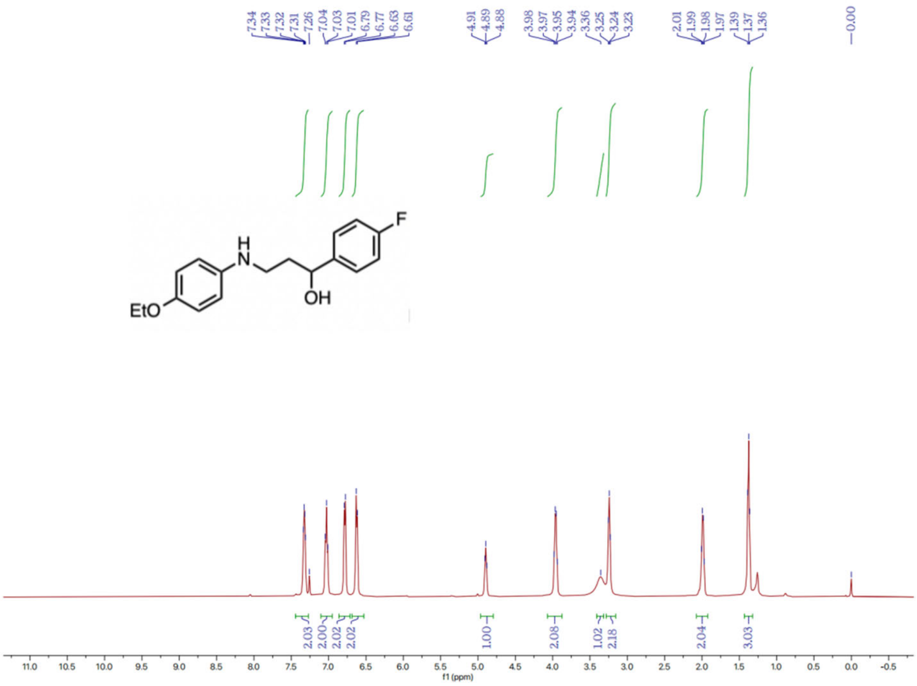

^{1}H NMR spectrum (500 MHz, Chloroform-*d*) of compound **93**

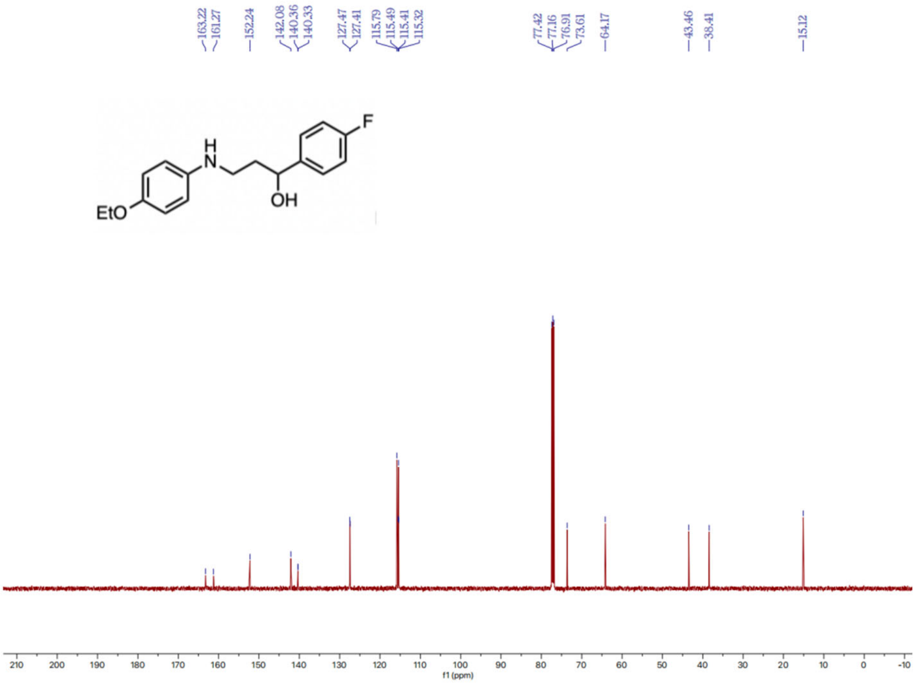

^{13}C NMR spectrum (126 MHz, Chloroform-*d*) of compound **93**

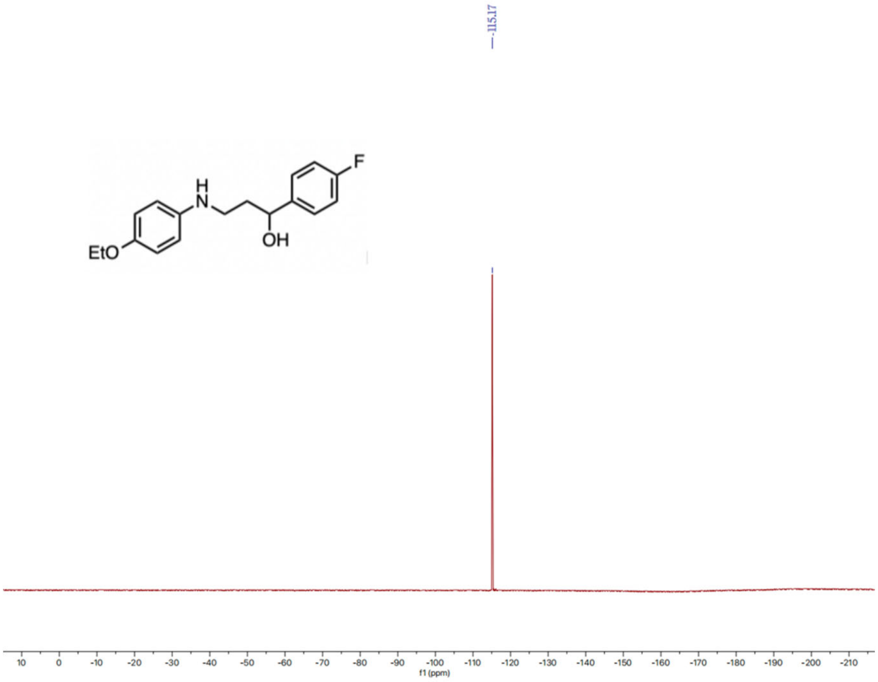

^{19}F NMR spectrum (471 MHz, Chloroform-*d*) of compound **93**

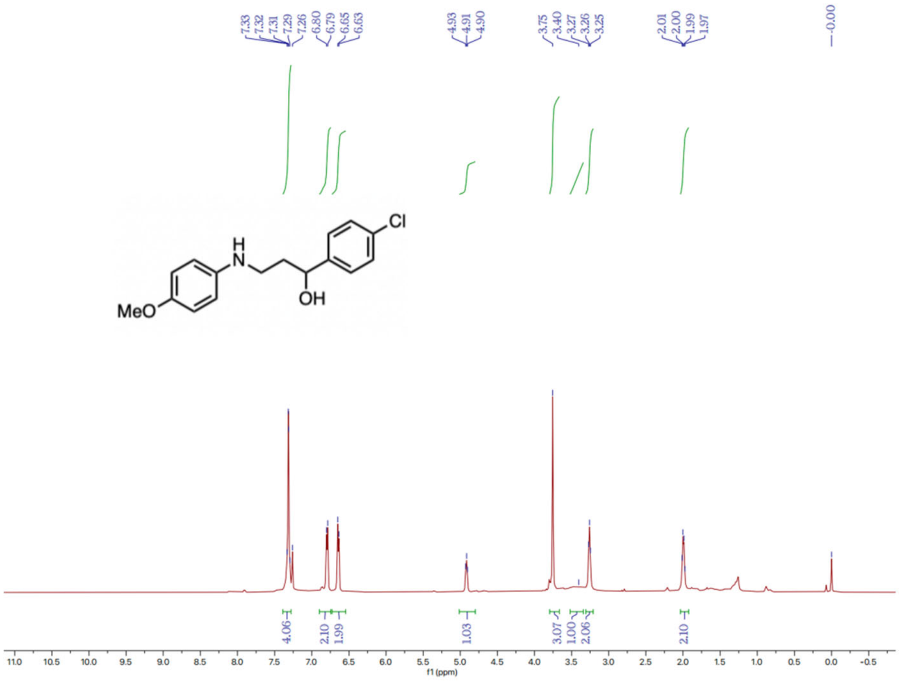

^{1}H NMR spectrum (500 MHz, Chloroform-*d*) of compound **94**

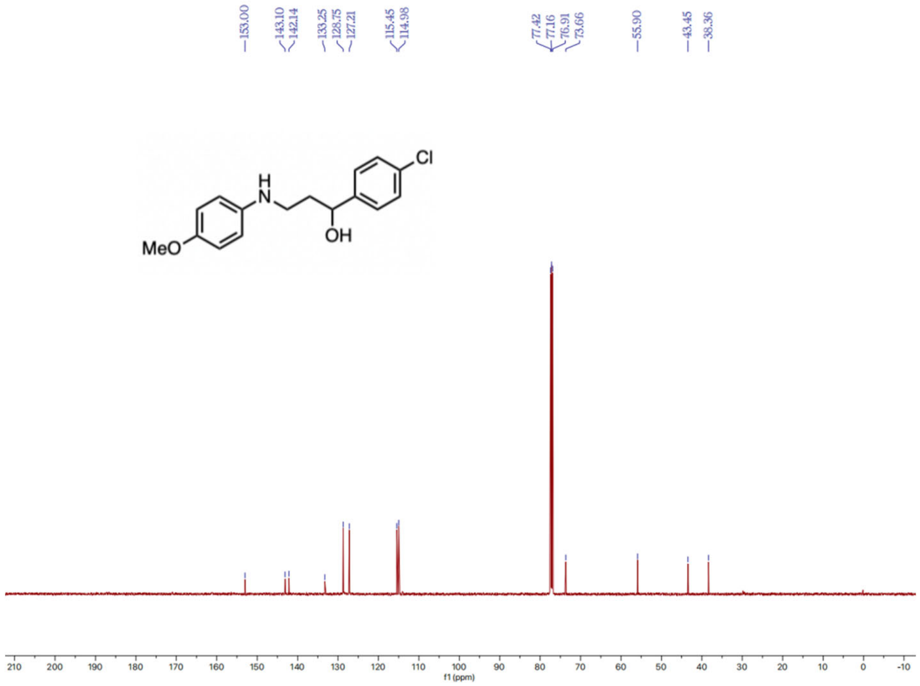

^{13}C NMR spectrum (126 MHz, Chloroform-*d*) of compound **94**

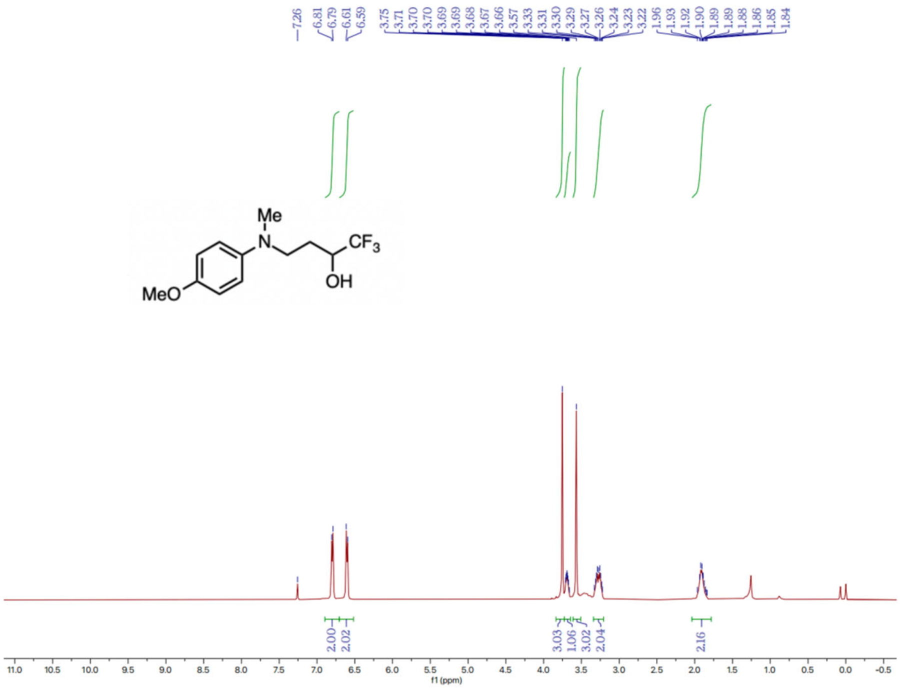

^{1}H NMR spectrum (500 MHz, Chloroform-*d*) of compound **95**

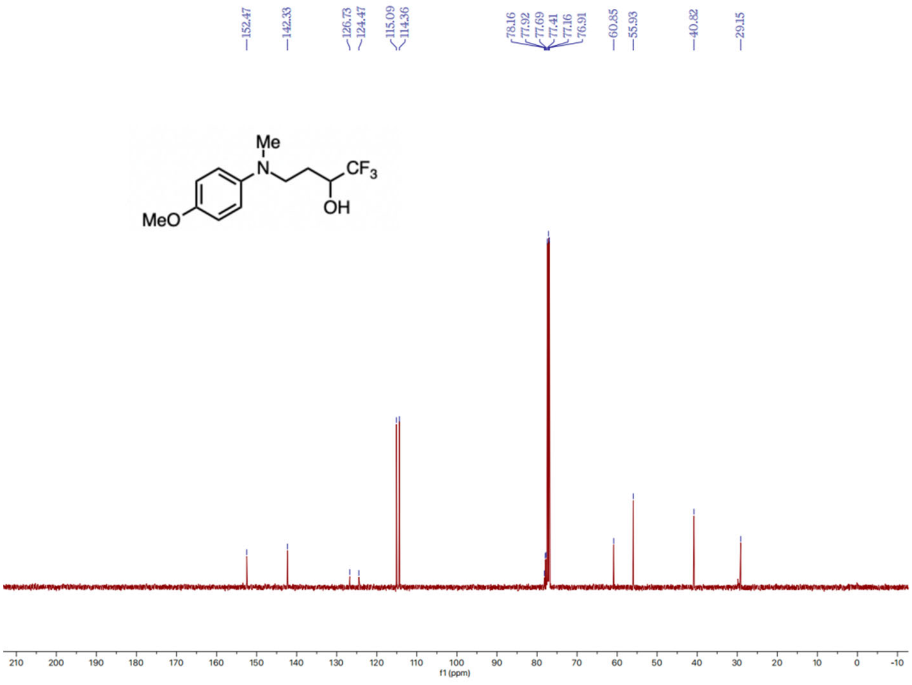

^{13}C NMR spectrum (126 MHz, Chloroform-*d*) of compound **95**

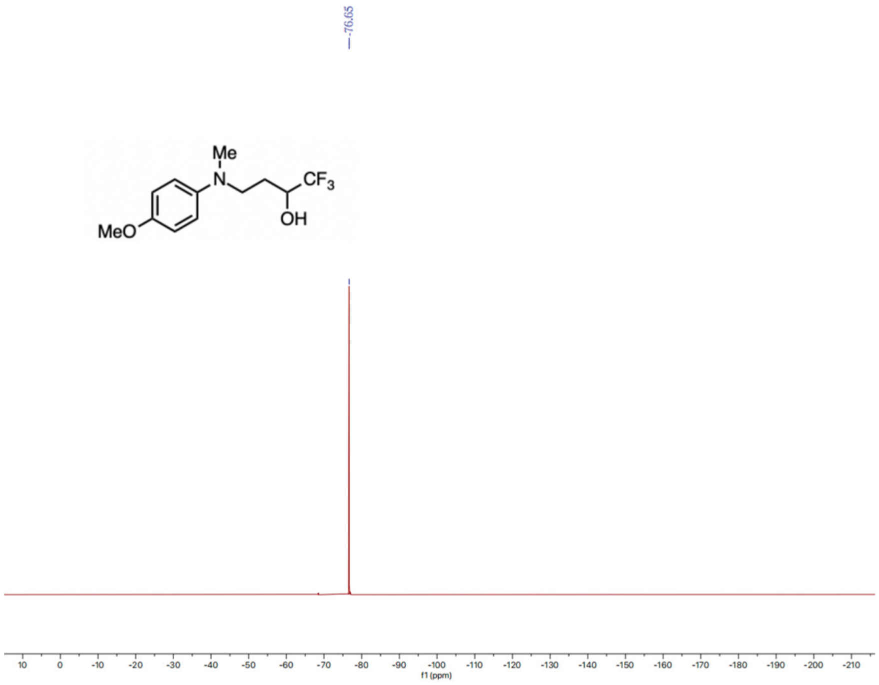

^{19}F NMR spectrum (471 MHz, Chloroform-*d*) of compound **95**

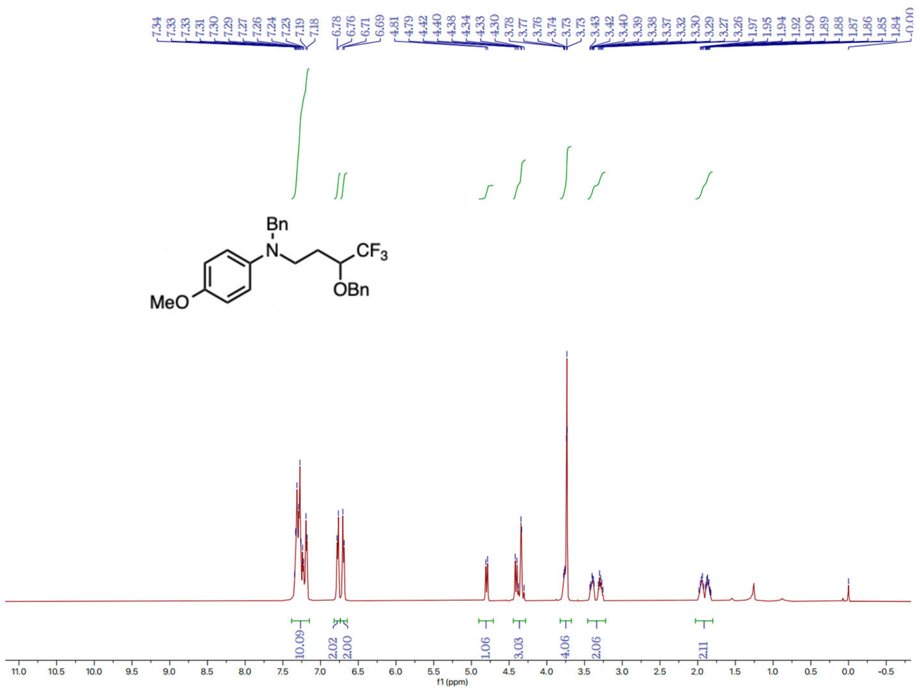

^1H NMR spectrum (500 MHz, Chloroform-*d*) of compound **96**

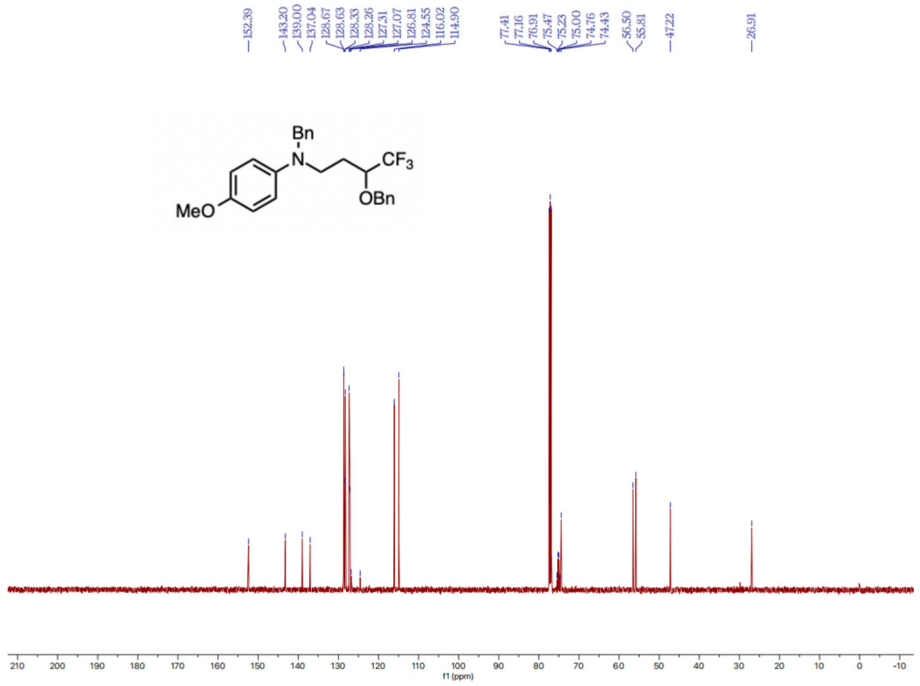

^{13}C NMR spectrum (126 MHz, Chloroform-*d*) of compound **96**

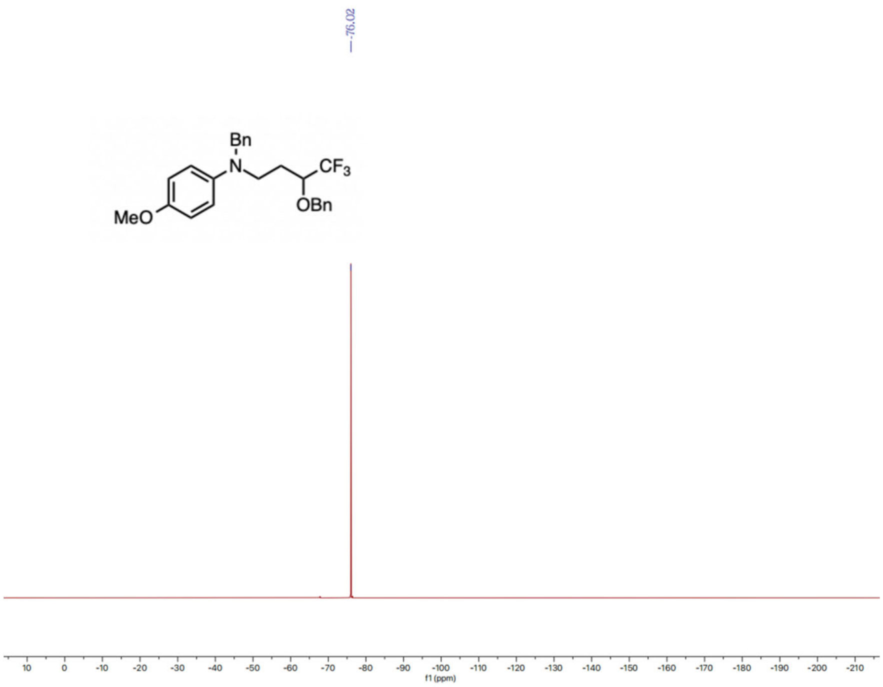

^{19}F NMR spectrum (471 MHz, Chloroform-*d*) of compound **96**

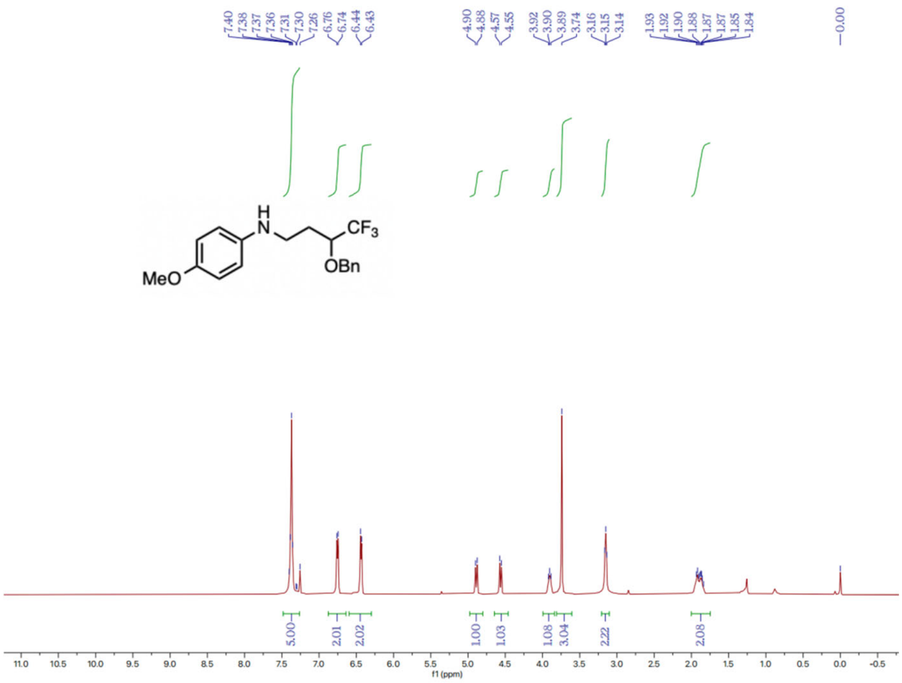

^{1}H NMR spectrum (500 MHz, Chloroform-*d*) of compound **97**

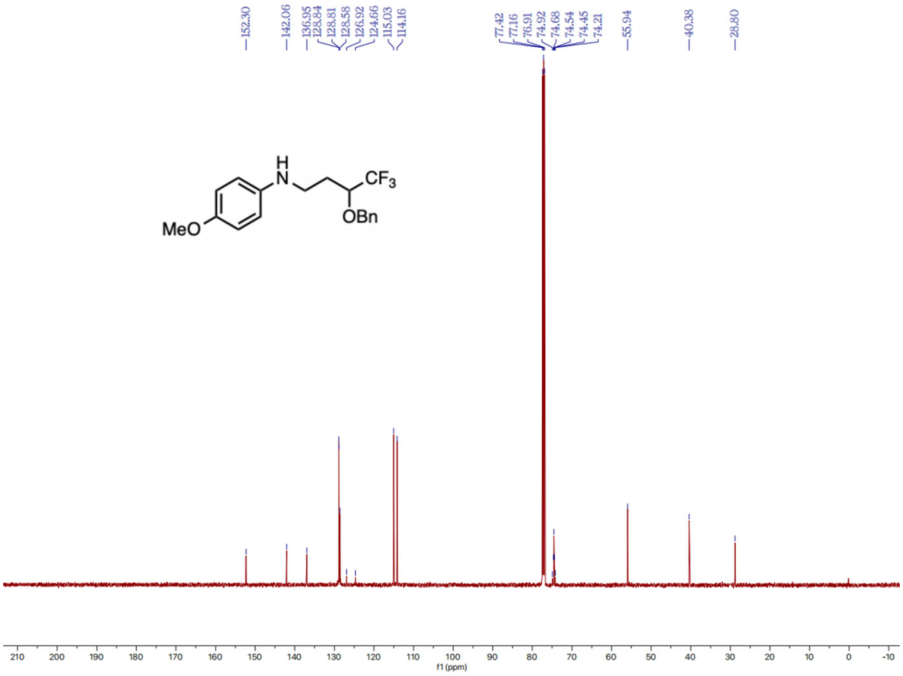

^{13}C NMR spectrum (126 MHz, Chloroform-*d*) of compound **97**

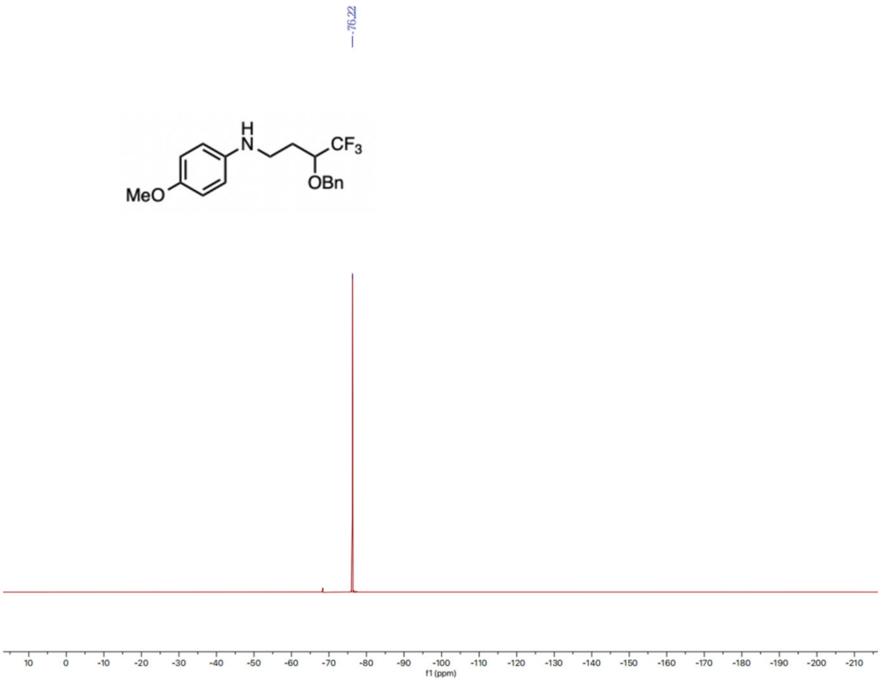

^{19}F NMR spectrum (471 MHz, Chloroform-*d*) of compound **97**

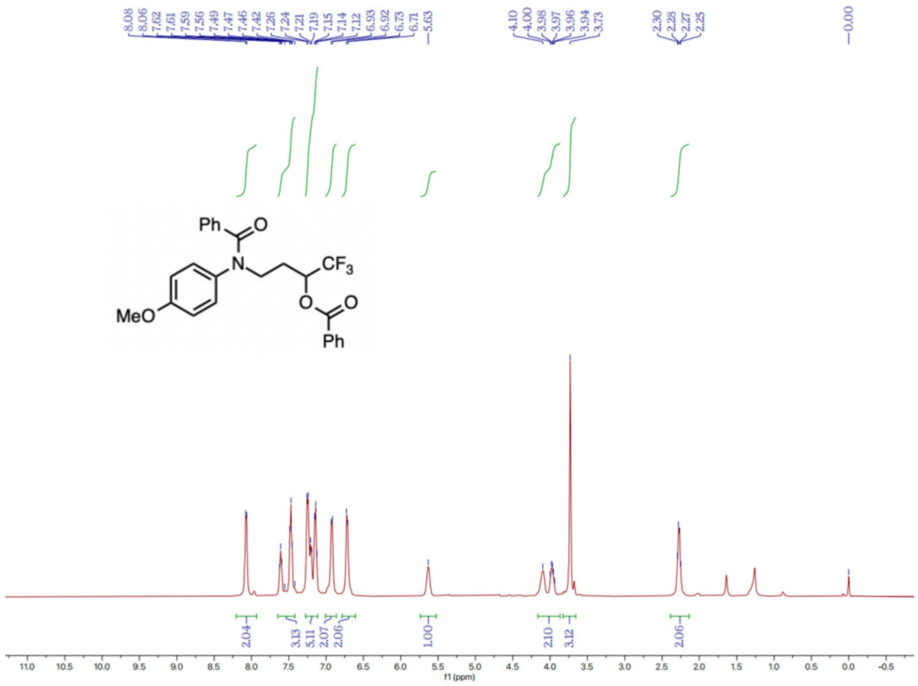

^{1}H NMR spectrum (500 MHz, Chloroform-*d*) of compound **98**

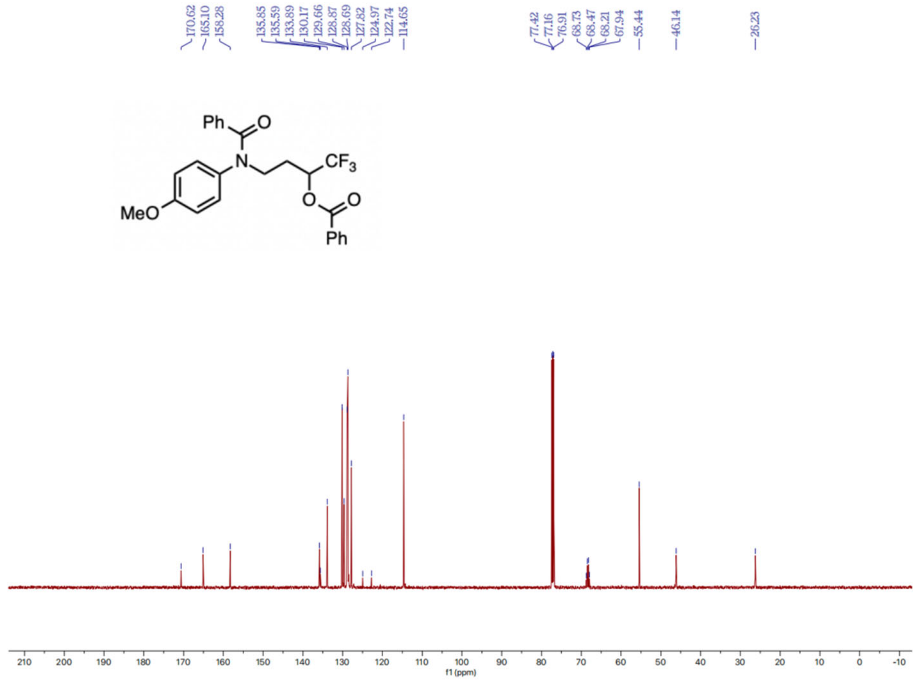

^{13}C NMR spectrum (126 MHz, Chloroform-*d*) of compound **98**

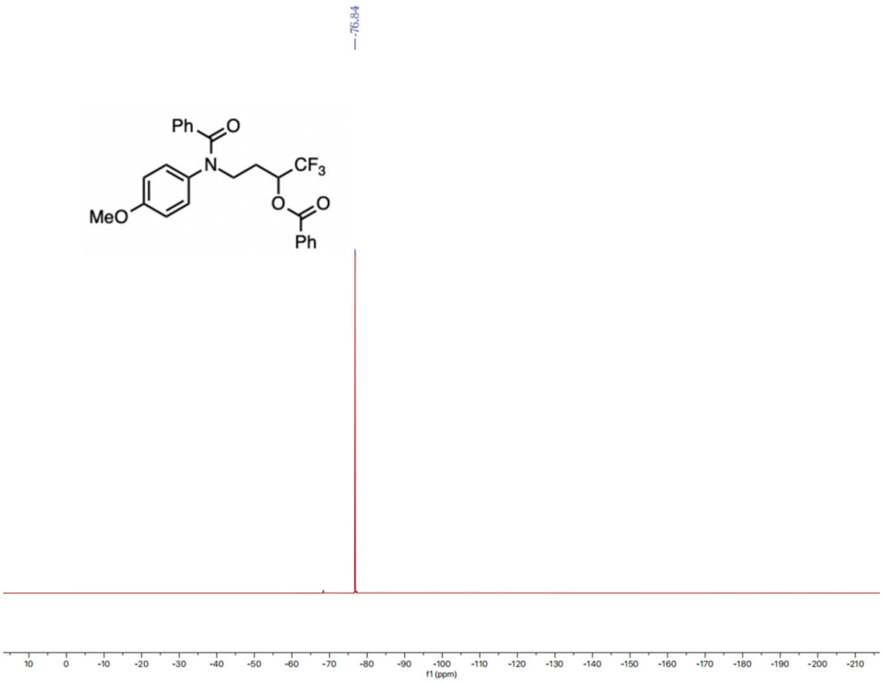

^{19}F NMR spectrum (471 MHz, Chloroform-*d*) of compound **98**

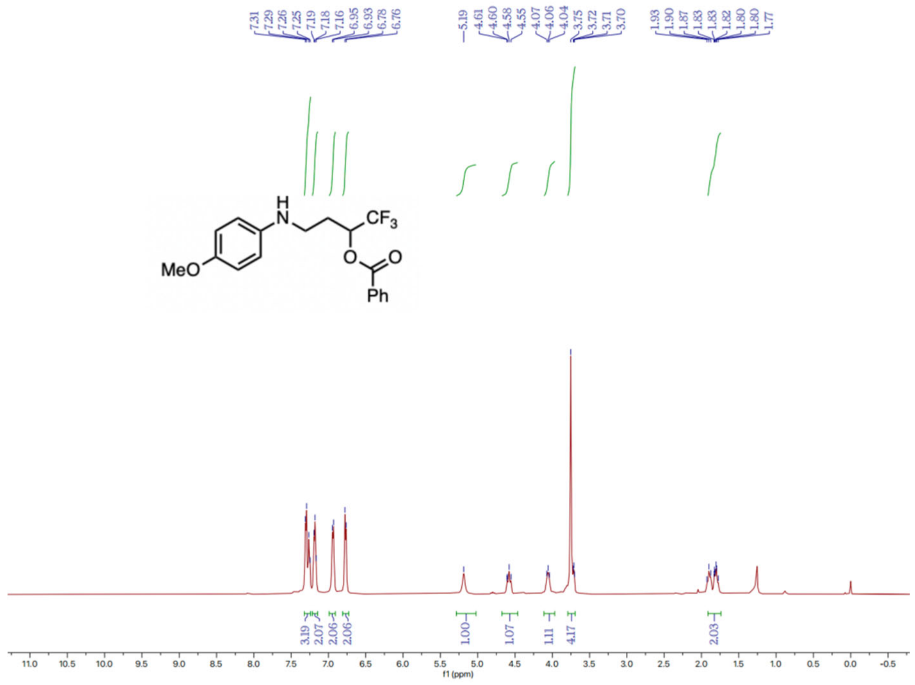

^{1}H NMR spectrum (500 MHz, Chloroform-*d*) of compound **99**

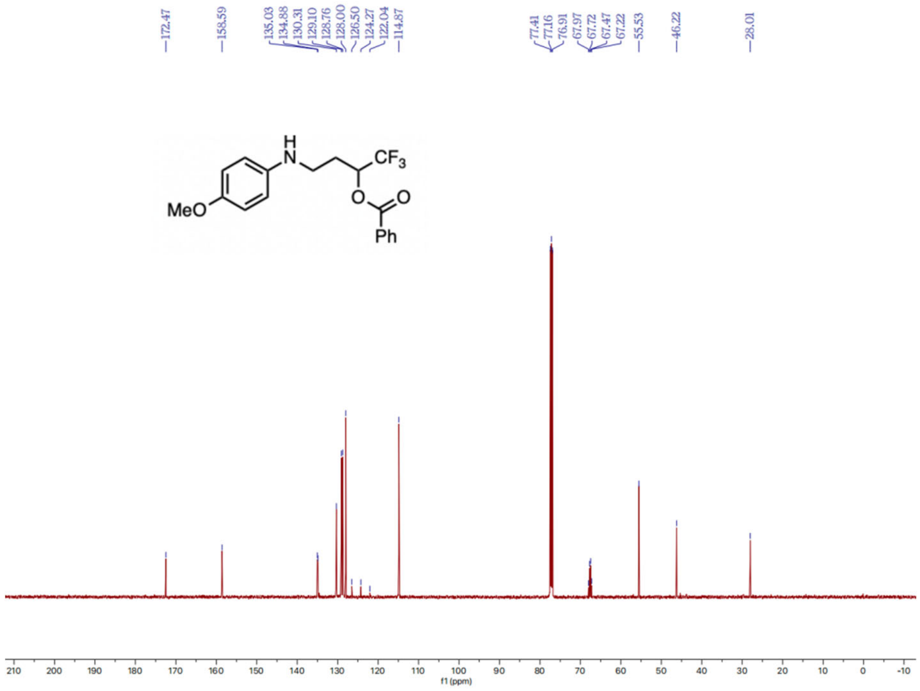

^{13}C NMR spectrum (126 MHz, Chloroform-*d*) of compound **99**

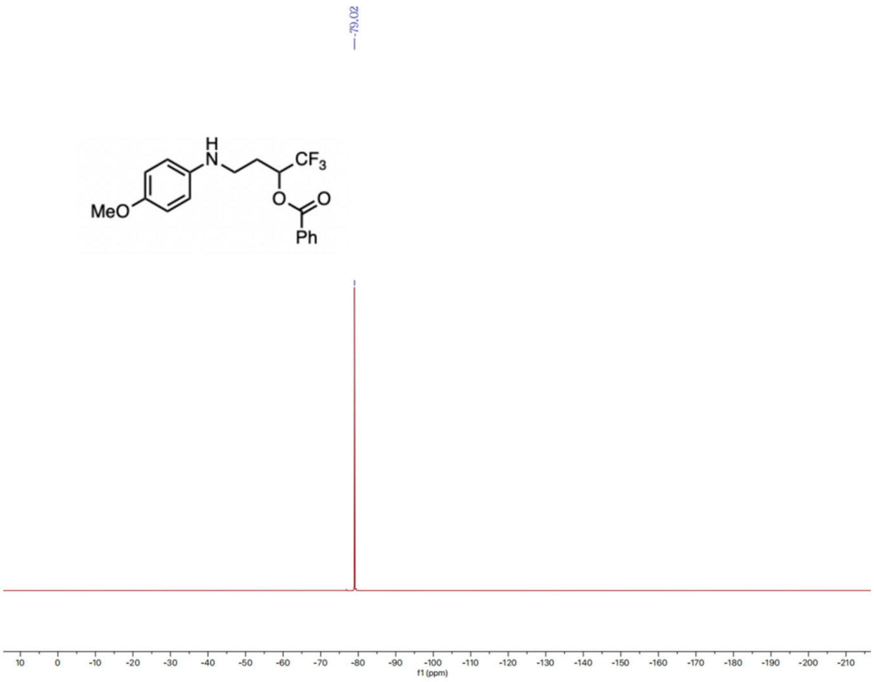

^{19}F NMR spectrum (471 MHz, Chloroform-*d*) of compound **99**

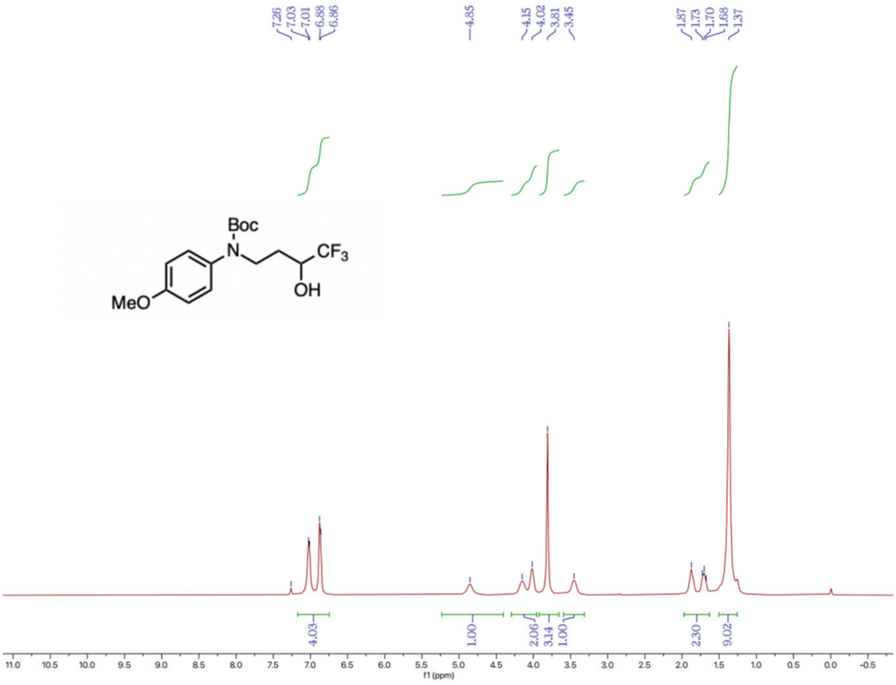

^{1}H NMR spectrum (500 MHz, Chloroform-*d*) of compound **100**

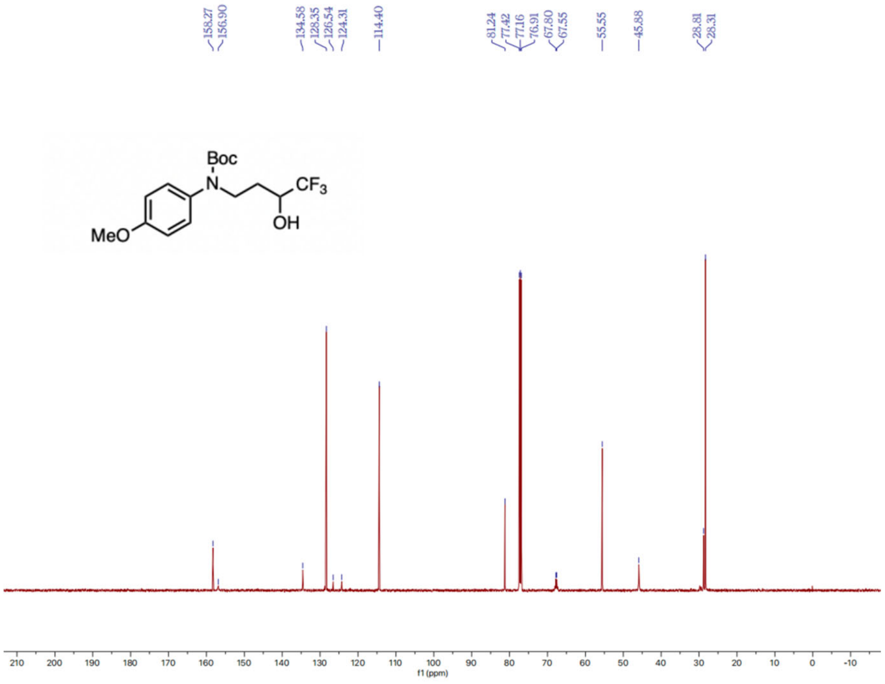

^{13}C NMR spectrum (126 MHz, Chloroform-*d*) of compound **100**

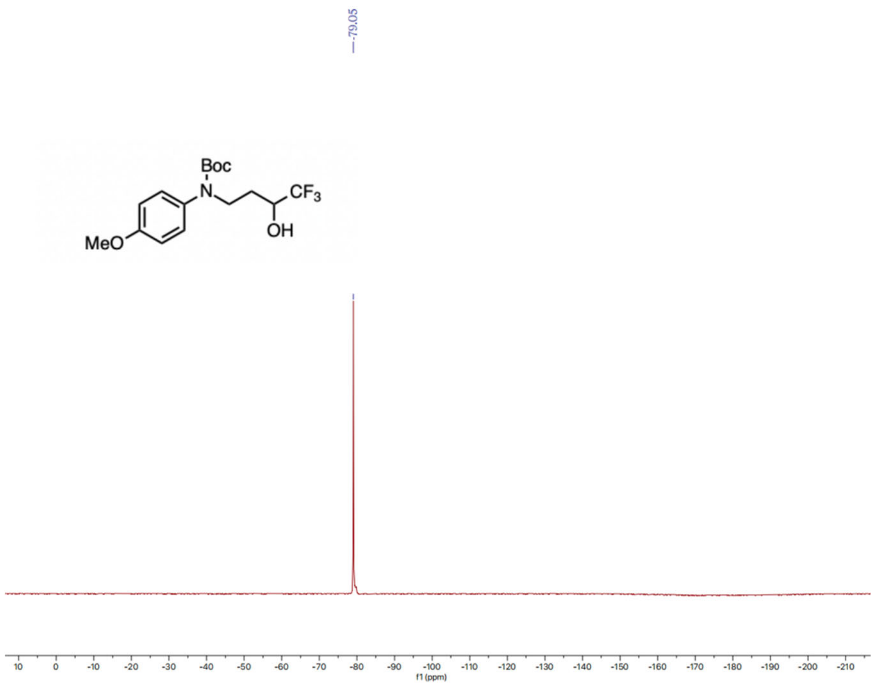

^{19}F NMR spectrum (471 MHz, Chloroform-*d*) of compound **100**

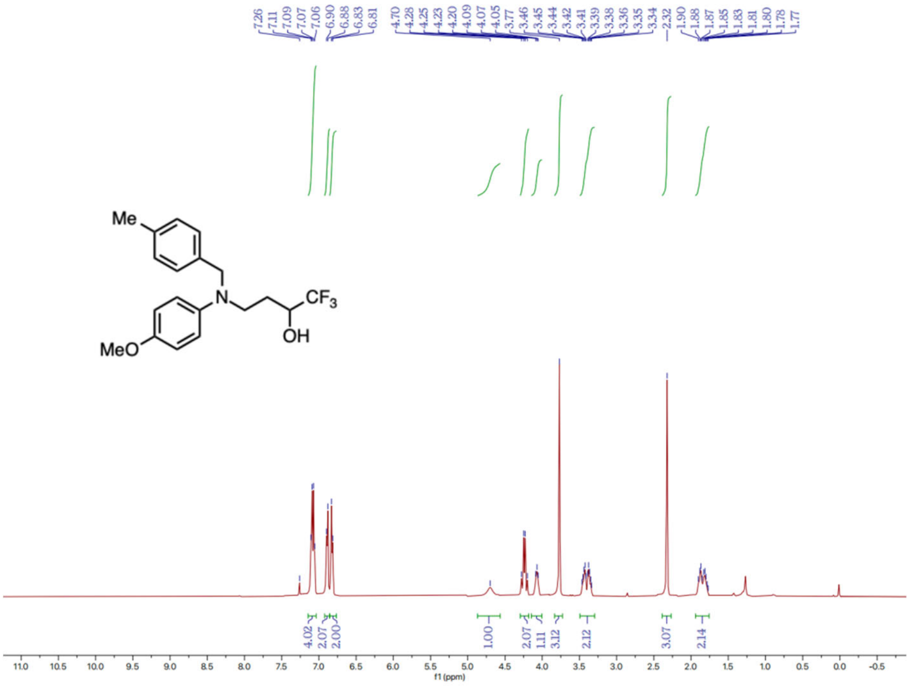

^{1}H NMR spectrum (500 MHz, Chloroform-*d*) of compound **101**

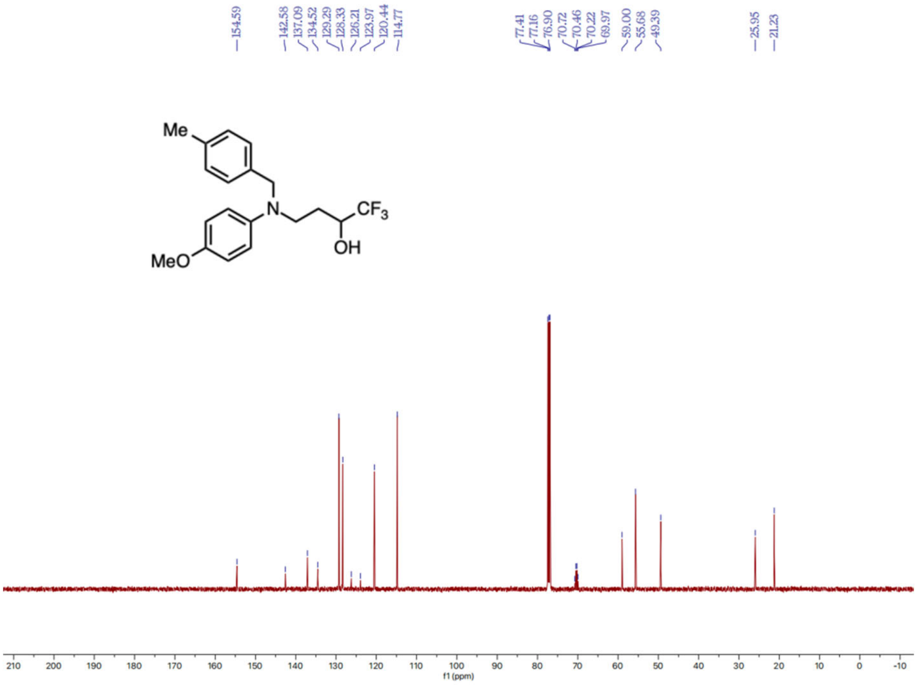

^{13}C NMR spectrum (126 MHz, Chloroform-*d*) of compound **101**

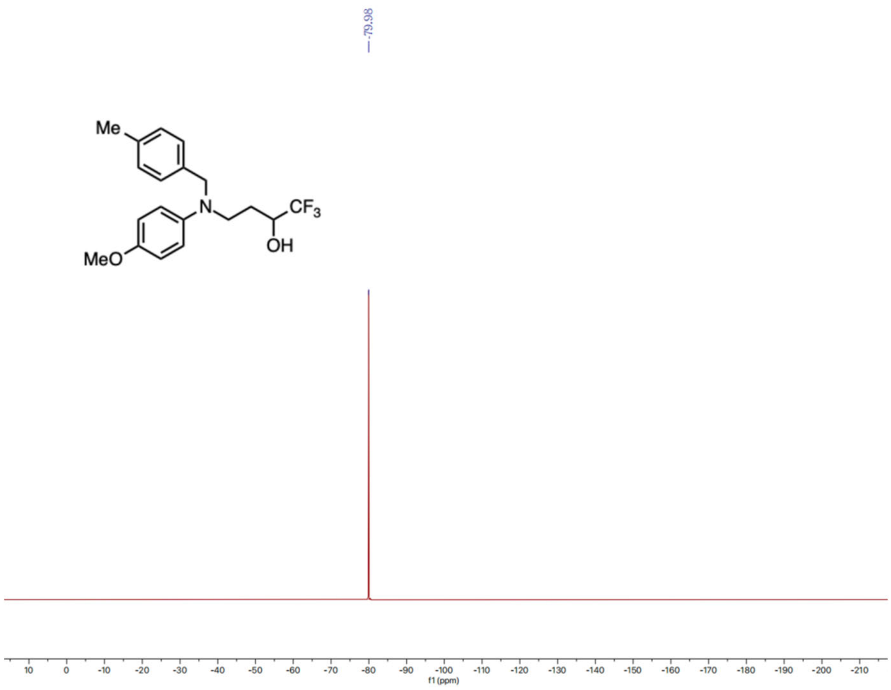

^{19}F NMR spectrum (471 MHz, Chloroform-*d*) of compound **101**

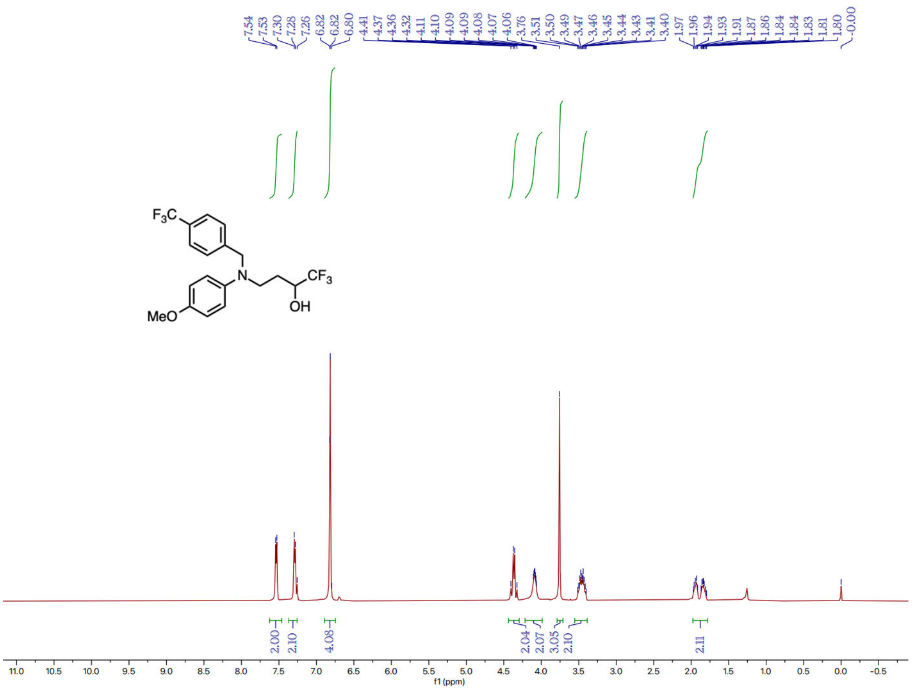

^{1}H NMR spectrum (500 MHz, Chloroform-*d*) of compound **102**

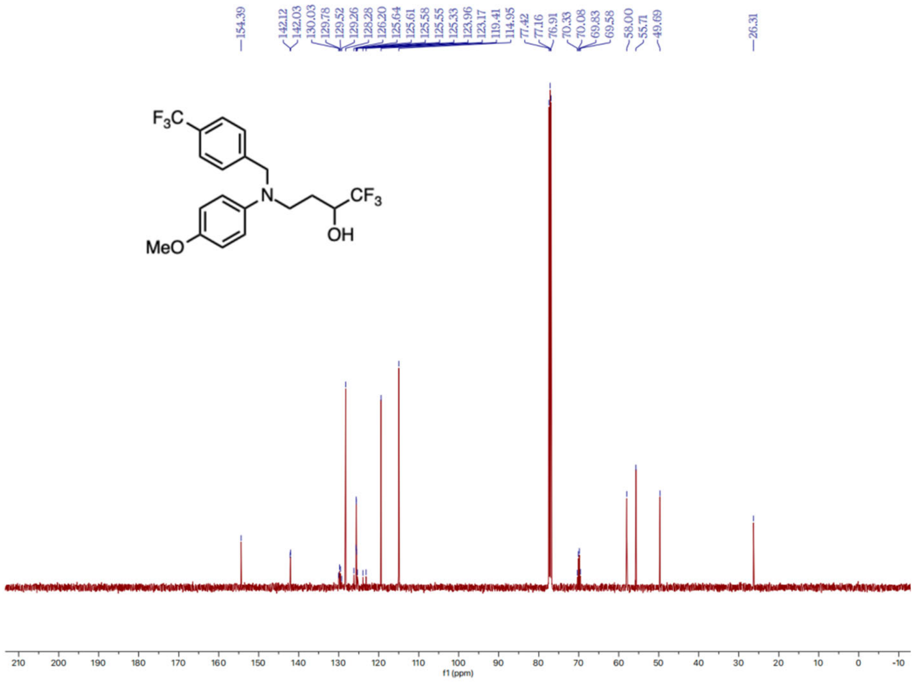

^{13}C NMR spectrum (126 MHz, Chloroform-*d*) of compound **102**

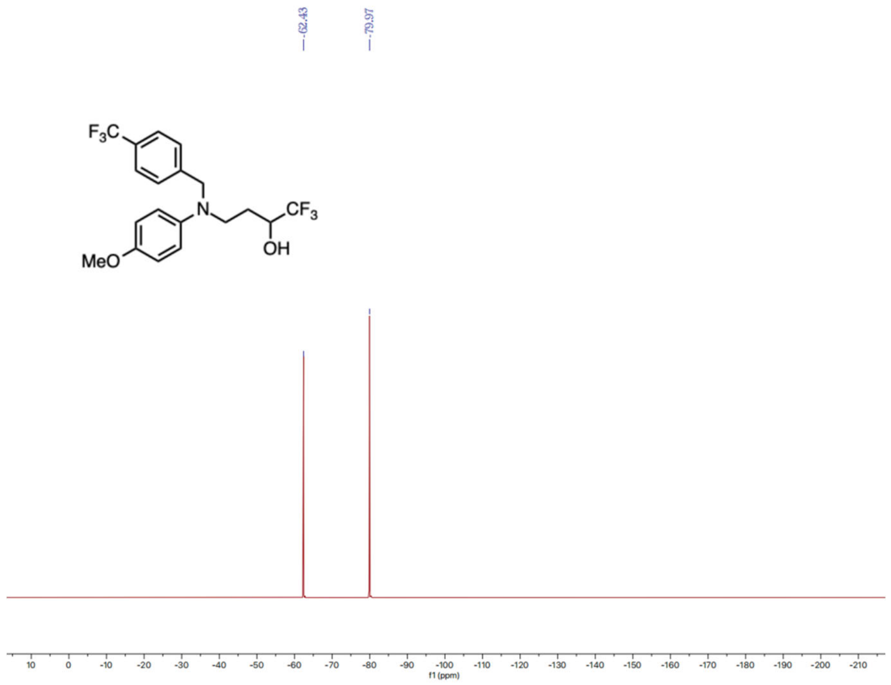

^{19}F NMR spectrum (471 MHz, Chloroform-*d*) of compound **102**

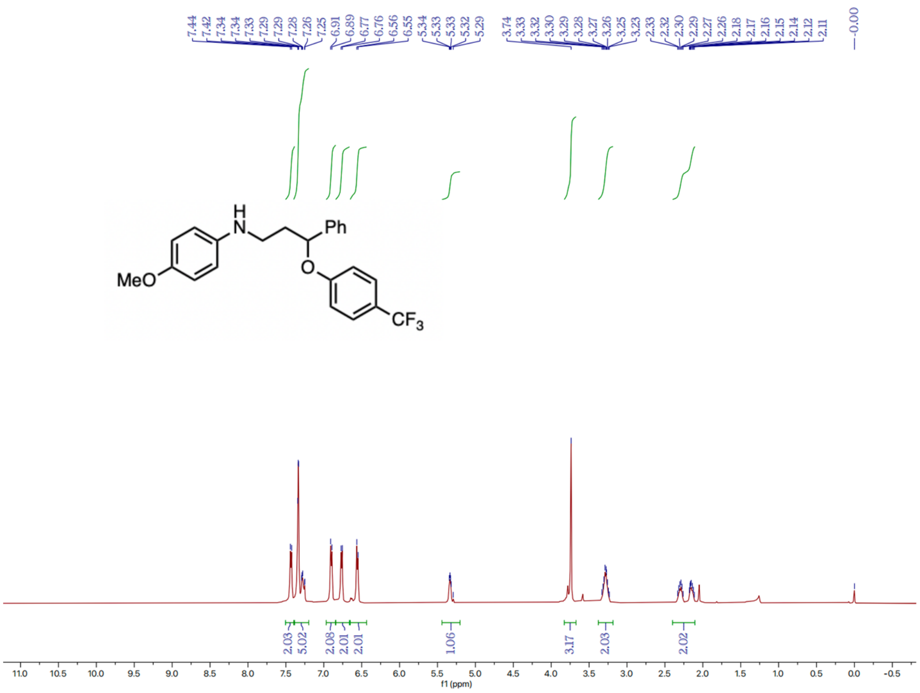

^{1}H NMR spectrum (500 MHz, Chloroform-*d*) of compound **103**

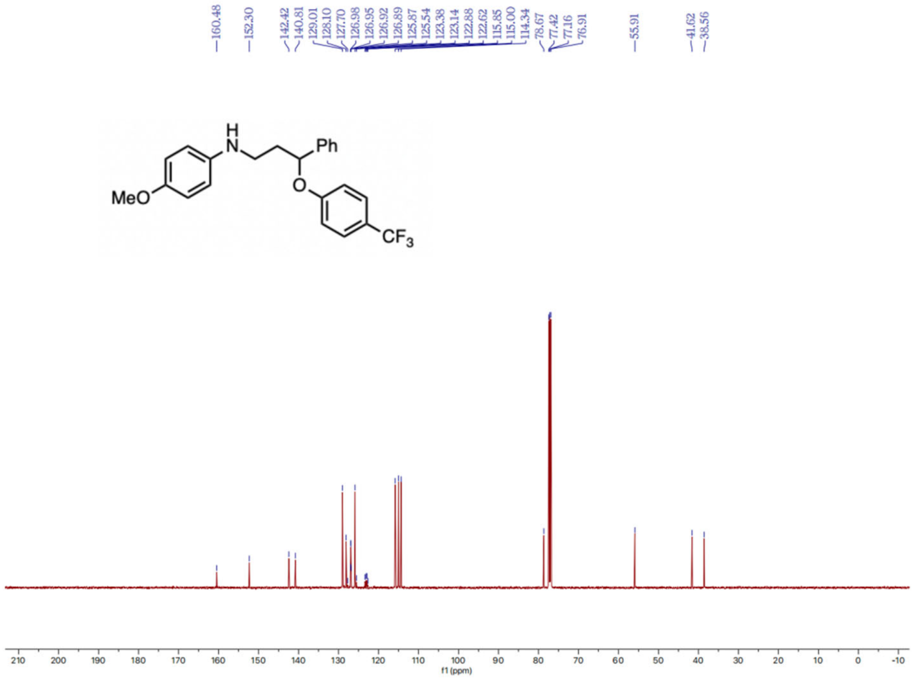

^{13}C NMR spectrum (126 MHz, Chloroform-*d*) of compound **103**

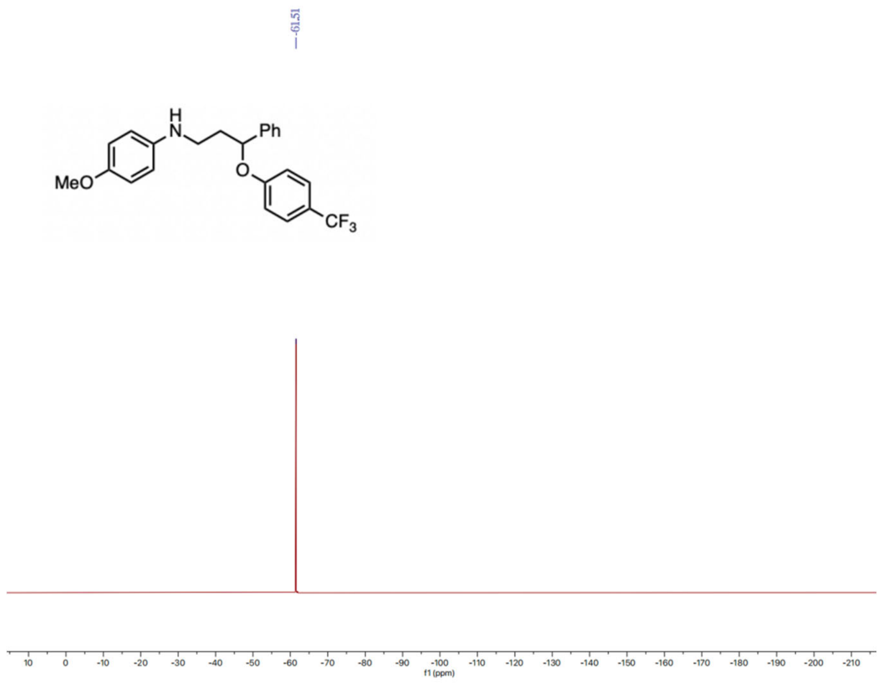

^{19}F NMR spectrum (471 MHz, Chloroform-*d*) of compound **103**

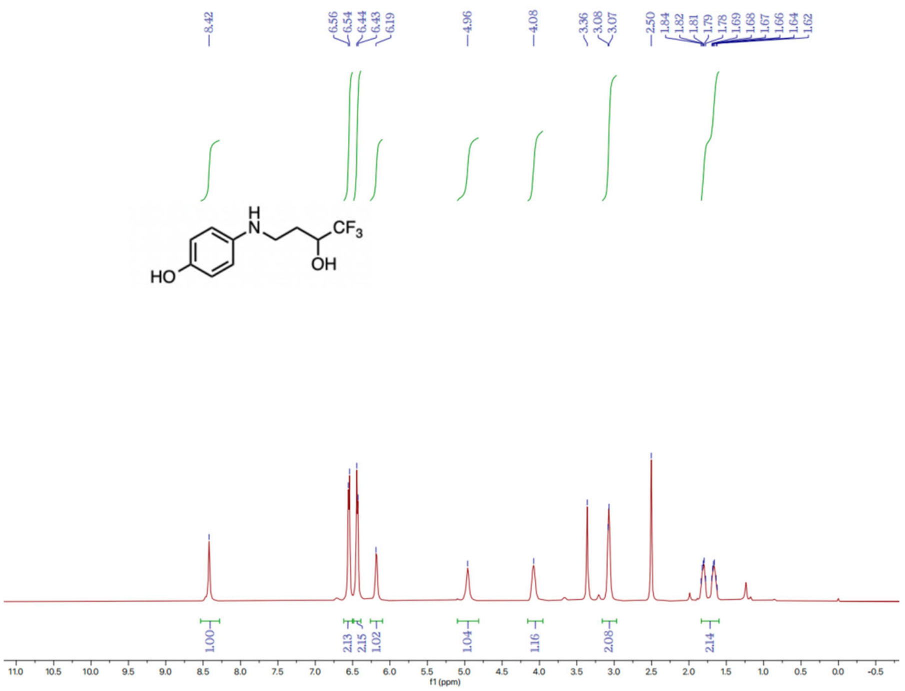

^{1}H NMR spectrum (500 MHz, DMSO-d_6) of compound **104**

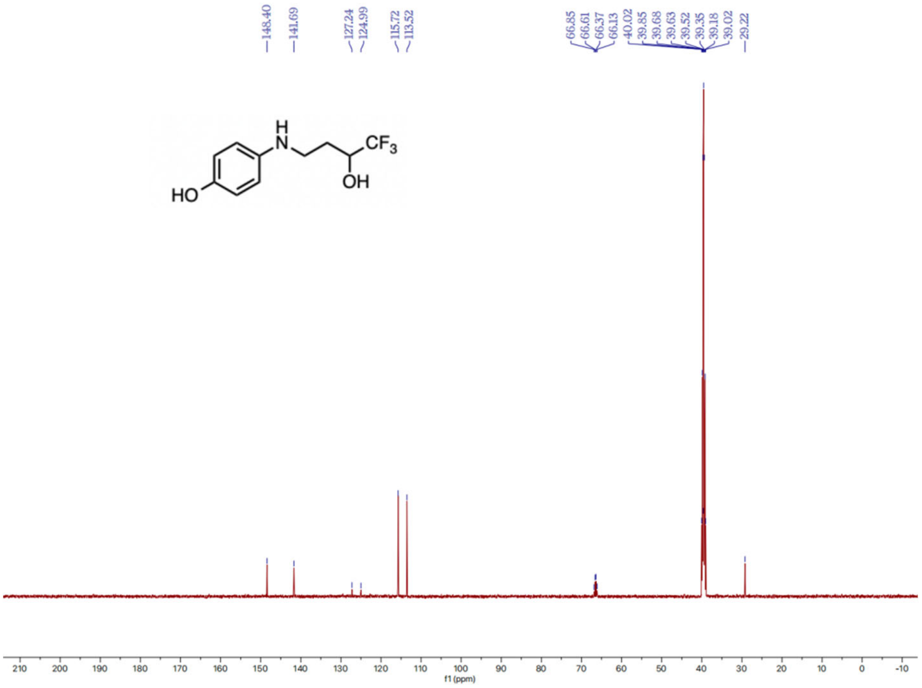

^{13}C NMR spectrum (126 MHz, DMSO-d_6) of compound **104**

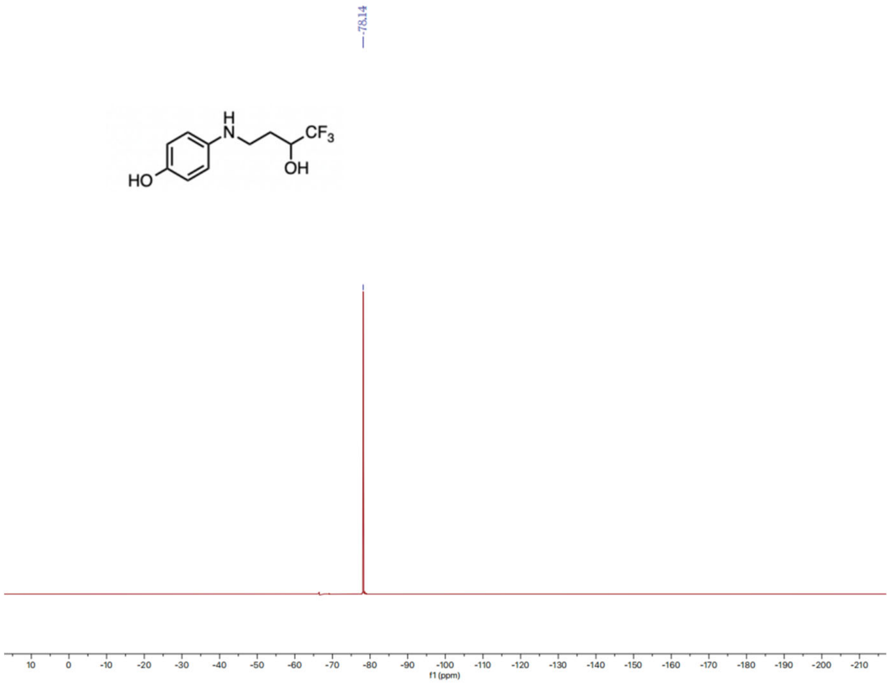

^{19}F NMR spectrum (471 MHz, DMSO-d_6) of compound **104**

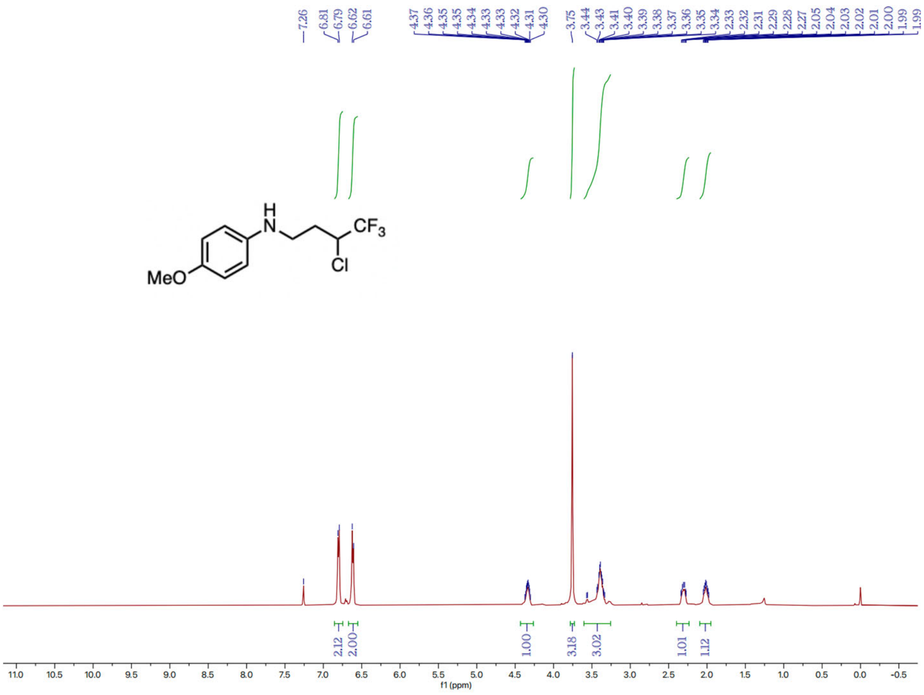

^{1}H NMR spectrum (500 MHz, Chloroform-*d*) of compound **105**

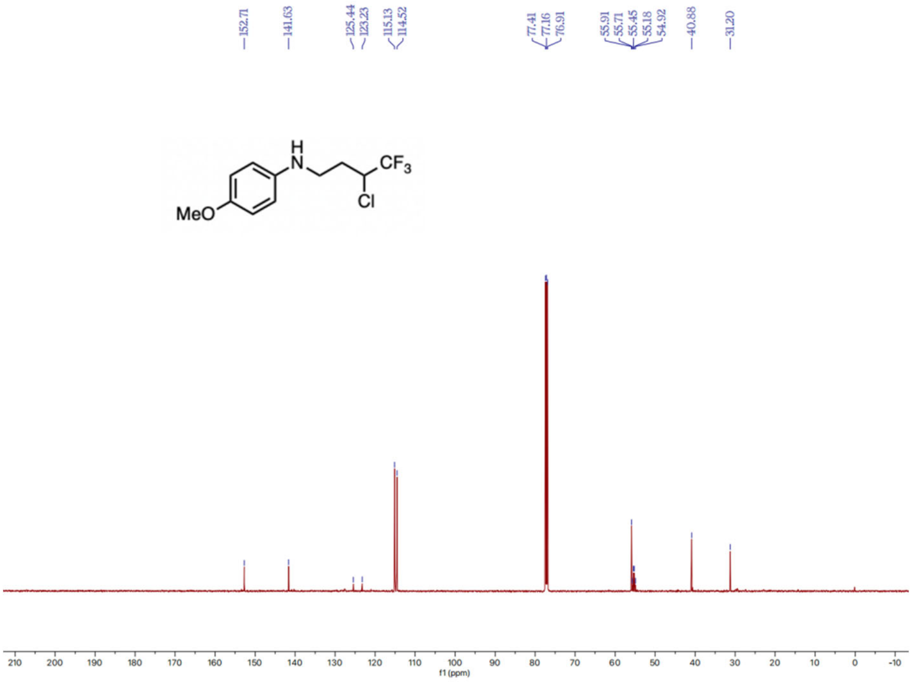

^{13}C NMR spectrum (126 MHz, Chloroform-*d*) of compound **105**

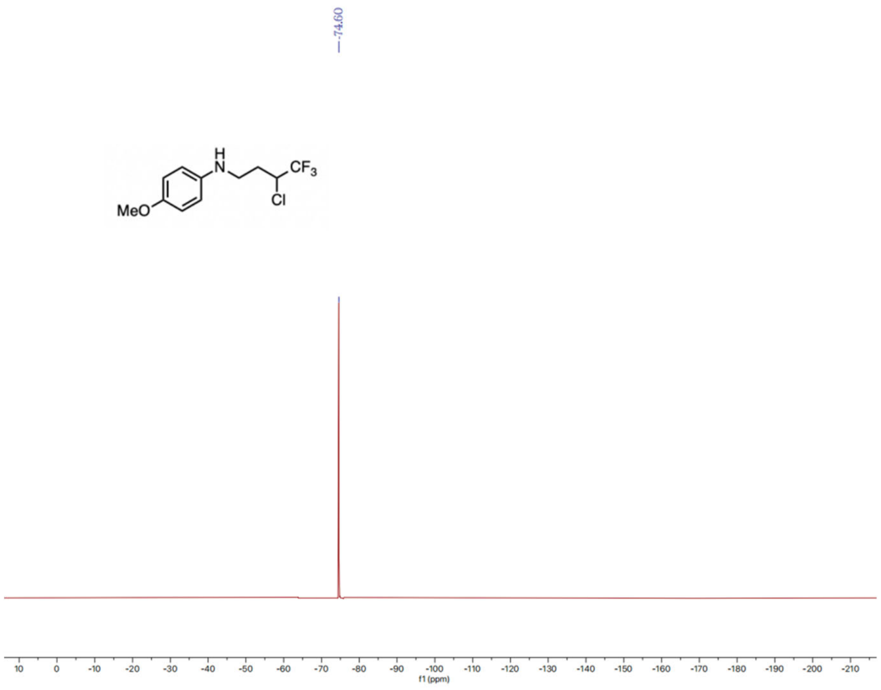

^{19}F NMR spectrum (471 MHz, Chloroform-*d*) of compound **105**

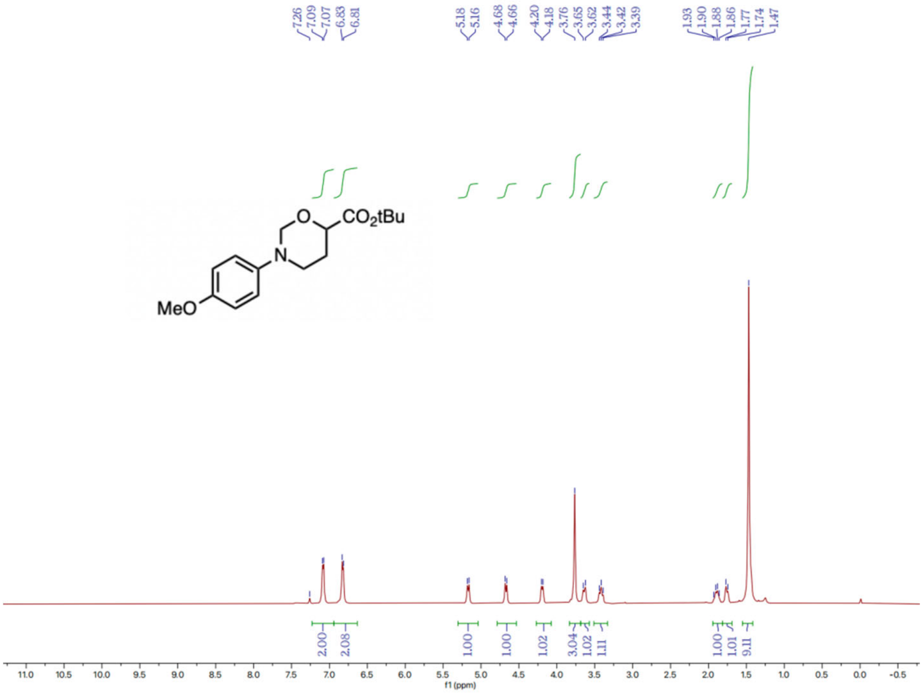

^{1}H NMR spectrum (500 MHz, Chloroform-*d*) of compound **106**

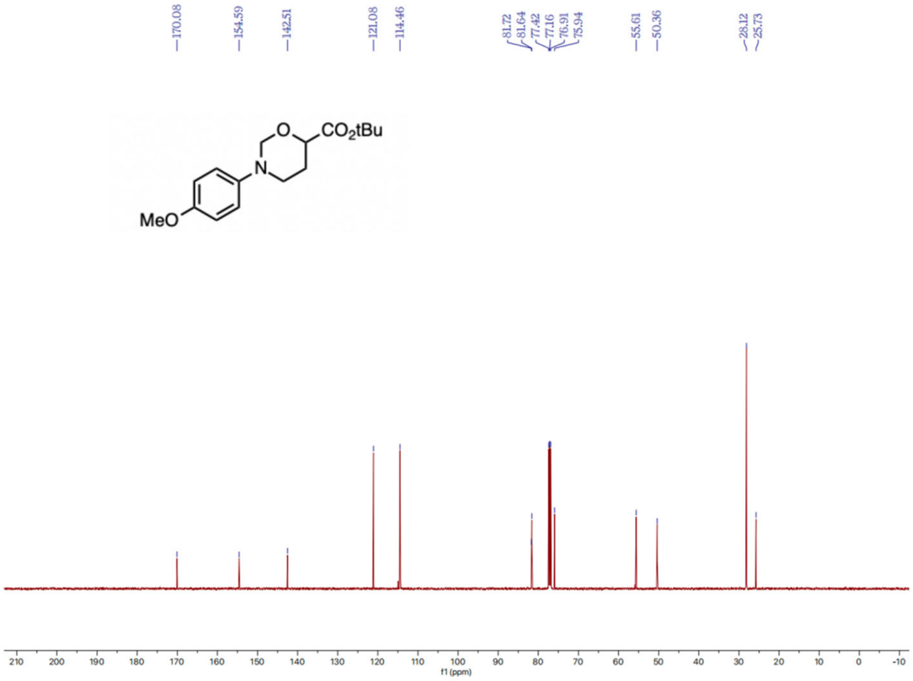

^{13}C NMR spectrum (126 MHz, Chloroform-*d*) of compound **106**

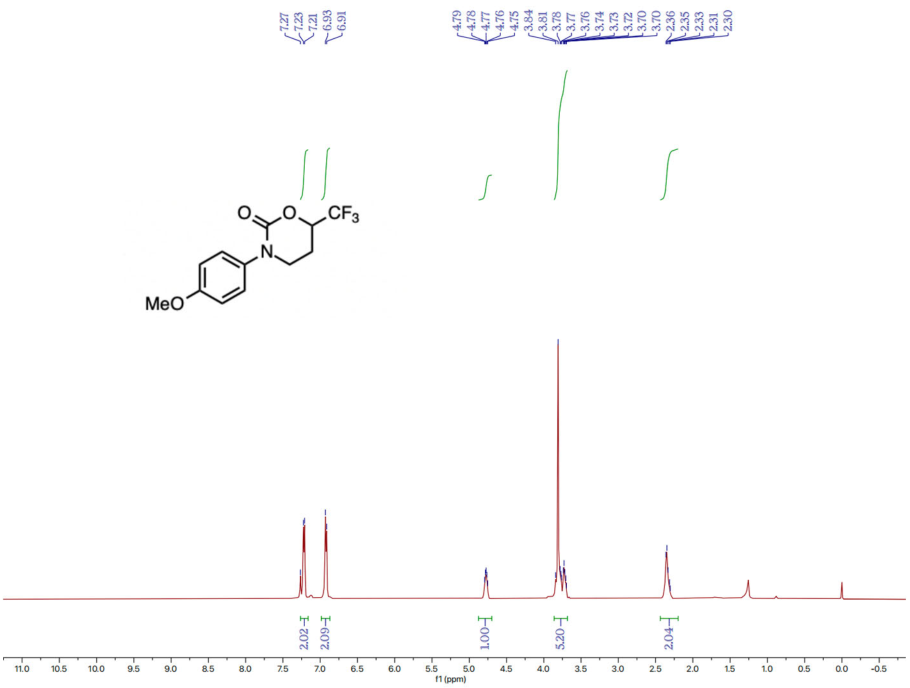

^{1}H NMR spectrum (500 MHz, Chloroform-*d*) of compound **107**

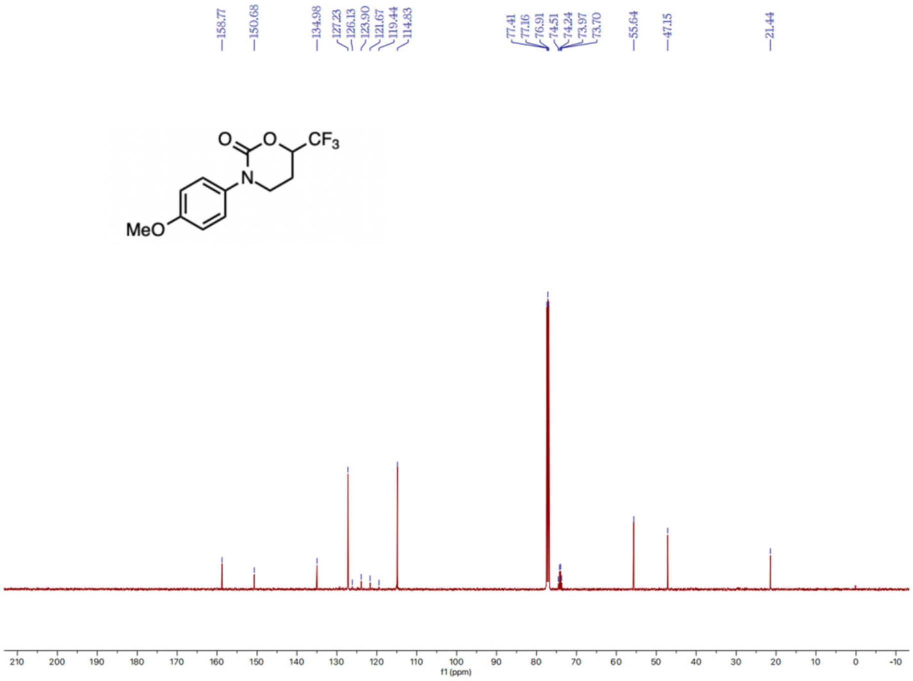

^{13}C NMR spectrum (126 MHz, Chloroform-*d*) of compound **107**

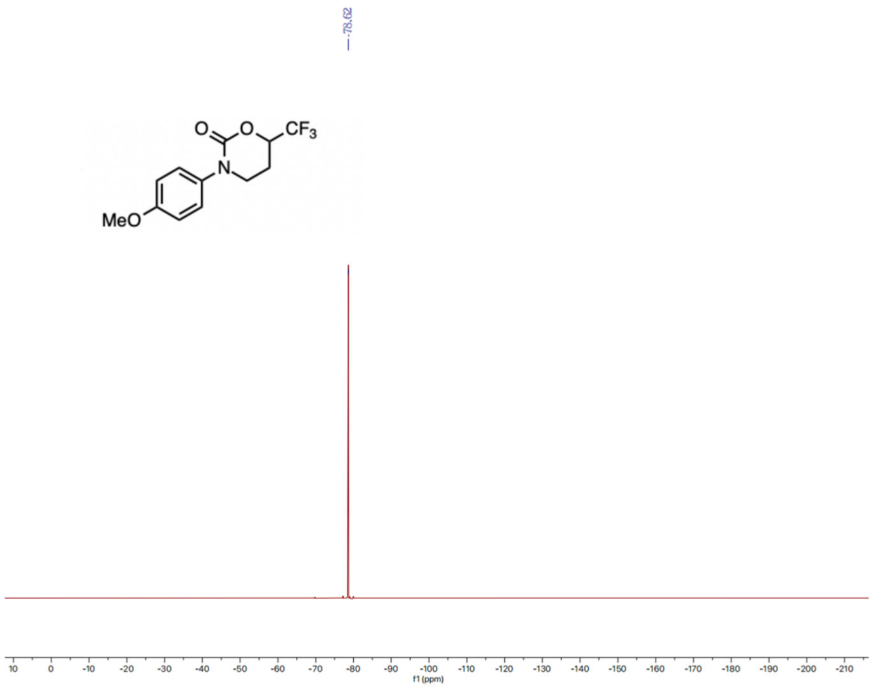

^{19}F NMR spectrum (471 MHz, Chloroform-*d*) of compound **107**

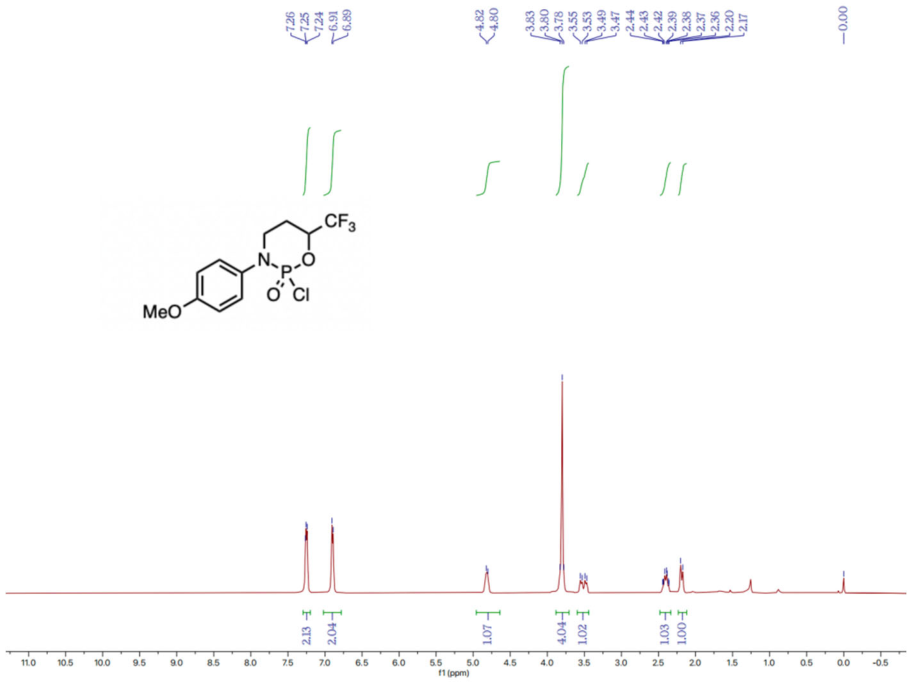

^{1}H NMR spectrum (500 MHz, Chloroform-*d*) of compound **108**

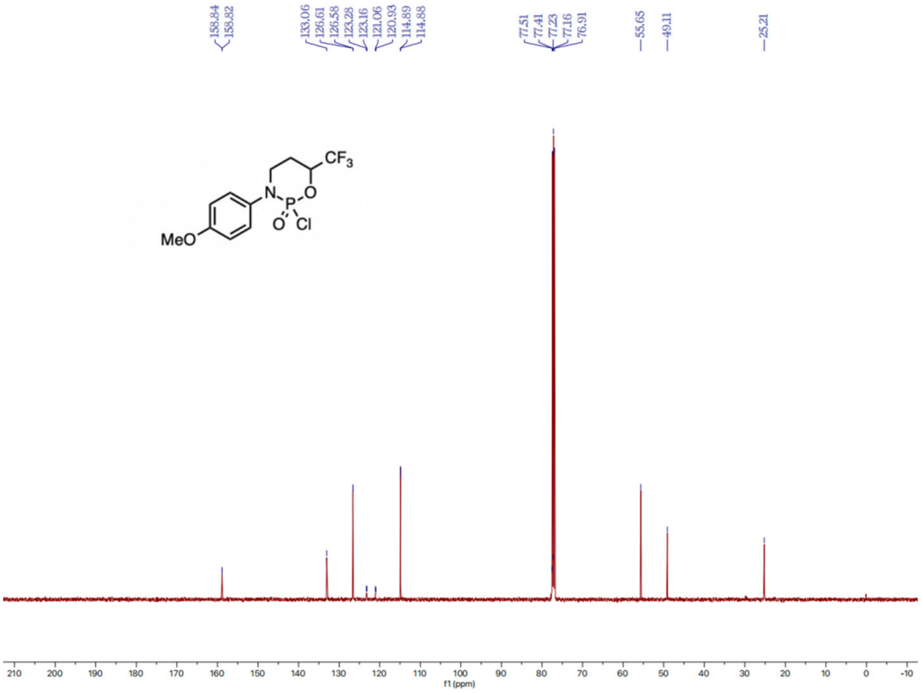

^{13}C NMR spectrum (126 MHz, Chloroform-*d*) of compound **108**

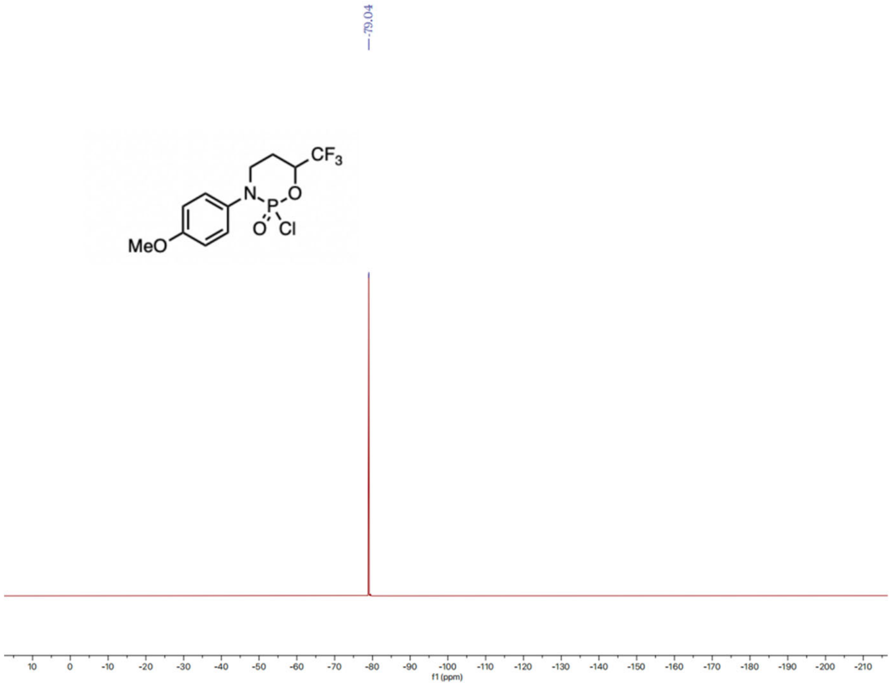

^{19}F NMR spectrum (471 MHz, Chloroform-*d*) of compound **108**

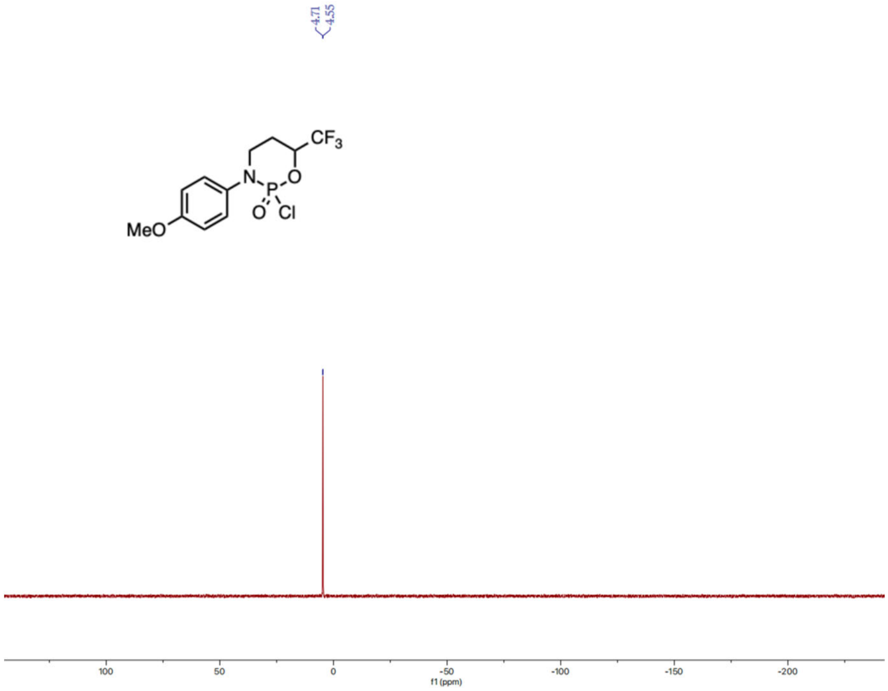

^{31}P NMR spectrum (202 MHz, Chloroform-*d*) of compound **108**

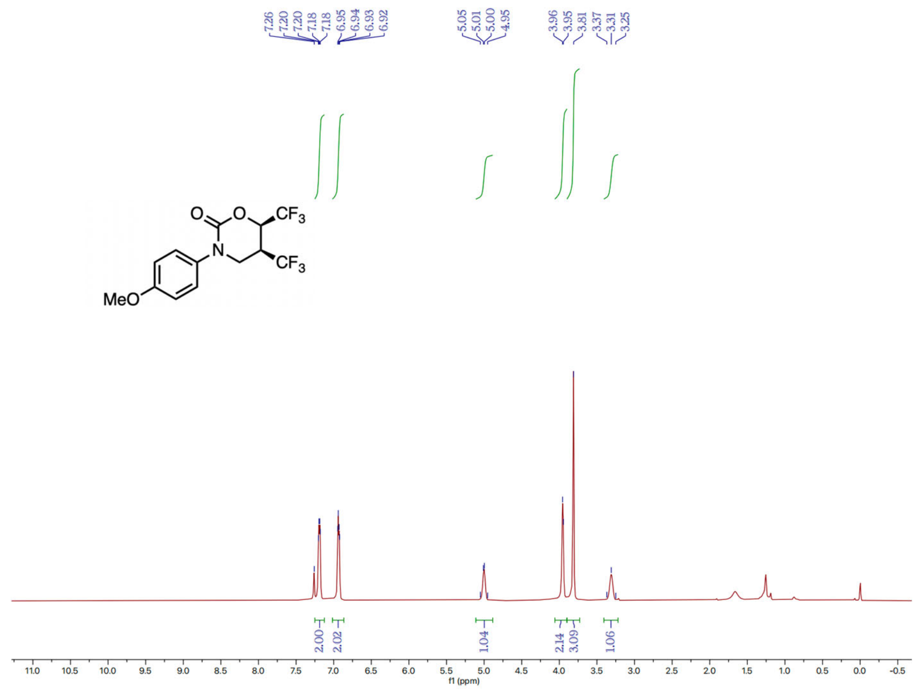

^{1}H NMR spectrum (500 MHz, Chloroform-*d*) of compound **(±)-109**

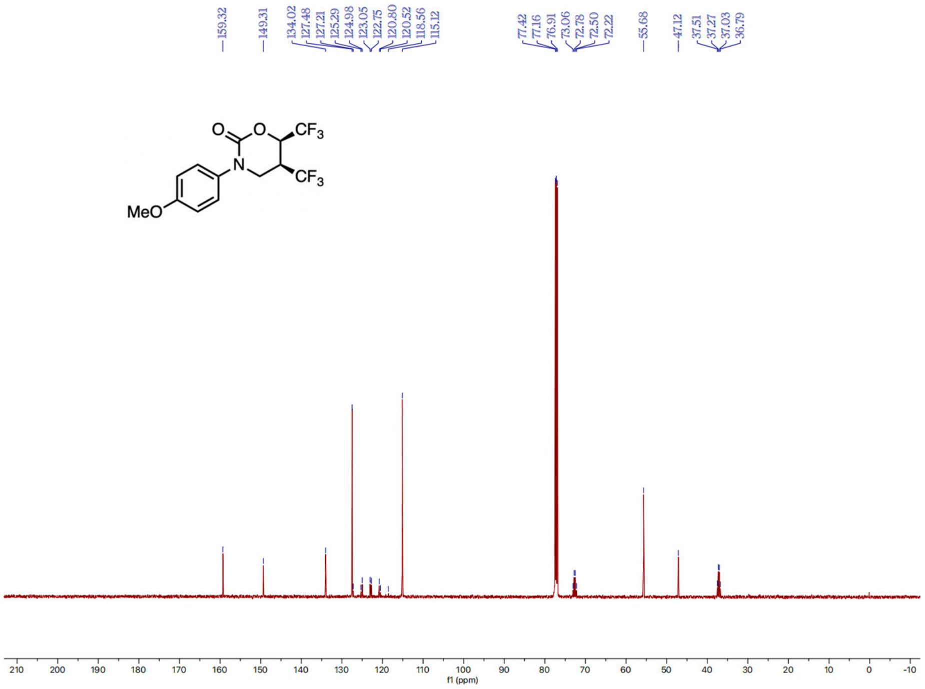

^{13}C NMR spectrum (126 MHz, Chloroform-*d*) of compound **(±)-109**

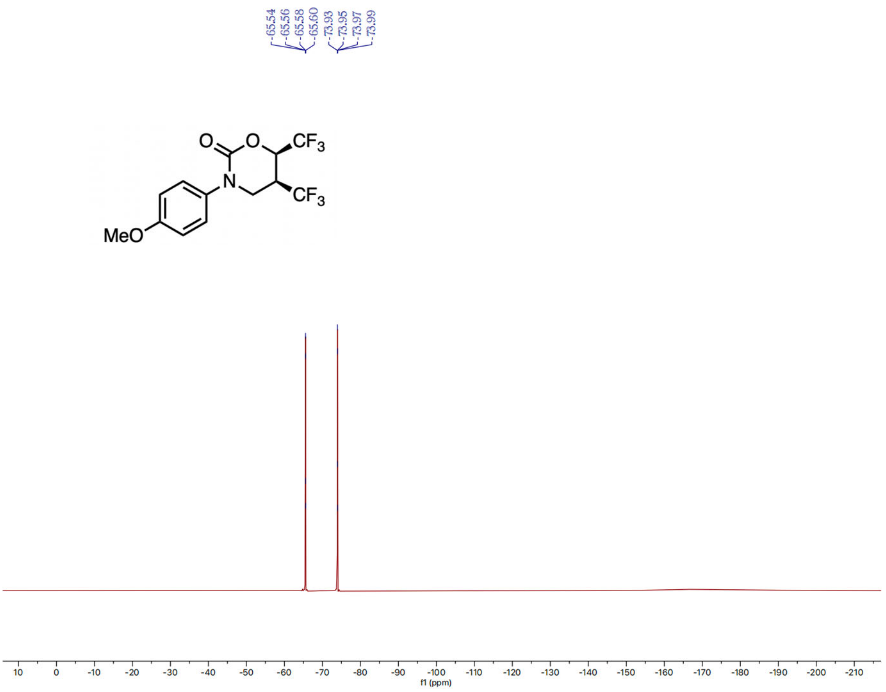

^{19}F NMR spectrum (471 MHz, Chloroform-*d*) of compound **(±)-109**

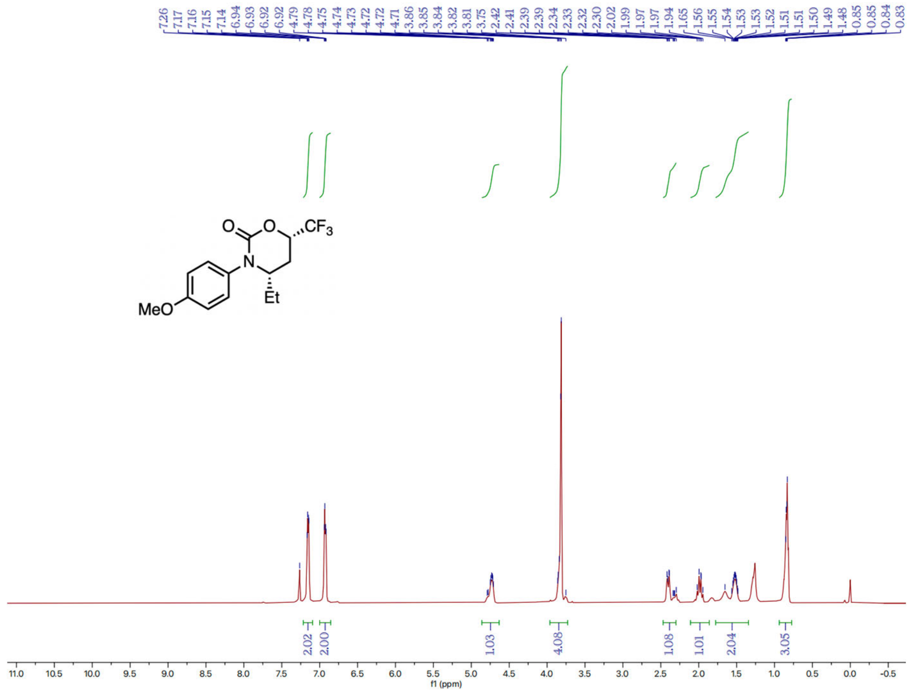

^{1}H NMR spectrum (500 MHz, Chloroform-*d*) of compound **(±)-110**

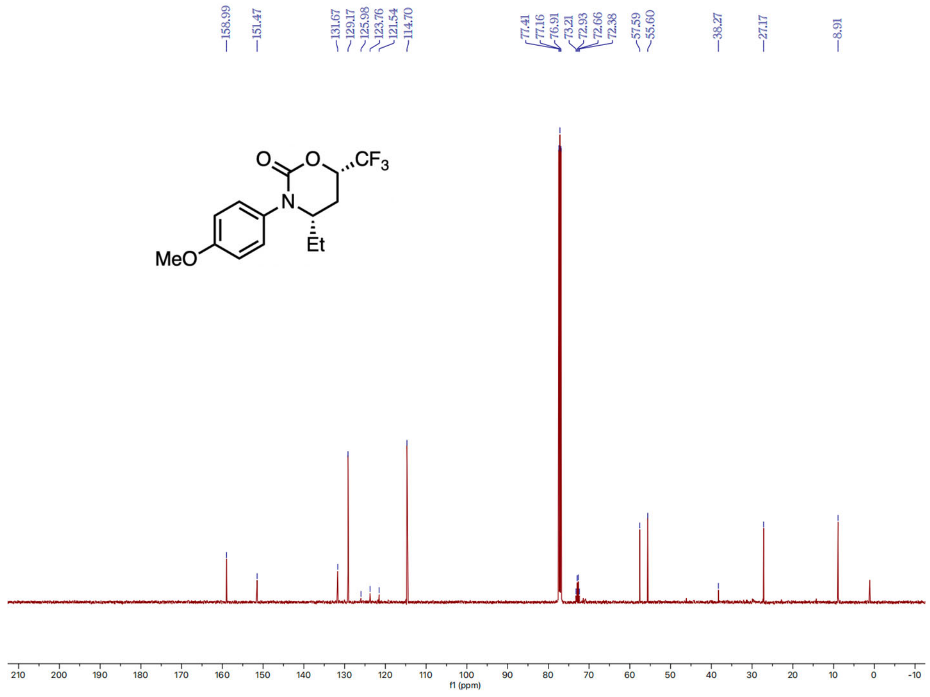

^{13}C NMR spectrum (126 MHz, Chloroform-*d*) of compound **(±)-110**

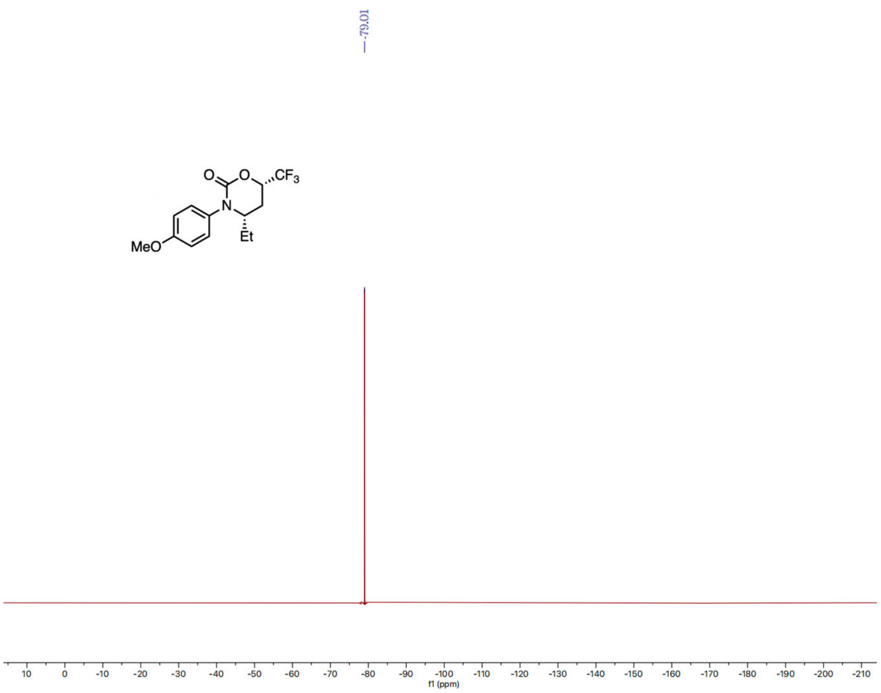

^{19}F NMR spectrum (471 MHz, Chloroform-*d*) of compound **(±)-110**

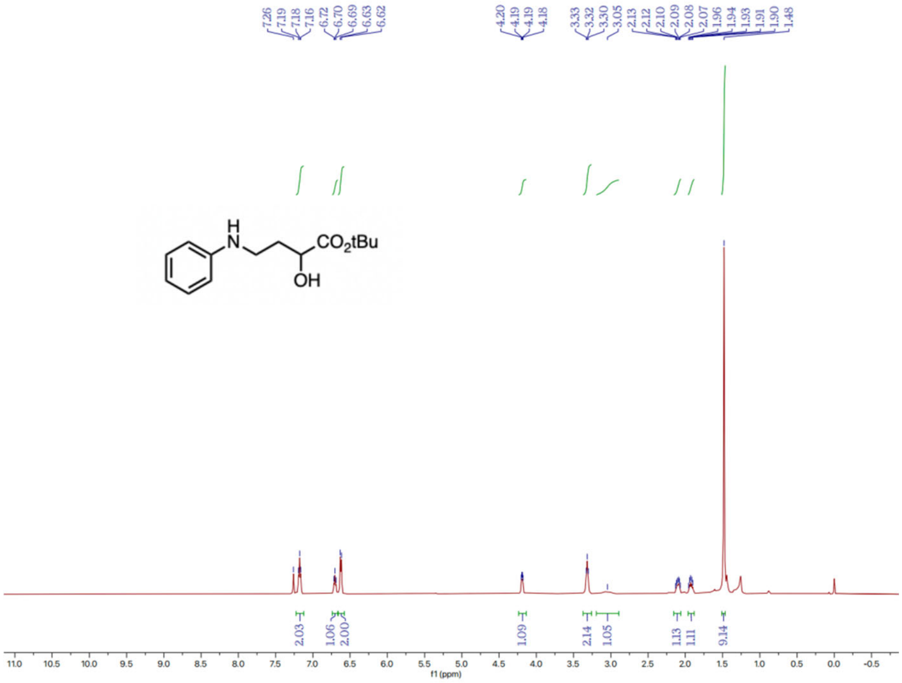

^{1}H NMR spectrum (500 MHz, Chloroform-*d*) of compound **113**

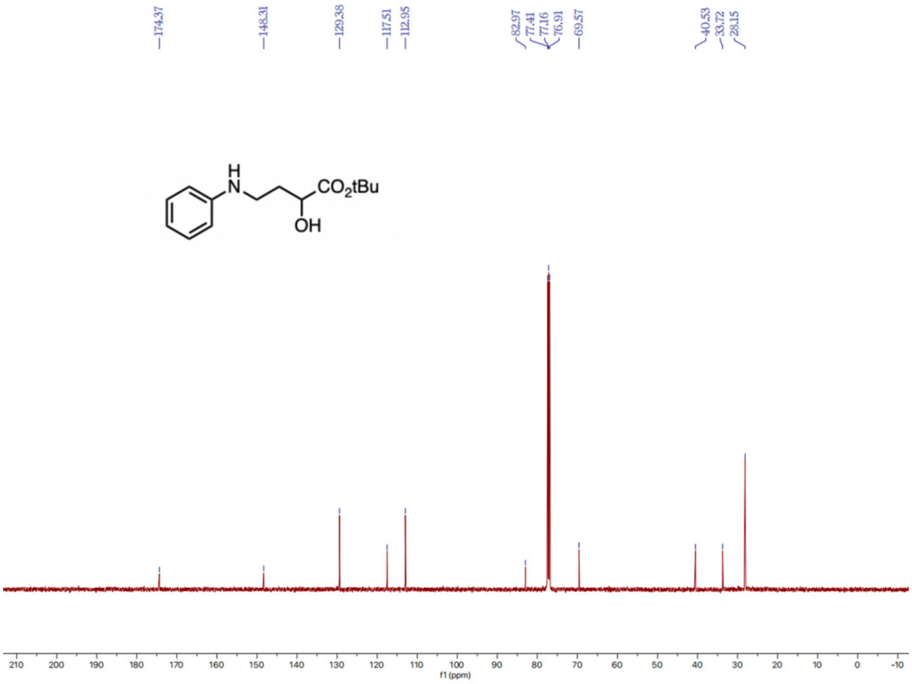

^{13}C NMR spectrum (126 MHz, Chloroform-*d*) of compound **113**

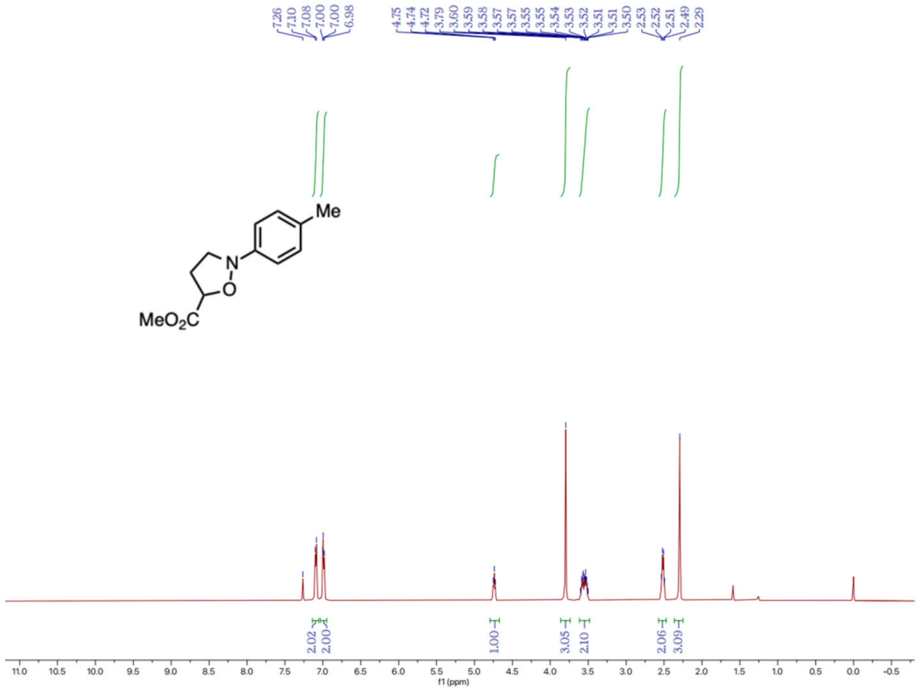

^{1}H NMR spectrum (500 MHz, Chloroform-*d*) of compound **114**

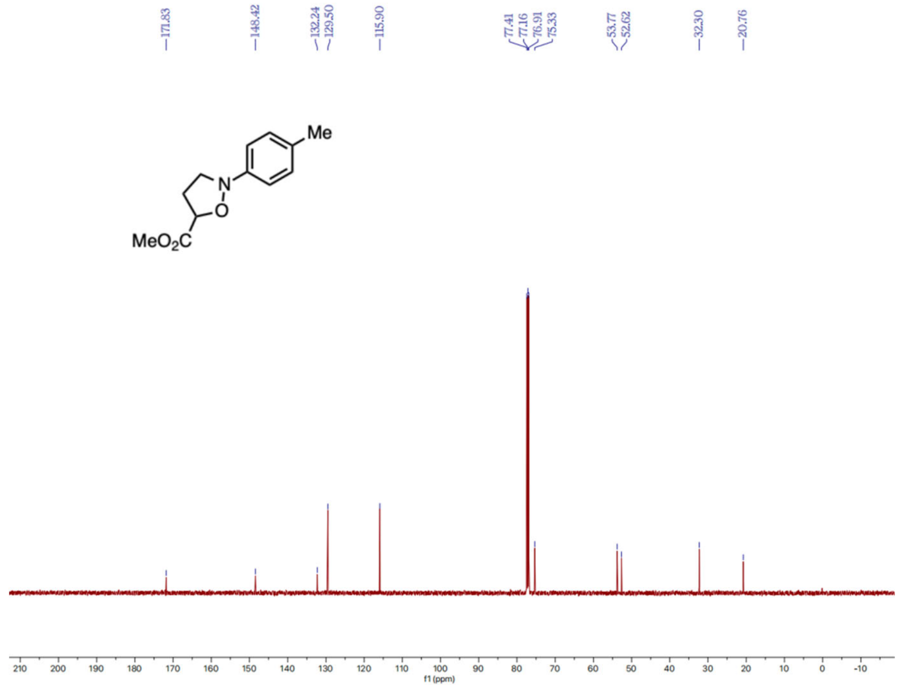

^{13}C NMR spectrum (126 MHz, Chloroform-*d*) of compound **114**

References

[1] Xia, P. J., Song, D., Ye, Z. P., Hu, Y. Z., Xiao, J. A., Xiang, H. Y., Chen, X. Q. &. Yang, H. Photoinduced Single-Electron Transfer as an Enabling Principle in the Radical Borylation of Alkenes with NHC–Borane. *Angew. Chem. Int. Ed.* **59**, 6706 –6710 (2020).

[2] López, M. M., Jamey, N., Pinet, A., Figadère, B. & Ferrié, L. Oxidative Ring. Expansion of Cyclobutanols: Access to Functionalized 1,2-Dioxanes. *Org. Lett.* **23**, 1626-1631 (2021).

[3] Guo, J., Xie, Y., Zeng, W. T., Wu, Q. L., Weng, J., Lu, G. Visible-Light Catalyzed. [1+2+2] Cycloaddition Reactions Enabled by the Formation of Methylene Nitrones. *Adv. Synth. Catal.* ***362***, 5450– 5456 (2020).

[4] Shi, J. B., Wang, Y. B., Bu, Q. Q., Liu, B. Y., Dai, B. & Liu, N. Cr-Catalyzed Direct *ortho*-Aminomethylation of Phenols. *J. Org. Chem*. **86**, 17567−17580 (2021).

[5] De, S., Ghosh, S., Bhunia, S., Sheikh, J. A. & Bisai, A. Intramolecular direct dehydrohalide coupling promoted by kotbu: total synthesis of amaryllidaceae alkaloids anhydrolycorinone and oxoassoanine. *Org. Lett.* **14**, 4466–4469 (2012).

[6] Bharadwaj, K. C. Chemoselective intramolecular morita–baylis–hillman reaction; acrylamide and ketone as sluggish reacting partners on a labile framework. *J. Org. Chem.* **89**, 1073−1082 (2024).

[7] Zhang, X. Q., Xu, T. Y., Zhang, C. D., Wang, C. & Wang, Y. L. Gold-catalyzed. formal (3 + 2) and (4 + 2) cycloadditions of alkynes to highly functionalized dihydropyrroles and tetrahydropyridines. *Org. Chem.Front.* **10**, 680–685 (2023).

[8] Chiba, k., Asanuma, M., Ishikawa, M., Hashimoto, Y., Dodo, K., Sodeoka, M., &. Yamaguchi, T. Specific fluorescence labeling of target proteins by using a ligand–4-azidophthalimide conjugate. *Chem. Commun.* **53**, 8751−8754 (2017).

[9] Guillén, M., Leutzsch, M. & List, B. Catalytic Asymmetric Cycloaddition of. Olefins with In Situ Generated *N*-Boc-Formaldimine. *J. Am. Chem. Soc.* **47**, 32292–32297 (2024).

[10] Fujii, S., Kikuchi,E., Watanabe, Y., Suzuyama, H. & Kagechika, H. Structural. development of *N*-(4-phenoxyphenyl)benzamide derivatives as novel SPAK inhibitors blocking WNK kinase signaling. *Bioorg. Med. Chem. Lett.* **30**, 127408 (2020).

[11] Shi, C., Liu, R. H., Wang, Z. M., Gao, C. X., Chen, J. S., Qin, H. Y., Shan, W. L., Zhuang, W. L., Zhou, N., Li, X. Q. & Shi, D. Y. Anaerobic 1,2-/1,3-hydroxytrifluoromethylation of unactivated alkenes enabled by photoexcited nitroarenes. *Org. Lett.* **27**, 922–926 (2025).

[12] Menche, D., Arikan, F., Li, J. & Rudolph, S. Directed Reductive Amination of β-Hydroxy-Ketones: Convergent Assembly of the Ritonavir/Lopinavir Core. *Org. Lett.* **9**, 267–270 (2007).

[13] Yang, X., Cheng, F., Kou, Y. D., C., Pang, S., Shen, Y. C., Huang, Y. Y. & Shibata, N. Catalytic Asymmetric 1,3-Dipolar Cycloaddition of β-Fluoroalkylated α, β-Unsaturated 2-Pyridylsulfones with Nitrones for Chiral Fluoroalkylated Isoxazolidines and γ-Amino Alcohols. *Angew. Chem. Int. Ed.* **56**, 1510–1514 (2017).

[14] Andrew, T. L., & Swager, T. M. Detection of Explosives via Photolytic Cleavage of Nitroesters and Nitramines. *J. Org. Chem.* **76**, 2976–2993 (2011).
